# Genomics of *Yoonia* sp. Isolates (Family *Roseobacteraceae*) from Lake Zhangnai on the Tibetan Plateau

**DOI:** 10.3390/microorganisms11112817

**Published:** 2023-11-20

**Authors:** Xiaoyuan Feng, Peng Xing

**Affiliations:** 1 Shenzhen Research Institute, Chinese University of Hong Kong, Shenzhen 518000, China; asdfeng188@gmail.com; 2 State Key Laboratory of Lake Science and Environment, Nanjing Institute of Geography and Limnology, Chinese Academy of Sciences, Nanjing 210008, China

**Keywords:** *Roseobacteraceae*, *Yoonia*, Tibetan Plateau lakes, habitat transition, extreme environment adaptation

## Abstract

Understanding the genomic differentiation between marine and non-marine aquatic microbes remains a compelling question in ecology. While previous research has identified several lacustrine lineages within the predominantly marine *Roseobacteraceae* family, limited genomic data have constrained our understanding of their ecological adaptation mechanisms. In this study, we isolated four novel *Yoonia* strains from a brackish lake on the Tibetan Plateau. These strains have diverged from their marine counterparts within the same genus, indicating a recent habitat transition event from marine to non-marine environments. Metabolic comparisons and ancestral genomic reconstructions in a phylogenetic framework reveal metabolic shifts in salinity adaptation, compound transport, aromatics degradation, DNA repair, and restriction systems. These findings not only corroborate the metabolic changes commonly observed in other non-marine Roseobacters but also unveil unique adaptations, likely reflecting the localized metabolic changes in responses to Tibetan Plateau environments. Collectively, our study expands the known genomic diversity of non-marine *Roseobacteraceae* lineages and enhances our understanding of microbial adaptations to lacustrine ecosystems.

## 1. Introduction

Although aquatic environments exhibit many shared ecological parameters, microbial transitions between marine and non-marine habitats are considered to be infrequent events [1]. As a result, microbes in these two types of aquatic systems are often phylogenetically distinct and often segregate into well-defined marine and non-marine clusters [2]. Notable exceptions to this pattern at the species level are scarce, with *Escherichia coli* serving as one of the few examples detected in both aquatic ecosystems. Instead, the transition from marine to lacustrine environments has been observed in several microbial lineages at higher taxonomic levels, such as *Nitrososphaerales* [3], SAR11 [4], *Methylophilaceae* [5], *Cyanobacteria* [6], and *Flavobacteriaceae* [7]. In support of this, studies have identified significant differences in isoelectric points (pI) at the level of global amino acid composition between marine and lacustrine microbes, suggesting that a considerable evolutionary timescale is requisite for such transitions [8]. Furthermore, these transition events are often accompanied by both genomic and phenotypic adaptations, encompassing alterations in salinity tolerance, transport functions, and environmental information processing [9].

The alphaproteobacterial Roseobacter group provides another illustrative example of ecological transitions between marine and lacustrine habitats. Comprising up to 20% of bacterial communities in coastal areas and 3–5% in pelagic oceans, the Roseobacter group plays a pivotal role in global carbon and sulfur cycling [10]. Recent taxonomic revisions have reclassified some marine Roseobacters into a novel *Roseobacteraceae* family, while their non-marine counterparts such as *Paracoccus* and *Rhodobacter* have been categorized under the *Rhodobacteraceae* family [11]. Intriguingly, non-marine lineages like *Rubellimicrobium*, *Ketogulonicigenium* [12], *Loktanella*, and *Yoonia* [13] are also phylogenetically placed within the *Roseobacteraceae* family. Phylogenetic analyses reveal a mixed structure between marine *Roseobacteraceae* and their non-marine relatives, thereby offering an ideal setting for studying microbial transitions between marine and non-marine aquatic ecosystems. Non-marine members have evolved high-affinity transporters to cope with lower sulfate concentrations and have lost genes associated with reduced sodium chloride and organohalogen concentrations in their habitats [14]. Additionally, these organisms have lost pathways related to mercury antitoxin, carbon monoxide oxidation, and de novo cobalamin synthesis, which have become largely dispensable in lacustrine environments [14]. Nevertheless, such transitions are rarely reported within the genus level [13].

In this study, we sequenced four novel genomes from Lake Zhangnai (also called Zhangnaicuo or Zhangnai Co) on the Tibetan Plateau. These genomes are taxonomically classified under the marine *Yoonia* genus, suggesting a habitat transition from marine to non-marine ecosystems. Metabolic comparisons and ancestral reconstructions elucidate metabolic shifts in salinity adaptation and compound transport. They further gain genes related to aromatic compound degradation and defense systems, likely reflecting localized adaptations to the unique conditions of the Tibetan Plateau. Our findings uncover a marine-to-lacustrine transition within the genus level, thereby offering a valuable genomic repertoire for advancing our understanding of adaptations to lacustrine environments.

## 2. Methods

### 2.1. Sample Collection, Bacterial Cultivation, and Sequencing

**Figure 1:**
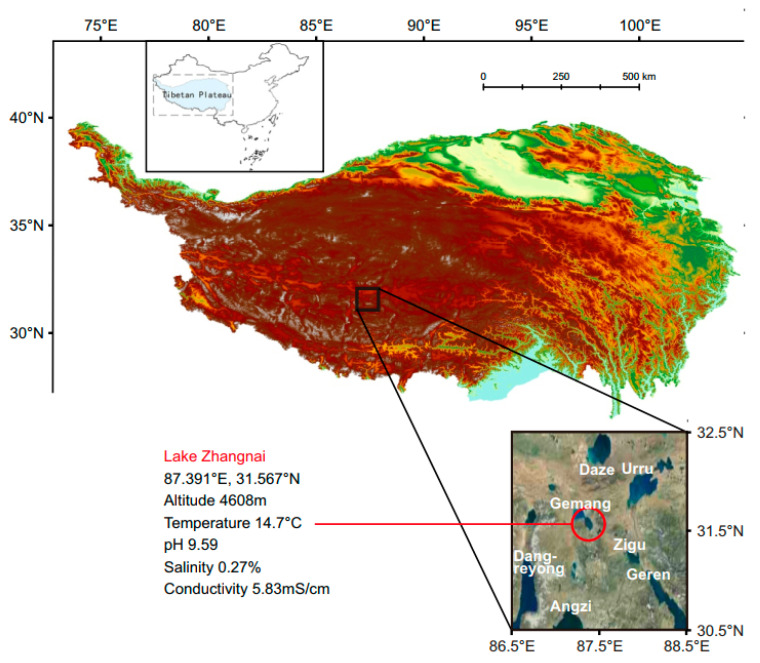
Geographical location and ecological characteristics of Lake Zhangnai on the Tibetan Plateau.

Lake Zhangnai is situated on the Tibetan Plateau with a maximal aquatic depth of 2.7 m (Figure 1). Water temperature, conductivity, salinity, and pH were measured using a 6600 Multi-Parameter Water Quality Sonde (YSI Inc., Yellow Springs, OH, USA). The lake surface waters were collected in 2017 and subjected to pre-filtration using a 20 μm mesh to exclude large particles and eukaryotic organisms. Bacterial isolation and genomic sequencing were conducted following our previously established protocol [15]. Briefly, the collected lake water was sprayed onto plates containing modified LB medium consisting of 2 g/L tryptone, 1 g/L yeast extract, 7 g/L NaCl, and 20 g/L agar. The four novel *Yoonia* strains were isolated through serial dilutions and cultured on modified LB plates. Genomic DNA was extracted using the Wizard Genomic DNA Purification Kit (Promega, Madison, WI, USA) and subsequently sequenced on the Illumina Miseq platform (Shanghai Biozeron Biotechnology Co., Ltd., Shanghai, China).

### 2.2. Genomic Assembly and Annotation

Raw reads derived from Illumina sequencing were quality-trimmed using Trimmomatic v0.39 [16] with the parameters ‘SLIDINGWINDOW:4:15 MAXINFO:40:0.9 MINLEN:40′. Assembly was performed using SPAdes v3.14.0 [17] with the ‘-careful’ parameter. Contigs exceeding 1000 bp in length and 5× in sequencing depth were retained for further analysis. The genome size, GC content, and coding density were estimated using CheckM v1.1.3 [18]. To minimize biases from varying gene prediction software employed in previous studies, protein-coding genes were re-predicted using Prokka v1.14.6 [19] for the four novel *Yoonia* isolates and 99 reference Roseobacter genomes. Functional annotation of these protein sequences was conducted against the KEGG database using the BlastKOALA (https://www.kegg.jp/blastkoala/, accessed on in 1 June 2023) [20].

### 2.3. Construction of Phylogenomic Trees

For the construction of Roseobacter phylogeny, a set of 120 single-copy genes (bac120) were identified, aligned, and trimmed (Supplemental Text S1) using GTDB-tk v1.7.0 [21]. These bac120 genes are universally conserved and have undergone minimal recombination events, thus providing a reliable framework for phylogenetic analysis [22]. GTDB-tk identified the bac120 genes through a hidden Markov model (HMM) searching against a defined reference database. The identified gene sequences were then aligned with a pre-existing alignment of over 62,293 bacterial genomes, followed by a trimming process [21]. The phylogenomic tree was built using IQ-TREE v2.2.0 [23], with ModelFinder [24] assigning the most appropriate substitution model for IQ-TREE analysis (LG + R9). A total of 1000 bootstrap replicates were sampled to assess the robustness of the phylogeny. Additionally, the phylogeny was validated using RAxML v8.2.12 [25], with ModelTest-NG [26] assigning the most appropriate substitution model for RAxML analysis (LG + I + G4 + F). Pairwise comparisons of average nucleotide identity (ANI) were computed using FastANI v1.3 [27].

### 2.4. Metabolic Comparisons

The four novel *Yoonia* isolates are postulated to have transitioned from marine environments to lake ecosystems on the Tibetan Plateau. To elucidate the metabolic adaptations accompanying this ecological shift, we reconstructed the metabolic profiles of the ancestral nodes relevant to the *Loktanella*, *Cognatiyoonia*, and *Yoonia* genera and also forecasted KEGG orthology (KO) acquisition and deletion events for each ancestral node. This analysis was performed using BadiRate v1.35 [28] with the parameters ‘-anc-bmodel FR-rmodel BDI-ep CSP’. A pruned phylogenetic subtree and the KO count per genome were employed as input data. The metabolic comparisons were further performed between marine and non-marine lineages within the *Loktanella*, *Cognatiyoonia*, and *Yoonia* genera by using the ‘binaryPGLMM’ function in the ‘ape’ R package v5.6 [29].

## 3. Results and Discussion

### 3.1. Genomic Characterization of Novel Roseobacteraceae Strains

**Figure 2:**
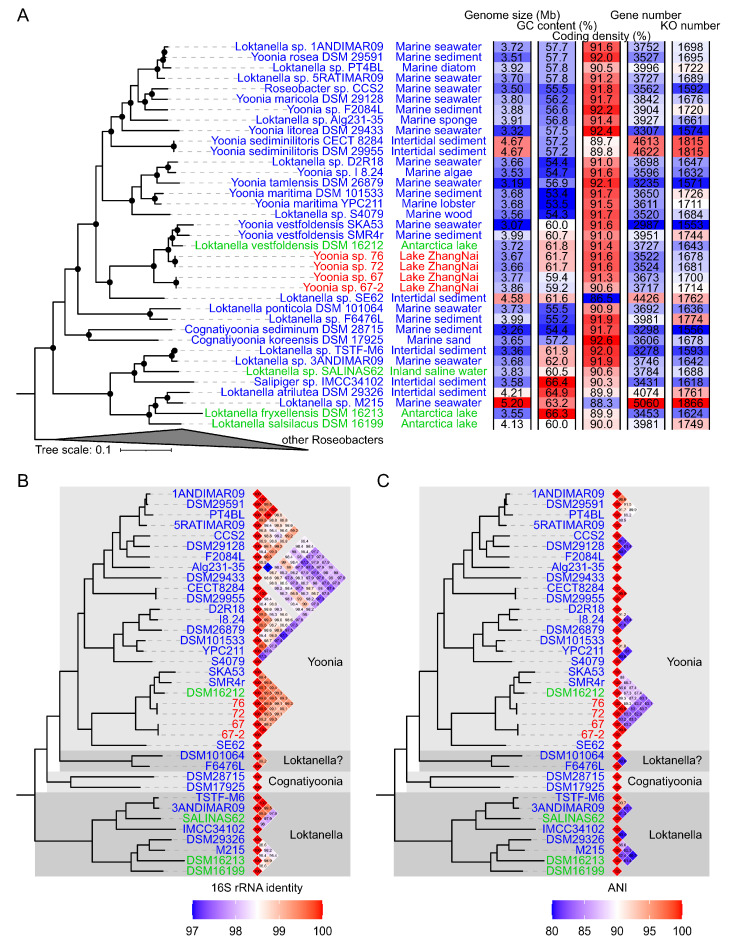
Phylogenomic placement of novel *Yoonia* strains. The phylogenomic tree was constructed based on 120 conserved single-copy genes using IQ-TREE v2.2.0 with the ‘LG + R9′ substitution model. Nodes with bootstrap support exceeding 95% are indicated by solid circles. (Panel **A**) presents the Roseobacter phylogeny. Sampling locations of *Loktanella*, *Cognatiyoonia*, and *Yoonia* genomes are marked next to their genome IDs. Isolates sampled from Lake Zhangnai on the Tibetan Plateau, lacustrine environments, and marine environments are colored in red, green, and blue in their genome IDs, respectively. (Panel **B**) shows the pairwise comparisons of 16S rRNA gene similarities among *Yoonia* and *Loktanella* genomes. Genus reassignments [13] are annotated alongside the similarity heatmaps. (Panel **C**) illustrates the pairwise comparisons of average nucleotide identities (ANIs) with values below 80% not shown.

Four novel *Roseobacteraceae* strains were isolated in 2017 from the surface waters of Lake Zhangnai on the Tibetan Plateau (Figure 1). These strains were taxonomically classified within the *Yoonia* genus (Figure 2A), which was a recently reclassified genus originally part of the *Loktanella* genus [13]. The phylogenetic topology of these strains was confirmed using both IQ-TREE v2.2.0 (Figure 2A) and RAxML v8.2.12 (Figure S1). It is worth noting that many published *Roseobacteraceae* strains have been misidentified within their genus classification due to the poor resolution of 16S rRNA genes in the *Roseobacteraceae* phylogeny [13]. To mitigate this issue, we used the genome IDs from the NCBI database and marked the corrected genus assignments [13] for reference genomes in the phylogenetic tree (Figure 2B,C). The novel strains formed two distinct clusters. Within each cluster, the genomes exhibited a nearly identical 16S rRNA gene identity and an average nucleotide identity (ANI) (Figure 2B,C), suggesting the homogenetic wild population in lacustrine environments. These four isolates exhibited a 16S rRNA gene identity ranging from 99.1% to 99.5% and an ANI ranging from 82.9% to 89.3% when compared to previously published genomes. The latter metric fell below the established ANI threshold of 95% that delineates a novel bacterial species [27].

The draft genomes of these isolated strains comprised 10–80 contigs, with N50 values spanning from 231 Kb to 770 Kb (Table 1). Strains designated as *Yoonia* sp. 72 and *Yoonia* sp. 76 possessed genome sizes of approximately 3.66 Mb, a GC content of 61.7%, and a coding density of 91.6% (Figure 2A and Table 1). These genomes encoded around 3522 genes, which formed ~1678 KEGG orthologs (KOs) according to the KEGG database. In contrast, strains *Yoonia* sp. 67 and *Yoonia* sp. 67-2 exhibited slightly larger genomes (~3.8 Mb) and a lower GC content (90.6% and 91.3%) and coding density (90.6% and 91.3%). These two genomes contained 3673 and 3717 genes that encoded 1700 and 1714 KOs, respectively. Collectively, these genomic attributes aligned well with the ranges reported for published *Yoonia* genomes, which have genome sizes of 3.1–4.7 Mb, GC content of 53.4–61.8%, and coding densities of 86.5–92.4%.

### 3.2. Habitat Transition from Marine to Lacustrine Environments

**Figure 3:**
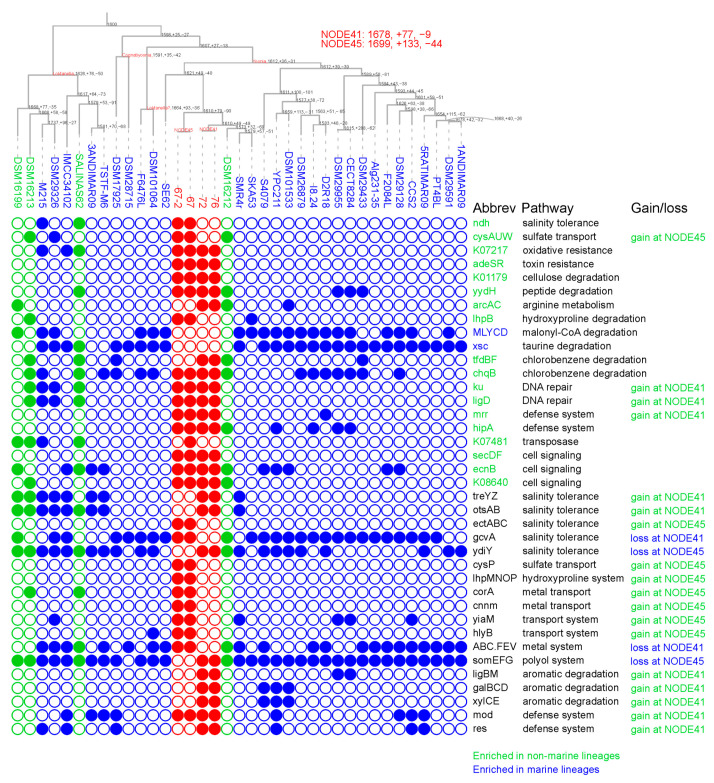
Ancestral metabolic profiling of *Loktanella*, *Cognatiyoonia*, and *Yoonia* genomes. The phylogenomic tree displayed in the upper panel is a replication of that presented in Figure 2. Metabolic comparisons between marine and non-marine genomes were performed using ‘binaryPGLMM’ function in the ‘ape’ R package v5.6. Ancestral genomic reconstructions were conducted using BadiRate v1.35. Each ancestral node is labeled with a triplet of numbers, denoting the aggregate count of KEGG orthologs (KOs) at that node, as well as the numbers of KOs gained and lost. Ancestral nodes of strains sp. 72 and sp. 76 and of strains sp. 67 and sp. 67-2 are labeled as NODE41 and NODE45, respectively. Isolates sampled from Lake Zhangnai on the Tibetan Plateau, lacustrine environments, and marine environments are colored in red, green, and blue in their genome IDs, respectively. The lower panel illustrates the phyletic distribution of selected genes, where filled and open circles signify the presence and absence, respectively, of the corresponding KOs. KOs enriched in marine and non-marine lineages are colored in blue and green, respectively, in the gene abbreviations. Gains and losses of KOs in novel *Yoonia* genomes in the ancestral reconstructions are also marked. The gene abbreviations and the corresponding functions are listed in Table S1.

*Roseobacteraceae* members, including those from the *Loktanella*, *Cognatiyoonia*, and *Yoonia* genera, are known for their ecological preference for marine and intertidal environments [30]. However, several isolates have also been discovered in non-marine habitats, such as inland saline waters and Antarctic lakes, indicating a possible ecological transition from marine to lacustrine habitats [31]. The four novel *Yoonia* isolates presented herein were sampled from a brackish lake on the Tibetan Plateau, thereby expanding the genomic resources available for non-marine lineages. To identify metabolic functions enriched in either marine or non-marine lineages, we employed a ‘binaryPGLMM’ analysis that accounts for the phylogenetic branching order and evolutionary history in the metabolic comparisons [29]. Our analysis revealed two genes related to the degradation of malonyl-CoA and taurine that were enriched in marine lineages (Figure 3). Conversely, eighteen genes or operons were found to be enriched in lacustrine members, many of which were associated with the utilization of various compounds such as cellulose, peptide, arginine, hydroxyproline, and chlorobenzene. Intriguingly, the enrichment of genes related to chlorobenzene degradation, specifically 2,4-dichlorophenol 6-monooxygenase (*tfdB*), maleylacetate reductase(*tfdF*), and hydroxyquinol 1,2-dioxygenase (*chqB*), contrasted with the previously reported loss of 2-haloacid dehalogenase in non-marine Roseobacters [14]. This discrepancy might be attributed to the prevalent presence of organohalogens in cold environments like Antarctic lakes [32]. Collectively, our findings suggest that the differentiated utilization of nutritional substrates is a key adaptive feature in lacustrine lineages within the *Loktanella*, *Cognatiyoonia*, and *Yoonia* genera.

Some of these metabolic enrichments in non-marine lineages were largely attributed to the KO presence in the four novel *Yoonia* isolates. For example, four genes were identified only in novel *Yoonia* isolates but not in previous published reference genomes, including those encoding toxin resistance two-component system (*adeSR*), endoglucanase for cellulose degradation (K01179), restriction system (*mrr*), and fusion protein for cell signaling (*secDF*). Next, we further explored the metabolic adaptations accompanying the ecological transition for these novel isolates. We analyzed the ancestral genomes of the *Loktanella*, *Cognatiyoonia*, and *Yoonia* genomes. The last common ancestor (LCA) of strains *Y.* sp. 72 and *Y.* sp. 76 (NODE41 in Figure 3) was estimated to harbor 1678 KOs and subjected to a net gain of 68 KOs (a gain of 77 KOs and a loss of 9 KOs). A similar trend of net KO gain was also identified at the LCA of strains *Y.* sp. 67 and *Y.* sp. 67-2 (NODE45 in Figure 3), which possessed 1699 KOs and experienced a net gain of 89 KOs (a gain of 133 KOs and a loss of 44 KOs).

One of the most crucial differences between marine and non-marine aquatic ecosystems is the variation in salinity levels. Such ecological transitions often involve alterations in genes linked to osmoregulation and ion transport [9]. For example, sodium antiporters have been documented to be consistently lost in many non-marine *Rhodobacteraceae*, while the biosynthesis pathway for ectoine is significantly enriched in marine *Roseobacteraceae* [14]. In the case of the newly identified *Yoonia* strains, only two salinity-related genes were found to be lost in either NODE41 or NODE45, while no metabolic alterations related to sodium and potassium transport were identified. Intriguingly, the gene clusters *ectABC* and *treYZ-otsAB*, which are responsible for the synthesis of ectoine and trehalose, were estimated to be acquired at NODE45 and NODE41, respectively. Both ectoine and trehalose function as compatible solutes that confer resilience to extreme osmotic stress [33]. Given that these novel *Yoonia* strains were isolated from brackish environments with a salinity of 0.27% and given the prevalence of brackish and saline lakes across the Tibetan Plateau [34], these metabolic acquisitions likely equipped these strains to withstand a wider range of salinity conditions, potentially enabling them to colonize a more diverse array of lake environments on the Plateau.

Transport functions stand as another key metabolic divergence between marine and lacustrine microbes [9]. In the case of LCA NODE45, the gene cluster *cysAUWP*, which is responsible for sulfate transport, was gained, which aligned well with previous research [9] and the significantly lower sulfate concentrations in lake environments [35]. Additionally, NODE45 acquired gene cluster *lhpMNOP*, which is responsible for hydroxyproline transport. Hydroxyproline is a major component of animal collagen, which may be more readily available in terrestrial niches. In contrast, NODE45 and NODE41 each lacked gene cluster *somEFG*, which is associated with polyol transport, and gene cluster ABC.FEV, which is responsible for iron complex transport, the latter of which was consistent with the generally higher bioavailability of iron in lacustrine ecosystems [36]. 

The comparative genomic analysis between marine *Roseobacteraceae* and lacustrine *Rhodobacteraceae* has previously identified other metabolic pathways typically lost in the latter, such as mercury antitoxin, carbon monoxide oxidation, and de novo cobalamin synthesis [14]. However, these metabolic changes were not identified in the ‘binaryPGLMM’ comparison or in the ancestral genomes of the newly identified *Yoonia* strains. Instead, strains *Y.* sp. 72 and *Y.* sp. 76 from Tibetan Plateau lakes exhibited enhanced metabolic capabilities for degrading aromatic compounds, as evidenced by the acquisition of the gene clusters *ligBM*, *galBCD*, and *xylCE*. These compounds are likely introduced into the lake water from the surrounding soil and plant residues through precipitation or glacial melt [37]. Additionally, all four novel strains acquired genes associated with restriction and defense systems, which may facilitate their adaptation to the extreme conditions of the Tibetan Plateau [38]. Taken together, these metabolic shifts diverged from other previous research, suggesting that adaptation to lacustrine environments may be phylogenetically specific and depend on the scope of genome samplings.

## 4. Conclusions

Given the large population sizes and high dispersal abilities of aquatic microbes, the genomic differentiation between marine and non-marine habitats remains one of the most enigmatic questions in ecological fields [9]. In this study, we isolated four novel *Yoonia* strains from a brackish lake on the Tibetan Plateau. Diverging from their marine counterparts within the same genus, these strains have transitioned from their original marine habitats to lacustrine niches. Serving as a supplement to previously reported non-marine *Loktanella* and *Yoonia* lineages, these new strains enrich the existing genomic repertoire for studying the microbial adaptations to lacustrine ecosystems within the genus level. These novel strains share certain metabolic changes commonly observed in other non-marine *Rhodobacteraceae* (e.g., specialized compound transport) and in non-marine *Loktanella* and *Yoonia* lineages (e.g., sulfate transport). Additionally, they display unique metabolic adaptations, such as aromatic compound degradation and defense systems. These specialized adaptations may reflect localized responses to Tibetan Plateau environments. Taken together, our study broadens the known genomic information of non-marine Roseobacter lineages and illuminates their ecological transitions characterized by both shared and unique metabolic adaptations.

## Figures and Tables

**Table 1:** Genomic features of four novel *Yoonia* isolates.

Genome ID	Contigs	N50 (bp)	Genome Size (bp)	GC Content	Coding Density	Genes
*Yoonia* sp. 72	10	770,970	3,664,109	61.7%	91.6%	3524
*Yoonia* sp. 76	16	704,701	3,665,672	61.7%	91.6%	3522
*Yoonia* sp. 67-2	80	299,240	3,855,349	59.2%	90.6%	3717
*Yoonia* sp. 67	41	231,947	3,767,971	59.4%	91.3%	3673

## Data Availability

The assembled contigs of the four novel *Yoonia* strains are available in the NCBI database under the accession number PRJNA1026722 and the NODE database (https://www.biosino.org/node/) accessed on 13 November 2023 under the accession number OED866054-OED866057.

## Supplementary Materials

The following supporting information can be downloaded at: https://www.mdpi.com/article/10.3390/microorganisms11112817/s1, Figure S1. Phylogenomic tree constructed using RAxML v8.2.12. Nodes with bootstrap support exceeding 80% are indicated by solid circles. Table S1. Metabolic alterations leading to the last common ancestor (LCA) of strains *Y.* sp. 72 and *Y.* sp. 76 (NODE41) and the LCA of strains *Y.* sp. 67 and *Y.* sp. 67-2 (NODE45). Text S1: Sequence of the bac120 alignment.
