# Supplementary material for: Genomics of Yoonia sp. Isolates (Family Roseobacteraceae) from Lake Zhangnai on the Tibetan Plateau
Source: Microorganisms. 2023 Nov 20;11(11):2817. doi: 10.3390/microorganisms11112817 (PMC10673129; doi:10.3390/microorganisms11112817)

Figure S1. Phylogenomic tree constructed using RAxML. Nodes with bootstrap support exceeding 80% are indicated by solid circles.

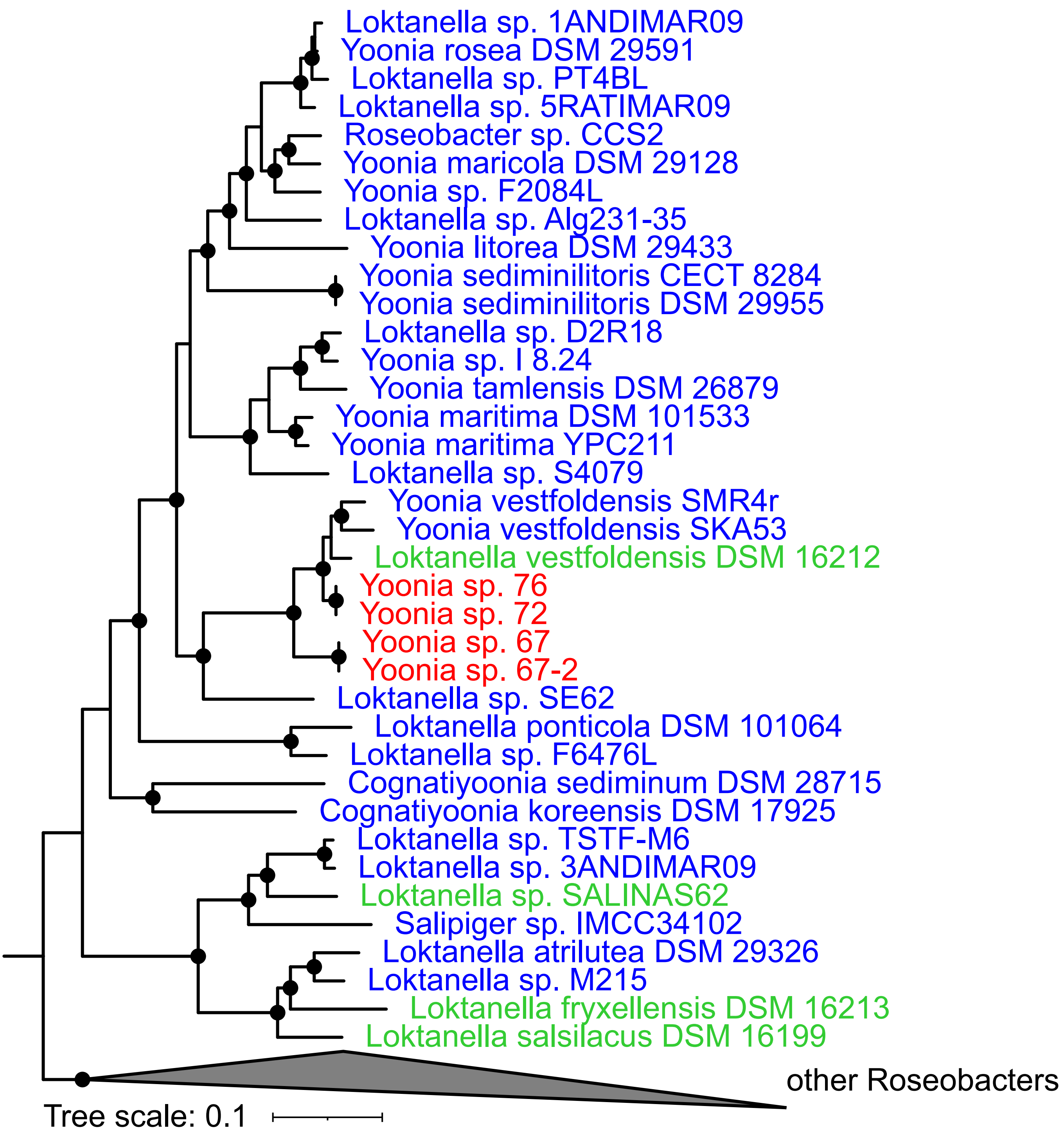

Supplement: Supplementary file 1 [file microorganisms-11-02817-s001.zip › Figure S1.pdf]
