# Supplementary material for: Genomics of Yoonia sp. Isolates (Family Roseobacteraceae) from Lake Zhangnai on the Tibetan Plateau
Source: Microorganisms. 2023 Nov 20;11(11):2817. doi: 10.3390/microorganisms11112817 (PMC10673129; doi:10.3390/microorganisms11112817)
Supplement: Supplementary file 1 [file microorganisms-11-02817-s001.zip › Table S1.pdf]

Supplemental Table S1. Metabolic alterations leading to the last common ancestor (LCA) of strains Y. sp. 72 and Y. sp. 76 (NODE41) and the LCA of strains Y. sp. 67 and Y. sp. 67-2 (NODE45).

KO	Abbreviation	Gene function	node41	node45
K00004	BDH, butB	(R,R)-butanediol dehydrogenase / meso-butanediol dehydrogenase / diacetyl reductase	gain	
K00010	iolG	myo-inositol 2-dehydrogenase / D-chiro-inositol 1-dehydrogenase		loss
K00064	E1.1.1.122	D-threo-aldose 1-dehydrogenase		gain
K00066	algD	GDP-mannose 6-dehydrogenase		gain
K00117	gcd	quinoprotein glucose dehydrogenase		gain
K00141	xylC	benzaldehyde dehydrogenase (NAD)	gain	
K00259	ald	alanine dehydrogenase		loss
K00262	E1.4.1.4, gdhA	glutamate dehydrogenase (NADP+)		gain
K00375	K00375	GntR family transcriptional regulator / MocR family aminotransferase		loss
K00389	yidH	putative membrane protein	gain	
K00446	dmpB, xylE	catechol 2,3-dioxygenase	gain	
K00451	HGD, hmgA	homogentisate 1,2-dioxygenase		loss
K00625	E2.3.1.8, pta	phosphate acetyltransferase		loss
K00697	otsA	trehalose 6-phosphate synthase	gain	
K00705	malQ	4-alpha-glucanotransferase	gain	
K00836	ectB, dat	diaminobutyrate-2-oxoglutarate transaminase		gain
K01087	otsB	trehalose 6-phosphate phosphatase	gain	
K01153	hsdR	type I restriction enzyme, R subunit	loss	
K01154	hsdS	type I restriction enzyme, S subunit	loss	
K01156	res	type III restriction enzyme	gain	
K01166	RNASET2	ribonuclease T2		loss
K01179	E3.2.1.4	endoglucanase	gain	gain
K01185	E3.2.1.17	lysozyme	gain	
K01193	INV, sacA	beta-fructofuranosidase		gain
K01236	treZ, glgZ	maltooligosyltrehalose trehalohydrolase	gain	
K01342	aprE	subtilisin	gain	
K01426	E3.5.1.4, amiE	amidase	loss	
K01438	argE	acetylornithine deacetylase		loss
K01473	hyuA	N-methylhydantoinase A		loss
K01474	hyuB	N-methylhydantoinase B		loss
K01559	oiaT	3-oxoisoapionate-4-phosphate transcarboxylase/hydrolase		gain
K01596	E4.1.1.32, pckA, PCK	phosphoenolpyruvate carboxykinase (GTP)	gain	
K01624	FBA, fbaA	fructose-bisphosphate aldolase, class II		loss
K01729	algL	poly(beta-D-mannuronate) lyase		gain
K01744	aspA	aspartate ammonia-lyase		gain
K01813	rhaA	L-rhamnose isomerase	gain	
K01854	glf	UDP-galactopyranose mutase		gain
K01945	purD	phosphoribosylamine---glycine ligase		loss
K01971	ligD	bifunctional non-homologous end joining protein	gain	
K02013	ABC.FEV.A	iron complex transport system ATP-binding protein	loss	
K02014	TC.FEV.OM	iron complex outermembrane recepter protein		gain
K02015	ABC.FEV.P	iron complex transport system permease protein	loss	
K02016	ABC.FEV.S	iron complex transport system substrate-binding protein	loss	

K02045	cysA	sulfate/thiosulfate transport system ATP-binding protein		gain
K02046	cysU	sulfate/thiosulfate transport system permease		gain
K02047	cysW	sulfate/thiosulfate transport system permease		gain
K02048	cysP	sulfate/thiosulfate transport system substrate-binding protein		gain
K02431	fucU, FUOM	L-fucose mutarotase	gain	
K02453	gspD	general secretion pathway protein D	gain	
K02454	gspE	general secretion pathway protein E	gain	
K02455	gspF	general secretion pathway protein F	gain	
K02456	gspG	general secretion pathway protein G	gain	
K02458	gspI	general secretion pathway protein I	gain	
K02480	K02480	two-component system, NarL family, sensor kinase		loss
K02510	hpaI, hpcH	4-hydroxy-2-oxoheptanedioate aldolase		loss
K02564	nagB, GNPDA	glucosamine-6-phosphate deaminase		gain
K02586	nifD	nitrogenase molybdenum-iron protein alpha chain		gain
K02606	ORC4	origin recognition complex subunit 4		gain
K02623	pcaQ	LysR family transcriptional regulator, pca operon transcriptional activator	gain	
K02624	pcaR	IclR family transcriptional regulator, pca regulon regulatory protein		gain
K02651	flp, pilA	pilus assembly protein Flp/PilA		gain
K02654	pilD, pppA	leader peptidase (prepilin peptidase) / N-methyltransferase	gain	
K02851	wecA, tagO, rfe	UDP-GlcNAc:undecaprenyl-phosphate/decaprenyl-phosphate GlcNAc-1-phosphate transferase		gain
K03149	thiG	thiazole synthase		gain
K03154	thiS	sulfur carrier protein		gain
K03284	corA	magnesium transporter		gain
K03307	TC.SSS	solute:Na+ symporter, SSS family	gain	
K03324	yjbB	phosphate:Na+ symporter	gain	
K03335	iolE	inosose dehydratase		loss
K03336	iolD	3D-(3,5/4)-trihydroxycyclohexane-1,2-dione acylhydrolase (decyclizing)		loss
K03337	iolB	5-deoxy-glucuronate isomerase		loss
K03338	iolC	5-dehydro-2-deoxygluconokinase		loss
K03386	PRDX2_4, ahpC	peroxiredoxin 2/4	gain	
K03427	hsdM	type I restriction enzyme M protein	loss	
K03566	gcvA	LysR family transcriptional regulator, glycine cleavage system transcriptional activator	loss	
K03585	acrA, mexA, adeI, smeD, mtrC, cmeA	membrane fusion protein, multidrug efflux system		gain
K03651	cpdA	3',5'-cyclic-AMP phosphodiesterase		gain
K03707	tenA	thiaminase (transcriptional activator TenA)		gain
K03717	nhaR	LysR family transcriptional regulator, transcriptional activator of nhaA		loss
K03781	katE, CAT, catB, srpA	catalase	gain	
K03818	wcaF	putative colanic acid biosynthesis acetyltransferase WcaF		loss
K03885	ndh	NADH:ubiquinone reductase (H+-translocating)		gain
K04034	bchE	anaerobic magnesium-protoporphyrin IX monomethyl ester cyclase	gain	

K04036	bchJ	divinyl protochlorophyllide a 8-vinyl-reductase	gain	
K04045	hscC	molecular chaperone HscC	gain	
K04063	osmC, ohr	lipoyl-dependent peroxiredoxin		gain
K04085	tusA, sirA	tRNA 2-thiouridine synthesizing protein A		loss
K04095	fic	cell filamentation protein		gain
K04101	ligB	protocatechuate 4,5-dioxygenase, beta chain	gain	
K04343	strB	streptomycin 6-kinase		gain
K04565	SOD1	superoxide dismutase, Cu-Zn family	gain	
K04749	rsbV	anti-sigma B factor antagonist		gain
K04757	rsbW	serine/threonine-protein kinase RsbW		gain
K05343	treS	maltose alpha-D-glucosyltransferase / alpha-	gain	
K05523	hchA	D-lactate dehydratase / protein deglycase		gain
K05785	rfaH	transcriptional antiterminator RfaH		gain
K05786	rarD	chloramphenicol-sensitive protein RarD		loss
K05835	rhtC	threonine efflux protein	gain	
K05979	comB	2-phosphosulfolactate phosphatase		gain
K06044	treY, glgY	(1->4)-alpha-D-glucan 1-alpha-D-glucosylmutase	gain	
K06118	SQD1, sqdB	UDP-sulfoquinovose synthase		gain
K06183	rsuA	16S rRNA pseudouridine516 synthase		gain
K06193	phnA	protein PhnA		gain
K06203	cysZ	CysZ protein		loss
K06223	dam	DNA adenine methylase	gain	
K06602	flaF	flagellar biosynthesis activator protein FlaF	gain	
K06718	ectA	L-2,4-diaminobutyric acid acetyltransferase		gain
K06720	ectC	L-ectoine synthase		gain
K06864	larE	pyridinium-3,5-biscarboxylic acid mononucleotide sulfurtransferase		gain
K06877	K06877	DEAD/DEAH box helicase domain-containing	gain	
K06898	larB	pyridinium-3,5-biscarboxylic acid mononucleotide synthase		gain
K06919	K06919	putative DNA primase/helicase	gain	
K06959	tex	protein Tex		gain
K07046	K07046	L-fuconolactonase		gain
K07062	fitB	toxin FitB		gain
K07089	K07089	uncharacterized protein	gain	
K07217	K07217	Mn-containing catalase	gain	
K07226	hutZ, hugZ	heme oxygenase (biliverdin-IX-beta and delta-forming)		loss
K07238	TC.ZIP, zupT, ZRT3, ZIP2	zinc transporter, ZIP family		gain
K07246	ttuC, dmlA	tartrate dehydrogenase/decarboxylase / D-malate dehydrogenase		gain
K07248	aldA	lactaldehyde dehydrogenase / glycolaldehyde dehydrogenase	gain	
K07273	acm	lysozyme		gain
K07283	ydiY	putative salt-induced outer membrane protein		loss
K07315	rsbU_P	phosphoserine phosphatase RsbU/P		gain
K07316	mod	adenine-specific DNA-methyltransferase	gain	
K07317	K07317	adenine-specific DNA-methyltransferase		gain
K07340	ybbJ	inner membrane protein		loss
K07389	cyaC, hlyC, rtxC	cytolysin-activating lysine-acyltransferase		gain
K07448	mrr	restriction system protein	gain	
K07451	mcrA	5-methylcytosine-specific restriction enzyme A	gain	
K07496	K07496	putative transposase		gain

K07516	fadN	3-hydroxyacyl-CoA dehydrogenase		gain
K07684	narL	two-component system, NarL family, nitrate/nitrite response regulator NarL		loss
K07734	paiB	transcriptional regulator		gain
K08281	pncA	nicotinamidase/pyrazinamidase		loss
K08355	aoxA	arsenite oxidase small subunit		gain
K08356	aoxB	arsenite oxidase large subunit		gain
K08678	UXS1, uxs	UDP-glucuronate decarboxylase	gain	
K08809	SPEG	striated muscle-specific serine/threonine protein		gain
K09001	anmK	anhydro-N-acetylmuramic acid kinase		loss
K09121	larC	pyridinium-3,5-bisthiocarboxylic acid mononucleotide nickel chelatase		gain
K09123	lhpI	cis-L-3-hydroxyproline dehydratase		gain
K09779	K09779	uncharacterized protein	gain	
K09794	K09794	uncharacterized protein		loss
K09803	K09803	uncharacterized protein		gain
K09922	K09922	uncharacterized protein		gain
K09930	K09930	uncharacterized protein		loss
K09955	K09955	uncharacterized protein		gain
K10192	togB	oligogalacturonide transport system substrate-binding protein	gain	
K10193	togM	oligogalacturonide transport system permease	gain	
K10194	togN	oligogalacturonide transport system permease	gain	
K10195	togA	oligogalacturonide transport system ATP-binding protein	gain	
K10218	ligK, galC	4-hydroxy-4-methyl-2-oxoglutarate aldolase	gain	
K10227	smoE, mtlE	polyol transport system substrate-binding protein		loss
K10228	smoF, mtlF	polyol transport system permease protein		loss
K10229	smoG, mtlG	polyol transport system permease protein		loss
K10546	ABC.GGU.S, chvE	putative multiple sugar transport system substrate-binding protein		gain
K10547	ABC.GGU.P, gguB	putative multiple sugar transport system permease protein		gain
K10548	ABC.GGU.A, gguA	putative multiple sugar transport system ATP-binding protein		gain
K10552	frcB	fructose transport system substrate-binding protein		loss
K10553	frcC	fructose transport system permease protein		loss
K10554	frcA	fructose transport system ATP-binding protein		loss
K10806	yciA	acyl-CoA thioesterase YciA		gain
K10820	ytfR	galactofuranose transport system ATP-binding	gain	
K10979	ku	DNA end-binding protein Ku	gain	
K11003	hlyD, cyaD	membrane fusion protein, hemolysin D		gain
K11004	hlyB, cyaB	ATP-binding cassette, subfamily B, bacterial HlyB/CyaB		gain
K11177	yagR	xanthine dehydrogenase YagR molybdenum-binding subunit		gain
K11178	yagS	xanthine dehydrogenase YagS FAD-binding		gain
K11447	UTX, KDM6A	lysine-specific demethylase 6A	gain	
K11475	vanR	GntR family transcriptional regulator, vanillate catabolism transcriptional regulator	gain	
K11690	dctM	C4-dicarboxylate transporter, DctM subunit	loss	
K11860	OTUD7A_B	OTU domain-containing protein 7		gain
K12257	secDF	SecD/SecF fusion protein	gain	gain
K12658	lhpA	4-hydroxyproline epimerase		gain
K12856	PRPF8, PRP8	pre-mRNA-processing factor 8	gain	

K12960	mtaD	5-methylthioadenosine/S-adenosylhomocysteine deaminase		gain
K13015	wbpA	UDP-N-acetyl-D-glucosamine dehydrogenase		gain
K13016	wbpB	UDP-N-acetyl-2-amino-2-deoxyglucuronate dehydrogenase		gain
K13017	wbpE, wlbC	UDP-2-acetamido-2-deoxy-ribo-hexuluronate aminotransferase		gain
K13018	wbpD, wlbB	UDP-2-acetamido-3-amino-2,3-dideoxy-glucuronate N-acetyltransferase		gain
K13019	wbpI, wlbD	UDP-GlcNAc3NAcA epimerase		gain
K13210	FUBP	far upstream element-binding protein		gain
K13483	yagT	xanthine dehydrogenase YagT iron-sulfur-binding subunit		gain
K13609	dpkA, lhpD	delta1-piperideine-2-carboxylate reductase		gain
K13622	btaA	S-adenosylmethionine-diacylglycerol 3-amino-3-carboxypropyl transferase		gain
K13623	btaB	S-adenosylmethionine-diacylgycerolhomoserine-N-methlytransferase		gain
K13641	iclR	IclR family transcriptional regulator, acetate operon repressor		loss
K13688	chvB, cgs,	cyclic beta-1,2-glucan synthetase		gain
K13876	araD	2-keto-3-deoxy-L-arabinonate dehydratase		gain
K13877	aldH	2,5-dioxopentanoate dehydrogenase		gain
K13924	cheBR	two-component system, chemotaxis family, CheB/CheR fusion protein	gain	
K13979	yahK	alcohol dehydrogenase (NADP+)	gain	
K15066	ligM	vanillate/3-O-methylgallate O-demethylase	gain	
K15228	mauA	methylamine dehydrogenase light chain		gain
K15229	mauB	methylamine dehydrogenase heavy chain		gain
K15532	yteR, yesR	unsaturated rhamnogalacturonyl hydrolase	gain	
K15598	thiY	putative hydroxymethylpyrimidine transport system substrate-binding protein		gain
K15599	thiX	putative hydroxymethylpyrimidine transport system permease protein		gain
K15600	thiZ	putative hydroxymethylpyrimidine transport system ATP-binding protein		gain
K15629	CYP152A	fatty-acid peroxygenase	gain	
K16147	glgE	starch synthase (maltosyl-transferring)	gain	
K16264	czcD, zitB	cobalt-zinc-cadmium efflux system protein		gain
K16302	CNNM	metal transporter CNNM		gain
K16514	galD	4-oxalomesaconate tautomerase	gain	
K16515	galB	4-oxalomesaconate hydratase	gain	
K16516	galR	LysR family transcriptional regulator, regulator for genes of the gallate degradation pathway	gain	
K16692	etk-wzc	tyrosine-protein kinase Etk/Wzc		gain
K16841	hpxA	allantoin racemase		loss
K17218	sqr	sulfide:quinone oxidoreductase		loss
K17226	soxY	sulfur-oxidizing protein SoxY		loss
K17229	fccB	sulfide dehydrogenase [flavocytochrome c] flavoprotein chain		loss
K17401	MRPS22	small subunit ribosomal protein S22		gain
K17588	PREX2	phosphatidylinositol 3,4,5-trisphosphate-dependent Rac exchanger 2 protein		gain
K17836	penP	beta-lactamase class A		gain

K17850	ampR	LysR family transcriptional regulator, regulator of gene expression of beta-lactamase		gain
K18009	budC	meso-butanediol dehydrogenase / (S,S)-butanediol dehydrogenase / diacetyl reductase		gain
K18138	acrB, mexB, adeJ, smeE, mtrD, cmeB	multidrug efflux pump		gain
K18143	adeS	two-component system, OmpR family, sensor histidine kinase AdeS	gain	gain
K18144	adeR	two-component system, OmpR family, response regulator AdeR	gain	gain
K18335	K18335	2-keto-3-deoxy-L-fuconate dehydrogenase	gain	
K18802	DUG3	glutamine amidotransferase		gain
K18910	dpe, lre	D-psicose/D-tagatose/L-ribulose 3-epimerase		gain
K19163	ccdB	toxin CcdB		gain
K19165	phd	antitoxin Phd		gain
K19271	catA	chloramphenicol O-acetyltransferase type A		loss
K19290	alg8	mannuronan synthase		gain
K19293	algX	alginate biosynthesis protein AlgX		gain
K19294	algI	alginate O-acetyltransferase complex protein AlgI		gain
K19295	algJ	alginate O-acetyltransferase complex protein AlgJ		gain
K20074	prpC, phpP	PPM family protein phosphatase		loss
K21001	pslH	polysaccharide biosynthesis protein PslH	gain	
K21060	lhpB	D-hydroxyproline dehydrogenase		gain
K21062	lhpC	1-pyrroline-4-hydroxy-2-carboxylate deaminase		gain
K21147	moeZR, moeBR	sulfur-carrier protein adenylyltransferase/sulfurtransferase		gain
K21310	mddA	methanethiol S-methyltransferase		loss
K21394	yiaM	TRAP-type transport system small permease		gain
K21802	vdh	vanillin dehydrogenase	gain	
K22441	paiA	diamine N-acetyltransferase		gain
K22479	argA	N-acetyltransferase		gain
K22548	lhpL	trans-L-3-hydroxyproline dehydratase		gain
K22602	hpxW	oxamate amidohydrolase		gain
K23054	desC	sn-1 stearoyl-lipid 9-desaturase	gain	
K23061	lhpP	hydroxyproline transporter system substrate-binding protein		gain
K23062	lhpM	hydroxyproline transport system permease protein		gain
K23063	lhpN	hydroxyproline transport system permease protein		gain
K23064	lhpO	hydroxyproline transport system ATP-binding		gain
K23186	fepD, fagA, cchC, desH	iron-siderophore transport system permease protein		gain
K23187	fepG, fagB, cchD, desG	iron-siderophore transport system permease protein		gain
K23188	fepC, fagC, cchE, desF	iron-siderophore transport system ATP-binding protein		gain
K23219	MBD5	methyl-CpG-binding domain protein 5		gain
K23238	rpiR, alsR	RpiR family transcriptional regulator, repressor of rpiB and als operon		gain
K23359	biuH	biuret amidohydrolase	gain	
K23508	ytfQ	galactofuranose transport system substrate-binding protein	gain	
K23509	ytfT, yjfF	galactofuranose transport system permease protein	gain	
K23533	LINGO, LRRN6	leucine-rich repeat and immunoglobulin-like domain-containing nogo receptor-interacting	gain	

K25286	fagD, cchF, irp1A, piaA	iron-siderophore transport system substrate-binding protein		gain
K25307	wzb, etp	low molecular weight protein-tyrosine phosphatase		gain
K26858				gain
