# Supplementary material for: Genomics of Yoonia sp. Isolates (Family Roseobacteraceae) from Lake Zhangnai on the Tibetan Plateau
Source: Microorganisms. 2023 Nov 20;11(11):2817. doi: 10.3390/microorganisms11112817 (PMC10673129; doi:10.3390/microorganisms11112817)
Supplement: Supplementary file 1 [file microorganisms-11-02817-s001.zip › Text S1.pdf]

>GCA_000011965
AVIKPVVQDAYAPVLLIRQQQVATVTGAGSPSLRAKTVYRKSGLQRKTSTKWVAKPLYY-
GVLKMGKIVQLVSISVDNRSVCTVAQVVEQEVVVHGLVALEAVKRKIALEAMDGMTTEDPAKVAEFE
EELDLKKFRDAEARADMLPVMLGLTLGLRFDPHEPVVHDLPVPVIYEVI-
GIMADKGGIEVDDCAISNAVSDVDPIAYVSPIDLAAVAGEQMAFIDQEAAVAAAREAIALEELALEAGI
LVVGIDPLLLPV-FGADADV-MSFANRSRELASIVALAVAAEELSRA-IDS--ASDM-
MLRAQAAALAEKRGTAEQIRIVKRMQRSAGLADDPELVLLEDAAEGNSSWAGQIYDDPISGSSESDS
AGNIVHAANARQNFARSITFLRKVEISDKAAAPLMRGVKHNSKSRSSRKLDDAITALNFPNVPPGIAIR
AAAGKPADTLLHDATEGERILLIVVRWHVTWKTAVIVALLSAAGDIVGLVALGGIAAVGKPDGLLNRH
HKLNGSLIEQRPIVEKLTLRLRAALKEKLAKDQIIIFVVKVQGDLVADAKVQMG-
GPVGAVVAIQGELIFVRRHRQTVITDQEATV-ILKGVAEGPADAQALLRIRVTHTIVELSVLLIPE-
PVLVDSVTIREGVGKALQIIQLDVYLLRDMGTGAETLAKSASREVIREVLAVDPITGVRLAYGRVKERM
GKKLGLKDLE-----
ISILPDVTEPRMENLPGGICRNFIVAGPRAAVGMLFLLLDDAGKLYTIDGDMRELMKSVKSLIGLVDSKS
PKVLSASYAEDRLRIRFADCEVLGSDANLYPYKEERRELLRMVPVYLIIEAKRRQLIIFALSLKGGHRRTM
MALGVMIAAAILESQAEMGPVHHARPQILLDDAELVGTSMMISRQAALQLKDLNKIDVIVIFPEFQEK
MVVSLRFFCTEVAIFVIALIVTVGEMLSREPEHAQEEGAGYQKRIRGHSTLFALEVTFDRTNTGRDATP
GLFLGMGLADPRYGILYVNQPKKKQMPSEPDDDRVDGFELVHAIKEDSAS-
HELEMPEAMALAVEADASINVTAGVLAMKGGMVAVEKVDSKRKVALAAEKWNISKLELIDVLLSGAK
PFRSCG-
IELADLSATLGKKKLWFLFLPSASNAELEEVKLDGAGASTAEAAEDKQIAILLVRPVLIVIQDSNTAGLIS
NGGLDEWGTMAYKKHYAFAARMRLHMRTIDAIEVGVAMEPNKQKSEKVKTVKLEGDTRFRQLAVE
GIKEQMP-LHALNPDFAV-LPILVTPAVGSVAADEGVLGATVPVDASL-
RELQKSQKGTALPISAVRLLAMRAARGMIEEDIMVAGKIAA-
LLKIRALETIVQIVLRNDSEATVAWRLSVAEKLEADTKRSAGFAARPFKSKPSLCDVQAHARIAEPIQEIA
AGYVAKEERFVYPVEDIAIKLILASPHSRVGKAKRAGEGHLHGMAKELLAKAVLMLCNEPNTNTEGLL
NYLTTNQPTDLLFEDCLDVIIDIRRMSVV--
VHLNTV-
HAVVKGVGEVGDYEIMAAAAASGLVQEERVDDRTGRERTLMWMYGAMEELWLMSLRWADWVV
DCAIGAIILHVYAQSINRAFSLRRLQLDGWPKEAARWWNTWQLWLNKDGFFGYVAAFRAVKQVLK
AALADFDMSDSQGVGRYLSKQEIRAGYRIRQNVQFARLDPRADILLQAQTRLSVIRHALAVVAILILRD
IASEFT-
VVAYQDVARGHVECVLAECFDFTEKNAHIGPVGLRTPESIFGDGTVLNEAVLGKIDLANTVADFRYLV
LLDGGRALGVEWITLQNMLYARIWLPPAQAALKNLLPICFFLSHPELDLVNV-
ELFLGISKTRAEQLMAGALIEEILEAVARFAEPEATTHQSKKVKHFKYYGEEVDPVHLAYILDLFALVSTQ
VTVSDRLLKDRYVLNLKVFRGFTSLWWLALQLAEKGPAATVITVGTIAGLGALVLFIILL-
RDKVLVETLYYRYVY--MRGYSAAKDVADADPEHFLELSAASRDLEWIEIVPAFFYYFDTAT-
FEPFNTAVAKEFFGLFAERLRVVDVLAVPPDRVLDLVEFVALAGTLATSAEA-
PGIIATGLCEPNAAAVLEAERDWNLHRSMGITLAFFEARESVKGYFDKGLHKGVAEVSLREAGAFTTN
FHCAVEAKRCAPAVHRVLDLGEIEAALIEIDSAVTVADEEQTLADKPTTRDKAVGAIGGVVLAGTMN
MGANLEQIERSGNIIIELEEEQYHAVNGKKADGKLTDKVLMVLSVAYFPVITRTSSGNALDHGVFDLLI
DAPADADNLDHGRVMSIPAPTIHAETTPLYADAEIDRIAWIAPLHLLGTLPAVLGFEPDQYSFISMMID
ASLDITETAEVLDDYHVLEKGSRPPAFRIVLIRGLHIRLLAAVGTILVVGSAGRAEITAYIQGRAVPISMRK
QTFLPAFAASSCKVVDIFSTMLRPPERSSVQMLILETVAEL-

LEVTRLTNDKLERMEVLLPGLDMAELLGLLAAAADFADIVTIDAAVTAILSVAAEAVVLREMLLTKAEIAI
TALALALALLLMLTGDKGLTQKDRAREAQMKELIQAGAVLQIHAADAMVLAATADKVRLETDEGTTS
AFASLNLATVSDGFYVLSSAVLQLSSDLMMIVSVARVLADKETKNALVVRHPEQVKTQPTYAEGSPG
MVLQTLTECIPPLAIKPEEVELLWTLVAALWRVKAHLASAARPRLERVLILLLALGLIIVLMQSEGGGMG
GG-
MGAAAKVLAAFSILTIKASVGDEVRKVTRRVMDLNTRLGRPEEAVNPEFKGEELDQAKYEAENVEVDC
VETVRRFVDIPFLVSIRTQHNESEDRGARLVLDVTRVRFIDL-
VTKKSDVILVERVETDGIIARQMAKDLQSLHEEAAGLGKVSALEHFIALNDGHISNYVTAGTHSVLGML
LLVLFIQQVRVSKVVWPTRREVTMVFIMAALAIFFLVIGLLLNLVFMVGGISPLQITTLVLGEGIMAVRQ
VSTFPAES-
ITILQNTYADLICAGRVTKADIENSLHIVVAARVSRGTWYVIYLYEISQAQSPAGILQRAFRQATLSQSLI
DGRMEVKIKVVTSDRYA-
TYEIKGGQHRKGTRYNRVKVGHNIPVEVLARLKMAFHPANLVLVKTFGAVVQKRFAQQLTHIRHGAV
AIGPMEVSSPYIKSKKARHAPGDSVVDIKPDNIVRSVAVVPELIWARENEDQSAVHEKYVKTTHKLAA
KRSTAVDKAQYDKRNQVHLSFIGLAVKVLPEQAIVAATAGTLLPTSSVSVTENLKGVAATIRIDPMEKA
LIAYIKRMRPQLNLAGQTDNKIVKCLNNNLDDVEWGKLGRRKIKPLTIKSVYDVEHRRATELVKLMPY
KDDVEGRKSQFAEK--
AIFEPLKAFMMLFGFIARAIYALSLRVVRRTILLVKTKIVVNADTALSAIKIIGVSIRLRHKSTNVADTKHHI
KTIEARARMTLAGTPAVKLHLSPGRIGKKGGRIKGYMPYKNFNIFEGLASGRRAKVKELAKLKKVDKTIT
VDGIRTRQQGGRLPKADLSNAL-
NTRVGVDIVGENEQEASNPRANAEMGQIFAAIDARVYSLVAALIGHALEAVLALCAILGIGRAHALLA
VKLKINAGIEMHSAA-VSEAVAAA-HAQPGGEC-
VLLDVSEGDLGVLLMVEASLDVCRLRRRQLGELKKTKKEAYMSLTCFSGKGDKVKIVEATQTQAFEIVA
TRTAERFGIHAKGTYGKERAMMQITPTKPVKVWVPVFVNDEELLAPIRAFAETNGDGVTVVKLTKDVK
ITMFEKAIASLAVAGKATSVVYTVLEKVTELSKDIIYEQRAKAHYGRGRLRSHVVTTTKQKRHLSMMKR
TVIASPSVNAARLHDGEVSVVTGATMAFVMSEQDAV--
GDVRMHFQQEPRREAQLGIAGPETFLATEQPRVLALLFLREYLIHIIEIGTVGGFADAATIYDILRAMQS
KLDRKLSADIAKVRMCLHDDVADMGVTEVAARRDTAGDAILSAYWINGVPANTDIILLVANLECATA
PENIDCAVRLFYQRVTLQAKERGGDVSVALVPAKNQIEAAEGTVIYTIKGNKPSTVQMKLNIDGISRSI
VKSLAVQLFQVELFLNDTWIATLLRAAQDVVFVDAREGREAVDHVIERDTELEFAPQKDIVARKIRIYTP
AAPSAVVIWDPAIERADKADAQGTILMIYGPDLQDISCFTDIGAEPKIESLKSWDEKEKYVVHRDDIPA
DITTLVGKSSKVNKEFGIPDVAVCAPAKPGVTESIDRQEPKAGSCGEVANLPYPQESLVNRVSSANVQ
KNISMVLGGHPIGEMGIARGVERHLETNMCEIFVDKDDIDVALDFYKLELTSLRVGLDKPNAKVEADG
KESRKLMAVRDGAAALQRHPKVSEESGTR-KAGEKAKLPDDTLIFDGVQHGDIKFTATDLPEGRV-
VTPVVMTVKAQLKIDKVMIKTFHVVTLSFSNDKGEEHLLKLRLNALKKSTRHYEMLLKIRDTECAQSPH
AIPVGKILKIDALLQTICKAHYTAIEPLMAEDLVALGVMGDQGLLHVVPRLMIL---
HLCELEAALQLPLWGLQQRAVVTDQTMGLAIRARDWSY-
AISEVVGNTFFARSKEDDTEQRLHARNPI-IIL-YGKFGMV-
ISPSGLRLSSTAPSDQEHGNDEKLILSKERLQDSMYYYILRVLLVFVDEIILAYGSGDQPLKFLVANQAVT
GRDNPQLVCLTRRTFPLDLQRDPVFVLVLTDSTMKNLSHRKMHMNLVLVKPKSWVENVSTKILISDQ
AIASYAEDAGAHTQPSESALAVITSRVIAPIVLVVSQKPAAPSKTLFLEALNLYDAVSTTVQKSPKRYFIF
DGDVISVLGSLDAAVILGMQRPQPDRLRRAVTATAIRLAIALRASGIVAALEAGLINFSRAVDFAALVY
PRA-------------AFRVRNHKVSTKRSRPYKGHTKFLHKAEEEKALVAEGVDTLA
>GCA_000013565

AVIKPITMPVYAPVLMMRQTEVATVSGAGSPSLRAKTVFAKSSLQRKTETKRVAADFYY-
GVLKMSGIVQLVSISVDNRSVCTIAQVVEQEVVVHGLVALDAVKKKIALDAIDGMTADDPAKAADF-
-EIELRRMREADDRSDLLPVMLGLTLGVRFNAHEPVVHDLPVPSIFEVI-
GIMAERGGIDVDECAISNAISDVDPLAYVSPIDLEAVAGDKMEYIDLEAAVSAVKEGVELEELALESTVL
VVGI-PLILPV-FGEDGDT-MSFENRARNLGSIVDLAVSSYELPRARIGPSIAAAM-
MRAAQAAALADKRGTAEEIRIVKRMQKAAGLADNPDLVMVEDAAEGNSSWAGQIYDDPISGSSES
DSVGNIVFAANKRQNFARSITHLRTVEISDQAAAPMMRGVKHNAKSRSSRKIDDAIATLNFPNVPEG
LALRADGGKPPDSLCHDADERERILLLIVRWHVTWRTAIIIVLVLIAGDIVGLIELGGIAAVGKPDGLLNR
HHKMNGSLIEQRPIIEKMTLRLRASLKQKLGAEQIIIFVVKVQGDLVAEAAVQM-
GGVVGAVVAIQGALIYVRRHRQTIITDQESIVTILRGVAEGPADAEALLRIRVDHTLVEMSVLLIPEVPVL
CESVQIREGVGKALQIIQLDVYLLRNMGTGDETLAESMSRELIRTVLAVDPITAVRLAYGSIKERLGKDL
KLKDLQ-----RHLL-
DVTEPRMRDLDAGVCRNMIAAGPGAAVGMLFLLLDDAGKLYSIDGDLRELLKSVKSMIGLADAKAPK
VLSAQYSTERLRIRFADVEVLQSEAELYAYKEERRELLRMVPVYIIAEAKRRQLVIFTLSLKGGHRKAAAAI
GILIAALLIESQADRGTVDHAEPVILLDDAEVVGSSIMVSRQAALDLKTLYKIDVIVIFPEFQEKMVVSLR
FFCTDVAIFVIALITVVGEMLSREPNHAQDEGAGYNKRIRAHSLLFALEVTFDARNCAD--
VPDLFLGLGILPPRYGIIYTNRPKKKQMPAEPDADKVDGFELVHAIKEDGSS-EALEMPEAMALAVE-
DASINVSAGVLATKGGMVAMERVDSKRKLSLAAERWQIAPIDVMDLRLSGACPVRCMD-
IGQADISASLGTKALN-
LFLPKANNAELEEEKLAGAGASTAEAAADRQIAILIVIPVLVVIQDSNAAALNANGGVDEWGTMAYKK
HYSYAARMRLHMRTID------DLAPNKQKAEKIKTAKLEGDTRFRQLVIGGIREQMP-
LHALNPDFAVLLSVLVSPAVGRVAADAAMVGGTVPCPFSL-
KELQKSQKGSALPISGLRLLPMTAARAMIEEDIFVAGKVAA-
LLKIRALETVIKIILRNDSEAALAWRLSVAERIAADTKRSSGFAARPFKTKPSLCNTQAHAEIAEPIETIAAG
YVATEERFVFPVEGLAIQLILASLHSRVGTAKRAGEGHLSEMAKELHAKAVLFVRNEPETSTEGLAKHTT
TGQPTDLLFEECLDVVIDMRRMTIV--IHLNTV-
HVVVKGVRDVGDYDIMAAAAPSGLVQVERVDDRTGMERTKMWMYGAMEELWLLSIKYADWVVN
TPIGAIILHVYAQTINRAFSLKRLQLSGWPPEASRWWNTWQLWLNKDGFFGYDAAFRA-
ARVLKDELSDFDMKDTEGVARYLSRQEIRAGYRIRENVAFNRLDPRQDILLQAMTRLSVIRHALQVVSI
LILREVVSEFS-
VVAQEDVAMGHVECVLADSFDFTAKNADIGPPGLRTPAPIFGDGTVFKDAMLGKMDLSKTVADFRYI
MLIDGGRQLGVWWVGLRNLLYARIWLPPAEARLKDLIPICFFLSHPELDL---------
ISKARADRLMATALIEQILDDVAAYAKPEATTHQSKKEPHFKYYGEEVDPVQLTYIISLFALVATQVTVS
DRLLTDRYVLNLKI-RAETALLWWCLNLAEKGPVATVITVGTIAGLGALVLFIILL-
RDTVLVETLVYRYVY--MRGYSAAKDVADANPEHFLPLANVSRDVEFIEIIPAFFYYFTTAK-
FQPFNTAVAREFFGLFAERLRIIDVLVVPPDRVETLVELVALAGTLATSADAPPGIIATGLCEPNAAAVLE
ADQDFNLHRHMGITLAFFKAQESVKGFFDKGLHKTIAAIGLKEAGALTTTFACGKETQRCVPVVHRVV
DLGELEAGLIEIKSRVVVADEEQTLADQPTTRDNRVGTVGGVVLAGTMNMGASLDAVERGNNIIIELE
EETYNAVNG--
KADKLTDKVLMVLSLAYFPVIARVSSANALDKGVIDLLIPAPEDADNLDHGRTMVIPAVTVHVETAPLY
AEDDIDKIAWVAPLHMLGTIPAVL-
FEPDHMSFISMMIEASLDIVETAEVLDDYHVLEKGSRPPAFRKILIRGLTIRAMAAIGTIIVVGSAGRDEIT
AYIEPGAVPISLRKQTFLPAFAASSCHAVDIFSTMLRPPERSAVQMLILETVAEL-
LEVTRLENEKLERMLMLLPGLDMAAALGLRAAAKDFSAIVTVDEAIVAILSVAAEAVILRELLLTLTEIALT

ALALAVALLLMLTPDEGLSQKEQPREDQMKELIQSGAILQIHADDAMVLASTADRVRLETDEGTTSAF
ASLNLATVSDGFYVMSSAVLQMSPELMMIVEVARVLADKENKNALVVRDPEQVKTQATYAEGSPG
MVLQTLTSVVPPLAVKPEEVELLWTLVTALWRVKAHFATAARPRVERILILILAICLIVVLLQSEGGGMG
GGGVTQAAKFFAAFSILTIKAAVGDEVRKVVRRVMDVSSRLGRPEESVNPEFRGAELEQSKYEAENVE
VDCLETVRRFVDIPFLVSTRTQHNESEDRGARLVLDVTRVRFIDVIVTKKSEIILAARAETDGIIARQMSK
DLQALHPEAAALGKVSALEHFITVQDAHVSNYVTADIHGVLGMLLLVLFLQQTRVAKVVWPTRKEVT
MVFLLALVAIFFGVIALLLQLVFFLSGISVASLTTLVLGEGVMAVRQTGTFPAEA-
IGVLQNTYADLIWAGRVSKADIEESLHIIVAARVARGTWYVIYHYEISQAQGEDGILQRAFRQATLSNS
LIDSRMEVKIKVVTSDRYA-
TYEIKGGQHRKGTRYFRVKVGHNIPVDVLARVKMAFGDPNLVLVKTFGAVVQRRFAQPLTHVHHGA
VTIGPMEVSSPYIRQKKARHAPGDAVVDLKPENIVRSVAVVPELIWARENEDNMAVHDRYTKTTHKIA
AKRTTAVDKAQYDKRQQVHLSFIGLAVKVLPDQAIVHATSGTLLPTEAVSVTEGLKGVAATIRLDPME
KALIAYIKRMKPQLNLGGKTDKKIVKCLNNGLDDVEWGKLGRRKIKPLTIKSVYDVEPERATELVRLMP
YKDDVESRKSKFAEK--
AIFEPLQAFMMLFGFIARAVYALSLRVVRRTIVLVKTRIVVNAATILSAIKIIGVSIRLRHKSTNVADTNHYI
KTIEKRARMTLAGAPDVKLHLSPGRVGTKGGRIKAYMPYKNFNIFDGLASGRRAKVKDLAKLKKVDKEI
SVDGIRTKQQGGRIPQPQLSNALANTRVDVDIVGENVQEESNPRANAEMGQIFADIDARVYHLVAA
MIGHALEAVLALCAIYGIGRAHALLALRLKVNAGIEFHSAA-VPTAVAAA----------------------
----
LKKTKKEAYMKLTCFSGKGERIKIVEASQTQAYEVIATRTAERQGIRAKGTYGKERAMMQITPTKPVKV
WVPVFVSEPELLAPVKAVAETKGDGVTVVKLTTDIKVTMFEKAVASLAVSGQNTSLVYTVLEKVTELAK
DIIYEQRAKAHYGRGRLRSHVVTTTKQKRHLSMMRRSVVADPQMDAARLTDGEVSVITGATIAFVMS
EQDAV--
GDVDMHFQEEPRAEAATNIGGRETFLATEQPRLLVLLFLRAYLIHIVEIETVGGFNDAATIYDILRAMQA
KLDRRLSADIAKVRMVLHDHVTDMGVRQEAPRRESADDAILSAFWINGVTANADVILLVANLECKTA
PENIDCAIRLFYQRITLQAKERGGDVSVALIPAQNQIEAAEGTVIYTIKGETPGTSQMVLNVEAISKSIVK
SLAVQLFQVTLFTEDTWIDTLLRAAETVVFVDAREGKEAVDHVVERDTDTLKGPQKDIVSRKIRIYTPR
APSAVVIWDPAIDREDKADAQGTILKIYGPNLQDINCFTDIGAEPKIESIKSWDEKEKYVVHRDDIPEDI
STMVGKSSKVNKEFGIPDVAVVAPAKPGVTESIDRGEPKAGSCGEVANLPYPQESIVNRVSSANVQK
NISMVLGGHPIGELGISRSVERHLETSMCEIFVDKADVDVALDFYKLELTPLRVGVDTPNAKVQIDGRE
ARRLMAVLDGAAELQRHPKVSSGGGTRVGAGAKARLPDDALVLDGVAHGDLPFTVTDIMDGRV-
VTPVINEVKAQLKIDKVILKTFHVVTLSFSSGKGEEHLIKLRLNALKKSTRHYEMLLKIRETECGESPHAIP
VGVVLKIDRLLQTICVAHYTALEPLLAEDLTVLGVAGEQGLLHLVPRLMILAVMTLWGLAAEMEHPKW
GLQARRIVTDQTMGLAERDRHYSF-AISEVVGNTFFARTKADETEQRIHARNPI-ILL-YGKKAMV-
ISPSGLRLASTQPPALQHGNQEKLILSPERLQDEMAYYVLRILLVFVDLVVLAYGSGEAPIRFLVAEQAV
TGKANPGLVCLTRRTFPLDLQKDATFVLVFEDTTMKNLSHRKMHMNLVLIKSKGWVVNVSTKILISDQ
AIASFAEDAGAHTQPSESALALISSRVMAPIVLVVSQKPTAPSKTLFLEALSLYDEVTCTILNAPKRYYIFE
GDLISVMGAVDAAVLKGLQRPQPDRLRRAVVAAALRLAIALAASDVRAALQAGTINFSRAVSLSA-
CYLDHNLIIDSAIP-PAIAFRVRNHKVSTKRARPYKGHTKFLHKEEMEKSLIAEGVDTIA
>GCA_000014045
AVIKPVTQNEYAPVLMIAQSQVATVSGAGSPSLRAKTVYAKSALQRKTITKWVAKPIYY-
GVLKMGNIVQLVSISVDARSVCTVAQLVEQEVVVHGLVALDAVKRKIALEAIDGMTTEDPAKVAQYE
EEVELKRFRDAEDRSDMLPVMLGLTLKVRFDPHQPLVHDLPIPVIFEVV-
GIMADRGGIEVDDCAISQAVSDVDPIEYVSPIDLAAVAGTEMAFIDQETAVAAARETIALDELALEASIL

VVGIKPLILPV-FGEDATV-MSFANRSRTLGSIVDLAVAFPRLSRA-IAA--ASEM-
MRAAQAKALAEKRGTAEEIRIVKRMSRSAGLADDPELVMMEDAAEGNSSWAGQIYDDPISGSSESD
SAGNIVFAANKRQNFQRSITFLRRVEISDKAAAPLMRGVKHNAKSRAARKYDDAIALLNFANVPTGV
AIRADLGKPAETLLHDAAQGEKILMIIVRWHVTWKTAVIVVLITDAGNIVGLIELGGIAAVGKPDGTLN
RHHKLNGSLIEQRPIIEKMTLRLRASLKEKLGKDQVIIFVVKVQGDLVADATVQM-
GGTVGAVVAVQGEVIFVRRHKQTVVTDQEATV-ILRGVAEGPADAQALLRIRVTHTLVKISVLLIPE---
LVDAVTIREGVGKALQIIQLDVYLLRDMGTGDETLAESLSRETIRDVLAVDTVTGVRLAYGQIKERLAKK
LTLKDLETML--LSIL-
DITEPRMENLPDGVCRTFVTAGPRAAVGMIFLLLDDAGKIYSIDGDLREVTKSVKSLIGLVDAKAPKVLS
ASYATDRLKVRFADCEVLEAEADLYAYKEERRQLFRMVPVYIVLEAKRRQLVVFALSLKGGHRRAQAA
LGVLIAATLLESQAIEGAVNHASPAILLDDAEIVGTEIMISRQSSLQIKDRVKIDVIVVFPEFQEKMVVSLR
FFCTEVAIFVIALIVTVGEMLSREPEHAQEDGAGFSKRIRAHSVLFALAVSFDRRNLRCDRQ-
DLFLGLGLAPPRYGILYKNQPKKKQMPSEPDADRVDGFELVNAVKEDGAD-QELDMPEAMALAVE-
DATINVSAGVLATNGGMVAVEKVDSKRKMSLAATKWNISKIEVWDVTLSGAAKVRALS-
IELADVSAALSSKKVWFLFLPGASNEELEEAKLDGAGASTAEAAEDRQSPILLVVPVLVVIQDSNTAGLI
SNGGLDEWGTMAYKKHYAFAARMRLHMRTIDGIEVGVEMMPNKQKSEKIKTVRLEGDTRFRQLATE
GVKEQMP-LHALNPDFAV-LPLLVTPAVGSVAADAGVLGARVPVDASL-
RELQKSQKGSALPISAIRILDMRAARAMIEEDIMVA------
LLQITALETVVQLVLRNDSEAILPWRLSVAERLEADTKRAAGYAARPFSSKPSLCDTQAHAIVADPIQEI
AAGYVATPDRFVFPVEGIAIQIVLSNPHSRVGGAKRAGEGHLHEMAKELMAKAVIMLQNEPDTNTEG
LMNYITTNQPTDLLFEDCLDVIIDMRRMSVV--
IHLNTV-
HVVVEGVRDVGNYAIMAAQAPSGLIQMERVDDRTGRERTLMWMYGAMEELWLLSVRYADWVTD
TPIGAIILHVYAQVINRAFSLKRLQLDGWPKEASRWWNTWQLWLNKDGFFGYEAGFRA-
KQVLKAALADYEMKDVHGVARLLSRQELRAGYRIRQQVPFARLSPRTDILLQAQTRLSVIRHALTVVAI
LVLRDVATEFT-
VVAMEDVAMGHVECVLADSFAFTAKDADIGPAGLRTPDTIYGDGTVLKDAVLGKVGLANTVADFRY
RMLIDGGRALGVSWADL-
NMLYPRIWVPPAQAALKNLTPITFFLSHPELDLWRVFSLWSGISKARADQLMASALVEEVLEGVARFA
DPEATTHQSHKERHFKYYGEEVDPVHLAYILKMFALVATQVTVSDRLLKDRYVLNLTV-
RGFTSLLWMSIQLSAKSPSATVITVGTIAGLGALVLFIILLMRDSVLLETLYFRYVY--
MRGYSAAKDVADADPEHFGPLAAASRDMEWIEIVPAFFYYFATAG-
FEPFNTAVAKEFFGLFEERLRLVDVLAVPPDRVEALVEFVALAGTLPTSADTLPGVIATDLCEPNAAAVL
EAERDWNLHRTMGITLAFFKAQISVKGWFDKGLHKDVAETGLSEAGGFTTPFHAGKSAKRCVPVVHR
VIDLGELEAGLIEIDSAVLVADEEQTLADQPTTRDNSVGAIGGVILAGTMNMGSSLDQVERGGNIIIELE
EEQYHAVNGKKADGKITDKILMVLSRAYFPVIERVASGTALDRGVFGLLIQAPDDADNLDHGQPLVIP
ASTVHAETTPLYAESEIDKIAWVAPLHLLGTLAAVLGFEPDHYSFISMMIEASLDITETAEVLDDYHVLEK
GARPPSFRKILIKGLHIRLLAAVGTILVVGSAGRSEITAYINGQAVPITMRKATFLPAFAASSCKTVDIFST
MLRPPERSTIEMLILETVAEL-
LEVTRLENDKLERILILLPGLDFAVIMDLAAVAKDFSQIVTISAAITAILSVAAEKVVLREMLLTKAEIAVTA
LALAMALLLMLTADAGLTQKETARDRQLTELIQGGVVLQVRVEDGMVLAATADKVRLSTDEGTTSAF
ASLNLATVSDGFYVLSSAVLQLSSDLMMIVAVSRVLADKESKNALVVRHPEQVKTQPTYAEGSPGMV
LQTLTACIPPLAIKPEEVELLWTLVAALWRVKSQIASAMRPRLERVLILILALGLIVVLLQSEGGGMGGG-
MGAAAKLLAAFSILTFKASVEDEVRKVTRRVMDLNSRLGRPEEAVAPEFRGEELEQNKYEAENVEVDCI

ETIRRFVDIPFLVSTRTQHNESEDRGSRMVLDVTRVRFIDL-
VTKRSEIILVERAETDGVIARQMAKDLQALHPEAAALGKIAALQHFISVNDGHISNYVTAEINSVLGML
LLVLFIQQVRVSKVVWPTRREVTMVFILAALAVFFLVIGLSVNLVFMVGGISVAQITTLVLGEGVMAVR
QVSTFPSES-VTILQNTYADLICAGRVTKADIEKSLHIVVGARVARGTWYVIYHYEI-
QAQGPAGIMQRAFRQATLSQSLIDGRMEVKVKVVTADRYA-
THEIKGGQHRKGTRYNRVKVKHNIPVDLIARIKMAFAPPNLVLVKTFGAVVQKRFAQQLTHMRHGA
VSIGPMEVSSPYIRSKKARHAPGDSVVTIKPDNIVRSVAVVPELIWARENEDQSAVHDKYTKTTHKIAA
KRSRAVDKAQYDKRNQVHLSFIGLAVKVLPEQAIVHATAGTLMPTSSEAVTESLKGVAATIRLDPMEK
ALIAYIKRMRPQLNLGGQTDKKIVKCLNNNLDDVEYGKLGRRKIKPITIKSVYDVEHERATELVKLMPYK
DDVEGRKSQFAEK--
ALFEPLQAFMMLFGFIARAIYALYLRVIRRTILLAKTKIIVNASTVLSAIKIIGVSIRLRHKSTNIADTKHHIKT
IEKRARMTLAGSPSVKLHLSDGRVGTKGGRIKGYMPYKNYNIFDGLASGRRAKVKELAKLKKVDKSISV
DGIRTRQQGGRIPKADLSNAYANTRVGVDIVGENVQEESNPRANAEMGQIFAAIDARVYSLVGALIG
HALEAVLALCAILGIGRAHVLLAVRLKVNAGIELHSAA-VAQAVAAA-
YAQNGGQAAILLDVSEAYLGALAISQASVDVCRPTNRRLG-
LKKTKKEAYMSLTCFTGKGDKIKIVEASQTQAFEIVATRTAERFGIKAKGTYGKERAMMQIVPTKPVKV
WVPVFVNDDELLAPIRAFAETNGDGVTVVKLTKDIKVTMFEKGVASLAVTGKATSVIYTVLEKVTELMK
DIIYEQRAKAHYGRGRLRSHVVTHTKSQRHLSMIRRTVVADPSMEASRLTDGEVSVITGATMAFVMS
EQDAV--
GDVDMHFHEEPRREAQLGIAGPETFLATEQPRLLALLFLQEYLIHIVDIGTVGNFSDAATIYDILRAMAN
KLERKLSADIAKTRMCLADDVADMGVTELAARRESAGDPILSAYWINGVTANADIILLVANLESKLTPE
NIDCAVRLFYQRITLQAKERGGDIEVALVPAQNQIEAAEGTVIYTIKGKTPSSSQMKLTIEAISRSIVKSLA
VQLFQVDLFVNDTWLGTLLRAAGDVVYVDARDGKEGVDHVVERDTLLEFAPQKDIVARKIRIYTPAA
PSAVVIWDPAIEREDKADAQGTILMIYGPDLQDISCFTDIGAEAKIESLASWDEKEKYVVHRDDIPEDIT
TLVGKSSKVNKEFGIPDVAVCAPAKPGVTESIDRGEPKAGSCGEVANLPYPQESLVNRVSSANVQKNI
SMVLGGHPVGEMGISRGIERHLETNMCEIFVDKADVDVALDFYRLNLTAMRVGLDNPNAKVEIDGRE
SRKLIAVQDGDADLQRHPKLSEESGALRKAESKAKLPDDTLILDGVAHGDVSFTVTDLPEGRV-
VTPVNNAVKAQLEIDKVLLKTFHVVTLSFSNNKGEDHLIKLRLNALKKSTRHYEMLLKIRDTECATSPH
AISVGKILKIDSLLQTICTAHYTEIEPLMAQDLAALGVMGDQGLLHVVPRLMILAVLVLFGLEARSYVPK
WDTQAVAIITDQTMGLPTRGRGWSF-CISEVVGNTFFARTKDDDTEQRLHARNPI-VLL-
YGKMGMV-
ISPSGLRLDSTAPDAQGHGNENALILSRENLQDAMSYYVLHVLLVFVDLVVLAYGSGDRPLKFLVAN
QAVTGRKNPQLVCLTRRTFPLDLQRDATFVLVFEDATMKNLSHKKMHMNLVLIKSKGWVANVSTKIL
ISDQAIASYAEDAGAHTQPSESALAAITSRVMAPIVLVVSQKPTAPSKTLFLEALNLLDAVTATVLNKPK
RYFVFEADLVSILQSVDAAVLKGMQRPQPDRMRRAVVAQAIRLAIGLR-------
LEAGIINFSRAVTFAG-FYSALEC-GGGAIT-
EAIAFRVRNHKVNTKRARPYKGHTKFLHKADSDKALIADGVDTIA
>GCA_000014065
AVIKPVEINKYAPVLMIRQTQVATVSGAGSPSLRAKTVFRKSGLQRKTMTKWVQSPLYY-
GVLKMGNIVQLVSISVDNRAVCTVAQVVEQEVVVHGLVSLDAVKRKIALEEMDGMTTEDPAKVAEF
EEELDLKRFRDADARADMLPVMLGLTLQVRFDPHEPVVHDLPIPVIYEVI-
GIMADRGGIEVDDCAISNAVSDVDPIAYVSPIDLAAVAGEKMAYIDQEAAVGAAKEEIALEELALESVV
LVVGI-PLLLPV-FGADGDV-MSFAARSRTLESIVGLAVAAAELTRA-ID-GIASAM-
MTAAQAEALAEKRGTAEEIRIVKRMQRSSGLADDPDLVMLEDTAEGKASWAGQIYDDPISGSSESDS

LGNIVHAANKRQNFARSITFLRKVEISDKAAAPLMRGVRHNSKSRAARKVDDAIADLNFPNVPAGIAI
KALAHKSEDRLLHDATETEGILLIVVRWHVTWKTAVAIGLTFLAGDIVGLIALGAIAGIGKPDGLLNRHH
KLNGSLIEQRPIIEKMTLRLRASLKEKLGKDQIIIFVVKVQGDLVADATVQMG-
GPVGAVVAVQGDVIFVRRHKQTVITDSESTV-ILRGVAEGPADAEALLRIRVNHTLVELSVLLIPE-
PVLVDNVSIREGVGKSLQIIQLDVYLLRDMGTGEETLAESKSREVIREVLAIDPITGVRLAYGKLKERAGK
KTGLKDLETLF--
RSILAAVTEPRMENLPEGVCRTFITAGPRAAVGMLFLLLDEPGKLYPIDGELRELTKSVKSLISLVDAKAP
KVLSAGYAEERLRIRFADCEVLASEAELYAYKEERREMLRMVPVYIINEAKRRQLILFALSLKGGHKRAM
MALGVMLAAAIIESQADKGAVHHDEPAILLEDAEVTGTSIMVSRQASLELKHVAKVDVIVIFPDFQEK
MVVSLKFFCTEVAIFVIALIVTVGDMLSREPEHAQEDGAGYSKRIRGHSVVFALEVAFDRRNCRSAKLG
DLFLSLGLLPPRYGVFYTNQPKKKQMPSEPDGDRVDGFELVHAVKEDGSSNHELEMPEAMALAVEAT
ETINLSAGVLALSGGMVAIERVDSKRKVALAATKWNISRIDLLDVAISGAAPIRCCG-
IEECDLSAALGAKKV-
FLYLPGASNAELEEEKLGGAGNSTADVAEDRQREILLVHPVLVVIQDSNSAGLISNGGLDEWGTMAY
KKHYAFAARMRLHMRTIE-------MEPNKQKSEKVKTVKLEGDTRFRQLAIEGVKEQMP-
LHALNPDFAV-LPILVTPAVGSVAADAQVLGATVPVDASL-
RELQKSQKASALPISGIRLLDMRAARGMIEADIMVAGKIAA-
LFKIRALETIIGILLRNDSEATLPWRLSVAERLEADTKRASGFAARPFKTKPSLCTTQAHARIADPIQEIAA
GYVATEERFVFPVEDIAIRLILYKPHSRVGEKKRGGEGHLHSMAKELLGKAVVMLRNEPGTNTEGLLNY
LTTNQPTDLLFEDCLDVIIDIRRMSVV---
VHLNTV-
HVVVAGVAPVGDYEIMNAAFPSALIQVEQVDDRTGEEKTLMWMYGAMEELWLMSVRWADWVAN
CAIGAIILHVYAQTLNRAFSLKRLQLDGWAKEAARWWNTWQLWLNKDGFFGYEAAFRA-
KQILKAALADFEMKDVEGVGRYLSKQEIRAGYRIREQVDFARLDPRTDILLQAKTRLSIIRHALQVVAILI
LRDIAPEFT-
VVAYQDVARGHVECVLAESFDFTEKDAHIGPVGLRTPDPIYGDGTVFKEAVLGKIDLANTVAEFRYLVL
LDGGRELGVEWVTLQNMLYDRIWLPPAQAALKNLQPICFFLQHPELDM---------
ISKARADQLMAGAMIEEILEAVARFAEPEATTHQSKKAKHFKYYGEEVDPVHLAYILDLFALVATQVVL
SDRLLKDRYVLNLKV-
RALTSLWWMSLQLAEKGPCATIISVGTISGLGALVLFIILLMRDSVLVETLFYRYVYF-
MRGYSAAKDVQDADPEHLLELAAASRDIEWIEIIPAFFYYFDTAT-
FEPFNTAVAREFFGLFKERLRVIEVLAVPPDRVEALVEFVALAGTLGTSADAAPGVIAFGLCEPNAAGVL
EAERDWNLHRSMGITLAFFEARDSVKGYFDKGLHKAVAEFSLKEAGAMTTHFRCDVEAKQVVPAIHR
TLDLGDIEAALIEIDSAVVVSEEEQTLADKPTTRDKSVGAVGGVVLAGTMNMGANLDSVERGGNIIIEL
EEEQYHAVNGKKADGKLTDKVLMVLSVAYFPVIARFASANALDRGVFGLLIDAPTDADNLDHGRDM
SIPAPTVHAETAPLYAESDIDRIAWIAPMHLLGTLDAVLLFEPDFQGFISMMIDASLEIMETAEVMEDY
HVLEKGARPPGFRVVLIKGLHIRLLAAVGTLLVVGSAGRTEVTAYIQGRAVPISLRKQTFLPAFAPSSCIV
VDIFSTLLRPPERSSVEMLILETVAEL-
LEVTRGTNDKLERMQVLLPGLDMAELLDLLAAADDFADIVTIGAAITAILSVAAEAVVLREMLLTKAEIA
ITALALAMALLLMLTGDTGLTQKESAREAQMKDLIQAGAVLQLHADDAMVLAATADKVRLSTDEGT
TSAFASLNLATVSDGFYVLSSAVLQMSSDLMMIVSVARVLADKESKNALVVRHPEQVKTQPTYAEGS
PGMVLQTLTGCIPPLAIKPDEVELLWTLVAALWRAKAHLASAARPHNERVLILLLALGLIVVLLQSEGG
GMGGG-
ATAAAKLLAAFSILTLKAAVGDEVRKVTRRVMDLSSRLGRPEEAVNPEFKGEELDQTKYEAENVEVDC

METVRRFVDIPFLVSVRTQHNESEDRGARLVLDVTRVRFIDL-
VTKKSDVILVERVETDGIIARQMAKDMQSLHPEAAALGKVSALQHFIALGDAHISNYVTAATHSVLG
MLLLVLFIQQVRVSKVVWPTRREVTMVFIMAALAAFFVVIGLGVNIVFMVGGISPLQITTLVLGEGVM
AVRQTSTFPAES-
VTILQNTYADLICAGRVTKADIESSLHIVVGARVARGTWYVIYHYEITQAQSPAGIMQRAFRQATLSQS
LIDGRMDVKIKVVTSDRYA-
TYEIKGGQHRKGTRYNRVKVGHNIPVEVLARIKLAFGPPNLVLVKTFGAVVQKRFAQPLTHMRHGAV
TIGPMEVSSPYIKSKKARHAPGDSVVDIKPDNVVRSVAVVPELIWARENEDQSAVHEKYVKTTHKIAA
KRNRAVDKAQYDKRNQVHLSFIGLAVKVLPEQAIVEASAGTLLPTESVAVTENLKGVAATIRLDPMEK
ALIAYIKRMRPQLNLQGKTDNKIVKCLNNNLDDVEWGKLGRRKIKPITIKSVYDVEHTRATELVKLMPY
KDDVEGRKSQFAEK--
ALFEPLKAFMMMFGFIARAVYALSLRVVRRTILLVKTKIVVNANTVLSAIKIIGVSIRLRHKSTNVAETKH
HIKTIEARARMTLAGAPSVKLNLRPGRVGKKGGRIKGYMPYKNYNIFDGLASGRRAKVKELAKLKKVD
KTIAVDGIRTRQQGGRLPKADLSNALSNTRVDVEVVGENEQEESNPRANAEMGQIFAAIDARVYSLV
AALIGHALLAVLAICSILGIGKAHVMLAVKLKINAGIEMHSAA-VAQAVAAA-
YSQPAGEATAIADVSEGDLGALAITEDSLDVCRLRRRQLG-
LKKTKKEAYMSLTCFAGKGDKVKIIEATQTQAVEIVATRTAERHGIKAKGTYGKERAMMQITPTKPVKV
WVPVFVNEDELLAPVRAFAETNGDGVTVVKLTTDVKVTMFEKGVASLAVSGKVTTLVYTVLEKVTELS
KDIIYEQRAKAHYGRGRLRSHVVTHTKQKRHLSMMKRTVVADPSMDAARLTDGEVSVVTGATMAFV
MSEQDAV--
GDVDMHFHEEPRREAALGIAGPETFLATDQPRLLALLFLREYLIHIVDIDTVGNFSDAATIYDILRAMQN
KLDRKLSAEIAKVRMCLSDDVADMGVTDLAARRDTAGDAILSAYWINGVTANSDIILLVANLECKTVP
ENIDCAVRLFYQRITLQAKERGGDVSVRLVPAQNQIEAAEGTVIYTIKGNNPSTAQMKLNIEAISRSIVK
SLAVQLFQVDLFLNDTWIATLLRAAEDVVYVDAREGREAVDHVVERDTELELAPQKDIISRKIRIFTPAA
PSAVVIWDPAIDREDKADAQGTILMIYGPDLQDISCFTDIGSEPKIESLAAWDEKEKYVVHRDDIPEDI-
TLVGKSSKVNKEFGIPDVAVCAPAKPGVTESIDRQEPKAGTCGEVANLPYPQDSLVNRVSSANVQKNI
SMVLGGHPVGEMGISRDVERHLETNMCEIFVDKEDVDVALDFYKLSLTPLRVGLDKPNAKVQIDGKE
SRKLMAVRDGEAKLQRHPKVSEESGTRIGADEKAKLPDDALIYDGVQYGDLSFTATDLPAGRV-
VTPVFNAVKAQLAIDKVMLKTFHVVTLSFSNDKGEEHLIKLRLNALKKSTRHYEMLLKIRDTECAQSPH
AIPVGKILKIDALLQTICKAHYTAIEPLLADDLTALGVMGDQGLLHVVPRLMILAVLHVMDLEAEFQLST
WSLQEAALVTDQTMGLPLRDRAWSF-AISEVVGNTFFARSKDEDTHERLHARNPI-MIL-
YGKEGML-
ISPSGLKLASTKPTEDDHGNTNKLILSTERLQDSMSYHILAILLVFVDEVILAYGSGEDPLKFLVAEQAVT
GRANPQLVCLTRRTFPLDLQRDAVFVLVLEDTTMKNLSHKKMHMNLVLVKPKSWVENVSTKILISDQ
AIASYAEDAGAHTQPSESALAIITNRVIAPIVLVLSQKPAAPSKTMFLEALNLYDEITATVAKRPKRYFIFE
GDVISILGTLDAAVILGMQRPQPDRLRRAITARAIRLAIALRGSGVLAQLEGEAINFSRAVSLAA-VY-
DLTLLAQAAIPLPALDFRVRNHKTSTKRDRPYKGHTKFLHKAEEEKALVAEGVDTLS
>GCA_000018145
AVIRPITMDTYAPVLMIRQQQVATVSGAGSPSLRAKTVYAKSGLQRKTRTKWVTKPLYY-
GVLKMGKIVQLVSISVDNRTVCTVAQLVEQEVVVHGLVAFEAVKRKIALQEIEGMTAEDPAKVADYM
LEVEIKRLREAEDRADMLPVLLGLTIGIPFDPHQPVVHDLPVPVIYEVL-
GIMAEKGGIEVDDCAISTEVSDVDPINYVSPIDLAKVAGDQMAFIDQETAISAARDAIALDELALESSIL
VVGI-PLILPI-FGEDGDN-MSFAARARTLESIVDLAVSYPELPRA-IAE-IAAAM-
MARMRAAALASKKGTAEEIRIVKRMQRSGGLADNQDLVMLEDAAEGNSSWAGQIYDDPISGSSESD

SAGNIVYAANKRQNAERSITFLRKVEISDSADAPLMRGVKHNSKSRSKRKIDDAIATLNFPNVPEGLAL
RAEAGKPADTLLHDAAEGEKILLIIVRWHVTWKTAIIIMLTYTAGDIVGVIMLGGIAAVGKPDGLLNRH
HKLNGSLIEQRPIVEKLTLRLRASLKQKLGAEQIIIFVVKVSGDLVAEATVQM---
GVGAVVAIQGKVIFVRRHRQTLVTDSEATV-ILKGVAEGPADAQALLRIRVTHTLVELSVLLIPE-
PVLVESVSIREGVGKILQIIQLDVYLLRDMGTGTETLAESASRELIRSVLNIDPITGVRLAYGHLRERASKE
LTLKELETLL--
LSLRQDVTEPRMEDLPDGVCRTFIVAGPEAAVGMLFLLLDDAGRLYRLDGELRELPKSVKSMISLVDAR
KPKVLSAVYVEDRLRIRRADVEVLESEADLYAYKEERRELLRMVPVYIVLQAKRRQLVLFALSLKGGHRR
ARASLPVLLAAAILESQAVKGAVHHEAPATLLQDAEVVGSSMMVSRQASLELKAVAKVDVVVVFPEF
QEKMVVSLRFFCTEVAIFVIALIVTVGAMLSREPSHAQESGAGYQKRIGAHAALFALAVTFERANLGSD
ACPALFLGLG-LPLRYGVVYTNQPKKKQMPSEPDTEKVDGFELVHAIKEDGSK-
EELEMPEAMALAVEADATINLSAGVLALTGAMAAVERVDSKRKLSLAVEKWSIAPYDTLDLKLSVGAK
FRCLQ-IEEGNITAALEVKNV--
LYLPGASNAELEEEKLDGAGASTAEAAADRQSRILLVTPVLVVIQDSNAASLVANGGVDEWGTMAYK
KHYAFAARMRLHMRTID------
RMEPNKQKSEKVKTVKLEGDTRFRQLATEGVKEQMPVLHALNPDFAVLTPVLVTPAVGAVAAEEALL
GATVPCPFSL-RELQKSQAGAALPISGVRILPMRAARAMIDEDIFVAGKVAA-
LLAILALETVMALILRNDSEARIPWRLSVAERLEADTARASGFAARPFRSKPSLCDTQAHAKSAEPIQEI
ATGYVAVEQRFVFPVEGIPIQLILTSPHSRVGAAKRAGEGHLHEMAKELLARAVLMVQNEPGSNTEGA
LKYLTTNQPTDLLFREALDLVIDLRRMTVV---
IQLTTV-
HIIIEGARDLGARQIMAAAAESGLVQVERIDTLPGPERTAMWMYGSMEDLWLMSVTWADWVPDCA
IGAIILAVYAQAINRAFSLKRLQLDGWPKEAARWWNTWQLWLNKDGFYGYDAAFRAVKQVLKAALF
DYAMSDVQGVGRYQSRQELRVGYRIREQVEFERLDPRRDILLQAKTRLSVIRHALAVVSILILREVVGEF
T-
VVAEEDVAMGHVECVLAESFDFTAKDADIGPPGLRTPDPIFGDGSVFQEAVLGKVDLANTVADFRYR
MIIDGGRRLGVSWTPLTNMLYPRIWLPPAQAALKDLLPICFFLGHPELDLEMWE-LFA-
ISKARAARLMAGALVERILEKVAAFAAPEATTHQSHKEKHFKYYGEEVDPVYLAYVLELFALVATQVTV
SDRLLKDRYVLNLKV-RSLTALWWVSLNLGGKSPCATVITVGTVSGLGALVIFIILL-
RDSVLVETLFYRYVYV-MRGYNAAKDVADADPEHLLSLATVTRDVEWIEIIPAFFYYFRTAG-
FQPFNTAVAREFFGLFAERLRLVAVLAVPPPRVEVLVEFVAWAGTLATSAEALPGVIACGFCEPNAAA
VLEADRDWNLHRAMGITLAFYEARESVKGWFDKGLNKDVAETGLSEAGGLSTPFHCALTAKRCVPVI
HRVLDLGEIEAALIEIDSAVKVADEEQNLADQPTTREKAVGAVGGVILAGTMNMGASLDQVERSGNII
IELEEEQYHAVNG-
KADGKLTDKVLMVLSEAFFPVIRRTSSANALDKGVFGLLVPAPDDADNLDHGGQLVIPAATIHGEQA
ALFAEDEIDKIAWIAPLHLLGTLPAVLLFEPDQYSFISMMIDASLEIMETAEVLDDYHVLETGSRPPAFRV
VLIRGLFLNRLGAIGTIIVVGSAGRSEATAYIAGGAVPITLRKATFLPAFAPSSCVAVDILSTLLRPPERSAV
QMLILETVTEL-
LVVTRFENEKLERLLVLLPGLDVAELLGLLAAAADFATIVTIDAAVVAILSVAAEAVVLRELLLSMIEIAITA
LGLAIALLFMLTRDMGLTQKEAAREEQVKELIQGGVVLTVHASADMVLASTADRVRLDTDEGTTSAF
ASLNLATVSDGFYVLSSAVLQLSADMMMIVEVARVLADKENENALVVRHPEQVKTQPTYAEGSPGM
VLQTLTGAVPPLAIKPQEVELLWTLVAALWEAKAHLASATRPHAERILVLLLALALIVVLLQSEGGGMG
GG-
ATSAAKILAAFSILTLKAAVGDEVRKVARRVMDLNSRLGRPEEAVTPEFRGAELEQTKFEAENVEVDCL

ETIRRFVDIPFLVSVRTQHNESEDRGARLVLDVTRVRFIDL-
VTKKSEIILVERAETDGVIAREMKGDLQALHPEIAALGKIAQLQHYISLGDGHISNYVTQDIHSVLGMVL
LVLFLQQVRVSKVTWPTRKEVSMVFAMAILAIFFFVIGLFVQLVFMLSGISPLQITTLVLGEGVMAVRQ
TSTFPEDS-
IGVLQNTYADLICAGRVTKADIEASLHIVVGARVARGTWYVIFNYEINQAQAPDGIMQRAFRQATLSN
SLIDGRMEVKIKVVTSDRYA-
TYEIKGGQHRKGTRYNRVKVGHNIPVELLPRVKMAFGPPNLILVKTFGAVVQRRFAQPLTHIRHGAVTI
GPMEVASPYIRMKKSRHAPGDSAVDLKPDNIVRSVAVVPELIWARENEDNSAVHEKYTKTTHKTAAK
RNRAVDKAQYDKRQQVHLSFIGLAVKVLPEQA--
--
RMRPQLNLQGKADHKIVKCLNNGLDDVEWGKLGRRKIKPITIKSVYDVEHERATELVRLMPYKDDVE
GRHSKFAEK--
AIFEPLKAFMMLFGFIARAVYALSLRVVRRTILLVKTRIVVNADTVLSAIKIIGISIRLRHKTTNVADTNHYI
KTIEKRARMTLAGAPSVKLNLRPGRVGMKGGRIKGFMPYMNFNIFEGLASGRRAKVKDVAKLKKVDK
EITVDGIRTRQ-
GGRIPQADLSNALSNTRVDVEVTGETIQEESNPRANAEMGQIFAAIDARVFALVGALIGHALEAILALC
AIMGIGRAHALLAVRLKINAGIEFHSAA-VPRAIAAA-YSQHGGDA-
AIADVSEGDLGDLSVTEATIAVCMARARQLQE---
-
RFGIRAKGTYGKERAMMQILPTKPVKVWVPIFVSEPELLAPIRAVAETCGDGVTVVKLTKDIKITMFEKA
VASLAVSGKATTVVYTVLEKVTELMKDIIYEQRAKAHYGRGRLRSHVVTHTKSKRHLSMMRRTVVADP
SMDAARLLDGEVSIITGATIAFVMSEQDAV--
GDARMHYHEEPRREAELGIAGPETFLATEQPRVICVLFLKEYLIHIVDIGTTGGFADAATIYDILRAMQN
KLDRKLSADIAKTRMCLHDDVKDMGVTAEAPRRDAAGDPILSAYWINGVTANADIILLVANLECATA
PENIDCAIRLFYQRITLQAKERGGDVTVALVPAQNQIEAAEGTVIYTIKGSTPSTVQMRLNIEGVSRSIV
KSLAVQLFQVDLFTNDTWIDTLLRAARDVVYVDARDGKEAVDHVIERDTELELAPQKDIIRRKIRIYTPA
APSAVVIWDPAIDRADKADAQGTIIKIYGPDLQDISCFTDIGSEPKIESIKSWDEKEKYVVHRDDIPEDI-
TMVGKSSRVNKEFGIPDVAVVAPAKPGVTESIDRQEPKAGSCGEVANLPYPQESLVNSVSSANVQKN
ISMVLGGHPVGEMGISRAIERHLETNMCEIFVDKDDIDVALDFYLIDLT-
LRVGLDPPNAKVEIGGRETRKLMAVRDGEAKLQRHPKLSEANGTG-
KGGSKARLPDDALIHDGVHHGDLKFTVTDLPEGRV-
VTPVFNAVKAQLSIDKVLLRSFHVVTLSFSNDKGDDHLIKLRLNALKKNTRHYEMLLKIRETECAESPHA
IPVGKILKIEALLQTICQGHYTAIEPLLADDLTVLGVLGDQGLLHVVPRLMILAMLHLWGLEIALTIPTWA
LQADACVTDQTLGLRVRARAWSF-AISEVVGNTFFARTKAGETEQRLHARNPI-MIL-YGKEGLV-
ISPSGLKLASTAPDNLGHGNRDKLVLSKERLQDGMSYYIIRVLLVFVDEVLLGYGSGDTPLKFLVADQA
VTGRDNPGLVCLTRRTFPLDLQRDATFVLVFEDSTMKNLSHKKMHMNLVLIKSKGWIENVSTKILISD
QAIASYAEDAGAHTQPSESALAVITARVIAPIVLVVSQKPTAPSKTLFLEALNLLEAVTCTILNKPKRYFIF
EGDLVSVLVAIDAAVLVGMQRPQPDRMRRAIVATAVRLAIAQA-------
LEAGILNFARAVSLAVRAYPLMTLLPAAAIA-
PIIAFRVRNHKVSTKRARPYKAHPKFLHKADTEKALVADGVDTLA
>GCA_000152605
AVIKPVIQNEYAPVLMIAQTQVATVSGAGSPSLRAKTVYAKSALSRKTMTKWVAAPIYY-
GVLKMGNIVQLVSISVDNRSVCTVAQLVEQEVVVHGLVALAAVKRKIALDEIDGMTTEDPAKVAQFE
EELDLKRFRDADDRADMLPVMLGLTLKIKFDPHQPVVHDLPIPVIFEVV-

GVMADRGGIEVDELAISQSISDVDPIEYVSPIDLAAVAGDAMAFIDQEAAVAAARETIALEELALEASIL
VVGIKPLLLPV-FGDAADV-MSFANRSRTLGSIVNLAVAFPEIKRA----AFAADM-
MRAAQAEALAEKRGTAEEIRIVKRMQRSAGLADDPDLVILEDAAEGNSNWAGQIYDDPISGSSESDS
AGNIVY-
ANKRQNFQRSITFLRKVEISDKAAAPLMRGVKHNSKSRASRKVDDAIATLNFPNVPEGIAIAAAAGRP
ADTLLHDATEREEILLLIVRWHVTWKTAAIVILLVEAGDIVGLITLGGIAAVGKPDGLLNRHHKLNGSLIE
QKPIIEKMTLRLRASLKQKLGKDQVIIFVVKVQGDLVADATVQM-
GGPLGAVVAVQGEVIFVRRHKQTVIGDQETTVEVLKGVAQGPADAEALLRIRVSHTLVKISVLLIPQ-
PVLVDAVSIREGVGKALQIIQLDVYLLRDMGTGEETLAESVSRETIRDVLAVDPITGVRLAYGDLKERLA
KKLVLKDLET----
LSIMADVTEPRMQDLPAGVRRTFITAAPRAAVGMIFLVLDEAGKIYPIDGDLRELQKSVKSLIGLVDAK
VPKVLSAGYATQRLKIRFADCEVLNAEADLYAYKEERKELLRMVPVYIILEAKRRQLIVFLLSLRGGHRRA
MAAIGVMLAAAILESQAEKDPVNHAQPAILLDDAEVVGTAIMVSRQAALQVKDRSKVDVIVVFPEFQ
EKMVVSLKFFCTEVAIFVIALITTVGEMLSREPEHAQEDGAGFGKRIRDHSMLFALRVVFERTNLRSDAT
PDLFLGMGLAPPRYGILYTNQPKKKQMPTEPDSDRTDGFELVHAVKEDSASNHELEMPEAMALAVE-
DAPINVSGGVLAMTGGMVAVEKVDSKRKMSLAATKWTISKVPMLDIHLSAASQFRCLE-
IELADLSASLGLKAD--
LFLPGASNEELEEDKLNGAGASTADVAADRQGSILLVIPVLVVIQDSNTAGLIANDGLDEWGTMAYKK
HYAFAARMRLHMRTIDAIEVGVEMVPNKQKAEKIKTVKLDGDTRFRQLATEGVKEQMPVLHALNPD
FAV-LPLLVTPAVGSVAADESVLGARVPVDASL-
RELQKSQKGNALPISAIRLLDMRAARGMIDEDIMVAGKVAA-
LLKISALETVIAVVLRNDSEAGIPWRLSVAERLEADTRRAGGYAARPFKSKPSLCETQAHAKIADAIQEIA
ATYVAKEKRFVFPQEGIAIQIVLNSPHSRVDKDKRAGEGHLHEMAKELIAKAVIMIRNDPDTNSEGLLN
YITTNQPTDLLFEDCLDVVIDMRRMSVV--
VHLNTV-
HVVVEGVRDVGNYEIMAAQAPSGLVQTERVDDRTGRERTIMWMYGAMEELWLLSIRYADWVTDC
AIGAIILHVYAQQINRAFSLRRLQLNGWPPEASSWWNTWQLWLNKDGFFSYDAAFKAVKQVLKAAL
ADFEMKDVEGVGRMLSRQEVRAGYRIRQQVDFARLDPRSDILLQAQTRLSVIRHALTVVAILILREVAS
EFTLVVAMEDVAMGHVECVLADCFDFTAKDADIGPAGLRTPDPIFGDGTVLNEAVLGKIDLANTVAD
FRYLMLVDGGRQLGVEWLTL-
NMLYDRIWVPPAQAALKNLTPICMFLSHPELDLAATETRFAGISKARAEQLMAGALIEEILEGVARFAD
PEATTHQSSKEKHFKYYGEQVDPVHLAYIAGNSALVATQVTVSDRLLKDRYVLNLRVFRGFTSLLWM
AAQLAEKGPSATVITVGTVAGLGALVLFIILL------------------MRGYQAAKDVADADPEHFL-
LASASRDVEWIEIVPAFFYYFATATSRLEKNTAVAREFFGLFKERLRVIDVLAVPPDRVEALVEFVALAGT
LTTSAEA-
PGVIAFGLCEPNAAGVLEAERDWNLHRTMGITLAFFKAQVSVKGWFDKGLHKDVAQTGLREAGGFS
FPFHAKVEAKRCVPVVHRVIDLGELEAGLIEIDSAVVVADEEQTLADQPTTRDNSVNAIGGVVLAGTM
NMGASLDSVERAGNIIIELEEQQYSAVNGKKADGKMTNAILMVLSRAYFPVIARTASANALDKGVFNL
LIQAPSDADNLDHGREMVIPAPTVHSETTPLYAESDIDKIAWIAPIHMLGTLAAVLVFEPDHYSFISMM
IDASLDITETAEVLDDYHVLEKGARPSAFRKILIRGLHIRLLAAVGTIIVVGSAGRDEVTAYIQGQAVPIT
MRKATFLPAFAASSCKTIDIFSTMLRPPERSSIKMLILETVTEL-
LEVTRGENDKLERILILLPGLDLAVIMGLVAAAKDFAQIVTVDAAITAILSVAAEAVVLREMLLTKAEIAV
TALALAMALLLMLTADKGLTQKEQAREKQMKELIQGGVVLQIRASDAIVLAATADKVRLSTDEGTTSA
FASLNLATVSDGFYVMSSAVLQLSSDLMMIVEVSRVLADKETKNALVVRHPEQVKTQPTYAEGSPGM

VLQTLTECIPPLAIKPEEVELLWTLVAALWRVKAHLANAARPRLERVLVLILALGLIVVLLQSEGGGMGG
GAVGAAAKVLAAFSILTIKATVEDEVRKVTRRVMDLNSRLGRPEEAVAPEFRGEELEQNKYEAENVEV
DCMETVRRFVDIPFLVSTRTQHNESEDRGSRMVLDVTRVRFIDL-
VTKRSEIILVERAETNGIIARQMAKDLQALHEEAAALGKVASLQHFISLNDGHISNYVTAQVNSVLGML
LLVLFIQQVRVSKVVWPTRREVTMVFILAALAVFFIVIGLYINLVFMVGGISPLQITTLVLGEGVMAVRQ
VSTFPAES-VTILQNTYADLICAGRVTKADIEKSLHIVVGARVARGTWYVIYHYEI-
QAQGPAGIMQRAFRQATLSKSLIDGRMEVKVKVVTSDRYA-
THEIKGGQHRKGTRYNRVKVGHNIPVDLLARIKMAFAPPNLVLVKTFGAVVQKRFAQQLTHMRHGA
VTIGPMEVSTPYIKAKKARHAPGDSVVNIKPDNIVRSVAVVPELIWARENEDQSAVHDKYTKVTHKIA
AKRNRAVDKAQYDKRNQVHLSFIGLAVKVLPEQAIVHATAGTLMATTSEAVTENLKGVAATIRLDPM
EKALIAYIKRMRPQLNLAGKTDKKIVKCLNNNLDDVEYGKLGRRKIKPITIKSVYDVEHERATELVKLMP
YKDDVEGRKSQFAEK--
ALFEPLSAFMMMFGFIARAVYALYLRVIRRTIVLVKTKIVVNAATVLSAIKIVGVSIRLRHKSTNVAETKH
HIKTIEARARMTLAGSPSVKLHLSEGRVGTKGGRIKGYMPYKNYNIFDKLSSGRRAKVKELAKLKKVDK
SISVDGIRTRQQGGRIPKADLSNALANTRVGVDIVGENEQAASNPRANAEMGQIFAAIDARVYHHV
GALIGHALAAVLAICAIQGIGKAHALLAVKLEINAGIEFHSAA-VAIAVAAA-YAQQAGEA-
ILADVSEPNLKALAVTEASVDVIR--RRKLG-
LKKTKKEAYMSLTCFAGKGDRVKIVEASQTQAFEIVATRTAERHGIKAKGTYGKERAMMQIVPTKPVK
VWVPVFVAEDELLAPIRAFAETNGDGVSVVKLAKDIKVTMFEKGVASLAVTGKATTVVYTVLEKVTQL
MKDIIYEQRSKAHYGRGRLRSHVVTHTKSKRHLSMMRRTVVADPRMDAARLTDGEVSVITGATMAF
VMSEQDAV--
GDVDMHFHEEPQREAQLGIAGPETFLATEQPRLLALLFLRAYLIHIVDIETQGGFADAATIYDILRAMQ
AKLDRKLSADIAKTRMCLHDDLADMGVTQVAARRESAGDTILSAFWINGVTANADIILLVANLECKTV
PENIDCAIRLFYQRVTLQARERGGDVSVELVKAQNQIEAAEGTVIYTIKGKTPSTTQMALVVESISRSIV
KSLAVQLFQVEHFLNDTWIATLLRAAAEVVYVDARGGDEAVDHVVERDTELEFAPQKDIVARKIRIYT
PAAPSAVVIWDPAIEREEKADAQGTILMIYGPDLQDISCFTDIGAEAKIESLKSWDEKEKYVVHRDDIPE
DISTLVGKSSKVNKEFGIPDVAVCAPAKPGVTESIDRGEPKAGSCNEVANLPYPQESLVNRVSSANVQ
KNISMVLGGNPVGEMGISREIERHLETNMCEIFVDKDDVDVALDFYRIALTALRVGLDPPNAKVEIDGK
ESRKLIAVLDGDADLQRHPKLSEESGALRKADTKARLPDDTLIYDGVQHGDTAFTATDLPEGRV-
VTPVNNTVKAQLKIDKVMVRTFHVVTLSFSSSKGEDHLIKLRLNALKKSTRHYEMLLKIRDTECATSPH
AISVGKILKVDSLLQTICVAHYTEIEPLMAEDLTALGVMGDQGLLHVVPRLMILAVLHLYGLEAAMQVP
RWSLQEKAIVTDLTMGLPVRDRAWSF-CISEVVGNTFFARTKDDDTEQRLHARNPI-VLL-
YGKTGMV-
ISPSGLRLDSTAVADMDRNNKDQLILSKERLQDAMGYYVLKILLVFVDLVILAYGSGDLPMRFLVADQ
AVTGRNNPQLVCLTRRTFPLDLQRDATFVMVFADATMKNLSHKKMHMNLVLIKSKGWVVNVSTKIL
ISDQAIASYAEDAGAHTQPSESALAAITSRVMAPIVLVVSQKPTAPSKTLFLEALNLLDEVTATVLNKPK
RYFVFEGDVVSVLATVDAAVLKGMQRPQPDRMRRAVVARAIGLAIAIQASGIIASLEADVINFSRAVSL
AAGVFDDMDLVPQAATR-PALAFRVRNHKVSTKRARPYKGHTKFLHKTDTDKALIAEGVDTIA
>GCA_000152625
AVIKPVSINVYAPVLMIRQTQVATVSGAGSPSLRAKTVYRKSGLGRKTMVKRVAEPIYF-
GAMKMGKIVQLVSISVDNRAVCTVAQVVEQEVVVHGLVALEAVKRKIALDAIDGMTTEDPAKVAEFE
EEIDLQKFRDADARADVLPVMLGLTLQVKFDPHEPVVHEIPVPVIFEVL-
GIMAERGGIEVDECAISTEVSDVDPIAYVSPIDLAAVAGKEMAFIDQEAAVAAARTAIALEELALEASILV
VGIDPLLIPV-LYADGDV-MSFANRSRVLESIVDLAVLIVQQSRAR--AGIAAEM-

MTERQAEALAEKRGTAEEIRIVKRMQKSAGLADDPELVIMEDAAEGNSSWAGQIYDDPISGSSESDSA
GNIVVAANKRQNFARSITYLRKVEISDKASAPLMRGVKHNSKSRSARKLDDAIAGLNFPNVPEGIAIAA
AAGKPEDTLLHDATEAEVILLIVVRWKVTWKTAIIIALLFLAGNIVGLIQLGGIAAVGKPDGLLNRHHKL
NGSLIEQRRIVEKLTLRLRAALKQKLGKEQVIIFVVKVQGDLVADAKVQMG-
GPLGAVVAVQGDLIFVRRHRQTLITDQEATV-ILKGVAEGPADAQALLRIRVTHTLVKLSVLLIPE-
PVLVDAVAIREGVGKSLQIIQLDVYLLRDMGTGSETLAESASRELIREVLMIDPVTGVRLAYGKVQERIG
RKLGLKELQTLF--
LAILEDVTEPRMENLPEGVCRNFVEAGPRAAVGMLFLLLDEAGRLYELDGDLRELTKGIKSLIGLVDAKA
PKVLSASYATARLKIRYADCEVLEAEADLYAYKEERKELLRMVPVYIINAAKRRQLVIFALSLRGGHRRA
MAAIGVLIAAALIESQARRDDVHHATPHVLLDDAEIVGTAIMISRQAALELKQVARIDIIVVFPEFQEKM
VVSLKFFCTEVAIFVIALITTVGEMLSREPEHAQEDGAGFQK----HSALFALSVTFERTNLKDA--
PDLFLSLGLASPRYGIIYVTQPKKKQMPTEPDKERVDGFDLVHAVKADGRS-
QELEMPEAMALAVEADATINLSGGVLALKGGMTAVEKVDSKRKMSLAAEKWNIARLNAEDIHISGAA
QFRCTS-IEQADLSAALGVKDLK-
LYLRGASNAELEEANLDGAGASTADAAEDRQTSILLVSPVLVVIQDSNTAGLVSNGGVEEWGTMAYK
KHYAFAARMRLHMRTID------EMEPNKQKSEKVKTVKLEGDTRFRQLSIGGVKEQMP-
LHALNPDFAVLVPLLVTPAVGQVAADEAVLGADVPCPFSL-
RELQKSMRGAALPITAIKLLAMRAARAMIDEDIMVAGKVAA-
LLKVGALETVIAVVLRNDSEATLPWRLSVAERLEADTKRASGFASRPFMSKPSLCDIQAHANVADPIETI
AAGYVAKDQRFVFPVEGLAIRLILSSPHSRVGEAKRAGEGHLHAMAKELHAKAVMMLKNAPGTNKE
GLLNYMTTRQPTNLLFEDCLDVVIDLRRMSVVLSADLCECLILLILLNIKQIISPLVITKQGAASLNTVVVE
SLVHLNTV-
HVVVKGVREVGDYEIMAAAAPSGLVQVEQVDDRTGRERTIMWMYGAMEELWLMSLRYADWVQD
CAIGAIILHVYAQTINRAFSLKRLQLNGWPKEADRWWNTWQLWLNKDGFFGYEAAFRAALQVLKSA
LFDYEMSNIEGVGRYQSRQQLRVGYRIRQQVEFARLNPRTDILLQAQTRLSIIRHALTVVAILILRDVVG
EFSLVVAQEDVAMGHVECVLADCFEFTEKNAHIGPVGLRTPETILGDGTVLSEAVLGKIDLANTIADFR
YLVIVDGGRALGVWWISLENMLVGRIWLPPAQAALKDLQPICFFLSHPNLDLTL-
DPLFEGISKARAEKLMAGALIEEILETVARFADPEATTHQTHKERHFKYYGEEVDPVHLAYILELFALVAT
QVVISDRLLKDRYVLNLKVFRTFTALLWLGLGLAHKGPAATVITVGTIAGLGALILFIILL-
RDSVLLETLFHRYVY--MRGYSAAKDVADADPEHLLALAAVSRDVEWVEIIPAFFYYFDTAT-
FEPFNTAEAKEFFGLFEERLRVVDVLAVPPLRVEELEEFVALAGTLPTSADALPGVIAVGLCEPNAASVLE
ADQDWNLHRSMGITLAFYKAQESVHGWFDKGLHKEVAATGLRDAGGFTTHNLCEVEAKKCVPAVH
RALDLGEIEASLIEIDSAVVVADEEQTLADTPTTRDKAVGAVGGVVLAGTMNMGASLDQVERGNNIII
ELEEEQYSAVNGKKADGKLTDAVLMVISLSYFPVIAGLASANALDRGVFDLLIQAPADADNLDHGRNL
TIPVPTIHTETAPLYAEADIDRIAWIAPLHMLGTLPAVL-
FEPDQYSFISMMIDASLEITETAEVAEDYHVLEKGARPSAFRVILIKGLHIRHLAAVGTILVVGSAGREEIT
AYIQDRAVPITLSKVTFLPAFAPSSCKVVDIFSTMLRPPERSTVQTLILETVTEL-
LEVTRGSNDKLERLKMLLPGLDAAELLQLRAKAADFSAIVTIEASITAILSVAAEAVVLRELLLTKAEIAITA
LALALALLLMLTPDRGLTQKEIARDGQLKALIQGGAVLQVHAADAMVLAATADKVRLDTDEGTTSAF
ASLNLATVSDGFYVLSSAVLQMSSDLMMVVAVARVLADKETKNALVVRHPEQVKTQPTYAEGSPG
MVLQTLTTAIPPLAIKPEEVELLWTLVAALWRVKAHLAEAQRPRNERVLILLVTLALIVVLLQSEGGGM
GGG-VGSAAKVLGGLSILTIKASVADEVRKVTRRVMDLNSRLGRPEETVNPEFRGEELEQAK--------
-----------------------------------DVTRVRFIDL-
VTKRSEIILAARAETDGIISREMAKDLQALQPEAAALGKIAALEHFIALNDGHISNYVTADIHSVLGMLLL

VLFIQQVRVAKVVWPTRREVTMVFIMATLAIFFLVIGLSVKLVFMLGGISPLQITTLVLGEGVMVVRQV
STFPAES-
VTILQNTYADLICAGRVTKADIEASLHIVVGARVARGTWYVIYHYEITQAQGEAGIMQRAFRQATLSQ
SLIDGRIEVKVKVVTSDRYA-
TYEIKAGQHRKGTRFNRVKMGHNIPVDLLARIKMAFGDPNLILVKTFGAVVQKRFAQPLTHVRHGAV
TIGPMEVASPYITMKKARHAPGDSVVDIKPDNIVRSVAVVPELIWARENEDQSAVHEKYVKTTHKIAA
KRNTAVDKAQYDKRNQVHLSFIGLAVKVLPDQAIVHATSGTLLPTESVAVTENLKGVAATIRLDPMEK
ALIAYIKRMRPQLNLAGKNDQKIVKCLNNNLDDVEYGKLGRRALKPLTIKSVYDVEHKRATELVKLIPYK
DDVEGRKSQFADK--ALFEPLKAFMMLFGFIARAVYALSLRVVRRTILLAKTKVVVNANTILS-
IKIIGVSIRLRHKTTNVADTNHHIKTIEARARMTLAGTPSVKLNLRPGKVGMKAGRIKGYMPYKNFNIFD
KLESGRRAKVKDLAKLKKVDKTITVEGIRTRQQGGRLPKADLSNAFANTRVGVDIVGENEQEESNPRA
NAEMGQVFGAIDARVYSHVGALIGHALTAILAICAILGIGRAHALLAV-LKINAGIEFHSAA-
VERAVAAA-YAQPAGEAAAIADVSEADLGDLSLSEQSTDVCRMRRRQLG-
LKKTKKDAYMSLTCFTGKGDKVKIIEASQTQAYEIVATRTAERHGIKAKGSFGKERAMMQITPTKPVKV
WVPVFVAEDELLAPIRAFAETNGDGIDVVKLTTDIKVTMFEKAVASLAISGKSTTIVYTVLEKVTELSKDII
YEQRAKAHYGRGRLRSHVVTHTKQKRHLSMMKRTVVASPKMDAARLHDGEVTVVTGATMAFVMS
EQDAV--
GDVDLHYHEEPRIEAELGIAGPETFLATEQPRVLALVFLRAYLIHLVDIGTVGGFADAATIYDILRAMQN
KLDRKLSAEIAKVRMCLHDDVAGMGVRAEAPRREAAGDSILSAYWINGVTANADIIMLVANLECKAV
PENIDCAVRLFYQRVTLQAKERGGDVRVALVPAQNQIEAAEGTVIYEIKGKTPGTAQWKLNVEAISRSI
VKSLAVQLFQVEHFVQDTWIDTLLRAAQGVVYVDAKEGREAVDFVVERDTELELAPQKDIVRRKIRIYT
PAAPSAVVIWDPAIERADKADAQGTILKIYGPDLQDISCFTDIGAEPKIESLKSWDEKERYVVHRDDIPP
DI-
VMVGKSSKVNKEFGIPDVAVCAPAKPGVTESIDRGEPKAGTCGEVANMPYPQESLVNRVSSANVQK
NISMVLGGHPVGEMGISRKVERHLETNMCQILVDKADVDVALDFYKLQLTPLRVGLDRPNAKVQIDG
KESRKLMAVLDGEADLQRHPKLSEAGGTG-TAGQKARLPDDALIFDGVLHGDIKFTATDLPEGRV-
VTPVANNVKAQLDIDKVLLKTFHVVTLSFSSDKGEENLIKLRLNALKKSTRHYEMLLKIRDTECAQSPH
AIPVGKILKIDDLLQTICQAHYTEIEPLLADDLTALGVLGDQGLLHVVPRLMILAVLLGWELAAEALLPE
WDLSARAILTNQTMGLGVRSRAWSY-AISEVVGNTFFARTKDDDTEDRLHARNPI-VLL-
YGKMGMV-
ISPSGLRLASTAEDAQQHGNESKLILSAERLQDAMEYFVLRVLLVFVDEVILAYGSGDEPLKFLVAEQA
VTGRANPSLVCLTRRTFPLDLQRHIVFVLVFEDSTMKNLSHRKMHMNLVLIKSKGWVVNVSTKILISD
QAIASYAEDAGAHTQPSESALAVITSRVIAPIVLVVSQKPVAPSKTLFLEALNLLDEVTATVLNRPKRYYI
FEGDVISVLGGVDAAVILGMQRPQPDRMRRAVVAEAIRLAIAARGSGVLAMLEAGHINFSRAVSFIA-
AY----------------DFRVRNHKTSTKRDRPYKAHPKVLHKAEDAKSLIAEGVDTLA
>GCA_000152645
AVIKPVIQNEYAPVLMIAQTQVATVSGAGSPSLRAKTVYAKSALSRKTMTKWVAAPIYY-
GVLKMGNIVQLVSISVDNRSVCTVAQLVEQEVVVHGLVALAAVKRKIALDEIDGMTTEDPAKVAQFE
EELDLKRFRDADDRADMLPVMLGLTLKIKFDPHQPVVHDLPIPVIFEVV-
GVMADRGGIEVDELAISQSISDVDPIEYVSPIDLAAVAGDAMAFIDQEAAVAAARETIALEELALEASIL
VVGIKPLLLPV-FGDAADV-MSFANRSRTLGSIVNLAVAFPEIKRA----AFAADM-
MRAAQAEALAEKRGTAEEIRIVKRMQRSAGLADDPDLVILEDAAEGNSNWAGQIYDDPISGSSESDS
AGNIVF-
ANKRQNFQRSITFLRKVEISDKAAAPLMRGVKHNSKSRASRKVDDAIATLNFPNVPEGIAIAAAAGRP

ADTLLHDATEREEILLLIVRWHVTWKTAAIVILLVEAGDIVGLITL------------
LNRHHKLNGSLIEQKPIIEKMTLRLRASLKQKLGKDQVIIFVVKVQGDLVADATVQM-
GGPLGAVVAVQGEVIFVRRHKQTVIGDQETTVEVLKGVAQGPADAEALLRIRVSHTLVKISVLLIPQ-
PVLVDAVSIREGVGKALQIIQLDVYLLRDMGTGEETLAESVSRETIRDVLAVDPITGVRLAYGDLKERLA
KKLVLKDLET----
LSIMADVTEPRMQDLPAGVRRTFITAAPRAAVGMIFLVLDDAGKIYPIDGDLRELQKSVKSLIGLVDAK
VPKVLSAGYATQRLKIRFADCEVLNAEADLYAYKEERKELLRMVPVYIILEAKRRQLIVFLLSLRGGHRRA
MAAIGVMLAAAILESQAEKDPVNHAQPAILLDDAEVVGTAIMVSRQAALQVKDRSKVDVIVVFPEFQ
EKMVVSLKFFCTEVAIFVIALITTVGEMLSREPEHAQEDGAGFGKRIRDHSMLFALRVVFERTNLRSDAT
PDLFLGMGLAPPRYGILYTNQPKKKQMPTEPDSDRTDGFELVHAVKEDSASNHELEMPEAMALAVE-
DAPINVSGGVLAMTGGMVAVEKVDSKRKMSLAATKWTISKVPMLDIHLSAASQFRCLE-
IELADLSASLGLKAD--
LFLPGASNEELEEDKLNGAGASTADVAADRQGSILLVIPVLVVIQDSNTAGLIANDGLDEWGTMAYKK
HYAFAARMRLHMRTIDAIEVGVEMVPNKQKAEKIKTVKLDGDTRFRQLATEGVKEQMPVLHALNPD
FAV-LPLLVTPAVGSVAADESVLGARVPVDASL-
RELQKSQKGNALPISAIRLLDMRAARGMIDEDIMVAGKVAA-
LLKISALETVIAVVLRNDSEAGIPWRLSVAERLEADTRRAGGYAARPFKSKPSLCETQAHAKIADAIQEIA
ATYVAKEKRFVFPQEGIAIQIVLNSPHSRVDKDKRAGEGHLHEMAKELIAKAVIMIRNDPDTNSEGLLN
YITTNQPTDLLFEDCLDVVIDMRRMSVV--
VHLNTV-
HVVVEGVRDVGNYEIMAAQAPSGLVQTERVDDRTGRERTIMWMYGAMEELWLLSIRYADWVTDC
AIGAIILHVYAQQINRAFSLRRLQLDGWPPEASSWWNTWQLWLNKDGFFSYDAAFKAVKQVLKAAL
ADFEMKDVEGVGRMLSRQEVRAGYRIRQQVDFARLDPRSDILLQAQTRLSVIRHALTVVAILILREVAS
EFTLVVAMEDVAMGHVECVLADCFDFTAKDADIGPAGLRTPDPIFGDGTVLNEAVLGKIDLANTVAD
FRYLMLVDGGRQLGVEWLTL-NMLYDRIWVPPAQAALKNLTPICMFLSHPELRLSGTKSKFL-
ISKARAEQLMAGALIEEILEGVARFADPEATTHQSSKEKHFKYYGEQVDPVHLAYILDMFALVATQVTV
SDRLLKDRYVLNLRVFRGFTSLLWMAAQLAEKGPSATVITVGTVAGLGALVLFIILL-
RDAVLVETLFFRYVY--MRGYQAAKDVADADPEHFL-
LASASRDVEWIEIVPAFFYYFATATSRLTKNTAVAREFFGLFKERLRVIDVLAVPPDRVEALVEFVALAGT
LTTSAEA-
PGVIAFGLCEPNAAGVLEAERDWNLHRTMGITLAFFKAQVSVKGWFDKGLHKEVAQTGLREAGGFS
SPFHAGKEAKRCVPVVHRVIDLGELEAGLIEIDSAVVVADEEQTLADQPTTRDNSVNAIGGVVLAGTM
NMGTSLDSVERAGNIIIELEEQQYSAVNGKKADGKMTNAILMVLSRAYFPVIARTASANALVKGVFNL
LIQAPSDADNLDHGREMVIPAPTVHAETTPLYAESDIDKIAWIAPIHMLGTLAAVLVFEPDHYSFISMM
IDASLDITETAEVLDDYHVLEKGARPSAFRKILIRGLHIRLLAAVGTIIVVGSAGRDEVTAYIQGQAVPIT
MRKATFLPAFAASSCKTIDIFSTMLRPPERSSIKMLILETVTEL-
LEVTRGENDKLERILILLPGLDLAVIMGLVAAAKDFAQIVTVDAAITAILSVAAEAVVLREMLLTKAEIAV
TALALAMALLLMLTADKGLTQKEHAREKQMKELIQGGVVLQIRASDAIVLAATADKVRLSTDEGTTSA
FASLNLATVSDGFYVMSSAVLQLSSDLMMIVEVSRVLADKETKNALVVRHPEQVKTQPTYAEGSPGM
VLQTLTECIPPLAIKPEEVELLWTLVAALWRVKAHLANAARPRRERVLVLILALGLIVVLLQSEGGGMGG
GAVGAAAKVLAAFSILTIKATVEDEVRKVTRRVMDLNSRLGRPEEAVTPEFRGEELEQNKYEAENVEVD
CMETVRRFVDIPFLVSTRTQHNESEDRGSRMVLDVTRVRFIDL-
VTKRSEIILVERAETNGIIARQMAKDLQALHEEAAALGKVASLQHFISLNDGHISNYVTAQVNSVLGML
LLVLFIQQVRVSKVVWPTRREVTMVFILAALAVFFIVIGLYINLVFMVGGISPLQITTLVLGEGVMAVRQ

VSTFPAES-VTILQNTYADLICAGRVTKADIEKSLHIVVGARVARGTWYVIYHYEI-
QAQGPAGIMQRAFRQATLSKSLIDGRMEVKVKVVTSDRYA-
THEIKGGQHRKGTRYNRVKVGHNIPVDLLARIKMAFAPPNLVLVKTFGAVVQKRFAQQLTHMRHGA
VTIGPMEVSTPYIKAKKARHAPGDSVVNIKPDNIVRSVAVVPELIWARENEDQSAVHDKYTKVTHKIA
AKRNRAVDKAQYDKRNQVHLSFIGLAVKVLPEQAIVHATAGTLMATTSEAVTENLKGVAATIRLDPM
EKALIAYIKRMRPQLNLAGKTDKKIVKCLNNNLDDVEYGKLGRRKIKPITIKSVYDVEHERATELVKLMP
YKDDVEGRKSQFAEK--ALFEPL---
AIKIVGVSIRLRHKSTNVAETKHHIKTIEARARMTLAGSPSVKLHLSEGRVGTKGGRIKGYMPYKNYNIF
DKLSSGRRAKVKELAKLKKVDKSISVDGIRTRQQGGRIPKADLSNALANTRVGVDIVGENEQAASNPR
ANAEMGQIFAAIDARVYHHVGALIGHALAAVLAICAIQGIGKAHALLAVKLEINAGIEFHSAA-
VAVAVAAA-YAQQAGEA-ILADVSEPNLKALAVTEASVDVIR--RRKLG-
LKKTKKEAYMSLTCFAGKGDRVKIVEASQTQAFEIVATRTAERHGIKAKGTYGKERAMMQIVPTKPVK
VWVPVFVAEDELLAPIRAFAETNGDGVSVVKLAKDIKVTMFEKGVASLAVTGKATTVVYTVLEKVTQL
MKDIIYEQRSKAHYGRGRLRSHVVTHTKSKRHLSMMRRTVVADPRMDAARLTDGEVSVITGATMAF
VMSEQDAV--
GDVDMHFHEEPQREAQLGIAGPETFLATEQPRLLALLFLRAYLIHIVDIETQGGFADAATIYDILRAMQ
AKLDRKLSADIAKTRMCLHDDLAEMGVTQMAARRESAGDTILSAFWINGVTANADIILLVANLECKT
VPENIDCAIRLFYQRVTLQARERGGDVSVELVKAQNQIEAAEGTVIYTIKGKTPSTTQMALVVESISRSI
VKSLAVQLFQVEHFLNDTWIATLLRAAAEVVYVDARGGDEAVDHVVERDTELEFAPQKDIVARKIRIY
TPAAPSAVVIWDPAIEREEKADAQGTILMIYGPDLQDISCFTDIGAEAKIESLKSWDEKEKYVVHRDDIP
EDISTLVGKSSKVNKEFGIPDVAVCAPAKPGVTESIDRGEPKAGSCNEVANLPYPQESLVNRVSSANV
QKNISMVLGGNPVGEMGISREIERHLETNMCEIFVDKDDVDVALDFYRIALTALRVGLDPPNAKVEID
GKESRKLIAVLDGDADLQRHPKLSEESGALRKADTKARLPDDTLIYDGVQHGDTAFTATDLPEGRV-
VTPVNNTVKAQLKIDKVMVRTFHVVTLSFSSSKGEDHLIKLRLNALKKSTRHYEMLLKIRDTECATSPH
AISVGKILKVDSLLQTICVAHYTEIEPLMADDLTALGVMGDQGLLHVVPRLMILAVLHLYGLEAAMQV
PMWSLQEKAIVTDLTMGLPARDRAWSF-CISEVVGNTFFARTKDDDTEQRLHARNPI-VLL-
YGKTGMV-
ISPSGLRLDSTAVADMDRNNKDQLILSKERLQDAMGYYVLKILLVFVDLVILAYGSGDTPMRFLVADQ
AVTGRNNPQLVCLTRRTFPLDLQRDATFVMVFADATMKNLSHKKMHMNLVLIKSKGWVVNVSTKIL
ISDQAIASYAEDAGAHTQPSESALAAITSRVMAPIVLVVSQKPTAPSKTLFLEALNLLDEVTATVLNKPK
RYFVFEGDVVSVLATVDAAVLKGMQRPQPDRMRRAVVARAIGLAIAIQASGIIASLEADVINFSRAISL
AAGVFDDMDLVPQAATQ-PALAFRVRNHKVSTKRARPYKGHTKFLHKTDTDKALIAEGVDTIA
>GCA_000152725
AVIKPVSLDKYAPVLMIRQQEVATVSGAGSPSLRAKTVYRKSGLQRKISTKWVAKPLYY-
GVLKMGDIVQLVSISVDARSVCTVAQVVEQEVVVHGLVALEAVKRKIALDEISGMTTEDPAKVAEFEE
EVDLKKWRDADQRADLLPIMLGLTLKIRFDPHEPVVSDLPVPVIFEVL-
GIMAERGGIEVDECAISNAVSDVDPVAYVSPIDLDVVAGAQMDYIDQEAAVAAAKEAIALEELALEST
VLVVGI-PLLIPV-FGEEGGN-MSFANRSRALGTIVDLAVSFEEITRARI-EGFASSL-
MRDLQAAALSEKRGTAEEIRIVKRMQKAAGLADDPDLVILEDAAEGNAQWAGQIYDDPISGSSESDS
AGNIVHAANKRQMTTRSITFLRKVEISDKAAAPLMRGVKHNAKSRSARKLDDAIAALNFPNVPEGIAI
RAEAGRPADTLLHDATEGEEILMLIIRLKVTWKTAIIVGLFA-
AGDIVGLITLGGIAAVGKPDGLLNRHHKMNASLIEQRPIVEKLTLKLRASVQQKLGAENIIIFVVRVKGD
LVADAKVQM-GGLVGAVVAVQGDLIYVRRHRQTLVTDQESTV-
VLRGVAEGPADAEALLRIRVSHTLVEMTVLLIPG-

PVLVDSVSIREGVGKSLQIIQLDVYLLRDMGTGEETLAESRSRETIREILMIDPITGVRLAFGTVKERMGK
KLGLKDLDTLF--
IAILRHVTEPRTENLPAGVHRTFVEAGPRAAVGQLFLVLDDGGRLMPIDGELRELPKSVKALIGLLDSKA
PKVLSAGYATARLRIRYADCEVLESEADLYAYKEERRELLRMVPVYIITDAKRRQLIIFALSLRGGHRRAM
ASIGVMLAAAILESQAEKGSVHHAEPRILLTDAEIVGSSMMISRQASLEIKEFTKIDVVVVFPDFQEKMV
VSLRFFCTEVAIFVIALIVTVGEMLSREPEHAQEGGAGFARRIRGHSMLFALAAVFDRSNLDTSRLPGLF
LDLGLAPPKYGILYTNQPKKKQMPNEPEGEKVDGFELVHAIKEDQVSNHELEMPEAMALALE-
DATINLSGGVLALKGDMAAIERIDSKRKLSLAVEQWNISKLDAADVLMSGAAPVRCAG-
IESASISAALQSKDL--
LYLLGASNAELEESRLDGAGNSTVAVTEERQPLILLIYPVLVVIQDSNAAGLISNGGLEEWGTMAYKKH
YAFAARMRLHMRTIEAVELGVALEPNKQKSEKVKTVKLEGDTRFRQLAVEGVKEQMP-
LHALNPDFAV-VPVLVTPAVGLVAADESVLGARVPCAFSL-
RELQKSQKGSALPISAVRILPMRQARAMIDEDIMVAGKIAA-
LFKITALETILALVLRNDSEAALPWRLSVAERLEADTRRAGGYAARPFMSKPSLCDTQAHANVAEPIETI
AAGYVAAEERFVFPVEGIPIQIVLASPHSRVGTAKRAGEGHLHEMAKELLAKAVLMVKHEPDNTTEGL
LTYLTTNQPTDLLFEECLDIIIDLRRMSVV--
VHLNTV-
HVVVEGVRAVGQYEIMSAAADSGLVQVERVDERTGRERTRMWMYGAMEELWTMSVTFADWVPD
TPIGAIILHVYAQEINRAFSLHRLQLDGWPPEATRWWNTWQLWLNKDGFFGYAAAFRALCQILKDAL
HDYEMSDVEGVGRYQSRQELRVGYRIRDCVEYSQLDPRTDILLQAQTRLSVIRHALTVVAILVLRDVV
GEFSLVVAMEDVAMGHVECVLADSFSFTAKNADIGPVGLRTPETILGDGTVFREAVLGKVDLANTVA
EFRYLVLVDGGRALGVRWVEMRNMLYARIWLPPAQAALKNLLPICFFLGHNQLNLEIFDSLFAGISKA
RADQLMASVLVEEILEAVAAFAQPEATTHQSNKERHFKYYGEEVDPVHLAYILPIFALVATQVVVSDRL
LKDRYVLNLRV-RGLTSLLWLALQLSDKGPAATVISVGTIAGLGALVIFIILL-RDSVLIETLYYRYVY--
MRGYSAAKDVADADPEHLLALAAASRDVEWIEIIPAFFYYFDTAKSRYGVNTAVAREFFGIFEDRLRVIE
VLVVPPDRVLELVEFVAVAGTLPTSATALPGTIASGLCEPNAAAVLEADQDWNLHRSMGITLAFYKAQ
ESVKGWFDKGLHKGVAEISLEEAGGVTVHNRCEVDARQCIPALHRVLDMGELEGALIEIDSAVTVSEE
EQSLADKPTTREKTVGAVGGVVLAGTMNMGSNLDSVERNGNIIIELEEEQYSAVNGKKADGKLTDKV
LMVISLAYFPVLRAQASASAMDRGVFDLLIEAPDDATNSDHGKTLSVPAATIHGETAPLYAEAEIDRIA
WIAPLHLLGTLPAVL-
FEPDHYSFISMMIDASLEITETAEVLDDYHVLEKGARPPAFRTILIRGLQIKLLAAIGAIIVVGSAGRDEVT
AYIQGRAVPITLRKNTFLPAFAPSSCKAVDIFSTMLRPPERSTVQMLILETVAEL-
LEVTRIENDKLERLLVLLPGLDLAELAALAAAAQDFAQIVTIDRAVTAILSVAAEAVVLRELLLTKAEIAIT
ALAMGLALLLMLTADGGLTQKERAREAQLRDLIQGGAVLQVHASDDMVLAATADKVRLNTDEGTT
SAFASLNLATVSDGFYVMSSAVLQLSADLMMIVTVARVLADKEEKNALVVRHPEQVKTQPTYAEGSP
GMVLQTLTGCIPPLAIKPEEVELLWTLVAALWRTKAHLAEAMRPRRERVLILFLALALIVVLLQSEGGG
MGGG-
MGAAAKILAAFSILTIKASVGDEVRKVTRRVMDLNSRLGRPEEQVNPEFRGEELEQTKYEAENVEVDCI
ETIRRFVDIPFLVSTRTQHNESEDRGARMVLDVTRVRFIDL-
VTKRSEIILAARAETDGIIAREMAAEMQSLHPEAAALGKIAALEHFIALQDAHISNYVTADIHSVLGMLL
LVLFINQTRISKITWPTRREVTMVFIMAALAVFFLVIGLGLNIVFMLGGISPAQITTLILGEGVMAVRQVS
TFPAES-
VTILQNTYADLICAGRVTKADIEKSLHIVVGARVARGTWYVIYHYEISQAQGEAGIVQRAFRQATLSQS
LIDGRMEVKVKVVTSDRYA-

TYEIKGGQHRKGTKYNRVKVGHNIPVEVLPRLKMAFGDPNLILVKTFGAVVQKRFAQPLTHMRHGAV
TIGPMEVASPYIQQKKARHAPGDSVVDVKPDNIVRSVAVVPELIWARENEDNSAVHDKYVKTTHKLA
AKRTTAVDKAQYDKRNQVHLSFIGLAVKVLPDKAIVHATAGTLLPTEAVAVTEGLKGVAATIRLDPME
KALIAYIKRMRPQLNLQGKTDKKIVKCLNNNLDDVEYGKLGRRRIKPLTIKSIYDVEPKESTELVKLMPYK
DDVEGRKSQFAEK--
ALFEPLKAFMMMFGFIARAVYALYLRVVRRTILLVKTKIVVNASTILSAIKIIGVAIRLRHKTTNVADTKHH
IKTIEARARMTLAGAPSVKLHLRPGRVGKKGGRIKGYMPYKNFNIFEGLSSGRRAKVKDLAKLKKVDKEI
TVDGVRTRQ-
GGRLPKADISNAFANTRVGVDIVGENEQEESNPRANAEMGQIFAAIDARVYHLVAALIGHALEAILAL
CRILGIGRAHVLLAVKLRINAGIEFHSAA-VETAVAAA-
YAQPAGEAAAIADISEAYLGDLMLTAASIDVCRVRRRQIA-
LKKTKKEAYLKLTCFSGKGEKVKILECTQTQAYEIVATRTAERFGIKAKGTYGKERAMMQIVPTKPVKV
WVPVFVNEDELLAPVRAFAETNGDGVDVVKLTKDIKITMFEKAVASLAVAGKSTTVIYTVLEKVTELMK
DIIYEQRAKAHYGRGRLRSHVVTHTKSRRHLSMMRRSVVADPHMEAARLTDGEVSVITGATMAFVM
SEQDAV--
GDVRWHFHDEPRREAELGIAGPETFLATEQPRLMALLFLREYLIHIVEIESVGGFADAATIYDILRAMQN
KLDRKLAADIAKVRMCLHDNVADMGVKAEAPRRDAAGDAILSAYWINGVPANADIILLVANLDCDT
VPENIHCAVRLFYQRVTLQAKERDGDVSVKLVPAQNQIEAAEGTVIYTIKGKEPGTVQMKLNVEGISR
SIVKSLAVQLFQVDLFTNDTWIATLLRAAQDVVYVDASEGREAVDHVVERDTELEMAPQKDIVRRKIR
IYTPAAPSAVVIWDPAIERENKADAQGTILKIYGPDLQDIACFTDIGAEPKIESLKSWDEKEKYVVHRDDI
PEDI-
TLVGKSSKVNKEFGIPDVQVCAPAKPGVTESIDRGEPKAGSCGEVANLPYPQESLVNRVSSANVQKNI
SMVLGGNPVGEMGISRGVERHLETNMCEIFVDRPEVDVAKDFYIIRLTPLRVGLDRPNAKVEVGGKES
RKLMAVLDGAAALQRHPKVSEAGGTR-KAGAKAKLEEDALIVDGVMHGDIKFTCTDLPEGRV-
VTSVFNAVKAQLDIDKVLLKTFHVVTLSFSNDKGDEHLIKLRLNALKKSTRHYEMLLKIRDTECAASPHA
IPVGKILKIDALLQTICKAHYTEIEPLLADDLTALGVMGDQGLLHIVPRLMILAVLHLWGLDAACIVPHW
GLSRRAVITDQTMGLSVRERRWTF-AISEVVGNTFFAKTADDETEQRLHARNPI-VLL-YGKMGMV-
ISPSGLRLDSTPPSSLGHGNEQQLILSTERLQDAMAYFVLRLLLVFVDHVILAYGSGEEPLRFLVADQAV
TGRTNPGLVCLTRRTFPLDLQRHATFVLVFEDCTMKNLSHKKMHMNLVLIKSKGWVVNVSTKILISDQ
AIASYAEDAGAHTQPSESALAVITSRVIAPIVLVISQKPTAPSKTMFLEALNLYDEITTTVLNAPKKYYIFE
GDVISVLGRIDAAVILGTQRPQPDRMRRAAVAAAVRLAIALRASGIVAALDAGHINFSRAVSFAH--
Y----------------EFRVRNHKTSTKRDRPYKGHPKFLHKEDEEKNLIADGVDTIA
>GCA_000152785
AVIRPVTMKTYAPVLMIAQSQVATVSGAGSPSLRAKTVYAKSGLQRKAETKWVVKDFYY-
GVVKLGNLVQLVSISVDNRAVCTVAQLVEQEVVVHGMVALDAVKKKIALEEITGMTAEDPAKVIDYA
GELELRKFRDADDRADMLPILLGLTLKIRFDPHQPLVHDLPIPVIFEVV-
GIMVQRGTIEVDQCAISTAISDVDPIVYVSPIDLATVSGDKMAYIDQEGAVSAAKADIAL--
LALESAVLVVGI-PLLIPV-FAAHADV-MSFANRSRTLKTIVNLAVSYPTIKRA-IDG-IAAIL-
MEASQAAALAEKRGTAEQIRIVKRMQRSAGLADDPDLVLLEDAAEGNSNWAGQIYDDPLSGSSESD
SAGNIVFAANKRQNFQRSITFLRKVEITDQAAAPLMRGVKHNAKSRSARKVDDAIAALNFPNVPEGIA
IRAAAGSQIHLLLHDATEGEEILMIIVRWHVTWKTAIIISLAVTAGDIVGLIALGGIGAVGRKDGLLNRHH
KMNGALIEQRRIVEKLTLRLRAALKQKIGAEHIIIFVVKVAGDLVAKAKIQM---
SVGAVVAIQGETIYVRRHRQTVIADEDATVEVLRGVAEGPADAQALLRIRVDHVVVKLSVLLIPE-
PVLVESVSIREGVGKALQIIQLDVYLLRDMGTGEETLAKSRSREMIREILQVDPITGVRLAFGILRERLVKG

GSMKELGTLL--
IAIIDKITEPRMANLPDGVCRTFINAGPRAAVGMIFLLLDDAGKIYTIDGDLRELTKSIKSLIGLVDAAKPKI
MSAAYAAVRLQVRYADVTVLVSDADLYAYAEERRELLRMVPVYITNAAARRQLIIFALSLRGGHRRAR
AAIGVLIAAAILESQALQGPVNHAHPAVLLDDGEIVGSEMMISRQAALDLKQIIKTDVIVIFPEFQEKMV
VSLKFFCTDVAIFVIALICTVGAMLSREPSSAQEDGAGFQKRIRAHSVLFALVATFDGRNLRSA---
DLFLALGLAPPRYGVLYTEQPKRKQMPSEPDKDEVDGFDLVHAIKDDTASNHQLDMPEAMALAVE-
DATINVTAGVLAMTGDMAAMEKVDSKRKMALAVTKWTISAHAIVDVLISGAHPFRVLG-
IEAADVSVGLPHKTIWFLFLPGANNAELEEDKLDGAGASTAEAAKDKQTGILLVNPVLVVIQDSNTAA
LIANGGLEEWGTMAYKKHYALAARMRLNMRTID------
QMEPNKQKAEKIKTVKLEGDTRFRQLATEGVKEQMP-LHALNPDFAV-
LPVLVTPAVGRVAADEQVLGAEVPCDASL-
RELQKSMSGDALPISGIRLLGMRAARAMVDEDIMVAGKVAA-
LLVILALETITHLVLRNDSEATLPWRLSVAERLEADMKRASGYAARPFQSKPSLCNTQAHAKIADPIQEI
AAGYVAKPDRFVFPVEGIPIQLILASPHSRVGAAKRAGLGHLHEMAKELHAKAVIMVLNEPDTNTDGL
LNYLTTQQPTDLLFEDCLDVVIDMRRMSVV--
VQLNTV-
HVLIPGVRDLGDREIMAAAAPSGLVQMERVDDRTGRERTLMWMYGAMEELWLLSVTYADWVVDC
AIGAILLHVYAQVINRAFSLDRLQLAGWPREANRWWNTWQLWLNKDGFFSYDAAFRTVLAVLSSDL
DDYDMSDVEGVGRHLSRQQLRVGYRIREQVAFEQLDPRTDILLQSRTRLSVIRHALAMVAILIVRDIVV
EFTLVVAKEDVAMGHVECVLADSFDFTAKDADIGPVGIRTPDTVLGDGTVFNEAVLGKIDLANTVADF
RYKMLADGGRDLGVSWITLQNMLYARIWLPPAQAALKNLVPIAFFLGHTELNLLLAPALWSGISKARA
QKLMAGALIEKILAKVAAFADPEATTHQSKKAPHFKYYGEEVDPVYLAYILQIFALVATQVTVSDRLLT
DHYVLNLKI-RGFTSLLWLSAQIADKGPAATVITVDTIMGLGALVLFIILL-RASVLVETLYYRYVY--
MLGYQAAKDVADADPEHFLSLAHASRDVEYIEIIPAFFYYFKTAT-
FEPFNTALAREFFGLFEERLRIIEVLVVPPDRVLELVQFVALAGTLATSAEA-
PGVIACGFCEPNAAGVLEADQDWNLHRSMGITLAFYDARESVKGWFDKGLHKDVAATGLTEAGALT
TPFHAGATAKQCVPAVHRVLDLGEVEAALIEIDSAVLVADEEQTLADKPTTRENSVGAVGGVVLAGT
MAMGANLDSVERAGNIIIELEEENYQAVNGKKSDGKITDKVLMVLSEAYFPVLRRMASANALDKGRF
GLLIDAPDDADNQDHGRSLTIPAPTVHSESAPLFAATEIERIAWIAPIHLLGTLTAVLGFEPDQYSFISM
MIDASLNIMQTAEVL-DYHVLEKGSRPPSFRVILIKGLHIRLLAGICTIIVVGSAGRAEVTAYIQG-
AVPITLNKKTFLPAFAASSCHAIDIFSTMLRPPERSTIMTLILETVTEL-
LEVTRLENDKLERMETLLPGLDVAELLGLVAAAGDFTAIVTIDRAITAILSVAAEAVVLREMLLTLAEIAV
TALALALALLLMLTKDQGLHQKDHAREAQMKDLIQGGVVLQVKAAERMMLASTADKVRLDTDDGT
TSAFASLNLATVSDGFYVLSSSVLQMSSDLMMIIKVARVLADKETKNALVVRHPEQVKTQSTYAEGSP
GMVLQTLTGAIPPLAIKPEAVELLWTLVAALWKVKAHLANAARPHQERVLILILALSLITVLLQSEGGG
MGGG--
NPPAKMLAAFSILTIKAKAGDEVRKVTRRVMDLNSRLGRPEESVNPEFRGAELEQSKYEAENVEVDTM
ETIRRFVDIPFLVSIRTQHNESEDRGARMVLDVTRTRFIDL-
VTKKSEIILAARAETDGIIAREMGKDLAALHPQAAALGKIAALQHFIAIQDAHISNYVTADIHAVLGMLL
LVLFIQEVRVAKVVWPTRREVTMVFILAALAVFFLVIGLGVQIVFMLSGISWNALTTIVLGEGIMVVRQT
STFPEDS-
IGILQNTFAELICSGRVAKADIENSLHIVVAGRVARGTWYVIYLYEISQAQGQSGIMQRAFRQMTLSQS
LIDGRMEVKIKVVTSDRYA-
TYEIKGGQHRKGTRYNRVKVGHNIPVQVLARMKMAFTPPNLVLVKTFGAVVQKRFAQALTHIRHGA

VSIGPMEVSSPYIQNKKARHAPGDAVVDLKPDNIVRSVAVVPELIWARENEDQGAVHDKYVKTTHKL
AAKRKKAVDKAQYDHRNQVELSFIGLAVKVLPEQAIVHATSGQLMPTASVAVTEGMKGIAATIRLDP
MEKALIAYIKRMRPQLNLAGKQDKKIVKCLNNNLDDVEYGKLGRRKIKPLTIKSVYDVESRRATELVKL
MPYKDDVESRKSQFAEK--
ALFEPLKAFMMMFGFIARAVYALYLRVIRRTILLAKTKVVVNADSVLSAIKIIGVSIRLRHKSTNVAETNH
HIKTIEKRARMTLAGAGKVKLNLSPGKIGMKGGRIKGYMPYKNFNIFDGLASGRRAKVQDLAKLKDVD
KTIAVDGIRQKQQGGRTPKAQLSNAI-
NTRVGVDIVGENEQEESNPRANAEMGQIFAAIDARVFSHVGALIGHALEAVLAVVAIWGIGRAHSLL
AVRLHINAGIEMHSAA-VPRAVAAA-
FAQNAGEASLIADVSEGDLGALAVTAMSVDVCRLRRRSLAELKKTKKDAYMGLTCFSGKGDKVKIVEA
TQTQAYEIVATRTAERHGIRAKGTYGKERAMMQILPTKPVKIWVPVFVSEEELLAPIRAFAETQGDGVT
VVKLTKDIKVTMFEKAVASLAVSGKATTVVYTVLEKVTELMKDIIYTQRAKAHYGRGRLRSHVVTTTKQ
RRHLSMIKRTVVADPSMEAARLTDGEVSVITGATMAFVMSEQEAV--
GDVDLHYHEEPRREVELGIGGPETFLTTEQPRLLAVLFLREYLLHIVEIGTTNGFSDAATIYDVLRAMVYK
LDRKLSADIAKTRMVLVDDLMEMGVTEVAARREAAGDKILSSYWINGVTANADIVLLVANMECPCVP
ENITCAIRLFYQRITLQAKERGGDVSVAVVAANKQIEAAEDTVIYTIKGKTPGTAQMKLTIEGISRSVVKS
FAVQLFQIDTFMDDTWIATWLRAADDVVFVAAEDGKEAVDHVVERDTAVEFAPQKDIVSRKIRIYTP
AAPSAVVIWDPAIDREDKAEAQGTILKIYGPNLQDINCFTDIGAEPKIESLKAWDEKEKYVVHRDDIPD
DIDTLVGKSSKVNKEFAIPDVAVCAPSKPGVTESIDRGEPKAGTCGEVANMVYPQESLVNAVSSANV
QKNISMVLAGHPVGEMGISREIERHLETNMCEIFVDKADVDVALDFYTLVLTILRVGLDPPNARVEVD
GKESRKLMAIQDGDAALQRHPKVSMATGTGLGAGVKARMPDDALILDGVVHGALSFTATDLPAGR
V-
VTPVFNTVKAQLKIDKVLLKSFHVVTLSFSNNKGEEHLVKLRLNALKKNTRHYEMLLKIRETECAESPHA
ISVGKVLKIDKLLQTICTAHYKEIEPLLADDLTALGVMGRQGLLHVVPRLMILAVLHLI-
IECGMILPKWRLQQRAVITDQTMGLAVRARAWSY-AISEVVGNTFFARTKEDDTEQRIHARNPV-
VLL-LGKFTMI-
ISPSGLGLQSTPATDQDLGNTEPLILSPERLQDEMGYYVIAILLVFVDQVILAYGSGDEPIKFLVADQAV
TGKQNPGMVTLTRRTFPLELQRDATFVMVFEDSTMKNLSHKKMHMNLVLIKSRGWLVNVSTKILISD
QAIASYAEDAGAHTQPSESALAAITSRVMAPIVLVVSQKPIAPSKSLFLEALALYDAVTATVLNKPKRYF
IFEGDVVAVLGHLDAAVLLGMQRPQPDAMRRAIVAAAIRLAIAALASGIITRLEQGLINFSRAVSFTQ-
AY--
>GCA_000152845
AVIKPVVMKEYAPVLMIKQTQVATVSGAGSPSLRAKTVYAKSGLQRKTSTKRVAMNIYY-
GVMKLGDIVQLVSISVDARAVCTVAQVVEQEVVVHGLVALAAVKKKIALEAINGMTTEDPAKVSEFD
EELDLRKFRDAEDRSDMLPVLLGLTLTIRFDPHEPLVHDIPIPVIYEVV-
GIMADKGGIEVDDCAISTAVSDVDPLAYVSPIDLAAVAGEQMAFIDQEHAISAAREAIALEELALESSV
LVVGIRPLLLPV-LGEDGDN-MSFANRSRELGSIVDLAVSAERLSRAR-EQGIASEM-
MVRMQAEALAERRGTAEEIRIVKRMSRSAGLADDPELVLLGDAAEGNSSWAGQIYDDPISGSSQSDS
AGNIVVAANKRQNFARSITFIRKVEISDQATAPLMRGVKHNSKSRSARKVDDAIAALNFPNVPEGIAIR
AEGDKSADTLLHDATETEEILLLVVRWKVTWKTAIIIALLVEAGNVVGIIELGGVAAVGRPEGLLNRHHK
LNGSLIEQRPIIEKLTLRLRASLKQKLGAEQVIIFVVKVSGDLVADAKVQMG-
GPIGAVVAVQGDVIFVRRHRQTLVTDSEATV-ILKGVAEGPADAEALLRIRVSHTLVALSILLIPQ-
PVLVDSVSIREGVGKALQIIQLDVYLLRDMGTGSETLAESSSRELIREVLMIDPVTGVRLAYGEIKERMGK
KVGLKDLETLF--

ISILEGITEPRMENLPAGVCRNFIQAGPRAAVGMIFLLLDEAGRIYTIDGDLRELPKSVKSLIGLVDSTAAK
VLSATYATDRLRIRYADCEVLVAEANLYAYKDERKEMLRMVPVYIINEAKRRQLVILALSLRGGHRRAM
ASIGVMIAAAILESQAEMGVVDHEEPDILLDDAEIVGTAMMISRQAALDLKQIVRSDVVVVFPDFQEK
MVVSLRFFCTEVAIFVIALITTVGDMLSREPNSAKETGAGFQKRIRKHSVLFALEVSFERRNCRDA-
RPDLFLGLGLATPRYGIFYTNQPKKKQMPSEPDSDRVDGFELVHAIKADTGSNHELDMPEAMALAVE
ADAAINLAAGVLAFKG-MTAIERVDSKRKMALATEKWNISKLDMADIHLSGAAQMRATG-
IEQATLSAALATKIL--
LYLPGASNSALEEDKLNGAGASSAEAAEERQTRILLVTPVLVVIQDSSAAGLITNGGLEEWGTMAYKK
HYAFAARMRLHMRTIDCIEVGVEMEPSKQKAEKVKTVKLQGDTRFRQLAIDGIKEQMP-
LHALNPDFAVLVPVLVTPAVGRVAADEAVLGARVPCEFSL-
RELQKSMRGSALPITAIRLLPMRAARAMIEEDIMVAGKVAA-
LLKIRAHETVMGIILRNDSEATAPWRLSVAERLEADAKRMAGYAARPFISKPSLCDVQAHANVAEAIET
IAAGYVAKDQRFVYPVEGIKIRLILSSPHSRVGEAKRASEAHLHEMAKELIAKAVMMLKSEPDTNTEGLL
NYVTTNQPTDLLFPDCLDVVIDMRRMSIV--
VQLTVV-
HVVVDGVRDVGDYEIMAAAAPSGLVQVERVDDRTGRERTIMWMYGAMEDLWLMSVRYADWVA
DCTIGVIILHVYAQTINRAFSLTRLQLEGWPPEATRWWNTWQLWLNKDGFFGYEAAFRAVRQALKA
ALFGYEMSDIEGVGRYQSRQELRVGYRIREQVDFARLDPRTDILLQAKTRLAVIRHALALVAILILRDVV
GEFTLVVAHEDVAMGHVECVLAECFDFTEKNAHIGPVGLRTPETVLGDGTVLQEAVLGKIDLANTVV
DYRYLFVVDGGRELGVWWLSIENMLYPRIWLPPAQAALKNLLPICFFLSHPELDLWRV-
GLWEGISKARAQQLMAGALIEEILDAVARFADPEATTHQSNKERHFKYYGEEVDPVHLAYILDLFALV
ATQVVVSDRLLKDRYVLSMRVFRGFTSLLWVALQLAEKGPAATVITLGTIAGLGALVLFIILL-
REHVLVETLFYRYVY-VMLGYSAAKDVADADPEHLLVLAAASRDVEFIEIVPAFFYYFVTAK-
FEPFNTAVAKEFFGLFEERLRVIDVLAVPPDRVEALVEFVALAGTLPTSAEALPGIIACGLCEPNAAGVLE
ADQDWNLHRTMGITLAFFEARESVKNWFDKGLHKEVAETGLREAGGFTTTNLCGKEAKKCVPAIHRV
VDLGELETGLIEIDSAVMVADEEQTLADRPTTREKKVGAVGGVVLAGTMNMGSALDQVERGGNIIIEL
EQEQYHAVNGKKADGKLTDKVLMVLSCAYFPIIRKMASATALDKGVFDLLITAPEEADNLDHGGVLTI
PIPTVHAE-
AILYAESEIDRIAWIAPMHLLGTLPAVLLFEPDQYSFISMMIDASLEITQTAEVLDDYHVLEKGARPPSFR
VILIRGLHIRLLAAIGTIIVVGSAGRSEITAYIQGKAVPISLSKATFLPAFAASSCKLVDIFSTMLRPPERSTV
QMLILETVAEL-
LDVTRLANDKLERLLLLLPGLDAAQLLALLAAATDFSDIVTISKAITAILSVAAERVVLRELLLELAEIAISSL
ALALALLLMLTPDRGLTQKERTREAQKKELIQGGAVLQVHAADAMVLASTADKVRLNTDEGTTSAFA
SLNLATVSDGFYVMSSAVLQLSSDLMMIVSVSRVLADKETKNALVVRHPEQVKTQPTYAEGSPGMVL
QTLTTCIPPLAIKPEDVELLWTLVAALWRVKAHLAEARRPRLERVLILLLALALIVVLLQSEGGGMGGG-
MGAAAKILASFSILTIKASVGDEVRKVARRVMDLSSRLGRPEESVNPEFRGEELEQTKFEAANVEVDCM
ETIRRFVDIPFLVSIRTQHNESEDRGARLVL--
PEIAALGKIAALDHFISVNDAHISNYVTADISSVLGMLLLVLFIQQVRVAKVVWPTRREVTMVFIMATIA
IFFLVIGLG-KLVFMLGGISPLQITTLVLGEGVMAVRQASTFPADS-
VTILQNTYADLICAGRVTKADIEASLHIVVGARVARGTWYVIYPYEITQAQGEAGIMQRAFRQATLSKS
LIDGRIEVKVKVVTSDRYA-
TYEIKGGQHRKGTRFTRVKMGHNIPVDLLARIKMAFGDPNLILVKTFGAVVQKRFAAPLTHMRHGAV
TIGPMEVASPYIKMKKARHAPGDSVVEIKPDNIVRSVAVVPELIWARENEDQSAVHDKYVKVTHKIAA
KRNAAVDKAQYDKRNQVHLSFIGLAVKVLPAQAIVDATAGTLLPTASVAVTENLKGVAATIRLDPME

KALIAYIKRMRPQLNLAGKTDNKIVKCLNNNLDDVEYGKLGRRALKPLTIKSVYDVESRRATELVKLMP
YKDDVESRKSQFAEK--
ALFEPLKAFMMLFGFIARAIYALYLRVVRRTILLVKTKIVVNAKTVLSAIKIIGVSIRLRHKSTNVAETNHHI
KTIEKRARMTLAGGPSVKLHLHPGRIGMKGGRIKGYMPYKNFNIFEGLASGRRNKVRELAKLKTVDKTI
TVEGIRTRQQGGRIPKADLSNAI-
NTRVGVDIVGENEQEESNPRANAEMGQIFAAIDARVYALVAALIGHALEAVLALAAIQGIGKAHALLA
VKLHVNAGIELHSAA-VEKAVAAA-YAQPAGEA-AIADVSEG-LGLTRVNSASLDVCRMRRRQLA-
LKKTKKDAYMELTCFAGKGDRVKIVDASQTQFYEIVATRTAERHGIKAKGTFGKERAMMQITPSKPVK
VWVPVFVSEEELLAPIRTFAETNGDGVTVVKLTKDVKVTMFEKAVASLAVTGKSTTVIYTVLEKVTELM
KDIIYEQRAKAHYGRGRLRSHVVTHTKQDRHLSMMRRTVVADPRMNAARLTDGEVSVITGATMAFV
MSEQDAV--
GDVDMHFHEEPRREAALGIAGPETFLATEQPRVLAVIFLREYLIHIIEIGTRGEFNNAATIYDILRAMQSK
LDRKLSAEIAKVRMCLHDDLADMGVRQEAPRRETAGDPILSAYWINGVTAQSDIILLVANLECKTAPE
NINCAVRLFYQRVTLQAKERGGDVTVALVKAQNQIEAAEGTVIYEIKGKTPGTIQWKLNIDAISRSIVKS
LAVQLFQVDLFLNDTWIATLLRAAEDVVYVDAKAGKEAVDHVVERDKALEMAPQKDIVRRKIRVYTP
AAPSAVVIWDAAIERADKADAQGTIIKVYGPDLQDISCFTDIGAEPKIESVKSWDEKEKYVVHRDDIPP
DI-TLVGKSSKVNKEFGIPDVAVCAPSK---
VESIDRGEPKAGSCGEVANLPYPQESLVNRVSSANVQKNISMVLGGHPVGEMGISRKVERHLETNM
CEIFVDKADVDVALDFYTLTLTPLRVGLDKPNAKVEIDGKESRKLMAILDGDAALQRHPKVSEASGTGT
-AGAKARLPDDALIFDGVLHGDISFTATDLPDGRV-
VTPVANTVKAQLQIDKVLLRTFHVVTLSFSSHKGEENLIKLRLNALKKSTRHYEMLLKIRDTECAQSPHA
IPVGKILKIDALLQTICVAHYVAIEPLLADDLTALGVMGDQGLLHIVPRLMILAVLHLWGLEAAEIVPHW
-LPRRALITDLTMGLRLRARDWTY-AISEVVGNTFFARTADDDTQQRLHARNPI-VLL-YGKFGMV-
ISPSGLRLDSTAPAPDEHGNADALILSRERLQDAMGYFVLRILLVFVDEIILAWGSGDEPLKFLVADQAV
TGRDNPGLVCLTRRTFPLDLQRDATFVMVFEDSTMKNLSHKKMHMNLILIKSKGWIVNVSTKILISDQ
AIASYAEDAGAHSQPSESALAVITSRVMAPIVLVLSQKPTAPSKTMFLEALNLLDEVTATVLNRPKRYYI
FEGDLISVLHTVQASVIIGMQRPQPDRMRRAVVATAIRLAIAALGSGVVVSLEAGVINFARAISLAR--
YPLPTLL----------DFRVRNHKTSTKRDKPYKAHPKVLHKADSNKGLVAEGVDTLA
>GCA_000152965
AVIKPVVMKAYAPVLMIRQTQVATVSGAGSPALRAKTVFRKSGLQRKTSSKWVAKPLYY-
GVVKMGNIVQLVSISVDKRAVCTVAQLVEQEVVVHGLVALDAVKRKIALEEMNGMTTEDPAKVAEFE
AELDLKRFRDADARSDILPVLLGLTLEVRFDPHEPVVHDLPLPVIYEVI-
GIMADKGGIEVDDCAVSSAVSDVDPIAYVSPIDLEAVAGTEMAFIDQEAAVAAARETIALEELALEAGI
LVVGI-PLLLPV-FGEEGDV-MSFAARSRTLKSIVALAVAYPALTRA-IE-AIASEM-
MEAAQAAALAEKRGTAEQIRIVKRMQRSSGLADDAELVILEDAAEGNSSWAGQIYDDPISGSSESDS
AGSIVHAANKRQNFARSITFLRKVEISNKAAAPLMRGVKHNSKSRTARKLDDDIAGLNFPNVPPGIAIR
AAANKPADRLLHDATEGEEILLVVVRWHVTWKTAVIVLLVLLAGDVVGLIRLGGIAAVGRPDGMLNR
HHKLNGSLIEQRPIIEKMTLRLRAALKEKLGKDQIIIFVVKVQGDLIADATIQMG-
GPVGAVVAIQGELIFVRRHKQTIITDQEATV-VLKGVAEGPADAQALLRIRVTHTLVELSVLLIPE-
PVLVDNVSIREGVGKALQIIQLDVYLLRDMGTGEETLAESKS-------------------------------
----
RSILAAVTEPRMENLPDGVCRTFITAGPRAAVGMLFLLLDEAGKLYAIDGDMRELTKSVKSLIGLVDSK
APKVLSAGYAEARLRIRFADCEVLETEADLYPYKEERRQLMRMVPVYIIAEAKRRQLILFALSLKGGHRR
AMMALNVMLAAAIIESQTEQGKVHHDEPQILLDDAEVVGTAIMITRQGALDLKQILKVDVIVLFPEFQ

EKLVVSLKFFCTEVAIFVIALITTVGEMLSREPEHAQEEGAGFQKRIRGHSVLFALEVTFDRANCRRNAL
PDVFLGLGLAEPRYGIVYTNQPKKKQMPSEPDGDRVDGFELVHAIKEDGRS-EELEMPEAMALAVE-
DATINVSAGVLAMTGGMVAVEKVDSKRKVALAATKWNISKLQLVDVSISGAAPVRSCG-
IEQADLSAALYAKKVWFLFLPAASNAELEEEKLDGAGASTAEAAEDKQIDILLVKPVMVVIQDSNSAGL
VANEGLDEWGTMAYKKHYAFAARMRLHMRTID-------
MNPNKQKSEKVKTVKLEGDTRFRQLAVDGVKEQMP-LHALNPEFAV-
LPILVTPAVGSVAADENVLGARVPVDASL-
RELQKSQKGSALPISAIRLLAMRAARGMIEEDIMVAGKVAA-
LFKVRALETIVKLVLRNDSEATLPWRLSVAERLEADTKRASGFAARPFKTKPSLCNTQAHSKIADPIQEIA
AGYVATEAKFVFPVEGIDIKLILSSPHSRVGEAKRAGEGHLHSMAKELLAKAVVMLRNEPDTNTEGILN
YLTTNQPTDLLFEDCLDVIIDIRRMSVV---
VHLNTV-
KVVVDGVRPVGDYEIMAAAAVSGLIQVEQVDDRTGREKTMMWMYGAMEDLWLMSVRWADWVP
NCAIGAIILHVYAQTINRAFSLSRLQLDGWPKEAARWWNTWQLWLNKDGFFGYQAAFRAVKQILKA
TQADFEMKDVEGVGRMQSRQEVRAGYRIREQVEFARLDPRADILLQAKTRLSIIRHALEVVAILILRDV
APEFT-
VVAYQDVARGHVECVLAESFDFTEKNTHIGPVGLRTPDSIFGDGTVLKEAVLGKIDLANTIADFRYLFIL
DGGRALGVVWLSL-NMLYARIWLPPAQADLKDLLPICFFLQHPELDM---------
ISKARAEQLMAGALVEEILEAVVRFADPEATTHQSKKEKHFKYYGEEVEPVHLAYILDLFALVSTQVTLS
DRLLKDRYVLNLRVFRGFTALLWLSLQLAAKGPVATVITVGTIAGLGALVLFIILL-
RDSVLVETLYYRYVYG-
MRGYSAAKDVQDADPEHLGELSAASRDIEWIEIIPAFFYYFKTAKSRRTLNTAVAREFFGLFKERLRIIEVL
AVPPDRVLDFVAFVALAGTLETSATALVGVIAVGLCEPNAAAVLEAERDWNLHRSMGITLAFFEARDS
VKGYFDKGLHKDVAEIGLREAGAFTTHFLCDVDAKRVVPAIHRVLDLGELEAGLIEIDSAVVVADEEQT
LADKPTTRDKSVGAVGGVVLAGTMNMGANLESVERGGNIIIELEEEQYHCVNGKKSDGKLTDKVLM
VLSVAYFPVIGRFASANALDRGVFGLLIEAPTDADNQDHGGQMVIPIPSILTETTPLYAESDIDKIAWIA
PMHLLGTLPAVLEFEPDFQGFISMMIDASLEIMDTAEVLDDYHVLEKGARPAAFRVVLIKGMHIRLLAA
VGTIIVVGSAGRSEVTAYIQGQAVPIILKKHTFLPAFAASSCKVVDIFSTLLRPPERSAVEMLILETVAEL-
LEVTRGENDKLERLLVLVPGLDLALLLTLLALADDFSDIVTISAAITAILSVAAEAVILREMLLTKAEIAITAL
ALALALLLMLTGDEGLTQKENIREEQMKELIQAGAVLQLHAGDAMVLASTADKVRLDTDEGTTSAFA
SLNLATVSDGFYVLSSAVLQMSSDLMMIVAVARVLADKESKNALVVRHPEQVKTQPTYAEGSPGMV
LQTLTECIPPLAVKPEDVELLWTLVAALWRVKAHLASAARPHKERVLILLLALGLIVVLLQSEGGGMGS
GAMGSAAKVLACFSILTLKASVRDEVRKVTRRVMDLSSRLGRPEEAVNPEFKGEELDQTKYEAENVEV
DCMETVRRFVDIPFLVSTRTQHNESEDRGARLVLDVTRVRFIDTVVTKRSDVILVERVETDGVIAREMA
KDMQALHETAAALGKVSALQHFIALGDAHISNYVTAQAHSVLGMLLLVLFIQQVRVSKVVWPTRREV
TMVFIMAALAVFFLVIGLSLNLVFMVGGISPLQITTLVLGEGVMAVRQTSTFPAES-
VTILQNTYADLICAGRVTKADIESSLHIVVGARVARGTWYVIYHYEITQAQAPAGIMQRAFRQATLSQ
SLINGRMEVKIKVVTSDRYA-
TYEIKGGQHRKGTRYNRVKVGHNIAVDLLPRIKMAFGPPNLVLVKTFGAVVQKRFAQPLTHMRHGA
VTIGPMEVSSPYITSKKARHAPGDSVVDIKPDNIVRSVAVVPELIWARENEDQSAVHEKYVKTTHKIAA
KRSRAVDKAQYDKRNQVHLSFIGLAVKVLPEQAIVAATAGTLLPTATTTVTEGLKGVAATIRLDPMEK
ALIAYIKRMRPQLNLQGKTDNKVVKCLNNNLDDVEWGKMGRRKIKPITIKSVYDVEHKQATELVKLM
PYKDDVEGRKSQFAEK--
ALFEPLKAFMMMFGFIARSVYALYLRVIRRTIILVKTKIVVNANNVLSAIKIIGVSIRLRHKTTNVAETKHHI

KTIEKRARMTLAGTGAVKLHLSPGRVGKKGGRIKGYMPYKNFNIFDGLASGRRAKVAELAKLKKVDKTI
AVDGIRTRQ-
GGRLPKAELSNALSKTRVDVEVVGENEQAESNPRANAEMGQIFAAIDARVYSLVAAMIGHALEAVLA
ICAILGIGKAHVMLAV-LKMNAGIELHSAA-VARAVAAA-YAQPGGEA-
AIADVSEGDLQALAISDQSLDVCRLRRRQLG-
LKKTKKEAYMSLTCFAGKGDRIKIIEATQTQAYEIVSTRTAERHGIKAKGTYGKERAMMQITPTKPVKV
WVPVFVNDDELLAPVRAVAETNGDGVTVVKLSNDIKITMFEKGVASLAISGKVTTLVYTVLEKVTELSK
DIIYEQRAKAHYGRGRLRSHVVTHTKQKRHLSMMKRTVVADPSMDAARLTDGEVSVVTGATMAFV
MSEQDAV--
GDVDLHYQEEPRREAPLGIAGPETFLSTEQPRVLALLFLRAYLMHIVDIGTVGGFSDAATIYDILRAMQ
NKLDRKLSAEIAKVRMCLHDDVEGMGVTAEAARRDTAGDSILSAYWINGVTANSDIILLVANMECKT
VPENINCAVRLFYQRVTLQAKERDGDVSVELVPAQNQIEAAEGTVIYTIKGKTPSTAQMKLNIEAISRSI
VKSLAVQLFQVNLFLNDTWIATLLRAAQNVVFVDAREGKEAVDHVVERDTALEMAPQRDIVARKIRI
YTPAAPSAVVIWDPAIEREDKADAQGTILMIYGPDLQDISCFTDIGAEPKIESLASWDEKEKYVVHRDDI
PEDITTLVGKSSKVNREFGIPDVQVCAPAKPGVTESIDRGEPKAGSCGEVANLPYPQESLVNNVSSAN
VQKNISMVLGGNPVGEMGIARDVERHLETNMCQIFVDKADVDVALDFYKIVLTPLRVGLDKPNAKVE
IEGKESRKLMAVKDGEAALQRHPKVSEESGGRVGGTEKAKLPSDALIFDGVQHGDLKFTATDLPEGRV
-
VTPVFNEVKAQLEIDKVMLKTFHVVTLSFSSEKGDEHLIKLRLNALKKSTRHYEMLLKIRDTECAQSPHA
IPVGKILKIISLLQTICVAHYTAIEPLMAEDLTVLGVMGDQGLLHVVPRLMILAVLHLCPLEAEDMLRTW
GLQQRAILTDQTMGLPLRERQWSY-AVSEVVGNTFFARTKEEDTEQRLHARNPI-MIL-YGKEGLL-
ISPSGLKLSSTNPSDADHGNAEQLILSTERLQDSMSYHILAILLVFVDEMILAYGSGDDPLKFLVANQA
VTGRANPQLVCLTRRTFPLDLQRDATFVLVLEDTTMKNLSHKKMHMNLVLVKPKSWVENVSTKILIS
DQAIASYAEDAGAHTQPGESALAIITSRVMAPIVLVVSQKPTAPSKTLFLEALNLYDEVTATVQKRPKR
YFVFEGDVVSILGTLDAAVILGMQRPQPDRLRRAVVAAAIRLAIAMRGSGVLAQLEAGKINFSRAIDFA
E-LY-GLEAA-KFAIS-VALDFRVRNHKVKTKRARPYKGHTKFLHKASAEKGLVAEGVDTIA
>GCA_000153305
AVIKPFLYSEYAPVLMIRQQQVATVSGAGSPSLRAKTVYAKSGLQRKTSTKWVASPFYY-
GVLAMAKIVQLVSISVDNRSVCTVAQVVEQEVVVHGLVALDAVKRKIALDEIDGMTSEDPAKVVVYD
GELELKRFRDAEDRADMLPVLLGLTLKIKFDPHQPVVHDLPIPVIFEVV-
GIMAQKGTIEVDECAISTAVSDVDPIAYVSPIDLEAVAGEKVAYIDQEAAISAAKDEIALEELALESAVLV
VGI-PLIIPV-FAEAG-T-MSFANRSRALATIVNLAVAHPALTRARIE-AMISEM-
MRAARAAALADRRGTAEAIRIVKRMQRSAGLADDPELVILEDAAEGNAQWAGQIYDDPLSGSSESD
SAGNIVHAANKRQALARSITYLRKVELSDKANAPLMRGVKHNSKSRASRKLDDAIAALNFPNVPEGIA
IRAEAGKPETTLLHDATEGEEILLIIVRWDVTWKTAAFILLALRAGNIVGLIALGGIAAVGKPDGVLNRHH
KMNGSLIEQKRIMDKMTLRLRASLKQKLGAEAVIILV--------------------------------------
-----
DSEATVEVLRGVAEGPADAQALLRIRVDHTLVKLSVLLIPQVPVLVESVTIREGVGKALQIIQLDVYLLR
DMGTGEDTLAKSRSRELIRDVLAVDPITSVQLAYGRLKERASKNLTLKDLETMF--
LAITAGVTEPRMATLPAGVYRTLVEAGPRAAVGTLFLLLDEAGRLYGISGDLRELTKSIKSLIGLVDASVP
KVLSAVYSTGRLKVRYADVTVLTSDAGLYAYAEERKEVLRMVPVYVTTAAKRRQLVLFALSLRGGHRR
ARAAIGVLIAAAIRESHAENGAVHHAAPAILLDDAEITGSEMMISRQSALDLKTVQKVDVIVVFPEFQE
KMVVSLRFFCTEVAIFVIALIVTGQMLSREPASAQESGAGFQK------------------------------
-------------YTLQPKKKQMPEEGDTDKVDGFELIHVVKEDASN-EELEMPEAMALAVE-

DSTINVTAGVLALSGEMAAVERIDSKRKMAQAVAKWNISKLALADLSLSGAAKVRALD-
IEMAHLSAALDSKKIWFLFLPGANNAELEEEKLDGAGASSADAAEDRQLAILLVQPVLVVIQDSNAAG
LIANDGVDEWGTMAYKKHYAFAARMRLHMRTID------
QLEPNKQKSEKVKTVKLEGDTRFRQLAVEGVKEQMP-
LHALNPDFAVLVPVLVSPAVGRVAAREDVAGARVPCDASL-
RELQKSQKGSALPISAVRLLGMGAARAMIEEDIMVAGKIAA-
LLRIGALQTICHIVLRNDSEATLPWRLSVAERLEADTRRGGGYAARPFKSKPSLCNVQAHAKVSEPIQEI
AAGYIATEERFVFPVEDIAIQLILANTHSRVGSEKRAGEGHLHEMAKELVAKAVLLVKSEPDTNTEGML
DYFTTGQPTDLLFEDCLDIIIDLRRMNIV--------------------------------------
VHLNTV-HVVVAGMRPVGDYEIMAAQAPSGLVQTERVDERH-
RERTKMWMYGAMEELWLMSVRYADWVVDTSIGAIILHVYAQTINRAFSLTRLQLAGWPPEADRWW
NTWQLWLNKDGFFGYAKAFRAVKAILKADLDDYELKDTEGVGRMQSRQELRVGYRIRRQVEFARLQ
PRTDILLQSHTRLAMIRHALTLVAILILREIVAEFT-
VVAEEDVAMGHVECVLADSFAFTAKNADIGPVGLRTPETVLGDGTVFRAAILGKVEFENTVAEFRYRF
VTDGGRRLGVTWVTLRNMLYARIWLPPAAAALKDLQPIRFFLGQPSLDLAQFDGGFE-
ISKARADRLMAGALVEEILEGVARFADPEATTHQSRKARHFKYYGEEVDPVHLAYI----
ILVATQVTISDRLLKDRYVLNLKI-RGFTSLLLLAAQLGEKSPAATIITVDTIMGLGALILFIILL-
RDLVLLETLYHRYVY-VMKGYEAAKDVADADPEHLLALAAVTRDVEWVEIIPAFFYYFKTAG-
FEPFNTAKALEFFGLFEERLRIVDVLAVPPDRVEALVEVVALAGTLATSAEALPGVIACGLCEPNAAEVL
EAGQDWNLHRHLGITLAFFEARQSVKKWFDKGLHKDVAATGLREAGAFTTPFHAEVEARKCVPVIHR
VLDLGEIEAALIEIDSAVTVADEEQTLADQPTTREKAVGAVGGVVLAGTMNMGNNLEQVERGNNIIIE
LEEEQYHAVNG-
AADGKMTDKVLMVLSAALFPVLARMVSANALDRGMFDLLIEAPNDVDNLDHGRELSIPAATIHGETT
PLYAEAEIERIAWVAPLHMLGTLDAVLLFEPDQYSFMSMMIDASLEITETAIVLDDYHVLEKGGRPPEFR
VVLIKGLHIRVLAGIGTIVVVGSAGRSEVTAYIAG-
AVPITLNKHTFLPAFAPSSCVVIDIFSTLLRPPERSAVQTLILETVAEL-LEVTRIENDKLERL-
LLLPGLDLAALLGLAAAAEDFAAIVTIDAAIVAILSVAAEAVVLRELLLSLAEIAVTALALALALLLMLTRD
RGLSQREHAREAQMKVLIQGGAVLQISAAEDMVLAATADKVRLDTDEGTTSAFASLNLATVSDGFYV
LSSAVLQLSSDLMMVVGVARVLADKEHKNALVVRHPEQVKTQPTYAEGSPGMVLQTLTAAIPPLAIK
PEAVELLWTLVAALWQVKARLASATRPHEERILVLLLALSLIVVLMQSEGGGIGGG-
MGSAAKGLAAFSILTMKAAVGDEVRKVTRRVMDLNSRLGRPEESVNPEFRGAELEQSKYEAANVEVD
TMETIRRFVDIPFLVSTRTQHNESEDRGARMVLDVTRVRFIDL-
VTKRSEIILAARAETDGIISREMQKDLQALHPEAAALGKIAALQHFIALNDGHISNYVTAAIHSVLGMLL
LVLFISQVRVSKVTWPTRREVTMVFLMATLAIFFLVIGL--
QLVFMLSGISYQQLTTLVLGEGVMVVRQTSTFPADS-
IGILQNTYADLICAGRVTKADIEKSLHIVVGARVARGTWYVIYHYEITQAQAEAGIMQRAFRQATLSQS
LIDGRMEVKIKVVTSDRYA-
TYEIKGGQHRKGTRFNRVKIGHNIPVEILPRLKLAYGDPNLILVKTFGAVVQKRFAQALTHIRHGAVTIG
PMEVASPYIKNKKARHAPGDSVVDLKPDNILRSVAVVPELIWARENEDNGAVHDRYVKTTHKLQAKR
NRAVDKAQYDHKNQVHLSFIGLAVKVLPEKAIVHATAGRLLPTEAVSVTEGLRGIAATIRLDPMEKALI
AYIKRMKPQLNLGGKTDKRVVKCLNNNLDDVEYGKLGRRRIKPITIKSVYDVEHKRATELVKLLPYKDD
VESRQSQFAEK--
ALFEPLRAFMMLFNYIARCVYALSLRVVRRTILLVKTRIVVNASTVLSAIKIIGVSIRLRHKSTNVADTNHY
IKTIEARARMTLAGAGKVKLNLRPGRVGKKGGRIKGYMPYNNYNIFDGLESGRRAKVKELAKLKKVDK

EITVDGIRQKQQGGRTPKADLSNAIANSRVDVDIVGENEQEESNPRANAEMGQIFAAIDARVYSLVA
ALIGHALEAVLAVAAIWGIGKAHGLLAVRLKINAGIEFHSAA-VDDSVAAA-
YAQPAGEASALWDVSEYPSVALLLSEASVAVCRLRRRQLSELKKTKKEAYMSLTCFSGKGDRVKIVEAS
QTQAYEIVATRTAERHGIHAKGTYGKERAMMQILPTKPVKVWVPVFVNEEELLAPIRAFAETNGDGV
DVVKLQNDIKITMFEKAVASLAVTGKNSSVVYTVLEKVTELMKDIIYEQRAKAHYGRGRLRSHVVTHT
QQKRHLSMMRRTVVADPRMDAARLTDGEVSVITGATMAFVMSEQDAV--
GDVDLHYHEEPSREAALGIAGPETFLATEQPRVLVLLFLRAYLIHIVEIDTVGGFSDAATIYDILRPLQNKL
DRKLSADIAKVRMVLHDDVADMGVTAEAPRREAAGDAILSAYWINGVTANSDIILLVANATCATVPE
NIDCAIRLFYQRVTLQAKERGGDVTVARVKAQNQIEAAEGTIIYTIKGKLPGTVQMKLNIEGISRSIVKSL
AVQLFQVDTFLDDTWIATLLRAARDVVFVDAREGREAVDHVVERDTELELAPQKDIVARKIRIYTPAA
PSAVVIWDPAIEREDKADAQGTILKIYGPNLQDISCFTDIGAEPKIESLKSWDEKEKYVVHRDDIPEDI-
TLVGKSSRVNKEFGIPDVAVVAPSKPGVTESIDRGEPKAGSCGEVANMAYPQESLVNSVSSANVQKN
ISMVLGGHPVGEMGISREVERHLETNMCEIFVDKADVDVALDFYRLELTTLRVGLDPPNAKVEIDGKET
RKLMAIRDGAAKLQRHPKVSAATGTG-TAGAKARLPDDALLYDGVHHGDVKFTITDLPEGRV-
VTPVLNDVKAQLKIDKVMLKTFHVVTLSFSNDKGEEHLIKLRLNALKKSTRHYEMLLKIRETECAESPHA
IPVGKILKIDSLLQTICRAHYTEIEPLLADDLTALGVLGDQGLLHVVPRLMILAVMHLWGLEAALELPRW
GLQARRIVTDQTLGLAARERHYSY-AISEVVGNTFFAMTKDDDTEQRLHARNPV-VLL-WGKFAMV-
ISPSGLTLASTPETALEHGNEADLILSRERLKDEMAYYVLNVLLVFVDEVILGYGSGRDPLRFLVAEQAV
TGRANPGLVCLTR-
TFPLDLQRDATFVLVFEDSTMKNLSHKKMHMNLVLIKSKGWVVNVSTKILISDQAIASYAEDAGAHT
QPSESALAAITSRVIAPIVLVVSQKPTAPSKTLFLEALNLYEDVTANVLNPPKKYYIFEGDVISVLRTVDA
AVLKGLQRPQPDRMRRAVIAAAVRLAIAG------HSLEDGTINFARALSFLR-
EYPALAPLLRAPLGVQAVEFRVRNHKTSTKRDRPYKGHTKFLHKADIEKSLVAAGVDTLA
>GCA_000153725
AVLKPVSLNTYAPVLMIRQSQVATVSGAGSPSLRAKTVFRKSGLQRKVSTKWVASNLYF-
GVLQMGKIVQLVSISVDNRAVCTVAQVVEQEVVVRGLVALAAVKRKIALQEISGMTTEDPAKVIEFEE
EIDLKRFRDADDRADLLPVLLGLTLSIKFDPHEPVVYDLPVPVIFEVL-
GIMAERGGIEVDECAISTAVSDVDPIAYVSPID-----
GDKMAYIDQQAAVSAAKEEIGLEELALESTVLVVKI-PLLLPV-FGEDGDN-
MSFAKRSRELSSIVDLAVTAVTLTRAR----IAAAM-
MGRAQAAALAERRGTAEEIRIVKRMQKAAGLADDPDLVMLEDAAEGNAAWAGQIYDDPVSGSSES
DSQGNIVHAANKRQNFARSITYLRKVEISDQAAAPLMRGVKHNSKSRASRKVDDAIAELNFPNVPEG
IAIRAEQGKPDHTLLHDATETEEILMLIVRWDVTWKTAAIIGLMFTAGDAVGMIDLGGIAAVGKPDGLL
NRHHKLNGSLIEQKRVIDKLTLRVRAALKQKLGAESVIILVVKVQGDLVADATVQM-
GGVVGAVVAIQGDVIFVRRHRQTVVTDSESTVEVLRGVAEGPADAQALLRIRVDHTLVEVSVLLIPE-
PVLVDSVSIREGVGKALQIIQLNVYLLRDMGTGDETLAESNSRELIREVLTIDPITGVTLAYGGFKERASK
GLTLKEIETL---
RAILADVTEPRMENLPEGVCRTFVQAGPRATVGMIFLLLDEAGRIYEIDGDLRELPKTVKSLIGLVDAKA
PKVLSASYATERLKIRFADVTVLESEADLYAYAEERKELLRMVPVYVINEAKRRQLVIFALSLKGGHRRAR
AALGVLLAAALLESQAEQGEVHHQAPEILLDNAEVVGTSVMVSRQSALELKHLLKIDVIVVFPEFQEK
M-----------------------------PEHAQESGAGFQKRIRRHSMLFALECTFDRRNLGDA--
ADLFLDLGLAPPRYGVVYINQPKKKQMPSEPDGDRVDGFELVNAVKADGRT-
HELEMPEAMALAVE-
SAKINLTAGVLALKGGMAAVERVDSKRKLGLAATKWSIAKLSGIDVLLAGAAPVRATG-

IELADLSAALSAKKVDALYLLGASNAELEEEKLDGAGNSTADVAEDRQRLILLVHPVLVVIQDSNAAGL
VSNGGVEEWGTMAYKKHYAFAARMRLHMRTID------
ELEPNKQKSEKVKTVKLEGDTRFRQLAVEGVKEQMP-
LHALNPDFAVLVAVLVSPAVGQVAADTTVLGAEVPVDFSL-
RELQKSQTGAALPISAVRLLEMRAARGMIDEDIMVAGKVAA-
LLKIRALETVLKVVLRNDSEAWGPWRLSVAERLEADTARASGYAARPFKTKPSLCNTQAHAKVADPIQ
EIAAGYVATEERFVFPVEDIDIQIVLASPHSRVGEAKRAGEGHLHEMAKELVAKAVLMLQNEPPTTTEG
LLNYLTTNQPTDLLFPDCLDVIIDLRRMAVV---
IHLTTV-
HVVVEGTSPVGDFEIMSAAAASGLVQVERVDDRTGRERTKMWMYGGMEELWLLSLTYADWVPDC
AIGAIILHVYAQTINRAFSLTRLQLEGWPKEADRWWNTWQLWLNKDGFFGYEAAFRAQAQVLKADL
FDYQMSDVEGVGRYQSRQQLRVGYRIRDQVEFSRLDPRTDILLQAKTRLSVIRHALKVVAILVLREVV
GEFSLVVAMEDVAMGHVECVLADSFDFTEKNAHIGPVGLRTPDPIFGDGTVLKEAVLGKIDLRNTVAE
FRYRMITDGGRELGVKWITIQNILYPRIWVPPAQAALKNLLPICFFLSHPQLDM---------
ISKARADKLMAGALVEEILEGVARFAKPEATTHQSRKTKHFKYYGEEVDPVHLAYILEVFALVATQVTVS
DRLLKDRYVLNLKV-RGFTSLLWMGAQLAEKGPAATIITVGTIAGLGALVLFIILL-
RDHVLLETLFYRYVY--MRGYSAAKDVADADPEHLL-LAAVTRDVEWIEIVPAFFYYFKTAT-
FEPFNTAVAKEFFGLFEERL---
SPNAAAVLEADQDWNLHRSMGITLAFYKAQKSVKGWFDKGLHKAVAETQLSEAGAMTSPFAAETE
AKQCVPAVHRVIDLGEIEAALIEIDSKVVVADEEQTLADKPTTREKSVGCVGGVVLAGTMNMGANLE
QVERGNNIIIELEEEQYHAVNGKKADGKLTDKVLMV-
SAALFPVLARWASANALPRGVLGLLIEAPSDADNLDHGGEMSIPAASVHAETAPLYAEADIDKIAWIA
PIHLLGTLDAVL-
FEPDQYSFISMMIDASLEITESAEVLEDYHVLEKGARPSSFRVILIKGLHIRLLAAVGTIIVVGSAGRGEITA
YIQTRAVPITLNKHTFLPAFSASSCHAVDIFSTMLRPPERSSVQMLILETVTEL-
LDVTRGENDKLERIEVLLPGLDAAELLELVAAAEDFANIVTIGAAITAILSVAAERVVLRELLLTKAEIAVT
ALALAIALLLMLTPDGGLTQREEAREEQMKELIQGGAVLQVHAADRMVLASTADKVRLDTDEGTTSA
FASLNLATVSDGFYVLSSAVLQMSSDLMMIVTVARVLADKESKNALVVRHPEQVKTQPTYAEGSPG
MVLQTLTGMIPPLAIKPEQVELLWTLVAALWRVKAHLAEAARPREERILVLLLALSLIAVLLQSEGGGM
GGG--
SAAAKILAGFSILTLKASVGDEVRKVTRRVMDLNSRLGRPEEAVNPEFRGEELEQSKYEAENVEVDCVE
TVRRFVDIPFLVSIRTQHNESEDRGARLVLDVTRVRFIDL-
VTKKSEIILVERAETDGVIAREMAKDLQALQPEIAALGKIAALQHFISLNDGHISNYVTAEIHSVLGMLLL
VLFIQQTRVAKIVWPTRREVTTVFIMAALASFFIVIGLG-
NLVFMVGGISPLQITTLVLGEGVMAVRQTSTFPAES-
VTILQNTYADLICAGRVTKADIESSLHIVVGARVARGTWYVIYNYEISQAQGEAGILQRAFRQATLSQS
LIDGRMEVKIKVVTSDRYA-
TYEIKGGQHRKGTRFHRVKVGHNIPVEVLPRLKMAFGPPNLILVKTFGAVVQKRFAQPLTHVRHGAVT
IGPMEVASPYIENKKARHAPGDSVVDVKPDNIVRSVAVVPELIWARENEDQSAVHDKYVKTTHKLQA
KRNAAVDKAQYDKRNQVHLSFIGLAVKVLPEQA--------------------------------------
---
RMRPQLNLAGKTDKKIVKCLNNGLDDVEYGKLGRRRIKPLTIKSVYDVEHREATELVKLMPYKDDVEG
RKSQFAEK--
AIFEPLRAFMMLFNYIARAVYALSLRVVRRTILLVKCKVVVNANTVLSAIKIIGVSIRLRHKTTNVADTKH

HIKTIEARARLTLAGAPAAKLNLRPGKIGKKGGRIKGYMPYKNYNIFDGLASGRRAATKELAKLKKVDKT
IEVGGIRTRQ-
GGRLPQADLSNAIANTRVGVDIVGENEQEESNPRANAEMGQVFAAIDARVYHLVAALIGHALEAVL
ALCAIWGIGKAHALLAD--RVNAGIEFHSAA-VETAVAAA-
FAQPAGEATVIADVSESYLGALALTDASLDVLRLRRRQLG----------------------------------
----
RFGIKAKGHYGKERAMMQITPTKPVKVWVPVFVAEDELLAPIRAFAETNGDGITVVKLTTDIKITMFEK
GVASLAVTGKSTTVVYTVLEKVTELAKDIIYEQRAKAHYGRGRLRSHVVTHTKQKRHLSMIKRTVVAKP
RMEAARLTDGEVTVITGATMAFVMSEQDAV--
GDVDMHFHEEPRREADLGIAGPETFLSTEQPRVLALLFLREYLIHIVEIDTVGGYNDAATIYELLRAMEN
KLDRKLSAEIAKVRMCLHDDVADMGIRQEAPRREQAGDSILSAYWINGVTANADIILLVANLTCATAP
ENIDCAVRLFYQRVTLQAKERGGDVSVALIPAKNQIEAAEGTVIYTIKGENPGTTQMKLNVEAISKSIVK
SLAVQLYQVDLFINDTWIATLLRAADDVVYVEASEGREAVDHVIERDTELEMAPQKDIIRRKIRIYTPAA
PSAVVIWDPAIDRE------------------DISCFTDIGAEPKIESLKAWDEKEKYVVHRDDIPPDI-
TMVGKSAHVNKEFGIPDVEVCAPAKPGVTESIDRGEPKAGTCGEVANLPYPQESLVNRVSSANVQKN
ISMVLGGNPVGEMGISREVERHLETNMCEIFVDKDDVDVALDFYRLDLT-
LRVGLDRPNAKVEVDGKESRKLMAVLDGEADLQRHPKVSEAAGTRVGAGRKARLPDDTLIFDGVLH
GDLSFTATDLPEGRV-
VTPVTIKVKAQLKIDKVILKTFHVVTLSFSSQKGEEHLIKLRLNALKKSTRHYEMLLKIRDTECAQSPHAIP
VGKILKIDKLLQTICEAHYNEIEPLLADDLTALGVKGDQGLLHTVPRLMILAVMTLWGLEAALTHPRWG
LMARRLVTDVTMGLTMRERHWTY-ALSEVVGNTFFARTKDDDTEQRLHARNPI-VLL-YGKFGMT-
ISPSGLKLESTATTAAEHGNDILLILSAERLQDDMSYFVLRVLLVFVDEIILGYGSGDDPIKFLVAEQAVT
GRANPQLVCLTRRTFPLDCQRDVVFVLVFEDSTMKNLSHKKMHMNLVLIKSKGWIVNVSTKILISDQ
AIASYAEDAGAHTQPSESALAAITSRVIAPIVLVVSQKPAAPSKTMFLEALNLYDQVTTTVENRPKRYYI
FEGDLISVLDPIDAAVLKGMQRPQPDRLRRAVVAAAIRLAIAGLASGVLCLLEAGLLNFARAVTPAVRV
Y--SEAAMSLVVPLPSV---
>GCA_000153745
AVIKPVVMNKYAPVLMIAQSEVATVSGAGSPSLRAKTVYAKSGLHRKTKTKWVAGPLYF-
GVLKSASIVQLISISVDQRAVCTVAQLVEQTVVVHGIVAFDVVKKKIALEEIGGMTSEDPAKVTEYEEEV
DLNRLREAEDRADLLSVMLGLTLAIKFDPHQPMVSDLPVPVIYEVI-
GIMADKGGIDVNDCSISTEISDVDPIEYVSPMDLSAVAGDSISYLDQESAVAAAKDLIDLEEFCLESEVVI
VGIDPLILPI-FADDAGTTMSFANRSRELKTIVDLAVSNYEKGRARIDD-IASSM-
LNKLLSEALCEKRGTAEEIRLVKRMQQADGLSNNPELSILEDNSEGNSNWAGMIYDDPVSNSSESAS
AGNIVVAANKRQATERSITFLRKTEISDQAEAPLMRGVKHNAKSRSSRKINDAIIELNFANVPLGIAIRA
ESNLHKDTLLHDALEKEQLLM-
VIKFHITWKTAIFILIIFIPGNIIGIIEIGGIASIGRKQAGLNRHHKLNGSLIEQRPIMEKMTLRLRASLKQKLG
KDQVIIFVVKVAGDLIGNAKVQM-
GGLVGAVVALQGPVIYVRRHRQTLVTDSEATVEVLRGVAEGPADAKVLLRIRVSHTIVEVTVLLIPK-
PVLVDSATIREGVGKTLQIIQLDVYLIRDMGTGSETVAKSTSREMLRDVLLIDAVTGVRLAYGEIKERSN
KLVSLKDLRTLL--
LALLKGTTEPRMENLAEGVCRAFIVAGPEAAVGTLFIIQDDAGKIYTVDGDLRENTKTVKALISLVDAKS
PKILSAIYSITKLKIRYAVCMVLIAEVNLYSYQEERKQILRMVAKYITQEKKRRQLVIFALSLKGGHKKARA
SLNILIAAAIIESQAENGKVHHEEPEVLLDDAEIVGSSIMVSRQATLSIKDYIKIDVIVVFPEFQEKMVVSL
RFFCTEVSIFVIALICTVGEMLNREPNSAQENGAGYQKRIRKQSLLFALSVLFEARNLNDA-

TKDLFLNLGLFDPRYGIIYKNQPKKKQMPSEPDKEKVNGFELVNAINADNSDNHVLEMGEAMALAVE
-DATINVSAGVLALNGGMTGVERVESKRKYSIAAEKWNISKINMLDLTISGANPVRCTN-
IELADVTAALDKKSLLK--

LHALNPDFAVLVAVLVTPAVGRVAATSEVVGAQVPCDYSL-
KELQKSKSSEVLAISGIKIVTLNSARAMIDEDIFVATKVAAKLLKITARETIFSIILMNDSEACIPWRLSVAE
RLEADMARASGYAARPFQTKPSLCETQAHFKLAESIKEIAAGYVAMEKKFVYPVEGIAIQLILDGPHSR
VGDSKRAGQGHLHEMAKELYVKAVLMIKANPATDTEGIARYMTTNQPTDLLFPESLDIVIDMRRMSV
V---IQLNSI-
HFVVPGTSEIAAFQIMAAAAASGLVQIEQVDDRTGPERTSMWMYGAMEELWTFSVTYAGWISNCKI
GIIILNVYAQELNRAFSLKRLQLDGWPKEAERWWNTWQLWLNKDGFFGYQAAFRA-
KRVLKAELYDYDMSDTEGVSRFLSRQQLRVGYRIREQVKYNKLTKRSDILLQANTRLSIIRHELALVSVLI
IRDIISEFLVVVAMEDVAMGHVECVLADKFDFTEKSASIGPAGLRTPDIIYGDGTVLKEALLGKVSLANT
VSNFRYLLLEDGGRELKVSWIDF-
NMIYIRIWLPPAEAALKNLQPICFFLSHPELDLLLQTSIFSGISKARANKLMASALVEEILEDLARFAKPEA
TMHQSPKNEHFRYYGEEVEPVFLAYILELFALVSTQVTVSDRLLKDRYVLNLKVFRSITSLSWVGVNIVD
KGPAATVITVGTVSGLGALVLFIILL-RLSVLIETLFFRYVY--
MLGYQAAKDVADAFPEHFTPMAAITRDLEWIEVIPAFFYYFSTAK-
FEPFNTAVAREFFGLFNQRLRIIEVIVVPPERVLELVEFVALAGTLPTASDVLPGIIAFE-
CEPNAASVMEASQDYNLHRHLGITMAFFEARASVKGYFDKGLHKNVAEIGLKEAGALTDPFHGGAD
AKRCVPAVHRILDLGLIEAALIEIDSAVLVGDSEQKLADQPTSRDKSVGAIGGVILAGTMNMGLAMNA
CERTDDMLIELEEEGYSAING-
KSDEKLTNKILMVISLAIFPVIEALASANSLNKGVFDSLIDRPNSADNLDHGGEMVIPAPTIFAETAVLYA
EDEIDKLSWIAGLHLLGTLHAVLQFEPDFIGFISMMINASLEIMETAEVLDDYHVLEKGGRPPKFRVVLIK
GLHIRILSAIDTIIVVGSAGRGEITAYIQEDAVPISLNKKSFLPAFSASSCISVDILSTMLRPPERSSIPTLILET
VAEI-
LEVTRGEVEKLERLFAILPGLDLNDLLTLLSYAKDFANIVTINAAITAILSVAAEEVVLRDLLLTLAEIAISSL
AMAIALLLMLIRDWGLSQKEKNRENQLKEIIQGGVILNVRAEEDIVLSAVSEKVRLGTDEGTTSSFASLN
LATVSDGFYVLSSAVLQMSSDLMMIIEVQRVLADKEAKNALVVSHPEQVKTQPTYAEGSPGMVLQTL
TNHIPPLAIRPQNIELLWTLVAALWKVKAHIREAARPIIERVLILILSVSLIIVLLQSEGGGIGGG--
GPKPKIIAAFSILTIKAAVGDQVRKVARRVMELNNRLGKPEENVTPEFRGAELEQMKYEAKSVEVDCTE
TIRRFVDIPFLVSIRTQHNESEDRGSRMVLDVIRVRFIDL-
VTKKSEIILVERAETDGVISREMQKDLQALQAEFVALGKVAFLQHFIALSDGHISNYVTATIHSALGMLL
LVLFMQQVRVSKVVWPNRRETTMVFIMATLAIFFGAIGLIISLVFVLSGISPLQITTLVLGEGVMAVKQT
STFPGDS-
VTILQNTYAKLVCAGRVSKADIEASLHIVVAARVARGTWYVIYRYEISQAQDQSGIMQRSFRQATLSK
SLISGRMETKVKVLTADRYA-
THEIKGGQHRKGTRYNRVKVGHNIPVEVIARIKMAFVDPNLVLVKTFGAVVQKRYAQPLTHVRHGAV
TIGPMEVASPYIGSKKSRHAPGDSAVDLKPDNIVRSVAVVVELIWARENEDNSAVHDKYVTKTHKIAA
KRNTAVDKAQYDKRNQVELSFIGVAVKVLPEQAIVTATAGTLMPTETVTVTEGLKGVAATIRLDPMEK
TLIAYIKKMRPQLNLQNKADKKIVKTLNNGLDDVEYGKLGRRKIKPLTIKSVYDVEHEEATELVKLIPYKD
DVETRLSKFAEK--
ALFEPLSAFMMLFGYIARAIYALSLRVVRRTIILVKTKIIVNADTVLSAIKFIGVSIRLRHKTTNVAETKHHIK
TIEKRARMRLAGAPNVKLNLSPGRIGTKGGRIKGYMPYNNYNIFDKLESGRRAKVAELAKLKKVDKEIT

VSGVRTKQQGGRVSVEQLSNALPETRVGVAIIGENYQKESNPRANAEMGQVFALIDAKVYDLVGRLK
GHALKAVLAIVVIWGVGKCHSVLAVSFRMNAGIELHCAA-IDEAVACA-YAQSAGEA-
IIADLSEGDANELGLTTQTLETCRARRKRLSELKKTKKEAYMELTCFSGKGEKVKIIEASQTQAYEVLSTR
TAERFGIKAKGSFGKERAMMQIVPTKPVKVWVPVFVSEAELLAPVRSFAETNGDGVTVVKLITDVKVT
MFEKAIASLAVEGKVTTLVYTVLEKVTEVMRDIIYQQRAKAHYGRGRLRSHVVTHTKSKRHLSLMRRT
VVVDLSMEAARLVGGEVSVITSATIAFVMAEQESV--
GEVEYHFHNEPRNEADLGIAGNETFLATEQPRLLCILFLRAYLLHIVDIDTQGNFAESATIYDILRAMSRK
LDQRLSADIAKTRMCLSDDLPDMGVREEAARRDSAGDIMLSSYFLNGVTANADIILLVANLELKTVPE
NIDKAIRLFYQRITLQARERNGDVSVSIIPAANQIEAAEGTVIYTIKGKTPSSKQMKLNIEAISRSIVKSLAV
QLFQVNLFLNDTWIDTLLRTAIDVAFVDAKDGNEAIDHVLERDEGTEMAPQKDIVRRLVRIFTPAAPS
AVVIWDPAIDRTDKAEAQGTILKIYGPDLQDIACFTDIGAEPKIQSIKSWDEKEKYVVHRDDIPEDI-
TLVGKSSAVNKEFSIPDVAVCAPNKPGITESIDRGEPKAGTCGEVANLPYPQESLVNRVSSANVQKNIT
MVLGGHPVGELGISRSVERHLETSMCDIFIDKDNVDVALSFFVLQLTTFRVGLDSPNAKIEIKGKESRKV
MAVLDGEANLQRHPKISEADGI-NKAGKRAKMDDDSLIFDGVNHGDIKFTSTDLPHGRV-
VTPVIHNVKAQLEIDKVLLKTFHVVTLAFSNDKGEEHLAKLRLQALKKATRHYEMLVKIRETECADSPH
AVDVGKILKIDDLMHTICQSHYTAIEPLLAQDLTALGVMGQQGLLHIVPRLMILSVLYLWDIEAQTIIEN
WDLQASALVTDQTLGLAFRERDWSF-AISEVINNTFFARSAQDETEQRLHARNPI-ILL-
YGRIGLLVISPSGLRLDSTGPTKLGEGNSSSVILSNERLQDSMEYYVLALLLVFVDRVILVFGSGKDPLKFL
VAEQAVTGRANPGLVCLTRRTFPMELQRDAQFVLVLDDSTMKNLSHKKMHMNLVLIKSKGWVENIS
TKILIADQAIASYAEDAGAHTQPSESALAIVTSRVMAPIVLVVSQKPVAPSKTCFLEALALYDTVTATVQ
KKPKKYYIFEGDVVSVLDKVDASVLKGMQRPYPDRMRRAVTADAIKLAIAIRASDCFDQLEAGIINFSR
SISLAAGVYALISII-DDPVN-SALSFRVLAHKTSTKNDRPYKAHTKFLHKADATKALIADGVDTLA
>GCA_000154745
AVIKPVSLNTYAPVLMIRQQEVATVSGAGSPSLRAKTVFRKSGLQRKTMTKWVKSKLYF-
GVLKMGNIVQLVSISVDSRAVCTVAQVVEQEVVVHGLVALDAVKRKIALEEMDGMTTEDPAKVAEFE
AELDLKRFRDADARADMLPVMLGLTLEIRFDPHEPVVHDLPIPVIFEVI-
GIMADKGGIEVDGCAISNAVSDVDPISYVSPIDLAAVAGEQMAFIDQEAAVAAAREAITLEELALEAGI
LVVGISPLLLPV-FGEDGGV-MSFAARSRTLGSIVALAVAYEELTRA-IEA-IASEM-
MQAAQAEALAEKRGTAEEIRIVKRMQRSSGLADDADLVILEDAAEGNSSWAGQIYDDPISGSSESDS
AGNIVHAANKRQNFARSITFLRKAEISDKAEAPLMRGVKHNSKSRTARKLDDAIMELNFPNVPPGIAIR
ADAGKSADRLLHDATEREQILMIVVRWHVTWKTAIAIGLVLTAGDIVGLMELGGIAAVGKPDGALNR
HHKLNGSLIEQRPIIEKMTLRLRASLKEKLGKDQIIIFVVKVQGDLVADATIQMG-
GPVGAVVAIQGEVIFVRRHKQTVITDQDATV-VLKGVAEGPADAQALLRIRVSHTLVDLSVLLIPE-
PVLVDSVSIREGVGKALQIIQLDVYLLRDMGTGEETLAESKSRELIREIFALDPITGVRLAYGKLKERMGK
KLALKELD-----
RSILEAVTEPRMENLPAGVCRTFITAGPRAAVGMLFLLLDDGGKLYSIDGDMRELPKSVKSLIGLVDTK
APKVLSAGYAEERLRIRFADCEVLVTEADLYAYKEERRELLRMVPVYIINEAKRRQLIMFALSLKGGHRR
TMMSLGVMIAAAIIESQAEKGTVHHKAPQVLLDDAEVVGTAIMISRQSALDLKQMVKVDIIVLFPEFQ
EKMVVSLKFFCTEVAIFVIALITTVGEMLSREPEHAQESGAGFQKRIRGHSVLFALAAVFDRTNLRDA-
TPDVFLSLGLAEARYGVLYTNQPKKKQMPSEPDGDRVDGFELVHAVKEDGSS-
QELEMPEAMALAVEADATINVSAGVLAMNGGMVAVEKVDSKRKVALAATKWSIAKLDVVDITISGA
APIRCCG-IEMADISAALGQKKV-
FLFLPGASNAELEEEKLDGAGASTADAAEDKQLSILLVKPVLVVIQDSNSAGLISNGGLDEWGTMAYK
KHYSFAARMRLHMRTID-------MEPNKQKSEKVKTVKLEGDTRFRQLAIEGVKEQMP-

LHALNPDFAV-LPLLVTPAVGSVAADERVLGADVPVDASL-
RELQKSQRGSALPITAIRLLEMRAARGMIEEDIMVAGKVAA-
LFKVRALETIVDLLLRNDTEATLPWRLSVAERLEADTKRASGFAARPFKSKPSLCKTQAHSKIADPIQEIA
AGYVAAEERFVFPVEGIDIQLILANPHSRVGTAKRAGEGHLHAMAKELLGKAVVMLRNEPNTNTEGLL
NYLTTNQPTDLLFEDCLDVIIDIRRMSVV--
VHLNTI-
HVVVPSVRDVAAFTIMSATFPSGLIQVEQVDDRTGREKTLMWMYGAMEELWLNSVRWADWVANC
AIGAIILHVYAQTINRAFSLKRLQLDGWPKEAARWWNTWQLWLNKDGFYGYDAAFRA-
KQILKATLADFDMKDVEGVGRMLSKQEIRAGYRIREQVEYARLDPRTDILLQAKTRLSIIRHALTVVAILI
LRDVAPEFTVVVAHEDVARGHVECVLAESFDFTEKDAHIGPVGLRTPAGIFGDGTVFKEAVLGKIDLA
NTVADFRYLILVDGGRALGVCWISLTNLLYTRIWLPPAQADLKDLLPICFFLQHPELDLKMTHHLFAGIS
KARAEQLMAGALVEEILEGIARFADPEATTHQSKKAKHFKYYGEEVDPVHLAYILDLFALVSTQVTVSD
RLLKDRYVLNLKVFRGLTALLWLSLQLAEKGPSATVITVGTIAGLGALVLFIILLMRDSVLVETLFYRYVY-
VMLGYEAAKDVQDADPEHLGPLAAASRDIEWIEIIPAFFYYFKTAK-
FEPFNTAVAREFFGLFKERLRIVDVLAVPPDRVEAFVQFVALAGTLATSAQALVGVIACGLCEPNAAAV
LEAERDWNLHRSMGITLAFYDARKSVKGYFDKGLHKDVAEIGLREAGALTTHFHCAVEAKRVAPAVH
RVLDLGEIEAGLIEIDSAVVVADEEQTLADKPTTRDKSVGAVGGVVLAGTMNMGANLDSVERGGNIII
ELEEDQYHAVNGKKADGKLTDKVLMVLSAAYFPVITRFSSANALDHGVFGLLIEAPSDADNLDHGGI
MTIPAPTVLTETTPLYAESDIDRIAWIAPMHMLGTLPAVLSFEPDHYSFISMMIDASLEIMETAEVLDDY
HVLEKGARPSAFRVILIKGLHIRLLAAVGTILVVGSAGRAEVTAYIQGQAVPITLRKHTFLPAFSASSCKV
VDIFSTLLRPPERSSVQMLILETVAEL-
LEVTRLTNEKLERMMILLPGLDLAALLGLLAAAEDFATIVTLGAAITAILSVAAESVVLREMLLTKAEIAIT
ALAMAMALLLMLTGDEGLTQKWVGREAQMKELIQAGAVLQIHADDAMVLAATADKVRLDTDEGT
TSAFASLNLATVSDGFYVLSSAVLQMSSDLMMIVSVARVLADKESKNALVVRHPEQVKTQPTYAEGS
PGMVLQTLTECIPPLAVKPEEVELLWTLVAALWRVKAHLANAARPHNERVLILLLALGLIVVLLQSEGG
GMGGG-
MGAAAKILAAFSILTLKASVGDEVRKVTRRVMDLSSRLGRPEEAVNPEFKGEELDQTKYEAENVEVDC
METVRRFVDIPFLVSVRTQHNESEDRGARLVLDVTRVRFIDL-
VTKRSDVILVERVETDGIIARQMAKDMQSLHPEAAALGKISQLQHFIALGDAHISNYVTAQTHSVLGM
LLLVLFIQQVRVSKVVWPTRREVTMVFVMAALAVFFIVIGLGINLVFMVGGISPLQITTLVLGEGVMAV
RQVSTFPAES-
VVILQNTYADLICAGRVTKADIESSLHIVVGARVARGTWYVIYHYEISQAQSPSGILQRAFRQATLSQSL
IDGRMDVKIKVVTSDRYA-
TYEIKGGQHRKGTRYNRVKVGHNIPVELLARVKMAFGDPNLVLVKTFGAVVQKRFAQQLTHMRHGA
VTIGPMEVSSPYIKSKKARHAPGDSVVDIKPDNVVRSVAVVPELIWARENEDQSAVHEKYVKTTHKIA
AKRNRAVDKAQYDKRNQVHLSFIGLAVKVLPEQAIV-
ATAGTLLPTESVAVTENLKGVAATIRLDPMEKTLIAYIKRMRPQLNLGGKTDNKVVKCLNNNLDDVEY
GKLGRRKIKPITIKSVYDVEHKQATELVKLMPYKDDVEGRKSQFAEK--
ALFEPLKAFMMIFGFIARAVYALHLRVVRRTILLVKTKIVVNAENVLSAIKIIGVSIRLRHKSTNVADTKHH
IKTIEARARMTLASAPSVKLNLRPGRVGKKGGRIKGYMPYKNYNIFDGLSSGRRAKVKDLAKLKKVDKT
IAVDGIRTRQ-
GGRLPKAQLSNALANTRVGVDIVGENVQEASNPRANAEMGQIFAAIDARVYSLVAALIGHALEAVLA
VCAILGIGRAHALLAVY-KMNAGIELHSAA-VAEAVAAA-YSQPAGEAAAL-
DVSEYPLRALAVTDQSVDVCRLRRRQLGELKKTKKEAYMSLTCFSGKGDKVKIVDATQTQFYEIVATRT

AERHGISAKGTYGKERAMMQITPTKPVKVWVPVFVNDDELLAPVRAFAETNGDGIDVVKLATDIKVT
MFEKGVASLAIAGKATTVVYTVLEKVTELSKDIIYEQRAKAHYGRGRLRSHVVTHTKQKRHLSMMKRT
VVADPSMDAARLTDGEVSVVTGATMAFVMSEQDAV--
GDVDMHFHEEPRREAPLGIAGPETFLSTEQPRLLALLFLREYLLHIVDIETVGNFADAATIYDILRAMQA
KLDRKLSADIAKVRMCLTDDVANMGVTDVAARRDSAGDSILSAYWINGVTANSDIILLVANLECKTA
PENIDCAVRLFYQRVTLQAKERGGDVSVELVPAKNQIEAAEGTVIYTIKGNNPSSVQMKLNIEAISRSIV
KSLAVQLFQVDSFLNDTWIATLLRAAAKVVFVDAREGKEAVDHVVERDTALELAPQKDIVARKIRIFTP
AAPSAVVIWDPAIEREDKADAQGTILMIYGPDLQDISCFTDIGAEPKIESLAAWDEKEKYVVHRDDIPE
DITTLVGKSSKVNKEFGIPDVAVCAPAKPGVTESIDRGEPKAGSCGEVANLPYPQESVVNRVSSANVQ
KNISMVLGGHPVGEMGISRGVERHLETNMCEIFVDKDDIDVALDFYKISLTPLRVGLDKPNAKVDIDG
KESRKLMAVKDGDANLQRHPKVSEESGVR-KAGEKAKLPDDTLMYDGVQHGDISFTATDLPEGRV-
VTPVFNAVKAQLEIDKVMLKSFHVVTLSFSNDKAEEHLVKLRLNALKKSTRHYEMLLKIRDTECAQSPH
AIPVGKILKIDSLLQTICKAHYTAIEPLLAEDLTALGVMGDQGLLHVVPRLMIL--
LHLCGITAQMAMAQWTLQQNAIVTDQTMGLSVRERAWSF-
AISEVTGNTFFARTKDDDTEERLHARNPI-VIL-YGKEGML-
ISPSGLKLSSTNPTDANHGNADQLILSGERLQDTMSYHILQILLVFVDQMILAWGSGDEPLKFLVAEQ
AITGRANPQLVCLSRRTFPLDLQRDAVFVLVLEDTTMKNLSHKKMHMNLVLIKSKGWVENVSTKILIS
DQAIASYAEDAGAHTQPSESALAVITSRVIAPIVLVVSQKPAAPSKTLFLEALNLYDEITATVLKRPKRYFI
FEGDVVSILDRMDAAVLLGMQRPQPDRLRRAVVARAIRLAIALRGSGILAQLEGGALNFSRAVNFAE-
TYPGLEAALATAIA-SALEFRVRNHKTSTKRDRPYKGHTKFLHKAEDAKALVAEGVDTLA
>GCA_000154765
AVIKPVSLNTYAPVLMIRQQEVATVSGAGSPSLRAKTVFRKSGLQRKTMTKWVKSKLYF-
GVLKMGNIVQLVSISVDSRAVCTVAQVVEQEVVVHGLVALDAVKRKIALEEMDGMTTEDPAKVAEFE
AELDLKRFRDADARADMLPVMLGLTLEIRFDPHEPVVHDLPIPVIFEVI-
GIMADKGGIEVDGCAISNAVSDVDPISYVSPIDLAAVAGEQMAFIDQEAAVAAAREAITLEELALEAGI
LVVGISPLLLPV-FGEDGGV-MSFAARSRTLGSIVALAVAYEELTRA-IEA-IASEM-
MQAAQAEALAEKRGTAEEIRIVKRMQRSSGLADDADLVILEDAAEGNSSWAGQIYDDPISGSSESDS
AGNIVHAANKRQNFARSITFLRKAEISDKAEAPLMRGVKHNSKSRTARKLDDAIMELNFPNVPPGIAIR
ADAGKSADRLLHDATEREQILMIVVRWHVTWKTAIAIGLVLTAGDIVGLMELGGIAAVGKPDGALNR
HHKLNGSLIEQRPIIEKMTLRLRASLKEKLGKDQIIIFVVKVQGDLVADATIQMG-
GPVGAVVAVQGEVIFVRRHKQTVITDQDATV-VLKGVAEGPADAQALLRIRVSHTLVDLSVLLIPE-
PVLVDSVSIREGVGKALQIIQLDVYLLRDMGTGEETLAESKSRELIREIFALDPITGVRLAYGKLKERMGK
KLALKELD-----
RSILEAVTEPRMENLPAGVCRTFITAGPRAAVGMLFLLLDDGGKLYSIDGDMRELPKSVKSLIGLVDTK
APKVLSAGYAEERLRIRFADCEVLVTEADLYAYKEERRELLRMVPVYIINEAKRRQLIMFALSLKGGHRR
TMMALGVMIAAAIIESQAEKGTVHHKAPQVLLDDAEVVGTAIMISRQSALDLKQMVKVDIIVLFPEFQ
EKMVVSLKFFCTEVAIFVIALITTVGEMLSREPEHAQESGAGFQKRIRGHSVLFALAAVFDRTNLRDA-
TPDVFLSLGLAEPRYGVLYTNQPKKKQMPSEPDGDRVDGFELVHAVKEDGSS-
QELEMPEAMALAVEADATINVSAGVLAMNGGMVAVEKVDSKRKVALAATKWSIAKLDVVDITISGA
APIRCCGNIEMADISAALGQKKV-
FLFLPGASNAELEEEKLDGAGASTADAAEDKQLSILLVKPVLVVIQDSNSAGLISNGGLDEWGTMAYK
KHYSFAARMRLHMRTID-------MEPNKQKSEKVKTVKLEGDTRFRQLAIEGVKEQMP-
LHALNPDFAV-LPLLVTPAVGSVAADERVLGADVPVDASL-
RELQKSQRGSALPITAIRLLEMRAARGMIEEDIMVAGKVAA-

LFKVRALETIVDLLLRNDTEATLPWRLSVAERLEADTKRASGFAARPFKSKPSLCKTQAHSKIADPIQEIA
AGYVAAEERFVFPVEGIDIQLILANPHSRVGTAKRAGEGHLHAMAKELLGKAVVMLRNEPNTNTEGLL
NYLTTNQPTDLLFEDCLDVIIDIRRMSVV--
VHLNTI-
HTVVPGARDVAAFTIMSANFESGLIQVEQVDDRTGREKTLMWMYGAMEELWLNSVRWADWVAN
CAIGAIILHVYAQTINRAFSLQRLQLDGWPKEAARWWNTWQLWLNKDGFYGYDAAFRA-
KQILKATLADFEMKDVEGVGRMLSKQEIRAGYRIREQVEYARLDPRTDILLQAKTRLSIIRHALTVVAILIL
RDVAPEFTVVVAHEDVARGHVECVLAESFDFTEKDAHIGPVGLRTPAEIFGDGTVFKEAVLGKIDLANT
VADFRYLILVDGGRALGVCWISLTNLLYTRIWLPPAQADLKDLLPICFFLQHPELDL---------
ISKARAEQLMAGALVEEILEGIARFADPEATTHQSKKAKHFKYYGEEVDPVHLAYILDLFALVSTQVTVS
DRLLKDRYVLNLKVFRGLTALLWLSLQLAEKGPSATVITVGTIAGLGALVLFIILLMRDSVLVETLFYRYVY
-VMLGYEAAKDVQDADPEHLGPLAAASRDIEWIEIIPAFFYYFKTAK-
FEPFNTAVAREFFGLFKERLRIVDVLAVPPDRVEAFVQFVALAGTLATSAQALVGVIACGLCEPNAAAV
LEAERDWNLHRSMGITLAFYDARKSVKGYFDKGLHKDVAEIGLREAGALTTHFHCAVEAKRVAPAVH
RVLDLGEIEAGLIEIDSAVVVADEEQTLADKPTTRDKSVGAVGGVVLAGTMNMGANLDSVERGGNIII
ELEEDQYHAVNGKKADGKLTDKVLMVLSAAYFPVITRFSSANALDHGVFGLLIEAPSDADNLDHGGI
MTIPAPTVLTETTPLYAESDIDRIAWIAPMHMLGTLPAVLSFEPDHYSFISMMIDASLEIMETAEVLDDY
HVLEKGARPSAFRVILIKGLHIRLLAAVGTILVVGSAGRAEVTAYIQGQAVPITLRKHTFLPAFSASSCKV
VDIFSTLLRPPERSSVQMLILETVAEL-
LEVTRLTNEKLERMMILLPGLDLAALLGLLAAAEDFATIVTLGAAITAILSVAAESVVLREMLLTKAEIAIT
ALAMAMALLLMLTGDEGLTQKWVGREAQMKELIQAGAVLQIHADDAMVLAATAD------------
----
IVSVARVLADKESKNALVVRHPEQVKTQPTYAEGSPGMVLQTLTECIPPLAVKPEEVELLWTLVAALW
RVKAHLANAARPHNERVLILLLALGLIVVLLQSEGGGMGGG-
MGAAAKILAAFSILTLKASVGDEVRKVTRRVMDLSSRLGRPEEAVNPEFKGEELDQTKYEAENVEVDC
METVRRFVDIPFLVSVRTQHNESEDRGARLVLDVTRVRFIDL-
VTKRSDVILVERVETDGIIARQMAKDMQSLHPEAAALGKISQLQHFIALGDAHISNYVTAQTHSVLGM
LLLVLFIQQVRVSKVVWPTRREVTMVFVMAALAVFFIVIGLGINLVFMVGGISPLQITTLVLGEGVMAV
RQVSTFPAES-
VVILQNTYADLICAGRVTKADIESSLHIVVGARVARGTWYVIYHYEISQAQSPSGILQRAFRQATLSQSL
IDGRMDVKIKVVTSDRYA-
TYEIKGGQHRKGTRYNRVKVGHNIPVELLARVKMAFGDPNLVLVKTFGAVVQKRFAQQLTHMRHGA
VTIGPMEVSSPYIKSKKARHAPGDSVVDIKPDNVVRSVAVVPELIWARENEDQSAVHEKYVKTTHKIA
AKRNRAVDKAQYDKRNQVHLSFIGLAVKVLPEQAIV-
ATAGTLLPTESVAVTENLKGVAATIRLDPMEKTLIAYIKRMRPQLNLGGKTDNKVVKCLNNNLDDVEY
GKLGRRKIKPITIKSVYDVEHKQATELVKLMPYKDDVEGRKSQFAEK--
ALFEPLKAFMMIFGFIARAVYALHLRVVRRTILLVKTKIVVNAENVLSAIKIIGVSIRLRHKSTNVADTKHH
IKTIEARARMTLASAPSVKLNLRPGRVGKKGGRIKGYMPYKNYNIFDGLSSGRRAKVKDLAKLKKVDKT
IAVDGIRTRQ-
GGRLPKAQLSNALANTRVGVDIVGENVQEASNPRANAEMGQIFAAIDARVYSLVAALIGHALEAVLA
VCAILGIGRAHALLAV-LKMNAGIELHSAA-VAEAVAAA-
YSQPAGEAAVILDVSEYPLRALAVTDQSVDVCRLRRRQLGELKKTKKEAYMSLTCFSGKGDKVKIVDA
TQTQFYEIVATRTAERHGISAKGTYGKERAMMQITPTKPVKVWVPVFVNDDELLAPVRAFAETNGDG
IDVVKLTTDIKVTMFEKGVASLAIAGKATTVVYTVLEKVTELSKDIIYEQRAKAHYGRGRLRSHVVTHTK

QKRHLSMMKRTVVADPSMDASRLTDGEVSVVTGATMAFVMSEQDAV--
GDVDMHFHEEPRREAPLGIAGPETFLSTEQPRLLALLFLREYLLHIVDIETVGNFADAATIYDILRAMQA
KLDRKLSADIAKVRMCLTDDVANMGVTDVAARRDSAGDSILSAYWINGVTANSDIILLVANLECKTA
PENIDCAVRLFYQRVTLQAKERGGDVSVELVPAKNQIEAAEGTVIYTIKGNNPSSVQMKLNIEAISRSIV
KSLAVQLFQVDSFLNDTWIATLLRAAAKVVFVDAREGKEAVDHVVERDTALELAPQKDIVARKIRIFTP
AAPSAVVIWDPAIEREDKADAQGTILMIYGPDLQDISCFTDIGAEPKIESLAAWDEKEKYVVHRDDIPE
DITTLVGKSSKVNKEFGIPDVAVCAPAKPGVTESIDRGEPKAGSCGEVANLPYPQESVVNRVSSANVQ
KNISMVLGGHPVGEMGISRGVERHLETNMCEIFVDKDDIDVALDFYKISLTPLRVGLDKPNAKVDIDG
KESRKLMAVKDGDANLQRHPKVSEESGVR-KAGEKAKLPDDTLMYDGVQHGDISFTATDLPDGRV-
VTPVFNAVKAQLEIDKVMLKSFHVVTLSFSNDKAEEHLVKLRLNALKKSTRHYEMLLKIRDTECAQSPH
AIPVGKILKIDSLLQTICKAHYTAIEPLLAEDLTALGVMGDQGLLHVVPRLMIL--
LHLCGITAQMAMAQWTLQQNAIVTDQTMGLSVRERAWSF-
AISEVTGNTFFARTKDDDTEERLHARNPI-VIL-YGKEGML-
ISPSGLKLSSTNPTDANHGNADQLILSGERLQDTMSYHILQILLVFVDQMILAWGSGDEPLKFLVAEQ
AITGRANPQLVCLSRRTFPLDLQRDAVFVLVLEDTTMKNLSHKKMHMNLVLIKSKGWVENVSTKILIS
DQAIASYAEDAGAHTQPSESALAVITSRVIAPIVLVVSQKPAAPSKTLFLEALNLYDEITATVLKRPKRYFI
FEGDVVSILDRMDAAVLLGMQRPQPDRLRRAVVARAIRLAIALRGSGILAQLEGGALNFSRAVNFAE-
TYPGLEAALATAIA-SALEFRVRNHKTSTKRDRPYKGHTKFLHKAEDAKALVAEGVDTLA
>GCA_000154785
AVIKPVTQNEYAPVLMIAQSQVATVSGAGSPSLRAKTVYAKSALQRKTMTKWVAKPIYY-
GVLKMGNIVQLVSISVDARSVCTVAQLVEQEVVVHGLVALDAVKRKIALEAIDGMTTEDPAKVAQYE
EEVELKRFRDADDRSDMLPVMLGLTLKIRFDPHQPVVHDLPIPVIFEVV-
GIMADKGGIEVDDCAISQAVSDVDPIEYVSPIDLAAVAGTEMAFIDQESAVSAARDTIALDELALEGSIL
VVGIKPLILPV-FGEDAAV-MSFANRSRTLGSIVDLAVAFPRLSRA-ITG-IASEM-
MRAAQAKALAEKRGTAEEIRIVKRMSRSAGLADDPDLVMMEDAAEGNSNWAGQIYDDPISGSSESD
SAGNIVFAANKRQNFQRSITFLRKVEISDKAAAPLMRGVKHNAKSRTARKYDDAITLLNFANVPAGVA
IRADLRKPADTLLHDATQGEKILMVIVRWHVTWKTAAIIVLMTDAGNIVGMIELGGIAAVGKPDGTLN
RHHKLNGSLIEQRPIIEKMTLRLRASLKQKLGKDQVIIFVVKVQGDLVADATVQMG-
GTVGAVVAVQGEVIFVRRHKQTIVTDQEATV-ILRGVAEGPADAQALLRIRVTHTLVKISVLLIPE-
PVLVDAVTIREGVGKALQIIQLDVYLLRDMGTGDETLAESLSRETIRDVLAVDTVTGVRLAYGQIKERLA
KKLTLKDLETML--LSIL-
DITEPRMDNLPDGVCRTFVTAGPRAAVGMIFLLLDDAGKIYTIDGDLREVTKSVKSLIGLVDAKAPKVL
SASYATDRLKVRFADCEVLEAEADLYAYKEERRQMFRMVPVYIVLEAKRRQLIVFALSLKGGHRRAQA
ALGVLIAATLLESQALEGAVNHARPAILLDDAEIVGTEIMISRQSSLQIKDRVKTDVIVVFPEFQEKMVV
SLRFFCTEVAIFVIALIVTVGEMLSREPEHAQEDGAGFSKRIRAHSVLFALAVSFDRRNLRCDQLPDLFL
GLGLAPPRYGILYKNQPKKKQMPSEPDTDRVDGFELVNAVKEDGADNHELDMPEAMALAVE-
DATINVSAGVLATNGGMVAVEKVDSKRKMSLAVTKWNISKIDVWDITLSGADPTRALS-
IELADVSAALSSKKVWFLFLPGASNEELEEAKLDGAGASTAEAAEDRQAPILLVVPVLVVIQDSNTAGLI
SNGGLDEWGTMAYKKHYAFAARMRLHMRTIDGIEVGVEMNPNKQKSEKIKTVKLEGDTRFRQLATE
GVKEQMP-LHALNADFAV-LPMLVTPAVGSVAADAGVLGARVPVDASL-
RELQKSQKGSALPISAIRILDMRAARAMIEEDIMVAGKVAA-
LLQITALETIAELVLRNDSEAILPWRLSVAERLEADTKRAGGYAARPFSSKPSLCDTQAHATVADPIQEI
AAGYVATADRFVFPVEGIAIQIVLSNPHSRVGGAKRAGEGHLHEMAKELLAKAVLMLRNEPDTNTEG
LINYITTNQPTDLLFEDCLDVIIDMRRMSVV--

IQLNTV-
HVVIPGADDLAERKIMAAAAPSGLIQTERVDDRTGRERTLMWMYGSMEELWLLSVRYADWVTDTPI
GAIILHVYAQVINRAFSLKRLQLDGWPKEASRWWNTWQLWLNKDGFFGYEAGFRA-
KQVLKAALADYEMKDVHGVARMLSRQELRAGYRIRQQVPFARLNPRTDILLQAQTRLSVIRHALAVV
AILVLRDVATEFT-
VVAMEDVAMGHVECVLADSFAFTAKDADIGPAGLRTPDTIYGDGTVLKDAVLGKVDLANTVADFRY
RMLIDGGRALGVSWADL-NMLYPRIWVPPAQAALKNLTPITFFLSHPELDLLGA-
DIFKGISKARAEELMASALIEEILEGVARFADPEATTHQSHKERHFKYYGEEVDPVHLAYILRMFALVAT
QVTVSDRLLKDRYVLNLTV-RGFTSLLWISIQLSAKSPSATVITVGTIAGLGALVLFIILL-
RESVLLETLYFRYVY--MRGYSAAKDVADADPEHFGPLAAASRDMEWIEIVPAFFYYFATAG-
FEPFNTAVAKEFFGLFEERLRLVDVLAVPPDRVEALVEFVALAGTLPTSADTLPSVIATDLCEPNAAAVL
EAERDWNLHRTMGITLAFFKAQISVKGWFDKGLHKDVAETGLSEAGGFTTPFHAGKNAKRCVPVVH
RVIDLGELEAGLIEIDSAVLVADEEQTLADQPTTRDNSVGAIGGVILAGTMNMGSSLDQVERGGNIIIEL
EEEQYHAVNGKKADGKITDKILMVLSRAYFPVIERVASGTALDRGVFGLLIQAPDDADNLDHGRALVI
PASTVLAETTPLYAESEIEKIAWIAPMHLLGTLAAVLGFEPDHYSFISMMIEASLDITETAEVLDDYHVLE
KGARPPSFRKILIKGLHIRLLAAVGTILVVGSAGRSEITAYINGQAVPITMRKATFLPAFAASSCKTVDIFS
TMLRPPERSTIEMLILETVTEL-
LEVTRLENDKLERILILLPGLDFAVILELAAAAKDFSQIVTIDAAITAILSVAAEKVVLREMLLTKAEIAVTAL
ALAMALLLMLTADAGLTQKEAARDTQLTELIQGGVVLQVRVEDRMVLAATADKVRLSTDEGTTSAFA
SLNLATVSDGFYVLSSAVLQLSSDLMMIVAVSRVLADKETKNALVVRHPEQVKTQPTYAEGSPGMVL
QTLTACIPPLAIKPEEVELLWTLVAALWQVKSHIASAMRPRQERVLILILALGLIVVLLQSEGGGMGGG-
MGAAAKALAAFSMLTFKASVEDEVRKVTRRVMDLNSRLGRPEEAVAPEFRGEELEQNKYEAENVEVD
CIETIRRFVDIPFLVSTRTQHNESEDRGSRMVLDVTRVRFIDL-
VTKRSEIILVERAETDGVIARQMAKDLQALHPEAAALGKIAALQHFISVNDGHISNYVTAEINSVLGML
LLVLFIQQVRVSKVVWPTRREVTMVFILAALAVFFLVIGLSVNLVFMVGGISVAQITTLVLGEGVMLVR
QVSTFPSES-VTILQNTYADLICAGRVTKADIEKSLHIVVGARVARGTWYVIYHYEI-
QAQGPAGIMQRAFRQATLSQSLIDGRMEVKVKVVTPDRYA-
THEIKGGQHRKGTRYNRVKVKHNIPVDLIARIKMAFAPPNLVLVKTFGAVVQKRFAQQLTHMRHGA
VSIGPMEVSSPYIRSKKARHAPGDSIVTIKPDNIVRSVAVVPELIWARENEDQSAVHDKYTKTTHKIAAK
RSRAVDKAQYDKRNQVHLSFIGLAVKVLPEQAIVHATAGTLMPTSSEAVTEALKGVAATIRLDPMEKA
LIAYIKRMRPQLNLGGQTDKKIVKCLNNNLDDVEYGKLGRRKIKPITIKSVYDVEHKRATELVKLMPYKD
DVEGRKSQFAEK--
ALFEPLQAFMMLYGFIARAVYALYLRVIRRTILLAKTKIIVNASTVLSAIKIIGVSIRLRHKSTNIADTKHHIK
TIEKRARMTLAGGPSVKLHLSDGRVGTKGGRIKGYMPYKNYNIFDGLASGRRAKVKELAKLKKVDKSIS
VDGIRTRQQGGRIPKADLSNAYANTRVGVDIVGENEQEESNPRANAEMGQIFAAIDARVYSLVGALI
GHALEA--ALCAILGIGRAHVLLAVKLKVNAGIELHSAA-VAQAVAAA-----------------------
----
LKKTKKEAYMSLTCFTGNGDKIKIVEASQTQAFEIVATRTAERFGIKAKGTYGKERAMMQIVPTKPVKV
WVPVFVNDDELLAPIRAFAETNGDGVTVVKLTKDIKVTMFEKGVASLAVSGKATSVVYTVLEKVTELM
KDIIYEQRAKAHYGRGRLRSHVVTHTKSQRHLSMIRRTVVADPSMEAARLTDGEVSVITGATMAFVM
SEQDAV--
GDVDMHFHEEPRREAELGIAGPETFLSTEQPRLLALLFLQEYLIHIVDIGTVGNFSDAATIYDILRAMASK
LDRKLSADIAKTRMCLADDVADMGVTELAARRESAGDPILSAYWINGVTATADIILLVANLESKLTPEN
IDCAVRLFYQRITLQAKERGGGVEVALVPAQNQIEAAEGTVIYTIKGKTPSSTQMKLTIEAISRSIVKSLA

VQLFQVELFVNDTWLGTLLRAAGDVVYVDARDGKEGVDHVVERDTLLEFAPQKDIVARKIRIYTPAAP
SAVVIWDPAIEREDKADAQGTILMIYGPDLQDISCFTDIGAEAKIESLASWDEKEKYVVHRDDIPEDITT
LVGKSSKVNKEFGIPDVGVCAPAKPGVTESIDRGEPKAGSCGEVANLPYPQESLVNRVSSANVQKNIS
MVLGGHPVGEMGISRGIERHLETNMCEIFVDKADVDVALDFYRLNLTAMRVGLDNPNAKVEIDGRES
RKLIAVQDGDADLQRHPKLSEEAGALRKAETKAKLPDDTLILDGVAHGDVSFTVTDLPEGRV-
VTPVNNNVKAQLEIDKVLLKTFHVVTLSFSNNKGDDHLIKLRLNALKKSTRHYEMLLKIRDTECATSPH
AISVGKILKIDSLLQTICTAHYTEIEPLMAQDLAALGVMGDQGLLHVVPRLMILAVLVLYRLEAEGYVPK
W-TQAVAIITDQTMGLAMRQRGWSF-CISEVVGNTFFARTKDDDTEQRLHARNPI-VLL-
YGKSGMV-
ISPSGLQLDSTAPDTQGHGNDNALILSRENLQDAMSYYVLHVLLVFVDLVILAYGSGDVPLKFLVANQ
AVTGRTNPQLVCLARRTFPLDLQRDATFVLVFEDATMKNLSHKKMHMNLVLIKSKGWVANVSTKILI
SDQAIASYAEDAGAHTQPSESALAAITSRVMAPIVLVVSQKPTAPSKTLFLEALNLLDEVTATVLNKPK
RYFVFEADLISILQPIDAAVLKGMQRPQPYRMRRAVVAHAIRLAIGLRAAGVLAWLEAGIINFSRAVTF
AG-FYSTIDC-GGGAIT-QALAFRVRNHKVNTKRARPYKGHTKFLHKADSDKSLIADGVDTIA
>GCA_000155735
AVIKPVIQTIYAPVLMILQEQVATVSGAGSPSLRAKTVYAKSGLQRKMATKWVAAPFYY-
GVVKMGDIVQLVSISVDSRAVCTVAQLVDQEVVVHGLVALEAVKKKIALEAIKGMTTEDPAKVTQYEE
NIVLKGYRDVDDRADLLPILLGLTLKIRFDPHEPLVHDLPIPTVFEVV-
GIMAQRGTIEVDDCGISTAVSDVDPIVFVSPIDLKAVAGKQMAFIDQETAVSDARTAIALEELALEASIL
VVGIKPLLIPI-FRDNGAT-MSFANRSRTLKSIVDLAVGSVTITRAR---EFVAEM-
MQQAKAAALAEKRGTAEEIRIVKRMQKSAGLADDPDLVILEDGAEGNAAWAGQIYDDPISGSSESDS
VGNIVHAANKRQNFQRSITFLRKAEISEKAAAPLMRGVKHNAKSRSSRKLDDAICVLNFPNVPEGIAIR
AQSGTPADTLLHDGAEGEEILLLIVRWHVTWKTAIFVILLPEAGNVVGLIELGGIAAVGKMDGLLNRHH
KLNGSLIEQKPIIEKMTLRLRAALKQKLGKDQVIIFVVRVTGDLVAETKIQMG--
NVGLVVAIQGDTIFVRRHRQTVIGDREATV-ILQGVAEGPADAAALLRIRVDHTLVKLTILLIPE-
PVLVESVTIREGVGKALQIIQLDVYLLRDMGTGEPTLAKSKSRELIRELLQVDPITGVRLAFGKIKERLSRG
MKLKDLD-----
LAILVGITEPRMENLPEGVCRNFIDAGPRAAVGMLFLLLDEAGRIYIIDGDLRELTKSIKSLIGLVDAATPK
ILSASYATTRLKIRYADCAVLVSDAELYAYAEDRKELMRMVPVYIIKAAARRQLVIFALSLKGGHRRTRA
ALEVLLPATILESQAEEGVVDHEDPAVLLDDGEIVGSEMMISRQAALDLKQILKVDVIVVFPEFQEKMV
VSLRFFCTEVAIFVIALITTVGEMLSREPEHAQEEGAGFQKRIRTHSVLFALLATFDRRNARCA-
APQLFLHLGVAAVRYGILYKLQPKKKQMPSEPDKDRIDGFELVHAILNDGTS-EDLEFGEAMALAVE-
DASINVSAGVLAMTGGMVDVEKVDSKRKVAVAATKWTIAPVPLVDVCLSAADQMRCLG-
ITAADLSAALGRKELWFLFLPGANNKELEEAKLDGAGNSTAESAADKQIDILIVKPVLVVIQDSNTAALI
SNDGVMEWGTMAYKKHYAFAARMRLHMRTID------
QMEPNKQKAEKIKTVKLEGDTRFRQLATEGVKEQMP-
IHALNPEFAVLRPVLVTPAVGQVAADEAVLGAEVPCDFSL-
RELQKSRRGNALPIAAMRLLGVRKARAMIDEGIMVAGKVAA-
LLKIGALETITHIVLRNDSEASLPWRLSVAERLEADTKRASGYAARPFNSKPSLCDTQAHANVADPIEIIA
VGYVATAERFVFPVEGIAIQLILASPHSRVGGDKRAGEGHLHEMAKELKAKAVLMVKGEPENDTEGLL
NYHTTGQPTDLLLTDCLDILIDMRRMSIV--
VQLNTV-
HVVVAGVRDVGAYEIMQAAAHSGLIQVEQIDDLTGRERTLMYMYGSMEELWLMSLRYADWVINTPI
GAIILHVYAQAINRAFSLTRLQIEGWPPEAQRWWNTWQLWLNKDGFFSYDAAFRALKAVLKADLAD

FDMSDTEGVARYLSRQEIRAGYRIRQQVTFARLTPRTDILLQSKTRFAVIRHALDMVAILVIREVAPEFT-
VVAQEDVAMGHVECVLADCFDFTEKNADIGPVGLKTPDNIFGDGTVLKDAVLGKIDLKNTVQDFRY
RFITDGGRDLGVEWVTLQNMLYARIWVPPAQAALKDLQPITFFLSHPEVDL---------
ISKARADQLMASVLIEEILEGIARFAGPEATTHQSNKAKHFKYYGEEVDPVHLAYILRIFALVATQVTISD
RLLKDRYVLNLQIFRSFTSLLWMSLQLANKGPSATIITLGTIAGLGALVLFIILL-RDSILVETLYYRYVY--
MLGYSAAKDVADADPEHLLILSHASRDIEWIEIIPSFFYYFK-----
KKTNTAVAKEFFGLFKDRLRIVDALCVPPDRVEALIGVVALAGTLATSADALPGIIAAGLTSPNAAGVLE
ADQDWNLHREMGITLAFYKAQSSVKGYFDKGLHKDVAAIALSEATGLTTPFHAGQTANKCAPAIHRII
DLGDMEASLIEIDSSVKVADSEQTLADKPTTRENSVGAVGGVILAGTMKMGANLDSVERGSNIIIELEE
QTYNGVNGKKNDGKMTNKVLMVLSAAFFVVLRQLASANALDKGAVDLLVDAPNDADNLDHGESL
TIPAPTVHSETTPLYAESEIERIAWVAPMHMLGTLPAVLEFEPDQYSFISMKINATLNIMQTAEVLDDY
NVLEKGARPSAFRVILIRGLHIRQLAGIGTIIVVGSAGRTEITAYIEG-
AVPITLNKKTFLPAFSASSCHAIDIFSTMLRPPERSTIMTMILETVTEL-
LEVTRFANDKLEHLRLLLPGLDLSEILPLLAAAKDFAEIVTISASITAILSVAAEAVVLREMLLTLAEIAVTAL
ALAMALLLMLTPDRGLTQKEAAREEQMKELIQGGVVLQVRAADKMVLASTADKVRLATDEGTTSAF
ASLNLATVSDGFYVMSSAVLQMSSDLMMIVAVARVLADKETKNALVVRHPEQVKTQPTYAEGSPG
MVLQTLTDHIPPLAIKPDDIELLWTLVAALWQVKAHLAQATRPHIERVLVLLLALTLIVVLLQSQGGGI
GGG-
ASSAAKVLAAFSLLTLKASVGEEVRKVARRVMDLNSRLGRPEEAVSPEFRGAELEQSKYEAENVELDCI
ETVRRFVDIPFLVSTRTQHNESEDRGARLVLDVTRIRLIDL-
VTKKSEIILAARAETDGVISREMAKDLQSLHPEAAALGKVAALQHYVAINAGHISNYVTANINSVLGML
LLVLFIQQVRVGKVVWPSRREVTMVLIMATVALFFVVIGL--
QLVFMLAGISWQALTTLVLGEGVMVVRQTSTFPQDS-
IGILQNTYADLICAGRVTKADIEASLHIVVGSRVARGTWYVIYHYEITQAQQKQGIMQRAFRQKTLSKS
LIDGRMEVKIKVVTSDRYA-
TYEIKGGQHRKGTRFNRVKVGHNIPIDVLARMKMAFGPPNLILVKTFGAVVQKRFAQQLTHMRHGA
VSIGPMEVASPYIKSKKARHAPGDSVVDLKPDNILRSVAVVPELIWAREKEDQGAVHDKYVKTTHKTA
AKRNRAVDKAQYDHRNQVELSFIGLAVKVLPDKAIVEATAGQLLPTATVTVTEGLRGVAATIRLDPME
KTLIAYIKRMRPQLNLAGKTDKKIVKCLNNNLDDIEYGKLGRRKIKPLTIKSVYDVEHERATELVKLLPYK
DDVESRKSQFAEKDEALFEPLKAFMMMFGFIARAIYALYLRVVRRTIVLVKTKIVVNAATVLSAIKIVGVS
VRLRHKSTNVAETNHTIKTIEKRARMTLAGAGKVKLNLHPGRVGMKGGRIKGFMPYKNFNIFDKLASG
RRAKVAELAKLKNVDKEISVDGIRVKQQGGRTPKAQLSNAVANTRVGVDIVGESEQEESNPRANAE
MGQIFATIDARIYHHVAALIGHALVAVLAVVAILGIGKAHALLAVE-KINAGIELHSAA-VNTAVAAA-
YLDSGGEV-
AIADVSEAYLGLISITSESLDVCRLRRRSISELKKTKKEAYMSLTCFGGKGDRVKIVEAVQTQFYEIVATRT
AERHGIKAKGTYGKERAMMQIVPTKPVKVWVPIFVSEDELLAPVRAFAETVGDGITVVKLIKDIKVTMF
EKAVASLAVAGKSSTVIYTVLEKVTELSKDIIYEQRAKAHYGRGRLRSHVVTHTKNRRHFSMMKRTVVA
SPSMNASRLTDGEVTVITGATMAFVMSEQDAV--
GDVDLHYHEEPRREAALGIAGPETFLATEQPRVLALVFLRAYLLHIIDVGTKGGFADAATIYDILRALTAK
LDRKLSADIAKTRMCLHDNVADMGVTELAARRESAGDPILSSYWINGVTALVDIILLVANLECDCAPE
NIHCAIRLFYQRVTLQAKERDGDVSVAVVAAKNQIEAAEGTVIYSIKGKTPSTTQMKLNIEGISRSIVKSL
AVQLFQVDLFLNDTWLGTLLRAAGDVAYVDAKGGEEAVDHVVERDTELEFAPQKDIVTRKIRIYTPAA
PSAVVIWDPAIEREDKADAQGTIIKIYGPNLQDISCFTDIGSEPKIESVKSWDEKEKYVVHRDDIPEDITT
LVGKSSKVNKEFGIPDVEVCAPSKPGVTESIDRGEPKAGSCGEVANMAYPQESLVNAVSSANVQKNIS

MVLGGHPVGEMGISRGIERHLETSMCEIFVDKDDIDVALDFSTIALTPLRVGLDVPNAKVQIDGKETRK
LMAVIDGDADLQRHPKVSEATGIVKTAGTKARLPDDALVYDGVQNGDIKFTITDLPRGRV-
VTPVNNNIKAQLKIDKVMLKTFHVVTLSFSNSKGEEHLIKLRLNALKKSTRHYEMLLKIRETECAESPHAI
SVGKILKIDALLQTICKAHYVEIEPLLADDLTALGVMGQQGLLHVVPRLMILAVLHLSRLGVALQLKNW
GLQAIAACTDQTMGLQERDRAWSF-CIAEVVNNTFFARTANDDTEQTLHARNPI-VLL-YGKFGMT-
ISPSGHRLDSTPISEMEEDNAGNVILSAVGLQDAMEYYVAKLLLVFVDEVILAFGSGDDPLRFLVAEQA
VTGRENPGMVCLTRRTFPLDLQRDATFVLVFEDSTMKNLSHKKMHMNLILIKSKGWVVNVSTTILISD
QAIASYAEDVAAHTQPSESALAAVTSRVMAPIVLVVSQKPTAPSKTLFLEALNLYDEVTATVQNKPKR
YYVFEGDVISVLNSIDAAVLLGMQRPQPEAMRRAVVAMAIRLAIALRASDIAARLEEGFINFSRAVSFIR
-HYAAHSEATHAPMGGTAVAFRVRNHKVKTKRARPYKGHTKFLHKADMDKSLIATGVDTLA
>GCA_000156135
AVIKPVEINKYAPVLMIRQQEVATVSGAGSPSLRAKTVYAKSGLQRKTMTKWVAKPIYF-
GVLKMGNIVQLVSISVDNRAVCTVAQVVEQEVVVHGLVALDAVKRKIALEEMGGMTTEDPAKVAEF
EAEIDLKRFRDADARADMLPVMLGLTLEVKFDPHEPVVHDLAVPVIYEVI-
GIMADKGGIEVDDCAISNAVSDVDPIAYVSPIDLEAVAGEQMAFIDQEAAVAAARETIALEELALEAGI
LVVGIDPLLLPV-FGDDADV-MSFAARSRTLGSIVALAVAYEELTRA-IDG-IASRM-
MAAAQAAALAEKRGTAEEIRIVKRMQRSSGLADDPELVILEDSAEGNSSWAGQIYDDPVSGSSESDS
AGNIVYAANKRQNFARSITFLRKVEISDKAAAPLMRGVKHNSKSRSARKLDDAIAGLNFPNVPPGIAIR
AEAGRTADRLLHDAAEGELILMIVVRWHVTWKTAVAIALVYQAEDLVGLIELGGIAAVGRPDGILNRH
HKLNGSLIEQRPIVEKLTLRLRASLKEKLGKDQVIIFVVKVQGDLVADATIQMG-
GPVGAVVAIQGELIFVRRHKQTVITDQEATV-VLKGVAEGPADAQALLRIRVTHTLVGLSVLLIPE-
PVLVESVQIREGVGKSLQIIQLDVYLLRDMGTGEETLAESRSRELIREVLAVDPITGVRLAYGKLKERMG
KKLGLKDLE---
GKLYQIDGDLRELPKSVKSLIGLVDTKAPKVLSAGYAEERLRIRFADCEVLGTDANLYPYKEERRELLRM
VPVYLILEAKRRQLIIFALSLKGGHRRTMMALGVMLAAAIIESQAEKGEVHHETPEILLDDAEVVGTSIM
LSRQASLDLKQINKVDVIVLFPEFQEKMVVSLKFFCTEVAIFVIALITTVGEMLSREPEHAQEEGAGFQK
RIRGHSMLFALAVVFDRRNCRDA-
LPDLFLALGLAEPRYGILYTNQPKKKQMPSEPDGDRVDGFELVHAVKEDGAS-
HELEMPEAMALAVE-
DATINLSAGVLAMNGGMVSIEKVDSKRKVALAATKWNISKLELADVHISGSAPTRSCG-
IEQAELSAALEAKKVWFLFLPGASNAELEEAKLDGAGASTADAADDKQIHILLVKPVLVVIQDSNSAGL
ISNGGLDEWGTMAYKKHYAFAARMRLHMRTID------
DMAPNKQKSEKVKTVKLEGDTRFRQLATDGVKEQMP-LHALNPDFAV-
LPLLVTPAVGSVAADENVLGAQVPVGASL-
RELQKSQKGSALPISAVRLLAMRAARGMIEEDIMVAGKVAA-
LLKIRALETVIAILLRNDTEATLPWRLSVAEKLEADTKRASGFAARPFKTKPSLCNTQAHAKIADPIQEIA
AGYVAAEERFVFPVEDIAIRLILSNPHSRVEEKKRGGEGHLHGMAKELLAKAVVMLRNEPGTNTEGLL
NYLTTNQPTDLLFEDCLDVIIDIRRMSVV---
VHLNTV-
HVVVAGVRSVGDYEIMSAKFPSGLVQVERVDDRTGREKTLMWMYGAMEELWLMSARWADWVPD
CAIGAIILHVYAQEINRAFSLKRLQLDGWPKEAARWWNTWQLWLNKDGFFGYEAAFRAVKQVLKAT
LFDFEMSNVEGVGRYQSKQELRVGYRIRQQVEFARLDPRTDILLQAKTRLSVIRHALEVVAILILRDVAP
EFTLVVAYQDVARGHVECVLAESFDFTEKNAHIGPVGLRTPASIFGDGTVLKEAVLGKIDLANTVAEFR
YLVILDGGRALGVEWISLRNMLYERIWLPPAQAALKNLLPICFFLQHPELDLAWA-

ALFEGISKARADKLMAGALVEEILEGVARFADPEATTHQSKKAKHFKYYGEEVDPVHLAYILDLFALVST
QVVVSDRLLKDRYVLNLKVFRGFTALWWMALQLSPKGPSATVITVGTIAGLGALVLFIILL-
RDSVLVETLYYRYVY--MKGYEAAKDVQDADPEHLGQLAAASRDIEWIEIIPAFFYYFDTAT-
FEPFNTAVAKEFFGLFEERLRIIDVLAVPPDRVAALVEFVALAGTLATSAEALVGVIACGLCEPNAAEVLE
AERDWNLHRSMGITLAFFEARESVKGYFDKGLHKDVAEIGLKEAGALTTHFRCDVDAKQIAPAVHRIL
DLGEIEAALIEIDSAVVVADEEQTLADKPTTRDKSVGAIGGVVLAGTMNMGANLDSVERGGNIIIELEE
EHYHAVNGKKADGKLTDKVLMVLSVAYFPVIRRFASANALDRGVFGLLIDAPEDADNLDHGRVMTIP
APSVLAETTPLYAESEIDKIAWIAPLHLLGTLPAVL-
FEPDFQGFISMMIDASLEIMETAEVLDDYHVLERGSRPPKFRVILIKGLHIRLLAAAGTILVVGSAGRTEV
TAYIQGQAVPISLSKNTFLPAFSPSSCKAVDIFSTLLRPPERSAVEMLILETVAEL-
LEVTRGSNDKLERLLVLLPGLDMAELLGLLAAAQDFADIVTL--------------------------------
----
TGDEGLTQKEQAREAQMKELIQAGAVLQIHAADAMVLASTADKVRLDTDEGTTSAFASLNLATVSD
GFYVLSSAVLQMSSDLMMIVAVARVLADKESKNALVVRHPEQVKTQPTYAEGSPGMVLQTLTGCIPP
LAIKPEEVELLWTLVAALWRVKAHLASAARPHTERVLILLLALGLIVVLLQSEGGGMGGG-
IGAAAKLLAAFSILTMKAAVGDEVRKVTRRVMDLSSRLGRPEETVNPEFKGEELDQTKYEAENVEVDC
VETVRRFVDIPFLVSVRTQHNESEDRGARLVLDVSRVRFIDL-
VTKKSDVILVERVETDGIIARQMAKDMQSLHDEAAALGKVSQLQHFIALGDAHISNYVTAEAHSVLG
MLLLVLFIQQVRVSKVVWPTRREVTMVFIMAALALFFLVIGLGLNIVFMVGGISPLQITTLVLGEGVMA
VRQTSTFPAES-
VTILQNTYADLICAGRVTKADIEKSLHIVVGARVARGTWYVIYHYEITQAQSPAGIMQRAFRQATLSQS
LIDGRMEVKIKVVTSDRYA-
TYEIKGGQHRKGTRYNRVKVGHNIPVDVLARIKMAFAPPNLVLVKTFGAVVQKRFAQPLTHMRHGA
VTIGPMEVSSPYIQAKKARHAPGDSVVDIKPDNIVRSVAVVPELIWARENEDQSAVHEKYVKTTHKIA
AKRNRAVDKAQYDKRNQVHLSFIGLAVKVLPEQAIVHATAGTLLPTESVSVTENLKGVAATIRLDPME
KALIAYIKRMRPQLNLQGKTDNKIVKCLNNNLDDVEWGKLGRRKIKPITIKSVYDVEHERATELVKLMP
YKDDVEGRKSQFAEK--
ALFEPLKAFMMLFGFIARAVYALYLRVIRRTILLVKTKIVVNAETVLSAIKIIGVSIRLRHKSTNIADTKHHIK
TIEARARMTLAGAPAVKLHLRPGRVGKKGGRIKGYMPYKNYNIFDGLASGRRAKVKELAKLKKVDKTI
AVEGIRTRQ-
GGRLSKADLSNALSNTRVDVEVVGENVQEASNPRANAEMGQIFAAIDARVYSLVAALIGHALEALLA
LCAILGIGRAHALLAV-LKMNAGIGMHSAA-VAAAVAAA-
YSQPGGEATALWDVSEGDLGALAISEGSLDVCRLRRRQIG-
LKKTKKEAYMSLTCFSGKGDKVKIIEATQTQAYEIVATRTAERHGIHAKGTYGKERAMMQITPTKPVKV
WVPVFVSDEELLAPVRAFAETNGDGVDVVKLAKDVKITMFEKGVASLAVSGKNTTVVYTVLEKVTEL
MKDIIYEQRAKAHYGRGRLRSHVVTHTKQKRHLSMMKRTVVADPSMDAARLSDGEVSVVTGATMA
FVMSEQDAV--
GDVDYHFHEEPRREAQLGIAGPETFLSTEQPRLLAMLFLREYLIHIVDIGTVGGFADAATIYDILRAMQN
KLDRKLSADIAKVRMCLHDDVEGMGVKAEAPRRDQAGDAILSAYWINGVTANNDIILLVANLECKTA
PENINCAVRLFYQRITLQAKERDGDVSVKLVPAQNQIEAAEGTVIYTIKGNNPSTVQMKLNIEGISRSIV
KSLALQLFQVDVFLNDTWIATLLRAAQDVVFVDAREGKEAVDHVIERDTELEMAPQKDIVARKIRVYT
PAAPSAVVIWDPAIEREDKADAQGTILMIYGPDLQDISCFTDIGSEPKIESLAAWDEKEKYVVHRDDIPE
DI-
TLVGKSSKVNKEFGIPDVAVCAPAKPGVTESIDRQEPKAGTCGEVANLPYPQESLVNRVSSANVQKNI

SMVLGGHPVGEMGISRGVERHLETNMCEIFVDKPDIDVALDFYKIALTSLRVGLDKPNAKVEIDGKESR
KLMAVKDGDATLQRHPKVSEESG--RKAGEKAKLPDDALIYDGVQHGDIKFTATDLPDGRV-
VTPVIMSVKAQLEIDKVMLKSFHVVTLSFSNDKAEEHLIKLRLNALKKSTRHYEMLLKIRDTECAQSPHA
IPVGKILKIDALLQTICKAHYTAIEPLLADDLTALGVMGDQGLLHVVPRLMIIALLHLCELGAAAAIPHW
RLQQAAILTDQTMGLAVRERRWSF-AISEVTGNTFFARTKDDDTEQRLHARNPI-VIL-YGKEGML-
ISPSGLRLASTAPSDKGHGNDEKLILSPERLQDAMYYYVLKVLLVFVDEIILAYGSGEGPLKFLVAHQAV
TGRDNPQLVCLTRRTFPLDLQRDATFVLVLADTTMKNLSHKKMHMNLVLVKPKSWVENVSTKILISD
QAIASYAEDAGAHTQPSESALAVITSRVIAPIVLVVSQKPAAPSKTLFLEALNLYDEITATVQKRPKRYFIF
EGDVVSVLGTLDAAVILGMQRPQPDRLRRAVTAHAIRLAIALRGSGVLASLEGGAINFSRAVDFAA-
TY-GLEA-----------DFRVRNHKTSTKRDRPYKGHTKFLHKAEAEKALIAEGVDTIA
>GCA_000156255
AVIKPVSLNAYAPVLMIRQQQVATVSGAGSPSLRAKTVFRKSGLQRKTLTKWVAKKIYF-
GVLKMGNIVQLVSISVDNRAVCTVAQVVEQEVVVHGLVALAAVKRKIALEEMDGMTTEDPAKVAEF
EVELDLKRFRDADARADMLPVMLGLTLEIKFDPHEPVVHDLPIPVIFEVI-
GIMADKGGIEVDDCAISNAVSDVDPIAYVSPIDLEAVAGTEMAFIDQEAAVAAARETIALEELALEAGIL
VVGIDPLLLPV-FGEDGGV-MSFAARSRTLGSIVALAVAAEELTRA-IDE-IASDM-
MGAAQAAALAEKRGTAEEIRIVKRMQRSSGLADDAELVILEDAAEGNSSWAGQIYDDPVSGSSESDS
AGNIVHAANKRQNFARSITFLRKAEISDKAAAPLMRGVKHNSKSRTARKVDDAIAVLNFPNVPPGIAI
RAEAGKPADRLLHDATEGEEILMIVVRWHVTWKTAVAIVLVLTAGNIVGLMDLGGIAAVGKPDGALN
RHHKLNGSLIEQRPIIEKMTLRLRASLKEKLGKDQIIIFVVKVQGDLVADATIQMG-
GPVGAVVAVQGEVIFVRRHKQTVITDQDATV-VLKGVAEGPADAQALLRIRVTHTLVELSVLLIPE-
PVLVDSVSIREGVGKALQIIQLDVYLLRDMGTGEETLAESKSRELIREIFTIDPITGVRLAYGKLKERMGKK
LGLKELETI---
RSILVAVTEPRMENLPGGVCRTFITAGPRAAVGMLFLLLDDGGKLYSIDGDMRELPKSVKSLIGLVDAK
APKVLSAGYAEERLRIRFADCEVLASEADLYAYKEERRELLRMVPVYIINEAKRRQLIMFALSLKGGHRR
TMMALGVMIAAAIIESQAEKGKVHHDAPAVLLDDAEVVGTAIMISRQSALDLKQLLKVDIIILFPEFQE
KMVVSLKFFCTEVAIFVIALIVTVGEMLSREPEHAQESGAGYQKRIRGHSVLFALSAVFDRTNLRDA-
TPDVFLALGLAQPRYGVLYTNQPKKKQMPSEPDGDRVDGFDLVHAVKEDESS-
QELEMPEAMALAVE-
DATINVSAGVLAMEGGMVAVEKVDSKRKVALAATKWSIAKLEVVDVTISGAAPVRCCG-
IEMAGLSAALESKKV-
FLFLPGASNAELEEEKLDGAGASTAEAAADKQISILLVKPVLVVIQDSNSAGLISNGGLDEWGTMAYKK
HYSFAARMRLHMRTIDAIEVGVQLEPNKQKSEKVKTVKLEGDTRFRQLAIEGVKEQMP-
LHALNPDFAV-LPLLVTPAVGSVAADERVLGAEVPVDFSL-
RELQKSQRGSALPITAIRLLEMRAARAMIEEDIMVAGKVAA-
LFKVRALETIVALLLRNDTEATLPWRLSVAERLEADTKRSSGFAARPFKSKPSLCKTQAHAKIADPIQEIA
AGYVATEERFVFPVEGIDIQLILANPHSRVGTAKRAGEGHLHEMAKELLGKAVVMLRNEPDTNTEGLL
NYLTTNQPTDLLFEDCLDVIIDIRRMSVV--
VHLNTI-
HFVIPGARDVAAFTIMSAKFDSGLIQVEQVDERTGRERTLMWMYGAMEELWLMSVKWADWVKDC
DIGAIILHVYAQTINRAFSLRRLQLDGWPKEAARWWNTWQLWLNKDGFYGYEAAFRA-
KQILKATLADFEMKDVEDVGRMLSKQEIRAGYRIREQVEFARLDPRTDILLQAKTRLSIIRHALTVVAILIL
RDVAPEFTLVVAYQDVARGHVECVLAESFDFTEKDAHIGPVGLRTPAGIYGDGTVFKEAVLGKIDLAN
TVAEFRYLILVDGGRELGVCWISLTNLLYPRIWLPPAQADLKDLLPICFFLQHPELDLAWG-VLFE-

ISKARADRLMAGALVEEILEGIARFADPEATTHQSKKAKHFKYYGEEVDPVHLAYILEIFALVSIQVVVSD
RLLKDRYVLNLKIFRALTALWWLSLQLAAKGPSATIITVGTIAGLGALVLFIILLMRDSVLVETLFFRYVY-
-MLGYEAAKDVQDADPEHLGALTAASRDIEWIEIIPAFFYYFDTAT-
FEPFNTAVAREFFGLFKERLRIVDVLAVPPDRVTELVDFVALAGTLSTSAQAAVGVIACGLCEPNAAAV
LEAERDWNLHRSMGITLAFFEARKSVKGYFDKGLHKEVAEIGLREAGALTTHFLCAVEAKQIVPAVHR
VLDLGEIEASLIEIDSAVVVADEEQTLADKPTTRDKSVGAVGGVVLAGTMNMGGNLDSVERAGNIIIEL
EEEQYSAVNGKKADGKLTDKVLMVLSAAYFPVITRFSSANALDRGVFGLLIAAPSDADNLDHGRTMTI
PAPTILTETTPLYAEADIDRIAWIAPLHMLGTLPAVLSFEPDHYSFISMMIDASLEIMETAEVL-
DYHVLEKGARPPAFRVILIKGLHIRLLAAIGTILVVGSAGRAEVTAYIQGQAVPISLRKHTFLPAFSPSSCK
VVDIFSTLLRPPERSSVEMLILETVAEL-
LEVTRGTNEKLERMLILLPGLDLASLLDLLAAAEDFATIVTLGAAITAILSVAAEAVVLREMLLTKAEIAITA
LAMAMALLLMLTGDEGLTQKETAREAQMKELIQAGAVLQIHADDAMVLASTADKVRLDTDEGTTSA
FASLNLATVSDGFYVLSSAVLQLSSDLMMIVSVARVLADKESKNALVVRHPEQVKTQPTYAEGSPGM
VLQTLTDCIPPLAVKPEEVELLWTLVAALWRVKAHLANAARPHNERVLILLLALGLIVVLLQSEGGGMG
GG-
ASAAAKILAAFSILTLKASVGDEVRKVTRRVMDLSSRLGRPEEAVNPEFKGEELDQTKYEAENVEVDCM
ETVRRFVDIPFLVSVRTQHNESEDRGARLVLDVTRVRFIDL-
VTKKSDVILVERVETDGIIAREMAKDMQSLHPEAAALGKVSQLQHFIALGDAHISNYVTAQTHSVLG
MLLLVLFIQQVRVSKVVWPTRREVTMVFVMAALAVFFIVIGLGINLVFMVGGISPLQITTLVLGEGVMA
VRQVSTFPAES------
NTYADLICAGRVTKADIESSLHIVVGARVARGTWYVIYHYEITQAQAPAGIMQRAFRQATLSQSLIDG
RMEVKIKVVTSDRYA-
TYEIKGGQHRKGTRYNRVKVGHNIPVELLARIKMAFGPPNLVLVKTFGAVVQKRFAQQLTHMRHGA
VTIGPMEVSSPYIKSKKARHAPGDSTVDIKPDNVVRSVAVVPELIWARENEDQSAVHEKYVKTTHKIA
AKRNRAVDKAQYDKRNQVHLSFIGLAVKVLPEQAIVHATAGTLLPTESVAVTENLKGVAATIRLDPME
KSLIAYIKRMRPQLNLGGKTDNKVVKCLNNNLDDVEYGKLGRRKIKPITIKSVYDVEHTQATELVKLMP
YKDDVEGRKSQFAEK--
ALFEPLKAFMMIFGFIARAVYALSLRVVRRTILLVKTKIVVNAETVLSAIKIIGVSIRLRHKSTNVADTKHHI
KTIEARARMTLAGAPAVKLHLRPGRVGKKGGRIKGYMPYKNYNIFDGLASGRRAKVKDLAKLKKVDKT
IAVEGIRTRQQGGRLPKAQLSNALANTRVGVDIVGENVQEASNPRANAEMGQIFAAIDARVYSLVAA
LIGHALEAVLAVCAILGIGRAHALLAVY-KMNAGIELHSAA-VAQAVAAA-
YSQPAGEATAIADVSEAYLQALAVTDQSLDVCRLRRRQLGELKKTKKEAYMNLTCFSGKGDKVKIVEA
TQTQAYEIVATRTAERHGISAKGTYGKERAMMQITPTKPVKVWVPVFVNDDELLAPVRAFAETNGDG
VDVVKLSNDIKVTMFEKGVASLAISGKATTVVYTVLEKVTELSKDIIYEQRAKAHYGRGRLRSHVVTHTK
QKRHLSMMKRTVVADPSMDAARLTDGEVSVVTGATMAFVMSEQDAV--
GDVDMHFHEEPRREAELGIAGPETFLSTEQPRLLALLFLREYLIHIVDIDTTGGFADAATIYDILRAMQN
KLDRKLSADIAKVRMCLSDDVADMGVTDVAPRRDTAGDTILSAYWINGVTANTDIILLVANLECKTAP
ENIDCAVRLFYQRVTLQAKERGGDVSVELVPAKNQIEAAEGTVIYTIKGNNPSTVQMKLNIDAISRSIV
KSLAVQLFQVDVFLSDTWIGTLLRAAEKVVFVDAREGKEAVDHVVERDTALEMAPQKDIVARKIRIFTP
AAPSAVVIWDPAIEREDKADAQGTILMIYGPDLQDISCFTDIGAEPKIESLAAWDEKEKYVVHRDDIPE
DI-
TLVGKSSKVNKEFGIPDVAVCAPAKPGVTESIDRGEPKAGSCGEVANLPYPQESLVNRVSSANVQKNI
SMVLGGHPVGEMGISRGVERHLETNMCEIFVDKDDIDVALDFYKITLTPLRVGLDKPNAKVQIDGKES
RKLMAVKDGDADLQRHPKVSEESGVLRKAGEKAKLPDDTLMYDGVQHGDISFTATDLPEGRV-

VTPVFNAVKAQLEIDKVLLKTFHVVTLSFSNDKAEEHLVKLRLNALKKSTRHYEMLLKIRDTECAQSPH
AIPVGKILKIDALLQTICKAHYTAIEPLLAEDLTALGVMGDQGLLHVVPRLMILAVLHLCGLGAMMELSE
WPLQENALITDQTMGLPVRQRAWSF-AISEVTGNTFFARTKDDDTEERLHARNPI-VIL-YGKAGML-
ISPSGLKLASTDPSDDDHGNGDQLILSHERLQDSMSYHILQILLVFVDEVILAYGSGDEPLKFLVANQA
VTGRANPQLVCLTRRTFPLDLQRDAVFVLVLEDTTMKNLSHKKMHMNLVLIKSKGWVENVSTKILISD
QAIASYAEDAGAHTQPSESALAIITSRVIAPIVLVVSQKPTAPSKTLFLEALNLYDEITATVLKRPKRYFIFE
GDVVSILETLDAAVILGMQRPQPDRLRRAVVARAIRLAIAIRGSGILAELEGNAINFSRAVDFSE-TY-
GLEA-VGLAID-TAL---
>GCA_000156335
AVIKPVIQNEYAPVLMIAQTQVATVSGAGSPSLRAKTVYAKSALSRKTMTKWVAKPIYY-
GVLKMGNIVQLVSISVDNRSVCTVAQLVEQEVVVHGLVALAAVKRKIALDEIDGMTTEDPARVAQF-
--IPVIFEVV-
GIMADRGGIEVDELAVSQAVSDVDPIEYVSPIDLAAVAGTEMAFIDQEAAVAAARETIALD-
LALEASILVVGIKPLLLPV-FGDDADV-MSFANRSRTLRSIVNLAVAFPEVKRA----AFAADM-
MREAQAAALAEKRGTAEEVRIVKRMQRSAGLADDPELVILEDAAEGNSNWAGQIYDDPISGSSESDS
AGNIVF-
ANKRQNFQRSITFLRKVEISDKSAAPLMRGVKHNSKSRASRKIDDAIADLNFPNVPEGIAIKAAAGKST
DTLLHDATEGEEILLLVVRWHVTWKTAIIIVLIA-
AGDVVGMIELGGIAAVGKPDGLLNRHHKLNGSLIEQRPIIEKMTLRLRASLKQKLGADQVIIFVVKVQG
DLVADATVQMG-
GPVGSVVAVQGDVIFVRRHKQTVIGDQETTVEILKGVAQGPAEAEALLRIRVTHTLVKISVLLIPQ-
PVLVEAVTIREGVGKALQIIQLDVYLLRDMGTGEETLAESMSRETIREVLAVDPITGVRLAFGELKERQSK
KLVLKDLET----
LSILLAVTEPRMEDLPDGVCRTFVTAGPRAAVGMIFLILDDTGKIYPIDGDLRELQKSVKSLIGLVDAKVP
KVLSAGYATTRLKIRFADCEVLNAEADLYAYKEERKQLLRMVPVYIILEAKRRQLVVFLLSLRGGHRRAM
AAIGVMLAAAILESQAENGEVNHTQPAILLEDAEIVGTAIMVTRQSALQVKDRTKVDVIVVFPEFQEK
MVVSLRFFCTEVAIFVIALITTVGEMLSREPEHAQEEGAGFGKRIRSHSMLFALRVVFERDNLRDALGP
DLFVGMGLAPPRYGIVYTNQPKKKQMPTEPDSDRVDGFDLVHAIKEDKASNHELEMPEAMALAVEA
DAPINVSAGVLAMTGGMVDVEKVDSKRKMSLAAT--------MQDLHLSAASQFRCLG-
IELADLSAALGAKAE--
LYLPGASNEELEEDKLNGAGASTADVAADRQRNILLVIPVLVVIQDSNTAGLIANDGLDEWGTMAYK
KHYAFAARMRLHMRTIDSIEVGVEMEPNKQKAEKIKTVKLDGDTRFRQLATEGVKEQMPVLHALNPD
FAV-LPLLVTPAVGSVAADENVLGARVPVDASL-
RELQKSQKGNALPISAIRLLEMRAARGMVDEDIMVAGKVAA-
LLKISALETVIDVILRNDSEAVLPWRLSVAEKLEADTKRASGYAARPFKSKPSLCDTQAHAKVADPIQEI
AASYVAKEERFVFPQEGIAIQIVLNNPHSRVDSEKRAGEGHLHEMAKELMAKAVIMIRNDPDTNSEGL
LNYITTNQPTDLLFEDCLDVIIDMRRMSVV---
VHLNTV-
HVVVEGVRDVGNYEIMAAQAPSGLIQTERVDDRTGRERTIMWMYGAMEDLWLLSIRYADWVVDC
AIGAIILHVYAQQINRAFSLRRLQLDGWPPEASRWWNTWQLWLNKDGFFSYDAAFKAVMQVLKAA
LADFEMKDVHGVGRMLSRQEVRAGYRIRQQVDFSRLDPRSDILLQAKTRLSVIRHALEVVAILILREVA
TEFTL--
DGTVLNDAVLGKIDLSNTVADFRYLMLIDGGRQLGVDWLTMQNMLYPRIWVPPAQAALKNLTPICF
FLSHPELGQLID-

SSFGGISKARAAQLMAGALIEEILEGVARFADPEATIHQTHKEKHFKYYGEEVDPVHLAYILDMFALVA
TQVTVSDRLLKDRYVLNLRVFRGFTSLLWMSAQLAEKGPSATVITVGTIAGLGALVLFIILL-
RESVLIETLFWRYVY--
MKGYQAAKDVADADPEHFLALASASRDVEWIEIVPAFFYYFSTATSRLEKNTAVAREFFGLFKERLRVIE
VLVVPPDRVEALVEFVALAGTLTTSAEALPGVIAFGLCEPNAAGVLEAERDWNLHRTMGITLAFFKAQ
VSVKGWFDKGLHKNVAETGLREAGGFTTPFHAGKDAKRCVPVVHRVIDLGELEAGLIEIDSAVVVADS
EQTLADQPTTRDNSVNAIGGVVLAGTMNMGASLDTVERSGNIIIELEESQYHAVNGKKADGKMTNA
ILMVLSRAYFPVIAYVASANALDRGTFNLLIQAPDDADNLDHGREMVIPAATVHAETTPLYAESDIDKI
AWIAPMHMLGTLAAVLVFEPDQYSFISMMIEASLDITETAEVLDDYHVLEKGARPPAFRKILIRGLHIRL
LAAVGTILVVGSAGRTEITAYIQGRAVPITMRKSTFLPAFAASSCKTIDIFSTMLRPPERSTIMMLILETVT
EL-
LEVTRGENDKLERILILLPGLDLAVILGLVAAAKDFAQIVTVDASITAILSVAAEAVVLREMLLTKAEIAVT
ALAMAMALLLMLTADKGLTQKEASREHQMKELIQGGAVLQVRADDAMVLAATADKVRLSTDEGTT
SAFASLNLATVSDGFYVMSSAVLQLSSDLMMIVAVSRVLADKETKNALVVRHPEQVKTQPTYAEGSP
GMVLQTLTACIPPLAIKPEDVELLWTLVAALWRVKAHLASATRPRIERVLVLILALGLIVVLLQSEGGGM
GGG-
VGAAAKVLALFSILTIKATVEDEVRKVTRRVMDLNSRLGRPEEAVAPEFRGEELEQNKFEAANVEVDC
METVRRFVDIPFLVSTRTQHNESEDRGSRMVLDVTRVRFIDL-
VTKRSEIILVERAETNGIIARQMAKDLQALHDEAAALGKIASLQHFISLNDGHISNYITAQVNSVLGMLL
LVLFIQQVRVSKIVWPTRREVTMVFILAALAVFFVVIGLYINLVFMVGGISPLQITTLVLGEGVMVVRQV
STFPAEA-
VTILQNTYADLICAGRVTKADIEKSLHIVVGARVARGTWYVIYHYEISQAQGPAGIMQRAFRQATLSKS
LIDGRMEVKVKVVTSDRYA-
THEIKGGQHRKGTRYNRVKVGHNIPVDLLPRMKMAFAPPNLVLVKTFGAVVQKRFAQQLTHMRHG
AVTIGPMEVSSPYIRAKKARHAPGDSVVTIKPDNIVRSVAVVPELIWARENEDQSAVHDKYTKVTHKIA
AKRNRAVDKAQYDKRNQVHLSFIGLAVKVLPEQA--

RMRPQLNLAGKTDKKIVKCLNNNLDDVEYGKLGRRKIKPITIKSVYDVEHTRATELVKLMPYKDDVEG
RKSQFAEK--ALFEPLSAFMMMFGFIARAIYALYLRVIRRTIVLVKTKIVVNAA----
AIKIVGVSIRLRHKSTNVAETKHHIKTIDARARMTLAGSPSVKLHLSPGRVGTKGGRIKGYMPYKNYNIF
DKLSSGRRAKVKELAKLKKVDKDITVAGIRTRQQGGRIPKADLSNSLANTRVGVDIVGENEQEESNPR
ANAEMGQIFAAIDARVYSHVGALIGHALAAVLAVCAIQGIGKAHALLAVKLKVNAGIEFHSAA-
VAVAVAAA-YAQQAGEA-ILADVSEPNLRALAVTEASVDVIR--RRKLG-----------------------
----
RHGIKAKGTFGKERAMMQILPTKPVKVWVPVFVNEDELLAPIRAFAETNGDGVTVVKLAKDIKVTMFE
KGVASLAVTGKATTVVYTVLEKVTELMKDIIYEQRAKAHYGRGRLRSHVVTHTKSKRHLSMMRRTVVA
APRMDAARLTDGETSVITGATMAFVMSEQDAV--
GDVDMHFHEEPQREAQLGIAGPETFLATEQPRLLALLFLREYLLHIVDIETQGGFADAATIYDILRAMQ
NKLDRKLSADIAKTRMCLHDDVADMGVQEVAARRDSAGDAILSSYWINGVTANADIILLVANLECKV
VPENINCAIRLFYQRITLQAKERGGDVSVELVKAQNQIEAAEGTVIYTIKGKTPSTTQMALVVEAISRSIV
KSLAVQLFQVEHFVNDTWIATLLRAADGVVYVDARGSDEAVDHVVERDTELEFAPQKDIVARKIRIYT
PAAPSAVVIWDPAIEREEKADAQGTILMIYGPDLQDISCFTDIGAEAKIESLKSWDEKEKYVVHRDDIPE
DISTLVGKSSKVNKEFGIPDVAVCAPAKPGVTESIDRGEPKAGSCGEVANLPYPQESLVNRVSSANVQ
KNISMVLGGHPVGEMGISREIERHLETNMCEIFVDKDDVDVALDFYRISLTALRVGLDPPNAKVEIDGK

ESRKLIAVMDGDADLQRHPKLSEESGALRKADTKARLPDDALLYDGVQHGDTSFTATDLPDGRV-
VTPVNNKVKAQLKIDKVMIRTFHVVTLSFSNSKGEDHLIKLRLNALKKSTRHYEMLLKIRDTECATSPHA
ISVGKILKVDSLLQTICEAHYTEIEPLMADDLTALGVMGDQGLLHVVPRLMILAVLHLFSLEAAMTLDH
WSLQEKGLITDQTMGLPARAQAWSY-CISEVVGNTFFARTKDDDTEQRLHARNPI-VLL-
YGKIGMV-
ISPSGLRLDSTAVDEMERGNKDQLILSRERLQDAMGYYILRILLVFVDLVILAYGSGDTPMKFLVADQA
VTGRNNPQLVCLSRRTFPLDLQRDATFVMVFADATMKNLSHKKMHMNLVLIKSKGWVVNVSTKILIS
DQAIASYAEDVGAHTQPSESALAAITSRVMAPIVLVVSQKPTAPSKTLFLEALNLLDEVTATVLNKPKR
YFVFEGDVVSVLGTVDAAVLKGVQRPQPDRMRRAVVAKAIRLAIAAQASGLLASLEAGVINFSRAVSL
AAGVFDDMDLVPQAATE-PALAFRVRNHKVSTKRARPYKGHTKFLHMNDTDKSLIAEGVDTIA
>GCA_000158135
AVIKPVTQDTYAPVLLIRQQEVATVTGAGSPSLRAKTVYRKSGLQRKTSTKWVAAPIYY-
GVLKMGNIVQLVSISVDNRSVCTVAQVVEQEVVVHGLVALEAVKRKIALEAMDGMTTEDPAKVAEFE
EEVDLKKFRDADARADMLPVMLGLTLQIRFDPHEPVVHDLPIPVIYEVI-
GIMADKGGIEVDDCAISNAVSDVDPIAYVSPIDLAAVAGEQMAFIDQEAAVAAAREAIELEELALEAGI
LVVGISPLLLPV-FGEEGDV-MSFANRSRTLESIVALAVAYPALTRARIDG-IASAM-
MQRAQAAALAEKRGTAEEIRIVKRMQRSSGLADDPELVILEDASEGNSSWAGQIYDDPISGSSESDSA
GNIVHAANKRQNFARSITYLRKVEITDKAAAPLMRGVKHNAKSRSARKLNDAIAILNFPNVPAGIAIRA
EAGKPGATLLHDATEAEEILLIVVRWHVTWRTAIIVVLTFTAGDIVGLIEMGGIAAVGKPDGLLNRHHKL
NGSLIEQRPIIEKLTLRLRASLKEKLGAEQIIIFVVKVQGDLVADAKVQMG-
GPVGAVVAIQGDVIFVRRHKQTVITDQESTV-VLKGVAEGPADAQTLLRIRVTHTLVELSVLLIPE-
PVLVESVTIREGVGKALQIIQLDVYLLRDMGTGTETLAESGSRELIREVLAVDPITGVRLAYGKIKERMGK
KVGLKDLD-----
LSILAAVTEPRMQNLPAGVCRTFIVAGPRAAVGMLFLLLSDEGKLYAIDGDMRELLKSVKSLISLVDAK
APKVLSAAYAEDRLKIRFADCEVLVSEADLYAYKEERRELLRMVPVYIILEAKRRQLIIFALSLKGGHRRT
MMALGVMIAAAIIESQADKGPIHHDQPEILLDDAEVVGSSVMISRQAALDIKQFTKVDVVVIFPEFQE
KMVVSLKFFCTDVAIFVIALITTVGDMLSREPEHALESGAGYQKRIRGHSVLFALAATFDRRNMGDA-
TSDLFLALGLIDPRYGILYTNQPKKKQMPSEPDSDRVDGFELVHAIKEDTASNEELEMPEAMELAVEYD
ASINVSAGVLALSGGMQAVEKVDSKRKVALAATRWNISRLKIQDIHLSGAAKFRSCG-
IELADLSAALSNKKLWFLFLPGASNAELEEEKLDGAGASTADAAEDKQIAILLVKPVMVVIQDSNAASL
VANGGVDEWGTMAYKKHYSFAARMRLHMRTID-------
MAPNKQKSEKVKTVKLEGDTRFRQLAVDGIKEQMP-LHALNPDFAV-
LPLLVTPAVGSVAADENVLGARVPVDASL-
RELQKSQKGAALPISGVRLLAMRAARGMIEEDIMVAGKIAA-
LLKIRALETVVDIVLRNDSEATVQWRLSVAEKLEADTKRGGGFAARPFKSKPSLCDTQAHAKVADPIQE
IAAGYVAADERFVYPVEGIAIQLILTNPHSRVGTAKRAGEGHLHEMAKELMAKAVLMLRNEPDTNTEG
LLNYLTTNQPTDLLFEDCLDVIIDIRRMSVV--
VHLNTV-
HVVVEGVCEVGDYTIMAAAFPSGLVQVERVDDRTGHERTLMWMYGAMEELWLLSVKWADWVFD
APIGAIILHVYAQNINRAFSLKRLQLDGWPKEAARWWNTWQLWLNKDGFFGYAAAFRA-
KQVLKATLADYEMSDIKGVGRMASKQEIRAGYRIRENVDFARLDPRTDILLQAQTRLSIIRHALTVVAIL
VLRDVAPEFT-
VVAYQDVARGHVECVLAESFDFTEKNAHIGPVGLRTPETIFGDGTVMREAVLGKIDLANTVADFRYLV
LVDGGRALGVEWLTIQNMLVGRIWLPPAQAALKNLLPICFFLQHPDLDL---------

ISKARAEKLMAGALVEEILEGIARFADPEATTHQSKKAKHFKYYGEEVDPVHLAYILDIFALVSTQVTVS
DRLLKDRYVLNLKVFRGLTSLWWLAGQLSDKGPAATVITVGTISGLGALVLFIILL-
RDSVLVETLYHRYVY--MRGYSAAKDVADADPEHFLVLSAASRDLEWIEIVPAFFYYFKTAS-
FEPFNTAVAKEFFGLFEERLRVIDVLAVPPDRVAALTEFVALAGTLATSAEASPGVIATGLCEPNAAAVL
EAERDWNLHRSMGITLAFFEARASVKGYFDKGLHKGVAEINLQEAGALTTHFQCKAEAKRCVPAVHR
VLDLGEIEAALIEIDSAVTVADEEQTLADKPTTRDKTVGAIGGVVLAGTMNMGANLDSVERGGNIIIELE
EEQYSAVNGKKADGKLTDKVLMVLSLCYFPVIERPASANALDRGVFHLLIDAPEDADNLDHGYTMTIP
APSVHTETTPLYAESDIDKIAWIAPLHLLGTLAAVL-FEPDQYSFISMMIEASLEITETAEV--
DYHVLEKGSRPPAFRVVLIKGLHIRLLSAVGTILVVGSAGRAEITAYIQGQAVPITLRKHTFLPAFSASSCK
VVDIMSTMLRPPERSTVEMLILETVAEI-
LEATRGENEKLERMQMLLPGLDMANLLALVAAAADFSDIVTIGAAITAILSVAAESVVLREMLLTKAEIA
ITALALALALLLMLTGDKGLTQKWRAREAQMKELIQSGAVLQVHADDAMVLAATADKVRLDTDEGT
TSAFASLNLATVSDGFYVLSSAVLQMSADLMMIVAVARVLADKESKNALVVRHPEQVKTQPTYAEGS
PGMVLQTLTECIPPLALKADEVELLWTLVAALWHAKAHLASAARPRIERVLILLLALGLIVVLMQSEGG
GMGGG-
MGAAAKILACFSMLTLKASVGDEVRKVTRRVMDLNSRLGRPEEAVNPEFKGEELDQTKYEAENVEVD
CLETIRRFVDIPFLVSIRTQHNESEDRGARLVLDVTRVRFIDL-
VTKKSDVILVERVETDGIIARQMAKDMQSLHPEAAALGKVSALAHFIALNDGHISNYVTADVGSVLG
MLLLVLFIQQVRVAKVVWPTRREVTMVFIMAALAVFFLVIGLLVNLVFMVGGISPLQITTLVLGEGVMA
VRQVSTFPSES-
ITILQNTYADLICAGRVTKADIERSLHIVVAARVARGTWYVIYHYEISQAQGPSGIMQRAFRQATLSQS
LIDGRMEVKIKVVTSDRYA-
TYEIKGGQHRKGTRYNRVKVGHNIPVDVLARIKMAFGPPNLVLVKTFGAVVQKRFAQQLTHIRHGAV
TIGPMEVSSPYIQSKKARHAPGDSVVDIKPDNIVRSVAVVPELIWARENEDQSAVHEKYVKTTHKIAAK
RSRAVDKAQYDKRNQVHLSFIGLAVKVLPEKAIVQATAGMLLPTESVAVTENLKGIAATIRLDPMEKAL
IALIKRMRPQLNLQGKTDNKVVKCLNNNLDDVEWGKLGRRKIKPITIKSVYDVEHKRATELVKLMPYK
DDVEGRKSQFAEK--
ALFEPLKAFMMLFGFIARAVYALSLRVVRRTILLVKTKIVVNAETVLSAIKIIGVSIRLRHKSTNVADTKHHI
KTIEARARMTLAGAPAVKLHLRPGRVGKKGGRIKGYMPYKNFNIFDGLASGRRAKVKELAKLKKVDKTI
TVDGIRTRQ-
GGRLPQAELSNALANTRVGVDIVGENEQGASNPRANAEMGQIFAAIDARVYSLVAALIGHALAAVLA
VCAILGIGRAHALLAVKLKINAGIELHSAA-VAAAVAAA-
YAQPGGEAAALWDVSEAYLGALVVTEESLDVCRLRRRQLG-
LKKTKKEAYMSLTCFSGKGDKVKIVEATQTQAFEIVATRTAERHGIKAKGTYGKERAMMQITPTKPVKV
WVPVFVNDDELLAPIRAVAETNGDGVAVVKLTTDIKVTMFEKGVASLAISGKATTLVYTVLEKVTELSK
DIIYEQRAKAHYGRGRLRSHVVTTTKQKRHLSMIKRTVVASPHMEAARLVDGEVSVVTGATMAFVMS
EQDAV--
GDVDMHFHEEPRREAQLGIAGPETFLSTEQPRALALLFLREYLIHIVDIDTTGNFSDAATIYDILRAMQN
KLDRKLSADIAKVRMCLADDVADMGVTDVAARRDTAGDQILSAYWINGVPANADIILLVANLECHT
APENIDCAVRLFYQRITLQAKERNGDVSVALVPAQNQIEAAEGTVIYTIKGENPGTSQMKLNVEGISRS
IVKSLAVQLFQVDVFLNDTWIATLVRAAQDVVFVDAREGKEAVDHVVERDTELEMAPQKDILGRKIR
VYTPAAPSAVVIWDPAIERADKADAQGTILMIYGPDLQDISCFTDIGAEPKIESLKSWDEKEKYVVHRD
DIPPDI-
TLVGKSSKVNKEFGIPDVAVCAPAKPGVTESIDRGEPKAGSCGEVANLPYPQDSLVNRVSSANVQKNI

SMVLGGHPVGEMGISRGVERHLETNMCQIFVDKADVDVALDFYKLSLTSLRVGLDKPNAKVQIDGKE
SRKLMAIRDGEADLQRHPKVSEESGVLRKAGEKASLPDDTLIYDGVQHGDLKFTATDLPEGRV-
VTPVVMAVKAQLEIDKVMLKTFHVVTLSFSNDKGDEHLIKLRLNALKKSTRHYEMLLKIRDTECAQSP
HAIPVGKILKIDDLLQTICVAHYTEIEPLLADDLTALGVMGDQGLLHIVPRLMILAVLRLIQLDAATLIPK
WDLQHRALITDQTMGLAVRRRDWSF-AISEVVGNTFFARTKDDDTEERLHARNPI-VIL-
YGKLGMV-
ISPSGLRLRSTAPSEQQHGNDTKLILSRERLQDAMYYYILRVLLVFVDEMILAYGSGDQPMKFLVASQA
ITGRANPQLVCLTRRTFPLDLQRDATFVLVLEDSTMKNLSHKKMHMNLVLVKPKSWVENVSTKILISD
QAIASYAEDAGAHTQPSESALAVITSRVIAPIVLVVSQKPTAPSKTLFLEALNLYDEITATVQKRPKRYFIF
EGDVISVLNDMDAAVLLGMQRPQPDRLRRAVTAAAIRLAIAIRASDIVAALDNDCINFSRAVSFAG-
HYSRA-------------DFRVRNHKTSTKRDRPYKGHPKFLHKAEEEKALVAEGVDTLA
>GCA_000161755
AVIKPVVLNTYAPVLMIRQTQVATVSGAGSPSLRAKTVFRKSGLQRKTSTKWVASPLYF-
GVLKMGKIVQLVSISVDNRAVCTVAQVVEQEVVVRGLVALAAVKRKIALQEISGMTTEDPAKVVEFEA
ELDLKRFRDADDRADLLPVMLGLTLSIKFDPHEPVVYDLPVPVIFEVI-
GIMAERGGIEIDECGISTAVSDVDPISYVGPIDLEVVGGEKMAYIDQEAAVSAVKEEIGLEELALESNVLV
VKI-PLLLPV-FGEDGGN-MSFAKRSRELASIVDLAVSAVATTRAR-----AATM-
MARAQAAALSEKRGTAEEIRIVKRMQKAAGLADDPDLVMLEDAAEGNAAWAGQIYDDPISNSSESD
SQGNIVHAANKRQNFARSITFLRKVEISDKAAAPLMRGVKHNSKSRASRKIDDAIAELNFPNVPEGIAI
RAEQNKPSATLLHDATETEEILLLIVRWDVTWKTAVIVGLLFIAGNAVGLIELGGIAAVGKPDGLLNRH
HKLNGSLIEQKRVIDKLTLRVRAALKQKLGTENVIILVVKVQGDLVADATVQM-
GGVLGAVVAIQGDVIFVRRHRQTVVTDSEATV-VLRGVAEGPADAQALLRIRVDHTLVEVSVLLIPE-
PVLVDSVSIREGVGKTLQIIQLDVYLLRDMGTGEETLAESMSRELIRDVLTIDPITGVVLAYGGFRERASK
GLTLKELETL---
LAIMGAVTEPRMENLPEGVCRDFVQAGPRAAVGMIFLLLDDAGRIYEIDGDLRELPKTVKSLIGLVDAK
APKVLSASYATERLRIRFADCSVLESEANLYAYAEERKELLRMVPVYVINEAKRRQLVIFALSLKGGHRR
ARAALGVLIAAALLESQATQGEVHHAVPEVLLDNAEVVGTSVMISRQSALELKHVLKSDVVVVFPEFQ
EKMVVSLKFFCTEVAIFVIALITTVGEIISREPEHAQESGAGFAKRIRRHSVLFALACCFDRRNLGDA--
PDLFLSLGLASPRYGVLYINQPKKKQMPSEPDGDRVDGFELVHAVKSDGRS-HELEMPEAMALAVE-
TEKINLTAGVLALNGGMAAVERVDSKRKLGVAASKWSISRLESLDVHFSGAAPVRCTK-
IELADISAALASKKLDALYLLGASNAELEEDKLDGAGNSTADVAADRQRQILLVHPVIVVIQDSNAAGL
VSNGGIDEWGTMAYKKHYAFAARMRLHMRTIE------
ELEPNKQKSEKVKTVKLEGDTRFRQLAVEGVKEQMP-
LHALNPDFAVLVPVLVTPAVGQVAADAEVLGAEVPVEFSL-
RELQKSQTGAALPISAVRLLEMRAARAMVDEDIMVAGKVAA-
LLKIRALETVLQVVLRNDSEAWGPWRLSVAERLEADTRRASGFAARPFRMKPSLCTTQAHAKLAEPIK
EIAAGYVATENRFVFPVEDIAIQIVLSSPHSRVETSKRAGEGHLHEMAKELVAKAVLLLQNQPPTTTEGL
LNYLTTNQPTDLLFPDCLDVIIDLRRMAVV--
VHLTTV-HVVVAGTSDVGDYEIMAAAA-
SGLVQVERVDDRTGRERTKMWMYGAMEELWLMSVTYADWVPDCAIGAIILHVYAQTINRAFSLRRL
QIEGWPKEADRWWNTWQLWLNKDGFFGYEAAFRAQAQVLKATLFDYEMSDVEGVGRYQSRQQL
RVGYRIRDQVEFARLDPRTDILLQAQTRLSVIRHALTVVAILVLREVVGEFSLVVAMEDVAMGHVECVL
ADSFSFTAKNAHIGPVGLRTPDTIFGDGTVLKEAVLGKIDLRNTVAEFRYRMITDGGRELGVKWITVQN
ILYARIWVPPAQAALKNLLPICMFLSHPEVDL---------

ISKARADKLMAGALIEEIIEPVARFAAPEATTHQSHKAKHFKYYCEEVDPVHLAYILDLFALVSTQVTVS
DRLLKDRYVLNLKVFRGFTSLLWISAQLGEKGPSATIITVGTIAGLGALVLFIILL-RDAVLLETLFYRYVY-
-MRGYSAAKDVADADPEHLL-LSAATRDVEWIEIVPAFFYYFSTATKN--
KNTAVAKEFFGLFEERLRVIDVLAVPPDRVEALEEFVALAGTLATSAEALVGIIATGFCSPNAAAVLEAD
QDWNLHRSMGITLAFYKAQKSVKGWFDKGLNKAVAETQLREAGALTSTFAAAVEAKQCVPAVHRVI
DLGEIEAALIEIDSRVIVADEEQTLADKPTTREKSVGCVGGVVLAGTMNMGSNLEQVERGGNIIIELEEE
HYHAVNG-
KADGKITDKVLMVLSAALFPVLARWASVNALPRGVLGLLIEAPNDADNLDHGGLMSIPAPSVHAETT
PLYAEAEIDKIAWIAPIHLLGTLDAVLLFEPDQYSFISMMIEASLEITETAEV--
DYHVLEKGARPPGFRVVLIRGLHIRLLAAVGTILVVGSAGRTEITAYIQTRAVPITLNKHTFLPAFSASSC
HVVDIFSTMLRPPERSAVMMLILETVAEL-
LDVTRGENDKLERILVLLPGLDAAALLELVAAAADFSNIVTLDAAITAILSVAAETVVLRELLLTKAEIAVT
ALALAIALLLMLTPDSGLTQREVAREGQMKELIQGGAVLQVHAGDRMVLASTADKVRLNTDEGTTSA
FASLNLATVSDGFYVLSSAVLQMSSDLMMIVSVARVLADKETKNALVVRHPEQVKTQPTYAEGSPG
MVLQT---ILVLLLALSLIVVLLQSEGGGMGGG--
SAAAKILACFSLLTLKASVGEEVRKVTRRVMDLNSRLGRPEEAVNPEFRGEELEQSKYEAENVEVDCVE
TVRRFVDIPFLVSIRTQHNESEDRGARLVLDVTRVRFIDL-
VTKKSEIILVERAETDGVIAREMAKDLQALQPEIAALGKIAALQHFVSLNDGHISNYVTADIHSVLGMLL
LVLFIQQTRIGKIVWPTRREVTTVFIMAALATFFLVIGLG-
NLVFMVGGISVQQITTLVLGEGAMLVRQTSTFPAES-
VTILQNTYADLICAGRVSKADIEKSLHIVVGARVARGTWYVIYNYEITQAQGEAGILQRAFRQATLSQS
LIDGRMEVKIKVVTSDRYA-
TYEIKGGQHRKGTRFLRVKVGHNIPVEVLPRLKMAFGPPNLILVKTFGAVVQKRFAQPLTHIRHGAVTI
GPMEVASPYIGNKKARHAPGDSVVDVKPDNIVRSVAVVPELIWARENEDQSAVHDKYVKTTHKLQA
KRNAAVDKAQYDKRNQVHLSFIGLAVKVLPEQA--

RMRPQLNLAGKSDKKIVKCLNNGLDDVEYGKLGRRRIKPLTIKSVYDVEHREATELVKLMPYKDDVEG
RKSQFAEK--
AIFEPLRAFMMLFNYIARAVYALSLRVVRRTIVLAKCKVVVNANTVLSAIKIIGVSIRLRHKTTNIADTKH
HIKTIEARARLTLAGAPSAKLNLRPGKIGKKGGRIKGYMPYKNYNIFDGLSSGRRAATKELAKLKKVDKTI
EVGGIRTRQQGGRLSQADLSNAIANTRVGVDIVGENEQEESNPRANAEMGQVFAAIDARVYHLVAA
LIGHALEAVLALCDIWGIGRAHSLLADW-RVNAGVEFHSAA-VETAVAAA-
YAQQAGEASVIADVSEGDLGALGLAEASLDVLRLRRRQLG---------------------------------
----
RFGISAKGHYGKERAMMQITPTKPVKVWVPVFVSEDELLAPIRAFAETNGDGITVVKLTTDIKITMFEK
GVASLAITGKSTTLVYTVLEKVTELAKDIIYEQRAKAHYGRGRLRSHVVTHTKQKRHLSMIKRTVVAKPR
MEAARLTDGEVSVITGATMAFVMSEQDAV--
GDVDMHFQEEPRREASLGIAGPETFLSTEQPRVLALLFLREYLIHIVEIDTVGGYNDAATIYEILRAMESK
LDRKLSAEIAKVRMCLHDDVADMGIRQEAPRREKAGDPILSAYWINGVTANADIILLVANLDCETPPE
NIDCAVRLFYQRVTLQAKERGGDVSVALIPAKNQIEAAEGTVIYTIKGEKPGTVQMKLNVEAISKSIVKS
LAVQLYQVDLFINDTWIATLLRAADDVVYVDAREGREAVDHVIERDTELELAPQKDIIRRKIRIYTPAAP
SAVVIWDPAIDREDRADAQGTILKIYGPDLQDISCFTDIGSEPKIESLKAWDEKE-YVVHRDDIPPDI-
TMVGKSAHVNKEFGIPDVEVCAPAKPGVTESIDRGEPKAGTCGEVANLPYPQESLVNRVSSANVQKN
ISMVLGGHPVGEMGISREVERHLETNMCELFVDSPDVDVALDFYRLALT-

LRVGLDRPNAKVEIDGKESRKLMAVLDGGADLQRHPKVSEAAGTRVGAGRKARLPDDTLIYDGVLH
GDLSFTVTDLPEGRV-
VTPVTIKVKAQLKIDKVLIKTFHVVTLSFSSQKGEEHLIKLRLNALKKSTRHYEMLLKIRDTECAQSPHAIP
VGKILKIDKLLQTICVAHYTAIEPLLADDLTALGVKGDQGLLHVVPRLMILAVMTLWGLEATLTWPRW
GLMARRLVTDVTMGLAMRERHWSY-CISEVVGNTFFARTKDDDTTERLHARNPI-VLL-YGKFGMT-
ISPSGLKMASTAPSTAEHGNDILLILSAERLQDTMSYFVLRVLLVFVDEVILAYGSGDTPLRFLVAHQAV
TGRANPQLVCLTRRTFPLDCQRDIVFVLVFEDSTMKNLSHKKMHMNLVIIKSKGWI--------------
----
IVLVVSQKPTAPSKTMFLEALNLYDEVTCTVQNRPKRYYIFEGDLISVVGSVDAAVLRGMQRPQPDRL
RRAVVAAAIRLAIASLAAEVLDLLEAGLLNFARAVSTAV-VYGAPQE--
GVVVPMATVEFRVRNHKVSTKRSRPYKAHPKVLMKSDDEKKLIADGVDTLA
>GCA_000161775
AVIKPVTQDVYAPVLLIRQQEVATVTGAGSPSLRAKTVYRKSGLQRKTITKWVAKPLYY-
GVLKMGNIVQLVSISVDNRSVCTVAQVVEQEVVVHGLVALDAVKRKIALEAIDGMTTEDPAKVAEFE
EELDLRKFRDADARADMLPVMLGLTLKIKFDPHEPVVHDLPIPVIYEVI-
GIMADKGGIEVDDCAISNAVSDVDPIAYVSPIDLEAVAGEQMAFIDQEAAVAAAREAIALEELALEAGI
LVVGISPLLLPV-FGEDGDV-MSFANRSRTLESIVALAVAYPQLTRARIDG-IASAM-
MQRAQAAALAEKRGTAEEIRIVKRMQRSSGLADDPELVILEDAAEGNSSWAGQIYDDPVSGSSESDS
AGNIVYAANKRQNYARSITFLRKVEISDKAAAPLMRGVKHNAKSRSARKIDDSIAELNFPNVPVGIAIR
AEAGKSDDVLLHDATEGEEILLVVIRWHVTWKTAVVVLLVFTAGDIVGLIAIGGIAAVGKPDGLLNRHH
KLNGSLIEQRPIVEKLTLRLRAALKEKLGKDQIIIFVVKVQGDLVADAKVQMG-
GPVGAIVAIQGDVIFVRRHKQTVITDQESTV-ILKGVAEGPADAQALLRIRVTHTLVELSVLLIPE-
PVLVDSVSIREGVGKALQIIQLDVYLLRDMGTGEETLAESKSRELIREVLAVDPITGVRLAYGKIKERMGK
KVGLKDLET----
ISILCAVTEPRMENLPEGICRTFIVAGPRAAVGMLFLLLDDAGKLYAIDGDLRELLKSVKSLIGLVDAKAP
KVLSASYAEERLKIRFADCEVLAAEADLYAYKEERRELLRMVPVYIIIEAKRRQLVIFALSLKGGHRRTMM
ALGVMIAAAILESQAEKGPVHHQEPAVLLDDAEVVGSSVMISRQASLDIKQFTKVDVIVIFPEFQEKMV
VSLKFFCTDVAIFVIALIVTVGEMLSREPDHALEQGAGFQKRIRGHSVLFALAVTFDRRNTRDALGPDLF
LEMGLADPRYGILYVNQPKKKQMPTEPDSDRVDGFDLVHAVKEDKASNHELEMPEAMALAVE-
DATINLSAGVLALSGGMVAVEKVDSKRKVALAATKWNISRLSALDVRLSGAWPFRSTGNIELADLSAA
LSRKKL-
FLYLPGASNAELEEEKLDGAGASTAEAAADKQVAILLVRPVLVVIQDSNTAGLIANGGLDEWGTMAY
KKHYAFAARMRLHMRTID------QLAPNKQKAEKVKTVKLEGDTRFRQLAVEGIKEQMP-
LHALNPDFVV-LPLLVTPAVGSVAADENVLGADVPVDASL-
RELQKSQKGAALPISGIRLLAMRAARAMIEEDIMVAGKIAA-
LLKIRALETVADIVLRNDSEATIPWRLSVAKKLEADTKRGGG-------------------------------
----
VEGIAIQLILTNPHSRVGTAKRAGEGHLHEMAKELLAKAVLMLRNEPGTNTEGLLNYLTTNQPTDLLFE
DCLDVIIDIRRMSVV---VHLNTV-
HVVVKGVCDVGDYEIMAAAFPSGLVQIERVDDRTG------------------------------------
-----INRAFSLKRLQLDGWPKEAARWWNTWQLWLNKDGFFGYEAAFRA-
KQVLKATLADFEMSDIEGVGRYLSKQEVRAGYRIREQVEFARLDPRTDILLQAQTRLSIIRHALKVVAILI
LRDVAPEFT-
VVAYQDVARGHVECVLAECFEFTEKNAHIGPVGLRTPETIFGDGTVLKEAVLGKIDLANTVAEFRYLVL

VDGGRELGVEWLSI-NMLYPRIWLPPAQAALKNLLPIKFFLQHPELDL---------
ISKARADKLMAGALVEEILDAVARFAEPEATTHQSKKAKHFKYYGEEVDPVHLAYILDIFVLVSTQVTVS
DRLLKDRYVLNLKVFRGFTSLWWLAAQLAMKGPSATVITVGTIAGLGALVLFIILL-
RDSVLVETLYHRYVY--MKGYTAAKDVADADPEHFLVLAAASRDLEWIEIVPAFFYYFKTAG-
FEPFNTAKAKEFFGLFEERLRVIEVLAVPPDRVEALTEFVALAGTLPTSAQA-
PGVIATGLCEPNAAAVLEAERDWNLHRSMGITLAFFEARESVKGYFDKGLHKDVAETSLREAGALTTH
FHCGKEAKRCVPAVHRVLDLGEIEASLIEIDSAVTVADEEQTLADKPTTRDKAVGAVGGVVLAGTMN
MGANLDSVERSGNIIIELEEEHYHAVNGKKANGKLTDKVLMVLSLAYFPVITRPASANAMDRGVFYLLI
AAPEDADNLDHGGVMSIPAPTVLTETTPLYAEAEIDRIAWIAPLHLLGTLPAVLGFEPDQYSFISMMIE
ASLEITETAEVLDDYHVLEKGSRPPAFRIVLIKGLHIRLLAAVGTILVVGSAGRAEITAYIQGQAVPITLRK
HTFLPAFAPSSCKVVDIMSTMLRPPERSTVEMLILETVAEI-
LEATRGENEKLERMQVLLPGLDVATLLGLVAAAEDFSDIVTIDAAITAILSVAAEEVVLREMLLTKAEIAIT
ALALALALLLMLTGDKGLTQKERAREAQMKELIQSGAVLQVHADDAMVLAATADKVRLDTDEGTTS
AFASLNLATVSDGFYVLSSAVLQLSADLMMIVAVARVLADKETKNALVVRHPEQVKTQPTYAEGSPG
MVLQTLTQCIPPLAVKAEEVELLWTLVAALWQAKAHLASAARPRTERVLILLLALGLIVVLMQSEGGG
MGGG-
MGSAAKILACFSILTLKAAVGDEVRKVTRRVMDLNSRLGRPEEAVNPEFKGEELDQTKYEAENVEVDC
LETIRRFVDIPFLVSIRTQHNESEDRGARMVLDVTRVRFIDL-
VTKKSDVILVERVETDGIIARQMAKDMQSLHPE-
AALGKVAALDHFISLSDGHISNYITAAINSVLGMLLLILFIQQVRVAKVVWPTRREVTMVFIMAALAVFF
LVIGLLINLVFMVGGISPLQITTLVLGEGVMAVRNVSTFPAES-
ITILQNTYADLICAGRVTKADIEASLHIVVAARVARGTWYVIYHYEISQAQAPAGIIQRAFRQATLSQSLI
DGRMEVKIKVVTSDRYA-
TYEIKGGQHRKGTRYNRVKVGHNIPVDVLARLKMAFSPPNLVLVKTFGAVVQKRFAQQLTHIRHGAV
TIGPMEVSSPYIKSKKARHAPGDSVVDIKPDNIVRSVAVVPELIWARENEDQSAVHEKYVKTTHKVAA
KRNRAVDKAQYDKRNQVHLSFIGLAVKVLPEKA---------------------------------------
--
RMRPQLNLAGKTDNKVVKCLNNNLDDVEWGKLGRRKIKPITIKSVYDVEHKRATELVKLMPYKDDVE
GRKSQFAEK--
ALFEPLKAFMMLFGFIARAVYALSLRVVRRTILLVKTKIVVNAKTVLSAIKIIGVSIRLRHKSTNVADTKHHI
KTIEARARMTLAGAPAVKLHLRPGRVGKKGGRIKGYMPYKNFNIFDGLASGRRAKVKELAKLKKVDKVI
TVDGIRTRQQGGRIPKADLSNAL-
NTRVGVDIVGENVQEASNPRANAEMGQIFAAIDARVYSLVAALIGHALEAVLAVCAILGIGRAHALLA
V-LKINAGIELHSAA-VTEAVAAA-YAQPGGEAAAIADVSEGDLGMLALTEESLDVCRLRRRQLG----
----
RHGIKAKGTYGKERAMMQITPTKPVKVWVPVFVNDEELLAPIRAVAETNGDGVDVVKLSNDIKITMFE
KGVASLAISGKATTVVYTVLEKVTELSKDIIYEQRAKAHYGRGRLRSHVVTTTKQKRHLSMIKRTVVASP
RLDAARLVDGEVSVVTGATMAFVMSEQDAV--
GDVDMHFHEEPRREAQLGIAGPETFLATEQPRALALLFLREYLIHIVDIDTVGGFADAATIYDILRAMQ
NKLDRKLSADIAK------------------------
GDPILSAYWINGVPANADIILLVANLECHTAPENIDCAVRLFYQRITLQAKERGGDVSVALVPAQNQIE
AAEGTVIYTIKGENPGTTQMKLTIEGISRSIVKSLAVQLFQVDLFLNDTWISTLLRAAQDVVFVDAREGK
EAVDHVVERDTELEVGPQKDILGRKIRVYTPAAPSAVVIWDPAIERA---------------
DLQDIACFTDIGSEPKIESLKSWDEKEKYVVHRDDIPPDI-

TLVGKSSKVNKEFGIPDVAVCAPAKPGVTESIDRQEPKAGSCGEVANLPYPQESLVNRVSSANVQKNI
SMVLGGHPVGEMGISREVERHLETNMCEIFVDKADVDVALDFYKLALTALRVGLDKPNAKVQIDGKE
SRKLMAVRDGEADLQRHPKVSEESGTR-KAGEKAKLPDDALIYDGVQHGDLKFTATDLPDGRV-
VTPVVMRVKAQLKIDKVLIKTFHVVTLSFSNDKGEEHLIKLRLNALKKSTRHYEMLLKIRDTECAQSPHA
IPVGKILKIDALLQTICQAHYTEIEPLLADDLTALGVMGDQGLLHVVPRLMILAVLHLIDLEAAKLIPKWG
LQQRAIITDQTMGLPARQRDWSF-AISEVVGNTFFARTKDDDTEQRLHARNPI-VIL-YGKLGMV-
ISPSGLHLNSTKPSDQQHGNTQKLILSKERLQDAMYYYILKVLLVFVDELILAYGSGDKPLKFLVAHQA
VTGRENPQLVCLTRRTFPLDLQRDATFVLVVEDSTMKNLSHKKMHMNLVLVKPKSWVENVSTKILIS
DQAIASYAEDAGAHTQPSESALAVITSRVIAPIVLVVSQKPTAPSKTLFLEALNLYDEITATVLKRPKRYFI
FEGDVVSVLGTMDAAVILGMQRPQPDRLRRAVTAAAIRLAIALRASGIIASLDAGLINFSRAVSLAA-
HYPGVQALADP-------AFRVRNHKTSTKRDRPYKGHPKFLHKADEEKALVAEGVDTLA
>GCA_000161815
AVIKPVEINKYAPVLMIRQTQVATVSGAGSPSLRAKTVFRKSGLQRKTMTKWVQSPLYY-
GVLKMGNIVQLVSISVDNRAVCTVAQVVEQEVVVHGLVALDAVKRKIALEEMDGMTTEDPAKVAEF
EEELDLKRFRDADARADMLPVMLGLTLQVRFDPHEPVVHDLPIPVIYEVI-
GIMADRGGIEVDDCAISNAVSDVDPIAYVSPIDLAAVAGEKMAYIDQEAAVGAAKEEIALEELALESVV
LVVGI-PLLLPV-FGAEGAV-MSFAARSRTLESIVGLAVAFAELTRA-ID-GIASAM-
MTAAQAEALAEKRGTAEEIRIVKRMQRSSGLADDPDLVILEDTAEGKASWAGQIYDDPISGSSESDSL
GNIVHAANKRQNFARSITYLRKVEISDKAAAPLMRGVKHNSKSRSARKVDDAIADLNFPNVPAGIAIK
AIAQKPEDRLLHDATETEGILLIVVRWHVTWKTAIAIGLTFLAGNIVGLIALGAIAGIGKPDGLLNRHHKL
NGSLIEQRPIIEKMTLRLRASLKEKLGKDQIIIFVVKVQGDLVADATVQMG-
GPVGAVVAVQGDVIFVRRHKQTVITDSESTV-ILRGVAEGPADAEALLRIRVEHTLVELSVLLIPE-
PVLVDNVSIREGVGKSLQIIQLDVYLLRDMGTGEETLAESKSREVIREVLAIDPITGVRLAYGKLKERAGK
KTGLKDLETLF--
GKLYPIDGELRELTKSVKSLISLVDAKAPKVLSAGYAEERLRIRFADCEVLASEAELYAYKDERREMLRMV
PVYIINEAKRRQL------------
RAMMALGVMLAAAIIESQAEKGAVHHDEPAILLEDAEVTGTSIMVSRQASLELKHVSKVDVIVIFPEFQ
EKMVVSLKFFCTEVAIFVIALIVTVGDMLSREPEHAQEDGAGYAKRIRKHSVVFALEVTFDRRNCRSA-
LADLFLSLGLLPIRYGVLYTNQPKKKQMPSEPDGDRVDGFELVHAVKEDGSSNHELEMPEAMALAVE
ATETINLSAGVLAMTGGMVAIERVDSKRKVALAATKWNISRLELLDVTISGAAPVRCCD-
IEEAGMSAALGKKKV-
FLYLPGASNAELEEEKLGGAGNSTADVAEDRQREILLVHPVLVVIQDSNSAGLISNGGLDEWGTMAY
KKHYAFAARMRLHMRTIE-------MEPNKQKSEKVKTVKLEGDTRFRQLAIEGVKEQMP-
LHALNPDFAV-LPILVTPAVGSVAADAQVLGATVPVDASL-
RELQKSQKGSALPISGIRLLDMRAARGMIEADIMVAGKIAA-
LFKIRALETIIGIILRNDSEATLPWRLSVAERLEADTKRASGFAARPFKTKPSLCTTQAHARIADPIQEIAA
GYVAADERFVFPVEDIAIRLILYNPHSRVGEKKRGGEGHLHAMAKELLGKAVVMLRNEPGTNTEGLLN
YLTTNQPTDLLFEDCLDVIIDIRRMSVV--
VHLNTV-
HVVVAGVADVGDYEIMNAAFPSALIQVEQVDDRTGEEKTLMWMYGAMEELWLMSVRWADWVA
DCAIGAIILHVYAQTLNRAFSLKRLQLDGWAKEAARWWNTWQLWLNKDGFFGYEAAFRA-
KQILKAALADFEMKDVEGVGRYLSKQEIRAGYRIREQVAFARLDPRTDILLQAKTRLSIIRHALQVVAILI
LRDIAPEFT-
VVAYQDVARGHVECVLAESFDFTEKDAHIGPVGLRTPDPIFGDGTVFKEAVLGKIDLANTVAEFRYLVL

LDGGRELGVEWVTLQNMLYARIWLPPAQAALKNLQPICFFLQLPELDM---------
ISKARAEQLMAGALIEEIIEAVARFADPEATTHQSKKAKHFKYYGEEVDPVHLAYILELFALVATQVVLS
DRLLKDRYVLNLKV-
RALTSLWWLSLQLAEKGPCATIISVGTISGLGALVLFIILLMRDSVLVETLYYRYVY-
VMRGYSAAKDVQDADPEHLLELAAASRDIEWIEIIPAFFYYFDTAT-
FEPFNTAVAREFFGLFKERLRVIEVLAVPPDRVEALVEFVALAGTLAVSAEALPGVIACGLCEPNAAGVL
EAERDWNLHRSMGITLAFFEARDSVKGYFDKGLHKAVAEFSLKEAGAMTTHFRCDVEAKQVVPAVH
RTLDLGEIEAALIEIDSAVVVSEEEQTLADKPTTREKSVGAVGGVVLAGTMNMGANLDSVERGGNIIIEL
EEEQYHAVNGKKSEGKLTDKVLMVLSVAYFPVIARFASANALDRGVFGLLIDAPTDADNQDHGREMS
IPAPTVHAETAPLYAESDIDRIAWIAPLHLLGTLDAVLLFEPDFQGFISMMIDASLEIMETAEVMEDYHV
LEKGARPPGFRVVLIKGLHIRLLAAVGTLLVVGSAGRTEVTAYIQGRAVPISLRKQTFLPAFAPSSCNVV
DIFSTLLRPPERSAVEMLILETVAEL-
LEVTRGSNDKLERMQVLLPGLDMAELLELLAAADDFADIVTIGAAITAILSVAAEAVVLREMLLTKAEIAI
TALALAMALLLMLTGDTGLTQKEAAREAQMKDLIQAGSVLQLHAADAMVLAATADKVRLSTDEGTT
SAFASLNLATVSDGFYVLSSAVLQMSSDLMMIVSVARVLADKESKNALVVRHPEQVKTQPTYAEGSP
GMVLQTLTGCIPPLAIKPEKVELLWTLVAALWKAKAHLASAARPHNERVLILLLALGLIVVLLQSEGGG
MGGG-
ATAAAKVLAAFSILTLKAAVGDEVRKVTRRVMDLSSRLGRPEEAVNPEFKGEELDQTKYEAENVEVDC
METVRRFVDIPFLVSIRTQHNESEDRGARLVLDVTRVRFIDL-
VTKKSDVILVERVETDGIIARQMAKDMQSLHPEAAALGKVSALQHFIALGDAHISNYVTAATHSVLG
MLLLVLFIQQVRVSKVVWPTRREVTMVFIMAALAAFFVVIGLGINIVFMVGGISPLQITTLVLGEGVMA
VRQTSTFPAES-
VTILQNTYADLICAGRVTKADIESSLHIVVGARVARGTWYVIYHYEITQAQGPAGIMQRAFRQATLSQ
SLIDGRMDVKIKVVTSDRYA-
TYEIKGGQHRKGTRYNRVKVGHNIPVEVLARIKLAFGPPNLVLVKTFGAVVQKRFAQPLTHMRHGAV
TIGPMEVSSPYIKSKKARHAPGDSVVDIKPDNVVRSVAVVPELIWARENEDQSAVHEKYVKTTHKIAA
KRSRAVDKAQYDKRNQVHLSFIGLAVKVLPEQAIVEASAGTLLPTESEAVTENLKGVAATIRLDPMEKA
LIAYIKRMRPQLNLQGKTDNKIVKCLNNNLDDVEWGKLGRRKIKPITIKSVYDVEHARATELVKLMPYK
DDVEGRKSQFAEK--
ALFEPLKAFMMMFGFIARAVYALSLRVVRRTILLVKTKIVVNANTVLSAIKIIGVSIRLRHKSTNVAETKH
HIKTIEARARMTLAGAPSVKLNLRPGRVGKKGGRIKGYMPYKNYNIFDGLASGRRAKVKELAKLKKVD
KTIAVDGIRTRQQGGRLPKADLSNALSNTRVDVEVVGENEQEESNPRANAEMGQIFAAIDARVYSLV
AALIGHALLAVLAICSILGIGKAHVMLAVKLKINAGIEMHSAA-VAAAVAAA-
YSQPAGEATAIADVSEGDLGALAITEGSLDVCRLRRRQLG-
LKKTKKEAYMSLTCFAGKGDKVKIIEATQTQAVEIVATRTAERHGIKAKGTYGKERAMMQITPTKPVKV
WVPVFVNEDELLAPVRAFAETNGDGVTVVKLTTDVKITMFEKGVASLAVTGKVTTLVYTVLEKVTELSK
DIIYEQRAKAHYGRGRLRSHVVTHTKQKRHLSMMKRTVVADPSMDAARLTDGEVSVVTGATMAFV
MSEQDAV--
GDVDMHFHEEPRREAALGIAGPETFLATDQPRLLALLFLREYLIHIVDIDTVGNFSNAATIYDILRAMQ
NKLDRKLSADIAKVRMCLSDDVADMGVTDAAARRDTAGDSILSAYWINGVTANSDIILLVANLECKT
APENIDCAVRLFYQRITLQAKERDGDVSVRLVPAQNQIEAAEGTVIYTIKGNNPSTAQMKLNIDAISRS
IVKSLAVQLFQVDLFLNDTWIATLLRAAEDVVYVDAREGREAVDHVVERDTELELAPQKDIISRKIRIFT
PAAPSAVVIWDPAIDREDKADAQGTILMIYGPDLQDISCFTDIGSEPKIESLAAWDEKEKYVVHRDDIP
EDI-

TLVGKSSKVNKEFGIPDVAVCAPAKPGVTESIDRQEPKAGTCGEVANLPYPQDSLVNRVSSANVQKNI
SMVLGGHPVGEMGISRDVERHLETNMCEIFVDKDDVDVALDFYKLSLTPLRVGLDKPNAKVQIDGKE
SRKLMAVKDGEAKLQRHPKVSEESGTR-KADDKAKLPDDALIFDGVQYGDLSFTATDLPEGRV-
VTPVFNNVKAQLAIDKVMLKTFHVVTLSFSNDKGEEHLIKLRLNALKKSTRHYEMLLKIRDTECAQSPH
AIPVGKILKIDSLLQTICKAHYTAIEPLLAEDLTALGVMGDQGLLHVVPRLMILAVLHLLGLDARGQLST
WSLQEAAIVTDQTMGLAIRDRAWSF-AISEVVGNTFFARSKDDDTEERLHARNPI-MIL-YGKEGML-
ISPSGLKLASTQPSDDEHGNKNKLILSTERL-----------
LLVFVDEMILAYGSGEEPLKFLVAEQAVTGRANPQLVCLTRRTFPLDLQRDAVFVLVLTDTTMKNLSH
KKMHMNLVLVKPKSWVENVSTKILISDQAIASYAEDAGAHTQPSESALAIITNRVIAPIVLVLSQKPAA
PSKTMFLEALNLYDEITATVAKRPKRYFIFEGDVISVLNTLDAAVILGMQRPQPDRLRRAITATAIRLAIA
LRGSGVLAQLEGGVINFSRAVSLAAKVYDALNLLARAAIPLPALDFRVRNHKTSTKRDRPYKGHTKFLH
KAEEEKALVAEGVDTLS
>GCA_000161835
AVIKPVVQNVYAPVLMIRQTQVATVSGAGSPSLRAKTVYAKSGLQRKVETKWVASPFYY-
GVLKMGKIVQLVSISVDNRAVCTIAQLVDQEVVVHGLVALEAVKKKIALEAIEGMTAEDPAKVVQFEE
ELELKRFRDADDRADMLPVMLGLTLKIRFDPHQPLVHDLPIPMMYEVI-
GIMAQKGTIEVDDCAISTAVSDVDPIAYVSPIDLAAVAGDAMDYIDQESAVAAAKDAIALEELALESG
VLVVGIKPLLLPV-FG-DGDT-MSFANRSRNLASIVDLAVAYPEITRARISD--ASAM-
MMRIQAAALAEKRGTAEEIRIVKRMQRSAGLADDPDLVILEDAAEGNSSWAGQIYDDPISGSSESDS
AGNIVFAANKRQNFQRSITYLRKVEISDKASAPLMRGVKHNAKSRSSRKVDDAIQVLNFANVPEGVAI
RAEAACDDDALLHDATETEQILLMIVRWDVTWKTAIFIFLVYLAGGIVDLITLGGIAAVGREEGLLNRH
HKLNGSLIEQKRIIEKLTLRLRAALKQKLGADQVIIFVVKVAGDLVATATVQM---
SVGMVVAIQGEVIFVRRHKQTVITDSEATVEVLRGVAEGPADAEALLRIRVEHTLVKLSVLLIPE-
PVLVDSVTIREGVGKALQIIQLDVYLLRDMGTGTETLAQSGSRELIRDALQIDPITGVRLAFGKIKERLGK
VTKLKDLK-----
LALQAGVTEPRMSTLDDGVRRDYIEAGPQAAIGMLFLILDDAGKIYAIDGDLRENTKSIKSMIGIVDAA
TPKVLSAGYATERLKIRFADCMVLTSDADLYAYAEERKEVLRMVPVYITNEAKRRQLIIFALSLKGGHRR
AQASLDVLLPATILESQTEEGTVNHDTPVILLADAEIVGSEMMISRQAALDVKSIFKIDVIVVFPEFQEK
MVVSLKFFCTEVAIFVIALIVTVGSMLSREPEHAQESGAGFQKRVRAHNILFGLEAVFDRKNLRDA-
AADLLLNLGLAEPRYGLFYVNQPKKKQMPSEPDKDRVDGFDLVHAIKADGAS-
EELEMPEAMALAVE-
DASINVSGGVLALSGDMVAVEKVDSKRKMALAVEKWNIAKLTIEDIHLSGASPIRALGSIEQASLSAAL
SAKSIRVLFLPGASNKELEEDKLNGAGSSSAEAAEDRQTAILLVKPVLVVIQDSNTASLIANGGVDEWG
TMAYKKHYAFAARMRLHMRTID-------MVPNKQKAEKIKTVKLEGDTRFRQLATEGVKEQMP-
LHALNPDFAVLVPVLVTPAVGSVAADTGVLGARVPCDFSL-
RELQKSQKGSALPIAGVRVMGVRAARAMIDEDIMVAGKVAA-
LLVIRALETIADLVLYNDSEATLPWRLSVAERLEADTKRAGGFASRPFNSKPSLCNTQAHANVADPIETI
AAGYVAIEKRFVFPVEGIAIQIVLASPHSRVGGAKRAGEAHLHEMAKELLAKAVLMVLNEPDTNTEGLL
NYMTTNQPTDLLFEDCLDIIIDMRRMSVV--
VQLNTV-
HIVVPGASDLAEREIMAADADSGLVQVERVDDRTGRERTTMWMYGAMEELWLLSVTYADWVINCA
IGAII-------INRAFSLTRLQLDGWPPEADRWWNTWQLWLNKDGFFSYEAAFRA-
MQVLKAELYYFEMSDVKGVGRYLSRQEIRAGYRIRQQVDFARLDPRTDILLQAKTRLSVIRHALTMVAI
LVIRDVAPEFS-

VVAQEDVAMGHVECVLADCFDFTAKNADIGPVGLRTPSPILGDGTVFKDAILGKIDLANTIADFRYRFI
IDGGRALGVSWMPFMNMLYARIWLPPAPAKLKDLQPIKFFLSHPELDM---------
ISKARAQKLMASALIEEILENVARFADPEATTHQSNKEKHFKYYGEEVDPVFLAYIFDATALVATQVTVS
DRLLQDRYVLNLTI-
RGFTSLLWLSAQLGNKGPAATVITVGTIMGLGALVVFIILLLRDVVLLETLYYRYVY--
MQGYSAAKDVADADPEHFLSLAAASRDIEWIEVIPAFFYYFKTATSRTTKNAANARTYFGLFKERLRVI
DVLVAPPDRVEAIVQFVALAGTLDTSADAVSGVIAIGFCAPNAASVLEAEQDWNLHRSMGITLAFYK
AQKSVSGYFDKGLHKEVAQTGLSEAGALTSNFLCGATARRCIPVVHRVIDLGEIEAALIEIDSAVKVADE
EQNLADQPTTRDNSVGAVGGVVLAGTMNMGANLDTVERGNNIIIELEEESYNAVNGKKAAGKLTDK
VLMVLSRAYFPVLEQIASANSLDRGVFGLLIDAPNDADNLDHGGDTVIPAPTIHTETTPLFAESDIERIA
WVAPIHLLGTLQAVLGFEPDQYSFISMMIDASLDITETAEVMLDYHVLEKGARPSFFRVVLIKGLHIRLF
AAVGTILVVGSAGRTEITAYIQT-AVPITLRKDTFLPAFAASSCIAIDIFSTMLRPPERSTVEMLILETVTEL-
LEVTRFENDKLERLDALLPGLDVADLMRLVAAAKDFSVIVTIDAAITAILSVAAEAVVLREMLLTLAEIAV
TALALALALLLMLTPDRGLTQKEVAREDQIKELIQGGTVLQVRAADKMVLASTADKVRLDTDEGTTSA
FASLNLATVSDGFYVLSSAVLQMSSDLMMIVAVARVLADKETKNALVVRHPEQVKTQATYAEGSPG
MVLQTLTDCIPPLAIKPEDVELLWTLVAALWRVKAHLANAARPYEERVLVLLLALSLIAVLLQSEGGGM
GGGA-
SSAAKILAAFSVLTIKASVEDEVRKVARRVMDLNSRLGRPEESVAPEFRGAELEQVKYEAENVEVDCME
TVRRFVDIPFLVSTRTQHNESEDRGARLVLDVTRVRFIDL-
VTKRSEIILASRAETDGIISREMAKDLQALHPEAAALGKIAALQHFIAINDGHISNYVTAAINSVLGMLLL
VLFINEVRVSKVVWPTRREVTMVFIMAALAVFFIVIGLGVQIVFMLSGISWQQITTLVLGEGIMVVRQT
STFPADA-
IGILQNTYADLICAGRVTKADIEASLHIVVGARVARGTWYVIYHYEITQAQQQSGIMQRAFRQATLSQ
SLIDGRMEVKIKVVTSDRYA-
THEIKGGQHRKGTRYNRVKVGHNIPVDVLARVKMAFGPPNLVLVKTFGAVVQKRFASPLTHMRHGA
VTIGPMEVSSPYITSKKARHAPGDSAVDLKPDNIVRSVAVVPELIWARENEDNGAVHDKYVKPTHKT
QAKRNTAVDKAQYDKRNQVELSFIGLAVKVLPDQAIVDATSGTLLPTESEAVTEGLKGVAATIRLDPM
EKALIAYIKRMRPQLNLAGKTDHKIVKCLNNNLDDVEYGKLGRRKIKPLTIKSVYDVEHRRATELVKLIPY
KDDVESRKSQFAEK-
LALFEPLKAFMMVFGFIARAIYALYLRVIRRTIVLAKTKVIVNAATVLSAIKIIGVSIRLRHKTTNVADTNH
HIKTIDARARMTLAGTGNVKLNLHPGRVGTKGGRIKGYMPYKNFNIFDGLASGRRAKVKELAKLKTVD
KTISVDGIRTKQQGGRIPQAALSNAIANTRVGVDIVGEDEQEESNPRANAEMGQIFAEIDARVYHLAA
AMIGHALEAVLAVVSIWGIGQAHVLLAIKLNINAGIELHSAA-VEEAVAAA-
FAQSGGEVSFIADVSEAYLGALSVSTESLDVCRLRRRSLSELKKTKKEAYMVLTCFGGKGDKVKIVEATQ
TQAYEIVATRTAERHGIKAKGTYGKERAMMQITPTKPVKVWVPVFVAEPELLAPVRAFAETNGDGVT
VVKLVKDIKVTMFEKAVASLSVSGKCTTIVYTVLEKVTELMKDIIYEQRAKAHYGRGRLRSHVVTHSKQ
KRHLSMIRRTVVADPSMDAARLVDGEVAVITGATMAFVMSEQDAV-
PGDVDMHYHEEPRREATLGIAGPETFLTTEQPRVLALLFLRAYLIHIVDIGTQGGFADAATIYDILRAMQ
RKLDRRLSAEIAKVRMCLADDVTEMGVTEVAARRETAGDPILSAYFINGVTANADIILLVANLECKVVP
ENIDCAIRLFYQRITLQAKERGGDVSIAIVPAQNQIEAAEGTVIYSIKGNNPSTSQMKLNVEAISRSIVKS
LATQLYQVTTFLNDTWIDTLLRAAQSVVFVDAVEGKEAVDHVIERDTELEFAPQKDIVSRKIRIYTPAAP
SAVVIWDPAIEREDKADAQGTIIKIYGPNLQDINCFTDIGAEPKIESLKAWDEKEKYVVHRDDIPEDITTL
VGKSSKVNKEFAIPDVAVVAPAKPGVTESIDRGEPKAGSCGEVANMSYPQESLVNTVSSANVQKNIS
MVLGGHPVGEMGISREIERHLETSMCEIFVDKDDVDVALDFYRLELTPLRVGLDPPNAKVEVDGKESR

KLMAVLDGEATLQRHPKVSQESGLTRNAGEKARMPDDTLMRDGVQHGDVGFTSTDLPDGRV-
VTPVFNDVKAQLKIDKVILRTFNVVTLSFSNNKGEEHLIKLRLNALKKSTRHYEMLLKIRETECAESPHAI
SVNKVLKIDALLQTICTAHYKAIEPLMADDLTALGVMGDQGLLHVVPRLMILAVLLAWGVEAIALVET
LEFQRDALVTDVTMGLVFRARDWSY-AISEVVGNTFFARTADDDTEQRLHARNPI-MIL-
YGKEGML-
ISPSGLKLKSTADEAQEHGNADAMVLSTERLQDAMEYFVLHVLLVFVDEVILAYGSGDEPLRFLVAEQ
AVTGRDNPGMVCLTRRTFPLDLQRDAVFVLVFEDSTMKNLSHKKMHMNLVLIKSKGWVVNVSTKILI
SDQAIASYAEDAGAHTQPSESALAVITSRVMAPIVLVVSQKPTAPSKTCFLEALSLLEEVSATVLNKPKK
YYIFEGDVISVLKGLSASVILGMQRPQPDRMRRAVVASAIRLAIAVKASGILTSLEHGLVNFSRAVSLAA
GRYPEQEGLPVAIMT-TALAFRVRNHKVKTKRARPYKGHTKFLHKADLDKSLIADGVDTLA
>GCA_000169415
AVIKPVIQDVYAPVLLIRQQEVATVTGAGSPELRAKTVFRKSGLQRKTSTKWVAKPFYF-
GVVKMGNIVQLVSISVDNRAVCTVAQLVEQEVVVRGLVALNGVKKKIAVEEIQGMTTEDPAKVVEFE
EEVDLKRFRDAADQADLLPVLLGLTLDVRFDPHEPVVHDLPVPVIFEVL-
GIMAERGGIEVDECAISTAVSDVDPIAYVSPID--
VVAGEKMVYIDQEAAVAAVKDEIALEELALESQVLVVKI-PLLLPV-FGEEGAN-
MSFANRSRLLGSIVGLAVGYTPITRAR-ETGIVAEM-
MDAAQAAALAEKRGTAEEIRIVKRMSKSAGLADDPDLVLLEDASEGKANWAGQIYDDPISGSSESDS
LGNIVYAANKRQSFARSITFIRKVEISDQAEAPLMRGVKHNSKSRAARKVSDGIAILNFPNVPHGLALR
AEQDKSADTLLHDATEGEGILLLIVKKFVTWKTAAIICLPSTAGDIVGLITLGGIAAIGRPDGLLNRHHKL
NGSLIEQKRIMDKLTLRMRASLKEKLGAESVIILVVKVAGDLVADATVQM-
GGVVGAVVAVQGELIFVRRHRQTLITDSESTV-ILRGVAEGPADAQALLRIRVDHTLVELSVLLIPE-
PVLVDSVSIREGVGKTLQIIQLDVYLLRDMGTGEETLAESLS-------------------------------------
----
GRIYEIDGDLRELPKSVKSLIGLVDAKAPKVLSAGYLTGRLKIRYADCTVLESEADLYAYAEERKELLRMV
PVYIITEAKRRQLVIFALSLKEGHRRARAAIRVLLAAVLLESQAEQGTVHHQTPNVLLDNAEVVGSSVM
ISRQASLELKSVQKMDVVVVFPEFQEKMVVSLKFFCTDVAIFVIALIVTVGDIISREPDAAQEGGAGYGK
RIRGHSMLFALLATFERRNLDDA--
PSLFLGLGLAAPRYGVVYTTQPKKKQMPSEPDTDKVDGFELVNAIKEDGRSNHELEMPEAMALAVE-
SAPINLT-------------------------------KWNISPLDALDLTLSGARPVRATE-
IMLADLTAALQMKKVRTLYLLGASNAELEEDRLGGAGNSTADVAADRQRTILLVHPVLVVIQDSNAA
GLVKNGGVEEWGTMAFKKHYAYAARMRLHMRTIDAIEVGVEMEPNKQKSEKVKTVKLEGDTRFRQL
AVEGVKEQMP-HHALNPDFAV-LLVLVTPAVGQVAADAGVLGARVPVPFSL-
RELQKSQSGQALPISAIRLLAMRAARAMIDEDIFVAGKVAA-
LLKIGALETVIGLILRNDTEAWGPWRLSVAEKLEADTKRASGFAARPFMTKPSLCNTQAHANVAEPIQE
IAAGYVATEKKFVFPVEGIAIQLILSSPHSRVGSAKRAGEGHLHEMAKELLAKAVLMLQNQPATTTEGL
LTYMTTNQPTDLLFPDSLDVIIDMRRMSVV--
VQLNLV-
HVIIPGTRELGDRTIMAAAADSGLVQVEQIDERTGRERTKMWMYGAMEELWLLSLKWADWVVDCAI
GAIILHVYAQDINRAFSLTRLQLEGWPPEATRWWNTWQLWLNKDGFFSYEAAFRAMAQVLKAELLD
YEMSDVEDVGRYQSRQELRVGYRIRGQVEFSRLDPRTDILLQAKTRLSVLRHALEVVAILVLREVVGEF
SLVVAMEDVAMGHVECVLAESFDFTEKNAHIGPVGLRTPAPIFGDGTVLKEALLGKIDLANTIAEFRYR
MITDGGRKLGVTWVTLQNMLYARIWLPPAPAALKDLLPICFFLSHPELEL---------
ISKARADQLMAGALVEDILEPVAIFADPEATTHQTHKEKHFKYYGEEVDPVHLAYILPLFALVATQVTV

SDRLLKDRYVLNLKIFRGFTSLLWMAAQLGEKSPAATIITVGTIAGLGALVLFIILL-
RDSVLLETMYYRYVYV-MRGYQAAKDVADADPEHLEPLAAVSRDVEWIEIVPAFFYYFDTAT-
FEPFNTAVARDFFGLFAERLRLVEVLVVPPDRVVELVEFVALAGTLSTSSEALPGIIATGFCEPNAAAVLE
ADQDWNLHRHMGITLAFFKAQESVHGWFDKGLHKAVAETSLTEAGGFTTRNDCRVEAKQCVPAVH
RVLDLGEIEAALIEVDSAVVVADEEQTLADKPTTREKSVGCVGGVVLAGTMNMGGSLDAVERSGNIII
ELEEEQYHAVNG-
HKDDKLTDKVLMVLSAALFPVIARFGSANSLERGVFGLLIPAPSDADNLDHGRSMSIPAASVHAETHP
LYAQADIDRIAYIAPIHLLGTLAAVLLFEPDHYSFISMMIEASLEIMKTAEVLDDYHVLDRGSRPPAFRVV
LIRGLHIRPLSAVSTILVVGSAGRAEVTAYIQGRAVPITLNKHTFLPAFAASSCFVIDLFSTMLRPPERSSV
QMLILETVAEL-
LDVTRGENDKLEKILVLLPGLDMAALLGLIAASDDFAGIVTLAAAITAILSVAAEAVVLRELLLTKAEIAVT
SLALALALLLMLTGDQGLTQREAAREAQMKELIQGGAVLQLHAADRMVIAATADRVRLETDEGTTSA
FASLNLATVSDGFYVLSSAVLQMSSDLMMIVQVARVLADKEQKNALVVRHPEQVKTQPTYAEGSPG
MVLQTLTQMVPPLAIKPEQVELLWTLVAALWRAKAHLAEAMRPRRERILILLLALGLIVVLLQNEGGG
MGGG--
GAPPKVLAAFSILTIKASVGDEVRKVTRRVMDLSNRLGRPEEAVNPEFRGEELDQSKYEAENVEVDCLE
TIRRFVEIPFLVSIRTQHNESEDRGARMVLDVTRVRFIDL-
VTKKSEIILVERAETDGIIARGMAKDLQALQPEVAALGKIAALQHYISLNDAHISNYVTAAIHSVLGMLL
LVLFIQQVRVAKIVWPTRREVSMVFIMASLAVFFLVIGL--
NLVFVVGGISPLQITTLVLGEGVAAVRQVSTFPAES-
VTILQNTYADLICAGRVTKADIENSLHIVVGARVARGTWYVIYNYEITQAQGEAGIMQRAFRQATLSQ
SLIDGRMEVKVKVVTSDRYA-
TYEIKGGQHRKGTRFLRVKVGHNIPVEILPRIKLAFGDPNLILVKTFGAVVQKRFAQPLTHIRHGAVTIGP
MEVASPYISNKKARHAPGDSVVDVKPDNIVRSVAVVPELIWARENEDQSAVHDKYVKTTHKTQAKR
NAAVDKAQYDKRQQVELSFIGLAVKVLPDQAIVHATAGTLMPTEAVAVTEGMKGVAATIRLDPMEK
ALIAYIKRMRPQLNLAGKTDKKIVKCLNNGLDDVEYGKLGRRRIKPITIKSVYDVEHREATELVKLMPYK
DDVEGRKSQFAEK--
ALFEPLNAFMMLFGFIARAVYALSLRVVRRTILLVKTKIVVNANTVLSAIKIIGIAIRLRHKSTNIADTKHHI
KTIEARARMTLAGAPNVKLNLRPGKIGKKGGRIKGYMPYKNFNIFDGLSSGRRAAVKELAKLKKVDKEI
QVEGIRTRQQGGRVPKADLSNAIANTRVGVDIVGENEQEDSNPRANAEMGQVFAAIDARVYHHVA
ALVGHALEAVLAVVAILGIGRAHSLLAVKLRVNAGIELHSAA-VEQAVAAA-
YAQNGGEATALADASEAYLGALLLSAESMDVLRLRRRQLG-
LKKTKKEAYMKLTCFTGKGEKVKIVEASQTQAYEIVATRTAERFGIKAKGHYGKERAMMQITPTKPVKV
WVPVFVAEDELLAPIRAFAETNGDGVTVVKLTTDVKITMFEKAVASLAVAGKSSTVVYTVLEKVTELSK
DIIYEQRSKCHYGRGRLRSHVVTHTKQKRHLSMIKRSVVAKPRMEAARLTDGEVSVVTGATMAFVMS
EQDAV-
PGDVDLHYHKEPRREAGLGIAGPETFLATEQPRVLALLFLREYLIHIVEINTTGGFNDAATIYDILRAMQ
AKLDRKLSAEIAKVRMVLHDNVADMGIHQEAPRRDKAGDAILSAYWINGVTANADIILLVANLECDC
VPENIDCAVRLFYQRVTLQAKERGGDVSVALIPAKNQIEAAEGTVIYTIKGEKPGTVQMKLNVEAISRSI
VKSLAVQLYQVELFVNDTWIAVLLRAAADVVYVDAVDGKEAVDHVVERDTELELAPQKDIVRRKIRIY
TPRAPSAVVIWDPAIDREDKADAQGTILKIYGPDLQDISCFTDIGAEPKIESLKAWDEKERYVVHRDDIP
PDITTMVGKSSKVNKEFGIPDVSVCAPSKPGV-
ESIDRQEPKAGSCGEVANLPYPQESLVNRVSSANVQKNISMVLGGHPIGEMGISRGAERHLETNMCE
ILIDCPDIDVAMNFYRLRLT-LRVGLDRPNAKVTIGGKESRKLMAVLDGDAPLQRHPKASREAGTR-

AVGAKAKLPDDTLIFDGVLHGDLSFTCTDLPEGRV-
VTPVTIKVKAQLKIDKVILKTFHVVTLSFSNDKGDEHLIKLRLNALKKSTRHYEMLLKIRDTECAESPHAID
VGKILKIDKLLQTICKAHYKAIEPLLADELTALGVRGDQGLLHIVPRLMILAVMALWGLEAEMEWPHW
GAMAERLVTDVTMGLQMRDRHFSY-CVSEVVGNTFFARTKDDDTTDRLRARNPI-VLL-
WGKFAMV-
ISPSGLRLDSTAPAETGHGHAGPLVLSAERLQDAMSYYVLRVLLVFVDHVIIAFGSGEDPIRFLVAEQA
VTGKENPQLVCLTRRTFPLDLQRNATFVLVFEDSIMKNLSHKKMHMNLVVIKPKGWIVNVSTKILISD
QAIASYAEDAGAHTQPSESALAVITSRVIAPIVLVVSQKPTAPSKTLFLEALNLYDDITCTIQNRPKRYYIF
EGDLIAVLGAIDAAVLKGMQRPQPDRLRRAVTAAAIRLAIASLSSGLLAALEAGAINFARAVTFVR--
YIQPTAAR---------AFRVRNHKVSTKRSRPYKAHPKVLMKND-AKKLIAEGVDTIA
>GCA_000169435
AVIKPVTMDTYAPVLMIRQQQVATVSGAGSPTLRAKTVYRKSGLQRKAETKWVSKDFYY-
GVVKLGNIVQLVSISVDKRSVCTVAQLVDQEVVVHGMVALEAVKKKIALEAIEGMTAEDPAKVVDYA
TEIELRKFRDTDDRADMLPVMLGLTLKIKFDPHQPLVHDLPIPVVFEVV-
GVMVQRGQIEVDECGVSTALSDVDPIVYVSPIDLAAVAGDKMAYIDQEAAVTAAKAEIELEELALESK
VLVVGI-PLLIPV-FAADGDV-MSFANRSRTLKTIVNLAVSYPEIKRARLD-NIAADM-
MDASQAAALAEKKGTAEEIRIVKRMQKSAGLADDPELVMLEDAAEGNSNWAGQIYDDPLSGSSESD
SAGNIVFAANKRQNFARSITYLRAVEISDQAAAPLMRGVKHNAKSRSSRKVDDAIAVLNFPNVPEGIA
IRAEAGKTQDILLHDATEGEEILLIIVRWHVTWKTAVIISLFPPANNIVGIIEMGGIGAVGRKDGLLNRHH
KLNGSLIEQRRIVEKLTLRDRAALKQKIGKEQIIIFVVKVAGDLVAKAKIQM---
SVGTVVAIQGETIFVRRHRQTVIADQEATV-VLRGVAEGPADAQALLRIRVDHTVVKLSVLLIPD-
PVLTESVTIREGVGKALQIIQLDVYLLRDMGTGDETLAKSKS--------------------------------
----
IAILKNITEPRMANLPAGVCRTFINAGPRAAVGMIFLLLGDAGKIYTIDGDLRENTKSIKSMIGLVDAAKP
KTLSAGYATARLKIRYADVMVLTSDADLYAYAEERRELLRMVPVYITNAAKRRQLIIFALSLKGGHRRAR
AALGVMIAAAILESQAEQGPVNHKDPEVLLDDGEIVGSEIMISRQAALDIKQIIKTDIIVIFPEFQEKMVV
SLKFFCTDVAIFVIALICTVGEMLSREPSHAQEEGAGFQKRIRGHSVLFALAVTFDRRNLRD--A-
QLFLDLGLAPPRYGIFYTEQPKRKQMPTEEDKKQIDGFDLVAAIKEDATKNHELDMPEAMALAVE-
DGTINVNAGVLAMTGDMAAVEKVDSKRKMALAVEKWTIASVALIDINMSGAQPFRACG-
IEQADVSATLPKKQI--
LFLPGANNAEMEEEKLDGAGSSTAEAAEDKQLGILLVEPVLVVIQDSNTASLIANGGLEEWGTMAYKK
HYAFAARMRLHMRTID------EMDPNKQKAEKIKTVKLDGDTRFRQLATEGVKEQMP-
LHALNPDFAVLVPVLVTPAVGRVAADAGVLGARVPCDASL-
RELQKSMKGNALPISGIRLLGMRAARAMVDEDIMVAGKVAA-
LLVIVALETITHLVLRNDSEATLPWRLSVAERLEADTKRAGGFAARPFNSKPSLCKTQAHAKIADPIQEI
AAGYVAIEERFVFPVEGIPIQLILASPHSRVGEAKRAGLGHLHDMAKEAQAKAVIMVLNEPDTNTEGLL
NYLTTQQPTDLLFEDCLDVVIDMRRMSVV---
VQLNTV-
HVLVPGVRDLADREIMAAAAHSGLVQVERIDERTGRERTLMWMYGAMEELWLLSVTYTDWVTDCA
IGAILLHVYAQQINRAFSLERLQLAGWPPEATRWWNTWQLWLNKDGFFSYEAAFRAVKAVLKADLD
DFDMSDVEGVGRYQSRQQLRVGYRIRQQVAFEQLDPRTDILLQSMTRLSVIRHALDMVAILIVREVV
VEFT-
VVAKEDVAMGHVECVLADSFDFTAKDADIGPVGIRTPDPVLGDGTVMKEAVLGKIDLANTVADFRYK
MLVDGGRDLGVSWVTLQNLIYPRIWVPPAQAALKNLVPIAFFLGHTELNLAIFPTLWAGISKARANKL

MAGALIEKILEKVAAFADPEATTHQSKKLRHFKYYGEEVDPVYLAYILDMFSLVATQVTVSDRLLKDRY
VLNLKIFRGFTSLLWLSAQIGDKGPAATIITVQTIAGLGALVLFIILLMRVVVLVETLYFKYVY--
MLGYQAAKDVADADPEHFLSLAHASRDVEWIEVVPAFFYYFKTATSRLSKNTALAKEFFGLFKERLRIID
VLAVPPDRVEALVAFVALAGTLATSAKVYPGVIACGFCEPNAAAVLEADQDWNLHRSMGITLAFYDA
RESVKGWFDKGLHKDVAATGLREAGALTTPFYAGATAKQCVPAVHRVLDLGEVEAALIEIDSAVVVA
DEEQSLADKPTTRENSVGAVGGVVLAGTMNMGANLDSVERGGNIIIELEEEQYSAVNGKKTDGKVT
DKVIMVLSQAYFPVLKRLASANALDKGAFGLLIDAPDDADNKDHGRELTIPAPTVHAESAPLYAESEIE
RIAWIAPLHLLGTLDAVLGFEPDQYSFISMMINASLEIMETAEV--
DYHVLETGSRPPGFRVILIKGLHIRLLAGVCTIIVVGSAGRAEVTAYIQG-
AVPITLNKSTFLPAFAASSCHVIDIFSTMLRPPERSAIEMLILETVAEL-
LEVTRIENDKLERMAALLPGLDIAALLSLVAAAKDFAAIVTIDQAITAILSVAAEAVVLREMLLTLAEIAVT
ALALALALLLMLTHDQGLHQKEHAREAQMKGLIQGGVVLQVKAADRMMLASTADKVRLDTDEGTT
SAFASLNLATVSDGFYVLSSAVLQMTSDLMMIVAVARVLADKETKNALVVRHPEQVKTQSTYAEGSP
GMVLQTLTGAIPPLAIKPDTVELLWTLVAALWKVKAHLANAARPHIERVLILILALALITVLLQSEGGGM
GGG--
GPAAKALAAFSILTIKAKAGDEVRKVARRVMDLNSRLGRPEEAVNPEFRGAELEQSKYEAENVEVDTM
ETIRRFVDIPFLVSTRTQHNESEDRGARMVLDVTRVRFIDL-
VTKRSEIILAARAETDGIIAREMGKDLAALHPEAAALGKIAALQHFIAVQDAHISNYVTAAIHAVLGMLL
LVLFIQEVRVSKVVWPTRREVTMVFIMAALAVFFLVIGLGVQLVFMLSGISWNQLTTIVLGEGIMVVRQ
TNTFPEDS-
IGILQNTYADLICSGRVAKADIEASLHIVVAGRVARGTWYVIYLYEISQAQDQSGIMQRAFRQMTLSQ
SLIDGRMECKIKVVTSDRYA-
TYEIKGGQHRKGTRYNRVKVGHNIPVQVLPRMKMAFGPPNLVLVKTFGAVVQKRFAQALTHIRHGA
VTIGPMEVSSPYIGNKKARHAPGDSVVDLKPDNIVRSVAVVPELIWARENEDNGAVHDKYVKTTHKL
QAKRKAAVDKAQYDHRNQVELSFIGLAVKVLPEKAIVHATSGELMPTASVAVTEGMKGIAATIRLDP
MEKALIAYIKRMRPQLNLGGQTDKKIVKCLNNNLDDVEYGKLGRRKIKPITI--------------------
----
SAFMMLFGYIARAVYALYLRVIRRTILLVKTKIVVNASTVLSAIKIIGVSIRLRHKSTNVCETNHHIKTIEKR
ARMTLAGTGKVKLNLSPGKIGKKGGRIKGYMPYKNFNIFDGLASGRRAKVKELAKLKNVDKSITVDGIR
TKQQGGRTPKAQLSNAI-
NTRVGVDIVGENEQEESNPRANAEMGQIFAAIDARVFSHVGALIGHALEAVLAVVAIWEIGRAHNLL
AIRLKINAGIEMHSAA-VEVAVAAAAFAQNAGEASVIADISESYLGTLAISATSVDVSRLRRRSLNE----
----
RHGIRAKGTYGKERAMMQITPTKPVKIWVPVFVSEPELLAPIRAFAESTGDGVTVVKLTTDVKVTMFEK
AVASIAVTGKATSIVYTVLEKVTQLMKDIIYTQRAKAHYGRGRLRSHVVTTTKQRRHLSMIKRTVVADP
SMDAARLTDGEVAVITGATMAFVMSEQEAV--
GDVDLHFHEEPRREADVGIGGPETFLTTEQPRVLALLFLRAYLIHIVEIGTTNGFSDAATIYDVLRAMQN
KLDRKLSADIAKVRMVLTDNVTDMGVTDLAPRREAAGDKILSAFWINGVTANADIILLVANMETHCV
PENINCAIRLFYQRITLQAKERGGDVSVAIVPAANQIEAAEGTVIYSIKGQTPGTGQMKLNIEGISRSIVK
SLAVQLFQVDTFLDDTWIATWLRAALDVVFVSAEDGKEAVDHVVERDTELEFAPQKDIVARKIRIFTP
AAPSAVVIWDPAIEREDKAEAQGTILKIYGPNLQDINCFTDIGAEPKIESLKAWDEKEKYVVHRDDIPED
I-
TLVGKSSKVNKEFAIPDVAVCAPSKPGVTESIDRGEPKAGTCGEVANMSYPQESLVNAVSSANVQKNI
SMVLSGHPVGEMGISREIERHLETSMCEIFVDKADVDVALDFYILDLTPLRVGLDPPNARVQIDGKESR

KLMAVQDGDADLQRHPKVSQATGTGLKAGIKARMPDDALIVDGVMHGDISFTATDLPDGRV-
VTPVFNEVKAQLKIDKVLLKAFHVVTLSFSNDKGEEHLVKLRLNALKKSTRHYEMLLKIRETECAESPHAI
SVGKVLKIDALMQTICQAHYKEIEPLLADELTALGVMGEQGLLHIVPRLMILSVLHLLRLEIALQIDPWG
LQAAAMVTDQTMGLYCRDRHWSF-AISEVVGNTFFARTKEADTEQKIRARNPV-VLL-LGKFNMI-
ISPSGLGLTSTAASPQGRGNTEELILSTERLQDEMGYYVLQILLVFVDEVILAYGSGDEPIKFLVAEQAVT
GRQNPGMVTLTR-
TFPLELQRNATFVMVFEDCTMKNLSHKKMHMNLVVIKSKGWIVNVSTKILISDQAIASYAEDAGAHT
QPSESALAVITSRVMAPIVLVVSQKPTAPSKTLFLEALSLYDEVTSTVQNKPKKYYIFEGDVVAVLETLDA
AVLLGMQRPQPDRMRRAIVAAAVRLAIAIRASGIVTRLEENLINFARAVNFAALYYQLGALI--------
--EFRVRNHDVSTKRSRPYKAHPKFLHKADVDKSLIATGVDTLA
>GCA_000169455
AVIKPVEMSKYAPVLMIRQQQVATVSGAGSPALRAKTVFRKSGLQRKTSSKWVAKPLYF-
GVVKMGNIVQLVSISVDNRAVCTVAQVVEQEVVVHGLVALEAVKRKIALEEMDGMTTEDPAKVAEF
EAELDLKRFRDADARADMLPVLLGLTLEVRFDPHEPVVHDLALPVIFEVI-
GIMADKGGIEVDDCAISNAVSDVDPIAYVSPIDLEAVAGTEMAFIDQEAAVAAARETIGLEELALEAGIL
VVGIKPLLLPV-FG-SGDV-MSFAARSRTLSSIVALAVAYPELTRA-IE-AIASEM-
MTAAQAAALAEKRGTAEEIRIVKRMQRSSGLSDDPELVMLEDAAEGNSSWAGQIYDDPISGSSESDS
VGNIVHAANKRQNFARSITYLRKVEISDKAAAPLMRGVKHNSKSRAARKLGDEIAELNFANVPPGIAIR
AAAQKPADRLLHDATEAEEILMVVVRWHVTWKTAVAVLLLFLAGDIVGLIELGGIAAVGRPDGILNRH
HKLNGSLIEQRPIIEKMTLRLRAALKEKLGAEQIIIFVVKVQGDLVADATIQMG-
GPVGAVVAIQGDVIFVRRHKQTVITDQEATV-VLKGVAEGPADAQALLRIRVTHTLVEISVLLIPE-
PVLVENVSIREGVGKALQIIQLDVYLLRDMGTGEETLA----
RELIREVLMLDPITGVRLAYGKLKERIGKKLGMKDLL-----
RSILAAVTEPRMENLPDGVCRSFITAGPRAAVGMLFLLLDEAGKLYQIDGDMRELTKSVKSLIGLVDAK
APKVLSAGYAEERLRIRFADCEVLNGEADLYPYKIERREIMRMVPVYIINAAKRRQLVLFALSLKGGHRR
AMMALNVMLAAAIIESQAEKGKVHHEAPEILLDDAEVVGTAIMISRQASLDLKQILKIDVIVIFPEFQEK
MVVSLKFFCTEVAIFVIALITTVGEMLSREPEHAQEEGAGYQKRIRGHSVLFALEVTFDRSNCKRNALPD
VFLGLGLADPRYGILYTNQPKKKQMPSEPDGDRVDGFELVHAVKEDGSSNHELEMPEAMALAVE-
DASINVSAGVLAMNGGMVDVEKVDSKRKVALAATKWNISKLQFVDVNISGAAAVRACD-
IEQANLSAALEHKKVWFLFLPGASNAELEEEKLDGAGASTADAAADKQIAILLVNPVLVVIQDSNSAG
LIANEGLDEWGTMAYKKHYSFAARMRLHMRTIDAIEVGVDLNPNKQKSEKVKTVKLEGDTRFRQLAV
EGVKEQMP-LHALNPDFAV-LPILVTPAVGSVAADENVLGAKVPVDASL-
RELQKSQKGSALPITAIRLLAMRAARAMIEEDIMVAGKVAA-
LFKIRALETITALVLRNDSEATLPWRLSVAERLEADTKRASGFAARPFKTKPSLCNTQAHAKIADPIQEIA
AGYVAAESRFVFPVEVVAIKLILSSPHSRVEEKKRGGEGHLHGMAKELLAKAVVMLRNEPDTNTEGLL
NYLTTNQPTDLLFEDCLDVIIDIRRMSVV--
VHLNTV-
HVVVEGVADVGDYEIMAAKFASGLVQVEQVDDRTGREKTAMWMYGAMEELWLNSVRWADWVP
NCAIGAIILHVYAQTINRAFSLRRLQLDGWPKEAARWWNTWQLWLNKDGFFGYDAAFRAVKQILKS
NQADFEMKDVEGVGRMQSKQEVRAGYRIREQVEYARLNPRADILLQAQTRLSIIRHALEVVAILILRD
VAPEFTLVVAYQDVARGHVECVLAESFDFTEKNAHIGPVGLRTPASIFGDGTVLKEAVLGKIDLANTVA
EFRYLFLLDGGRALGVEWISL-NMLYARIWLPPAPAALKNLLPICFFLQHPEIDLEWT-SLFD-
ISKARADQLMAGALVEEILEGVAKFAEPEATTHQSKKEKHFKYYGEEVDPVHLAYILDLFALVATQVVL
SDRLLKDRYVLNLKVFRGFTALLWMSLQLAMKGPSATVITVGSIAGLGALVLFIILL-

RDSVLVETLYYRYVYVVMRGYSAAKDVQDADPEHLGQLSAASRDLEWIEVIPAFFYYFKTATSMAREN
TAVVREFFGLFKERLRIVEVLAVPPERVAALVEFVALAGTLDTSATA-
VGVIAVGLCEPNAAAVLEAERDWNLHRSMGITLAFFEARDSVKGYFDKGLHKDVAEIGLREAGAFTT
HFQCDVDAKQVVPAIHRVLDLGEMEAALIEIDSAVVVADEEQTLADKPTTRDKSVGAVGGVVLAGT
MNMGANLDSVERGGNIIIELEEEQYHAVNGKKADGKLTDKVLMVLSAAYFPVIAAFASANALDRGVF
GLLIEAPTDADNLDHGREMVIPAPSVHTETTPLYAESDIDRIAWIAPLHLLGTLQAVLLFEPDFQGFISM
MIDASLEIMETAEVLDDYHVLEKGARPPKFRVILIKGMHIRLLAAVGTLLVVGSAGRSEITAYIQGQAVPI
TLRKNTFLPAFSPSSCKAVDIFSTLLRPPERSSVEMLILETVAEL-
LEVTRGENEKLERLLVLLPGLDLASLLTLLAAAEDFSDIVTIGAAITAILSVAAEAVVLREMLLTKAEIAITAL
ALALALLLMLTGDEGLTQKEQPREAQMKELIQAGAVLQVHAEDAMVLASTADKVRLDTDEGTTSAF
ASLNLATVSDGFYVLSSAVLQLSSDLMMIVAVARVLADKESKNALVVRHPEQVKTQPTYAEGSPGMV
LQTLTECIPPLAVKPEEVELLWTLVAALWRVKAHLASAARPHIERVLILLLALGLIVVLLQSEGGGMGGG
-
MGSAAKALAAFSVLTLKASVGDEVRKVTRRVMDLSSRLGRPEEAVNPEFKGEELDQTKYEAENVEVD
CMETVRRFVDIPFLVSIRTQHNESEDRGARLVLDVTRVRFIDTVVTKRSDVILVERVETDGIIAREMAKD
MQSLHETAAALGKVAALQHFIALSDAHISNYVTAQTHSVLGMLLLVLFIQQVRVSKVVWPTRREVTM
VFIMAALAVFFLVIGLGLNLVFMVGGISPLQITTLVLGEGVMAVRQTSTFPAES-
VTILQNTYADLICAGRVTKADIEASLHIVVGARVARGTWYVIYHYEITQAQSTAGIMQRAFRQATLSQS
LIDGRMEVKIKVVTSDRYA-
TYEIKGGQHRKGTRYNRVKVGHNIPVDLIARIKMAFGPPNLVLVKTFGAVVQKRFAQPLTHIRHGAVTI
GPMEVSSPYITSKKARHAPGDAVVDIKPDNIVRSVAVVPELIWARENEDQSAVHEKYVKTTHKIAAKR
NRAVDKAQYDKRNQVHLSFIGLAVKVLPEQAIVHSTAGTLLPTESVSVTENLKGVAATIRLDPMEKALI
AYIKRMRPQLNLQGKTDNKIVKCLNNNLDDVEWGKMGRRKIKPITIKSVYDVEHERATELVKLMPYK
DDVEGRKSQFAEK--
ALFEPLKAFMMMFGFIARSVYALSLRVVRRTILLVKTKIVVNANNVLSAIKIIGVSIRLRHKSTNVADTKH
HIKTIEKRARMTLAGTGSVKLNLRPGRVGKKGGRIKGYMPYKNYNIFDGLASGRRAKVAELAKLKKVD
KAIAVDGIRTRQQGGRLPKADLSNALSNTRVDVEVVGENTQEASNPRANAEMGQIFAAIDARVYSLV
AALVGHALEAVLAICAILGIGKAHVMLAV-LKMNAGIELHSAA-VAQAVAAA-YAQAGGEA-
AIADVSEGDLGALAVSEQSLDVCRLRRRQLG-
LKKTKKEAYMSLTCFSGKGDKVKIIEATQTQAYEIVSTRTAERHGIKAKGTYGKERAMMQITPTKPVKV
WVPVFVNDDELLAPV------
NGDGVAVVKLANDVKITMFEKGVASLAISGKATTVVYTVLEKVTELSKDIIYEQRAKAHYGRGRLRSHV
VTHTKQKRHLSMMKRTVVADPSMDAARLTDGEVSVVTGATMAFVMSEQDAV--
GDVDYHFHEEPRREAPLGIAGPETFLSTEQPRLLALLFLRAYLIHIVDIETVGGFADAATIYEVLRAMQN
KLDRKLSAEIAKVRMCLHDDVEDMGVTAEAPRRDAAGDAILSAYWINGVTANSDIILLVANMECKTA
PENIDCAVRLFYQRVTLQAKERDGDVSVELVPAQNQIEAAEGTVIYTIKGNNPSSPQMKLNIEAISRSI
VKSLAVQLFQIDLFLNDTWIATLLRAAQEVVFVDAREGKEGVDHVVERDTELEMAPQKDIVARKIRIYT
PAAPSAVVIWDPAIEREDKADAQGTILMIYGPDLQDINCFTDIGAEPKIESLAAWDEKEKYVVHRDDIP
EDITTLVGKSSKVNKEFGIPDVAVCAPAKPGVTESIDRGEPKAGSCGEVANLPYPQESLVNNVSSANV
QKNISMVLGGHPVGEMGISREVERHLETNMCEIFVDKDDVDVALDFYKIKLTPLRVGMDKPNAKVEI
DGKESRKLMAVKDGEAQLQRHPKVSEESGTRIGGGEKAKLPSDALVYDGVQHGDLKFTATDLPEGRV
-
VTPVNMAVKAQLEIDKVMLKTFHVVTLSFSNDKAEEHLIKLRLNALKKSTRHYEMLLKIRDTECAQSPH
AIPVGKILKIESLLQTICTAHYTAIEPLMAEDLTALGVMGDQGLLHVVPRLMILAVLHLCPIEAKWVLRI

WGLQQHAILTDQTMGLPVRARSWSF-AVSEVVGNTFFARTKDDDTEQRLHARNPI-MIL-
YGKEGML-
ISPSGLKLSSTQTPEADHGNDEQLILSTERLQDSMSYHILAILLVFVDEVILAYGSGEEPLKFLVANQAIT
GRDNPQLVCLSRRTFPLDLQRDAVFVLVLEDTTMKNLSHKKMHMNLVLVKPKSWVENVSTKILISDQ
AIASYAEDAGAHTQPSESALAIITSRVIAPIVLVVSQKPTAPSKTLFLEALNLYDEITATVQKRPKRYFIFEG
DVVAILGTLDAAVILGMQRPQPDRFRRAVVAQAIRLAIALRASGVLCELECAQINFSRAVSLAQ-
VYPGLEA--ALAIT-SALDFRVRNHKTSTKRDRPYKGHTKFLHKAEAEKALVAEGVDTIA
>GCA_000170775
AVIKPVVMNEYAPVLMIKQTQVATVSGAGSPSLRAKTVYRKSGLQRKTLTKRVAMNIYY-
GVMKLGDIVQLVSISVDARAVCTVAQVVEQEVVVHGLVALAAVKKKIALEAINGMTTEDPAKVCEFD
EELDLRKFRDAEDRADMLPVLLGLTLTIRFDPHEPLVHDIPIPVIYEVV-
GIMADKGGIEVDDCAISTAVSDVDPLAYVSPIDLAAVAGEQMAFIDQEHAISAAREAIAL--
LALESTVLVVGIRPLLIPV-LADDGDN-MSFANRSRELGSIVDLAVSAERLSRAR-EQGIASDM-
MVRMQAEALSERRGTAEEIRIVKRMNRSAGLADDPELVLLGDAAEGNSSWAGQIYDDPISGSSQSDS
AGNIVVAANKRQNFARSITFLRKVEISDKATAPLMRGVKHNSKSRSSRKIDDAIAVLNFPNVPEGIAIRA
EGEKPADTLLHDATETEEILLLVVRWKVTWKTAIIIALLV-
AGNIVGIIELGGIAAVGRPEGLLNRHHKLNGSLIEQRPIIEKLTLRLRASLKQKLGKDQVIIFVVKVSGDLV
ADAKVQMG-GPIGAVVAVQGDVIFVRRHRQTLVTDSEATV-
ILKGVAEGPADAEALLRIRVSHTLVALSVLLIPQ-
PVLVDSVSIREGVGKALQIIQLDVYLLRDMGTGSETLAESSSRELIREVLMIDPVTGVRLAYGEIKERMGK
KVGLKDLETLF--
ISILEGITEPRMENLPAGVCRNFIQAGPRAAVGMIFLLLDEAGRVYTIDGDLRELPKSVKSLIGLVDSTAA
KVLSATYATERLRIRYADCGVLVAEANLYAYKDERKELLRMVPVYIINEAKRRQLVIVALSLRGGHRRV
MASIGVMIAAAILESHAEMGVVDHAEPGILLDDAEVVGTAMMISRQAALDLKQIVRTDVVVVFPDFQ
EKMVVSLRFFCTEVAIFVIALITTVGDMLSREPNSAKETGAGFQKRIRKHAVLFALQVSFERRNCRDA-
RPDLFLGLGLADPRYGIFYINQPKKKQMPSEPDSDRVDGFELVHAVKADTSSN-
ELEMPEAMALAVEADAAINLAAGVLAFKG-
MTAIERVDSKRKMALATEKWNISRLDMADIHLSGAAQVRATG-IEQADLSATLEAKNM-
FLYLPGASNSALEEDKLNGAGASSADAAEERQTRILLVTPVLVVIQDSSAAGLITNGGLEEWGTMAYK
KHYAFAARMRLHMRTVDCIEVGIEMEPSKQKAEHIKTVKLQGDTRFRQLAIEGVKEQMP-
LHALNPDFAVLVPVLVTPAVGRVAADEAVLGARVPCEFSL-
RELQKSMRGAALPITAIRLLPMRAARAMIEEDIMVAGKVAA-
LLKIRAHETVVGIILRNDSEATVPWRLSVAERLEADAKRMAGYAARPFISKPSLCDVQAHANVAEAIETI
AAGYVAKDARFVYPVEGIKIRLILSSPHSRVGDAKRAGEGHLHEMAKELIAKAVMMLKSEPDTNTEGLL
NYVTTNQPTDLLFPDCLDVVIDMRRMSIV--
VQLNVV-
HVVVDGVKDVGDYEIMAAAAPSGLVQVERVDDRTGRERTIMWMYGAMEDLWLMSVRYADWVA
DCAIGAIILHVYAQTINRAFSLTRLQLEGWPQDASRWWNTWQLWLNKDGFFGYEAAFRAVRQALKE
ALFGYDMSDIEGVGRYQSRQQLRVGYRIREQVDFARLDPRTDILLQAKTRLAVIRHALALVAILILREVV
GEFTLVVAHEDVAMGHVECVLAESFEFTEKNAHIGPVGLRTPETVLGDGTVLQEAVLGKIDLANTVLD
YRYLFVVDGGRELGVWWLSI-
NMLYARIWLPPAQAALKNLLPICFFLSHPELDLLLGDLLFDGISKARAQQLMAGALIEEILEAVARFADP
EATTHQSNKERHFKYYGEEVDPVHLAYILDMFALVATQVVVSDRLLKDRYVLNMRV-
RGFTSLLWLSLQLAEKGPAATVITLGTIAGLGALVLFIILL-RESVLVETLYYRYVY--

MLGYSAAKDVADADPEHLLVLAAASRDVEYIEIIPAFFYYFDTAT-
FEPLNTAVAKEFFGLFEERLRVIDVLAVPPDRVEALVTFVALAGTLPTSAAALPGVIACGLCEPNAAGVL
EADQDWNLHRSMGITLAFFDARESVKNWFDKGLHKDVAETGLREAGGFTTTNLCGKEAKKCVPAIH
RVVDLGELEAGLIEIDSAVLVADEEQTLADRPTTREKKVGAVGGVVLAGTMNMGGALDQVERGGNIII
ELEQEQYHAVNGKKGDGKLTDKVLMVLSCAYFPVIRKMASASALDKGAFDLLISAPEDAENLDHGGE
LTIPVPTVHAE-
AVLYAESEIDRIAWIAPMHLLGTLPAVLLFEPDQYSFISMMIDASLEITQTAEVLDDYHVLEKGARPPGF
RTILIRGLHIRLLAAIGTIIVVGSAGRSEITAYIQGRAVPISLSKASFLPAFAASSCTVVDIFSTMLRPPERST
VQTLILETVAEL-
LDVTRLVNDKLERLRLLLPGLDAALLLGLVAEAEDFSDIVTISRAITAILSVAAERVVLRELLLELAEIAISSL
ALALALLLMLTPDRGLTQKERAREAQKKELIQGGAVLQIHAADAMVLASTADKVRLNTDEGTTSAFA
SLNLATVSDGFYVMSSAVLQLSSDLMMIVSVARVLADKETKNALVVRHPEQVKTQPTYAEGSPGMV
LQTLTDCIPPLAIKPEDVELLWTLVAALWRMKAHLAEARRPRLERVLVLLLALALIVVLLQSEGGGMGG
G-
MGAAAKILAAFSILTIKASVGDEVRKVARRVMDLSSRLGRPEESVNPEFRGEELEQTKFEAENVEVDCM
ETIRRFVDIPFLVSIRTQHNESEDRGARLVLDVTRVRFIDL-
VTKRSEIILAARAETDGIIARQMAKDMQSLHSEIAALGKIAALDHFISLNDGHISNYVTAEISSVLGMLLL
VLFIQQVRVAKVVWPNRREVTMVFIMATIAIFFLVIGLG-
KLVFMLGGISPLQITTLILGEGVMAVRQASTFPADS-
VTILQNTYADLICAGRVTKADIEASLHIVVGARVARGTWYVIYPYEITQAQGEAGIMQRAFRQATLSKS
LIDGRIEVKVKVVTSDRYA-
TYEIKGGQHRKGTRYNRVKMGHNIPVDLLARIKMAFGDPNLILVKTFGAVVQKRFAAPLTHMRHGAV
TIGPMEVASPYIRMKKARHAPGDSVVEIKPDNIVRSVAVVPELIWARENEDQSAVHDKYVKVTHKIAA
KRNAAVDKAQYDKRNQVHLSFIGLAVKVLPAQAIVDATAGTLLPTASVAVTENLKGVAATIRLDPME
KALIAYIKRMRPQLNLAGKTDNKIVKCLNNNLDDVEYGKLGRRALKPLTIKSVYDVESRRATELVKLMP
YKDDVEGRKSQFAEK--
ALFEPLKAFMMLFGFIARAIYALYLRVVRRTILLVKTKIVVNAKTVLSAIKIIGVSIRLRHKSTNVADTNHHI
KTIEKRARMTLAGGPSVKLHLHPGKVGMKAGRIKGYMPYKNFNIFEGLSSGRRNKVRELAKLKTVDKTI
TVEGIRTRQQGGRIPKADLSNAI-
NTRVGVDIVGENEQEASNPRANAEMGQIFAAIDARVYSLVAALIGHALEAVLALAAIQGIGKAHALLA
VRLHVNAGIEFHSAA-VDEAVAAA-YAQPAGEA-AIADVSEGDLGLTRINAASLDVCRMRRRQLG-
LKKTKKDAYMELTCFAGKGDKVKIVEASQSQFYEIVATRTAERFGIKAKGTFGKERAMMQIVPSKPVKV
WVPVFVSEEELLAPIRAFAETNGDGVTVVKLTKDIKVTMFEKAVASLAVTGKSTTVVYTVLEKVTELMK
DIIYEQRAKAHYGRGRLRSHVVTHTKQDRHLSMMRRTVVADPRMNAARLTDGEVSVITGATMAFV
MSEQDAV--
GDVDMHFHEEPRREAALGIAGPETFLATEQPRVLALIYLRAYLIHIIEIGTRGEFNDAATIYDILRAMHSK
LDRKLSAEIAKVRMCLHDDVADMGVRQEAPRRETAGDPILSAYWINGVTAQSDIILLVANLECKTAPE
NINCAVRLFYQRVTLQAKERGGDVTVALVKAQNQIEAAEGVVIYEIKGKTPGTIQWKLNIDAISRSIVKS
LAVQLFQVDLFLSDTWIATLLRAAQDVVYVDAKDGKEAVDHVVERDKAVEMAPQKDIVRRKIRVYTP
AAPSAVVIWDAAIERADKADAQGTIIKIYGPDLQDISCFTDIGSEPKIESIKSWDEKERYVVHRDDIPPDI
-
TLVGKSSKVNKEFGIPDVAVCAPSKPGVVESIDRGEPKAGSCGEVANLPYPQESLVNRVSSANVQKNI
SMVLGGHPVGEMGISRKVERHLETNMCEIFVDKPDVDVALDFYTLTLTPLRVGLDQPNAKVEIDGKES
RKLMAVLDGEAALQRHPKVSEASGTGT-AGSKARLPDDALIYDGVLHGDISFTATDLPDGRV-

VTPVANTVKAQLKIDKVLLRTFHVVTLSFSSHKGEENLIKLRLNALKKSTRHYEMLLKIRDTECAQSPHAI
PVGKILKIDALLQTICQAHYVAIEPLLADDLTALGVMGDQGLLHIVPRLMILAVLHLWGPTAVDIIPRW
DLPHRALITDVTMGLRLRARDWTYMAISEVVGNTFFARTADDDTEQRLHARNPI-VLL-YGKFGMV-
ISPSGLRLDSTAPSPDDHGNADALILSRERLQDAMGYFVLRILLVFVDESILAWGSGDEPLKFLVADQA
VTGRDNPGLVCLTRRTFPLDLQRDATFVLVFEDSTMKNLSHKKMHMNLVLIKSKGWIVNVSTKILISD
QAIASYAEDAGAHSQPSESALAVITSRVMAPIVLVLSQKPTAPSKTMFLEALNLLDDVTATVLNRPKRY
YIFEGDLISVLHTVGASVILGMQRPQPDRMRRAVVATAIRLAIAAL-------LEAGVINFARAISLAR-
GYPVPELL----------DFRVRNHKTSTKRDKPYKAHPKVLHKADSNKGLVAEGVDTIA
>GCA_000170875
AVIKPVSMKAYAPVLMIRQTQVATVSGAGSPSLRAKTVFRKSGLGRKTSCKWVAKPLYF-
GAMKMGKIVQLVSISVDARAVCTVAQVVEQEVVVHGLVALAAVKRKIALEEIGGMTTEDPAKVAEFE
TEIELQKFRDADDRADMLPVMLGLTLSIRFDPHEPLVHDIPIPVIFEVV-
GIMAERGGIEVDDCAISNAVSDVDPVPYVSPIDLDAVAGDKVAYIDQEAAVSAAKEAIALEELALEASV
LVVGISPLLLPV-LGEQGDI-MSFANRSRDLRSIVDLAVSAAPVQRA-I-AGIAAEM-
MARLQAKALAEKRGTAEEIRIVKRMQRSAGLADDPDLVIMEDAAEGHSSWAGQVYDDPISGSSESD
SAGNIVVAANKRQSLTRSITFLRKVEISDKAAAPLMRGVKHNSKSRSARKIDDAIAALNFPNVPPGIAIR
AAAGKPPDTLLHDATETEAILLLVVRWKVTWKTAVIVTLVFVAGDIVGLIELGGIAAVGKPDGLLNRHH
KLNGSLIEQRRIVEKLTLRLRAALKQKLGKEQVIIFVVKVQGDLVADAKVQMG-
GPIGAVVAVQGDVIFVRRHRQTLVTDQEATVQILKGVAEGPADAQALLRIRVGHTLVELSVLLIPE-
PVLVDSVSIREGVGKALQIIQLDVYLLRDMGTGSETLAESTSRELVRDLLMVDPVTGVRLAYGDIRERL
GKKLDLKDLET----
LSIIAGVTEPRMQNLPEGVCRDFIQAGPRAAVGMIFLLLDEAGRIYSIDGDLRELPKSVKSLIGLVDAKA
PKVLSATYATARLRVRFADCEVLQAEANLYAYKEERRELLRMVPVYIITEAKRRQLVILALSLRGGHRRA
RASIGVLIAAAIIESQAINGAIHHAEPRILLDDAEIVGSAMMISRQSALELKQVSKIDVVVVFPDFQEKM
VVSLKFFCTEVAIFVIALIVTVGEMLSREPSSAQEDGAGFQKRIRGHSVLFALAAVFERRNCDDAKI-
TLFLALGLAPPRHGIIYITQPKKKQMPSEPDADRVDGFDLVHAIKEDDKD-QELHMPEAMALAVE-
DATINLSGGVLALKGGMTAVERVDSKRKMALAAEKWTIARLPLVDIHLSGASLTRCTG-
IELANLSAALGRKKV--
LYLPGASNKELEEEQLDGAGNSSAIVAEDRQRQILLVHPVLVVIQDSNAAGLISNGGIDEWGTMAYKK
HYSFAARMRLHMRTID------EMEPNKQKAEKVKTVRLEGDTRFRQLAVDGVKEQMP-
LHALNPDFAVLVPLLVSPAVGCVAARAGVLGAEVPCAFSL-
RELQKSMKGHALPITAIRLLPMRAARAMIDEDIMVAGKIAA-
LLKIRATETIAQLVLRNDSEATLPWRLSVAERLEADTKRASGYAARPFMSKPSLCDTQAHARVADPIQE
IAAGYVAKEGRFVFPVEGIAIRLILADPHSRVGSSKRAGEGHLHEMAKELLAKAVLMLKNEPETNTEGLL
TYLTTNQPTDLLFEDCLDIVIEMRRMSVV---
IQLTTV-HVVVDGVRDVGDYEIMAAAA-
SGLVQTERVDERTGRERTLMWMYGAMEDLWLMSLRYADWIQDCTIGAIILHVYAQTINRAFSLKRLQ
LSGWPPDASRWWNTWQLWLNKDGFFGYEAAFRAACQVLKSALLDFEMQDIEGVGRYQSKQQLRV
GYRIRQQVAFARLDPRTDILLQAKTRLSVIRHELDVVAILILREIVGEFT-
VVAQEDVAMGHVECVLADCFDFTEKNAHIGPVGLRGPETILGDGTVLQEAVLGKIELANTVAEYRYLF
VIDGGRALGVKWLSL-NMLYPRIWLPPAQAALKNLLPICFFLSHPEVDLGVW--
LFAGISKARAEQLMAGALIEEILEGVARFAEPEATTHQSKKERHFKYYGEEVDPVHLAYILDLFALVATQ
VTVSDRLLKDRYVLNMRVFRTLTSLLWLSAQLAEKGPAATIITVGTIAGLGALILFIILL-
RASVLVETLYYRYVY--MLGYTAAKDVADADPEHLLPLSSASRDVEWIEIIPAFFYYFKTAT-

FEPFNTAKAREFFGLFEERLRLIDVLAVPPDRVEELVDFVALAGTLQTSAEALPGVIACGLCEPNAASVL
EADQDWNLHRSMGITLAFFEARESVKNWFDKGLHKQVAETGLREAGGFTIHNLCKVEAKKCVPAVH
RVLDMGEVEAGLIEIDSAVLVADEEQTLADRPTTREKKVGAVGGVVLAGTMNMGGALDQVERGGNI
IIELEEEQYHAVNGKKADGKLTNAVLMVLSLAYLPVLRRQASASALDRGLHDLLIQAPADADNLDHG
REMSIPLPTIHAETAPLYAESEIDRIAWIAPLHLLGTLPAVLLFEPDQYSFISMMIDASLEITETAEVLDDY
HVLERGARPPGFRVILIKGLHIRLLAAIGTIIVVGSAGRAEITAYIQGRAVPIGLAKVTFLPAFAASSCKVV
DIFSTMLRPPERSTVQMLILETVAEL-
LEVTRLQNDKLERLLVLLPGLDAATLLSLLATAEDFADIVTISAAITAILSVAAERVVLRELLLTLAEIAITAL
ALALALLLMLTADRGLTQKERAREGQKKELIQGGAVLQVHAADAMVLAATADKVRLDTDEGTTSAF
ASLNLATVSDGFYVMSSAVLQMSSDLMMIVAVSRVLADKETKNALVVRHPEQVKTQPTYAEGSPG
MVLQTLTGSIPPLAIKPEQVELLWTLVAALWRVKAHIAEAKRPRLERVLVLLLALALIVVLLQSEGGGM
GGG-
MGAAAKILAAFSLLTLKASVGEEVRKVTRRVMDLNSRLGRPEESVNPEFRGEELEQTKFEAANVEVDC
METIRRFVDIPFLVSTRTQHNESEDRGARMVLDVTRVRFIDL-
VTKRSEIILAARAETDGIISREMAKDLQALQPELAALGKIAALEHFIALNDGHISNYVTADIHSVLGMLLL
VLFIQQVRVAKVVWPTRREVTMVFIMAALAVFFLVIGLG-
KLVFMLGGISPLQITTLVLGEGVMAVRQASTFPAES-
VTILQNTYADLICAGRVTKADIERSLHIVVAARVARGTWYVIYHYEITQAQGEAGIIQRSFRQATLSQSL
IDGRIEVKVKVVTSDRYA-
TYEIKGGQHRKGTRFLRVKMGHNIPVNLLARLKMAFGDPNLILVKTFGAVVQKRFAQPLTHGRHGAV
TIGPMEVASPYIQMKK-
RHAPGDSVVGAKPDNIVRSVAVVPELIWARENEDNSAVHEKYVKTTHKVAAKRNRAVDKAQYDKR
NQVHLSFIGLAVKVLPAQAIVHATAGTLLPTESVAVTENLKGVAATIRLDPMEKALIAYIKRMRPQLNL
AGKNDKKIVKCLNNNLDDVEYGKLGRRALKPLTIKSVYDVEHRRATELVKLIPYKDDVEGRKSQFAEKE
-
ALFEPLKAFMMLFGFIARSVYALYLRVVRRTILLVKTKIVVNAKSVLSAIKIIGVSIRLRHKSTNVADTNHYI
KTIEARARMTLAGTPSVKLHLRPGKVGKKAGRIKGFMPYKNYNIFEKLESGRRSKVKDLAKLKKVDKTIL
VDGIRTKQQGGRIPTADLSNAF-
NTRVGVDIVGENEQEQSNPRANAEMGQLFAAIDARVYHHVAALIGHALVAVLALAAILGIGRAHALL
AVKLKINASIEFHSAA-VETAVAAA-HAQPAGEAA-ILDVSEADLGDLHMSEASLDVCRVRRRQLG-
LKKTKKDAYMSLTCFSGKGDKIKIVEASQTQFYEIVATRTAERHGIHAKGTFGKERAMMQITPTKPVKV
WVPIFVSETELLAPIRAFAETNGDGVDVVKLTKDIKVTMFEKAVASLAVAGKSTSIVYTVLEKVTELSKDII
YEQRAKCHYGRGRLRSHVVTHTKQKRHLSMMRRTVVARPRMDAARLTDGEVSVITGATMAFVMSE
QDAV--
GDARMHFHDEPRREAALGIAGQDTFLSTEHPRTLALLFLREFLIHLVDIGTVGAFADAATIYDILRAMEY
KLDRKLSAEIAKVRMCLHDDVANMGVRQEAPRRETAGDPILSAYWINGVTANADIILLVANLECKTAP
ENIDCAVRLFYQRVTLQAKERNGDVSVRLVPAKTQIEAAEGTVIYEIKGNTPGSTQWKLNVDAISRSIV
KSFAVQLFQVDLFLNDTWIATLLRAAQDVVFVDAKEGKEAVDHVVERDNELEMAPQKDIVRRKIRVY
TPAAPSAVVIWDPAIERADKAEAQGTILKIYGPDLQDISCFTDIGSEPKIESLKSWDEKEKYVVHRDDIPP
DI-
TLVGKSSKVNKEFGIPDVAVCAPSKPGVTESIDRGEPKAGSCGEVANLPYPQESLVNRVSSANVQKNI
SMVLGGHPVGEMGISRKVERHLETNMCEIFVDRPDIDVALDFYTLELTPLRVGLDRPNAKVEIAGKESR
KLMAVRDGDAKLQRHPKLSEAGGSGSGAGTKARLPDEALIYDGVLHGDLTFTATDLPEGRV-
VTPVVNTVKAQLKIDKVMIKTFHVVTLSFSSNKGEEHLIKLRLNALKKSTRHYEMMLKIRDTECAQSPH

AIPVGKILKIEALLQTICQAHYTAIEPLLADDLTALGVLGDQGLLHIVPRLMILAVLVVWGLEAAKDVPT
WDLSQRAIITDQTMGLSIRERAWSY-AISEVVGNTFFSRTKDDDTEQRLHARNPI-VLL-YGKAGMV-
ISPSGLRLDSTTETADDHGNADPLILSRERLQDAMEYYVLRILLVFVDEVILGYGSGDDPLKFLVADQA
VTGRDNPGLVCLTRRTFPLDLQRDIVFVMVFADCTMKNLSHKKMHMNLVLIKSKGWIVNVSTKILISD
QAIASYAEDAGAHTQPSESALAVITSRVIAPIVLVISQKPSAPSKTMFLEALNLYDEVTATVLNRPKRYYI
FEGDLISVLNTLDAAVILGMQRPQPDRMRRAVVAAAIRLAIAALASGIVAALEGGAINFSRAVSFVA-
AY----------------DFRVRNHKTSTKRDRPYKAHPKVLHKADTDKGLIAEGVDTIA
>GCA_000172095
AVIKPVIQNEYAPVQLIAQGQVATVSGAGSPSLRAKTVYAKSALSRKTSTKWVAKPIYY-
GVLKMSNIVQLVSISVDSRSVCTVAQLVEQEVVVHGLVALAAVKRKIALEEIDGMTTEDPAKVAQFEE
AVELRRFRDADDRADMLPVMLGLTLKIKFDPHEPVVHDLPIPVIFEVV-
GVMADRGGIEVDECAISQAIGDVDPIEYVSPIDLAAVAGDEMAFIDQEAAVAAARETIGLEELALEASIL
VVGIRPLILPV-FGEAADV-MSFANRSRTLKSIVNLAVAFPELSRARIASAFASEM-
MRRVQAAALAEKRGTAEEIRIVKRMGRSAGLSDDPELVILEDAAEGNSSWAGQIYDDPISGSSESDSA
GNIVY-
ANARQNFARSITYIRKVEISDKANAPLMRGVKHNSKSRSARKLDDAIAVLNFANVPEGIAMAAAAAK
PNATLLHDAMEGEEILMIIVRWKVTWKTAAIIALVLTAGNIVGLIELGGIAAVGKPDGLLNRHHKLNGS
LIEQKPIIEKMTLRLRASLKEKLGKDQVIIFVVKVQGDLVANATVQMG-
GPLGAVVAVQGEVIFVRRHKQTIITDQEATVEVLRGVAHGPADAQALLRIRVTHTLVKISVLLIPD-
PVLVDAVSIREGVGKTLQIIQLDVYLLRDMGTGEETLAESLSRETVRDVLAVDTITGVRLAYGELKERQA
KKLTLKDLRTMM--
LSILAAVTEPRMENLPAGVCRTFITAGTRAAVGMLFLLLDEAGKLYAIDGDLRELQKSVKSLIGLVDAKA
PKVLSAGYATTRLKIRFADCEVLEAEADLYAYKEERRQLLRMVPVYIVLEAKRRQLIVFLLSLKGGHRRA
QAAIGVMLAAALLESQAEEGEVNHATPAILLDDAEIVGTAIMISRQAALQVKDRTKMDVVVVFPEFQ
EKMVVSLRFFCTEVAIFVIALIVTVGEMLSREPEHAQEEGAGFGKRIRGHSMLFALAVCFERSNLGDAL-
ADLFLSMGLAPPRYGVLYTSQPKKKQMPAEPDKDRVDGFELVHAVKEDGASNHELEMPEAMALAVE
ADASINLSAGVLAMNGGMVAVEKVDSKRKMSLIATKWNISKLDLLDVNISGANQVRCLG-
IELADLSASLAKKQVYFLYLPGASNEELEEEKLNGAGASTAEAAADRQGQILVVVPVLVVIQDSNTAGL
IANDGLDEWGTMAYKKHYAFAARMRLHMRTID------
EMEPNKQKSEKIKTVKLEGDTRFRQLATDGVKEQMPVLHALNPDFAVLVPLLVTPAVGSVAADAGVL
GAEVPVDASL-RELQKSQKGSALPISAIRLLEMRVARAMIEEDIMVAGKVAA-
LLKISALETVIKLVLRNDSEATLPWRLSVAERLEADTKRAGGYAARPFKSKPSLCNTQAHAKVADPIQEI
AAGYVAVEEKFVFPVEGIAIQIVLSSPHSRVGAEKRAGEGHLHEMAKELVGKAVMMIKNEPDTNSEGL
MNYITTHQPTDLLFEDCLDVIIDMRRMSVV--
VHLNTV-
HVVVEGVRDVGDYEIMAAKAPSGLIQVERVDDRTGRERTIMWMYGAMEELWLLSVRYADWVTNC
AIGAIILHVYAQQINRAFSLRRLQLDGWPPEASSWWNTWQLWLNKDGFFGYDAAFRA-
KQVLKATLADYEMKDVEGVGRLLSRQEVRAGYRIRQQVEFERLDPRADILLQAKTRLSVIRHALQVVAI
LILREVASEFTLVVAMEDVAMGHVECVLAEAFDFTAKDADIGPAGLRTPDPIFGDGTVLKEAVLGKIDL
SNTVADFRYRMILDGGRQLGVEWLTL-NMLYTRIWVPPAQAALKDLTPIKFFLSHPELDCEW--
SFFSGISKARAQQLMAGALIEEILEAVARFADPEATTHQSNKEKHFKYYGEEVDPVHLAYILDMFALVA
TQVTVSDRLLKDRYVLNMKVFRGFTSLLWMSAQLGEKGPAATVITVGTIAGLGALVLFIILLMRDSVIV
ETLFYRYVY--MQGYSAAKDVADADPEHFLALAAASRDMEWVAIAPAFFYYFKTAT-
FEPFNTAVAREFFGLFEERLRLIEVLCVPPPRVEELAEFVALAGTLATSAKALPGVIAFG-

CEPNAAGVLEAERDWNLHRTMGITLAFFKAQVSVKGWFDKGLHKDVAATGLREAGALTTPFHAQV
DAKRCVPAVHRVVDLGEIEAGLIEIDSAVVVADEEQTLADQPTTRDNSVGAIGGVVLAGTMNMGASL
EQVERGGNIIIELEEQQYHAVNGANDGAKLTNTILMVLSRAYFPVIARTASASALDKGAFDLLIQAPDD
ADNLDHGKVLVIPAPTVLAETAPLYAESDIDKIAWIAPMHLLGTLDAVLLFEPDHYSFISMMIEASLDIT
ETAEVLDDYHVLEKGSRPPAFRKILIKGLHIRLLSAVGTILVVGSAGRTEVTAYIQGRAVPITMQKVTFLP
AFAASSCKAVDIFSTMLRPPERSTIYMLILETVTEL-
LEVTRLENDKLERIAILLPGLDMAAILGLVARAKDFAQIVTVAAAITAILSVAAEAVVLREMLLTKAEIAVT
ALALALALLLMLTADQGLTQKWEAREAQMKELIQGGAVLQVRASDAMVLAATADKVRLSTDEGTTS
AFASLNLATVSDGFYVMSSAVLQLSSDLMMVVEVARVLADKETKNALVVRHPEQVKTQPTYAEGSP
GMVLQTLTDCIPPLAIKPEEVELLWTLVAALWRVKSHLASAMRPRIERVLILILALGLIVVLLQSEGGGM
GGG-
VGSAAKILAGFSILTIKASVEDEVRKVTRRVMDLNSRLGRPEEAVAPEFRGEELEQNKFEAENVEVDCLE
TVRRFVDIPFLVSIRTQHNESEDRGARLVLDVTRVRFIDL-
VTKRSEIILVKRAETDGIIARQMAKDLQALQPEAAALGKIAALDHFISLNDGHISNYVTAEVHSVLGMLL
LVLFIQQVRVAKVVWPTKREVTMVFILAALAVFFIVIGLQINLVFMVGGISPLQITTLVLGEGVMAVRQ
VSTFPAES-
VTILQNTYADLICAGRVTKADIEKSLHIIVGARVARGTWYVIYHYEISQAQGPAGIMQRAFRQATLSQS
LIDGRMEVKIKVVTSDRYA-
THEIKGGQHRKGTRYNRVKVGHNIPVDVLPRLKLAFGDPNLVLVKTFGAVVQKRFAQQLTHMRHGA
VTIGPMEVSSPYIKSKKARHAPGDSVVTIKPENIVRSVAVVPELIWARENEDQSAVHDKYVKTTHKIAA
KRNTAVDKAQYDKRNQVHLSFIGLAVKVLPEQAIVDATAGTLMATETVTVTEGLKGVAATIRLDPME
KALIAYIKRMRPQLNLAGKTDKKIVKCLNNNLDDVEYGKLGRRKIKPITIKSVYDVEHERATELVKLMPY
KDDVEGRKSQFAEK--
ALFEPLQAFMMLYGFIARAVYALYLRVIRRTIVLVKTKVVVNANTVLSAIKIVGVSIRLRHKSTNVAETKH
HIKTIEARARMTLAGSPSVKLHLSEGRVGKKGGRIKGFMPYKNYNIFDKLASGRRAKVKEVAKLKKVDK
SISVDGIRTRQ-
GGRIPKADLSNALANTRVGVDIVGENEQEASNPRANAEMGQLFAGIDARVYSHVGALIGHALTAVLA
SCTILGIGRAHVLLAVALRINAGIEFHSAA-VAKAVAAA-YAQPAGEA-
ILADVSEPNLGALALGDASVDVTRYGDRRLG-
LKKTKKEAYMELTCFSGKGDRVKIVEAMQTQAFEVVATRTAERFGIKAKGTYGKERAMMQIVPTKPVK
VWVPIFVGEDELLAPIRAFAETNGDGVTVVKLSKDIKVTMFEKGVASLAVAGKNTSVVYTVLEKVTELM
KDIIYEQRAKAHYGRGRLRSHVVTHTKSKRHLSLMRRTVVADPSMDAARLTDGEVSVITGATMAFVM
SEQDAV--
GEVDMHFHEEPQREAKLGIAGPETFLATEQPRLLALLFLREYLIHIVDIETQGGFADAATIYDMLRAMA
AKLDRKLSAEIAKTRMCLHDDLAEMGVTQLAARRESAGDPILSAFWINGVTANADIIMLVANLECQV
VPENIDCAVRLFYQRITLQAKERDGDVSVVLVKAQNQIEAAEGTVIYTIKGKTPSTTQAALNVEAISRSI
VKSLAVQLFQVDHFVNDAWIATLLRAAHEVVYVDAHEGREAVDHVVERNTDLEFAPQKDIVARKIRI
YTPAAPSAVVIWDPAIEREEKADAQGTILMIYGPDLQDISCFTDIGAEPKIESLKSWDEKEKYVVHRDDI
PPDITTMVGKSSRVNKEFGIPDVAVCAPAKPGVTESIDRGEPKAGSCGEVANLPYPQESLVNRVSSAN
VQKNISMVLGGHPVGEMGISRGIERHLETNMCEIFVDKDDVDVALDFYRIALTALRVGLDPPNAKVEI
DGKESRKLIAVLDGEADLQRHPKVSEERGALHKAETKARLPDDALIYDGVQHGDLSFTATDLPHGRV-
VTPVNNNVKAQLEIDKVMLKTFHVVTLSFSNNKGDEHLIKLRLNALKKSTRHYEMLLKIRDTECAGSP
HAISVGKILKIDSLLQTICVAHYTEIEPLLADDLTALGVMGDQGLLHVVPRLMILAVLSLLGLEAAFHLPQ
WDLQAEALITDQTMGLAARSRDWSY-CISEVVGNTFFARTKDDDTEQRLHARNPI-VLL-

CGKEGMV-
ISPSGMRLASTAPGEAEHGNADTLILSTERLQDAMGYFVLRILLVFVDMVILAYGSGDEPLKFLVANQA
ITGRANPQLVCLTRRTFPLDLQRDATFVMVFADATMKNLSHKKMHMNLVLIKSKGWVVNVSTKILIS
DQAIASYAEDAGAHTQPSESALAAITSRVMAPIVMVVSQKPTAPSKTLFLEALNLLDEVTATVLNKPKR
YFVFEGDVVAVLGTIDAAVLKGMQRPQPDRMRRAVVAAAIRLAIAG-
ASGVLASLEAGVLNFSRAVDFARGCFAILALTLKPAIN-
PALAFRVRNHKTSTKRDRPYKGHTKFLHKADTEKALVAEGVDTIA
>GCA_000203975
AVIKPVEINKYAPVLMIRQQEVATVSGAGSPSLRAKTVYAKSGLQRKTMTKWVAKPIYF-
GVLKMGNIVQLVSISVDNRAVCTVAQVVEQEVVVHGLVALDAVKRKIALEAMGGMTTEDPAKVAEF
EAELDLKRFRDADARADMLPVMLGLTLEVKFDPHEPVVHDLAVPVIYEVI-
GIMADKGGIEVDDCAISNAVSDVDPIAYVSPIDLEAVAGTEMAFIDQEAAVAAARETIGLEELALEAGIL
VVGIKPLLLPV-FGEEGDV-MSFAARSRTLGSIVALAVAAEELTRA-IDG-IASEM-
MEAAQAAALAEKRGTAEEIRIVKRMQRSSGLADDPELVILEDAAEGNSSWAGQIYDDPVSGSSESDS
AGNIVHAANKRQNFARSITFLRKVEISDKAAAPLMRGVKHNSKSRSARKLDDPIAELNFPNVPPGIAIR
AEAGKATDRLLHDAAEGELLLLIVVRWHVTWKTAVAIALVYAADDLVGLIELGGIAAVGRPDGVLNRH
HKLNGSLIEQRPIIEKLTLRLRASLKEKLGKDQVIIFVVKVQGDLVADATIQMG-
GPVGAVVAIQGELIFVRRHKQTVVTDQEATV-VLKGVAEGPADAQALLRIRVTHTLVGLSVLLIPE-
PVLVESVQIREGVGKSLQIIQLDVYLLRDMGTGEETLAESKSRELIREVLAVDPITGVRLAFGKLKERMGK
KLGLKDLET----
LSILAAVTEPRMENLPEGVCRDFIAAGPAAAVGMVFLLLDDAGKLYQIDGDMRELTKSVKSLIGLVDTK
APKVLSAGYAEERLRIRFADCEVLGTDAELYPYKEERRELLRMVPVYLILEAKRRQLIIFALSLKGGHRRT
MMALGVMLAAAIIESQAEKGEVHHGAPEILLDDAEVVGTSIMLSRQAALDLKQITKVDVIVIFPEFQEK
MVVSLKFFCTEVAIFVIALITTVGEMLSREPEHAQEEGAGFQKRIRGHSTLFALAVLFDRKNCKDA-
LPDLFLALGLAEPRYGVLYTNQPKKKQMPSEPDGDRVDGFELVHAVKEDATS-
QELEMPEAMALAVE-
DGSINLSAGVLAMNGGMVAIEKVDSKRKVALAATKWNISRLDLADVHISGAAPIRSCG-
IEQADLSAALGAKKVWFLFLPGASNAELEEEKLDGAGASTADAAEDKQIAILLVKPVLVVIQDSNSAGL
ISNGGLEEWGTMAYKKHYAFAARMRLHMRTID------
DMAPNKQKSEKVKTVKLEGDTRFRQLATEGVKEQMP-LHALNPDFAV-
LPLLVTPAVGSVAADENVLGAQVPVDASL-
RELQKSQKGSALPITAVRLLGMRAARGMIEEDIMVAGKVAA-
LLKIRALETVIAILLRNDTEATLPWRLSVAEKLEADTKRASGFAARPFKTKPSLCNTQAHSSIADPIQEIAA
GYVAEEEQFVFPVEEVAIKLILSNPHSRVEEKKRGGEGHLHGMAKELLAKAVVMLRNEPGTNTEGLLN
YLTTNQPTDLLFEDCLDVIIDIRRMSVV--
VHLNTV-
HVVVKGVAPVGDYEIMSAKFPSGLVQVEQVDDRTGREKTLMWMYGAMEELWLLSVRWAEWVAN
CAIGAIILHVYAQELNRAFSLKRLQLDGWPKEAARWWNTWQLWLNKDGFFGYEAAFRAVKQVLKAT
LFDFEMSNVEGVGRYQSKQELRVGYRIRQQVEFARLDPRADILLQAQTRLSIIRHALEVVAILILRDVAP
EFTLVVAYQDVARGHVECVLAESFDFTEKNAHIGPVGLRTPAYIFGDGTVLKEAVLGKIDLANTVAEFR
YLVILDGGRALGVEW----NMLYARIWLPPAQAALKNLLPICFFLQHPELDLAWA--
LFAGISKARADKLMAGALVEEILEGVARFADPEATTHQSKKAKHFKYYGEEVDPVHLAYILDLFALVST
QVVVSDRLLKDRYVLNLKVFRGFTALWWLALQLAPKGPSATVITVGTIAGLGALVLFIILL-
RDSVLVETLYYRYVY--MKGYEAAKDVQDADPEHLLQLAAASRDIEWIEIIPAFFYYFDTAT-

FEPFNTAVAKEFFGLFEERLRIIDVLAVPPDRVAALVEFVALAGTLAISAEA-
VGVIACGLCEPNAAEVLEAERDWNLHRSMGITLAFFEARESVKGYFDKGLHKDVAEIGLKEAGAFTTH
FRCDVDAKQVAPAVHRTLDLGEIEAALIEIDSAVVVADEEQTLADKPTTRDKSVGAIGGVVLAGTMN
MGANLDSVERGGNIIIELEEEQYHAVNGKKADGKLTDKVLMVLSVAYFPVIRRFASANALDRGVFGLLI
EAPEDADNLDHGH-
MSIPAPTVLAETTPLYAEADIDKIAWIAPLHLLGTLPAVLLFEPDFQGFISMMIDASLEIMETAEVLDDYH
VLEKGARPPKFRVILIKGLHIRLLAAAGTILVVGSAGRTEITAYIQGQAVPISMQKNTFLPAFSPSSCKAV
DIFSTLLRPPERSSVEMLILETVAEL-
LEVTRGENEKLERLLVLLPGLDQAELLGLLAAAQDFADIVTLGAAITAILSVAAEAVVLREMLLTKAEIAIT
ALALAIALLLMLTGDEGLTQKWQAREAQMKELIQAGAVLQIHAADAMVLASTADKVRLDTDEGTTS
AFASLNLATVSDGFYVLSSAVLQMSSDLMMIVAVARVLADKESKNALVVRHPEQVKTQPTYAEGSPG
MVLQTLTGCIPPLAVKPEEVELLWTLVAALWRVKAHLASAARPHTERVLILLLALGLIVVLLQSEGGGM
GGG-
IGAAAKLLAAFSILTMKASVGDEVRKVTRRVMDLSSRLGRPEEAVNPEFKGEELDQTKYEAENVEVDC
METVRRFVDIPFLVSVRTQHNESEDRGARLVLDVSRVRFIDL-
VTKKSDVILVERVETDGIIAREMAKDMQSLHDEAAALGKVSQLQHFIALGDAHISNYVTAEAHSVLG
MLLLVLFIQQVRVSKVVWPTRREVTMVFIMAALALFFLVIGLGLNIVFMVGGISPLQITTLVLGEGVMA
VRQTSTFPAES-
VTILQNTYADLICAGRVTKADIEKSLHIVVGARVARGTWYVIYHYEITQAQSPAGIMQRAFRQATLSQS
LIDGRMEVKIKVVTSDRYA-
TYEIKGGQHRKGTRYNRVKVGHNIPVDLLARIKMAFGPPNLVLVKTFGAVVQKRFAQPLTHMRHGA
VTIGPMEVSSPYIQSKKARHAPGDSVVDIKPDNIVRSVAVVPELIWARENEDQSAVHEKYVKTTHKIAA
KRNRAVDKAQYDKRNQVHLSFIGLAVKVLPEQAIVHATAGTLLPTESVSVTENLKGVAATIRLDPMEK
ALIAYIKRMRPQLNLQGKTDNKIVKCLNNNLDDVEWGKLGRRKIKPITIKSVYDVEHEQATELVKLMPY
KDDVEGRKSQFAEK--
ALFEPLKAFMMIFGFIARSVYALSLRVVRRTIVLVKTKIVVNAETVLSAIKIIGVSIRLRHKSTNIAETKHHIK
TIEARARMTLAGAPAVKLHLRAGRVGKKGGRIKGYMPYKNYNIFDGLASGRRAKVADLAKLKKVDKTI
AVEGIRTRQ-
GGRLPKADLSNALSNTRVDVEVVGENVQEASNPRANAEMGQIFAAIDARVYSLVAALIGHALEAVLA
LCAIMGIGRAHALLAVKLKMNAGIGMHSAA-VAAAVAAA-
YSQPGGEATAIADVSEGDLGALAISDGSLDVCRLRRRQIG-
LKKTKKEAYMSLTCFSGKGDKIKIIEATQTQAYEIVATRTAERHGIHAKGTYGKERAMMQITPTKPVKV
WVPVFVSDEELLAPVRAFAETNGDGVAVVKLAKDVKITMFEKGVASLAVTGKNTTVVYTVLEKVTELA
KDIIYEQRAKAHYGRGRLRSHVVTHTKQKRHLSMMKRTVVADPSMNAARLSDGEVSVVTGATMAF
VMSEQDAV--
GDVDYHFHEEPRREAQLGIAGPETFLSTEQPRLLAMLFLREYLIHIVDIGTVGNFADAATIYDILRAMQN
KLDRKLSADIAKVRMCLHDDVEDMGVKAEAARRDQAGDAILSAYWINGVTANNDIILLVANLECKTA
PENIDCAVRLFYQRITLQAKERDGDVSVKLVPAQNQIEAAEGTVIYTIKGNNPSTVQMKLNIDAISRSIV
KSLAVQLFQVDVFLNDTWIATLLRAAQDVVFVDAREGKEAVDHVIERDTDLEMAPQKDIVARKIRVY
TPAAPSAVVIWDPAIEREDKADAQGTILMIYGPDLQDISCFTDIGAEPKIESLAAWDEKEKYVVHRDDI
PEDITTLVGKSSKVNKEFGIPDVAVCAPAKPGVTESIDRGEPKAGTCGEVANLPYPQESLVNRVSSANV
QKNISMVLGGHPVGEMGISRGVERHLETNMCEIFVDKDDIDVALDFYKIALTSLRVGLDKPNAKVEID
GKESRKLMAVKDGDASLQRHPKVSEESGTIRKAGEKAKLPDDTLIYDGVQHGDIKFTATDLPEGRV-
VTPVFMSVKAQLEIDKVMLKSFHVVTLSFSNEKAEEHLIKLRLNALKKSTRHYEMLLKIRDTECAQSPHA

IPVGKILKIDALLQTICKAHYTAIEPLLAEDLTALGVMGDQGLLHVVPRLMILAVLHLAPLEASWAIAQW
GLQQEAILTDQTMGLPVRERQWSY-AISEVTGNTFFARTKDDDTVERLHARNPI-VIL-YGKEGMV-
ISPSGLRLASTAPSDKGHGNDEKLILSTERLQDSMYYYVLKVLLVFVDEIILAYGSGEEPLKFLVAHQAVT
GRENPQLVCLTRRTFPLDLQRDATFVLVLADTTMKNLSHKKMHMNLVLVKPKSWVENVSTKILISDQ
AIASYAEDAGAHTQPSESALAVITSRVIAPIVLVVSQKPAAPSKTLFLEALNLYDDITATVQKRPKRYFIFE
GDVVSVLGCMDAAVILGMQRPQPDRLRRAVTARAIRLAIALRGSGLLASLEGGAINFSRAVDFAA-
TYPGLEA--GLAIV-EALDFRVRNHKTSTKRDRPYKGHTKFLHKAEAEKALIAEGVDTIA
>GCA_000220565
AVIKPVTMDKYAPVLMIRQTEVATVSGAGSPSLRAKTVYAKSGLSRKTSTKWVAKPLYY-
GVLKMGKIVQLVSISVDARSVCTVAQVVEQEVVVHGLVALQVVKRKIALEAMEGMTTADPAKVAEFE
DEIELKKWREADQRSDMLPVLLGLTLRIKFDPHEPVVHDLPVPVIFEVIG-
IMAERGGIEVDECAISNAVSDVDPVAYVSPIDLDAVAGDKMAYIDQESAVAAVKEEIALEELALESTVL
VVGIRPLLLPV-FGEDGDNPMSFANRSRELNTIVDLAVTAVAQTRA----GFAAAM-
MGRLRAMALAEKRGTAEEIRIVKRMQQAAGLADDPDLVFLDDAAEGNASWAGQIYDDPISGSSESD
SAGNIVHAANKRQITTRSITFLRKVEISDKAAAPLMRGVKHNSKSRAARKVDDAIAELNFPNVPEGIAIR
AAGGLESHTLLHDAEEGEEILMVIIRIKVTWKTAFILLLLLLAGDIVGLITLGGVAGVGKPDGILNRHHKL
NGSLIEQRPIVEKLTLGLRASLKQKLGAEQIIIFVVKVQGDLVADAKIQM-
GGVLGAVVAIQGDVIFVRRHKQTLITDSESTIEVLRGVATGPADAEALLRIRVTHTVVELSVLLIPE-
PVLVDSVSIREGVGKALQIIQLDVYLLRDMGTGEETLAESASRETIREVLQVDPITGVRLAYGGIRERLGR
KLNLKDLDTL---
LALIRSVTEPRTETLPGGVHRNFVEAGPRAAVGTLFLVLSNAGKIFLIDGDLRELPKSVKSLIGLVDAKAP
KVLSAAYLMGRLRVRFADCHVLISEADLYAYAEPRKELLRMVPVYLINAAKRRQLVILALSLKGGHKRA
HAALGVMPAAALLESQAEQGETNHETPAILLDDAEIVGSSIMVSRQAALELKERTKVDVIVVFPEFQEK
MVVSLKFFCTEVAIFVIALITTVGEMLSREPDTAQEGGAGYSKRIRGHSILFALVATFDRSNLGCNATPT
LFLGLGLADPRFGMFYTNQPKKKQMPAEPDSDKVDGFDLVNAIKEDGAS-HELEMPEAMALAVE-
SAKINLSAGVLAMKGGMAAVERVDSKRKLGLAAEKWNIARIPLLDVHLSGAASFRCCG-
IEQADLSAALGKKEV--
LYLLGASNAELEEEKLDGAGNSTVIVAEERQPQILLVHPVLVVIQDSNAAGLISNGGLDEWGTMAYKK
HYAFAARMRLHMRTIDAIEVGVAMEPNKQKSEKVKTVKLEGDTRFRQLAVEGVKEQMP-
LHALNPDFAV-LPVLVTPAVGRVAADEKVLGARVPCDFSL-
RELQKSQKGAALPISAVRILGMRAARGMIEEDIMVAGKVAA-
LFKIGALETIIRIVLRNDSEATLPWRLSVAERLEADTRRASGYAARPFNSKPSLCDTQAHANVADPIETIA
AGYVAAEERFVFPVEDIAIQIVLSSPHSRV----------------------------------
TSGLLNYLTTNQPTDLLFEDCLDVIIDMRRMSVV---
--VHLNTV-
HVVVAGTSPVGDFQIMTAAADSGLVQVEQVDERTGRERTLMWMYGAMEDLWTMSVTWADWVP
DCAIGA---------
INRAFSLTRLQIEGWPKEAERWWNTWQLWLNKDGFFGYAAAFRALKQVLKQALADYQMSNVEGV
GRYQSRQELRVGYRIRQQVEFERLDPRTDILLQAQTRLSVIRHAMRVTAILVLRDVVGEFSLVVAKEDV
AMGHVECVLADCFSFTEKNAHIGPVGIRTPETILGDGTVFKEATLGKVDFANTIADFRYRMLLDGGRRL
GVHWLTF-
NILYERIWLPPAQAALKDLLPICFFLGHPELDLELDAEIWISISKARADKLMAGALIEGILEGVARFAQPEA
TTHQSKKEKHFKYYGEEVDPVHLAYILELFALVSTQVTISDRLLKDRYVLNLKVFRGLTSLWWLALQLA
DKGPAATIITLGTIAGLGALVLFIILL-RDSVLIETLYHRYVY--

MRGYDAAKDVADADPEHLLPLSAASRDVEWIEIVPAFFYYFDTAT-
FEPFNTAVAREFFGLFEERLRVIEVLAVPPDRVLELVDFVALAGTLAVSAEA-
VGVIAVGLCEPNAAAVLEADQDWNLHRSMGITLAFYKAQESVKGWFDKGLHKGVAAISLEEAGAIST
FNRCEAEAKKCVPAIHRMIDLGEMEAALIEIDSAVVVSEEEQTLADKPTTREKSVGCVGGVVLAGTMN
MGSNLDQVERGNNIIIELEEEQYHAINGKKADGKLTDKVLMVISLSYFPVLARQASANAMDRGVFNLL
IEAPEDADNVDHGGELSIPAATVHWETAPLYAEAEIDRIAWVAPIHMLGTLDAVLLFEPDHYSFISMMI
NASLEITKTAEVL-
DYHVLEKGARPPAFRTILIKGLQVRLLAALGEILVVGSAGREETTAYIQGSAVPITLRKRTFLPAFAASSCK
VVDIFSTMLRPPERSTVQMLILETVAEL-
LEVTRGENDKLERILALLPGLDMAVLLGLLALAADFSDIVTLDAAITAILSVAAEAVVLRELLLTLSEIAISA
LAMGLALLLMLTGDQGLTQKERAREAQLKELIQGGAILQSHARDAMVLAATADKVRLSTDEGTTSAF
ASLTLATVSDGFYVLSSAVLQMSSDLMMVVTVARVLADKEQKNALVVRHPEQVKTQPTYAEGSPG
MVLQTLTGCIPPLAIKPEQVELLWTLVAALWRAKAHLAEAQRPRLERVLVLILALALIVVLLQSEGGG--
MG-MGAAAKVLAAFSLLTVKASVGDEVRKVTRRVMDLNSRLGRPEEAVNPEFRGEELEQTK---
ENVEVDCMESVRRFVDIPFLVSIRTQHNESEDRGARLVLDVTRVRFIDL-
VTKKSEIILVERAETDGIIAREMAKDLQALHADLAALGKVAFLDHFIALSDAHISNYVTAQIHSVLGMLL
MVLFIQQTRIGKVAWPTRREVTMVFVMAAISVFFLVIGL--
KIVFMLGGISPLQITTLVMGEGVMAVRQTSTFPAEA-
VTILQNTYADLICAGRVTKADIEKSLHIVVAARVARGTWYVIYHYEITQAQGEAGILQRAFRQATLSQS
LIDGRMEVKIKVVTSDRYA-
TYEIKGGQHRKGTRFLRVKVGHNIPVEILPRLKLAFGPPNLILVKTFGAVVQKRFAQPLTHIRHGAVTIG
PMEVASPYITQKKARHAPGDSAVDVKPDNIVRSVAVVPELIWARENEDNSAVHDKYVKTTHKIAAKR
NRAVDKAQYDKRNQVHMSFIGLAVKVLPEQAIVGATAGTLLPTEAVAVTEGLKGVAATIRLDPMEKA
LVAYIKRMRPQLNLQGRTDKKIVKCLNNGLDDVEWGKLGRRKIKPITIKSIYDVEPKESTELVKLIPYKDD
VEGRESQFAEKELALFEPLKAFMMMFGFIARAVYALSLRVVRRTIVLVKTKIVVNANT---
AIKIIGVSIRLRHKSTNVADTKHFIKTIEKRARMTLAGSPAVKLNLRAGKVGKKAGRIKGYMPYKNFNIFE
GLASGRRAAVKELAKLKKVDKEITVEGVRTRA-
GGRLPKAAISNAFANTRVGVDIVGENEQEESNPRANAEMGQIFAAIDARVYHLVAAMIGHALEAVLA
VAAILGIGKAHVLLAVKLKVNAGIEFHSAA----------
YAQQGGEAAAIADISEAYLGDLLVTETSLDTCRLRRRGVGELKKTKKEAYLKLTCFAGKGEKVKILAATQ
RQAYEIVATRTAERHGIHAKGTFGKERAMMQITPTKPVKVWVPIFVSEGELLSPVRAFAETNGDGVDV
VKLTTDVKITMFEKAVASLAVAGKSSSVIYTVLEKVTELSKDIIYEQRSKAHYGRGRLRSHVVTHTADKR
HLSMIKRTVVAKPRMDAERLTDGEVSVVTGATMAFVMSEQDAV-
DSDAPMHFHKEPRREAALGIAGPETFLATEQPKVLVLLFLREYLIHIVEIESVNGFADAATIYEILRPLQNK
LDRKLSADIAKVRMVLHDNVANMGVKQEAPRRDQAGDAILSAYWINGVTANVDIILLVANLEIKCVP
ENIHCAIRLFYQRVTLQAKEREGDVSVRLVKAQNQIEAAEGTVIYTIKGQTPGTVQMRLNIEGISRSIVK
SLAVQLYQVDLFTNDTWLGTLLRAAEEVVYVDAKEGKEAVDHVVERDTDLEMAPQKDIVRRKIRIYTP
AAPSAVVIWDPAIERENKAEAQGTILKIYGPDLQDISCFTDIGSEPKIESIKSWDEKEKYVVHRDDIPPDI
-
VMVGKSAKVNKEFGIPDVAVCAPSKPGVTESIDRGEPKAGTCGEVANLPYPQESLVNRVSSANVQKN
ISMVLGGHPVGEMGISRGVERHLETNMCEIFVDKPDVDVAKDFHLLALTPLRVGLDRPNAKIEIEGKES
RKLMAVMDGDAKLQRHPKVSAATG--RKAGEKAKLPDDALIVDGVMHGDISFTATDLPEGRV-
VTSVANNVKAQLQIDKVLLKTFHVVTLSFSNDKGEEHLIKLRLNALKKSTRHYEMLLKIRDTECAQSPH
AIPVGKILKIDALLQTICQAHYTAIEPLLADDLTALGVMGDQGLLHIVPRLMILAVLYLIDLEAAMHVPR

WQLQQRAIVTDQTMGLAARCRAWSF-AISEVVGNTFFASTRDDETEQRLHARNPV-VLL-
YGKMGMT-
ISPSGLRLQSTAPTELNTGNENKLILSKERLQDEMEYYVLRILLVFVDEVILAWGSGEAPLKFLVAEQAIT
GRDNPGLVCLTRRTFPLELQRNATFVLVFEDSTMKNLSHKKMHMNLVLVKSKGWVVNVSTKILISDQ
AIASYAEDAGAHTQPSESALAIITSRVIAPIVLVVSQKPTAPSKTLFLEALNLLDAVTTTILNKPKKYFIFEG
DVVSQLETIDAAVLKGMQRPQPDRMRRAVVAQAIRLAIALRASGLVAELEAGRLNFARAVTFAARLY
APLALLCAPAIPPQALEFRVRNHKVSTKRSRPYKAHTKFLHKSDEEKALIAEGVDTIA
>GCA_000259095
AVIKPVVMKVYAPVLMIRQSQVATVSGAGSPSLRAKTVFRKSGLQRKVSTKWVASPLYF-
GVLAMGKIVQLVSISVDNRAVCTVAQLVEQEVVVRGLVALAGVKRKIALEGIEGMTAEDPAKVVEFED
ELDLKRFRDADDRADLLPVLLGLTLSIRFDPHEPLVHDLPVPVIFEVL-
GIMAERGGIEVDQCAISTAVSDVDPLAYVSPID---
VGGDKMAYIDQAAAVSAAKDEITLEELALESTVLVVKI-PLLIPV-FGDDGGN-
MSFAKRSRELASIVDLAVSALATTRA-ID-GIASAM-
MVRAQAAALAEKRGTAEEIRIVKRMQSAAGLADDPDLVMLEDAAEGNAAWAGQIYDDPISNSSESD
SQGNIVHAANKRQNFARSITFLRKVEISDKAAAPLMRGVKHNSKSRASRKVDDAIAVLNFPNVPEGIA
IRAEAGKSADTLLHDATETEEILLLIVRWDVTWKTAAIVGLLFLAGNAVGMIDLGGIAAVGKPDGLLNR
HHKLNGSLIEQKRIIDKLTLRLRASLKEKLGAESVIILVVKVQGDLVADATVQM-
GGVVGAVVAVQGDVIFVRRHRQTVVTDSESTV-VLRGVAEGPAEAQALLRIRVDHTLVEVSVLLIPE-
PVLVDSVSIREGVGKALQIIQLDVYLLRDMGTGEETLAESVSRELIREVLQIDPITGVSLAYGGFQERASK
ALTLKELETL---
IAILAGVTEPRMENLPDGVRRTFIRAGPRAAVGMLFLLLDDAGRIYDIDGDLRELPKSIKSLIGLVDAKTP
KVLSASYATARLRIRFADVSVLESEANLYAYAEERKELLRMVPVYVINEAKRRQLVIFALSLKGGHRRAR
AAIGVLLAAALLESQAQNGDVHHAAPAILLDNAEVVGTAVMISRQAAVELKHILRIDVIVVFPEFQEK
MVVSLRFFCTEVAIFVIALITTVGEIISREPEHAQESGAGFQKRIRRHSMLFALQCTFDRRNLDDA--
PTLFLALGLAAPRYGVMYINQPKKKQMPSEPDGDRVDGFELVHAVKADAAS-
EELEMPEAMALAVE-
SAKINLTAGVIAMTGGMADIERVDSKRKLALAATKWSIAQLEALDAHFSGAAQVRCVR-
IELATVSAALTSKKV--
LYLLGASNAEFEESKLDGAGNSTIDIAEDRQRLILLVHPVIVVIQDSNAAGLVSNGGINEWGTMAYKK
HYAFAARMRLHMRTIDAIELGQELEPNKQKSEKVKTVKLEGDTRFRQLAVEGVKEQMP-
LHALNPDFAALVPVLVTPAVGQVAADAEVLGADVPVDFSL-
RELQKSQTAAALPISGVRLLAMRAARGMIEEDIMVAGKVAA-
LLKIRAQETVLDIVLRNDSEAWGPWRLSVAERIEADTARASGYAARPFKTKPSLCNTQAHANLAEPIQ
EIAAGYVATESRFVFPVEGIAIQIVLASPHSRVQTAKRAGEGHLHEMAKELVAKAVIMMQNAPATTTE
GLLNYLTTNQPTDLLFPDCLDVIIDLRRMAVV---
IHLTTI-
HVVVPGVRDVAAFQIMTAAAPSGLVQVESVDDRTGDSRTLMWMYGAMEELWLLSVTYADWVKD
CAIGAIVLHVYAQTINRAFSLTRLQLEGWPKEADRWWNTWQLWLNKDGFFGYEAAFRAQAQVLKA
ALYDYDMSDTEGVGRYQSRQQLRVGYRIREQVKFTRLDPRTDILLQAKTRLSVLRHALAVVAILVLREV
VGEFTLVVAMEDVAMGHVECVLADCFSFTEKNAHIGPVGLRTPDPIFGDGTVLRDAVLGKIDLRNTV
REFRYRMIIDGGRELGVQWISL-
NIMYSRIWVPPAQAALKDLLPICFFLSHPSVNLKAWSLLFSGISKARAAKLMAGALVERILDPVAAFAV
PEATTHQSHKAKHFKYYGEEVDPVHLAYILELFALVATQVTVSDRLLKDRYVLNLKVFRGFTSLLWMS

AQLGDKGPSATIITVGTIAGLGALVLFIILL-RDSVLLETLFYRYVYV-
MRGYQAAKDVADADPEHLGPLAAMTRDVEWIEVVPAFFYYFDTAT-
FEPFNTAVAKEFFGLFEERLRVIDVLAVPPDRVDALSEFVALAGTLPTGARALAGIIATGLCEPNAAAVL
EADQDWNLHRSMGITLAFYKAQKSVKGWFDKGLNKAVAATQLTEAGALTSTFAASATARQCCPAV
HRVIDLGEIEAALIEIDSRVVVADEEQTLADKPTTREKSVGCVGGVVLAGTMNMGANLDQVERGNNII
IELEEEHYSAVNG-KADGKLTDKVLMV-----
FPVLARWASANALPRGVLGLLIQAPNDADNLDHGGQMVIPAATIHAETAPLYADAEIDKIAWIAPIHL
LGTLDAVL-FEPDQYSFISMMIDASLEIMDTAEV--
DYHVLEKGARPPSFRTILIRGLHIRLLAAVGTILVVGSAGRDEITAYIQTRAVPITLNRHTFLPAFSASSCL
VVDIFSTMLRPPERSAVMTLILETVAEL-
LDVTRGENDKLERILVLLPGLDSAALLELVAAGDDFSNIVTIGAAITAILSVAAERVVLRELLLTKAEIAVT
ALALALALLLMLTGDQGLVQKELAREQQMKELIQGGVVLQIHAADAMVLASTADKVRLGTDEGTTS
AFASLNLATVSDGFYVLSSAVLQMSSDLMMIVGVARVLADKETKNALVVRHPEQVKTQPTYAEGSP
GMVLQTLTGMIPPLAIKPEQVELLWTLVAALWRVKAHLAEAARPHHERILVLILALSLIVVLLQSEGGG
MGGG--
SAAAKILAAFSLLTLKASVGDEVRKVTRRVMDLNSRLGRPEEAVNPEFRGEELEQSKYEAENVEVDCM
ETVRRFVDIPFLVSTRTQHNESEDRGARLVLDVTRVRFIDL-
VTKKSEIILVERAETDGVIARGLAKDLQSLQAEIAALGKIAALQHFISLNDGHISNYVTAEIHSVLGMLLL
VLFIQQTRIGKIVWPSRREVTTVFIMAALATFFLVIGL--
NLVFMVGGISPLQMTTLVLGEGAMVVRQT-TFPAES-
VTVLQNTYADLICAGRVTKADIESSLHIVVGARVARGTWYVIYGYEITQAQGEAGIMQRAFRQATLSK
SLIDGRMDVKIKVVTSDRYA-
TYEIKGGQHRKGTRFLRVKVGHNIPVEVIPRLKMAFGPANLILVKTFGAVVQKRFAQPLTHIRHGAVTI
GPMEVASPYITNKKARHAPGDSVVDIKPENIVRSVAVVIELIWARENEDQSAVHDKYVKTTHKIQAKR
NAAVDKAQYDKRNQVHLSFIGLAVKVLPEQA---
-
RMRPQLNLAGKTDKKIVKCLNNGLDDVEYGKLGRRRIKPITIKSVYDVEHREATELVKLLPYKDDVESR
KSQFAEK--
AIFEPLRAFMMLFNYIARAVYALSLRVVRRTIVLVKTKVVVNANTVLSAITIIGVSIRLRHKTTNVADTKH
HIKTIEARARMTLAGTPSVKLNLRPGKIGKKGGRIKGYMPYKNYNIFDGLSSGRRAATKELAKLKKVDKT
IEVGGIRTRQQGGRLPVADLSNALANTRVGVDIVGENEQEESNPRANAEMGQVFAAIDARVYHLVA
ALIGHALEAVLALCDIWGIGKAHALLAVTR-VNAMIELHSAA-VDTAVAAA-
FHEPAGEATTLADVSESYLGALLLTVLSLDVLRLRRRQLG------------------------------------
----
RFGIKAKGHFGKERAMMQITPTKPVKVWVPVFVDEEELLAPIRAFAETNGDGITVVKLTTDIKITMFEKG
VASLAIAGKSTTIVYTVLEKVTELSKDIIYEQRAKAHYGRGRLRSHVVTHTKQKRHLSMIKRTVVAQPR
MEAARLTDGEVSVVTGATMAFVMSEQDAV--
GDVDMHYQEEPRHEAELGIAGPETFLSTEQPRVLALLFLREYLIHIVEIESVGGFADAATIYDILRAMHN
KLDRKLSAEIAKVRMCLHDDVADMGVRQEAPRREAAGDPILSAYWINGVTANADIIMLCANLECATV
PENIDCAIRLFYQRVTLQAKERDGDVSVALIPAKNQIEAAEGTVIYTIKGVTPGTIQMKLNVEAISKSIVK
SLAVQLYQVDLFINDTWIATLLRAADDVIYVETQEGREAVDHVIERDQELEFAPQKDIVRRKIRIYTPAA
PSAVVIWDPAIDREDRADAQGTILKIYGPNLQDISCFTDIGSEPKIESLKAWDEKEKYVVHRDDIPPDI-
TMVGKSAKVNKEFGIPDVEVCAPSKPGVTESIDRGEPKAGSCGEVANLPYPQESLVNRVSSANVQKNI
SMVLGGHPVGEMGISREVERHLETNMCEIFVDKDDIDVALDFYELDLT-

LRVGLDRPNAKVEIDGKESRKLMAVLDGDANLQRHPKVSEAAGTGVGAGRKANLPDDALIFDGVHH
GDLSFTVTDLPEGRV-
VTPVTIAVKAQLKIDKVLLKTFHVVTLSFSGNKGEEHLIKLRLNALKKSTRHYEMLLKIRDTECAQSPNAI
PVGKILKIDKLLQTICTAHYTAIEPLLADDLTALGVKGDQGLLHIVPRLMIMAVMTLWGLEVDLTHPSW
GLMARLNLTDVTMGLAMRERHWSY-CIAEVVGNTFFAMTRDDDTTERLHARNPI-VLL-
YGKFGMT-
ISPSGLKMASTAPTSAEHGNDILLILSAERLQDSMSYFVLHILLVFVDEIILAYGSGDRPMKFLVAHQAV
TGRANPQLVCLTRRTFPVELQRDATFVLVFEDSTMKNLSHKKMHMNLVVIKSKGWIVNVSTKILISDQ
AIASYAEDAGAHTQPSESALAAITSRVIAPIVLVVSQKPTAPSKTMFLEALNLYDQVTATVQNRPKRYYI
FEGDLISVIGHLDAAVLKGMQRPQPDRLRRAVVAAAIRLAIAALGSGVLARLQQGLINFARSITFAR--
-----------------GFRVRNHKVSTKRSRPYKAHPKVLMKADTEKKLIAEGVDTLA
>GCA_000299875
AVIKPVTQNVYAPVLMIRQTEVATVSGAGSPSLRAKTVFAKSGLQRKVSTKWVLKPLYY-
GVLKLGKIVQLVSISVDNRAVCTV--
MVEQDVVVHGLVALEVVKKKIALEALDGMTTEDPAKVADYEEELELKRFRDADDRADMLPIMLGLTL
KIAFDPHEPVVHDLPIPVIFEVVSTIMAERGGIEVDECAISTAVSDVDPIEYVSPIDLAAVAGDKMAYIDQ
EAAVSAAKDDIAL--LALESSVLVVGI-PLLLPI-FGAEGGT-MSFANKSKELGTIVDLAVAAVAELRAR-
-QGIASSM-
MVRAQASALAEKRGTAEQIRIVKRMQKSSGLADDPELMVMEDAAEGNSSWAGQIYDDPISGSSESD
SAGNIVFAANKRQATDRSITFIRKVEITDQAAAPMMRGVKHNAKSRAARKVDDAIAELNFPNVPEGI
AIAAAGGTPNDTLLHDAAEGEEILMVVVRWKVTWKTAIAVVIVLTAGDVFGMIEIGGFAAIGKPDGVL
NRHHKMNGSLIEQKRIMDKLTLRLRAALKQKLGAENVIILVVKVQGDLVAAAKIQM---
SIGAVVAIQGELIYVRRHRQTLITDQEATVEILKGVAEGPADAEALLRIRVTHTIVELSVLLIPE-
PVLVDAVSIREGVGKALQIIQLDVYLLRDMGTGSETLAESMSREAIREMLAIDPITGVRLAYGKLKERQG
KALGIKEMDTIF--LRIL-
GLTEPRMADMPQGVCRKFVVAGPKAAVGTVFLLLTDAGKLYTIDGDMRELSKSIKAMVGLTDAKAPK
VMSAQYATARLKIRYADCTVMTTEADLYAYAEERKELLRMVPVYLINAAERRQLIIFALSLKGGHRRAM
ASLGVLIAAAILESQAERGPVHHKEPAITLQDAEIVGSEMMISRQAALALKMVFRIDVIVIFPEFQEKMV
VSLKFFCTEVAIFVIALICTVGQMLSREPNAAQEGGAGFTKRIRKHSVLFALDVTFDRMNCKVDATPDL
FLALGMAPPRYGIIYTNQPKKKQMPTEADGDKVDGFELVNAIKEDGASNHVLDFPEAMALAVEADA
AINLTAGVLGLNGAMAAIEKVDSNRKIALAVEKWNIAKIEALDVSISGAAPFRALS-
IELADISAALNSKDIWFLFLPEASNKELEAKLDGQAGSSTADAAADRQKAILLVLPVLVVIQDSNAAGLI
ANGGLEEWGTMAYKKHYAFAARMRLHMRTID-------
MIPNKQKSEKIKTVKLEGDTRFRQLATAGIKEQMP-
LHALNPDFAVLLPVLVTPAVGEVAADKECLGAGVPVPFSL-
RELQKSQSGAALPITAMVILDIKQARAMIEEDIMVAGKVAA-
LFKIRATTTIFEIVLRNDSEATIPWRLSVADRLEADMARAGGYAARPFSTKPSLCDVQAHAKVAEPIETIA
AGYVATEERFVFPVEDIAIKLILASPHSRVGAAKRAGEGHLHEMAKELLAKAVVMVKNEPNTNTEGTL
RYITTGQPTDLLFEECLDIVIDMRRMSVV---
LHLNTI-
HVVVPGARDVAAFEIMSAAFDSGLIQVERVDDRTGRERTSMWMYGAMEDLWTLSITYADWVKNCA
IGAIILHVYAQTLNRAFSLTRLQLSGWPQDAARWWNTWQLWLNKDGFFGYEAAFKAVKQVLKADL
AEYDMKDVEGVGRMLSRQEVRAGYRIRENVDFNRLAPRTDILLQSRTRLSVLRHALQVVAILVIRDVV
GEFSVVVAQEDVAMGHVECVLADSFDFTAKNADIGPVGLRTPDTIFGDGTVLQEAVLGKIDLSNTVA

DFRYLFIIDGGRELGVWWITV-NMIYARIWVPPAQADLKDLQPICFFLSHPQLDLLLED-
LFAGISKARADKLMAGALVEEILADIARFADPEATTHQSHKEPHFKYYGEEVDPVYLAYILTLFGLVATQ
VTMSDRLLKDRYVLNLKIFRTFTSLLWYGLALAEKSPAATAITLGTVAGLGALVLFIILL-
RDAVILETLYYRYVY--MLGYKAAKDVADAPPEHFGPLAAASRDMEWIEIVPAFFYYFRNAK-
FEPFNTAIAKEFFGLFKERI---EVLAVPPDRVEALVEFVALAGTLATSAEAAPGIIAAG-
CEPNAAEVLEADQDWNLHRSMGITMAFFKAQESMNGWFDKGLHKGVAAISLEEAGAMTSTNDCA
VREHECCPAVHRVLDLGEIEEALIEIDSAVVVSAEEQTLADKPTTRNKSVGAVGGVVLAGTMNMGAN
LDSVERGNNIIIELEEEGYHAVNGRKGDGKLTNKILMVLSEAYFPVLRRLASANSLDRGVFGLLIQAPSD
ADNLDHSKNMTIPLPSIHAETHALFAEDDIEKIAWVAPLHLLGTIPAVLEFEPDYYGFISMMIEASLDIM
KTAEVLDDYHVLEKGARPPSFRVVLIRGLHIRRMAAIGTIIVVGSAGRDEVTAYIEGRAVPISLKKHSFLP
AFAASSCEVVDIFSTILRPPERSSIVTMILETVAEL-
LEVTRIENDKLERLMLLLPGLDASEILGLVAAAKDFAQIVTLSQAIVAILSVAADAVILRELLLTLAEIAISSL
ALALALLLMLTPDEGLTQKEAAREEMDKELIQAGVVLHSHAEDQMVLAATADKVRLDTDEGTTSAFA
SLNLATVSDGFYVLSSAVLQLSSDLMMIIAVARVLADKETKNALVVRHPEQVKTQATYAEGSPGMVL
QTLTKCVPPLAVKAEEIELLWTLVKALWNVKAHFAEAARPRNERILILILALCLIIVLLQSEGGG--MG-
VTAAAKIFAGFSLLTLKAAVGDEVRKVVRRVMDLNSRLGKPEEAVGAEFRGAELEQNKYEAANVEVD
CMETVRRFVDIPFLVSIRTQHNESEDRGARLVLDVTRVRFIDL-
VTKRSDVILVEKTETDGVIAREMAKDLQALHPEVAALGKVSALEHFISLQDGHISNYVTQAIHSVLGML
LLVLFIQQVRVTKIVWPTRREVTMVFVMAALGTFFLVIGL--
QLVFMLGGISPLAITTLVLGEGVMGVRQTSTFPEDS-
VTILQNTYADLVCAGRVTKADIEASLHIVVAARVAKGTWYVIYHYEISQAQQEEGIFQRAFRQATLSQ
SLIDGRMEVKIKVVSSDRYA-
TYEIKGGQHRKGTRFNSVKVGHNIPVDVLARIKMAFGPANLILVKTFGAVVQKRFAQPLTHTRHGAVT
IGPMEVASPYIKSKKARHAPGDSAVSLKPDNVVRSVAVVVELIWARENEDNGAVHDKYTKTTHKIAA
KRRTAVDKAQYDKRNQVHLSFIGLAVKVLPDQA--

KMRPQLNLQGKTDNKIVKCLNNNLDDVEWGKLGRRKIKPITIRSVYDVEHKEATELVKLMPYKDDVES
RQSQFAEK--
AIFEPLKAFMMLFGFIARAIYALSLRVVRRTILLAKTKIVVNAATVLSAIKIIGVSIRLRHKTTNIADTKHHIK
TIEKRARMTLAGAPNVKLHLRAGRVGTKGGRIKGYMPYKNFNIFEGLSSGRRAKVKELAKLKKVDKEIT
VEGIRTRAQGGRKPIADLSNALANTRVGVDIVGENEQEESNPRANAEMGQIFAAIDARVYHLVGALIG
HALEAVLALCSIWGIGRAHALLAIW-KMNANIELHSAA-VPVAVAAA-
YAQSGGEAAAMLDVSEADLGDISLSEQTVDVSRARRRQLSE---------------------------------
----
RHGIHAKGTFGKERAMMQILPTKPVKVWVPVFVTEEELLAPIRAFAETNGDGVTVVKLTKDIKITMFEK
AVASLAVTGRSSSIVYTVLEKVTELMKDIIYEQRAKAHYGRGRLRSHVVTHTKSKRHLSMIRRTVVASPS
VDAARLTDGEVSVITGATMAFVMSEQDAV--
GDVDMHFHEEPRREAELGISGPERFLTTEQPRVLALLFLKAYLIHIVEIETVGGINNQATIYDILRAMQN
KLDRKLSAEIAKTRMCLHDNLAHMGVTAESPRRDTAGDHILSSYWINGVTAKADIILLVANLETRCVPE
NIQCAIRLFYQRITLQAKERDGDISVRLVPAKNQIEAAEGTVIYTIKGKTPGTLQMKLNVEAISRSIVKSL
AVQLFQVDLFINDTWIATLIRAASLVVFVDAKEGREAVDHVVERDTELEMAPQKDIVRRKIRIFTPSAPS
AVVIWDPAIDRAEKADAQGTIIKIYGPNLQDISCFTDIGAEPKIESLKAWDEKEKYVVHRDDIPEDI-
TLVGKSSKVNNEFGIPDVEVCAPAKPGVTESIDRGEPKAGSCGEVANLPYPQESLVNRVSSANVQKNI
SMVLGGHPVGEMGISRGIERHLETNMCEIFVDKPDVDVALDFYTLSLTVLRVGLDRPNAKVEIDGKES

RKLMAVRDGAATLQRHPKVSEGSGTR-KAGTKAKLPDDALILDGVLHGDVKFTVTDLPEGRV-
VTPVVLSVKAQLSIGQVILKTFHVVTLSFSSGKGEDHLIKLRLNALKKSTRHYEMLLKIRETECAESPHAID
VGKILKIDSLLQTICQAHYTAIEPLLADELTALGVMGDQGLLHIVPRLMILAVLHLWKLEARFVVKTWGL
QARAIITDQTMGLQQRERQWSF-AISEVVGNTFFARTKEDDTEQRLRARNPI-VLL-YSKEGMV-
ISPSGLRLESTAPDAMEHGNAEDLILSRERLQDAMGYYVLRILLVFVDEIILAYGSGKAPLKFLVADQAV
TGKENPGLVCLTRRTFPLELQRDATFVLVFEDSTMKNLSHKKMHMNLVLIKSKGWVVNVSTKILISDQ
AIASYAEDAAAHTQPSESALAVITSRVIAPIVMVVSQKPSAPSKTMFLEALNLYEAVTSTVLNKPKKYYIF
EGDVVAKMSTLTGRLLVGILLVMPRVFRIACVAAALRMAIAW------------------------------
-------------AFRVRNHKVSTKRSRPYKGHTKFLHKSELEKALIAAGVDTLS
>GCA_000376545
AVIKPVEINKYAPVLMIRQTQVATVSGAGSPSLRAKTVFRKSGLQRKTMTKWVQSPLYY-
GVLKMGNIVQLVSISVDNRAVCTVAQVVEQEVVVHGLVALDAVKRKIALEEMDGMTTEDPAKVAEF
EEELDLKRFRDADARADMLPVMLGLTLQVRFDPHEPVVHDLPIPVIYEVI-
GIMADRGGIEVDDCAISNAVSDVDPIAYVSPIDLAAVAGEKMAYIDQEAAVGAAKEEIALEELALESVV
LVVGI-PLLLPV-FGAEGAV-MSFAARSRTLESIVGLAVAAAELTRA-ID-GIASTM-
MTAAQAEALAEKRGTAEEIRIVKRMQRSSGLADDPDLVILEDTAEGKASWAGQIYDDPISGSSESDSL
GNIVHAANKRQNFARSITYLRKVEISDKAAAPLMRGVKHNSKSRSARKVDDAIADLNFPNVPAGIAIK
AIAQKPEDRLLHDATETEGILLIVVRWHVTWKTAIAIGLTFLAGDIVGLIALGAIAGIGKPDGLLNRHHKL
NGSLIEQRPIIEKMTLRLRASLKEKLGKDQIIIFVVKVQGDLVADATVQMG-
GPVGAVVAVQGDVIFVRRHKQTVITDSESTV-ILRGVAEGPADAEALLRIRVEHTLVELSVLLIPE-
PVLVDNVSIREGVGKSLQIIQLDVYLLRDMGTGEETLAESKSREVIREVLAIDPITGVRLAYGKLKERAGK
KTGLKDLETLF--RSILP-
VTEPRMENLPEGVCRTFITAGPRAAVGMLFLLLDEPGKLYPIDGELRELTKSVKSLISLVDAKVPKVLSAG
YAEERLRIRFADCEVLASEAELYAYKDERREMLRMVPVYIINEAKRRQLILFALSLKGGHKRAMMALGV
MLAAAIIESQAEKGAVHHDEPAILLEDAEVTGTSIMVSRQASLELKHVSKVDVIVIFPEFQEKMVVSLKF
FCTEVAIFVIALIVTVGDMLSREPEHAQEDGAGYAKRIRGHSVVFALEVTFDRRNCRSA-
LADLFLSLGLLPIRYGVLYTNQPKKKQMPSEPDGDRVDGFELVHAVKEDGSSNHELEMPEAMALAVE
ATETINLSAGVLAMTGGMVAIERVDSKRKVALAATKWNISRLELLDVTISGAAPVRCCG-
IEEAGMSAALGKKKV-
FLYLPGASNAELEEEKLNGAGNSTADVAEDRQREILLVHPVLVVIQDSNSAGLISNGGLDEWGTMAY
KKHYAFAARMRLHMRTIE-------MEPNKQKSEKVKTVKLEGDTRFRQLAIEGVKEQMP-
LHALNPDFAV-LPLLVTPAVGSVAADAQVLGATVPVDASL-
RELQKSQKGSALPISGIRLLDMRAARGMIEADIMVAGKIAA-
LFKIRALETIIGIILRNDSEATLPWRLSVAERLEADTKRASGFAARPFKTKPSLCTTQAHARIADPIQEIAA
GYVASDERFVFPVEDIAIRLILYNPHSRVGEKKRGGEGHLHAMAKELLGKAVVMLRNEPGTNTEGLLN
YLTTNQPTDLLFEDCLDVIIDIRRMSVV---
VHLNTV-
HVVVKGVADVGDYEIMNAAFPSALIQVEQVDDRTGEEKTLMWMYGAMEELWLMSVRWADWVA
NCAIGAIILHVYAQTLNRAFSLKRLQLDGWAKEAARWWNTWQLWLNKDGFFGYEAAFRA-
KQILKAALADFEMKDVEGVGRYLSKQEIRAGYRIREQVAFARLDPRTDILLQAKTRLSIIRHALQVVAILI
LRDIAPEFT-
VVAYQDVARGHVECVLAESFDFTEKDAHIGPVGLRTPDPIFGDGTVFKEAVLGKIDLANTVAEFRYLVL
LDGGRELGVEWVTLQNMLYDRIWLPPAQAALKNLQPICFFLQHPELDM---------
ISKARAEQLMAGALIEEIIEAVARFADPEATTHQSKKAKHFKYYGEEVDPVHLAYIFTATALVATQVVLS

DRLLKDRYVLNLKV-
RALTSLWWLSLQLAEKGPCATIISVGTISGLGALVLFIILLMRDSVLVETLYYRYVY-
VMRGYSAAKDVQDADPEHLLELAAASRDIEWIEIIPAFFYYFDTAT-
FEPFNTAVAREFFGLFKERLRVIEVLAVPPDRVEALVEFVALAGTLAISAEALPGVIACGLCEPNAAGVLE
AERDWNLHRSMGITLAFFEARDSVKGYFDKGLHKAVAEFSLKEAGAMTTHFRCDVEAKQVVPAVHR
TLDLGEIEAALIEIDSAVVVSEEEQTLADKPTTREKSVGAVGGVVLAGTMNMGANLDSVERGGNIIIELE
EEQYHAVNGKKSEGKLTDKVLMVLSVAYFPVIARFASANALDRGVFGLLIDAPTDADNQDHGREMSI
PAPTVHAETAPLYAESDIDRIAWIAPLHLLGTLDAVLLFEPDFQGFISMMIEASLEIMETAEVMEDYHVL
EKGARPPGFRVVLIKGLHIRLLAAVGTLLVVGSAGRTEVTAYIQGRAVPISLRKQTFLPAFAPSSCNVVDI
FSTLLRPPERSAVEMLILETVAEL-
LEVTRGSNDKLERMQVLLPGLDMAELLELLAAADDFADIVTIGAAITAILSVAAEAVVLREMLLTKAEIAI
TALALAMALLLMLTGDTGLTQKEAAREAQMKDLIQAGAVLQLHAADAMVLAATADKVRLSTDEGTT
SAFASLNLATVSDGFYVLSSAVLQMSSDLMMIVSVARVLADKESKNALVVRHPEQVKTQPTYAEGSP
GMVLQTLTGCIPPLAIKPEEVELLWTLVAALWKAKAHLASATRPHNERVLILLLALGLIVVLLQSEGGG
MGGG-
ATAAAKVLAAFSILTLKAAVGDEVRKVTRRVMDLSSRLGRPEEAVNPEFKGEELDQTKYEAENVEVDC
METVRRFVDIPFLVSIRTQHNESEDRGARLVLDVTRVRFIDL-
VTKKSDVILVERVETDGIIARQMAKDMQSLHPEAAALGKVSALQHFIALGDAHISNYVTAATHSVLG
MLLLVLFIQQVRVSKVVWPTRREVTMVFIMAALAAFFVVIGLGINIVFMVGGISPLQITTLVLGEGVMA
VRQTSTFPAES-
VTILQNTYADLICAGRVTKADIESSLHIVVGARVARGTWYVIYHYEITQAQGPAGIMQRAFRQATLSQ
SLIDGRMDVKIKVVTSDRYA-
TYEIKGGQHRKGTRYNRVKVGHNIPVEVLARIKLAFGPPNLVLVKTFGAVVQKRFAQPLTHMRHGAV
TIGPMEVSSPYIKSKKARHAPGDSVVDIKPDNVVRSVAVVPELIWARENEDQSAVHEKYVKTTHKIAA
KRSRAVDKAQYDKRNQVHLSFIGLAVKVLPEQAIVEASAGTLLPTESEAVTENLKGVAATIRLDPMEKA
LIAYIKRMRPQLNLQGKTDNKIVKCLNNNLDDVEWGKLGRRKIKPITIKSVYDVEHTRATELVKLMPYK
DDVEGRKSQFAEK--
ALFEPLKAFMMMFGFIARAVYALSLRVVRRTILLVKTKIVVNANTVLSAIKIIGVSIRLRHKSTNVAETKH
HIKTIEARARMTLAGAPSVKLNLRPGRVGKKGGRIKGYMPYKNYNIFDGLASGRRAKVKELAKLKKVD
KTIAVDGIRTRQQGGRLPKADLSNALSNTRVDVEVVGENEQEESNPRANAEMGQIFAAIDARVYSLV
AALIGHALLAVLAICSILGIGKAHVMLAVKLKINAGIEMHSAA-VAAAVAAA-
YSQPAGEATAIADVSEAYLGALAITESSLDVCRLRRRQLG-
LKKTKKEAYMSLTCFAGKGDKVKIIEATQTQAVEIVATRTAERHGIKAKGTYGKERAMMQITPTKPVKV
WVPVFVNEDELLAPVRAFAETNGDGVTVVKLTTDVKITMFEKGVASLAVTGKVTTLVYTVLEKVTELSK
DIIYEQRAKAHYGRGRLRSHVVTHTKQKRHLSMMKRTVVADPSMDAARLTDGEVSVVTGATMAFV
MSEQDAV--
GDVDMHFHEEPRREAALGIAGPETFLATDQPRLLALLFLREYLIHIVDIDTVGNFSNAATIYDILRAMQ
NKLDRKLSADIAKVRMCLSDDVADMGVTDAAARRDTAGDSILSAYWINGVTATSDIILLVANLECKT
APENIDCAVRLFYQRITLQAKERDGDVSVRLVPAQNQIEAAEGTVIYTIKGNNPSTAQMKLNIEAISRSI
VKSLAVQLFQVDLFLNDTWIATLLRAAEDVVYVDAREGREAVDHVVERDTELELAPQKDIISRKIRIFTP
AAPSAVVIWDPAIDREDKADAQGTILMIYGPDLQDISCFTDIGSEPKIESLAAWDEKEKYVVHRDDIPE
DI-
TLVGKSSKVNKEFGIPDVAVCAPAKPGVTESIDRQEPKAGTCGEVANLPYPQDSLVNRVSSANVQKNI
SMVLGGHPVGEMGISRDVERHLETNMCEIFVDKDDVDVALDFFKLSLTPLRVGLDKPNAKVQIDGKE

SRKLMAVKDGEAKLQRHPKVSEESGTR-KADDKAKLPDDALIFDGVQYGDLSFTATDLPEGRV-
VTPVFNNVKAQLAIDKVMLKTFHVVTLSFSNDKGEEHLIKLRLNALKKSTRHYEMLLKIRDTECAQSPH
AIPVGKILKIDSLLQTICKAHYTAIEPLLAEDLTALGVMGDQGLLHVVPRLMILAVLHLLGLDARGQLST
WSLQEAAVVTDQTMGLAIRDRSWSF-AISEVVGNTFFARSKDDDTEERLHARNPI-MIL-YGKEGML-
ISPSGLKLASTQPSDDEHGNKNKLILSTERLQDQMSYHILAILLVFVDEMILAYGSGEEPLKFLVAEQAV
TGRANPQLVCLTRRTFPLDLQRDAVFVLVLEDTTMKNLSHKKMHMNLVLVKPKSWVENVSTKILISD
QAIASYAEDAGAHTQPSESALAIITNRVIAPIVLVLSQKPAAPSKTMFLEALNLYDEITATVAKRPKRYFIF
EGDVISVLNTLDAAVILGMQRPQPDRLRRAITARAIRLAIALRGSGVLAQLEGGVINFSRAVSLAAKVY
DALNLLARAAISLPALDFRVRNHKTSTKRDRPYKGHTKFLHKAEEEKALVAEGVDTLS
>GCA_000382265
AVIRPVTMKTYAPVLMIAQSQVATVSGAGSPSLRAKTVYAKSGLQRKSETKWVVKDFYY-
GVVKLGNLVQLVSISVDNRAVCTVAQLVEQEVVVHGMVALDAVKKKIALEEITGMTAEDPAKVVDY
AGELDLRKFRDADDRADMLPILLGLTLKIRFDPHQPLVHDLPVPVVFEVV-
GIMVQRGTIEVDECAISTAISDVDPIVYVSPIDLAAVSGDKMAYIDQESAVSAAKADIAL--
LALESAVLVVGI-PLLIPV-FAADADV-MSFANRSRTLKTIVNLAVSYPEIKRA-ID-GIAASL-
MEASQAAALAEKRGTAEQIRIVKRMQRSAGLADDPELVLLEDAAEGNSNWAGQIYDDPLSGSSESD
SAGNIVFAANKRQNFQRSITFLRKVEISDQAAAPLMRGVKHNAKSRSARKVDDTIAALNFPNVPEGIA
IRAVAGKPVDLLLHDATEGEEILMIIVRWHVTWKTAIIIALFPRAGDIVGLIELGGIGAVGRKEGLLNRHH
KMNGALIEQRRIVEKLTLRLRAALKQKIGAEQIIIFVVKVAGDLVAKAKIQM---
SVGAVVAIQGETIYVRRHRQTVIADEDATVEVLRGVAEGPADAQALLRIRVDHVLVKLSVLLIPE-
PVLVESVSIREGVGKALQIIQLDVYLLRDMGTGEETLAKSRSREMIREILQVDPITGVRLAFGILRERLVKG
GTMKELGTL---
LAILDQITEPRMANLPDGVCRTFINAGPRAAVGMIFLLLDDAGKIYTIDGDLRELTKSIKSLIGLVDAAKP
KIMSAAYAAARLKVRYADVTVLVSDANLYAYAEERRELLRMVPVYITNAAARRQLIIFALSLRGGHRRA
RAAIGVLIAAAILESQAERGAVNHAEPAVLLDDGEIVGSEMMISRQAALDLKQIIKTDVIVIFPEFQEKM
VVSLKFFCTDVAIFVIALICTVGAMLSREPSSAQEDGAGFQKRIRAHSVLFALVATFDRRNLRSA---
NLFLALGLAPPRYGVLYTEQPKRKQMPSEPDKEEVDGFDLVHAIKEDTASNHELDMPEAMALAVEAD
ATINVTAGVLAMTGDMAAMEKVDSKRKMALAVEKWTISAESIVDIIISGAHPFRALN-
IETADVSAGLPRKSIWFLYLPGANNAEMEEEKLDGAGASTAEAAMNKQTGILLVNPVLVVIQDSNTA
ALIANGGLEEWGTMAYKKHYALAARMRLHMRTID------
QMEPNKQKAEKIKTVKLEGDTRFRQLATEGVKEQMP-LHALNPDFAV-
LPVLVTPAVGRVAADEQVLGARVPCDASL-
RELQKSMSGDALPISGIRLLGMRAARAMVDEDIMVAGKVAA-
LLVILALETITHLVLRNDSEATLPWRLSVAERLEADMKRASGYAARPFKSKPSLCNTQAHAKIADPIQEI
AAGYVAKDERFVFPVEGIPIQLILASPHSRVGSAKRAGLGHLHEMAKELHAKAVIMVLNEPETNTDGLL
NYLTTHQPTDLLFEDCLDVVIDMRRMNVV---
VQLNTV-
HIVIPGARELGDREIMAAAAPSGLVQVERVDDRTGRERTLMWMYGAMEELWLLSVTYADWVVNCAI
GAILLHVYAQVINRAFSLDRLQLAGWPRDASRWWNTWQLWLNKDGFFGYEAAFRAVLAVLSADLD
DYEMSDVEGVGRHLSRQQLRVGYRIREQVAFEQLDPRTDILLQSRTRLSVIRHALAMVAILIVRDIVVE
FTLVVAKEDVAMGHVECVLADSFDFTAKDADIGPVGIRTPDTVLGDGTVFNEAVLGKIDFANTVADF
RYKMLADGGRDLGVSWITLQNMLYARIWLPPAQAALKNLVPIAFFLGHTELNLAIFPALWAGISKARA
QKLMAGALIEKILAKVAAFADPEATTHQSKKAPHFKYYGEEVDPVYLAYILEIFALVATQVTVSDRLLTD
HYVLNLKI-RGFTSLLWLSAQIADKGPAATVITVDTIMGLGALVLFIILL-RTSVLVETLYYRYVYV-

MLGYQAAKDVADADPEHFLSLAHASRDVEYIEIIPAFFYYFKTAT-
FEPFNTALAREFFGLFEERLRIVEVLAVPPDRVLELVQFVALAGTLPTSADAAPGVIACGFCEPNAAGVL
EADQDWNLHRSMGITLAFYDARESVKGWFDKGLHKDVAATGLREAGALTTPFHAEVTAKQCVPAV
HRVLDLGDVEAALIEISSAVLVADEEQTLADKPTTRENSVGAVGGVVLAGTMNMGANLDSVERGGNI
IIELEEEHYSAVNGKKADGKLTDKVLMVLSEAYFPVLRRMASANALDKGRFGLLIDAPDDADNQDHG
RSLTIPAPTVHSESASLFAETEIERIAWIAPIHLLGTLDAVLGFEPDQYSFISMMIDASLNIMKTAEVLDDY
HVLEKGSRPPSFRIILIKGLHIRLLAGICTIIVVGSAGRAEVTAYIQG-
AVPITLNKQTFLPAFAASSCHAIDIFSTMLRPPERSTIQTLILETVTEL-
LEVTRLENDKLERMETLLPGLDIAELLGLLAAAADFTAIVTIDRAITAILSVAAEAVVLREMLLTLAEIAVT
ALALALALLLMLTKDQGLHQKDHAREAQMKELIQGGVVLQVKAAERMMLASTADKVRLDTDEGTTS
AFASLNLATVSDGFYVLSSSVLQMSSDLMMIIKVARVLADKETKNALVVRHPEQVKTQGTYAEGSPG
MVLQTLTGAIPPLAIKPEAVELLWTLVAALWKVKAHLANAARPHQERVLILILALSLITVLLQSEGGGM
GGG--
NPPAKMLAAFSILTIKAKAGDEVRKVTRRVMDLNSRLGRPEESVNPEFRGAELEQSKYEADNVEVDTV
ETIRRFVDIPFLVSIRTQHNESEDRGARMVLDVTRIRFIDL-
VTKKSEIILAARAETDGIIAREMGKDLAALHPQAAALGKIAALQHFIAVQDAHISNYVTADIHAVLGML
LLVLFIQEVRVAKVVWPTRREVTMVFILAALAIFFLVIGLGVQIVFMLSGISWNALTTIVLGEGIMVVRQT
STFPEDS-
IGILQNTFAELICSGRVAKADIENSLHIVVAGRVARGTWYVIYLYEINQAQGQSGIMQRAFRQMTLSQ
SLIDGRMEVKIKVVTSDRYA-
TYEIKGGQHRKGTRYNRVKVGHNIPVQVLPRMKMAFTPPNLVLVKTFGAVVQKRFAQALTHIRHGA
VTIGPMEVSSPYIQNKKARHAPGDAVVDLKPDNIVRSVAVVPELIWARENEDQGAVHDKYVKTTHKL
AAKRKKAVDKAQYDHRNQVELSFIGLAVKVLPEQA--------------------------------------
-----
RMRPQLNLAGKQDKKIVKCLNNNLDDVEYGKLGRRKIKPLTIKSVYDVESRRATELVKLMPYKDDVES
RKSQFAEK--
ALFEPLKAFMMMFGFIARAVYALYLRVIRRTILLAKTKVVVNADSVLSAIKIIGVSIRLRHKSTNVAETNH
HIKTIEKRARMTLAGAGKVKLNLSPGKIGMKGGRIKGYMPYKNFNIFDGLASGRRAKVQDLAKLKDVD
KTISVDGIRQKQQGGRTPKAQLSNAI-
NTRVGVDIVGENEQEESNPRANAEMGQIFAAIDARVFSHVGALIGHALEAVLAVVAIWGIGRAHSLL
AVRLHINAGIEVHSAA-VPRAVAAA-
FAENAGEASLVADVSEGDLGALAVTAMSVDVCRLRRRSLAE-------------------------------
----
RHGIRAKGTYGKERAMMQILPTKPVKIWVPVFVSEEELLAPIRAFAETQGDGVTVVKLTTDIKVTMFEK
AVASLAVTGKATTVVYTVLEKVTELMKDIIYTQRAKAHYGRGRLRSHVVTTTKQRRHLSMIKRTVVAD
PSMEAARLTDGEVSVITGATMAFVMSEQEAV--
GDVDLHFHEEPRREVELGIGGPETFLTTEQPRLLAVLTIREYLLHIVEIGTTNGFSDAATIYDVLRAMVYK
LDRKLSADIAKTRMVLVDDLMKMGVTEVAARREAAGDKILSSYWINGVTANADIVLLVANLECPCVP
ENINCAIRLFYQRITLQAKERGGDVSVAVVAADKQIEAAEGTVIYTIKGKTPGTAQMKLTIEGISRSVVK
SFAVQLFQIDTFMDDTWIATWLRAADDVVFVAAEDGKEAVDHVVERDTAVEFAPQKNIVSRKIRIYT
PAAPSAVVIWDPAIDREDKAEAQGTILKIYGPNLQDINCFTDIGAEPKIESLKAWDEKEKYVVHRDDIP
DDIDTLVGKSSRVNKEFAIPDVAVCAPSKPGVTESIDRGEPKAGTCGEVANMAYPQESLVNAVSSAN
VQKNISMVLAGHPVGEMGISREIERHLETNMCEIFVDKPDVDVALDFYTLVLTVLRVGLDPPNARVQI
DGKESRKLMAIQDGDAALQRHPKVSAATGTGLGAGVKARMPDDALILDGVVHGALSFTATDLPAGR

V-
VTPVFNTVKAQLKIDKVLLKSFHVVTLSFSNNKGEEHLVKLRLNALKKNTRHYEMLLKIRETECAESPHA
ISVGKVLKIDKLLQTICVAHYKAIEPLLADDLTALGVMGRQGLLHVVPRLMILAVLHLI-
ISCAMILPRWRLQQKAIITDQTMGLAVRARAWSY-AISEVVGNTFFARTKDDDTEQRIHARNPV-
VLL-LGKFVMI-
ISPSGLGLKSTAATDQDLGNAEPLILSPERLQDEMGYYVIAILLVFVDQVILAYGSGEAPIKFLVADQAV
TGKQNPGMVTLTRRTFPLELQRDATFVMVFEDSTMKNLSHKKMHMNLVLIKSKGWLVNVSTKILISD
QAIASYAEDAGAHTQPSESALAVITSRVMAPIVLVVSQKPTAPSKSLFLEALALYDEVTATVLNKPKKYF
IFEGDVVSVLGGLDSAVLLGMQRPQPDAMRRAIVAAAIRLAIAALASGIVTRLEEGLINFSRAVSFTR-
AYL----------------AFRVRNHDVSTKRSKPYKAHTKFLHKADVDKSLIAEGVDTLA
>GCA_000423645
AVVKPVTINVYAPVLMIRQSQVATVSGAGSPSLRAKTVYAKSGLQRKVSTKWVAKPLYF-
GVLKMGKIVQLVSISVDARTVCTVAQVVEQEVVVHGLVALDAVKRKIALEEIDGMTTEDPAKVTEFEE
EIDLRRFRDAEDRADMLPVLLGLTLKIAFDPHQPVVHDLPIPVVFEVV-
GIMADRGGIEVDDCAISTALSDVDPIEYVSPIDLEAVAGDAMAFIDQEAAISAAKQAIELEELALESTVL
VVGIKPLLLPV-FGDDAGV-MSFAARSRTLASIVDLAVSADEQTRARIE-GIAAEM-
MKRIQAAALAERRGTAEEIRIVKRMQRAAGLADDPDLVMLGDAAEGNSAWAGQIYDDPISGSSESD
SAGNIVYAANKRQSLQRSITHLRKVEISDKATAPLMRGVKHNSKSRSARKVDDAIAVLNFANVPEGIAI
RAEAGTPADTLLHDATEGERILLIVIRWRVTWKTAVVIIIIITAGDIVGLIELGGIGAVGKPDGLLNRHHKL
NGSLIEQRPIVEKLTLRLRASLKQKLGKEQIIIFVVRVQGDLVADAKVQMG-
GTLGAVVAVQADVIFVRRHKQTLVTDQEATV-ILKGVAEGPADAEALLRIRVEHTLVELSVLLIPR-
PVLCDTVSIREGVGKALQIIQLDVYLLRDMGTGEETLAESVSRELVRDILMIDTVTGVRLAYGKVQERM
GRKLTLKEIEAL---
LSILDGVTEPRMENLAEGVRRCFLQAGPRAAVGMLFLLLDDAGKLYEIDGELREITKTVKSMIGLVDAK
APKVLSAGYATARLKIRFADCEVLQAEADLHPYKEERRELMRMVPVYIIKEAKRRQLVLFALSLRGGHR
RAGAALDVLIAAAIIESQAVKDAVHHAEPRIILDDAEIVGTAIMISRQAALKLKQRVRVDVVVIFPEFQE
KMVVSLRFFCTEVAIFVIALIVTVGDMLSRNPDHAQEDGAGFQKRIRGHSMLFALAATFERRNCGDA-
RADLFLGLGMAPPRYGILYTNQPKKKQMPSEPDAERVDGFELVHAIKADTRKNHELEMPEAMALAVE
-DATINLSAGVLATKGGMAAVERVDSKRKLSLAAEKWNISKLGLHDLLISGAAPVRAAG-
IEQADISAALEAKAV--
LYLRGASNAQLEEEKLGGAGNSSANIAEDRQRQILLVHPVLVVIQDSNTAGLIANGGIDEWGTMAFK
KHYAFAARMRLHMRTIN------EMEPNKQKSEKIKTVKLEGDTRFRQLATDGVKEQMP-
LHALNPDFAVLRAVLVSPAVGRVAASEALLGAAVPCGASL-
RELQKSQKGSALPISGIRLLPMRAARAMIDEDIMVAGKVAA-
LLKILALETIAALVLRNDSEATLPWRLSVAERLEADTKRASGYAARPFASKPSLCHTQAHSRVADPIETIA
AGYVAAEKRFVFPVEEIPIQIVLANPHSRVGSAKRAGEGHLHEMAKELVAKAVLMLQNEPETNTEGLL
NYLTTNQPTDLLFEDCLDVIIDMRRMSVV---
IQLNTV-
HVLIPGTRDIGDRGIMAAAADSGLVQVEQVDERTGRERTLMWMYGAMEDLWLLSVRYADWVPQC
PIGAIILHVYAQKINRAFSLRRLQLEGWPREAARWWNTWQLWLNKDGFFGYDAAFRAAKQVLKTAL
LDYDMSDVEAVGRMQSRQELRVGYRIRQQVSFARLDPRTDILLQAQTRLSVIRHALQVVAILILREVV
GEFTLVVAKEDVAMGHVECVLAESFDFTEKNAHIGPVGLRDPETVLGDGTVLNEAVLGKIDLANTIAD
YRYLVIVDGGRELGVSWLSL-NMLYPRIWLPPAQAALKNLEPICFFLSHPELDLESGDGSFE-
ISKTRADQLMASALVEEILEDVARFAEPEATTHQSNKERHFKYYGEEVDPVHLAYILSMFALVATQVTIS

DRLLKDRYVLNLRV-
RGFTSLLWVAANFGEKGPSATVITVGTIAGLGALVLFIILLMRDRVIVETLFYRYVY--
MRGYEAAKDVADADPEHFLELAAVSRDAEWIEIIPAFFYYFRTAG-
FEPFNTAVAKEFFGLFEERLRVIDVLAVPPDRVEALVEFVALAGTLPTSAEALTGVIAAGLCEPNAAAVL
EADRDWNLHRTMGITLAFFKAQVSVKGWFDKGLHKEVAETGLTEAGGFTTTNLCEVEARKCVPALH
RQIDLGEIEAGLIEIDSAVTVADEEQTLADRPTTREQTVGAVGGVVLAGTMKMGASLEQVERSNNIIIEL
EEEQYHAVNGKKGDGKLTDGVLMVISKAYFPVLRRAASATALNQGVFDLLISAPTDADNRDHGREM
SIPAPSVHTETAALYAEAEIDRIAWIAPLHMLGTLDAVLLFEPDQYSFISMMIEASLDIMETAEV--
DYHVLERGGRPPSFRVILIKGLHIRLMAAIGTIIVVGSAGRAEVTAYIQGRAVPITLNKHTYLPAFAPSSC
HVVDIFSTMLRPPERSTVQTLILETIAEL-
LEVTRLENDKLERLLMLLPGLDMAEMLGLRATADDFASIVTIDAAITAILSVAAEAVVLREMLLSLAEIAV
TALAVAMALLLMLTGDLGLTQRETARERQLKELIQGGVVLQVHALDLMVLAATADKVRLETDEGTTS
AFASLNLATVSDGFYVMSSAVLQLSPDLMMIVSVARVLADKETKNALVVRHPEQVKTQPTYAEGSPG
MVLQTLTKCIPPLAIKPDAVELLWTLVAALWRVKAHLAEAMRPVRERILILLLALALIVVLIQSEGGGMG
GGGVSAAARVLAAFSLLTIKAAAGDEVRKVARRVMDLNGRLGRPEESVNPEFRGEELEQTKYEAENVE
VDCMETIRRFVDIPFLVSIRTQHNESEDRGARMVLDVTRVRFIDL-
VTKRSEIILAARVETDGIISRDMAKDLQALHPELAALGKIAALEHFISLTDGHISNYVTADIHSVLGMLLL
VLFIQQVRVAKVVWPTRREVTMVFIMAALALFFLVIGL--
NLVFMLGGISPLQITTLVLGEGVMAVRQTSTFPEDS-
VTILQNTYADLIAAGRVAKADIEASLHIVVGARVARGTWYVIYHYEITQAQGEAGILQRAFRQATLSKS
LIDGRLEVKIKVVTSDRYA-
TYEIKGGQHRKGTRYNRVKVGHNIPVEILPRLKLAFHPPNLVLVKTFGAVVQKRFAQPLTHMRHGAVT
IGPMEVSTPYIKNKKSRHAPGDSVVGVKPDNIVRSVAVVPELIWARENEDNSAVHDKYVKTTHKVAA
SRNRAVDKAQYDKRNQVHLSFIGLAVKVLPDQA--

RMRPQLNLAGQTDKKIVKCLNNDLDDVEYGKLGRRKIKPLTIKSVYDVEHRRATELVKLMPYKDDVES
RKSQFAEK--ALFEPLKAFMMLFGFIARAVYALYLRVVRRTILLVKTKIVVNAKSVLS-
IKIIGVSIRLRHKQTNIADTKHYIKTIEARARMTLAGAPAVKLNLRDGRVGTKGGRIKGYMPYNGYNVF
DGLDSGRRAKVRELAKLKKVDKTISVDGIRTRQQGGRIPQADLSNAI-
NTRVGVDIVGENKQAASNPRANAEMGQIFAAIDARVYHLVAALIGHALAA-
LARCAIQGIGQAHALLAMKLEINAGIEFHSAA-VETAVAAA-
FAEPAGEAAVIADVSEAYLGDLSISDASLDVCRMRRRLA---------------------------------
----
RHGIHAKGTYGKERAMMQITPTKPVKVWVPIFVDESELLAPIRAVAETNGDGVTVVKLTKDVKITMFE
KAVASLAVAGKATTIVYTVLEKVTELMKEIIYEQRSKTHYGRGRLRSHVVTNTKSKRHLSMMRRTVVAD
PRMDAARLTDGEVTVITGATMAFVMSEQDAV--
GDVRMHFHEEPRREAGLGIAGPETFLATEQPRLLALLFLRAYLIHLVDIGTVGGYAEAATIFDILRAMYN
RLDRKLAADIAKTRMVLHDDVQNMGVRAESPRRETAGDSILSAYWINGVTANADIILLVANLETTMV
PENINCAVRLFYQRVTLQAKERDGDVSVHLVPAQNQIEAAEGTEIYTIKGATTGTTQMRLTIEGISRSIV
KSLAVQLFQVALFINDTWIDTLLRAANDVVYVDAREGKEAIDHVVERDTELEMAPQKDIVRRKIRIYTP
AAPSAVVIWDAAIDRSDKADAQGTILKVYGPDLQDMACFTDIGAEPKIESLKSWDEKERYVVHRDDIP
PDI-
TMVGKSARVNKEFGIPDVAVCAPSKPGVTESIDRGEPKAGSCGEVANLPYPQESLVNRVSSANVQKN
ISMVLGGHPVGEMGISRAIERHLETNMCEIFVDSPNVDVALDFYTIELTPLRVGLDSPNAKVEISGKESR

KLMAVLDGEADLQRHPKVSLAAGQVGKVGERARLPDDALIRDGVPHGDISFTATDLPEGRV-
VTPVVNNVKAQLEIDKVMMKTFHVVTLSFSSDKGDEHLIKLRLNALKKSTRHYEMLLKIRDTECAQSP
HAIPVGKILKIESLLQTICQAHYTEIEPLLSDDLTALGVMGDQGLLHIVPRLMILAVLRMWGLEARALAA
HWDVQAQAIVTDQTMGMPLRARRWGY-AISEVVGNTFFARTREDDTETRLHARNPI-MIL-
YGKEGLL-
ISPSGLRLDSTTETADDHGNNDQLILSKEHLQDAMEYYILRVLLVFVDEVIVGWGSGDEPLKFLVADQ
AVTGRANPGLVCLTRRTFPLDLQRDAVFVLVFEDCTMKNLSHKKMHMNLVLIKSKGWVVNVSTKILIS
DQAISSYAEDAGAHTQPSESALAVITSRVIAPIVLVVSQKPAAPSKTMFLEALSLYDEITCTILKEPKKYYV
FEADVISVLNSLDAAVILGMQRPQPDRMRRAVVAEAIRLAIALQGSGVLASLEGGAINFSRAVSLAAG
VYPSLALVPHAPID-PSIEFRVRNHKTSSKRDRPYKGHTKFLHKAEQEKGLVADGVDTIA
>GCA_000442275
AVIKPVTQNTYAPVLMIRQTQVATVSGAGSPSLRAKTVYAKSALQRKTETKWVAKPLYY-
GVLKMGNIVQLVSISVDNRSVCTVAQLVEQEVVVHGIVALEAVKRKIALEEISGMTAEDPAKVVHYDE
EIQLKRLRDIEDRADMLPVLFGLTLGIKFDPHEPLVHDLPAPVIYEVM-
GVMAEKGGIEVDDCAISTALSDVDPIEYVSPIDLEAVKGEAMAYIDQEAAVSAAKETAELEELALESTVL
VVKINPLILVTPFAKAADT-MSFANRSRTLESIVDLAVASYELTRA-IS-DFASEM-
MARAKAEALSDKRGTAEEIRIVKRMQKSSGLADDPDLVILEDAVEGNSSWAGQIYDDPISGSSESDSA
GNIVYAANKRQTTERSITFLRNVEISDSADAPLMRGVKHNAKSRSSRKLDDAVAELNFPNVPEGIALR
AAANKPKDTLLHDAAEGERILLVIVRWHVTWKTAIIVIFILEAGNVAGLIDMGGIAAVGRPDGLLNRHH
KLNGSLIEQRPIIEKMTLRLRASLRQKLGKDQIIIFVVKVAGDLVAAAKVQM---
GVGAVVAIQGVTIFVRRHRQTLITDAESTV-ILSGVAEGPADAEALLRIRVTHTLVDISVLLIPE-
PVLVEGVSIREGVGKALQIIQLDVYLLRDMGTGEETLAESKSRELIREVLSIDPITSVRLAFGKLKERATKQ
MSVKDIDTLF--
IAIMVAITEPRMENLAEGVCRNFIRAGPRAAVGMLFILLDDDGKLYTIDGELRELTKSIKSMISLVDAKAP
KVLSAVYATARLKVRFADCEVLNAEADLYPYKDERQELLRMVPVYIILEAKRRSLIVFALSLKGGHRRAA
AALEVLIAAAILESQAERGGVDHAAPAIVLSEAELVGSEIMISRQAALEIKLRFKIDVIVIFPEFQEKMVVS
LRFFCTEVAIFVIALITTVGEMLSREPSSAQESGAGFQK---AHSVLFALAVCFERSNLRDA--
PDLFLALGLTDPKYGIFYVNQPKKKQMPSEPDKDRTDGFELVHAIKADSAKNHELEMPEAMALAVE-
NESINLSAGVLALSAEMVDVEKVDSKRKMALAVEKWTIAKVPIVDVKLSGAAPVRAMD-
VEQADVTAALSKKQLNELFLPGATNKDLEEDKLGGAGASTADAAADRQERILVVRAVLVVIQDSNTA
GLISNGGVEEWGTMAYKKHYAFAARMRLHMRTID-------
MEPNKQKAEKIKTVKLDGDTRFRLLAVGGVKEQMP-LHALNPDFAV-
LAVLVTPAVGRVAATAELVGGQVPCDASL-
NELQKSQRGDALPISAMRILGMRAARGMIEEDIMVAGKIPASLLVLSALETIMKIVLLNDSEATVPWRL
SVAERLEADTKRAGGFAARPFNSKPSLCKIQAHARVADPIQEIAAGYVALEEKFVFPVEGIAIQLILASPH
SRVGGAKRSGEGHLHEMAKELFAKAVLMARNEPGTDTEGLLRYLTTQQPTDLLLEESLDIVIDLRRMQ
VV--VQLTTV-
HVVVPGIRDVGDYEIMAAAAPSGLVQIERVDDRTGRERTSMWMYGSMEELWLMSAKWADWVIDT
PIGAIILAVYAQDINRAFSYKRLQLDGWPPEASRWWNTWQLWLNKDGFFGYEAAFRASKQVLKADL
LDYQMKDVEGVGRMQSRQELRVGYRIREQVVHEQLDPRTDILLQSRTRLSMLRHALKVVAILVLREV
VTEFTLVVAQEDVAMGHVECVLADSFEFTAKDADVGPVGLRTPDEIFGDGTVFKDAVLGKVDLANTV
AEFRYLVIIDGGRELGVEWVTLENMLYARIWLPPAPAALKDLLPIKFFLSHPELDLDWQLHWFNGISKA
RAQRLMASALVEGILDGVARFADPEATTHQSAKEKHFKYYGEEVDPVYLAYILSLFALVATQVTVSDRL
LKDRYVLNLKI-RSLTSLLWVAANLGDKGPTSTVITLGTIAGLGALVLFIILL-RDLVLVETLYYRYVY--

MLGYQAAKDVADADPEHLLQLAAASRDVEWIEIIPAFFYYFKTAG-
FEPFNTAVAREFFGLFEERLKVVDVLAVPPDRVEALVEFVALAGTLKTSADALPGIIACGLTEPNAAAVL
EADQDWNLHRAMGITLAFFEARESVKGYFDKGLHKAVAATGLKEAGALSDPFHCKMTAKQCVPVV
HRLVDLGEIEAALIEIDSAVEVADEEQTLADQPTTRDKSVGAVGGVILAGTMAMGASLDSVERGGNIII
ELEEEQYHAVNAKKADGKMIDKVLMVVSAAYFPVIQRCSSANALDRGVFNLLIDAPSDADNLDHGR
KLSIPAPTVH-
ETALLYAEDEIDRIAWVAPLHLLGTIPAVLLFEPDQYSFISMMIDASLEITETAEVLDDYHVLETGSRPPA
FRII--
KGLHIRLMSAIGTIIVVGSAGRDEITAYIETGAVPISLKKETFLPAFAASSCHAIDIFSTMLRPPERSSVMT
MILETVAEL-LEVTRFENDKLERM-
ILLPGLDVAELLELVAEAADFSTIVTIDAAITAILSVAAEAVVLRELLLTLAEIAITALALAMALLLMLTRDQ
GLTQKERARETQMKELIQGGVVLQVKVADSMVLASTADKVRLNTDEGTTSAFASLNLATVSDGFYVL
SSAVLQMSADLMMIIEVARVLADKETKNALVVRDPEQVKTQSTYAEGSPGMVLQTLTGCVPPLAVKP
EEVELLWTLVKALWRVKAHLASGARPRRERVLVLIIALCLIIVLIQSEGGGMGGG-
ASPAAKLFGAFSILTIKAAVGDEVRKVARRVMDLNSRLGKPEEAVTPEFRGAELEQTKYEAENVEVDT
METIRRFVDIPFLVSTRTQHNESEDRGSRMVLDVTRVRFIDL-
VTKRSEIILVERVETDGIIAREMAGDLQALQPEAAALGKIAALEHYISLVDAHVSNYVTAEIHSVLGMLLL
VLFIQQVRVAKVVWPTRREVTMVFIMATIAIFFLVIGLSVQIVFVLGGISPQQITTLVLGEGVVAVRQTS
TFPAEA-
VTIMQNTYADLICAGRVTKADIEASLHIVVGARVARGTWYVIYHYEISQAQAPAGIMQRAFRQATLS
NSLIDGRMEVKIKVVTSDRYAQTYEIKGGQHRKGTKYNRVKVGHNIPVEVLPRMKMAFGPPNLILVKT
FGAVVQKRFAQPLTHVRHGAVTIGPMEVASPYIVMKKARHAPGDSAVDLKPELIVRSVAVVPELIWA
REHEDNSAVHDKYVTVTHKIAAKRNTAVDKAQYDKRNQCELSFIGLAVKVLPEKA--------------
----
RMRPQLNLQGKADKKIVKCLNNNLDDVEYGKLGRRKIKPLTIKSVYDVEHERATELVKLMPYKDDVES
RKSQFAEK--
AIFEPLKAFMMLFGFIARAIYALSLRVIRRTILLAKTKIVVNANTVLSAIKIIGVSIRLRHKSTNIADTNHHIK
TIEKRARMTLASAPSVKLHLRPGKVGKKGGRIKGYMPYKNFNIFDKLEAGRRAKVKELAKLKKVDKTIA
VEGIRVKQQGGRVPQAQLSNALANTRVGVDIVGENEQEDSNPRANAEMGQIFAAIDARVYHLVGA
LLGHALLAVLAIAAIWGISKAHALLAVKLRVNAGIEFHSAA-VPTAVAAA-YAQPAGEA-
AIADVAEYPLGDLGVTDATLDVCQRPRKRLSE--
RHGIRAKGTYGKERAMMQILPTKPVKVWVPMFVNDDELLAPIRQFAETNGDGVTVVKLTKDIKVTMF
EKAVASLAVAGKVTSVVYTVLEKVTELMKDIIYEQRAKAHYGRGRLRSHVVTHSKQKRHLSMIRRSVV
ADPSMDASRLVNGEVSVITGATMAFVMSEQDAV--
GDVDMHYHEEPRREAQLGIAGPETFLSTEQPRLLCLVFLREYLIHIVDIDTVGGFSDAATIYDILRAMQR
KLDRRLAAEIAKVRMVLADDVAGMGVTEVAAKRDTAGDEILSAYFINGVTANADIILLVANLECKCAP
ENIHCAIRLFYQRITLQAKERGGDVSVAIVPAQNQIEAAEGTVIYSIKGKTPSTTQMKLNIEAISRSIVKSL
AVQLFQVDTFLADTWIDTLLRAAAKVVFVDAKEGKEAVDHVVERDTEIELAPQKDIIRRKIRIYTPAAPS
AVVIWDPAIERE-
KADAQGTILKIYGPDLQDISCFTDIGAEPKIESLKSWDEKEKYVVHRDDIPEDIDTLVGKSSHVNKEFGIP
DVEVCAPAKPGVTESIDRGEPKAGSCGEVANLPYPQESMVNAVSSANVQKNISMVLGGHPVGEMGI
SRAIERHLETNMCEIFVDKDDVDVALDFYIIQLTTLRVGIDPPNAKVEIEGKESRKLMAVRDGAASLQR
HPKVSEAAGTRIGAGRKARLPDDALILDGVLHGDLKFTATDLPEGRV-
VTPVFNDVKAQLKIDKVILKSFNVVTLSFSNNKGEDHLIKLRLNALKKNTRHYEMLLKIRETECAESPHAI

SVGKILKIDSLLQTICTAHYTEIEPLLAEDLTVLGVLGDQGLLHVVPRLMILAVLHLR-
VGIAMFVPHWGLQQRAIVTDDTMGLRVRMRDWSY-TVSEVVGNTFFARTKDDDTEQRLHARNPI-
MIL-YGKEGML-
ISPSGLGLKSTPESDMEEDNDKEVILSVEKLQDAMEYYVLQLLLVFIDEIILAYGSGTEPLKFLVADQAIT
GRDNPQLVALTRRTFPLDLERDATFVLVFEDATMKNLSHKKMHMNLVLIKSKGWVVNVSTKILISDQ
AIASYAEDAGAHTQPSESALAAISSRVMAPIVLVVSQKPTAPSKTLFLEALNLLDEVTSTVVNKPKRYFV
FEGDVISELGTMDAAVLKGIQRPQPDSMRRAVVATAIRLAIAIQASGILASLEAGRINFSRAVSLAA-
TYALLDIALEAVIG--ALAFRVRNHKVNTKRARPYKGHPKVLHKADIDKKLIAAGVDTIA
>GCA_000473145
AVIKPVEINKYAPVLMIRQQEVATVSGAGSPSLRAKTVYAKSGLQRKTMTKWVAKPIYF-
GVLKMGNIVQLVSISVDNRAVCTVAQVVEQEVVVHGLVALDAVKRKIALEEMGGMTTEDPAKVAEF
EAEIDLKRFRDADARADMLPVMLGLTLEVKFDPHEPVVHDLAVPVIYEVI-
GIMADKGGIEVDDCAISNAVSDVDPIAYVSPIDLEAVAGEQMAFIDQEAAVAAARETIALEELALEAGI
LVVGIDPLLLPV-FGEDADV-MSFAARSRTLGSIVALAVAYEELTRA-IDG-IASRM-
MAAAQAAALAEKRGTAEEIRIVKRMQRSSGLADDPELVILEDSAEGNSSWAGQIYDDPVSGSSESDS
AGNIVYAANKRQNFARSITFLRKVEISDKAAAPLMRGVKHNSKSRSARKLDDAIAGLNFPNVPPGIAIR
AEAGRTADRLLHDAAEGELILMIVVRWHVTWKTAVAIALVYQAEDLVGLIELGGIAAVGRPDGILNRH
HKLNGSLIEQRPIVEKLTLRLRASLKEKLGKDQVIIFVVKVQGDLVADATIQMG-
GPVGAVVAIQGELIFVRRHKQTVITDQEATV-VLKGVAEGPADAQALLRIRVTHTLVGLSVLLIPE-
PVLVESVQIREGVGKSLQIIQLDVYLLRDMGTGEETLAESRSRELIREVLAVDPITGVRLAFGKLKERMGK
KLGLKDLE--
GKLYQIDGDLRELPKSVKSLIGLVDTKAPKVLSAGYAEERLRIRFADCEVLGTDANLYPYKEERRELLRM
VPVYLILEAKRRQLIIFALSLKGGHRRTMMALGVMLAAAIIESQAEKGEVHHETPEILLDDAEVVGTSIM
LSRQASLDLKQINKVDVIVLFPEFQEKMVVSLKFFCTEVAIFVIALITTVGEMLSREPEHAQEEGAGFQK
RIRGHSMLFALAVVFDRRNCRDA-
LPDLFLALGLAEPRYGILYTNQPKKKQMPSEPDGDRVDGFELVHAVKEDGAS-
HELEMPEAMALAVE-
DATINLSAGVLAMNGGMVAIEKVDSKRKVALAATKWNISKLELADVHISGSAPTRSCG-
IEQAELSAALEAKKVWFLFLPGASNAELEEAKLDGAGASTADAADDKQIHILLVKPVLVVIQDSNSAGL
ISNGGLDEWGTMAYKKHYAFAARMRLHMRTID------
DMAPNKQKSEKVKTVKLEGDTRFRQLATDGVKEQMP-LHALNPDFAV-
LPLLVTPAVGSVAADENVLGAQVPVGASL-
RELQKSQKGSALPISAVRLLAMRAARGMIEEDIMVAGKVAA-
LLKIRALETVIAILLRNDTEATLPWRLSVAEKLEADTKRASGFAARPFKTKPSLCNTQAHAKIADPIQEIA
AGYVAAEERFVFPVEDIAIRLILSNPHSRVEEKKRGGEGHLHGMAKELLAKAVVMLRNEPGTNTEGLL
NYLTTNQPTDLLFEDCLDVIIDIRRMSVV--
VHLNTV-
HVVVAGVRSVGDYEIMSAKFPSGLVQVERVDDRTGREKTLMWMYGAMEELWLMSARWADWVPD
CAIGAIILHVYAQEINRAFSLKRLQLDGWPKEASRWWNTWQLWLNKDGFFGYEAAFRAVKQVLKAT
LFDFEMSNVEGVGRYQSKQELRVGYRIRQQVEFARLDPRTDILLQAKTRLSVIRHALEVVAILILRDVAP
EFTLVVAYQDVARGHVECVLAESFDFTEKNAHIGPVGLRTPASIFGDGTVLKEAVLGKIDLANTVAEFR
YLVILDGGRTLGVEWISLRNMLYERIWLPPAQAALKNLLPICFFLQHPELDLAWA-
ALFEGISKARADKLMAGALVEEILEGVARFADPEATTHQSKKAKHFKYYGEEVDPVHLAYILDLFALVST
QVVVSDRLLKDRYVLNLKVFRGFTALWWMALQLSPKGPSATVITVGTIAGLGALVLFIILL-

RDSVLVETLYYRYVY--MKGYEAAKDVQDADPEHLGQLAAASRDIEWIEIIPAFFYYFDTAT-
FEPFNTAVAKEFFGLFEERLRIIDVLAVPPDRVAALVEFVALAGTLATSAEALVGVIACGLCEPNAAEVLE
AERDWNLHRSMGITLAFFEARESVKGYFDKGLHKDVAEIGLKEAGALTTHFRCDVDAKQIAPAVHRIL
DLGEIEAALIEIDSAVVVADEEQTLADKPTTRDKSVGAIGGVVLAGTMNMGANLDSVERGGNIIIELEE
EHYHAVNGKKADGKLTDKVLMVLSVAYFPVIRRFASANALDRGVFGLLIDAPEDADNLDHGRVMTIP
APSVLAETTPLYAEAEIDKIAWIAPLHLLGTLPAVL-
FEPDFQGFISMMIDASLEIMETAEVLDDYHVLERGSRPPKFRVILIKGLHIRLLAAAGTILVVGSAGRTEV
TAYIQGQAVPISLSKNTFLPAFSPSSCKAVDIFSTLLRPPERSAVEMLILETVAEL-
LEVTRLSNDKLERLLVLLPGLDMAELLGLLAAAQDFADIVTLGAAITAILSVAAESVVLREMLLTKAEIAIT
ALALAIALLLMLTGDEGLTQKEQAREAQMKELIQAGAVLQIHAADAMVLASTADKVRLDTDEGTTSA
FASLNLATVSDGFYVLSSAVLQMSSDLMMIVAVARVLADKESKNALVVRHPEQVKTQPTYAEGSPG
MVLQTLTGCIPPLAIKPEEVELLWTLVAALWRVKAHLASAARPHTERVLILLLALGLIVVLLQSEGGGM
GGG-
IGAAAKLLAAFSILTMKAAVGDEVRKVTRRVMDLSSRLGRPEETVNPEFKGEELDQTKYEAENVEVDC
VETVRRFVDIPFLVSVRTQHNESEDRGARLVLDVSRVRFIDL-
VTKKSDVILVERVETDGIIARQMAKDMQSLHDEAAALGKVSQLQHFIALGDAHISNYVTAEAHSVLG
MLLLVLFIQQVRVSKVVWPTRREVTMVFIMAALALFFLVIGLGLNIVFMVGGISPLQITTLVLGEGVMA
VRQTSTFPAES-
VTILQNTYADLICAGRVTKADIEKSLHIVVGARVARGTWYVIYHYEITQAQSPAGIMQRAFRQATLSQS
LIDGRMEVKIKVVTSDRYA-
TYEIKGGQHRKGTRYNRVKVGHNIPVDVLARIKMAFAPPNLVLVKTFGAVVQKRFAQPLTHMRHGA
VTIGPMEVSSPYIQAKKARHAPGDSVVDIKPDNIVRSVAVVPELIWARENEDQSAVHEKYVKTTHKIA
AKRNRAVDKAQYDKRNQVHLSFIGLAVKVLPEQAIVHATAGTLLPTESVSVTENLKGVAATIRLDPME
KALIAYIKRMRPQLNLQGKTDNKIVKCLNNNLDDVEWGKLGRRKIKPITIKSVYDVEHERATELVKLMP
YKDDVEGRKSQFAEK--
ALFEPLKAFMMLFGFIARAVYALYLRVIRRTILLVKTKIVVNAETVLSAIKIIGVSIRLRHKSTNIADTKHHIK
TIEARARMTLAGAPAVKLHLRPGRVGKKGGRIKGYMPYKNYNIFDGLASGRRAKVKELAKLKKVDKTI
AVEGIRTRQ-
GGRLSKADLSNALSNTRVDVEVVGENVQEASNPRANAEMGQIFAAIDARVYSLVAALIGHALEALLA
LCAILGIGRAHALLAVY-KMNAGIGMHSAA-VAAAVAAA-
YSQPGGEATALWDVSEGDLGALAISEGSLDVCRLRRRQIG-
LKKTKKEAYMSLTCFSGRGDKVKIIEATQTQAYEIVATRTAERHGIHAKGTYGKERAMMQITPTKPVKV
WVPVFVSDEELLAPVRAFAETNGDGVDVVKLAKDVKITMFEKGVASLAVSGKNTTVVYTVLEKVTEL
MKDIIYEQRAKAHYGRGRLRSHVVTHTKQKRHLSMMKRTVVADPSMDAARLSDGEVSVVTGATMA
FVMSEQDAV--
GDVDYHFHEEPRREAQLGIAGPETFLSTEQPRLLAMLFLREYLIHIVDIGTVGGFADAATIYDILRAMQN
KLDRKLSADIAKVRMCLHDDVEGMGVKAEAPRRDQAGDAILSAYWINGVTANNDIILLVANLECKTA
PENINCAVRLFYQRITLQAKERDGDVSVKLVPAQNQIEAAEGTVIYTIKGNNPSTVQMKLNIEGISRSIV
KSLALQLFQVDVFLNDTWIATLLRAAQDVVFVDAREGKEAVDHVIERDTELEMAPQKDIVARKIRVYT
PAAPSAVVIWDPAIEREDKADAQGTILMIYGPDLQDISCFTDIGSEPKIESLAAWDEKEKYVVHRDDIPE
DI-
TLVGKSSKVNKEFGIPDVAVCAPAKPGVTESIDRQEPKAGTCGEVANLPYPQESLVNRVSSANVQKNI
SMVLGGHPVGEMGISRGVERHLETNMCEIFVDKPDIDVALDFYKIALTSLRVGLDKPNAKVEIDGKESR
KLMAVKDGDATLQRHPKVSEESG--RKAGEKAKLPDDALIYDGVQHGDIKFTATDLPDGRV-

VTPVIMSVKAQLEIDKVMLKSFHVVTLSFSNDKAEEHLIKLRLNALKKSTRHYEMLLKIRDTECAQSPHA
IPVGKILKIDALLQTICKAHYTAIEPLLADDLTALGVMGDQGLLHVVPRLMIIALLHLCELGAAAAIPHW
RLQQAAILTDQTMGLAVRERRWSF-AISEVTGNTFFARTKDDDTEQRLHARNPI-VIL-YGKEGML-
ISPSGLRLASTAPSDKGHGNDEKLILSPERLQDAMYYYVLKVLLVFVDEIILAYGSGEGPLKFLVAHQAV
TGRDNPQLVCLTRRTFPLDLQRDATFVLVLADTTMKNLSHKKMHMNLVLVKPKSWVENVSTKILISD
QAIASYAEDAGAHTQPSESALAVITSRVIAPIVLVVSQKPAAPSKTLFLEALNLYDEITATVQKRPKRYFIF
EGDVVSVLGTLDAAVILGMQRPQPDRLRRAVTAHAIRLAIALRGSGVLASLEGGAINFSRAVDFAA-
TYPGLEA-----------DFRVRNHKTSTKRDRPYKGHTKFLHKAEAEKALIAEGVDTIA
>GCA_000473165
AVIKPVEINKYAPVLMIRQQEVATVSGAGSPALRAKTVYAKSGLQRKTLTKWVAKPIYF-
GVLKMGNIVQLVSISVDSRAVCTVAQVVEQEVVVHGLVALDAVKRKIALEDMGGMTTEDPAKVAEF
EAEIDLKRFRDADARADMLPVMLGLTLEVKFDPHEPVVHDLAIPVIYEVI-
GIMADKGGIEVDDCAISNAVSDVDPIAYVSPIDLEAVAGTEMAFIDQEAAVAAARETIGLEELALEAGIL
VVGI-PLLLPV-FGEDGNV-MSFAARSRTLKSIVALAVAYEELTRA-IDG-IASEM-
MAAAQAAALAEKRGTAEEIRIVKRMQRSSGLADDPELVILEDAAEGNSSWAGQIYDDPVSGSSESDS
AGNIVHAANKRQNFARSITYLRKVEISDKAAAPLMRGVKHNSKSRSARKLDDPIADLNFPNVPPGIAIR
ADAGKPGDRLLHDAAEGELLL-
IVVRWHVTWKTAVAIALVYQAENVVGLIALGGIAAVGRPDGILNRHHKLNGSLIEQRPIIEKMTLRLRA
SLKQKLGKDQIIIFVVKVQGDLVADATIQMG-GPVGAVVAIQGELIFVRRHKQTVITDQEATV-
VLKGVAEGPADAQALLRIRVTHTLVGLSVLLIPE-
PVLVEAVQIREGVGKSLQIIQLDVYLLRDMGTGEETLAESKSRELIREVLAVDPITGVRLAFGKLKERAGK
KLGLKDLE-----
QSILAAVTEPRMENLPEGVCRNFIAAGPAAAVGMLFLLLDEAGKLYQIDGEMRELTKSVKSLIGLVDSK
APKVLSAGYAEERLRIRFADCEVLGSDANLYPYKEERRELLRMVPVYLINAAKRRQLIIFALSLKGGHRRT
MMALGVMLAAAIIESQAENGEVHHRAPEILLDDAEVVGTAIMLSRQAALDLKQLGKVDVIVIFPEFQE
KMVVSLKFFCTEVAIFVIALITTVGEMLSREPEHAQEEGAGFQKRIRGHSMLFALAAVFDRRNCRDA-
LPDLFLGLGLAEPRYGILYTNQPKKKQMPAEPDGERVDGFELVHAVKEDATSNHELEMPEAMALAVE
-DATINVSAGVLAMNGGMAAVEKVDSKRKVALAATKWNISKLEIADVHISGAAPVRSCS-
IEQADLSAALESKKVWFLFLPGASNAELEEAKLDGAGASTADAAADKQIAILLVKPVLVVIQDSNSAGL
ISNGGLEEWGTMAYKKHYSFAARMRLHMRTID-------
MAPNKQKSEKVKTVKLEGDTRFRQLAIEGVKEQMP-LHALNPDFAV-
LPLLVTPAVGSVAADENVLGAKVPVDASL-
RELQKSQKGSALPITAIRLLAMRAARGMIEEDIMVAGKVAA-
LLKIRALETVIEILLRNDSEATLPWRLSVAEKLEADTRRASGFAARPFKTKPSLCNTQAHSKIADPIQEIAA
GYVAAEGRFVFPVEEVAIKLILSNPHSRVEEKKRGGEGHLHGMAKELLAKAVVMLRNEPGTNTEGLLN
YLTTNQPTDLLFEDCLDVIIDIRRMSVV--
VHLNTV-
HVVVEGVADVGDYEIMSAKFASGLIQVEQVDDRTGREKTLMWMYGAMEELWLMSVRWADWVAD
CAIGAIILHVYAQEINRAFSLKRLQLDGWPKEAARWWNTWQLWLNKDGFFGYDAAFRAVKQVLKAT
LFDFEMSNVEGVGRYQSKQELRVGYRIRQQVEFARLDPRADILLQAQTRLSIIRHALEVVAILILRDVAP
EFTLVVAYQDVARGHVECVLAESFDFTEKNAHIGPVGLRTPANIFGDGTVLKEAVLGKIDLANTVAEFR
YLVLLDGGRALGVQWISL-NMLYARIWLPPAQAALKNLLPICFFLQHPELDLAW-
AGLFEGISKTRADKLMAGALVEEILEGVARFADPEATTHQSKKAKHFKYYGEEVDPVHLAYILGLFALV
ATQVVVSDRLLKDRYVLNLKVFRGFTSLLWLALQLAAKGPSATVITVGTIAGLGALVLFIILL-

RDSVLVETLYYRYVY--MKGYEAAKDVQDADPEHLGQLAAASRDIEWIEIIPAFFYYFKTAS-
FEPFNTAVAREFFGLFKERLRIIDVLAVPPDRVAALVEFVALAGTLATSAEA-
VGVIACGLCEPNAAEVLEAERDWNLHRSMGITLAFFEARESVKGYFDKGLHKDVAEIGLREAGAFTTH
FRCDVDAKQVAPAVHRTLDLGEIEAGLIEIDSAVVVADEEQTLADKPTTRDKSVGAIGSVVLAGTMN
MGANLDSVERGGNIIIELEEEQYHGVNGKKADGKLTDKVLMVLSVAYFPVIRRFASANALDRGVFGLLI
DAPADADNLDHGGDMTIPAPSVLAETTPLYAESDIDKIAWIAPLHLLGTLPAVLLFEPDFQGFISMMID
ASLEIMETAEVLDDYHVLEKGARPPKFRVILIKGLHIRLLAAIGTLLVVGSAGRTEVTAYIQGQAVPISLRK
HTFLPAFSPSSCKAVDIFSTLLRPPERSSVLMLILETVAEL-
LEVTRGTNEKLERLLVLLPGLDGAELLGLLAAAEDFADIVTLGAAITAILSVAAEAVVLREMLLTKAEIAIT
ALALAIALLLMLTGDEGLTQKWQAREAQMKELIQAGAVLQIHASDAMVLASTADKVRLDTDEGTTS
AFASLNLATVSDGFYVLSSAVLQMSSDLMMIVAVARVLADKETKNALVVRHPEQVKTQPTYAEGSPG
MVLQTLTGCIPPLAVKPEEVELLWTLVAALWRVKAHLASATRPYHERVLILLLALGLIVVLLQSEGGGM
GGG-
VGAAARILAAFSILTMKASVGDEVRKVTRRVMDLSSRLGRPEEAVNPEFKGEELDQTKYEAENVEVDC
METVRRFVDIPFLVSVRTQHNESEDRGARLVLDVSRVRFIDL-
VTKKSDVILVERVETDGIIAREMAKDMQSLHETAAALGKVSQLQHFIALGDAHISNYVTAEAHSVLGM
LLLVLFIQQVRVSKVVWPTRREVTMVFIMAALAVFFLVIGLGLNIVFMVGGISPLQITTLVLGEGVMGV
RQTSTFPAES-
VTILQNTYADLICAGRVTKADIEASLHIVVGARVARGTWYVIYPYEITQAQAPSGIMQRAFRQATLSQS
LIDGRMEVKIKVVTSDRYA-
TYEIKGGQHRKGTRYNRVKVGHNIPVDVLARIKLAFGPPNLVLVKTFGAVVQKRFAQPLTHMRHGAV
TIGPMEVSSPYIQSKKARHAPGDSVVDIKPDNIVRSVAVVPELIWARENEDQSAVHEKYVKTTHKIAAK
RNRAVDKAQYDKRNQVHLSFIGLAVKVLPEQAIVHSTAGTLLPTESVSVTENLKGVAATIRLDPMEKA
LIAYIKRMRPQLNLQGKTDNKIVKCLNNNLDDVEWGKLGRRKIKPITIKSVYDVEHEQATELVKLMPYK
DDVEGRKSQFAEK--
ALFEPLKAFMMLFGFIARAVYALYLRVIRRTIVLVKTKIVANAETVLSAIKIIGVSIRLRHKSTNIADTKHHIK
TIEARARMTLAGAPAVKLHLSPGRVGKKGGRIKGYMPYKNYNIFDGMASGRRAKVADLAKLKKVDKA
IAVDGIRTRQ-
GGRLPKADLSNALSNTRVDVEVVGENVQEASNPRANAEMGQIFAAIDARVYSLVAALIGHALEAVLA
VCAIMGIGRAHALLAVY-KMNAGIGMHSAA-VAAAVAAA-
YSQPGGEATAIADVSEGDLSALAISEGSLDVCRLRRRQIG-
LKKTKKEAYMSLTCFSGKGDKVKVIEATQTQAYEIVATRTAERHGIHAKGTYGKERAMMQITPTKPVK
VWVPVFVTDEELLAPVRALAETSGDGVAVVKLANDVKITMFEKGVASLAISGKNTTVVYTVLEKVTELA
KDIIYEQRAKAHYGRGRLRSHVVTHTKQKRHLSMMKRTVVADPSMDAARLSDGEVSVVTGATMAFV
MSEQDAV--
GDVDFHFHEEPRREAQLGIAGPETFLSTEQPRLLAMLFLREYLMHIVDIGTTGDFADAATIYEILRAMQ
AKLDRKLSADIAKVRMCLHDDVEDMGVKAEAPRRDQAGDAILSAFWINGVTANSDIIMLVANLECKT
APENIDCAVRLFYQRITLQAKERDGDVSVKLVKAQNQIEAAEGTVIYTIKGKTPSTAQMKLNIDAISRSI
VKSLAVQLFQVDIFLNDTWIATLLRAAQDVVFVDAREGKEAVDHVIERDKELELAPQKDIVARKIRVYT
PAAPSAVVIWDPAIEREDKADAQGTILMIYGPDLQDISCFTDIGAEPKIESLAAWDEKEKYVVHRDDIP
EDITTLVGKSSKVNKEFGIPDVAVCAPAKPGVTESIDRGEPKAGTCGEVANLPYPQESLVNRVSSANV
QKNISMVLGGHPVGEMGISREVERHLETNMCEIFVDKADIDVALDFYKIALTPLRVGLDKPNAKVEID
GKESRKLMAVKDGEARLQRHPKVSEESGTR-
KAGEKAQLPDDALMYDGVQHGDIKFTATDLPDGRV-

VTPVFMSVKAQLEIDKVMLRTFHVVTLSFSNEKAEEHLIKLRLNALKKSTRHYEMLLKIRDTECAQSPHA
IPVGKILKIDALLQTICTAHYTAIEPLLAEDLTALGVMGDQGLLHVVPRLMILAILHLCELEAEAAISRWQ
LQQDAILTDQTMGLAVRERDWSF-TISEVTGNTFFARTKDDDTVERLHARNPI-VIL-YGKEGML-
ISPSGLRLASTAPSDKGHGNDEKLILSPERLQDAMYYYVLRVLLVFVDELILVYGSGESPLKFLVANQAV
TGRDNPQLVCLTRRTFPLDLQRDATFVLVLADTTMKNLSHKKMHMNLVLVKPKSWVENVSTKILISD
QAIASYAEDAGAHTQPSESALAVITSRVIAPIVLVVSQKPAAPSKTLFLEALNLYDDITATVQKRPKRYFI
FEGDVVSVLGTMDAAVILGMQRPQPDRLRRAVTARAIRLAIALRGSGVLASLEGGAINFSRAVDFAT-
TYPGLEA--GLAIV-EALDFRVRNHKTSTKRDRPYKGHTKFLHKAEAEKALVAEGVDTIA
>GCA_000473205
AVIKPVVMKAYAPVLMIRQTQVATVSGAGSPSLRAKTVFRKSGLQRKTTSKWVAKPLYY-
GVVKMGNIVQLVSISVDNRAVCTVAQLVEQEVVVHGLVALEAVKRKIALEEINGMTTEDPARVAEFE
AELDLKRFRDADARSDMLPVLLGLTLEVRFDPHEPVVHDLPLPVIYEVI-
GIMADKGGIEVDDCAVSSAVSDVDPITYVSPIDLEAVAGTEMAFIDQESAVAAARETIALEELALEAGIL
VVGI-PLLLPV-FGEDGDV-MSFAARSRTLKSIVALAVAYPELTRA-IEVAIASGM-
MQAAQAAALAEKRGTAEQIRIVKRMQRSSGLADDAELVILEDAAEGNSSWAGQIYDDPISGSSESDS
AGSIVHAANKRQNFARSITFLRKVEISNKAAAPLMRGVKHNSKSRTARKLDDDIAGLNFPNVPPGIAIR
AAAKKPADRLLHDATEGEEILLVVVRWHVTWKTAVIVLLVLLAGDVVGLIRLGGIAAVGRPDGMLNR
HHKLNGSLIEQRPIIEKMTLRLRAALKEKLGKDQVIIFVVKVHGDLIADATIQMG-
GPVGAVVAIQGELIFVRRHKQTIITDQEATV-VLKGVAEGPADAQALLRIRVTHTLVELSVLLIPE-
PVLVDNVSIREGVGKALQIIQLDVYLLRDMGTGEETLAESRS-------------------------------
----
RSILADVTEPRMENLPEGVCRTFITAGPRAAVGMLFLLLDQAGKLYSIDGDMRELTKSVKSLIGLVDSK
APKVLSAGYAEARLRIRYADCEVLETEADLYPYKEERRQLMRMVPVYIIAEAKRRQLILFALSLKGGHRR
AMMALNVMLAAAIIESQAEQGSVHHQAPQILLDDAEVVGTAIMITRQGALDLKQILKVDVIVLFPEF
QEKLVVSLKFFCTEVAIFVIALITTVGEMLSREPEHAQEEGAGFQKRIRGHSVLFALEVTFDRANCRRDA
LPDVFLGLGLAEPRYGILYTNQPKKKQMPSEPDGDRVDGFELVHAIKEDGRS-EELEMPEAMALAVE-
DATINVSAGVLAMTGGMVAVEKVDSKRKVALAATKWNISKLPLVDVSISGAAPVRSCG-
IEQAGLSAALEAKKVWFLFLPAASNAELEEEKLDGAGSSTAEAAEDKQIDILLVKPVLVVIQDSNSAGLV
ANDGLDEWGTMAYKKHYAFAARMRLHMRTID-------
MNPNKQKSEKVKTVKLEGDTRFRQLAVEGVKEQMP-LHALNPEFAV-
LPVLVTPAVGSVAADENVLGAKVPVDASL-
RELQKSQKGSALPISAIRLLSMRAARGMIEEDIMVAGKVAA-
LFKVRALETIVKLVLRNDSEATLPWRLSVAERLEADTKRASGFAARPFKTKPSLCNTQAHSKIADPIQEIA
AGYVATESKFVFPVEGIDIKLILSNPHSRVGEAKRAGEGHLHAMAKELLAKAVVMLRNEPDTNTEGILN
YLTTNQPTDLLFEDCLDVIIDIRRMSVV---
VHLNTV-
HVVVDGVRPVGDYEIMAAAAESGLIQVEQVDDRTGREKTMMWMYGAMEELWLMSVRWADWVA
DCAIGAIILHVYAQTINRAFSLSRLQLDGWPKEAARWWNTWQLWLNKDGFFGYDAAFRAVKQILKA
TQADFEMKDVEGVGRMQSRQEVRAGYRIREQVEFARLDPRADILLQAKTRLSIIRHALEVVAILILRDV
APEFTLVVADQDVARGHVECVLAESFDFTAKNTHIGPVGLRTPQSILGDGTVLKEAVLGKIDLANTVA
DFRYLFIVDGGRALGVVWLTLENMLVGRIWLPPAQAALKNLMPICFFLSHPELDLGWI--
LFAGISKARAEQLMAGALVEEILEAVVRFAEPEATTHQSKKEKHFKYYGEEVEPVHLAYILAIFALVATQ
VTMSDRLLKDRYVLNLRVFRGFTALLWLSLQLAAKGPVATVITVGTIAGLGALVLFIILL-
RDSVLVETLYYRYVY--

MKGYSAAKDVQDADPEHLGELSAASRDIEWIEIIPAFFYYFKTATSRRTLNTAVAREFFGLFKERLRIIEVL
AVPPDRVAEFVAFVALAGTLDTSATALEGVIAVGLCEPNAAAVLEAERDWNLHRSMGITLAFFEARDS
VKGYFDKGLHKDVAEIGLREAGAFTTHFLCDVDAKQVVPAIHRVLDLGELEAGLIEIDSAVVVADEEQT
LADKPTTRDKSVGAVGGVVLAGTMNMGANLESVERGGNIIIELEEEQYHCVNGKKADGKLTDKVLM
VLSVAYFPVIGRFASANALDRGVFGLLIEAPTDADNQDHGGQMVIPIPSIHSETTPLYAESDIDKIAWIA
PMHLLGTLPAVLLFEPDHYGFISMMIDASLDIMLTAEVLDDYHVLEKGARPPAFRVVLIKGMHIRLLAA
VGTIIVVGSAGRSEVTAYIQDQAVPIILKKQTFLPAFAASSCKVVDIFSTLLRPPERSAVEMLILETVAEL-
LEVTRGENDKLERLLVLLPGLDLASLLALLALADDFSDIVTISAAITAILSVAAEAVILREMLLTKAEIAITAL
ALALALLLMLTGDEGLTQKEIAREAQMKELIQAGAVLQLHADDAMVLASTADKVRLDTDEGTTSAFA
SLNLATVSDGFYVLSSAVLQMSSDLMMIVAVARVLADKESKNALVVRHPEQVKTQPTYAEGSPGMV
LQTLTECIPPLAVKPEDVELLWTLVAALWRVKAHIASAARPHQERVLILLLALGLIVVLLQSEGGGMGS
G-
ASSAAKVLACFSILTLKASVGDEVRKVTRRVMDLSSRLGRPEEAVNPEFKGEELDQTKYEAENVEVDC
METVRRFVDIPFLVSTRTQHNESEDRGARLVLDVTRVRFIDTVVTKRSEVILVERVETDGVIAREMAKD
MQALHETAAALGKVAALQHFIALGDAHISNYVTAQAHSVLGMLLLVLFIQQVRVSKVVWPTRREVT
MVFIMAALAVFFLVIGLN-NLVFMVGGISPLQITTLVLGEGVMAVRQTSTFPAES-
VTILQNTYADLICAGRVTKADIESSLHIVVGARVARGTWYVIYHYEITQAQAPAGIMQRAFRQATLSQ
SLIDGRMEVKIKVVTSDRYA-
TYEIKGGQHRKGTRYNRVKVGHNIAVDLLARIKMAFGPPNLVLVKTFGAVVQKRFAQPLTHIRHGAV
TIGPMEVSSPYITSKKARHAPGDSVVDIKPDNIVRSVAVVPELIWARENEDQSAVHEKYVKTTHKIAAK
RSRAVDKAQYDKRNQVHLSFIGLAVKVLPEQA--
-
RMRPQLNLQGKTDNKVVKCLNNNLDDVEWGKMGRRKIKPITIKSVYDVEHRKATELVKLMPYKDDV
EGRNSQFAEK--
ALFEPLKAFMMMFGFIARSVYALYLRVIRRTIILVKTKIVVNANNVLSAIKIIGVSIRLRHKTTNVAETKHHI
KTIEKRARMTLAGTGAVKLHLSPGRVGKKGGRIKGYMPYKNYNIFDGLASGRRAKVAELAKLKKMDKV
IAIDGIRTRQ-
GGRLPKADLSNALSNTRVDVEVVGENVQEESNPRANAEMGQIFAAIDARVYSLVAAMIGHALEAVL
AVCAILGIGKAHVMLAV-LKMNAGIELHSAA-VARAVAAA-YAQPGGEA-
AIADVSEAYLQALIISEQSLDVCRLRRRQLG--
RHGIKAKGTYGKERAMMQITPTKPVKVWVPVFVNDDELLAPVRAVAETNGDGVTVVKLSNDIKITMF
EKGVASLAISGKVTSLVYTVLEKVTELMKDIIYEQRAKTHYGRGRLRSHVVTHTKQKRHLSMMKRTVV
ADPSMDAARLTDGEVSVVTGATMAFVMSEQDAV---
DVDLHYQEEPRREAPLGIAGPETFLSTEQPRVLALLFLRAYLMHIVDIGTVGGFSDAATIYDILRAMQN
KLDRKLSAEIAKVRMCLHDDVEGMGVSAEAARRDTAGDPILSAYWINGVTANNDIILLVANLECKTVP
ENIDCAVRLFYQRVTLQAKERDGDVSVELVAAQNQIEAAEGTVIYTIKGKTPSTAQMKLNIEAISRSIVK
SLAVQLFQVNLFLNDTWIATLLRAAQNVVFVDAREGKEAVDHVVERDTALEFAPQRDIVARKIRIYTP
AAPSAVVIWDPAIEREDKADAQSTILMIYGPDLQDISCFTDIGAEPKIESLASWDEKEKYVVHRDDIPED
I-
TLVGKSSKVNREFGIPDVQVCAPAKPGVTESIDRGEPKAGSCGEVANLPYPQESLVNNVSSANVQKNI
SMVLGGHPVGEMGIARDVERHLETNMCEIFVDKADVDVALDFYKIALTPLRVGLDKPNAKVEIEGKES
RKLMAVKDGEAALQRHPKVSEESGTRVGGGEKAKLPSDALIFDGVQHGDLKFTVTDLPEGRV-
VTPVFNEVKAQLEIDKVMIKTFHVVTLSFSSEKGDEHLIKLRLNALKKSTRHYEMLLKIRDTECAQSPHAI
PVGKILKIVSLLQTICVAHYTAIEPLMADDLTVLGVMGDQGLLHVVPRLMILAVLHLCPLEAEEMLRSW

PLQQRAILTDQTMGLPLRDRRWSY-AISEVVGNTFFARTKEEDTEQRLHARNPI-MIL-YGKEGML-
ISPSGLKLSSTNPSDADHGNEEQLILSTERLQDKMSYHILAILLVFVDEMILAYGSGDEPLKFLVANQAV
TGRANPQLVCLSRRTFPLDLQRDAVFVLVLADTTMKNLSHKKMHMNLVLVKPKSWVENVSTKILISD
QAIASYAEDAGAHTQPGESALAIITSRVMAPIVLVVSQKPTAPSKTLFLEALNLYDEITATVQKRPKRYF
VFEGDVVSILGTLDAAVILGMQRPQPDRLRRAVVAAAIRLAIALRGSGVLAQLEARMINFARAIDFAQ
-VYEGLEAALASAIS-AALDFRVRNHKVKTKRARPYKGHTKFLHKASAEKGLVAEGVDTIA
>GCA_000473225
AVIKPFLYTKYAPVLMIRQSEVATVSGAGSPSLRAKTVYAKSALSRKTFTKWVAKPIYY-
GVLKMGNIVQLVSISVDARNVCTVAQVVEQEVVVHGLVALEAVKRKIALEEISGMTTEDPAKVAEFEE
EVDLRRYREADSRADVLPVMFGLTLGIRFDPHEPVVHDLPIPVLFEVV-
GIMADKGGIEVDDCAISNAVSDVDPITYVSPIDLEAVAGTEMAFIDQEAAVAAARESISLEELALEAGIL
VVGIKPLLLPV-FAQDADV-MSFANRSRTLGSIVELAVAAEQVSRARID-GIASAM-
MVRVLSAALAEKRGTAEEIRIVKRMQRSAGLADDPDLVMMEDAAEGNSSWAGQIYDDPISGSSESD
SAGNIVHAANKRQNFARSITYLRKVEITDKAAAPLMRGVKHNAKSRTSRKLDDAIAQLNFANVPGGI
AIRAEADKPADTLLHDATQQEEILLLVVRWHVTWKTAVIVALTFTAGDIVGLIELGGIAAVGKPDGLLN
RHHKLNGSLIEQRPIIEKMTLRLRASLKEKLGKDQVIIFVVKVQGDLVADATVQMG-
GTVGAIVAVQGELIFVRRHRQTIVTDQEATV-ILKGVAEGPADAQALLRIRVTHTLVEISVLLIPD-
PVLVESVSIREGVGKTLQIIQLDVYLLRDMGTGEETLAESTS--------------------------------
----
LSMLVAVTEPRMENLPEGVCRRFITAGPRAAVGMLFLLLDDAGRLYIIDGDLRELTKSIKSLIGLVDSTVP
KVLSASYAEDRLKIRFADCEVLQAEADLYAYKEERRELLRMVPVYIINEAKRRQLVILALSLKGGHRRAQ
MALGVLIAAAIIESQAQNDPIHHAIPAILLDDAEVVGTEIMISRQAALDLKQRLKVDVLVIFPEFQEKMV
VSLRFFCTEVAIFVIALIVTVGEMLSREPDHAQEEGAGFQKRIRKHSTLFALSVTFERRNTGDA-
VPDLFLGMGLAPPRYGVIYTSQPKKKQMPSEPDSERVDGFELVQAIKEDTGSNHELDMPEAMALAVE
-DASINVSAGVLAMTGGMVAVEKVDSKRKMALAAT------------------------------------
------
LFLPGASNAELEEVKLDGAGSSTIDAAEDRQTAILLIVPVIVVIQDSNTAGLITNGGLDEWGTMAYKKH
YAFAARMRLHMRTID------EMAPNKQKAEKIKTVKLEGDTRFRQLATEGIKEQMP-
LHALNPDFAV-LPILVTPAVGSVAADENVLGAAVPVDASL-
RELQKSRKGTALPISAVRLLGMHAARAMIEEDIMVAGKVAA-
LLKIRAVETIVDILLRNDSEASLPWRLSVAEKLEADTKRASGFAARPFSSKPSLCNTQAHACIADPIETIA
AGYVAAEQRFVFPVEGIPIQIVLSNPHSRVGTAKRAGEGHLHEMAKELVAKAVLMLRNEPDTNTEGLL
NYLTTNQPTDLLFEDCLDVIIDIRRMSVV--
VHLNTV-
HVVVQGVRPVGDYEIMAANAASGLVQMERVDDRTGRERTIMWMYGAMEELWLMSVTWADWVV
DCAIGAIILHVYAQNLNRAFSLKRLQLDGWAPEANRWWNTWQLWLNKDGFFGYDAAFRAVRQVL
KAALAEFEMRDVKGVGRMLSKQEVRAGYRIRAQVEYARLDPRTDILLQAKTRLSVIRHALQVVAILILR
DVAPEFT-
VVAHQDVARGHVECVLAESFDFTAKNAHIGPVGLRTPETIFGDGTVMQDAVLGKIDLANTIEEFRYR
MLVDGGRQLGVSWLSIQNMLYARIWLPPAQAALKNLLPICFFLSHPELNLEGR-SRFA-
ISKARADQLMAGALIEEILEGVARFAQPEATTHQSHKEKHFKYYGEEVDPVNLAYIFTETALVATQVTIS
DRLLKDRYVLNLRI-RGFTALLWLSVQLAEKGPSATVITVGTIAGLGALVLFIILL-
RDSVLVETLFHRYVY--MLGYSAAKDVADADPEHFVVLAAASRDLEWVEIVPAFFYYFKTAT-
FEPFNTAVAKDFFGLFKERLRLVEVLAVPPDRVETLKEFVALAGTLRTSAQA-

PGIIATKLCEPNAAAVLEAERDWNLHRSMGITLAFFKAQVSVHGWFDKGLHKDVAEIGLREAGAITTH
FLCAVEAQRCVPIVHRVLDLGEVEAGLIEIDSAVLVADEEQNLADKPTTRDNSVNAIGGVVLAGTMN
MGANLDAVERGSNIIIELEEEQYHAVNGANDGAKMTDAVLMVLSMAYFPVIAGFGSANALDRGVFD
LLISAPGDADNLDHGGQMSIPAATIHTETTPLYAEAEIDRIAWIAPMHLLGTLAAVLGFEPDHYSFISM
MIDASLDITETAEVL-
DYHILERGARPPSFRKILIKGLHIRLLAAVGTILVVGSAGRSEITAYIQGRAVPITLNKATFLPAFAPSSCKV
VDIFSTLLRPPERSTVMMLILETVTEL-
LDVTRLENDKLERMAILLPGLDMAQLMDLLAAAQDFADIVTIDAAITAILSVAAEAVVLREMLLTLAEI
AINALALALALLLMLTADNGLTQKERAREAQLKDLIQAGAVLQVHADDAMVLAATADKVRLDTDEG
TTSAFASLNLATVSDGFYVLSSAVLQMSSDLMMIVAVSRVLADKEHKNALVVRHPEQVKTQPTYAEG
SPGMVLQTLTDCIPPLAVKAQAIELLWTLVVALWRVKAHLASAMRPRRERILVLILALGLIVVLLQSEGG
GMGGG-
MGAAAKILAAFSILTLKASAGDEVRKVTRRVMDLNSRLGRPEEAVNPEFRGEELEQTKYEAENVEVDT
VETIRRFVDIPFLVSTRTQHNESEDRGARMVLDVTRVRFIDL-
VTKRSEIILAARAETDGIISRQMAKDLQALHPEAAALGKVSAFEHFIALNDGHISNYVTAEINSVLGMLL
LVLFIQQVRVAKVVWPTRREVTMVFVMAALGLFFLVIGLGVNLVFMVGGISPLQITTLVLGEGVMAVR
QVSTFPAES-
VTILQNTYADLICAGRVTKADIENSLHIVVGARVARGTWYVIYHYEISQAQGPAGIMQRAFRQATLSK
SLIDGRMEVKIKVVTSDRYA-
TYEIKGGQHRKGTRYNRVKVGHNIPVDLLARIKMAFQPPNLVLVKTFGAVVQKRFAQQLTHMRHGA
VTIGPMEVSSPYIRSKKARHAPGDSVVDIKPDNIVRSVAVVPELIWARENEDQSAVHEKYVKVTHKIAA
KRSTAVDKAQYDKRNQVHLSFIGLAVKVLPDKA--
--
RMRPQLNLAGRTDNKIVKCLNNNLDDVEYGKMGRRKIKPLTIKSVYDVESKHATELVKLMPYKDDVE
GRKSQFAEK--
ALFEPLKAFMMVFGFIARSVYALYLRVIRRTILLVKTKVVVNANTVLSAIKIIGVSIRLRHKSTNIAETKHHI
KTIEARARMTLAGTPSVKLHLRDGRIGTKGGRIKGYMPYKNFNIFDKLESGRRAKVKELAKLKKVDKTIA
VDGIRTRQQGGRIPKAQLSNALANTRVGVDIVGENVQQESNPRANAEMGQIFAAIDARVYHLVAAL
IGHALTAVLALCAILGIGRAHTLLAVRLKMNAGIELHSAA-VTEAVAAA-YAQPGGEA-
AVADASEAYLGDLTVTEASLEVCRLRRRQLGE--
RHGIKAKGTFGKERAMMQITPTKPVKVWVPVFVGEDELLAPIRAFAETNGDGVTVVKLIKDVKVTMFE
KAVASLAIAGKFTSIVYTVLEKVTELMKDIIYEQRAKAHYGRGRLRSHVVTHTKSKRHLSMMRRTVVAD
PRMDAARLTNGEVAVITGATMAFVMSEQDAV--
GDVDMHFHEEPRREAQLGIAGPETFLATEQPRLLALLFLREYLIHIVDIGTVGGFADAATIYDILRAMHN
KLDRKLSAEIAKTRMCLSDDVADMGVTDLAARRDSAGDAILSAYWINGVTANADIILLVANMECKTA
PENIDCAVRLFYQRVTLQAKERGGDISVELVPAQNQIEAAEGTVIYTIKGKTPSTAQMKLNIEGISRSIVK
SLAVQLFQVKLFLNDTWIATLLRAANNVVYVDAREGKEAVDHVVERDTDLEFAPQRDIVARKIRIYTP
AAPSAVVIWDPAIEREDKADAQGTILMIYGPDLQDISCFTDIGSEPKIESLKSWDEKEKYVVHRDDIPED
I-
TLVGKSSKVNKEFGIPDVAVCAPAKPGVTESIDRGEPKAGSCGEVANLPYPQVSLVNRVSSANVQKNI
SMVLGGHPVGEMGISRAIERHLETNMCEIFVDMAEVDVALDFYKIALTSLRVGLDPPNAKVQIDGKES
RKLMAILDGGAELQRHPKVSEESGIG-QAGSKAKLPDDTLILDGVQHGDIKFTNTDLPEGRV-
VTPVNNAVKAQLEIDKVLMKTFHVVTLSFSNNKGEEHLIKLRLNALKKSTRHYEMLLKIRDTECADSPH
AIPVGKILKIDALLQTICQAHYTAIEPLLAEDLTALGVMGDQGLLHVVPRLMILAVLHICTLEAQALLPH

WSLQQKALVTDQTMGLPLRDRDWSF-AISEVVGNTFFARTKGDDTEERLHARNPI-IVL-YGKAGIL-
ISPSGLRLDSTRDSPQEHGNEDQLILSTERLQDAMGYFILRVLLVFVDQMILAYGSGDDPLKFLVADQ
AVTGRTNPQLVCLTRRTFPLDLQRDATFVLVLEDSTMKNLSHKKMHMNLVLVKSKGWVENVSTKILI
SDQAIASYAEDAGAHTQPSESALAVITSRVMAPIVLVVSQKPSAPSKTLFLEALNLLDEVTATVLNKPK
RYFIFEGDVISVLASLDAAVLLGMQRPQPDRLRRAVVARAIRLAIAIRGSGVLAALEGRHINFSRAVTCA
R--YPTLEAARSPAIT-HAAEFRVRNHKVSTKRARPYKGHTKFLHKAETEKALIAEGVDTIA
>GCA_000473325
AVIKPVEINKYAPVLMIRQQQVATVSGAGSPSLRAKTVYAKSGLQRKTMTKWVAKPIYF-
GVLKMGNIVQLVSISVDSRAVCTVAQVVEQEVVVHGLVALDAVKRKIALEAMGGMTTEDPAKVAEF
EDEIDLKRFRDADARADMLPVMLGLTLEIKFDPHEPVVHDLAVPVIYEVI-
GIMADKGGIEVDDCAISNAVSDVDPIAYVSPIDLEAVAGTEMAFIDQEAAVAAARETIALEELALEAGIL
VVGIKPLLLPV-FGEEGDV-MSFAARSRTLGSIVALAVAYPELTRA-IDG-IASEM-
MAAAQAAALAEKRGTAEEIRIVKRMQRSSGLADDPELVILEDAAEGNSSWAGQIYDDPVSGSSESDS
AGNIVHAANKRQNFARSITFLRKVEISDKAAAPLMRGVKHNSKSRSARKLDDAIAGLNFPNVPPGIAIR
AEAGKPADRLLHDAAEGELILMIVVRWHVTWKTAVVIALVYQAEDLVGLIELGGIAAVGRPDGILNRH
HKLNGSLIEQRPIVEKLTLRLRAALKEKLGKDQVIIFVVKVQGDLVADATIQMG-
GPVGAVVAIQGELIFVRRHKQTVITDQEATV-VLKGVAEGPADAQALLRIRVTHTLVGLSVLLIPE-
PVLVESVQIREGVGKSLQIIQLDVYLLRDMGTGEETLAESKSRELIREVLAVDPITGVRLAFGKLKERMGK
KLGLKDLETML--
LSILAAVTEPRMENLPEGVCRNFIAAGPAAAVGMLFLLLDDAGKLYQIDGDMRELTKSVKSLIGLVDSK
APKVLSAGYAEERLR--
IIFALSLKGGHRRTMMALGVMLAAAIIESQAEKGEVHHDTPDILLDDAEVVGTSIMLSRQASLDLKQIS
KVDVIVLFPEFQEKMVVSLKFFCTEVAIFVIALITTVGEMLSREPEHAQEEGAGFQKRIRGHSMLFALAV
VFDRRNCRDA-LPDLFLGLGLAEPRYGILYTNQPKKKQMPSEPDGDRVDGFELVHAVKEDGAS-
HELEMPEAMALAVEAGETINLSAGVLAMNGGMVAVEKVDSKRKVALAATKWNISRLKIADVHISGA
APVRSCG-
IEQAGLSAALEGKKVWFLFLPGASNAELEEEKLDGAGASTADAAADKQIHILLVKPVLVVIQDSNSAGL
ISNGGLDEWGTMAYKKHYAFAARMRLHMRTID-------
MAPNKQKSEKVKTVKLEGDTRFRQLATEGVKEQMP-LHALNPDFAV-
LPLLVTPAVGSVAADENVLGADVPVDASL-
RELQKSQKGSALPITAIRLLAMRAARGMIEEDIMVAGKVAA-
LLKIRALETVIQILLRNDSEATLPWRLSVAEKLEADTKRASGFAARPFKTKPSLCNTQAHSKIADPIQEIA
AGYVALEERFVFPVEDIAIRLILANPHSRVEEKKRGGEGHLHGMAKELLAKAVVMLRNEPGTNTEGLLN
YLTTNQPTDLLFEDCLDVIIDIRRMSVV--
VHLNTV-
HVVVAGVRDVGDYEIMSARFPSGLVQVEQVDDRTGREKTLMWMYGAMEELWLMSVRWADWVP
DCEIGAIILHVYAQEINRAFSLKRLQLDGWPKEAARWWNTWQLWLNKDGFFGYEAAFRAVKQVLKA
TLFDFEMSNVEGVGRYQSKQELRVGYRIRQQVEFARLDPRTDILLQAQTRLSIIRHALEVVAILILRDVA
PEFTLVVAYQDVARGHVECVLAESFDFTEKNAHIGPVGLRTPASIFGDGTVLKEAVLGKIDLANTVAEF
RYLVILDGGRALGVEWISL-NMLYARIWLPPAQAALKNLLPICFFLQHPELDLEGL-
MLFDGISKARADKLMAGALVEEILEGVARFADPEATTHQSKKAKHFKYYGEEVDPVHLAYILEVFALVS
TQVVVSDRLLKDRYVLNLKVFRGFTALWWMALQLSPKGPSATVITVGTIAGLGALVLFIILL-
RDSVLVETLYYRYVY--MKGYEAAKDVQDADPEHLGQLAAASRDIEWIEVIPAFFYYFKTAK-
FEPFNTAVAKEFFGLFEERLRIIDVLAVPPDRVAALVEFVALAGTLATSAEALVGVIACGLCEPNAAEVLE

AERDWNLHRSMGITLAFFEARESVKGYFDKGLHKDVAEIGLSEAGALTTHFRCDVDAKQIAPAVHRTL
DLGEIEAALIEIDSAVVVADEEQTLADKPTTRDKSVGAIGGVVLAGTMNMGANLDSVERSGNIIIELEEE
QYHAVNGKKADGKLTDKVLMVLSVTYFPVIRRFASANALDRGVFGLLIDAPEDADNLDHGREMTIPA
PSVLAETTPLYAESEIDKIAWIAPLHLLGTLPAVLGFEPDFQGFISMMIEASLEIMETAEVLDDYHVLERG
SRPPKFRVILIKGLHIRLLAAVGTILVVGSAGRTEITAYIQGQAVPISLSKNTFLPAFSPSSCKAVDIFSTLLR
PPERSAVEMLILETVAEL-
LEVTRGSNDKLERLLVLLPGLDMAELLELLAAAQDFADIVTLGAAITAILSVAAESVVLREMLLTKAEIAIT
ALALAIALLLMLTGDEGLTQKWQARGAQMKELIQAGAVLQIHAADAMVLASTADKVRLDTDEGTTS
AFASLNLATVSDGFYVLSSAVLQMSSDLMMIVSVARVLADKESKNALVVRHPEQVKTQPTYAEGSPG
MVLQTLTGCIPPLAVKPEEVELLWTLVAALWRVKAHLASAARPHNERVLILLLALGLIVVLLQSEGGGM
GGG-
IGAAAKLLAAFSILTMKAAVGDEVRKVTRRVMDLSSRLGRPEEAVNPEFKGEELDQTKYEAENVEVDC
METVRRFVDIPFLVSVRTQHNESEDRGARLVLDVSRVRFIDL-
VTKKSDVILVERVETDGIIAREMAKDMQSLHDEAAALGKVSQLQHFIALGDAHISNYVTAEAHSVLG
MLLLVLFIQQVRVSKVVWPTRREVTMVFIMAALALFFLVIGLGLNIVFMVGGISPLQITTLVLGEGVMA
VRQTSTFPAES-
VTILQNTYADLICAGRVTKADIEKSLHIVVGARVARGTWYVIYHYEITQAQSPAGIMQRAFRQATLSQS
LIDGRMEVKIKVVTSDRYA-
TYEIKGGQHRKGTRYNRVKVGHNIPVDVLARIKMAFSPPNLVLVKTFGAVVQKRFAQPLTHMRHGA
VTIGPMEVSSPYIQSKKARHAPGDSVVDIKPDNIVRSVAVVPELIWARENEDQSAVHEKYVKTTHKIAA
KRNRAVDKAQYDKRNQVHLSFIGLAVKVLPEQAIVHATAGTLLPTESVSVTENLKGVAATIRLDPMEK
ALIAYIKRMRPQLNLQGKTDNKIVKCLNNNLDDVEWGKLGRRKIKPITIKSVYDVEHTRATELVKLMPY
KDDVEGRKSQFAEK--
ALFEPLKAFMMIFGFIARAVYALSLRVVRRTILLVKTKIVVNAETVLSAIKIIGVSIRLRHKSTNIADTKHHIK
TIEARARMTLAGAPAVKLHLRPGRVGKKGGRIKGYMPYKNYNIFDGLASGRRAKVADLAKLKKVDKTI
AVEGIRTRQ-
GGRLPKADLSNALSNTRVDVEVVGENVQKASNPRANAEMGQIFAAIDARVYSLVAALIGHALEALLA
LCVIMGIGRAHALLAVKLKMNAGIGMHSAA-VAAAVAAA-YSQPGGEA-
AIADVSEGDLGALAISESSLDVCRLRRRQIG-
LKKTKKEAYMSLTCFSGKGDKVKIIEATQTQAYEIVATRTAERHGIHAKGTYGKERAMMQITPTKPVKV
WVPVFVSDEELLAPVRAFAETNGDGVDVVKLSNDVKITMFEKGVASLAVSGKNTTVVYTVLEKVTELA
KDIIYEQRAKAHYGRGRLRSHVVTHTKQKRHLSMMKRTVVADPSMDAARLSDGEVSVVTGATMAFV
MSEQDAV--
GDVDYHFHEEPRREAQLGIAGPETFLSTEQPRLLAMLFLREYLIHIVDIGTVGNFADAATIYDILRAMQN
KLDRKLSADIAKVRMCLHDDVENMGVTAEAPRRDAAGDSILSAYWINGVTANSDIILLVANLECKTAP
ENIDCAVRLFYQRITLQAKERDGDVSVKLVPAQNQIEAAEGTVIYTIKGTNPSTAQMKLNVEAISRSIVK
SLALQLFQVDVFLNDTWIATLLRAAQDVVFVDAREGKEAVDHVIERDTELEMAPQKDIVARKIRVYTP
AAPSAVVIWDPAIEREDKADAQGTILMIYGPDLQDISCFTDIGAEPKIESLAAWDEKEKYVVHRDDIPE
DI-
TLVGKSSKVNKEFGIPDVAVCAPAKPGVTESIDRQEPKAGTCGEVANLPYPQESLVNRVSSANVQKNI
SMVLGGHPVGEMGISRGVERHLETNMCEIFVDKPDIDVALDFYKIALTALRVGLDKPNAKVEIDGKES
RKLMAVKDGDASLQRHPKVSEESG--RKAGEKAKLPDDALIYDGVQHGDVKFTATDLPEGRV-
VTPVFMSVKAQLEIDKVMLKTFHVVTLSFSNEKAEEHLIKLRLNALKKSTRHYEMLLKIRDTECAQSPHA
IPVGKILKIDALLQTICKAHYTAIEPLLAEDLTALGVMGDQGLLHVVPRLMII--

LHLCELGAAAAIPQWQLQQAAILTDQTMGLAVRERRWSF-
AISEVTGNTFFARTKDDDTEQRLHARNPI-VIL-YGKEGML-
ISPSGLRLASTAPSDKGHGNDEKLILSPERLQDAMYYYVLKVLLVFVDEIILAYGSGEDPLKFLVAHQAV
TGRENPQLVCLTRRTFPLDLQRDATFVLVLADTTMKNLSHKKMHMNLVLVKPKSWVENVSTKILISD
QAIASYAEDAGAHTQPSESALAVITSRVIAPIVLVVSQKPAAPSKTLFLEALNLYDEITATVQKRPKRYFIF
EGDVVSVLGTMDAAVILGMQRPQPDRLRRAVTARAIRLAIALRGSGLLASLEGGAINFSRAVDFSA-
TYPGLEAA-GLAIV-EALDFRVRNHKTSTKRDRPYKGHTKFLHKAEAEKALIAEGVDTIA
>GCA_000511355
AVIKPVEINKYAPVLMIRQQEVATVSGAGSPALRAKTVYAKSGLQRKTLTKWVAKPIYF-
GVLKMGNIVQLVSISVDSRAVCTVAQVVEQEVVVHGLVALDAVKRKIALEEMDGMTTEDPAKVAEFE
AEIDLKRFRDADARADMLPVMLGLTLEVKFDPHEPVVHDLAIPVIYEVI-
GIMADKGGIEVDDCAISNAVSDVDPLAYVSPIDLEAVAGTEMAFIDQEAAVAAARETIALEELALEAGI
LVVGI-PLLLPV-FGEDGSV-MSFAARSRTLKSIVALAVAAEELTRA-IDG-IASEM-
MAAAQAAALAEKRATAEEIRIVKRMQRSSGLADDPELVILEDAAEGNSSWAGQIYDDPVSGSSESDS
AGNIVHAANKRQNFSRSITYLRKVEISDKAAAPLMRGVKHNSKSRSARKLGDAIAELNFPNVPPGIAIR
AAAGKPGDRLLHEAAEEELLL-
IVVRWHVTWKTAVAIALVYQAENVVGLIALGGIAAVGRPDGILNRHHKLNGSLIEQRPIIEKMTLRLRA
SLKQKLGKDQIIIFVVKVQGDLVADATIQMG-GPVGAVVAIQGELIFVRRHKQTVITDQEATV-
ILKGVAEGPADAQALLRIRVTHTLVGLSVLLIPE-
PVLVEAVQIREGVGKSLQIIQLDVYLLRDMGTGEETLAESKSRELIREVLAVDPITGVRLAFGKLKERAGK
KLGLKDLET----
QSILAAVTEPRMENLPEGVCRDFIAAGPAAAVGMLFLLLDEAGKLYQIDGEMRELTKSVKSLIGLVDSK
APKVLSAGYAEERLRIRFADCEVLGTDANLYPYKEERRELLRMVPVYLINAAKRRQLIIFALSLKGGHRRT
MMALGVMLAAAIIESQAENGAVHHRAPEILLDDAEVVGTAIMVSRQAALDLKQVGKVDVIVIFPEFQ
EKMVVSLKFFCTEVAIFVIALITTVGEMLSREPEHAQEEGAGFQKRIRGHSMLFALAAVFDRRNCRDA-
LPDLFLGLGLAEPRYGILYTNQPKKKQMPSEPDGERVDGFELVHAVKEDATS-EELEMPEAMALAVE-
DATINVSAGVLAMTGGMAAVEKVDSKRKVALAATKWNISKLELVDVHISGAAPVRSCD-
IEQADLSAALGSKKVWFLFLPGASNAELEEAKLDGAGASTADAAADKQIAILLVKPVLVVIQDSNSAGL
ISNGGLEEWGTMAYKKHYSFAARMRLHMRTID-------
MAPNKQKSEKVKTVKLEGDTRFRQLAIEGVKEQMP-LHALNPDFAV-
LPLLVTPAVGSVAADERVLGAKVPVDASL-
RELQKSQKGSALPISAIRLLAMRAARGMIDEDIMVAGKVAA-
LLKIRALETVTAILLRNDSEATLPWRLSVAEKLEADTKRASGFAARPFKTKPSLCNTQAHSKIADPIQEIA
AGYVATEARFVFPVEGVAIKLILSNPHSRVEEKKRGGEGHLHGMAKELLAKAVVMLRNEPGTNTEGLL
NYLTTNQPTDLLFEDCLDVIIDIRRMSVV--
VHINTV-
HVVVAGVRPVGDYEIMSAKFDSGLVQVEQVDDRTGREKTLMWMYGAMEELWLMSVRWADWVP
DCAIGAIILHVYAQEINRAFSLKRLQLDGWPKEAARWWNTWQLWLNKDGFFGYDAAFRAVKQVLK
AALFDFEMSNVEGVGRYQSKQELRVGYRIRQQVEFARLDPRSDILLQAQTRLSIIRHALEVVAILILRDV
APEFT-
VVAYQDVARGHVECVLADSFDFTEKNAHIGPVGLRTPATIFGDGTVLKEAVLGKIDLANTVAEFRYLVL
LDGGRALGVQWISL-NMLYARIWLPPAQAALKNLQPICFFLQHPQLDLAWQ--
LFSGISKTRAERLMAGALVEEILEGVARFADPEATTHQSKKAKHFKYYGEEVDPVHLAYILDLFALVATQ
VVVSDRLLKDRYVLNLKVFRGFTSLLWLALQLSAKGPSATVITVGTIAGLGALVLFIILL-

RDSVLVETLYYRYVYG-MKGYEAAKDVQDADPEHLGQLAAASRDIEWIEIIPAFFYYFKTAT-
FEPFNTAVAREFFGLFKERLRIVEVLAVPPDRVAALVEFVALAGTLATSADT-VGVIACG-
CEPNAAEVLEAERDWNLHRSMGITLAFFEARDSVKGYFDKGLHKDVAEIGLSEAGAFTTHFRCDVDA
KQVAPSVHRTLDLGEIEAGLIEIDSAVVVADEEQTLADKPTTRDKSVGAIGSVVLAGTMNMGANLDSV
ERGGNIIIELEEEQYHGVNGKKADGKLTDKVLMVLSVAYFPVIKRFASANALDHGVFGLLIDAPPDAD
NLDHGR-
MTIPAPTVHAETTPLYAESDIDKIAWIAPLHLLGTLPAVLLFEPDFQGFISMMIDASLDIMETAEVLDDY
HVLEKGARPPKFRVVLIKGLHIRLLAAIGTLLVVGSAGRTEVTAYIQGQAVPISLRKHTFLPAFSPSSCKA
VDIFSTLLRPPERSSVQMLILETVAEL-
LEVTRGTNEKLERLLVLLPGLDLAELLGLLAAAEDFADIVTLGAAITAILSVAAEAVVLREMLLTKAEIAIT
ALALAIALLLMLT-
AEGLTQKWQAREAQMKELIQAGAVLQIHASDAMVLASTADKVRLDTDEGTTSAFASLNLATVSDGF
YVLSSAVLQMSSDLMMIVAVARVLADKESKNALVVRHPEQVKTQPTYAEGSPGMVLQTLTDCIPPLA
VKPEEVELLWTLVAALWRVKAHLASAARPHNERVLILLLALGLIVVLLQSEGGGMGGG-
ASAAARLLAAFSILTLKASVGDEVRKVTRRVMDLSSRLGRPEEAVNPEFKGEELDQTKYEAENVEVDC
METVRRFVDIPFLVSVRTQHNESEDRGARLVLDVSRVRFIDL-
VTKRSEVILVERVETDGIIAREMAKDMQSLHDTAAALGKVSQLQHFIALGDAHISNYVTAEAHSVLGM
LLLVLFIQQVRVSKVVWPTRREVTMVFIMAALAVFFLVIGLGLNIVFMVGGISPLQITTLVLGEGVMAV
RQTSTFPAES-
VTILQNTYADLICAGRVTKADIESSLHIVVGARVARGTWYVIYPYEITQAQAPAGIMQRAFRQATLSQS
LIDGRMEVKIKVVTSDRYA-
TYEIKGGQHRKGTRYNRVKVGHNIPVDVLARIKLAFAPPNLVLVKTFGAVVQKRFAQPLTHMRHGAV
TIGPMEVSSPYIQAKKARHAPGDSVVDIKPDNIVRSVAVVPELIWARENEDQSAVHEKYVKTTHKIAA
KRNRAVDKAQYDKRNQVHLSFIGLAVKVLPEQAIVHSTAGTLLPTESVSVTENLKGVAATIRLDPMEK
ALIAYIKRMRPQLNLQGKTDNKIVKCLNNNLDDVEWGKLGRRKIKPITIKSVYDVEHERATELVKLMPY
KDDVEGRKSQFAEK--
ALFEPLKAFMMLFGFIARAIYALYLRVIRRTIVLVKTKIVVNAETVLSAIKIIGVSIRLRHKSTNIADTKHHIK
TIEARARMTLAGAPAVKLHLSPGRVGKKGGRIKGYMPYKNYNIFDGLASGRRAKVADLAKLKKVDKAI
AVEGIRTRQ-
GGRLPKADLSNALSNTRVDVEVVGENVQEASNPRANAEMGQIFAAIDARVYSLVAALIGHALEAVLA
VCAIMGIGRAHALLAVY-EMNAGIGMHSAA-VAAAVAAA-YSQPAGEA-
AIADVSEGDLGALAISDSSLDVCRLRRRQIG-
LKKTKKEAYMSLTCFSGKGDKVKIIEATQTQAYEIVSTRTAERHGIHAKGTYGKERAMMQITPTKPVKV
WVPVFVTDEELLAPVRALAETSGDGVAVVKLASDIKITMFEKGVASLAVAGKNTTVVYTVLEKVTELAK
DIIYEQRAKAHYGRGRLRSHVVTHTKQKRHLSMMKRTVVADPSMDAARLSDGEVSVVTGATMAFV
MSEQDAV--
GDVDFHFHEEPRREAQLGIAGPETFLSTEQPRLLAMLFLREYLMHIVDIGTVGDFAEAATIYELLRAMQ
AKLDRKLSADIAKVRMCLHDDVEDMGVKAEAPRRDAAGDAILSAFWINGVTANSDIIMLVANLECKT
APENINCAVRLFYQRITLQAKERDGDVSVKLVKAQNQIEAAEGTVIYTIKGKTPSTAQMKLNIESISRSIV
KSLAVQLFQVDVFLNDTWIATLLRAAQDVVFVDAREGKEAVDHVIERDKELEMAPQKDIVARKIRVYT
PAAPSAVVIWDPAIEREDKADAQGTILMIYGPDLQDISCFTDIGAEPKIESLAAWDEKEKYVVHRDDIP
EDITTLVGKSSKVNKEFGIPDVAVCAPAKPGVTESIDRGEPKAGTCGEVANLPYPQESLVNRVSSANV
QKNISMVLGGHPVGEMGISREVERHLETNMCEIFVDKADIDVALDFYKIALTPLRVGLDKPNAKVEID
GKESRKLMAVKDGEARLQRHPKVSAVSGTR-

KAGEKAQLPDDALMYDGVQHGDIKFTATDLPDGRV-
VTPVFMSVKAQLEIDKVMLRTFHVVTLSFSNEKAEEHLIKLRLNALKKSTRHYEMLLKIRDTECAQSPHA
IPVGKILKIDALLQTICQAHYTAIEPLLAEDLTALGVMGDQGLLHVVPRLMILAVLHLCGLEAEAAISRW
QLQQQAILTDQTMGLAVREREWSF-TISEVTGNTFFARTKDDDTVERLHARNPI-VIL-YGKEGML-
ISPSGLRLASTAPSDKGHGNDEKLILSPERLQDSMYYYVLRVLLVFVDELILVYGSGESPLKFLVANQAV
TGRDNPQLVCLTRRTFPLDLQRDATFVLVLADTTMKNLSHKKMHMNLVLVKPKSWVENVSTKILISD
QAIASYAEDAGAHTQPSESALAVITSRVIAPIVLVVSQKPAAPSKTLFLEALNLYDDITATVQKRPKRYFI
FEGDVISVLGAMDAAVILGMQRPQPDRLRRAVTARAIRLAIALRGSGLLASLEGGAINFSRAVDFAA-
TYPGLEA--GLAIA-EALDFRVRNHKTSTKRDRPYKGHTKFLHKAEAEKALVAEGVDTIA
>GCA_000511385
AVIKPVSLNAYAPVLMIRQQEVATVSGAGSPSLRAKTVFRKSGLQRKTMTKWVKSKLYF-
GVLKMGNIVQLVSISVDSRAVCTVAQVVEQEVVVHGLVALEAVKRKIALEEMDGMTTEDPAKVAEFE
AELDLKRFRDADARADMLPVMLGLTLEIRFDPHEPVVHDLPIPVIFEVI-
GIMADKGGIEVDGCAISNAVSDVDPISYVSPIDLAAVAGEQMAFIDQEAAVAAARDAIALEELALEAGI
LVVGISPLLLPV-FGEDGGV-MSFAARSRTLGSIVALAVAYEELTRA-IEA-IASEM-
MQAAQAEALAEKRGTAEEIRIVKRMQRSSGLADDADLVILEDAAEGNSSWAGQIYDDPISGSSESDS
AGNIVHAANKRQNFARSITFLRKAEISDKAAAPLMRGVKHNAKSRTARKLDDAITELNFPNVPPGIAIR
AAAHKPVDRLLHDATEREQILMIVVRWHVTWKTAIAIGLVLTAGDIVGLMELGGIAAVGKPDGALNR
HHKLNGSLIEQRPIIEKMTLRLRASLKEKLGKDQIIIFVVKVQGDLVADATIQMG-
GPVGAVVAVQGEVIFVRRHKQTVITDQDATV-VLKGVAEGPADAQALLRIRVTHTLVELSVLLIPE-
PVLVDSVSIREGVGKALQIIQLDVYLLRDMGTGEETLAESKSRELIREIFALDPITGVRLAYGKLKERMGK
KLALKELD-----
RSILEAVTEPRMENLPAGVCRTFITAGPRAAVGMLFLLLDDGGKLYTIDGDMRELPKSVKSLIGLVDAK
APKVLSAGYAEERLRIRFADCEVLVTEADLYAYKEERRELLRMVPVYIINEAKRRQLIMFALSLKGGHRR
TMMALGVMIAAAIIESQAEKGSVHHKAPQVLLDDAEVVGTAIMISRQSALDLKQMVKVDIIVLFPEFQ
EKMVVSLKFFCTEVAIFVIALITTVGEMLSREPEHAQESGAGFQKRIRGHSVLFALAAVFDRSNLRDT-
TPDVFLSLGLAEPRYGVLYTNQPKKKQMPSEPDGDRVDGFELVHAVKEDGSS-
QELEMPEAMALAVE-
DATINVSAGVLAMNGGMVAVEKVDSKRKVALAATKWSIAKLSIVDITISGAAPIRCCG-
IEMADISAALGKKKV-
FLFLPGASNAELEEEKLDGAGASTADAAEDKQLSILLVKPVLVVIQDSNSAGLISNGGLDEWGTMAYK
KHYSFAARMRLHMRTIDAIEVGVQMEPNKQKSEKVKTVKLEGDTRFRQLAIEGVKEQMP-
LHALNPDFAV-LPLLVTPAVGSVAADERVLGADVPVDASL-
RELQKSQRGSALPITAIRLLEMRAARGMIEEDIMVAGKVAA-
LFKVRALETIVALLLRNDTEATLPWRLSVAERLEADTKRASGFAARPFKSKPSLCKTQAHSKIADPIQEIA
AGYVAAEERFVFPVEDIAIKLILSNPHSRVEEKKRGGEGHLHGMAKELLGKAVVMLRNEPNTNTEGLL
NYLTTNQPTDLLFEDCLDVIIDIRRMSVV--
VHLNTI-
HIVVPGARDVAAFTIMSATFDSGLIQVEQVDDRTGREKTLMWMYGAMEELWLNSVRWADWVPNC
AIGAIILHVYAQTINRAFSLKRLQLDGWPKEAARWWNTWQLWLNKDGFYGYDAAFRA-
KQILKATLADFEMKDVEGVGRMLSKQEVRAGYRIREQVEYARLDPRTDILLQAKTRLSIIRHALTVVAILI
LRDVAPEFTVVVAHEDVARGHVECVLAESFDFTEKDAHIGPVGLRTPAGIFGDGTVFKEAVLGKIDLA
NTVADFRYLILVDGGRALGVCWISLTNLLYTRIWLPPAQADLKDLLPICFFLQHPELDLEMW-
GLFAGISKARADQLMAGALVEEILEGIARFAEPEATTHQSKKAKHFKYYGEEVDPVHLAYILELFALVST

QVTVSDRLLKDRYVLNLKVFRGLTALWWLSLQLAEKGPSATVITVGTIAGLGALVLFIILLMRDSVLVET
LFYRYVY-VMLGYEAAKDVQDADPEHLGPLAAASRDIEWIEIIPAFFYYFKTAT-
FEPFNTAVAREFFGLFKERLRIVDVLAVPPDRVDALVQFVALAGTLATSARA-
VGVIACGLCEPNAAAVLEAERDWNLHRSMGITLAFYDARKSVKGYFDKGLHKDVAEIGLREADALTT
HFHCGAEAKRVAPAVHRVLDLGEIEAGLIEIDSAVVVADEEQTLADKPTTRDKSVGAVGGVVLAGTM
NMGANLDSVERGGNIIIELEEDQYHAVNGKKADGKLTDKVLMVLSAAYFPVVTRFSSANALDRGVFG
LLIEAPTDADNLDHGGSMTIPAPTVLTETTPLYAESDIDRIAWIAPMHMLGTLPAVLTFEPDHYSFISM
MIDASLEIMETAEVLDDYHVLEKGARPPAFRVILIKGLHIRLLAAVGTILVVGSAGRAEVTAYIQGRAVPI
TLRKHTFLPAFSASSCKVVDIFSTLLRPPERSSVEMLILETVAEL-
LEVTRLTNEKLERMMILLPGLDLAALLGLLAAAEDFATIVTLGAAITAILSVAAESVVLREMLLTKAEIAIT
ALAMAMALLLMLTGDEGLTQKWVGREAQMKELIQAGAVLQIHADDAMVLAATADKVRLDTDEGT
TSAFASLNLATVSDGFYVLSSAVLQMSSDLMMIVSVARVLADKESKNALVVRHPEQVKTQPTYAEGS
PGMVLQTLTECIPPLAVKPEEVELLWTLVAALWRVKAHLANAARPHNERVLILLLALGLIVVLLQSEGG
GMGGG-
MGAAAKILAAFSILTLKASVGDEVRKVTRRVMDLSSRLGRPEEAVNPEFKGEELDQTKYEAENVEVDC
METVRRFVDIPFLVSVRTQHNESEDRGARLVLDVTRVRFIDL-
VTKRSDVILVERVETDGIIARQMAKDMQSLHPEAAALGKVSQLQHFIALGDAHISNYVTAQTHSVLG
MLLLVLFIQQVRVSKVVWPTRREVTMVFVMAALAVFFIVIGLGINLVFMVGGISPLQITTLVLGEGVMA
VRQVSTFPAES-
VVILQNTYADLICAGRVTKADIESSLHIVVGARVARGTWYVIYHYEISQAQSPSGILQRAFRQATLSQSL
IDGRMEVKIKVVTSDRYA-
TYEIKGGQHRKGTRYNRVKVGHNIPVELLPRVKMAFGDPNLVLVKTFGAVVQKRFAQQLTHMRHGA
VTIGPMEVSSPYIKSKKARHAPGDSVVDIKPDNVVRSVAVVPELIWARENEDQSAVHEKYVKTTHKIA
AKRNRAVDKAQYDKRNQVHLSFIGLAVKVLPEQAIVHATAGTLLPTESVAVTENLKGVAATIRLDPME
KTLIAYIKRMRPQLNLGGKTDNKVVKCLNNNLDDVEYGKLGRRKIKPITIKSVYDVEHKQATELVKLMP
YKDDVEGRKSQFAEK--
ALFEPLKAFMMIFGFIARAVYALHLRVVRRTILLVKTKIVVNAENVLSAIKIIGVSIRLRHKSTNVADTKHH
IKTIEARARMTLASAPSVKLNLRPGRVGKKGGRIKGYMPYKNYNIFDGLSSGRRAKVKDLAKLKKVDKT
IAVDGIRTRQ-
GGRLPKAQLSNALANTRVGVDIVGENVQEASNPRANAEMGQIFAAIDARVYSLVAALIGHALEAVLA
ICAIMGIGRAHALLAVY-KMNAGIELHSAA-VAEAVAAA-YSQPAGEAAAL-
DVSEYPLRALAVTDQSVDVCRLRRRQLGELKKTKKEAYMSLTCFSGKGDKVKIVEATQTQFYEIVATRT
AERHGISAKGTYGKERAMMQITPTKPVKVWVPVFVNDDELLAPVRAFAETNGDGIDVVKLTTDIKVT
MFEKGVASLAIAGKATTVVYTVLEKVTELSKDIIYEQRAKAHYGRGRLRSHVVTHTKQKRHLSMMKRT
VVADPSMDAARLTDGEVSVVTGATMAFVMSEQDAV--
GDVDMHFHEEPRREAPLGIAGPETFLSTEQPRLLALLFLREYLLHIVDIETVGNFADAATIYDILRAMQN
KLDRKLSADIAKVRMCLSDDVADMGVTDVAARRDSAGDSILSAYWINGVTANSDIILLVANLECKTA
PENIDCAVRLFYQRITLQAKERGGDVSVELVPAKNQIEAAEGTVIYTIKGNNPSSVQMKLNIDAISRSIV
KSLAVQLFQVDSFLNDTWIATLLRAASKVVFVDAREGKEAVDHVVERDTALEMAPQKDIVARKIRIFT
PAAPSAVVIWDPAIEREDKADAQGTILMIYGPDLQDISCFTDIGAEPKIESLAAWDEKEKYVVHRDDIP
EDITTLVGKSSKVNKEFGIPDVAVCAPAKPGVTESIDRGEPKAGSCGEVANLPYPQESVVNRVSSANV
QKNISMVLGGHPVGEMGISRGVERHLETNMCEIFVDKDDIDVALDFYKISLTPLRVGLDKPNAKVDID
GKESRKLMAVKDGDASLQRHPKVSEEAGVR-
KAGEKAKLPDDTLMCDGVQHGDISFTATDLPDGRV-

VTPVFNAVKAQLEIDKVMLKTFHVVTLSFSNDKAEEHLVKLRLNALKKSTRHYEMLLKIRDTECAQSPH
AIPVGKILKIDALLQTICKAHYTAIEPLLAEDLTALGVMGDQGLLHVVPRLMILAVLHLCGITAQMAMA
QWTLQQIAIVTDQTMGLSVRERDWSF-AISEVTGNTFFARTKDDDTEERLHARNPI-VIL-
YGKEGML-
ISPSGLKLSSTNPTDANHGNADQLILSGERLQDTMSYHILQILLVFVDQMILAWGSGDDPLKFLVAEQ
AITGRANPQLVCLTRRTFPLDLQRDAVFVLVLEDTTMKNLSHKKMHMNLVLIKSKGWVENVSTKILIS
DQAIASYAEDAGAHTQPSESALAVITSRVIAPIVLVVSQKPAAPSKTLFLEALNLYDEITATVLKRPKRYFI
FEGDVVSVLDHMDAAVLLGMQRPQPDRLRRAVVARAIRLAIALRGSGILAQLEGGVLNFSRAVNFAE
-TYPLALRAEAA-------EFRVRNHKTSTKRDRPYKGHTKFLHKAEDAKALVADGVDTIA
>GCA_000526275
AVIKPVTMNVYAPVLMIRQDEVATVSGAGSPSLRAKTVYRKSGLQRKMSTKWVASPLYF-
GVVKMGKIVQLVSISVDNRAVCTVAQVVEQEVVVHGIVALEGVKRKIALEAIDGMTAEDPAKVSEFEE
ELDLKKWRDADDRADMLPILLGLTLQIMFDPHQPVVYDLPIPVIFEVV-
GIMAERGGIEVDECGISAAVSDVDPIQYVSPIDLEAVAGTKMEYIDQEAAVHAAKDAIALEELALESVV
LVVGI-PLLLPV-FAEDAEV-MSFANRSRTLESVVDLAVSFPELVRA-I-EGIASAM-
MIRAQAAALAEKRGTAEEIRIVKRMQRAAGLADDPDLVILGDAAEGNAQWAGQIYDDPVSGSSESD
SAGNIVHAANKRQNTARSITFLRKVEISDKAAAPVMRGVKHNSKSRSARKVDDSIAVLNFPNVPEGIA
LRAMAGRPVDTLLHDATEREEILLLIVRWHVTWKTAVILVLVSVAGDIVGLIELGGVGAVGRPDGVLN
RHHKLNGSLIEQRPIVEKMTLRLRAALKQKLGKDQIIIFVVKVQGDLVADATVQMG-
GPLGAVVAIQGDVIFVRRHKQTIVGDQDSTV-ILKGVAEGPADAEALLRIRVDHTLVQLTVLLIPD-
PVLCESVTIREGVGKALQIIQLDVYLLRDMGTGEETLAESKSREVIREVLAIDPITGVTLAYGRLKERMGK
KVGLKDLETL---
LSILAGVTEPRMENLPDGVCRNFIEAGPRAAVGMIFLLLDEAGKLYEIDGDLRELLKSVKSLIGLVDAKA
PKVLSASYATTRLKIRYADCEVLVAEAELYAYKDERRELLRMVPVYIINAAKRRQLIILALSLKGGHRRAL
AALRVMIAAALLESQAEQSTVHHATPAILLEDAEVVGTSIMISRQSALELKSIARTDVVVVFSEFQEKM
VVSLRFFCTEVAIFVIALICTVGDMLSREPDHAQETGAGFQKRIRAHSVLFALDATFERSNLQDA-
TPDLFLGMGLANCRYGILYVHQPKKKQMPTEPDGDRVDGFELVNAIKEDASKNHELEMPEAMALAV
EADATINLSAGVLALKGGMVAVERVESKRKMSLAAEKWNIGKISAVDIHLSGAAMIRGTG-
IELADISAALKTKKVSVLYLLKASNKELEEEKLDGAGSSTANVAEDRQRAILLVHPVLVVIQDSSAAGLV
ANGGVDEWGTMAYKKHYAFAARMRLHMRTIESIEVGVVMEPNKQKAEKVKTVRLEGDTRFRQLAV
EGIKEQMP-LHALNPDFAVLVPILVTPAVGAVAAQAAVLGARVPCDFSL-
RELQKSMRGQALPITAIRILGMRAARAMIEEDIMVAGKVAA-
LLRITALETICALVLRNDSEATLPWRLSVAERLEADTKRASGYAARPFNSKPSLCDTQAHANVADPIETI
AAGYVAKDQRFVFPVEDIAIQIVLSNPHSRVASAKRAGEGHLHEMAKELLAKAVLMLRNEPDTNTEG
LLNYLTTNQPTDLLFEDCLDIIIDLRRMSVV---
IHLNAV-
HVLISGARDIGDRAIMAAAADSGLVQMEQVDDRTGRERTIMWMYGAMEELWLLSVTYADWVPDC
TIGAIILHVYAQQINRAFSLTRLQLEGWPPEAARWWNTWQLWLNKDGFFGYDAAFRAAVQVLKAAL
LDFEMTDVNGVGRHQSKQELRVGYRIREQVAYARLDPRTDILLQAKTRLSVIRHALTVVAILVLREVVG
EFN-
VVAQEDVAMGHVECVLAESFEFTEKDAHIGPVGLRTPETILGDGTVFNEAVLGKVDLANTVADFRYLL
LTDGGRQLGVRWATLRNMLYARIWLPPAAAALKNLLPIRFFLSHPEIHLLLMVALFGGISKARADKLM
AGALIEEILEGVARFAAPEATTHQSYKERHFKYYGEEVDPVHLAYILQVFALVATQVTVSDRLLKDRYVL
QLKVFRGFTSLLWVSLQLAEKGPSATIITVGTVAGLGALVLFIILLMRDSVLVETLYYRYVYVVMLGYEA

AKDVADADPEHFLALSAASRDVEWVEIIPAFFYYFTTAT-
FEPFNTAVAKEFFGLFEERLRVIAVLAVPPDRVLELAEFVALAGTLATSADALPGVIATGLCEPNAAEVLE
ADRDWNLHRSMGITLAFFKAQESVNGWFDKGLHKGVAAVSLSEAGGFTTTNDCAVQAKKCVPAIH
RQVDLGEIEAALIEVESAVLVSEEEQTLADKPTTRDKTVGAIGGVVLAGTMNMGTSLDQLERSSNIIIEL
EEEQYHAVNGKKD-
QKLTDKILMVLSLAYFPVLRRTASASALNSGVFDLLIQAPPDADNSDHGRTLVIPAPTVH-
ETAPLYAEAEIDRIAWIAPLHMLGTLPAVL-
FEPDQYSFISMMIDASLEIMETAEVLDDYHVLERGSRPPSFRVVLIRGLHIRLMAAVGTIIVVGSAGREEI
TAYIQGRAVPIALNKHSFLPAFAASSCEIVDIMSTILRPPERSTVQMLILETVAEL-
LEVTRIGNDKLERLLVLLPGLDMAELLELVAAGADFAEIVTIDAAITAILSVAAESVVLREMLLTLAEIAVT
ALALALALLLMLTADQGLTLKEAAREEQMKELIQGGVVLQVSAADAMVLASTADKVRLDTDEGTTSA
FASLNLATVSDGFYVMSSAVLQMSSDLMMIVEVARVLADKESKNALVVRHPEQVKTEPTYAEGSPG
MVLQTLTQCIPPLAVKPEEVELLWTLVAALWQVKAHLAEARRPKLERILVLLLALSLIIVLLQSEGGGMG
GG-
MGPPPKILAAFSMLTVKASVGDEVRKVTRRVMDLNSRLGRPEEAVNPEFRGEELEQTKFEAENVEVDT
MEDIRRFVDIPFLVSIRTQHNESEDRGARMVLDVTRVRFIDL-
VTKRSEVILVAATETDGIIAREMAKDLQALHPEVAALGKIAALDHFISINDAHISNYVTAAINSVLGMLLL
VLFIQQTRVAKVVWPTRREVTMVFIMSALAVFFLVIGLAVNLVFMLGGISPMQMTTLVLGEGVMAVR
QTSTFPAES-
VTILQNTYADLICAGRVTKADIETSLHIVVAARVARGTWYVIYLYEITQAQGEAGILQRAFRQATLSQSL
IDGRMEVKIKVVTSDRYA-
TYEIKGGQHRKGTRFLRVKIGHNIPVDILARLKLAFGPPNLILVKTFGAVVQKRFAQPLTHIRHGAVTIGP
MEVASPYIQSKKARHAPGASVVEIKPDNIVRSVAVVPELIWAREHEDQSAVHDKYVKTTHKIAAKRNR
AVDKAQYDKRNQVHLSFIGLAVKVLPAQAIVHATAGTLLPTEAVSVTEGLKGVAATIRLDPMEKALIAY
IKRMRPQLNLAGRTDKKIVKCLNNNLDDVEYGKLGRRRIKPLTIKSVYDVEHREATELVKLMPYKDDVE
GRKSQFAEK-
AALFEPLKAFMMVFGFIARAIYALYLRVVRRTILLVKTKIVVNAKSVLSAIKIIGVSIRLRHKSTNIADTNH
HIKTLEKRARMTLAGAPSVKLNLREGRVGKKGGRIKGYMPYKNFNIFEGLSSGRRAGVKDLAKLKKVD
KTIAVDGIRTRQQGGRIPKAQLSNAI-
NTRVGVDIVGENIQEESNPRANAEMGQIFAAIDARVYSLVAALIGHALEAVLAVGTIWGIGRAHALLA
LAFRVNAGIEMHSAA-VRQAVAAA-FAEAAGEA-VIADVSEAYLGDLALTDASIDVCRMRRRQLG-
LKKTKKDAYMKLTCFAGKGDKIKIIEATQTQAYEIVATRTAERFGIKAKGTYGKERAMMQITPTKPVKV
WVPVFVSEDELLAPIRAVAETNGDGVDVVKLTTDIKVTMFEKAVASLAVAGKSTSVVYTVLEKVTELSK
DIIYEQRAKAHYGRGRLRSHVVTHTKQKRHLSMIKRTVVATPRMEAARLTDGEVTVITGATMAFVLSE
QDAV--
GDVDMHFHEEPRREAALGISGPETFLTTEQPRVLALLFLREYLIHIVDIDTCGGFADAATIFDLLRAMQN
KLDRKIAADIAKTRMCLHDNVADMGVRQESPRRDKAGDAILSAYWINGVTANADIIMLVANLECKTA
PENIHCAVRLFYQRVTLQAKERDGDVTVKLVPAKNQIEAAEGTVIYTIKGKTPGTVQMKLNIEAISRSIV
KSLAVQLFQVDLFLNDTWLGVLLRAASDVVFVDAGDGREAVDHVVERDTELELAPQKDIVRRKIRIYT
PAAPSAVVIWDPAIDRADKADAQGTILKVYGPDLQDISCFTDIGSEPKIESLKAWDEKEKYVVHRDDIP
PDI-
TLVGKSSRVNKEFGIPDVGVCAPSKPGVTESIDRGEPKAGSCGEVANLPYPQESVVNRVSSANVQKNI
SMVLGGHPVGEMGISRGVERHLETNMCEILVDKDDVDVALDFYKIALTPLRVGLDKPNAKVDIGGKE
SRKLMAVLDGEADLQRHPKVSEAAGVVRNAGKKAKLPDDALLFDGVHHGDIKFTATDLPEGRV-

VTPVSNNVKAQLKIDKVMLKTFHVVTLSFSSNKGQEHLIKLRLNALKKSTRHYEMMLKIRDTECATSPH
AIPVGKILKIDALLQTICQAHYTAIEPLLADDLTALGVMGDQGLLHIVPRLMILAVLLCWGLAAQAIIPT
WDLQHTALVTDQTMGLPIRDRRWSF-AISEVVGNTFFARTAEDDTEQRLHARNPV-VLL-
YGKFAMV-
ISPSGLKLGSTPSTEEGHGNEERLILSAERLQDAMSYYVLDVLLVFVDQVILAYGSGDTPLKFLVADQAV
TGRDNPQLVCLTR-
TFPLDLQRDATFVLVFEDTTMKNLSHKKMHMNLILIRSKGWIVNVSTKILISDQAIASYAEDAGAHTQP
SESALAVITSRVIAPIVLVLSQKPTAPSKTMFLEALALYDKVTCTILNSPKRYFIFEGDVISILGDIDAAVILG
MQRPQPDRMRRAVVAGAIRLAIAMRASGIVARLDSGAINFSRAVSFAR-AY----------------
DFRVRNHKTSTKRDRPYKGHPKFLHKAEEDKALIAEGVDTLA
>GCA_000768555
AVIKPVVMNEYAPVLMIKQTQVATVSGAGSPSLRAKTVYAKSGLQRKTMTKRVAMNIYY-
GVMKLGDIVQLVSISVDARAVCTVAQVVEQEVVVHGLVALAAVKKKIALEAISGMTTEDPAKVCEFD
DELDLRKFRDAEDRADMLPVLLGLTLAIRFDPHEPLVHDIPIPVIYEVV-
GIMADKGGIEVDDCAISTAVSDVDPLAYVSPIDLAAVAGEQMAFIDQEHAISAAREAIALEELALESAV
LVVGIRPLLIPV-LAEDGAN-MSFANRSRELGSIVDLAVSAERLSRAR-EQGIASEM-
MARMQAEALTERRGTAEEIRIVKRMNRSAGLADDPELVLLGDAAEGNSSWAGQIYDDPISGSSQSDS
AGNIVVAANKRQNFARSITFLRKVEISDQATAPLMRGVKHNSKSRSARKLDDAIAVLNFPNVPEGIAIR
AEGDKPADTLLHDATETEEILLLVVRWKVTWKTAIIIALLVLAGNIVGIIELGGIAAVGRPEGLLNRHHKL
NGSLIEQRPIIEKLTLRLRASLKQKLGKEQVIIFVVKVSGDLVADAKVQM-
GGLIGAVVAVQGDVIFVRRHRQTVVTDSEATVQILKGVAEGPADAEALLRIRVSHTLVALSILLIPQ-
PVLVDSVSIREGVGKALQIIQLDVYLLRDMGTGSETLAESSSRELIREVLMIDPVTGVRLAYGEIKERIGKK
VGLKDLETLF--
ISIMQGITEPRMENLPAGVCRNFIQAGPRAAVGMLFLLLDEAGRVYTIDGDLRELPKSVKSLIGLVDSTA
AKVLSATYGTDRLRIRYADCEVLVAEAILYAYKEERKELLRMVPVYIINEAKRRQLVILALSLRGGHRRA
MASIGVMIAAAILESQADKGVVDHEEPEILLDDAEVVGTAMMISRQAALDLKQIVRTDVVVVFPEFQE
KMVVSLRFFCTEVAIFVIALITTVGEMLSREPNSAKETGAGFQKRIRKHAVMFALEVSFERRNCRDA-
RAELFLGLGLADPRYGIFYINQPKKKQMPSEPDSDRVDGFELVHAVKADTASN-
ELEMPEAMALAVEADAAINLAAGVLAFTG-
MTAIERVDSKRKMALATEKWNISKLEMADIHMSGAGRVRAAG-IEQANLSATLAAKNL--
LYLPGASNSALEEDKLNGAGASSAEAAEERQTRILLVTPVLVVIQDSSAAGLITNGGLEEWGTMAYKK
HYAFAARMRLHMRTVD----GIEMEPSKQKAEKVKTVKLQGDTRFRQLAIDGVKEQMP-
LHALNPDFAVLVPVLVTPAVGRVAADEAVLGARVPCEFSL-
RELQKSMRGAALPITAIRLLPMRAVRAMIEEDIMVAGKVAA-
LLKIRAHETVAGIILRNDSEATLPWRLSVAERLEADAKRMAGYAARPFISKPSLCDVQAHANVAEAIETI
AAGYVAKEERFVYPVEGIKIRLILSSPHSRVGEAKRAGEGHLHEMAKELIAKAVMMLKSEPDTNSEGLL
NYVTTNQPTDLLFPDCLDVVIDMRRMSIV--
VQLTVV-
HVVVEGVRDVGDYEIMAAAAPSGLVQVERVDDRTGRERTIMWMYGAMEELWLMSVRYADWVAD
CAIGAIILHVYAQSINRAFSLTRLQLEGWPQEASRWWNTWQLWLNKDGFFGYEAAFRAVRQALKDA
LFGYEMSDIEGVGRYQSRQELRVGYRIREQVDFARLDPRTDILLQAKTRLAVIRHALALVAILILREVVGE
FTLVVAHEDVAMGHVECVLAESFEFTDKNAHIGPVGLRTPETVLGDGTVLQEAVLGKIDLANTVVDYR
YLFVVDGGRELGVWWLSI-
NMLYTRIWLPPAQATLKNLLPICFFLRHPELNLEEISQAFKRISKARAEQLMAGALIEEILEAVARFADPE

ATTHQSNKERHFKYYGEEVDPVHLAYILELFALVATQVVVSDRLLKDRYVLHMRI-
RGFTSLLWLSLQLAEKGPAATVITLGTIAGLGALVLFIILL-RESVLVETLYYRYVY--
MLGYSAAKDVADADPEHLLVLAAASRDVEYIEIIPAFFYYFDTAT-
FEPFNTAVAKEYFGLFEERLRVIDVLAVPPARVEALVVFVALAGTLPTSAEALPGVIACG-
CEPNAAGVLEADQDWNLHRSMGITLAFFEARESVKNWFDKGLHKDVAETGLREAGGFTTTNLCGKE
AKKCVPAIHRMVDLGELEAGLIEIDSAVLVADEEQTLADRPTTREKKVGAVGGVVLSGTMNMGSALD
QVERGGNIIIELEQEQYHAVNGKKGDGKLTDKVLMVLSCAYFPVLRRMASATALDRGVFDLLISAPED
AENLDHGRWLTIPVPTVHAE-
AILYAESEIDRIAWIAPMHLLGTLPAVLLFEPDQYSFISMMIDASLEITQTAEVLDDYHVLEKGARPPGFR
IILIRGLHIRLLAAIGTIIVVGSAGRAEITAYIQGKAVPISLAKASFLPAFAASSCKLVDIFSTMLRPPERSTV
QTLILETVAEL-
LDVTRGVNDKLERLQLLLPGLDAALLLGLVAKAEDFADIVTISRAITAILSVAAERVVLRELLLELAEIAISS
LALAIALLLMLTPDRGLTQKERAREAQKKELIQGGAVLQIHAADAMVLASTADKVRLNTDEGTTSAFA
SLNLATVSDGFYVMSSAVLQLSSDLMMIVSVARVLADKETKNALVVRHPEQVKTQPTYAEGSPGMV
LQTLTACIPPLAIKPEDVELLWTLVAALWRVKAHLAEARRPRLERVLILLLALALIVVLLQSEGGGMGGG
-
MGAAAKILAAFSILTIKASVGDEVRKVARRVMDLSSRLGRPEESVNPEFRGEELEQTKFEAENVEVDCM
ETIRRFVDIPFLVSIRTQHNESEDRGARLVLDVTRVRFIDL-
VTKRSEIILAARAETDGIIARQMAKDMQSLHPEIAALGKIAALDHFISLNDGHISNYVTADINSVLGMLL
LVLFIQQVRVAKVVWPTRREVTMVFIMATIAIFFLVIGLG-
KLVFMLGGISPLQITTLILGEGVMAVRQASTFPADS-
VTILQNTYADLICAGRVTKADIEASLHIVVGARVARGTWYVIYPYEITQAQGEAGIMQRAFRQATLSKS
LIDGRIEVKVKVVTSDRYA-
TYEIKGGQHRKGTRYNRVKMGHNIPVDLLARIKMAFGDPNLILVKTFGAVVQKRFAAPLTHMRHGAV
TIGPMEVASPYIRMKKARHAPGDSVVEIKPDNIVRSVAVVPELIWARENEDQSAVHDKYVKVTHKIAA
KRNAAVDKAQYDKRNQVHLSFIGLAVKVLPAQAIVDATAGTLLPTASVAVTENLKGVAATIRLDPME
KALIAYIKRMRPQLNLAGKTDNKIVKCLNNNLDDVEYGKLGRRALKPLTIKSVYDVEPRRATELVKLMP
YKDDVEGRKSQFAEK--
ALFEPLKAFMMLFGFIARAVYALYLRVVRRTILLVKTKIVVNAKTVLSAIKIIGVSIRLRHKSTNVADTNHH
IKTIEKRARMTLAGGPSVKLHLHPGKIGMKGGRIKGYMPYKNFNIFEGLASGRRAKVRELAKLKTVDKTI
TVEGIRTRQQGGRIPKADLSNAI-
NTRVGVDIVGENEQEESNPRANAEMGQIFAAIDARVYSLVAALIGHALEAVLALAAILGIGKAHALLA
VRLHVNAGIEFHSAA-VDEAVAAA-YAQPAGEA-AIADVSEGDLGLTRISAASLDVCRMRRRQLG-
LKKTKKDAYMELTCFAGKGDKVKIVEASQSQFYEIVATRTAERFGIKAKGTFGKERAMMQIVPSKPVKV
WVPVFVSEEELLAPIRAFAETNGDGVTVVKLTKDIKVTMFEKAVASLAVTGKSTTVVYTVLEKVTELMK
DIIYEQRAKAHYGRGRLRSHVVTHTKQDRHLSMMRRTVVADPRMNAARLTDGEVSVITGATMAFV
MSEQDAV--
GDVDMHFHEEPRREAELGIAGPETFLATEQPRVLALIFLREYLIHIVEIGTRGGFNDAATIYDILRAMHN
KLDRKLSAEIAKVRMCLHDDVADMGVRQEAPRRETAGDPILSAYWINGVTAQSDIILLVANLECKTAP
ENINCAVRLFYQRVTLQAKERGGDVTVALVKAQNQIEAAEGTVIYEIKGKTPGSIQMKLNVEAISRSIV
KSLAVQLFQVDLFLSDTWIGTLLRAAQDVVYVDAKSGKEAVDHVVERDKAVEMAPQKDIVRRKIRVY
TPAAPSAVVIWDAAIERADKADAQGTIIKIYGPDLQDISCFTDIGSEPKIESIKSWDEKERYVVHRDDIPP
DI-
TLVGKSSKVNKEFGIPDVAVCAPSKPGVVESIDRGEPKAGSCGEVANLPYPQESLVNRVSSANVQKNI

SMVLGGHPVGEMGISRKVERHLETNMCEIFVDKADVDVALDFYTLSLTPLRVGLDQPNAKVEIDGKES
RKLMAVLDGEAALQRHPKVSEASGTGTTAGTKARLPDDALIFDGVLHGDISFTATDLPDGRV-
VTPVANTVKAQLKIDKVLLRTFHVVTLSFSSHKGEENLIKLRLNALKKSTRHYEMLLKIRDTECAQSPHAI
PVGKILKIDALLQTICQAHYVAIEPLLADDLTALGVMGDQGLLHVVPRLMILAVLHLWGLSAADIIPN
WDLPQRALITDLTMGLRVRRREWTY-AISEVVGNTFFARTAEDDTEQRLHARNPI-VLL-YGKFGMV-
ISPSGLRLDSTAPTADEHGNADALILSRERLQDAMGYFILRILLVFVDESILAWGSGDEPLKFLVADQAV
TGRDNPGLVCLTRRTFPLDLQRDATFVMVFEDSTMKNLSHKKMHMNLVLIKSKGWIVNVSTKILISD
QAIASYAEDAGAHSQPSESALAVITSRVMAPIVLVLSQKPTAPSKTMFLEALNLLDDVTATVLNRPKRY
YIFEGDLISVLRTVGASVILGMQRPQPDRMRRAVVTAAIRLAIAALASGVLVALEAGIINFARAISLAR--
YT-V-------------DFRVRNHKTSTKRDKPYKAHPKVLHKADSNKALVAEGVDTIA
>GCA_000836695
AVIKPFLYTAYAPVLMIRQQQVATVSGAGSPSLRAKTVYRKSGLQRKVSTKWVASPLYY-
GVLKMSSLVQLVSISVDNRAVCTVAQVVEQEVVVHGMVALDAVKRKIALQEIDGMTSEDPAKVIDY
AGAIALKRFREADDRADLLPVLLGLTLKLKFDPHQPVVHDLPVPVVFEVV-
GIMAQKGTIEVDDCAISTAVSDVDPVAYVSPIDLAKVSGQKMDYVDQEAAVSAAKEAIALEELALESA
VLVVKI-PLLIPV-FAEEGDR-MSFANRSRNLETIVELAVSAVLITRARIA-DIAAAM-
MARARAAALAERRGTAEEIRIVKRMSRSAGLADDPELAVLEDAAEGNAEWAGLIYDDDLSGS-
LSEAAGNIVFAANQRQATARSITYLRKVEISDKAAAPLMRGVKHNSKSRSSRK------------------
------------------------LVVRWDVTWKTALFILLL-
RAGDIVGVLQLGGLAAVGRMDGVFNRHHKLNGSLIEQKRVIDKLTLRLRAALKQKLGAESVIILVVRVT
GDLVADATVQMG--
DIGSVVAIQAEVIFVRRHRQTVITDQDATVEVLRGVAEGPADAKALLRIRVDHTLVEISVLLIPE-
PVLVESVSIREGVGKALQIIQLDVYLLRDMGTGEDTLAKSMSRELIRDVLMVDPITGVTLAYGRLKERQ
AKAMSLKDLET----
LALVRGVTEPRMENLPAGVCRTFIRAGPRAAVGMLFLMLDPAGRIYTIDGDLRELPKSIKSLIGLVDGAK
PKVLSASYATARLRVRYADVTVLTSEAGLYAYAEERKEIMRYVPVYITVEAKRRQLVILALSLRGGHRRA
GAAIGIMIAAALLESQPVEGEVNHAEPAILLDDAEVVGSDMMISRQAALDLKQLVKMDVVVVFPEFQ
EKMVVSLRFFCTEVAIFVIALITTVGEMISREPSHAQEEGAGYSKRIRGHALLFALAATFDRRNARPA-
APSLFLNLGLAPPRYGVLYKTQPKKKQMPAEPDTDRVDGFDLVHAVKADGRS-
HELDLREAMALAVE-
SQPINLTAGVLAMTGGMAGLEKVDSKRKMAVAATKWTIAPAGVLDLTLSGAQPVRCLG-
IELADVSAALGAKAI-
FLFLPGANNAELEEAKLDGAGASTVDAAEDKQTGILLVSPVVVVIQDSNAAGLIANGGLDEWGTMAY
KKHYAFSARMRLHMRTVDAIQVGVAAEPNKQKAEKIKTVKLEGDTRFRQLAVEGVKEQMP-
LHALNPDFAALVPVLVTPAVGRVAAQTAVLGARVPCPASL-
RELQKSQKGDALPISAVRLLGMGAARAMIDEDVMVAGKVAA-
LLKIGALETIAGIVLLNDSEATLPWRLSVAERLEADTKRASGYAARPFASKPSLCNTQAHARAAEPIQEI
AAGYIAKAERFVFPVEALGIQLFLANLHSRVGGAKRAGEGHLHEMAKELRAKAVLLVRSEPDTNTEGIK
KWMTTGQPTDVLFEDCLDIVIDMRRMNVV--
VQLNLV-
HVVVPGASDIGDRSIMAANADSGLVQTERVDDRTGRERTKMWMYGAMEELWLMSVTWADWIIN
VKIGAIVLHVYAQTINRAFSLHRLQLAGWPPEADRWWNTWQLWLNKDGFFGYQAAFRA-
LQILHEDLYSYELRDVQGVGRMNSRQALRVGYRIREEVEYSRLSPRTDILLQSMTGLAVLRGALAIVAIL
VVREIVAEFSLVVAMEDVAMGHVECVLADMFSFTAKNAHIGPVGLRTPETVLGDGTVFKEALLGKVD

LANTVAEFRYRVLTDGGRALGVAWATLENMLYARIWVPPAPAALKDLLPISFFLGHPEIDL---------
ISKARADRLMAGALVEKILSGVAAFAEPEATTHQSGKEKHFKYYGEEVDPVHLAYILDFFILVATQVTVS
DRLLKDRYALNLRVFRGFTSLLWLALQLGDKGPAATIITVDTIAGLGALVLFIILL-
RDSVLVETLYHRYTY--
HRGYEAAKDVADADPEHFLALAALTRDVEWIEIVPAFFYYFALEDREHAHNTAVAKEFFGLFEERLRIV
DVLAVPPDRVKAIEEFVALAGTLPTAAEALPGVIACGFCEPNAAEVLEASQDWNLHRHLGITLAFFKA
QSSVNGWFDKGLHKDVAETGLREAGAFTTPFLAAVEARQCLPAVHRVVDLGEIEAALLEIDSAVVVA
DEEQTLADKPTTREKSVGAVGGVVLAGTMNMGANLESVERGGNIIIELEEDDYRAVNGASDGSKLTD
KVLMVLSAAYFPVIARAASANSLNRGAVDLLIDAPDDADNLDHGR-
MTIPAPTVHAETAPLYAEAEIERIAWVAPLHLLGTLPAVLEFEPDLYSFMSMMIDASLGITETSEV--
EYHVLETGSRPPEFRIILIRGLHLRQLAGVGLIIVVGSAGRSEVTTYIRD-
AVPISLKRHTFLPAFSPSSCHKVDIFSTLLRPPERSAVQMLILETVAEL-
LDVTRIENDKLERLRLLLPGLDIAALLAFAAAAADFAAIVTIDAAITAILSVAAEAVVLRELLLTLAEIAVTA
LALALALLLMLTPDKGLTLKEHAREAQLKELIQGGVMLQVRAADRMVLAATADKVRLSTDEGTTSAFA
SLNLATVSDGFYVLSSAVLQLSSDLMMIVGVARVLADKETKNALVVRHPEQVKTQPTYAEGSPGMVL
QTLTACIPPLAIKPEDVELLWTLVSALWRAKAHLASAARPRVERILVLILALSLIIVLMQSEGGGIGGG-
MGSAAKILAAFSILTIKAKVGEEVRKVTRRVMDLNSRLGRPEEAVNPEFRGAELEQSKFEAESVEVDTVE
TIRRFVDIPFLVSTRTQHNESEDRGARMVLDVTRTRFIDL-
VTKRSEIILAARVETDGIIAREMQKDLQALHPEAAALGKIAAFRHFIALQDGHISNYVTAAINSVLGMLL
LVLFIQQTRIAKVVWPTRREVTMVFLLAAVAIFFLVIGLGVQIVFMLGGISELQITTLVLGEGVMVVRQT
STFPADS-
VGILQNTYAELIAAGRVAKADIEASLHIVVGARVARGTWYVIYLYEISQAQGEAGIFQRAFRQATLSQS
LIDGRMEVKIKVVTSDRYA-
TYEIKGGQHRKGTRYNRVKVGHNIPVDVLARIKMAFQDPNLVLVKTFGAVVQKRFAAPLTHQRWGA
VSIGPMEVASPYITSKKARHAPGDSVVDLKPDNVVRSVAVVPELIWAREHEDNGAVHDRYTKTTHKL
AAKRRRAVDKAQYDKRNQVELSFMGLAVKVLPEKAIVHASAGTLLPTESVSVTEDLRGVAATIRLDPM
EKALIAYIKRMRPQLNLAGKTDKRIVKCLNNNLDDVEFGKLGRRRIKPLTIKSVYDVEHRRATELVKLMP
YKDDVEGRKSQFAE---------
KAFMMLFGFIARAVYALSLRVVRRTILLAKTRIVVAAATVLSAIKIIGVSIRLRHKTTNVADTRHHIKTIEAR
ARMTLAGAGQSKLNLSEGPIGKKGGRIKGYMPYKNYNIFDGLDSGRRAKVKEVAKLKKVDREIEVGGI
RTRQQGGRKPKAHLSNAMANTRVGVDIVGENVQEESNPRANAEMGQVFAAIDARVYSLVGALIGH
ALEAVLAVAAILGIGRAHALFAVAWRVNAGIELHSAA-
VDAAVAAAAHAEPAGEAATIADVSEAYCGALSVTAASLDVCRLRRRELG-
LKKTKRDAYMKLTCFSGKGERINIVEASQTQAYEIVATRTAERHGIHAKGQYGKERALMQIVPTKPVKV
WVPIFVAEDELLAPIRQFAETNGDGVAVVKLTTDIKVTMFEKAIASLAVAGKSTSVLYTVLEKVTELAKDI
IYEQRAKAHYGRGRLRSHVVTTTQQKRHLSMIRRTVVADPRMDAARLTDGEVSVVTGATMAFVMSE
QDAV--
GDADWHYQEEPRRDAALGIAGPETFLTTEHPRVLALLFLRAYLIHIVEIDTVGQVNDAATIYDILRPLQN
KLDRKLSADIAKTRMCLHDDVPGMGVTEEAARREAAGDPILSAYWINGVPATADIILLVANMETACV
PENIDCAVRLFYQRVTMMAKERGGDVSVAVVKAQNQIEQAEGTVIYTTKGVTPGTLQMKLTVEGVS
RSIVRSLAVQLYQVDTFLNDTWIAVLLRAAMKVVFVEAREGKEAVDHVVERDSTMQADPQKDILPRK
IRIYTPAAPSAVVIWDPAIEREDKADAQGTILKIYGPNLQDIACFTDIGSEPKIESLKAWDEKEKYVVHRD
DIPPDIDTFVGKSSKVNKEFGIPDVAVCAPSKPGVTESIDRGEPKAGSCGEVANMAYPQESLVNAVSS
ANVQKNISMVLGGHPVGEMGISRDVERHLETNMCEIFVDKPDVNVALDFFTIRLT-

LRVGLDKPNARVEIGGKETRKLMAVLDGAAGLQRHPKVSEAAGIT-
AAGARAKMPEDALIHDGVHHGDITFTATDLPEGRV-
VTPVTLKVKQQLKIDKVMLKTFHVVTLSFSSNKGDDHLIKLRLNALKKSTRHYEMLLKIRETECAESPHA
IPVGKILKIDSLLQTICVKFTTPIEPLLSDDLTALGVMGRQGLLHVVPRLMILAVMHLWGLEAALELPRW
PLMRRRCVTDQTMGLATRERHFTY-AISEVVGNTFFARTADDDTQQRLHARNPI-ILL-FSKFGMV-
ISPSGLRLDSTAVTEMERGNKSDLILSRERLQDEMGYYVVRVLLVFVDEVILGYGSGEDPLKFLVAEQA
VTGRANPGMVTLTRRTFPLDLQRDRTFVLVFEDSTMKNLSHKKMHMNLVLIKNKGWIVNVSTKILISD
QAIASYAEDAGAHTQPSESALAVITSRVIAPIVLVVKQKPTAPSKSLFLEALALYDQVTATVQNPPKRYY
IFEGDVTSVLGAVDAAVLLGMQRPQPDRLRRAIVARAIRLAIALRASGILAELEAGLINFARAVSFAA-
CYPAPEAI----------EFRVRAHKVSTKRSRPYKEHTKFLHKAEEDKSLIAAGVDTLA
>GCA_000967725
AVVKPVTMNTYAPVLMIRQQQVATVSGAGSPSLRAKTVYRKSGLQRKTETKWVSKDFYY-
GVVKLGNIVQLVSISVDNRAVCTVAQLVDQEVVVRGMVALEAVKKKIALEAIEGMTAEDPAKVVDY-
GEIELKKFRDADDRADLLPVMIGLTLKIKFDPHEPVVHDLAVPVVFEVV-
GIMVQKGTMEVDDCGVSTALSDVDPIIYVSPIDLEAVAGDAMAYIDQEAAVAAAREDIALEELALESG
VLVVGI-PLLIPV-FEADGDT-MSFANRSRTLKTIVNLAVSYPEIKRA-ID-NIAAAM-
MEASQAAALAEKKGTAEEIRIVKRMQRSAGLADDPDLVMLEDSAEGNSNWAGQIYDDPISGSSESD
SAGNIVHAANKRQNFARSITYLRAVEISDQAAAPLMRGVKHNAKSRAARKISDEIAVLNFPNVPEGIAI
KAAMGKFEDTLLHDAAEREQILL--
LNRHHKLNGSLIEQRRIVEKLTLRLRAALKQKLGKDQIIIFVVKVAGDLVAAATVQM---
SVGAVVAIQGPTIFVRRSRQTVIGDQEATVEVLRGVAEGPADAEALLRIRVDHTVVKLSVLLIPAVPVLV
ESVTIREGVGKALQIIQLDVYLLRDMGTGEETLAKSKSREQFREVLQVDPITGVRLAFGHLRERLVKGGT
MKELGTL---
ISILKNVTEPRMENLPEGVCRSFVNAGPHAAVGMLFLLLNDAGKIYTIDGDLRENTKSIKSLIGLVDASK
PKILSAAYATARLKIRYADVHVLTSDADLYAYAEERRELLRMVPVYIINAAKRRQLIIFALSLKGGHRRAR
AALGVMIAAAILESQAEQGAVNHEEPAVLLDDGEIVGSEMMISRQAALEVKQTFKIDVIVIFPEFQEKM
VVSLKFFCTEVAIFVIALICTVGEMLSREPKHAQEGGAGFQKRIRGHSVLFALSVTFDRRNLRD--A-
GLFLGLGLAPPRYGIFYTEQPKRKQMPSEADADEIDGFDLVHAIKEDATS-EQLDMPEAMALAVE-
DASINVNGGVLALSGEMADVEKVDSKRKMALAVEKWTISPIPIVDIKISGANPFRALG-
IEQADVSASLPKKAVEELFLPGANNAELEEEKLDGAGSSTAEAAEDKQLGILLVNPVLVVIQDSNSAALI
ANGGLEEWGTMAYKKHYALAARMRLHMRTID-------
MDPNKQKAEKIKTVKLDGDTRFRQLATDGVKEQMP-
LHALNPDFAVLVPVLVTPAVGRVAADADVLGADVPCDFSL-
RELQKSQKGSALPISGIRVLGMRTARAMVDQDIMVAGKVAA-
LLAIVALETITAIVLMNDTEATLPWRLSVAERLEADTKRSGGFASRPFKSKPSLCKTQAHAKIADPIQEIA
AGYVATEERFVFPVEGVDIQLILANPHSRVGTAKRAGLGHLHEMAKELQAKAVIMVQNEPETNTDGL
LNYLTTQQPTDLLFEDCLDVVIDMRRMSVV--
VQLNTI-
HFVVPGARDVAAFEIMAAAAHSGLVQVERVDERTGRERTLMWMYGAMEELWLMSVKYADWVIDC
AIGAILLHVYAQKINRAFSLERLQLAGWPPEATRWWNTWQLWLNKDGFFSYEAAFRAVKAVLKSDL
DDYDMSDVEGVGRYQSRQELRVGYRIREQVEFEQLDPRTDILLQSLTRLSMIRHALNMVAILIVREIVV
EFT-
VVAQEDVAMGHVECVLADAFDFTAKNADIGPVGLRTPEPVLGDGTVLKEAVLGKIDLANTVADFRYK
MLIDGGRELGVSWLNI-NMLYARIWVPPAQADLKDLLPICFFLQHPQIDLLLD-

ALFAGISKARANKLMAGAMIEKILEKVAAFADPEATTHQSKKLRHFKYYGEEVDPVYLAYILAMFALVS
TQVTVSDRLLKDRYVLNLKVFRGFTSLWWLSAQIGDKGPAATIITVQTIAGLGALVVFIILLMRAVVLVE
TLYYKYVY--
MLGYQAAKDVADADPEHFLVLAHASRDVEWIEVVPAFFYYFKTATSMNNINTALAKEFFGLFKERLRV
VDVLVVPPDRVEALVDFVALAGTLTTSARALVGVIACGFCEPNAAAVLEADQDWNLHRSMGITLAFY
KAQVSVKKWFDKGLHKDVAEIKLNEAGALTTPFHAEVTAKKCVPAVHRVLDLGEVEAALIEIDSAVVV
ADEEQTLADKPTTRENSVGAVGGIVLAGTMNMGANLDSVERAGNIIIELEEENYAAVNGKKADGKLT
DKVLMVLSQAYFPVLKRMASANALDRGAFGLLIDAPDDADNKDHGGVLTIPAPTIHTESVPLFTETDI
ERIAWIAPLHLLGTLEAVLAFEPDQYSFISMMVEASLDIMQTAEVLDDYHVLEKGSRPPSFRVILIKGLHI
RLLAGICTIIVVGSAGRTEVTAYIQT-
AVPITLNKSTFLPAFAASSCHAIDIFSTMLRPPERSTIEMLILETVAEL-
LEVTRIENDKLERMDALLPGLDVAALLQLLAAAKDFADIVTIDAAITAILSVAAEAVVLREMLLTMAEIA
VTALALAMALLLMLTRDQGLHQRETAREDQLKELIQGGTVLQVKAADRMMLASTADKVRLDTDEGT
TSAFASLNLATVSDGFYVMSSSVLQMSSDLMMIVAVARVLADKETKNALVVRHPEQVKTQSTYAEG
SPGMVLQTLTQAIPPLAIKPEDVELLWTLVAALWRVKAHLANAGRPHHERVLILILALSLITVLLQSEGG
GMGGA--
GPAAKILAAFSILTIKAKAGEEVRKVTRRVMDLNSRLGRPEEAVNPEFRGAELEQTKYEAESVEVDTMET
IRRFVDIPFLVSVRTQHNESEDRGARMVLDVTRVRFIDL-
VTKRSEIILAARVETDGIIAREMGKDLAALQPEAAALGKIAALQHFIAIQDAHISNYVTAEIHAVLGMLLL
VLFFQEVRISKVTWPTRREVTMVFILAALALFFLVIGLWVQIVFMLSGISWNQLTTIVLGEGIMVVRQTS
TFPADS-
IGILQNTYADLICAGRVAKADIEKSLHIVVAGRVARGTWYVIYLYDISQAQGQSGIMQRAFRQLTLSQS
LIDGRMEVKIKVVTSDRYA-
TYEIKGGQHRKGTRYNRVKVGHNIPVQVLPRMKMAFSPPNLVLVKTFGAVVQKRFAQPLTHMRHG
AVTIGPMEVSSPYIANKKARHAPGDSVVDLKPDNIVRSVAVVPELIWARENEDNGAVHDKYVKTTHK
LAAKRKKAVDKAQYDHRNQVELSFIGLAVKVLPEKAIVHATSGELMPTASVAVTEGMKGIAATIRLDP
MEKALIAYIKRMRPQLNLAGQTDKKIVKCLNNNLDDVEYGKLGRRKIKPITIKSVYDVEHRRATELVKL
MPYKDDVETRKSQFAEK--
ALFEPLKAFMMLFGFIARSVYALHLRVIRRTILLVKTKIVVNAATVLSAIKIIGVSIRLRHKSTNVADTKHHI
KTIEARARMTLAGAGKVKLNLREGKIGMKGGRIKGFMPYKNFNIFDGLASGRRAKVKELAKLKDVDKTI
SVDGVRVKQQGGRTPKAQLSNAI-
NTRVGVDIVGENEQEESNPRANAEMGQIFAKIDACVYSHVGALVGHALEAVLAVVVIWAIGRAHNLL
AVRLKVNAGIEMHSAA-VEAAVAAA-FAQPAGEA-
AIADVSEGDLGALALTEMSVDVCRLRRRSLNELKKTKKDAYMSLTCFSGKGDKVKIVEASQTQAYEIVA
TRTAERHGIKAKGTYGKERAMMQILPTKPVKVWVPVFVNEDELLAPIRAFAETQGDGVTVVKLTTDVK
VTMFEKAVASLAVTGKATTVVYTVLEKVTQLMKDIIYTQRAKAHYGRGRLRSHVVTTTKQRRHLSMM
KRTVIADPSMKASRLTDGEVTVITGATMAFVMSEQDAV--
GDVDLHFHEEPRREAELGIGGPETFLTTEQPRVLVLLYLRAYLIHIVEIETTNGFADAATIYDVLRAMQN
KLDRKLSADIAKTRMVLTDNVTDMGVTDVAPRRNAAGDKILSAFWINGVPANADIILLVANMECPCV
PENIDCAIRLFYQRITLQAKERGGDVSVAVVPAANQIEAAEGTVIYSIKGQTPGTVQMKLTVEGISRSV
VKSFAVQLFQVDTFLNDTWIATCLRAANQVVFVSSEDPKEAVDHVVERDTELEFAPQKDIVARKIRIYT
PAAPSAVVIWDPAIDREDKAEAQGTILKIYGPNLQDINCFTDIGSEPKIESLKAWDEKEKYVVHRDDIPE
DITTLVGKSSKVNKEFGIPDVAVCAPSKPGVTESIDRGEPKAGTCGEVANMAYPQESLVNTVSSANVQ
KNISMVLTGHPVGEMGISREIERHLETNMCEIFVDKEDVDVALDFHILQLTPLRVGLDKPNARVQIEGK

ESRKLMAVQDGDAALQRHPKVSEATGGQ-KAGTKARMPDDALILDGVMHGDISFTNTDLPEGRV-
VTPVFNAVKAQLKIDKVILRGFHVVTLSFSNEKGEDHLIKLRLNALKKNTRHYEMLLKIRETECAESPHAI
SVGKVLKIDKLLQTICEAHYKAIEPLLADDLTALGVLGQQGLLHVVPRLMILAVMHLVRLELALQISYW
GLQADAIVTDQTLGLTMRERLWSY-AISEVVGNTFFARTKEDDTEQRVHARNPV-VLL-LGKFEMI-
ISPSGLGLKSTEVTPQDRGNAEELILSTERLQDEMGYYVLRILLVFVDMVILAYGSGDDPIKFLVADQAV
TGRQNPGMVTLTRRTFPLELQRDATFVLVLEDSTMKNLSHKKMHMNLVIIKSKGWLVNVSTKILISDQ
AIASYAEDAGAHTQPSESALAVITSRVMAPIVLVVSQKPTAPSKTLFLEALSLYDEVTATVLNKPKKYYIF
EGDVISVLGTLDASVILGMQRPQPDRMRRAVVAQAIRLAIAIMASKVFTRLEQGLINFARAVTFAALTY
ELDDAQL---------EFRVRNHDVSTKRSRPYKAHTKFLHKADVDKSLVATGVDTLA
>GCA_001046955
AVIKPVSLNTYAPVLMIRQTQVATVSGAGSPSLRAKTVYRKSGLSRKTSTKWVAKPLYY-
GVLKLGDIVQLVSISVDNRSVCTVAQLVEQEVVVHGLVALDAVKRKIALEAIDGMTTEDPARVAEFEA
ELDLQKFRDAQEHADLLPVMLGLTLKIKFDPHEPVVHDLPIPVIFEVI-
GIMAERGGIEVDECAISNAVSDVDPITYVSPIDLDAVTGEQMAFIDQEAAVNAARTAIELEELALEAGIL
VVGIDPLLIPV-FADDAAV-MSFANRSRELGSIVDLAVAYPEITRARI-AGIASAL-
MVRAQAEALAEKRGTAEEIRIVKRMSRSSGLADNPDLMVLEDAAEGNSAWAGQIYDDPISGSSESDS
AGNIVHAANKRQNFARSITYLRQVEISDKAKAPLMRGVKHNSKSRSARNVDDAIAVLNFPNVPHGV
AIRAEQDKPAATLLHDATEAEAILLLIVRWKVTWKTAVFLLLVF-
AGDIVGLVQLGGLAAVGRPDGILNRHHKLNGSLIEQKRVVEKLTLRIRAALKEKLGADQVIILVVKVSG
DLVADAKVQMG-
GTLGSVVAIQGDVIFVRRHKQTIVTDQESTVEILRGVAEGPADAAALLRIRVDHTLVKLTVLLIPE-
PVLVESVTIREGVGKALQIIQLDVYLLRDMGTGDETLAESQSRELIRELLMVDPITGVRLAYGQVKERLN
KQLTIKDLD-----RSIL-
DITEPRMENLPEGVCRTFIQAGPEAAVGQLFLLLAEAGKLYELDGDLRELPKTVKALIGLVDAKAPKVLS
ASYATARLKLRYADCTVLEAEADLYAYAEERRELLRMVPVYIINAAKRRQLVIFMLSLRGGHRRALAAIG
VMLAAAILESHADQGKVHHAAPEILLDSAEIVGTAMMISRQASLDLKARSKTDVIVVFPEFQEKMVVS
LRFFCTDVAIFVIALIVTVGEMLSREKSHAQEAGAGYQKRIRGHSMLFALAVVFDRRNLRDA-
TGDLFLALGLAAPRYGILYTNQPKKKQMPSEPDSDRVDGFELVHAIKEDERKNHELEMPEAMALAVE-
SETINLSAGVLALSGEMAAVERVDSKRKMSKAVEKWNISRLQVRDVALSGAQPVRATG-
IEPFAVLAALGAKNV-
FLYLLGASNEELEESRLDGAGSSTANVAEDRQRQILLVHPVLVVIQDSGAASLVSNGGVDEWGTMAY
KKHYAYAARMRLHMRTIDAIEVGVEMAPNKQKSEKVKTVKLEGDTRFRQLAVEGIKEQMP-
IHALNPDFAVLVQLLVTPAVGRVAADAGVLGAEVPCDFSL-
RELQKSMKGAALPISAIRLLAMRAARAMIEEDIMVAGKIAA-
LLKISALETILAVILRNDSEAWGPWRLSVAERLEADTKRASGFAARPFKSKPSLCETQAHAKVADVIQEI
AAGYVAAEQKFVFPVEGIAIQLILSSPHSRVGEAKRAAEGHLHDMAKELVAKAVLMLQNEPDTNTEG
LLNYITTNQPTDLLFPDCLDVIIELRRMSVV--
IHLNTV-
HVVVRGVRDVGDFQIMDANAPSGLVQVERIDERTGMEKTIMWMYGAMEELWLLSITYADWVTDC
AIGAIVLHVYAQSINRAFSLKRLQLNGWPKEAARWWNTWQLWLNKDGFFGYEAAFRAAAQVLKAA
LYDYDMSDVEGVGRYQSRQQLRVGYRIREQVEFARLDPRTDILLQARTRLSVIRHALTVVAILVLREVV
GEFT-
VVAEEDVAMGHVECVLADCFDFTRKNAHIGPVGLRTPETVLGDGTVFKEALLGKVDWANTVADFRY
LVIVDGGRRLGVTWVAL-NILYPRIWLPPAEAALKNLLPICFFLSHPELDL---------

ISKARADKLMAGALVEEILEGVARFADPEATTHQTNKERHFKYYGEEVDPVYLAYILEIFALVATQVTVS
DRLLKDRYVLNLKV-RGVTALLLLALQLAEKGPAATVITVGTIAGLGALVLFIILL-
RDEVLVETLYHRYVYV-MRGYSAAKDVADADPEHFLRLAAVSRDVEWIEIIPAFFYYFKTAG-
FQPFNTAVAREFFGLFAERLRVVDVLAVPPDRVAALVEFVALAGTLPTSAQALPGIIATD-
CEPNAAAVLEAEQDWNLHRSMGITLAFFEARESVKGWFDKGLHKEVAETGLSEAGGFTTHNDCEVE
AKKCLPAIHRVLDLGEIEAALIEIDSAVVVADEEQTLADTPTTRDKAVNAIGGVVLAGTMNMGANLDS
VERGGNIIIELEEDQYHAVNGKKTDGKLTDKVLMVLSRAYFVVLRALASANALDRGVINLLIDAPQEAD
NLDHGG-MVIPAPSVLGETTPLYAESEIDRIAYIAPIHLLGTLAAVL-
FEPDQFSFISMMIEASLEIMETAEV--
DYHVLEKGGRPPAFRVILIKGLHIRLMAAIGTIIVVGSAGRAEVTAYIQGRAVPITLNKNTFLPAFAASSC
KAVDIFSTMLRPPERSTVQMLILETVAEL-
LEVTRLENEKLERIEMLLPGLDMAELLDLAAAATDFANIVTIGAAITAILSVAAEAVVLRELLLTKAEIAITA
LALAIALLLMLTGDRGLTQKESARDGQMKELIQGGAVLQIRADDRMVLAATADKVRLDTDEGTTSAF
ASLNLATVSDGFYVLSSAVLQMSADLMMIVTVARVLADKEAKNALVVRHPEQVKTQPTYAEGSPGM
VLQTLTGCIPPLAIKPEEVELLWTLVTALWRVKAHLAQARRPLRERVLILLLALALIVVLMQSEGGGMG
GG-
VGSAAKVLAAFSILTIKAAVGDEVRKVTRRVMDLNSRLGRPEETVSPEFRGEELEQSKFEAENVEVDTM
ETVRRFVDIPFLVSVRTQHNESEDRGARLVLDVTRVRFIDL-
VTKKSDVILVERVETDGIIAREMAKDLQALQTEAAALGKIAALDHFIALNDGHISNYVTEKIHSVLGMLL
LVLFIQQVRIAKVVWPTRREVTMVFIMATLAIFFIVIGLAVNLVFMVGGISPLQITTLVLGEGVMAVRQT
STFPAES-
VTILQNTYADLICAGRVTKADIEASLHIVVGARVARGTWYVIHHYEISQAQGEAGIMQRAFRQATLSQ
SLIDGRMEVKVKVVTSDRYA-
TYEIKGGQHRKGTRFHRVKMGHNIPVEILARIKLAFGDPNLILVKTFGAVVQKRFAQPLTHIRHGAVTIG
PMEVASPYITMKKARHAPGDSVVDVKPDNIVRSVAVVPELIWARENEDNSAVHDKYVKTTHKTAAK
RNRAVDKAQYDKRSQVHLSFIGLAVKVLPEQAIVHATAGTLLPTESVAVTENLKGVAATIRLDPMEKA
LIAYIKRMRPQLNLGGKTDKKIVKCLNNGLDDVEYGKLGRRKIKPITIKSVYDVEHREATELVKVMPYKD
DVESRKSQFAEK--
AIFEPLKAFMMLFGFIARAVYALYLRVVRRTILLAKTKIVVNANTVLSAIKIIGVSIRLRHKSTNIADTKHHI
KTIEARARMTLAGTPSVKLNLREGKVGMKAGRIKGYMPYKNFNIFEGLASGRRAKVKDLAKLKKVDKTI
SVDGIRTRQ-
GGRIPKAQLSNALANTRVGVDIVGENMQEESNPRANAEMGQVFAAIDARVYSLVAALRGHALAAVL
AVCAIQGIGRAHSLLAVY-KINAGIEFHSAA-VETAVAAA-
YAQPGGEAAALWDVSEADLGTLLLTEASLDVVRRPRRSLGELKKTKKEAYMSLTCFGGKGDKVKIVEA
TQTQAYEIVATRTAERFGIAAKGTYGKERAMMQITPTKPVKVWVPVFVSEDELLAPIRAFAETNGDGV
TVVKLTKDIKVTMFEKAVASLAVAGKSTSVVYTVLEKVTELSKDIIYDQRAKAHYGRGRLRSHVVTHTK
QKRHLSMIKRTVVASPKMDAARLHDGEVTVITGATMAFVMSEQDAV--
GDVDMHFHEEPRREAALGIAGPETFLATEQPRVLALLLLREYLIHIVDIETVGGFADAATIFDILRAMQN
KLDRKLSAEIAKVRMCLHDDVADMGVKAEAPRREAAGDPILSAYWINGVTASSDIILLVANLECHTAP
ENIDCAVRLFYQRITLQAKERGGDVSVALVPAQNQIEAAEGTVIYTIKGKTPSTVQMKLNIEAISNSIVK
SLAVQLFQVELFLNDTWIAVLLRAAEEVVFVDSGEGREAVDHVVERDTELEMAPQKDIVRRKIRIYTPA
APSAVVIWDPAIERADKADAQGTILKIYGPDLQDIACFTDIGAEPKIESLKAWDEKERYVVHRDDIPPDI
TTLVGKSSRVNKEFGIPDVAVCAPSKPGV-
ESIDRGEPKAGSCGEVANLPYPQDSLVNRVSSANVQKNISMVLGGHPVGEMGISRGVERHLETNMC

QIFVDKDDVDVALDFYKLALTPLRVGLDSPNAKVQIDGKESRKLMAVLDGEAKLQRHPKVSTAAGG-
RKAGMKARLPDDALIFDGVHHGDIKFTATDLPEGRV-
VTPVFNNVKAQLEIDKVILKTFHVVTLSFSNDKGEEHLIKLRLNALKKSTRHYEMLLKIRDTECATSPHAI
PVGKILKIDSLLQTICQAHYKAIEPLLAEDLTALGVLGDQGLLHIVPRLMILAVLTLWGLEAALEHRRW-
LQATRLVTDQTMGLAVRERHWSF-AISEVVGNTFFARTKDDDTAEQLHARNPI-VIL-YGKEGMV-
ISPSGLKLASTKPSATGHGNENRLVLSTERLQDSMSYFVLKVLLVFVDEMLVAWGSGEPPLKFLVAEQ
AITGRANPQLVCLTRRTFPLDLQRHAVFVMVFEDSTMKNLSHKKMHMNLVLIKSKGWVVNVSTKILI
SDQAIASYAEDAGAHTQPSESALAIITSRVIAPIVLVISQKPAAPSKTMFLEALNLLDEITVTVQNKPKRY
YIFEGDVVAVLGGLDAAVLLGMQRPQPARMRRAVVAAAIRLAIALRGSAVVAQLEGGVLNFARAVSL
SA-VYPDLDF-TGAAIE-RAIAFRVRNHKTSTKRDRPYKGHPKVLHKAEAEKSLVADGVDTLA
>GCA_001399515
AVIKPVTLPVYAPVLMIRQQQVATVSGAGSPSLRAKTVYAKSGLQRRTVTKWVLSPFYY-
GVIKMGKLVQLVSISVDHRAVCTVAQLVEQEVVVRGMVALDVVKKKIALQEIDGMTSEDPAKVVDYE
LTLETKRLRDSDDRADMLPVLLGLTLAIKFDPHEPVVHDLAVPVIFEVI-
GIMAQKGTIEVDDCAISTAVSDVDPINYVSPIDLAAVAGEDMAFITQDAAIDAAVAKIALEELVLESDV
LIVGISSLLVPV-FDADGDT-MSFANRSRTLESIVDLAVAYPQLTRAR----IASAW-
MRAAQAAALSEKRGTAEEIRIVKRMSRSAGLADDPDLLILEDAAEGNSSWAGQIYDDDISGSSQSDS
AGNIVHAANARQNFARSITYLRKVELSDQAEAPLMRGVKHNSKSRAARKLNDAIAELNFPNVPEGIAI
RAEGGKADVTLLHDAAEGEEILM-IVRWHVTWRTAIIIALVG-
AGDVVGLVPLGGAASIGRRDGMLNRHHKMNGSLIEQKRVIDKLTLRLRAALKQKLGKDNVIIFVVKV
QGDLVAAATIQVG--
DIGAVVAIQGETIFVRRHKQTVIKDEEATVEVLKGVAEGPADAEALLRIRVSHTVVDISVLLIPQ-
PVLVESVAIREGVGKALQIIQLDVYLLRDMGTGEPTLAVSKSRELIRSVLQVDPITGVRLAYGGLKERMG
HGMTLKDLRTLFN-
AAIHQNVTEPRMRNLADGVHRNMVEAGPRAATGTLFLILDDAGWLEGLDGDLRELTKGVKALIGLL
DSAKPKVLSAGYAAALLKLRFQDCTVLTAEANLYAYREERRELLRMVPVYIILEAKRRQLVILALSLRGG
HLRARMAIGVMIAAAIIESQALQGDVHHAEPRVMLDDAEIVGSSMMISRQSALDLKQILKMDIIVIFPE
FQEKMVVSLRFFCTEVAIFVIALITTVGQMLSRDPAHAQEDGAGYSRRIRAHSLLFALAVTLDRRNMG
VDALADLFLGLGLAEPRYGILYTRAPKKKQMPSEPDGDRVDGFELVHAVKADKAS-
TVLELREAMALAVEATVTINLSAGVLALNGEMTAVELVQSNRKLSLAVEKWTISAEDILDVTFSGAARV
RALGNIELADISAALAQKAID-
LYLPGATNAELEEETLAGAGVSAIEAAADRQIAILVVRPVVVVIQDSNAAGLIANGGVLEWGTMAYKK
HYAYAARARLHMRTID-------LEPNKQKAEKIKTVKLGGDTRFRQLAVAGVKEQMP-
LHALNPDFAVLVPVLVTPAVGLVAADEALLGGRVPCPASL-
RELQKSQNADALPISAIRVLGMRAARAMVEEDVMIAGKLPAELLVILALETIIAIVLLNDSEATLPWRLS
VAERLEADTKRDGGYAARPFTSKPSLCNTRAHAKIAENIQEIAAGYVAKAEKFVYPVEGIPIQLILASPHS
RVNTNKRAGLGHLHQMAKELQAKAVVLVQNAPDTNTQGLLNYMTTQQPSDLLFSDCLDIVIDMRR
MSIV--IHLNTV-
HPVVEGVRDVGRYQIMNAAFPSALVQVERVDDRTGRERTRMWMYGAMEELWLMSIRYADWVVN
TAIGAIILHVYAQTINRAFSFMRLQIAGWPKEAVRWWNTWQLWLNKDGFFGYTAAFRAVRQVLKAD
LYDYQLKDVEGVGRMQSRQQLRVGYRIREQVAFSRLDPRTDILLQSLSRLSTLRHALKIVSLLVMRQFV
IEFTLVVAQEDVAMGHVECVLADSFAFTAKNADVGPVGLRTIETVLGDGTIFQDALLGKVDFSSTVAD
FRYLFIVDGGRALGVKWVTMQNLLYARIWLPPAAATLKNLQPICFFLGHPTLDLLLD-
ALFAGISKARADRLMAGAMIEKIIEKVVAFADPEATTHQSRKLKHFKYYGEEVDPVFLAYILAMFALVST

QVTVSDRLLKDRYVLQLKIFRGFTALWWLGAQLGAKGPAATIISVGSVAGLGALVLFIILL-
RVSVLVETLYYRYVY--MKGYQAAKDAADADPEHFLALAEVSRDVEWIEIVPSFFYYFKTAT-
FEPFNTALAKEFFGLFPERLRVVDVLAVPPDRVESLKDFVALAGTLATSADTLVGVIACDFCGPNAAGV
LEADQDWNLHRSMGITLAFYDARESIKGYFDKGLHKGVATVQLEEAGALTSPFQAAVTAKRCAPAVH
RVLDMGELEAALIQVDSAVVVADEEQTLADQPTTRDRAVGVVGGVILAGTMNMGAALDQLERGSN
IIIELEEEGYHAVNGKKSDGKLTDKVLMVLSAAYFPVLRRLASANSLEKGVFHLLIPAPDDDENSDHGR
AMSVPAPTIYAETAPLYAEAEIDSIAWIAPLHMLGTLPAVLLFEPDQYSFISMMIDASLNIVENAEVL-
DYHVLEKGGRPPDFRIILIRGLHVRQMGAISTIIVVGSAGRTEITAYIHG-
AVPIGLRRNTFLHAFAATSCFVVDIFSTMLRPPERSTILMLILETVAEL-
VDVTRWEADKLERLLLLLPGLDVAELLVLLAAARDFAQIVTIDAAITAILSVPAEPVVLRDLLLTLAEIAIS
ALAMGIALLLVLTPAEGLHQREHAREAQGKALIQSGAVLQVHAADAMVLAATADKVRLATDEGATS
AFASLNLATVSDGFYVLSSSVLQMGPDLMMIVKVARVLADKETKNALVVRHPEQVKTQPTYAEGSPG
MVLQTLTGSIPPLSIKPEQIELLWTLVHALWRVKAHLAEAARPITERVIVLILALVLIVVLLQSEGGGMGA
G-
LSSGAKWLAAFSLLTLKAKVGDEVRKVTRRVMDLSSRLGRPEDTVNPEFRGAELEQQKYEAANVEVD
TMETIRRFVDIPFLVSIRTQHNESEDRGSRMVLDVSRTRFIDVIVTKRSEIILAARVETDGIIAREMSKDLQ
ALHPQHAALGKIAALQHYISLNDGHISNYVTAEIHSVLGMLLLVLFAQQVRIGKITWPTRREVTMVVAI
AALALFFAVILLVIQIVFMLSGISWQQITTLVLGEGIVLVRQTSTFPVDA-
IGILQNTYADLVAAGRVAKADIEASLHIVVGARVARGTWYVIYLYEITQAQGESGILQRAFRQATLSQR
LIDGRLEVKIKVVTSDRYA-
THEIKGGQHRKGTRYNRVKVGHNIPVAVLARIKLAYSDPNLVLVKTMGAVVQKRFAQQLTHIRHGAI
TIGPMEVSSPYIKAKKARHAPGDSVVDAKPDNIVRSVAVVPELIWARENEDNNAVHDKYTKTTHKLA
AKRRKAVDKAQYDRRNQVQLSFIGLAVKVLPEKAIVQATAGTLLPTSSVSVTEGIKGIAATIRLDPMEKT
LIAYIKRMRPQLNLGGKTDKKIVKCLNNNLDDVEFGKLGRRKIKPITIRSIYDVEHKEAVELVKLMPYKD
DVEGRKSQFAEK--
AIFEPLKAFMMMFGFIARAIYALSLRVVRRTILLVKCRIVVNADTVLSAIKVIGVSIRLRHKSTNVAETDH
HIKTIEKRARLTLAGTTNVKLHLHEGRVGKKGGRIKGFMPYKNYNIFDGLASGRRAKVQEVAKLKKVDK
EIVVDGIRVKQQGGRQSKADLSNAIANTRVDVD-
VGETTQEGSNPRSNAEMGRVFAAIDARVYALVGAITGHALAAVLAIVRIWFIGRTHCLLAVAFRVNAG
IELHSAA-VEQAVAAA-
AARAAGEATAMWDISEYPGVALLIGEASTTVCRATRRRINELKKTKKEAYLALSCFAGKGEKVVVIDAS
QTQAYEVVTTRTAERHGIHAKGTFAKERAIMQITPTKPVKIWVPIFVSETELLAPVRAFAETNGDGVTV
VKLIKDIKITVFEKAIASLAVTGKSTSVVYTVLEKVTELMKDIIYEQRAKAHYGRGRLRSHVVTHTKSKRHL
SMIRRTVVADPSMNAARLTDGEVSVITGATMAFVMSEQDAV--
GDVDMHYHEEPRREVALGISGPETFLATAQPRVLALIILREYLLHIVEVDTTGNVADAATIYDVLRAMQ
SKLDRKLSADIAKVRMVLVDDVPEMGVTDVAARREKAGDAILSAYWINGVPAVADIILLVANLEVAAV
PETVDFAIRLFYPRITLQARERGGDVSVAVVKAQNQIEAAEGTVIYTTKGVKPGTLQFKLIIEGISKSIVKS
LAVQLFQVDLFLNDTWIATLLRAALEVVFMDASDPKEAVDHVVDRDHAVEMAPQKHIVARKIRIYTP
RAPSAVVIWDPAIDRSDKADAQGTILKIFGPNLQDISCFTDIGAEPKIESIKSWDEKEKYVVHRDDIPADI
-TLVGKSSRVDKEFGIPDVAVCAPAKPGVTESIDRGEP-
VGSCGEVANLAYPQEGLVNGVSSANVQKNISMVLGGHPVGEMGISRSVERHLETSMCEIFVDKPDV
DVALDFFRLILT-
LRVGLDKPNRRVAMDGRETRKVMAVLDGDAALQRHPKLSEATGITQGAGSKARLPDDSLILDGVLH
GDVPFTITDLPEGRV-

VTPVVLAVKAQLSIDKVMVKTFHVVTLSFSNNKGEDHLIKLKLNALKKSTRHYEMLLKIRETECAESPHA
IPVGRILKIDALLQTICKAHYTAIEPLLADDLTALGVMGDQGLLHLVPRLMILAVALLA-
IDAEMFIAPWALPRIALVNEQTMGLRLRARDWTY-AISEVVGNTFFARTAADDTEQRVFARNPI-
MLL-YSRAGMV-
ISPSGLRLASTAATDLQEGCA_AAVILSAESLQDAMEYYVLQILLVFVDQVILAYGSGSSPIRFLVADQA
VTGRGNPGMVTLTRRTFPFELQRDAVFVLVVADSTMKNLSHRKMHMNLILVKNKGWIVNVSTKILIS
DQAISSYAEDAGAHTQPSESALAIITSRVIAPIVIVVSQKPTAPSKSLFLEALSLYDKVTSTIRNAPKKYFIF
EGDLISVLGPIDAAVLKGLQRPQPDRMRRAVIAIAIRLAIAYAASDIWVQLAHHVVNFARAISFVALCY
DEP-------------AFRVRAHKVSTKRSKPYKEHTKFLHKAEVEKALVAAGVDTLA
>GCA_001411945
AVIKPVTMDAYAPVLMIRQQQVATVSGAGSPTLRAKTVYAKSGLQRKSETKWVSKDFYY-
GVVKLGNIVQLVSISVDKRAVCTVAQLVDQEVVVRGMVALEAVKKKIALEAIQGMTAEDPAKVVDYA
SEIELRKFRDADDRADMLPVLLGLTLKIKFDPHQPVVHDLPIPVVFEVV-
GIMVQRGQIEVDECAISTAISDVDPIVYVSPIDLAAVAGDKMAYIDQEAAVANAKQEIDLEELALESSV
LVVGI-PLLIPV-FAADGDV-MSFANRSRTLKTIVNLAVSYPAIKRA-----IAAAM-
MDASQAAALAEKRGTAEEIRIVKRMQRSSGLADDPELLMLEDAAEGNSNWAGQIYDDPISGSSESDS
AGNIVYAANKRQNFARSITYLRAVEISDPAAAPLMRGVKHNSKSRAARKLDDTIAALNFPNVPEGIAIR
AEAGKPRDLLLHDATEGEEILLIIVRWHVTWKTAVIISLFPLADNVVGIITMGGIGAVGRKDGLLNRHHK
LNGSLIEQRRIVEKLTLRLRAALKQKLGAEQIIIFVVKVAGDLVAKAKVQM---
SVGTVVAIQGETIFVRRHRQTVIADQEATV-ILKGVAEGPADAQALLRIRVDHTVVKLSVLLIPD-
PVLTESVSIREGVGKALQIIQLDVYLLRDMGTGEETLAKSRSREVIREVLQVDDITGVRLAFGQLRERLVK
GGTMKELG-----
IAILKDITEPRMANLPAGVCRTFVNAGPRAAVGMIFLLLDEAGKIYTIDGDLRENTKSIKSMIGLVDAAK
PKTLSAGYATTRLKIRYADVTVLTSDADLYAYAEERKELLRMVPVYITNQAKRRQLIIFALSLKGGHRRA
RAALGVMIAAAILESQAEQGAVNHAEPAVLLDDGEIVGSEIMISRQAALDLKQIIKTDIIVIFPEFQEKM
VVSLKFFCTDVAIFVIALICTVGEMLSREPSHAQEEGAGFQKRIRAHSVLFALAVTFDRRNLRTD-
TPTLFLNLGLAPPRYGVFYTEQPKRKQMPSEADDDEVDGFDLVNAIKEDGTK-
EELDMPEAMALAVE-
DGTINVNAGVLAMSGDMAAVEKVDSKRKMALAVDKWTIARVDIVDLKISGAAPFRALN-
IEQADVSATLSNKQV-
FLFLPGANNAEMEEAKLDGAGSSTAEAAADKQLGILLINPVLVVIQDSNTAGLIANGGLEEWGTMAY
KKHYALAARMRLHMRTID----GVEMDPNKQKAEKIKTVKLEGDTRFRQLATEGVKEQMP-
LHALNPDFAVLVPVLVTPAVGRVAADADVLGARVPCDASL-
RELQKSMKGNALPISGIRLLGMRAARAMVDEDIMVAGKVAA-
LLGIVAVTTITHIVLRNDSEATLPWRLSVAERIEADTKRASGFAARPFKSKPSLCKTQAHAQIADPIQEIA
AGYVATEGRFVFPVEGIPIQLILASPHSRIGAAKRAGEGHLHEMAKELQAKAVIMVLNEPDTNTEGLLN
YLTTQQPTDLLFEDCLDVVIDMRRMSVV--
VQLNTV-
HVLVPGVRELADREIMAAAAHSGLVQIERVDERTGRERTLMWMYGAMEELWLMSIRYADWVVDVA
IGAILLHVYAQQINRAFSLERLQLAGWPMEATRWWNTWQLWLNKDGFFSYEAAFRAVKAVLKSDLD
DFDMSDVEGVGRYQSRQQLRVGYRIRQQVAFEQLDPRTDILLQSMTRLSVIRHALTMVAILIVREVVV
EFTLVVAKEDVAMGHVECVLADSFDFTAKDADIGPVGIRTPDPVLGDGTVLKEAVLGKIDLANTVADF
RYKMLVDGGRELGVSWVTLQNLIYKRIWVPPAQAALKNLVPIAFFLGHTELNLHIFPALWAGISKARA
NKLMAGALIEKILEKVAAFADPEATTHQSKKIRHFKYYGEEVDPVYLAYILDMFALVATQVTVSDRLLK

DRYVLNLKIFRGFTSLLWLSAQIGDKGPAATVITVQTIMGLGALVLFIILL-RVVVLVETLYYKYVY--
MLGYQAAKDVADADPEHFLSLAHASRDVEWIEVIPAFFYYFKTAGSRLSKNTALAKEFFGLFKERLRVI
DVLAVPPDRVEALVAFVALAGTLGTSAQALPGIIACGFCEPNAAAVLEADQDWNLHRSMGITLAFYD
ARESVKGWFDKGLHKDVAATGLKEAGALTTPFHAGATAKQCVPAIHRVLDLGEVEAALIEIDSAVVVA
DEEQTLADKPTTRENSVGAVGGVVLAGTMNMGANLDSVERGGNIIIELEEEQYSAVNGKKSDGKVT
DKVVMVLSAAYFPVLKRMASANALDKGAFGLLIDAPDDADNKDHGRELTIPAPTVHAESAPLYAESEI
ERIAWIAPLHLLGTLDAVLGFEPDQYSFISMMIDASLEIMETAEVLDDYHVLETGSRPPGFRVILIKGLHI
RLLAGVCTIIVVGSAGRTEVTAYIQG-
AVPITLNKKTFLPAFAASSCHAIDIFSTMLRPPERSTIQMLILETVAEL-
LEVTRIENDKLERMFALLPGLDVASLLDLVAAATDFAAIVTIDQAITAILSVAAEAVVLREMLLTLAEIAV
TALALALALLLMLTPDQGLHQKEQAREAQMKALIQGGVVLQVKAADRMMLASTADKVRLDTDEGT
TSAFASLNLATVSDGFYVLSSAVLQMTADLMMIVAVARVLADKETKNALVVRHPEQVKTQSTYAEGS
PGMVLQTLTGAIPPLAIKPETVELLWTLVAALWRVKAHLANAARPHNERVLILILALSLITVLLQSEGGG
MGGG--
GPAAKALAAFSILTIKAKAGDEVRKVARRVMDLNSRLGRPEEAVNPEFRGAELEQSKYEAENVEVDTM
ETIRRFVDIPFLVSIRTQHNESEDRGARMVLDVTRVRFIDL-
VTKRSEIILAARAETDGIIAREMGKDLAALHPEAAALGKIAALQHFIAVQDAHISNYVTAEIHAVLGMILL
VLFIQEVRVGKVVWPTRREVTMVFIMAALAIFFLVIGLGVQIVFMLSGISWNQLTTIVLGEGIMVVRQT
STFPEGS-
IGILQNTYADLICSGRVAKADIEASLHIVVAGRVARGTWYVIYLYEISQAQDQSGIMQRAFRQMTLSQ
SLIDGRMEVKIKVVTSDRYA-
TYEIKGGQHRKGTRYNRVKVGHNIPVQVLPRMKMAFGPPNLVLVKTFGAVVQKRFAQALTHIRHGA
VTIGPMEVSSPYISNKKARHAPGDSVVDLKPDNIVRSVAVVPELIWARENEDNGAVHDKYVKTTHKL
AAKRKAAVDKAQYDHRNQVELSFIGLAVKVLPEKAIVHATSGQLMPTASVAVTDKMKGIAATIRLDP
MEKALIAYIKRMRPQLNLGGQTDKKIVKCLNNNLDDVEYGKLGRRKIKPITIKSVYDVEHRRATELVKL
MPYKDDVESRKSQFAEK--
ALFEPLKAFMMLFGYIARAVYALYLRVIRRTILLVKTKIVVNASTVLSAIKIIGVSIRLRHKSTNVAETNHHI
KTIEARARMTLAGAGKVKLNLSPGKIGKKGGRIKGYMPYKNFNIFDGLASGRRAKVKELAKLKEVDKTIS
VDGVRQKQQGGRTPKAQLSNAI-
NTRVGVDIVGENEQEESNPRANAEMGQIFAKIDARVFSHVGALIGHALEAVLAVVSIWDIGRAHNLL
AVRLKINAGIEMHSAA-
VDTAVAAAAFAQSAGEASVIADISESYLGALAISAMSVDVCRLRRRGLSELKKTKKDAYMSLTCFGGK
GDKVKIVEATQTQAYEIVATRTAERHGIRAKGTYGKERAMMQILPTKPVKIWVPVFVTEVELLAPIRAF
AESVGDGVTVVKLTKDIKVTMFEKAVASIAVTGKSTSVVYTVLEKVTELMKDIIYTQRAKAHYGRGRLR
SHVVTTTKQSRHLSMIKRTVVADPSMDASRLTDGEVTVITGATMAFVMSEQEAV--
GDVDLHFHEEPRREAEVGIGGPETFLTTEQPRVLALLAVKAYLIHIVEIGTVNGFSDAATIYDVLRAMQ
NKLDRKLSADIAKTRMVLTDNVTDMGVTEVAPRREAAGDKILSAFWINGVTANADIILLVANLEVQC
APENIDCAIRLFYQRITLQAKERGGDVSVAIVPAANQIEAAENTVIYTIKGKTPGTGQMKLTIEGISRSIV
KSLAVQLFQVDTFLDDTWIATWLRAALQVVFVGAEDGKEAVDHVVERDTELEFAPQKDIVSRKIRIYT
PAAPSAVVIWDPAIEREDKAEAQGTILKIYGPNLQDINCFTDIGAEPKIESLKAWDEKEKYVVHRDDIPE
DI-
TLVGKSSKVNKEFAIPDVAVCAPSKPGVTESIDRGEPKAGTCGEVANMAYPQESLVNAVSSANVQKN
ISMVLSGHPVGEMGISREIERHLETSMCEIFVDKDDVDVALDFYILQLTPLRVGLDPPNARVQIDGKES
RKLMAIQDGDAALQRHPKVSQATGTGLKAGIKARMPDDALMVDGVMHGDISFTATDLPDGRV-

VTPVFNNVKAQLKIDKVLLKAFHVVTLSFSNDKGEEHLIKLRLNALKKSTRHYEMLLKIRETECAESPHAI
SVGKVLKIDALMQTICQAHYKEIEPLLADELTALGVMGEQGLLHIVPRLMILAVLQLFRLEIALQIDNW
GLQAEALVTDQTMGLYLRDREWSF-AISEVVGNTFFARTKETDTEQKIRARNPV-VLL-LGKFEMI-
ISPSGLGLASTAESPQERGNAEELILSTERLQDEMGYYVLQILLVFVDEVILAYGSGEGPIKFLVAEQAVT
GRQNPGMVTLTRRTFPLELQRNATFVMVFEDSTMKNLSHKKMHMNLVVIKSKGWIVNVSTKILISDQ
AIASYAEDAGAHTQPSESALAVITSRVMAPIVLVVSQKPTAPSKTLFLEALSLYDEVTSTVQNKPKKYYIF
EGDVVAVLGTLDAAVLLGMQRPQPDRMRRAIVADAVRLAIALRASGTITRLEENLINFSRAVNFAALF
Y-SNGA-----------EFRVRNHDVSTKRSRPYKAHPKFLHKADVDKGLIATGVDTLA
>GCA_001419985
AVIKPVTMDDYAPVLMIRQQQVATVSGAGSPTLRAKTVYAKSGLQRKSETKWVSKDFYY-
GVVKLGNIVQLVSISVDKRAVCTVAQLVDQEVVVRGMVALDAVKKKIALEAIQGMTAEDPAKVVDY
ASEIELRKFRDADDRADMLPVLLGLTLKIKFDPHQPVVHDLPIPVVFEVV-
GIMVQRGQIEVDECAISTAVSDVDPIVYVSPIDLAAVAGDKMAYIDQEAAVANAKQEIDLEELALESSV
LVVGI-PLLIPV-FAADGDV-MSFANRSRTLKTIVNLAVSYPAIKRA-----IAAAM-
MDASQAAALAEKRGTAEEIRIVKRMQRSSGLADDPELVMLEDAAEGNSNWAGQIYDDPISGSSESD
SAGNIVYAANKRQNFARSITYLRAVEISDPAAAPLMRGVKHNSKSRAARKLDDTIAALNFPNVPEGIAI
RAEAGKPRDLLLHDATEGEEILLIIVRWHVTWKTAVIISLFPLADNVVGIITMGGIGAVGRKDGLLNRHH
KLNGSLIEQRRIVEKLTLRLRAALKQKLGAEQIIIFVVKVAGDLVAKAKVQM---
SVGTVVAIQGETIFVRRHRQTVIADQEATV-ILKGVAEGPADAQALLRIRVDHTVVKLSVLLIPD-
PVLTESVSIREGVGKALQIIQLDVYLLRDMGTGEETLAKSRSREVIREVLQVDDITGVRLAFGQLRERLVK
GGTMKELG-----
IAILKNITEPRMANLPAGVCRTFVNAGPRAAVGMIFLLLDEAGKIYTIDGDLRENTKSIKSMIGLVDAAK
PKTLSAGYATARLKIRYADVTVLTSDADLYAYAEERKELLRMVPVYITNQAKRRQLIIFALSLKGGHRRA
RAALGVMIAAAILESQAEQGTVNHAEPAVLLDDGEIVGSEIMISRQAALDLKQIIKTDIIVIFPEFQEKMV
VSLKFFCTDVAIFVIALICTVGEMLSREPSHAQEEGAGFQKRIRAHSVLFALAVTFDRRNLRTD-
TPTLFLNLGLAPPRYGVFYTEQPKRKQMPSEADDDEVDGFDLVNAIKEDGTK-
EELDMPEAMALAVE-
DGTINVNAGVLAITGDMAAVEKVDSKRKMALAVEKWTIARVDIADLKISGAAPFRALN-
IEQADVSATLSNKQVWFLFLPGANNAEMEEAKLDGAGSSTAEAAADKQLGILLINPVLVVIQDSNTA
GLIANGGLEEWGTMAYKKHYALAARMRLHMRTID----
GVEMDPNKQKAEKIKTVKLEGDTRFRQLATEGVKEQMP-
LHALNPDFAVLVPVLVTPAVGRVAADAGVLGARVPCDASL-
RELQKSMKGNALPISGIRLLGMRAARAMVDEDIMVAGKVAA-
LLVIVALETITHLVLMNDSEATLPWRLSVAERLEADTKRAGGFAARPFKSKPSLCKTQAHAKIADPIQEI
AAGYVATEDRFVFPVEGIPIQLILASPHSRVGEAKRAGEGHLHEMAKELQAKAVIMVLNEPDTNTEGL
LNYLTTQQPTDLLFEDCLDVVIDMRRMSVV--
VQLNTV-
HVLVPGVRDLADREIMAAAAHSGLVQIERVDERTGRERTLMWMYGAMEELWLMSVRYADWVVDC
SIGAILLHVYAQQINRAFSLERLQLAGWPPEASSWWNTWQLWLNKDGFFSYEAAFRAVKAVLKSDL
DDFDMSDVEGVGRYQSRQQLRVGYRIRQQVAFEQLDPRTDILLQSMTRLSVIRHALTMVAILIVREIV
VEFTLVVAKEDVAMGHVECVLADSFDFTAKDADIGPVGIRTPDPVLGDGTVLKEAVLGKIDLANTVAD
FRYKMLIDGGRELGVSWVTLQNLIYSRIWVPPAQAALKNLVPIAFFLGHTELNLAIFPQLWAGISKARA
NKLMAGALIEKILERVAAFAEPEATTHQSKKIRHFKYYGEEVDPVYLAYILDMFALVATQVTSDRLLKD
RYVLNLKIFRGFTSLLWLSAQIGDKGPAATVITVQTIMGLGALVLFIILL-RVVVLVETLYYKYVY--

MLGYQAAKDVADADPEHFLSLAHASRDVEWVEVIPAFFYYFKTAGSRLTKNTALAKEFFGLFKERLRII
DVLAVPPDRVEVLVTFVALAGTLATSAQALAGIIACGFCEPNAAAVLEADQDWNLHRSMGITLAFYD
ARESVKGWFDKGLHKDVAATGLKEAGALTTPFHAGATAKQCVPAIHRVLDLGEVEAALIEIDSAVVVA
DEEQTLADKPTTRENSVGAVGGVVLAGTMNMGANLDSVERGGNIIIELEEEQYSAVNGKKSDGKVT
DKVVMVLSAAYFPVLKRIASANALDKGAFGLLIDAPDDADNKDHGRELTIPAPTIH-
ESAPLYAESEIERIAWIAPLHLLGTLDAVLGFEPDQYSFISMMIDASLEIMETAEVL-
DYHVLETGSRPPAFRIILIKGLHIRLLAGVCTIIVVGSAGRTEVTAYIQG-
AVPITLNKKTFLPAFAASSCHAIDIFSTMLRPPERSTIQMLILETVAEL-
LEVTRIENDKLERMVALLPGLDVASLLDLVAAATDFAAIVTIDQAITAILSVAAEAVVLREMLLTLAEIAV
TALALALALLLMLTPDQGLHQKEQAREAQMKALIQGGVVLQVKAADRMMLASTADKVRLDTDEGT
TSAFASLNLATVSDGFYVLSSAVLQMTSDLMMIVAVARVLADKETKNALVVRHPEQVKTQSTYAEGS
PGMVLQTLTGAIPPLAIKPETVELLWTLVAALWRVKAHLANAARPHNERVLILILALSLITVLLQSEGGG
MGGG--
GPAAKALAAFSILTIKAKAGDEVRKVARRVMDLNSRLGRPEEAVNPEFRGAELEQSKYEAENVEVDTM
ETIRRFVDIPFLVSTRTQHNESEDRGARMVLDVTRVRFIDL-
VTKRSEIILAARAETDGIIAREMGKDLAALHPEAAALGKIAALQHFIAVQDAHISNYVTAEIHAVLGMILL
VLFIQEVRVGKVVWPTRREVTMVFIMAALAIFFLVIGLGVQIVFMLSGISWNQLTTIVLGEGIMVVRQT
STFPEDS-
IGILQNTYADLICSGRVAKADIEASLHIVVAGRVARGTWYVIYLYEISQAQDQSGIMQRAFRQMTLSQ
SLIDGRMEVKIKVVTSDRYA-
TYEIKGGQHRKGTRYNRVKVGHNIPVQVLPRMKMAFGPPNLVLVKTFGAVVQKRFAQALTHIRHGA
VTIGPMEVSSPYISNKKARHAPGDSVVDLKPDNIVRSVAVVPELIWARENEDNGAVHDKYVKTTHKL
AAKRKAAVDKAQYDHRNQVELSFIGLAVKVLPEKAIVHATSGQLMPTASVAVTDKMKGIAATIRLDP
MEKALIAYIKRMRPQLNLGGQTDKKIVKCLNNNLDDVEYGKLGRRKIKPITIKSVYDVEHRRATELVKL
MPYKDDVESRKSQFAEK--
ALFEPLKAFMMLFGYIARAVYALYLRVIRRTILLVKTKIVVNASTVLSAIKIIGVSIRLRHKSTNVAETNHHI
KTIEARARMTLAGAGKVKLNLSPGKVGKKGGRIKGYMPYKNFNIFDGLASGRRAKVKELAKLKEVDKTI
SVDGVRQKQQGGRTPKAQLSNAI-
NTRVGVDIVGENEQEESNPRANAEMGQIFAKIDARVFSHVGALIGHALEAVLAVVAIWQIGRAHNLL
AVRLKVNAGIEMHSAA-
VEIAVAAAAFAQSAGEASVIADISESYLGALAISAMSVDVCRLRRRGLNELKKTKKDAYMSLTCFGGKG
DKVKIVEATQTQAYEIVATRTAERHGIRAKGTYGKERAMMQILPTKPVKIWVPVFVTEVELLAPIRAFAE
SVGDGVTVVKLTKDIKVTMFEKAVASIAVTGKSTSVVYTVLEKVTELMKDIIYTQRAKAHYGRGRLRSH
VVTTTKQSRHLSMIKRTVVADPSMDAARLTDGEVTVITGATMAFVMSEQEAV--
GDVDLHFHEEPRREAELGIGGPETFLTTEQPRVLALLFLRAYLIHIVEIGTVNGFSDAATIYDVLRAMQN
KLDRKLSADIAKTRMVLTDNVTDMGVTEVAPRREAAGDKILSAFWINGVTANADIILLVANLEVHCAP
ENIDCAVRLFYQRITLQAKERGGDVSVAIVPAANQIEAAENTVIYTIKGKTPGTGQMKLTIEGISRSIVKS
LAVQLFQVDTFLDDTWIATWLRAALQVVFVAAEDGKEAVDHVVERDTELEFAPQKDIVSRKIRIYTPA
APSAVVIWDPAIEREDKAEAQGTILKIYGPNLQDINCFTDIGAEPKIESLKAWDEKEKYVVHRDDIPEDI-
TLVGKSSKVNKEFAIPDVAVCAPSKPGVTESIDRGEPKAGTCGEVANMAYPQESLVNAVSSANVQKN
ISMVLSGHPVGEMGISREIERHLETSMCEIFVDKDDVDVALDFYILQLTPLRVGLDPPNARVQIDGKES
RKLMAIQDGDAALQRHPKVSQATGTGLKAGVKARMPDDALIVDGVMHGDVSFTVTDLPDGRV-
VTPVFNNVKAQLKIDKVLLKAFHVVTLSFSNDKGEEHLIKLRLNALKKSTRHYEMLLKIRETECAESPHAI
SVGKVLKIDALMQTICKAHYKEIEPLLADELTALGVMGEQGLLHIVPRLMILAVLQLLTLEIALQIDDWG

LQAEALVTDQTMGLYLRDRAWSF-AISEVVGNTFFARTKETDTEQKIRARNPV-VLL-LGKFKMI-
ISPSGLGLASTAESPQEQGNADELILSTERLQDEMGYYVLQILLVFVDEVILAYGSGEGPIKFLVAEQAV
TGSQNPGMVTLTRRTFPLELQRNATFVMVFEDSTMKNLSHKKMHMNLIVIKSKGWIVNVSTKILISD
QAIASYAEDAGAHTQPSESALAVITSRVMAPIVLVVSQKPTAPSKTLFLEALSLYDEVTSTVQNKPKKYY
IFEGDVVAVLGTLDAAVLLGMQRPQPDRMRRAIVADAVRLAIALRASGIVTRLDEDLINFSRAVNFAA
LYYPVPK------------EFRVRNHDVSTKRSRPYKAHTKFLHKADVDKGLIATGVDTLA
>GCA_001420005
AVIKPVTMNAYAPVLMIAQSQVATVSGAGSPSLRAKTVYAKSGLQRKSETKWVAKDFYY-
GVVKMANLVQLVSISVDNRAVCTVAQLVEQEVVVHGMVALDAVKKKIALEAIEGMTAEDPAKVVDY
QTRIAIRKWRDADDRADLLPVLLGLTLKIKFDPHQPLVHDLPIPVVYEVI-
GIMAQKGTIEVDDCGISTAVSDVDPIAYVSPIDLAAVSGQKMDYIDQEAAVAQAKQDIDLEELALESG
VLVVGIKPLIIPI-FRDDADE-MSFANRSRTLKTIVDLAVSYPEQTRAR----IAAAM-
MDASRAAALAEKRGTAEEIRIVKRMQRSAGLADDPELVMLEDAAEGNSNWAGQIYDDPISGSSESDS
AGNIVYAANKRQNFARSITFLRTVEISDQAAAPLMRGVKHNAKSRASRKLDDAIAALNFPNVPEGIAI
RAAAGKTTATLLHDADQGEQILMLIVRWHVTWKTAAIIYILEVAGNIVGLIELGGIAAVGRKDGLLNRH
HKLNGSLIEQKRVIDKLTLRLRAALKQKLGQDQVIILVVRVQGDLVAEAEIQMG--
DVGAVVAIQGPTIFVRRHRQTVIGDQEATV-VLKGVAEGPADAQALLRIRVDHTLVKLSVLLIPE-
PVLVESVTIREGVGRALQIIQLDVYLLRDMGTGEETLAKSRSRELIRELLQIDPITGVRLAYGNLREREAKV
LNIKEIDTLF--
LAIIQGVTEPRMGNLPEGVCRTFVRAGPRAAAGMLFLILDDAGKIYTIDGDLREITKSIKSLIGLVDAAKP
KALSAVYMTERLKIRYEDVHVLISDADLYAYAEARRELLRMVPVYITNQAKRRQLVLLALSLRDGHRRV
RMAIDVMIAAAIIESQAVNGAVHHAEPRILLDDAEVVGSEMMINRQAALAMKEVLKVDVIVIFPEFQE
KMVVSLRFFCTDVAIFVIALICTVGAMLSREPSHAQESGAGFQK---AHSMLFALRATFDAQNLRDA-
-ADLFIRLGLAPPRYGILYTVRPKRKQMPSEPDADKVDGFELVHAVKEDGRDN-
KLDMPEAMALAVE-
DATINVSGGVLAMDGDMAAIEKVDSKRKLALAVTKWNIARVSILDLTISGARPIRAFD-
IAIAQVTAALGEKAVS-
LYLPKANNAELEADKIDGAGSSTVDAAADRQQGILLLNPVLVVIQDSNTAALISNEGLDEWGTMAYK
KHYAYAARMRLHMRTIDGIEVGVAMEPNKQKAEKIKTVKLAGDTRFRQLATEGVKEQMP-
LHALNPDFAV-LAVLVTPAVGRVAADQDVLGARVPCDASL-
RELQKSQKGDALPISGIRLLGMRAARAMVEEDIMVAGKVAASLLVIVAVETICKMVLLNDSEATLPWR
LSVAERIEADTARAAGYAARPFMSKPSLCKVQAHANVADPIQEIAAGYVASEQRFVFPVEGLPIQLILA
SPHSRVGAAKRAGLGHLHEMAKELIGKADLMVQAASDTNTEGILNYLTTRQPTDLLHDDCLDIVIDM
RRMSVV---VQLNTV--
VVVSGVRDVGAYEIMQAAAPSGLIQVERIDDRTGRERTMMYMYGAMEELWLLSVRYADWVVDCAI
GAIVLHVYAQVINRAFSLERLQLAGWPPEASRWWNTWQLWLNKDGFFGYEAAFRAQAAVLSAALY
DYEMTDVEGVGRYPSRQELRVGYRIRAQVAFDRLDPRTDILLQSRTRLSVIRHALEVVAIMIVREIVIEFT
LVVAQEDVAMGHVECVLADCFDFTAKNADIGPVGLKTPDPVLGDGTVLNDAVLGKIDLANTVADFR
YRMIDDGGRQLGVEWLTLRNLLVGRIWLPPAEAALKNLQPICFFLGHPDLNLQLLVALWAGISKARM
QALMASALIEQILDKVAAFADPDATTHQSNKVKHFKYYGEEVDPVYLAYILDVFALVATQVTVSDRLL
KDRYVLNLKV-RGFTSLWWLGAQLATKSPAATIIALDSIAGLGALVLFIILL-RDSVLVETLYYRYVY--
MKGYEAAKDVADADPEHVLNLAAVSRDVEYIEIVPAFFYYFKTAG-
FEPFNTAVAREFFGLFKERLRVIDVLVVPPDRVMSLVAFVALAGTLTTSAEALPGIIATGLCEPNAASVLE
ADQDWNLHRSMGITLAFYDARESVKGWFDKGLHKDVAQTGLSEAGALTTPFHVAVTAKACVPAIHR

VLDLGEIEASLIEIDSAVTVADEEQTLADKPTTRDNSVGAVGGVVLAGTMNMGANLDSVERGNNIIIEL
EEESYSAVNGKKD-GKLTDKVLMV-----FVVMERIASANALDRGAFDLLIDVPDDADNRDQGWEL-
---PDQYSFISMMINASLDIMQTAEV--
DYHVLEKGSRPPAFRVILIKGLHVRNLAGIATIIVVGSAGRSEVTAYIQG-
AVPITLKRVTFLPAFAASSCHVIDIFSTMLRPPERSTIKMLILETVAEL-
LEVTRLENDKLERMEVLLPGLDMAQLLPLEAAAADFPQIVTIDASIVAILSVAAEPVVLRDMLLSLAEIA
VEAMALALALLLMLTPDQGLTQKETSREAQMKELIQGGVVLQVRAADRMVLAATADKVRLDTDDG
TTSAFASLNLATVSDGFYVMSSAVLQLSSDLMMIISVARVLADKEHKNALVVRHPEQVKTQSTYAEGS
PGMVLQTLTGAIPPLAIKAEEVELLWTLVTALWAVKAHLANATRPRNERVLILILALTLITVLLQSEGGGI
GGG--
GPPAKGLAAFSILTIKAKAGDEVRKVTRRVLDLNSRLGRPEESVNPEFRGAELEQSKYEAANVEVDTME
TIRRFVDIPFLVSTRTQHNESEDRGARMVLDVTRVRFIDL-
VTKKSEIILAARAETDGIIAREMKGDLAALHPEAAALGKIAALQHFIAIQDAHISNYVTANIHSVLGMLLL
VLFINETRIAKVVWPTRREVTMVFIMAALATFFLVIGLVISIVFMLSGISWNQMTTIVLGEGVMAVRQTS
TFPKDS-
IGILQNTFADLICSGRVAKADIEKSLHIVVAGRVTRGTWYVIYLYEISQAQSPAGIMQRAFRQKTLSQSLI
DGRMEVKIKVVTSDRYAQTYEIKGGQHRKGTRYNRVKVGHNIPVDVLPRMKMAFTPPNLVLVKTFG
AVVQKRFASALTHMRHGAVQIGPMEVSSPYIRNKKARHAPGDAVVDLKPELIVRSVAVVPELIWARE
NEDNGAVHDKYVKPTHKLKAKRTTAVDKAQYDHRNQVEMSFIGLAVKVLPQKAIVHATAGMLMPT
TSVAVTEGMKGIAATIRLDPMEKALIAYIKRMRPQLNLEGRTDKKIVKCLNNNLDDVEYGKLGRRKIKPI
TIKSVYDVESRRATELVKLLPYKDDVEGRKSQFAEK--
ALFEPLKAFMMLFGYIARSVYALNLRVIRRTILLVKCKIVVAAATVLSAIKIIGLSIRLRHKSTNIADTKHHIK
TIEDRANLTMAGAGKVKLNLSAGKVGKKGGRIKGYMPYNNYNIFDGLASGRRAKVKEVAKLKKVDKEI
KVEGIRVKQEGGRTPKAQLSNAVANTRVGVDIVGEKIQEQSNPRANAEMGQIFAAIDARVYSHVGAL
IGHALEAVLAIVAIWGIGRAHNLLALKLRINAGIEMHSAA-VTRAVACA-
FAAPAGDASALWDASEAYLGAIAITEHAVDTCRLRRRSLSELKKTKKDAYMSLTCFAGKGDRIKIVEAS
QTQAYEIVATRTAERHGIKAKGTFGKERAMMQILPTKPVKVWVPVFVDEVELLAPIRNFAETVGDGVT
VVKLQNDIKITPFEKAVASIAVTGKSTSVVYTVLEKVTELSKDIIYTQRAKAHYGRGRLRSHVVTHTKSSR
HLSMMKRTVVADPSMDAARLTDGEGSVVTGATMAFVMSEQDAV--
GDVDMHFHEEPRREADLGIGGPETFLATEHPRTLAVLFLRAYLIHIVEIETTKDFSNAATIYDVLRAMAN
KLDRKLSADIAKTRMVLVDNLMDMGVTEVAAKRDAAGDKILSAFWINGVTANADIVLLVANLEVPTV
PENIHCAIRLFYQRITLQAKERDGDVSVAVVPANKQIEAAEDTVIYSIKGKTPGNAQMKLTIEGISRSVV
KSLAVQLFQVDTFLDDTWLGTLLRAAIDVVFVDAGETKEAVDHVVERDTEYEFAPQKDIVSRKIRIYTP
AAPSAVVIWDPAIDREDKADAQGTILKIYGPNLQDINCFTDIGAEPKIESLKAWDEKEKYVVHRDDIPE
DIDTLVGKSSKVNKEFAIPDVAVCAPSKPGVTESIDRGEPKAGTCGEVANMAYPQESLVNAVSSANV
QKNISMVLAGHPVGEMGISREIERHLETNMCEIFVDKADVDVALDFYILSLTPLRVGLDPPNARVSIDG
KESRKLMAVMDGDADLQRHPKVSQATGTG-NASIKARLPDDTLIVDGVVHGDVSFTATDLPEGRV-
VTPVFNDVKAQLKIDKVILKAFHVVTLSFSNGKGEDHLIKLRLNALKKNTRHYEMLLKIRETECAESPHAI
SVGKVLKIDSLLQTICVAHYKAIEPLLADDLTALGVMGHQGLLHVVPRLMILAVLHLWGMAAAMEVA
QWGLQANA-VTDQTMGLAMRERHFTY-SISEVVGNTFFAKTKEDDTEQRLHARNPI-VLL-
AAKFGMV-
ISPSGLKLTSTQASALGRENAEDLILSTERLQDAMGYYVLRILLVFVDEVILAYGSGEIPIKFLVGDQAVT
GRQNPGMVTLTRRTFPLDLQRHAVFVMVFEDCTMKNLSHKKMHMNLILIKSKGWIVNVSTKILISDQ
AIASYAEDAGAHTQPSESALAVITSRVMAPIVLVVSQKPTAPSKTLFLEALALYDEVTATVQNKPKKYYI

FEGDVVSILGACDAAVLLGMQRPQPDRMRRAVVADAIRLAIALRASPITTRLEAGLINFARSVSLAAG
VYIQFQTPP-APMP-PALAFRVRNHSVSTKRSKPYKGHTKFLHKADINKSLIAAGVDTIA
>GCA_002150005
AVVKPVTMSAYAPVLLIKQQQVATVSGAGSPSLRAKTVYRKSGLQRKTSTKWVAKPIYY-
GVLKMGQIVQLVSISVDNRSVCTVAQLVEQDVVVHGMVALEAVKKKIALQEIDGMTSEDPAKVVDY
-
DAIALKRFRDAEDRADLLPVMLGLTLEIKFDPHEPLVHDLAIPVIFEVVSTVMAQKGTIEVDDCGISTAIS
DVDPVAYVSPIDLNVVAGDQMAYIDQEAAVAAAKEAVALEELALESNVLVVGI-PLLIPV-
FEKDGDT-MSFANRSRTLKTIVDLAVSYPQMERARIGVSIASAM-
MEAAQAAALSEKRGTAEEIRIVKRMQKSSGLADNAELVILEDAAEGNSSWAGQIYDDPISGSSESDSA
GNIVFAANKRQNADRSITFLRTVEISDKAAAPLMRGVKHNSKSRASRKVDDAIRELNFANVPEGIAIRA
EAGKPQDMLLHDAEEGEEILMIIVRWGVTWKTAILILLAVTAGNVVGVIEISGIGAVGRKDGLLNRHHK
LNGSLIEQKRVMDKLTLRLRAALKQKLGAEQVIILVVKVAGDLVADAKIQM---
SVGAVVAIQGETIFVRRHKQTVITDQEATVEVLRGVAEGPADAQALLRIRVDHTVVDLSVLLIPEVPVL
VDSVSIREGVGKALQIIQLDVYLLRDMGTGAETLAKSNSRELLRDVLQVDPITGVRLAYGHLSERLTRGI
TLKKLR-----
LSILFGVTEPRMRNLPEGVYRDLVEAGPQAAVGMIFLLLDDAGKLYLIDGDLRELSKGIKSLIGLVDAAR
PKVLSAQYLTAKLKIRFADVMVLNSEADLYAYKEERKELLRMVPVYIVNAAKRRQLIIMALSLRSGHKR
AGAAIRVMLAAAIIESQQTRGEVHHEEPEVLLDDAEVVGTEVMISRQASLELKEILKVDILVVFPEFNEK
MVVSLKFFCTDVAIFVIALICTVGQMLSREPSTAQEGGAGYGKRIRKHSVLFALSVTFDRRNTRDA-
TGDLFLTLGIADPRYGVFYTNQPKKKQMPSEPDDDKVDGFDLVNAIKADGAE-
QALDLPEAMALAVE-
DATINVSAGVLALSAEMAAIERIESKRKIALAVEKWTIAKVDVLDIHISAAASFRGLD-
IEQADLSAALGSKQI-
FLFLPGANNADLEEDRLNGAGASTIDVAEDKQNGILLVVPVIVVIQDSNAASLVANGGVEEWGTMAY
KKHYAFAARMRLHMRTIDAIEIGVALDPNKQKAEKIKTVKLDGDTRFRQLAVEGIKEQMP-
LHALNPDFAV-LAILVTPAVGRVAADEEVLGAKVPCDFSL-
RELQKSMKGQALPISAIRILGMRAARAMIEEDIMVAGKIAA-
LLVIGALETILKIVLLNDTEATIPWRLSVAERLEADTKRDGGFASRPFNSKPSLCNIQAHANVADEIQEIA
AGYIAMSNRFVFPVEDIAIQLILANPHSRVGTEKRAGEGHLHEMAKELKAKAAIMIRGEPNTDTEGIKT
YLTTRQPTDVLMPDCLDLVIDLRRMNVV--
IQLNTV-
HPVVPGVGDIAKYEIMAAAFPSGLVQTEQVDDRTGRERTMMWMYGSMEDLWLMSARWADWVV
DCAIGAIILHVYAQSINRAFSLTRLQLDGWPKEADRWWNTWQLWLNKDGFFGYEAAFRA-
LQVLKAELYDFEMKDVNGVGRYQSRQELRVGYRIRQNVDFEKLTPRTDILLQSQSRLSVLRHALKVVA
ILIIRDFVVEFTLVVAQEDVAMGHVECVLADCFDFTKKNADIGPVGLRTPDPIFGDGTVFKDAVLGKID
LANTIADFRYLFIIDGGRELGVWWVTLQNMLYERIWLPPAAAKLKDLTPICFFLGHPEVDL---------
ISKARAQRLMAGALIEQILDMVAAFAEPDATTHQSKKEKHFKYYGEEVDPVYLAYILDMFALVSTQVT
VSDRLLKDRYVLNLKI-
RGFTSVMLLCAQLGNKSPAATIITVGTIAGLGALVLFIILLMRDSVLIETLYYRYVY--
MQGYDAAKDAADADPEHLLVLAHATRDVEWIEVVPAFFYYFKTAK-
FEPFNTAVAKEFFGLFEERLRVVDVLVAPPDRVEAIVAFVALAGTLATSAKALVGIIACGFCEPNAAAVL
EADQDWNLHRTLGITLAFYKAQESVKGWFDKGLHKAVAEVSLPEAGALTTPFHAGKEAKVCVPAIHR
VLDLGEIEAALIEIDSAVVVADEEQTLADQPTTREKSVGTVGGVVLAGTMNMGAALDQVERSGNIIIEL

EEEQYHAVNGKKGDGKLTDKILMVLSAAYFPVMKRMTSANALNRGVFELLIDAPDDADNQDHGGE
LTIPAPTIH-ETTPLYAESEIERIAWIAPIHMLGTLPAVL-
FEPDQYGFISMMIEASLDITATAEVLDDYHVLEKGSRPSAFRVILIKGLHIRQMAGIGTIIVVGSAGRSEV
TAYIQH-AVPITLRRNTFLPAFAPSSCFAVDIFSTMLRPPERSTIETLILETVSEL-
LEVTRIENEKLERLLTLLPGLDMAELLELMAAAADFADIVTIDAAIAAILSVAAEAVVLRELLLNLAEIAVT
ALALALALLLMLTRDNGLTQRETAREAQMKALIQGGVVLQIRAADKMVLASTADKVRLNTDEGTTSA
FASLNLATVSDGFYVLSSAVLQLSSDLMMIVAVARVLADKESKNALVVRHPEQVKTQPTYAEGSPGM
VLQTLTNAIPPLAIKPEEVELLWTLVAALWRVKAHLASAARPHIERVLIMILALALIVVLMQSDGGGMS
GG-
ASGAAKGLAAFSLLTIKAKAGDEVRKVTRRVLDLNSRLGRPEEAVNPEFRGAELEQTKYEAENVEVDC
METVRRFVDIPFLVSIRTQHNESEDRGARLVLDVTRVRFIDL-
VTKRSEIILAARVETDGIIARDMAGDLQALQPELAALGKVAALQHFIAIQDAHISNYVTAAIHSVLGLLL
LVLFIQQTRIAKVVWPTRREVTMVFIMTVLALFFLTIGL--
QIVFFLSGISYQKLTTILLGEGVMAVRQTTTFPADSFIGILQNTYADLIAAGRVAKADIENSLHIVVGGRV
ARGTWYVIYLYEITQAQQQSGIFQRAFRQATLSQSLIDGRAEVKIKVVTSDRYA-
TYEIKGGQHRKGTRYNRVKVGHNIPVQLLTRIKMAFGDPNLVLVKTFGAVVQKRFAQPLTHIRHGAV
TIGPMEVSSPYIKSKKARHAPGDSVVDLKPDNVVRSVAVVPELIWARENEDNGAVHDKYTKTTHKLA
AKRRRAVDKAQYDKRNQVELSFIGLAVKVLPEKAIVGSTAGTLLPTGTVSVTENLKGVAATIRIDPMEK
SLIAYIKRMRPQLNLAGKSDKKIVKCLNNNLDDVEYGKLGRRRIKPLTIKSIYDVESEKATELVKLFPYKD
DVEGRQSQFAEK--
AIFEPLKAFMMMFGFIARAVYALSLRVVRRTILLVKTKIVVNANTVLSAIKIIGISIRLRHKSTNVADTNHH
IKTIEARARMTLAGAGKVKLNLRPGRVGKKGGRIKGYMPYKNYNIFDGLESGRRAAVKELAKLKTVDKE
ITVDGIRTRQ-
GGRLPKAQLSNAIANSRVDVDIVGENEQQQSNPRANAEMGQIFAAIDARVYHLVAALIGHALEAVL
AIASIWGIGKAHVLLAVKLKINAGIEMHSAA-VDRAVACA-
FAKSGGEATTFADVSEGDLGAISISEDSLDTCRLRRRSLSELKKTKKDAYVSLTCFSGKGERVKILEASQT
QAYEIVSTRTAERHGIHAKGTYGKERAMMQILPTKPVKVWVPVFVNEDELLAPVAAFAETNGDGVTV
VKLTKDIKVTMFEKAIASLAVAGKSTSVVYTVLEKVTELAKDIIYEQRAKAHYGRGRLRSHVVTHTKSNR
HLSMIKRTVVASPRMNAARLHDGEVSVVTGATMAFVMSEQDAV--
GDVDMHYHEEPRREADLGIAGPETFLTTDQPRVLALLFLRAYLIHIVDIESVNGFATAATIYDILRAMQ
NKLDRKLSADIAKVRMCLHDDVADMGVTDEAPRRDEAGDPILSAYWINGVPANADIILLVANLEVET
APENIDCAVRLFYQRITLQAKERNGDVSIAVVPAQNQIEAAEGTIIYTVKGNNPGTAQMKLNVEAISR
SIVKSFAVQLFQVDMFLNDTWLGTLLRAAEEVVFVQAEESKEAVDHVVERDTELEMAPQKDIVARKIR
IYTPAAPSAVVIWDPAIEREEKADAQGTILKIFGPDLQDIACFTDIGAEPKIESLKAWDEKEKYVVHRDDI
PEDIETFVGKSSAVNKEFAIPDVAVVAPAKPGVTESIDRGEPKAGSCGEVANLSYPQESLVNAVSSANV
QKNISMVLQGHPVGELGISRSVERHLETTMCEIFIDSPDIDVAMNFYTLSLTPLRVGLDKPNAKVEIDG
RESRKLMAVLDGEAELQRHPKVSEATGLTMNAGVKAKLPDDALIFDGVQHGDVKFTNTDLPEGRV-
VTPVTLKVKAQLEIDKVILKTFHVLTLSFSNDKGEEHLIKLRLNALKKNTRHYEMLLKIRETECAESPHAIS
VGKILKIDALLQTICQAHYTAIEPLLAEDLTALGVMGDQGLLHVVPRLMIMAVLDLANVEARMFIHRW
ALMAEACVTDETMGLDVREREWTY-AISEVVGNTFFARTAEDDTEQRLHARNPI-VLL-YGKFDMV-
ISPSGLKLASTQPSELGEGNEAQVILSADSLQDEMEYYVLELLLVFVDEVILAYGSGEEPIKFLVAEQAVT
GRANPQMVTLTR-
TFPLDLQRDATFVIVFEDSTMKNLSHRKMHMNLVLIKSKGWIVNVSTKILISDQAIASYAEDAGAHTQ
PSESALAAITSRVLAPIVLVVSQKPAAPSKSLFLEALALYDAVTATVLNKPKRYYIFEGDVVSVLGALDAS

VILGMQRPQPDRMRRAVIALAIRLAIAARASGSVATLSNDLVNFSRAVNFVSRIYTVPQT----------
-EFRVRAHKVSTKRSKPYKAHTKFLHKAELEKSLVAEGVDTLA
>GCA_002158905
AVIRPVTMKTYAPVLMIAQSQVATVSGAGSPSLRAKTVYAKSGLQRKAETKWVVKDFYY-
GVVKLGDLVQLVSISVDNRAVCTVAQLVEQEVVVHGMVALDVVKKKIALEAISGMTAEDPAKVVDF
AGELELRKFRDADDRADMLPILLGLTLKIRFDPHQPLVHDLPIPVVFEVV-
GIMVQRGTIEVDECAISTAISDVDPIVYVSPIDLAAVAGDKMAYIDQESAVSAAKADIAL--
LALESAVLVVSI-PLLIPV-FAADADV-MSFANRSRTLKSIVNLAVSYPAIKRA------AAAL-
MEASQAAALAEKRGTAEQIRIVKRMQRSAGLADDPDLVLLEDAAEGNSNWAGQIYDDPLSGSSESD
SAGNIVFAANKRQNFQRSITFLRKVEISDQAAAPLMRGVKHNAKSRSARKVDDTIAALNFPNVPEGIA
IRAAAGSQIHVLLHDATEGEEILMIIVRWHVTWKTAIIISLFPTAGDIVGLIALGGIGAVGRKDGLLNRHH
KMNGALIEQRRIVEKLTLRLRAALKQKIGAEQIIIFVVKVAGDLVAKAKIQM---
AVGAVVAIQGETIYVRRHRQTVIADEDATVEVLRGVAEGPADAQALLRIRVEHTLVKLSVLLIPE-
PVLVESVSIREGVGKALQIIQLDVYLLRDMGTGEETLAKSRSREMIREILQVDPITGVRLAFGILRERLVKG
GTMKELGTLL--
IAIIDKITEPRMANLPDGVCRTFINAGPRAAVGMIFLLLDDAGKIYTIDGDLRELTKSIKSLIGLVDAAKPKI
MSAAYAAARLKVRYADVTVLVSDANLYAYAEERRELLRMVPVYITNAAARRQLIIFALSLRGGHRRAR
AAIGVLIAAAILESQAAQGAVNHAQPAVLLDDGEIVGSEMMISRQAALDLKQIIKTDVIVIFPEFQEKM
VVSLKFFCTDVAIFVIALICTVGAMLSREPSSAQEDGAGFQKRIRAHSVLFALVATFDRRNLRTS-V-
NLFLALGLAPPRYGILYTEQPKRKQMPSEPDKDEVDGFDLVHAIKEDTAS-QELDMPEAMALAVE-
DATINVTAGVLAMTGDMAAMEKVDSKRKMALAVTKWTISAEAIVDILISGAHPFRVLG-
IESADVSAGLPQKAIWFLFLPGANNAEMEEEKLDGAGASTAEAAKNKQTGILLVNPVLVVIQDSNTAA
LIANGGMEEWGTMAYKKHYALAARMRLHMRTID------
QMEPNKQKAEKIKTVKLEGDTRFRQLATEGVKEQMP-
LHALNPDFAVLVPVLVTPAVGRVAADEQVLGARVPCDASL-
RELQKSMSGEALPISGIRLLGMRAARAMVDEDIMVAGKVAA-
LLVILALETITHLVLRNDSEATLPWRLSVAERLAADMKRASGYAARPFQSKPSLCNTQAHAKIADPIQEI
AAGYVAKPERFVFPVEGIPIQLILASPHSRVGSAKRAGLGHLHEMAKELHAKAVIMVLNEPDTNTDGLL
NYLTTQQPTDLLFEDCLDVVIDMRRMNVV---
VQLNTV-
HVVIPGARDLADREIMAAAAPSGLVQVERVDDRTGRERTLMWMYGAMEELWLLSLTYADWVVNC
AIGAILLHVYAQVINRAFSLDRLQLAGWPREAHRWWNTWQLWLNKDGFFGYEAAFRAVLAVLSAD
LDDYEMSDVEGVGRHLSRQQLRVGYRIRAQVAFAQLDPRTDILLQSRTRLSVIRHALAMVAILIVRDIV
VEFTLVVAKEDVAMGHVECVLADSFDFTAKDADIGPVGIRTPDTVLGDGTVFNEAVLGKIDLANTVA
DFRYKMLANGGRELGVSWITLQNMLYARIWLPPAQAALKNLVPIAFFLGHTELNLAIFPALWAGISKA
RAQKLMAGALIEKILAKVAAFADPEATTHQSKKAPHFKYYGEEVDPVYLAYILAVFALVATQVTVSDRL
LTDHYVLNLKI-RGFTSLLWLSAQIADKGPAATVITVDTIMGLGALVLFIILL-RASVLVETLYYRYVY--
MLGYQAAKDVADADPEHFLSLAHASRDVEYIEIIPAFFYYFKTAT-
FEPFNTALAREFFGLFEERLRIIAVLAVPPDRVLDLIDFVALAGTLPTSADASPGVIACGFCEPNAAGVLE
ADQDWNLHRSMGITLAFYDARESVKGWFDKGLHKDVAATGLREAGALTTPFHAGATAKQCVPAVH
RVLDLGEVEAALIEIDSAVLVADEEQTLADKPTTRENSVGAVGGVVLAGTMNMGANLDSVERAGNIII
ELEEEHYSAVNGKKADGKITDKVLMVLSEAYFPVLRRMASANALDKGRFGLLIDAPDDADNQDHGRS
LTIPAPSVHSESAPLFAETEIERIAWIAPIHLLGTLDAVLGFEPDQYSFISMMIDASLNIMKTAEVLDDYH
VLEKGSRPPSFRIILIKGLHIRLLAGICTIIVVGSAGRAEVTAYIQG-

AVPITLNKQTFLPAFAASSCHAIDIFSTMLRPPERSTIQTLILETVTEL-
LEVTRLENDKLERMETLLPGLDIAELLGVVAAAGDFTAIVTIDRAITAILSVAAEAVVLREMLLTLAEIAVT
ALALALALLLMLTPDQGLHQKDHARESQMKDLIQGGVVLQVKAAERMMLASTADKVRLDTDEGTT
SAFASLNLATVSDGFYVLSSSVLQMSSDLMMIIKVARVLADKETKNALVVRHPEQVKTQSTYAEGSPG
MVLQTLTGAIPPLAIKPEAVELLWTLVAALWKVKAHLANAARPHRERVLILILALSLITVLLQSEGGGMG
GG--
NPPAKMLAAFSILTIKAKAGDEVRKVTRRVMDLNSRLGRPEESVNPEFRGAELEQSKYEAENVEVDTM
ETIRRFVDIPFLVSIRTQHNESEDRGARMVLDVTRIRFIDL-
VTKKSEIILAARAETDGIIAREMGKDLAALHPQAAALGKIAALQHFIAIQDAHISNYVTADIHAVLGMLL
LVLFIQEVRVAKVVWPTRREVTMVFILAALAVFFLVIGLGVQIVFMLSGISWNALTTIVLGEGIMVVRQT
STFPEDS-
IGILQNTFAELICSGRVAKADIENSLHIVVAGRVARGTWYVIYLYEISQAQGQSGIMQRAFRQMTLSQS
LIDGRMEVKIKVVTSDRYA-
TYEIKGGQHRKGTRYNRVKVGHNIPVQVLPRMKMAFTPPNLVLVKTFGAVVQKRFAQALTHIRHGA
VTIGPMEVSSPYIQNKKARHAPGDAVVDLKPDNIVRSVAVVPELIWARENEDQGAVHDKYVKTTHKL
AAKRKKAVDKAQYDHRNQVALSFIGLAVKVLPEQAIVHATSGQLMPTASVAVTEGMKGIAATIRLDP
MEKALIAYIKRMRPQLNLAGKQDKKIVKCLNNNLDDVEYGKLGRRKIKPLTIKSVYDVESRRATELVKL
MPYKDDVESRKSQFAEK--
ALFEPLKAFMMMFGFIARAVYALYLRVIRRTILLAKTKVVVNADSVLSAIKIIGVSIRLRHKSTNVAETNH
HIKTIEKRARMTLAGAGKVKLNLSPGKIGMKGGRIKGYMPYKNYNIFDGLASGRRAKVQDLAKLKNVD
KTIAVDGIRQKQQGGRTPKAQLSNAI-
NTRVGVDIVGENEQEESNPRANAEMGQIFAAIDARVFSHVGALIGHALEAVLAVVTIWGIGRAHSLLA
VRLHINAGIEMHSAA-VPQAVAAA-
FAQNAGEASLIADVSEGDLGALAVTAMSVDVCRLRRRTLAELKKTKKDAYMSLTCFGGKGDKVKIVE
ATQTQAYEIVATRTAERHGIRAKGTYGKERAMMQILPTKPVKIWVPVFVSEEELLAPIRAFAETQGDGV
TVVKLTKDIKVTMFEKAVASLAVTGKATTVVYTVLEKVTELMKDIIYTQRAKAHYGRGRLRSHVVTTTK
QRRHLSMIKRTVVADPSMEAARLTDGEVSVITGATMAFVMSEQEAV--
GDVDLHYHEEPRREVQFGIGGPETFLTTEQPRLLAVLFLREYLLHIVEIGTTNGFADAATIYDVLRAMVH
KLDRKLSADIAKTRMVLVDDLMDMGVTEAAARREAAGDKILSAYWINGVTANADIVLLVANLECPCV
PENIDCAIRLFYQRITLQAKERGGDVSVAVVAADKQIEAAEGTVIYSIKGKTPGTAQMKLTIEGISRSVV
KSYAVQLFQIDTFMDDTWIATWLRAADDVVFVAAEDGKEAVDHVVERDTAVEFAPQKDIVSRKIRIY
TPAAPSAVVIWDPAIDREDKAEAQGTILKIYGPNLQDINCFTDIGAEPKIESLKAWDEKEKYVVHRDDIP
DDIDTLVGKSSKVNKEFAIPDVAVCAPSKPGVTESIDRGEPKAGTCGEVANMAYPQESLVNTVSSANV
QKNISMVLAGHPVGEMGISREIERHLETNMCEIFVDKADVDVALDFYTLVLTVLRVGLDPPNARVQID
GKESRKLMAIQDGEGALQRHPKLSAATGTGLGAGVKARMPDDALILDGVVHGALSFTATDLPAGRV
-
VTPVFNTVKAQLKIDKVLLKSFHVVTLSFSNNKGEEHLVKLRLNALKKNTRHYEMLLKIRETECAESPHA
ISVGKVLKIDKLLQTICIAHYKAIEPLLADDLTALGVMGRQGLLHVVPRLMILAVLHLM--
QCAMILPAWGLQQRALITDQTMGLAIRARAWGY-AISEVVGNTFFARTKEDDTEQRIHARNPV-
VLL-LGKFTMT-
ISPSGLGLQSTPESDQDLGNAEPLILSPERLQDEMGYYVIAILLVFVDQVILAYGSGDEPIKFLVADQAV
TGKQNPGMVTLTRRTFPLELQRDATFVMVFEDSTMKNLSHKKMHMNLVLIKSRGWLVNVSTKILISD
QAIASYAEDAGAHTQPSESALAVITSRVMAPIVLVVSQKPTAPSKSLFLEALALYDAVTATVLNKPKKYF
IFEGDVVSVLGGLDAAVLLGMQRPQPDAMRRAIVAAAIRLAIAALASGIITRLEQGLINFSRAVSFTQG

AYPL--------------AFRVRNHDVSTKRSKPYKAHTKFLHKADVDKSLIAEGVDTLA
>GCA_002797915
AVIKPVTMDKYAPVLMIRQQQVATVSGAGSPSLRAKTVYRKSGLQRKSETKWVSKDFYY-
GVVKLGNIVQLVSISVDKRSVCTVAQLVDQEVVVHGMVALEAVKKKIALEAIEGMTAEDPAKVVDYA
SEIELRKFRDTDDRADMLPVMLGLTLKIKFDPHQPLVHDLPIPVVFEVV-
GVMVQRGQIEVDECGISTALSDVDPIVYVSPIDLAAVAGDKMAYIDQEAAVTAAKAEIELEELALESQV
LVVGI-PLLIPV-FEADGDV-MSFANRSRTLKTIVNLAVSYPAIKRA-----IAAEM-
MDASQAAALAEKKGTAEEIRIVKRMQKSAGLADDPELVMLEDAAEGNSNWAGQIYDDPISGSSESD
SAGNIVFAANKRQNFARSITYLRTVEISDQAEAPLMRGVKHNAKSRSSRKLNDAIAVLNFPNVPEGIAI
RAEAGRPRDLLLHDATEGEEILLIIVRWHVTWKTAVVISLF-
QADNIVGIIEMGGIGAVGRKDGLLNRHHKLNGSLIEQRRIVEKLTLRLRAALKQKIGKEQIIIFVVKVAGD
LVAKAKVQM---
SVGTVVAIQGETIFVRRHRQTVIADQEATVEVLRGVAEGPADAQALLRIRVDHTVVKLSVLLIPD-
PVLTESVTIREGVGKALQIIQLDVYLLRDMGTGDETLAKSKSREVIREVLQVDDITGVRLAFGQLKERLV
KGGTMKELGTL---
IAILKRITEPRMANLPDGVCRTFINAGPRAAVGMIFLLLEDKGKIYSIDGDLRENTKSIKSMIGLVDAAKP
KTLSAGYATARLKIRYADVTVLTSDADLYAYAEERRELLRMVPVYITNEAKRRQLIIFALSLKGGHRRAR
AALGVMIATAILESQAKQGPVNHKDPDVLLDDGEIVGSEIMISRQAALDIKQIIKTDIIVIFPEFQEKMVV
SLKFFCTDVAIFVIALICTVGEMLSREPSHAQEEGAGFQKRIRGHSVLFALAVTFDRRNLRDA--
PQLFLELGLAPPRYGVFYTEQPKRKQMPSEDDKKQVDGFDLVAAIKEDATK-EELDMPEAMALAVE-
DGTINVNAGVLAMTGDMAAVEKVDSKRKMALAVEKWTIATVALVDLKLSGARPFRACK-
IEQADVSATLPKKQVWFLFLPGANNAEMEEEKLDGAGSSTAEAAEDKQLGILLVEPVLVVIQDSNTAS
LIANGGMEEWGTMAYKKHYAFAARMRLHMRTID------
VMDPNKQKAEKIKTVKLEGDTRFRQLATEGVKEQMP-
LHALNPDFAVLVPVLVTPAVGRVAADAGVLGADVPCDASL-
RELQKSMKGNALPISGIRLLGMRAARAMVDEDIMVAGKVAA-
LLAIVALETITHLVLMNDSEATLPWRLSVAERLEADTKRAGGFAARPFNSKPSLCKTQAHAKIADPIQEI
AAGYVATDERFVFPVEGIAIQLILASPHSRVGAAKRAGLGHLHEMAKELMGKAVVMVLNEPDTNTEG
LLNYLTTQQPTDLLFEDCLDVVIDMRRMSVV--
VQLNTV-HVLVPGVRDLGDREIMAAAAHSGLVQIERVDERTG----------------------------
----
INRAFSLERLQLAGWPPEASSWWNTWQLWLNKDGFFSYEAAFRAVKAVLKADLDDFDMSDVEGVG
RYQSRQQLRVGYRIRQQVAFEQLDPRTDILLQSMTRLSVIRHALPMVAIMIVREVVVEFT-
VVAKEDVAMGHVECVLADSFDFTAKDADIGPVGIRTPDPVLGDGTVMKEAVLGKIDLANTVADFRYK
MLVDGGRDLGVSWVTLQNLMYARIWVPPAQAALKNLVPIAFFLGHTELNLAIFPGLWAGISKARAN
KLMAGALIEKILEKVAAFADPEATTHQSKKLRHFKYYGEEVDPVYLAYI----
SLVATQVTVSDRLLTDRYVLNLKIFRGFTSLLWLSAQIGDKGPAATIITVQTIAGLGALVLFIILL-
RVVVLVETLYFKYVYV-
MLGYQAAKDVADADPEHFLSLAHASRDVEWIEVVPAFFYYFKTATSRMSKNTALAKEFFGLFKERLRV
IDVLVVPPDRVEALVAFVALAGTLATSAQTLPGVIACGFCAPNAAAVLEADQDWNLHRSMGITLAFY
DARESVKGWFDKGLHKDVAATGLREAGALTTPFHAGDTAKQCVPAIHRVLDLGELEAALIEIDSAVVV
ADEEQSLADKPTTRENSVGAVGGVVLAGTMNMGANLDSVERGANIIIELEEEQYSAVNGKKADGKV
TDKVIMVLSAAYFAVLKRIASANALDKGAFGLLIDAPNDADNKDHGRRLTIPAPTVHAESAPLYAESEI
ERIAWIAPLHLLGTLDAVLGFEPDQYSFISMMIEASLEIMETAEV--

DYHVLETGSRPPGFRIILIKGLHIRLLAGVCTIIVVGSAGRTEVTAYIQG-
AVPITLNKQTFLPAFAASSCHVIDIFSTMLRPPERSAIEMLILETVAEL-
LEATRIENDKLERMVALLPGLDAAALLDLVAAAKDFAAIVTIDQAITAILSVAAEAVVLREMLLTLAEIAV
TALALALALLLMLTPDQGLHQKEQAREGQMKALIQGGVVLQVKATDRMMLASTADKVRLDTDEGT
TSAFASLNLATVSDGFYVLSSAVLQMSADLMMIVAVARVLADKETKNALVVRHPEQVKTQSTYAEGS
PGMVLQTLTGAIPPLAIKPETVELLWTLVAALWKVKAHLANAARPHIERVLILILALSLITVLLQSEGGG
MGGG--
GPAAKALAAFSILTIKAKAGEEVRKVARRVMDLNSRLGRPEEAVNPEFRGAELEQTKYEAENVEVDTM
ETIRRFVDIPFLVSTRTQHNESEDRGARMVLDVTRVRFIDL-
VTKRSEIILAARAETDGIIAREMGKDLAALHPEAAALGKIAALQHFIAVQDAHISNYVTAAIHAVLGMLL
LVLFIQEVRVSKVVWPTRREVTMVFIMAALAVFFLVIGLGVQLVFMLSGISWNQLTTIVLGEGIMVVRQ
TNTFPEDS-
IGILQNTYADLICSGRVAKADIEASLHIVVAGRVARGTWYVIYLYEISQAQDQSGIMQRAFRQMTLSQ
SLIDGRMECKIKVVTSDRYA-
TYEIKGGQHRKGTRYNRVKVGHNIPVQVLPRMKMAFGPPNLVLVKTFGAVVQKRFAQALTHIRHGA
VTIGPMEVSSPYISNKKARHAPGDSVVDLKPDNIVRSVAVVPELIWARENEDNGAVHDKYVKTTHKL
QAKRKAAVDKAQYDHRNQVELSFIGLAVKVLPEKAIVHATSGALMPTASVAVTEGMKGIAATIRLDP
MEKALIAYIKRMRPQLNLGGQTDKKIVKCLNNNLDDVEYGKLGRRKIKPITIKSVYDVEHRRATELVKL
MPYKDDVESRKSQFAEK--
ALFEPLSAFMMMFGFIARAVYALYLRVIRRTILLVKTKIVVNASTVLSAIKIIGVSIRLRHKSTNVAETNHHI
KTIEARARMTLAGTGKVKLNLSPGKVGKKGGRIKGYMPYKNFNIFDGLASGRRAKVKELAKLKNVDKA
ITVDGIRTKQQGGRTPKAQLSNAI-
NTRVGVDIVGENEQEESNPRANAEMGQIFAAIDARVFSHVGALIGHALEAVLAVVAIWDIGRAHNLL
AIRLKINAGIEMHSAA-
VEVAVAAAAFAQSAGEASVIADISESYLGALAISETSVDVSRLRRRSLSELKKTKKDAYMSLTCFGGKGD
KVKIIEATQTQAYEIVATRTAERHGIRAKGTYGKERAMMQITPTKPVKIWVPVFVNETELLAPIRAFAES
TGDGVTVVKLTTDVKVTMFEKAVASIAVTGKATSIVYTVLEKVTQLMKDIIYTQRAKAHYGRGRLRSHV
VTTTKQRRHLSMIKRTVVADPSMDAARLTDGEVAVITGATMAFVMSEQEAV--
GDVRLHYHEEPRREAELGIGGPETFLTTEQPRVLALLFLRAYLIHIVEIGTVNGFSDAATIYDVLRAMQN
KLDRKLSADIAKVRMVLSDNVTDMGVTDVAPRREAAGDKILSAFWINGVTANADIILLVANLETHCVP
ENIDCAIRLFYQRITLQAKERGGDVSVAIVPAANQIEAAENTVIYSIKGQTPGTGQMKLTIEGISRSIVKS
LAVQLFQVDTFLDDTWIATWLRAALQVVFVGAEDGKEAVDHVVERDTELEFAPQKDIVARKIRIYTPA
APSAVVIWDPAIEREDKAEAQGTILKIYGPNLQDINCFTDIGAEPKIESLKAWDEKEKYVVHRDDIPEDI-
TLVGKSSKVNKEFAIPDVAVCAPSKPGVTESIDRGEPKAGTCGEVANMSYPQESLVNTVSSANVQKNI
SMVLSGHPVGEMGISREIERHLETSMCEIFVDKDDVDVALDFYILTLTPLRVGLDPPNARVQIDGKESR
KLMAVQDGDADLQRHPKVSQATGTGLKAGVKARMPDDALIVDGVMHGDVSFTATDLPDGRV-
VTPVFNEVKAQLKIDKVLLKAFHVVTLSFSNDKGEEHLIKLRLNALKKSTRHYEMLLKIRETECAESPHAI
SVGKVLKIDALMQTICEAHYKEIEPLLADELTALGVMGEQGLLHIVPRLMILAVLHLHQLEIAEQLHEW
GLQAAAIVTDQTMGLYCRDRAWSF-AISEVVGNTFFARTKESDTEQKIRARNPV-VLL-LGKFNMI-
ISPSGLGLASTAESPQGRGNTEELILSAERLQDEMGYYVLRILLVFVDEVILAYGSGDDPIKFLVAEQAVT
GRQNPGMVTLTR-
TFPLELQRNATFVMVFEDCTMKNLSHKKMHMNLVVIKSKGWIVNVSTKILISDQAIASYAEDAGAHT
QPSESALAVITSRVMAPIVLVVSQKPTAPSKTLFLEALSLYDEVTATVQNKPKKYYIFEGDVVAVLGGLD
AAVLLGMQRPQPDRMRRAIVAGAVRLAIAIRASGVVTRLEENLINFSRAVNFAALFYQIGNLV------

----EFRVRNHDVSTKRSRPYKAHPKFLHKADVDKSLIATGVDTLA
>GCA_002933395
AVIKPVTMNKYAPVLMIRQQQVATVSGAGSPSLRAKTVYRKSGLQRKAETKWVSKDFYY-
GVVKMGNLVQLVSISVDNRAVCTVAQLVDQEVVVHGMVALDAVKKKIALEAIEGMTAEDPAKVVD
Y-GEIELKKFRDADDRADMLPVMLGLTLKIKFDPHQPVVHDLAIPVVFEVV-
GIMVQKGTMEVDDCGVSTALSDVDPIVYVSPIDLAAVAGDAMAYIDQEAAVSAAKASIDLD-
LALESNVLVVGI-PLLIPV-FEADGDT-MSFANRSRTLKTIVNLAVSYPEIKRA-ID-NIAAAM-
MDASQAAALSEKKGTAEEIRIVKRMQRSAGLADDPDLVMLEDGAEGNSNWAGQIYDDPLSGSSESD
SAGNIVFAANKRQNFARSITYLRFVEISDKAAAPLMRGVKHNAKSRASRKVDDAIAVLNFPNVPEGIAI
RAEAEKLEDMLLHDAAEGEQILLIVVRWHVTWKTAIIVGLFPVADDVVGLIAIGGIGAVGRKDGFLNR
HHKLNGSLIEQRRIVEKLTLRLRAALKQKLGQDQIIIFVVKVAGDLVASATVQM---
AVGAVVAIQGPTIFVRRSRQTVIGDQEATVEILQGVAEGPADAEALLRIRVDHTVVKLSVLLIPEVPVLV
ESVTIREGVGKALQIIQLDVYLLRDMGTGEETLAKSKS-----------------------------------
------
VSILKNVTEPRMENLPEGICRTFVNAGPRAAVGMLFLLLDDAGKIYTIDGDLRENTKSIKSLIGLVDAAK
PKILSAGYATTRLKIRYADVHVLISDADLYAYAEERRELLRMVPVYIINAAKRRQLILFALSLKGGHRRAR
AALGVMIAAAILESQAEQGAVNHEEPAVLLDDGEIVGSEMMISRQAALELKQTFKIDIIVIFPEFQEKM
VVSLKFFCTDVAIFVIALICTVGCA_LSREPSHAQEGGAGFQKRIRGHSVLFALTVTFDRRNLRSSDA-
ALFLGLGLAPPRYGIFYTEQPKRKQMPSEADKEEVDGFDLVNAIKEDTTS-EELDMPEAMALAVE-
DASINVNAGVLALNGDMAAVEKVDSKRKMALAVEKWTISPIPLVDIKISGAQPFRALD-
IEQADVSASLPKKEIEELFLPGANNAELEEEKLDGAGASTAEVAEDKQSAILLVNPVLVVIQDSNSAALI
ANGGLEEWGTMAYKKHYALAARMRLHMRTID----
GVAMDPNKQKAEKIKTVKLEGDTRFRQLATEGVKEQMP-
LHALNPDFAVLIPVLVTPAVGRVAADAGVLGAEVPCDASL-
RELQKSQKGAALPISGIRVLGMRAARAMVDEDIMVAGKVAA-
LLVILALETITALVLMNDSEATLPWRLSVAERLEADTKRSGGFASRPFKSKPSLCKTQAHAKIADPIQEIA
AGYVATEERFVFPVEDIPIQLILASTHSRVGSAKGAGTGHLHEMAKELQAKAVIMVQNQPATNTDGLL
NYLTTQQPTDLLFEDCLDVVIDMRRMSVV---
VHLNTV--
VVVEGVSPVGSYEIMAAAAHSGLVQVERLDERTGRERTLMWMYGAMEELWLMSVTYADWVIDCAI
GAILLHVYAQKINRAFSLERLQLAGWPPEATRWWNTWQLWLNKDGFFSYEAAFRA-
KAVLKSDLDDYDMSDVEGVGRYQSRQELRVGYRIREQVAFEQLDPRTDILLQSLTRLSVIRHALKMVA
ILIVREIVVEFT-
VVAQEDVAMGHVECVLADSFDFTAKNADIGPVGLRTPDPVLGDGTVLKEAVLGKIDLANTVADFRYK
MLIDGGRELGVSWVTLKNMLYARIWLPPAEAALKNLVPICFFLGHTELNLNIFPALWAGISKARANKL
MAGALIEKILEKVAAFADPEATTHQSKKLRHFKYYGEEVDPVYLAYIFDTTALVSTQVTVSDRLLKDRYV
LNLKVFRGFTSLLWLSAQIGDKGPSATIITVQTIAGLGALVLFIILLMRAVVLVETLYYKYVY--
MLGYQAAKDVADADPEHFLALSHASRDVEWIEVVPAFFYYFNTAKKNLTKNTALAKEFFGLFEERLRV
VDVLVVPPDRVEALVEFVALAGTLVTSAQALVGVIACGFCEPNAGAVLEADQDWNLHRSMGITLAFY
KAQVSVKKWFDKGLHKDVAEIKLNEAGALTTPFHAEVTAKKCVPAVHRVLDLGEVEAALIEIDSAVVV
ADEEQTLADKPTTRENSVGAVGGIVLAGTMNMGANLDSVERGGNIIIELEEDNYSAVNGKKNDGKLT
DKVLMVLSQAYFPVLKRMASANALDRGAFGLLIDAPDDADNKDHGRTLTIPAPTVHAESAPLFAESDI
ERIAWIAPIHLLGTLDAVLGFEPDQYSFISMMIEASLDIMQTAEVLDDYHVLEKGSRPPSFRVILIKGLHIR
LLAGVCTIIVVGSAGRAEVTAYIQD-

AVPITLNKHTFLPAFAASSCHVIDIFSTMLRPPERSTIEMLILETVAEL-
LEVTRIENDKLERMNALLPGLDVAALLDLLAAAKDFADIVTIDAAIAAILSVAAEAVVLREMLLTLAEIAV
TALALAMALLLMLTRDQGLHQREVAREAQLKELIQGGTVLQVKASDRMMLASTADKVRLDTDEGTT
SAFASLNLATVSDGFYVMSSSVLQMSSDLMMIVAVARVLADKETKNALVVRHPEQVKTQSTYAEGS
PGMVLQTLTGAIPPLAIKPEDVELLWTLVAALWRVKAHLANAARPHNERVLILILALSLISVLLQSEGGG
MGGG-
AGPAAKILAAFSILTIKAKAGEEVRKVTRRVMDLNSRLGRPEEAVNPEFRGAELEQTKYEAESVEVDTM
ETIRRFVDIPFLVSTRTQHNESEDRGARMVLDVTRVRFIDL-
VTKRSEIILAARVETDGIIAREMGKDLAALHPEAAALGKIAALQHFIAVQDAHISNYVTAEIHAVLGMLL
LVLFFQEVRVSKVVWPTRREVTMVFILASIAIFFLVI----
QIVFMLSGISWNQLTTIVLGEGIMVVRQTSTFPADS-
IGILQNTYADLICAGRVAKADIEKSLHIVVAGRVARGTWYVIYLYDISQAQGQSGIMQRAFRQMTLSK
SLIDGRMEVKIKVVTSDRYA-
TYEIKGGQHRKGTRYNRVKVGHNIPVQVLPRMKMAFTPPNLVLVKTFGAVVQKRFAQALTHIRHGA
VTIGPMEVSSPYISNKKARHAPGDSVVDLKPENIVRSVAVVPELIWARENEDNGAVHDKYVKTTHKLA
AKRKKAVDKAQYDHRNQVELSFIGLAVKVLPDKAIVHATSGELMPTASVAVTDKMKGIAATIRLDPM
EKSLIAYIKRMRPQLNLAGQTDKKIVKCLNNNLDDVEYGKLGRRKIKPITIKSVYDVEHKRATELVKLMP
YKDDVESRKSQFAEK--
ALFEPLKAFMMLFGFIARSVYALYLRVIRRTILLVKTKIVVNAATVLSAIKIIGVSIRLRHKSTNVCETNHHI
KTIEARARMTLAGTGKVKLNLHEGKIGMKGGRIKGFMPYKNFNIFDGLASGRRAKVAELAKLKDVDKTI
SVDGVRVKQQGGRTPKAQLSNAI-
NTRVGVDIVGENEQEESNPRANAEMGQIFAKIDARVFSHVGALVGHALEAVLAVVSIWTIGRAHNLL
AVRLKVNAGIEMHSAA-VEAAVAAA-FEQAAGEA-
AIADVSEGDLGALALSTMSVDVCRLRRRSLSELKKTKKDAYMSLTCFGGKGDKVKIVEATQTQAYEIVA
TRTAERHGIRAKGTYGKERAMMQITPTKPVKIWVPVFVNEDELLAPIRAFAETQGDGVTVVKLTKDVK
VTMFEKAVASLAVSGKATSVVYTVLEKVTQLMKDIIYTQRAKAHYGRGRLRSHVVTTTKQRRHLSMM
KRTVIADPSMKAARLTDGEVTVITGATMAFVMSEQDAV--
GDVDLHYHEEPRREAEVGIGGPETFLTTEQPRVLALIYLRAYLIHIVEIETTNGFADAATIYDVLRAMQN
KLDRKLSADIAKTRMVLTDNVTDMGVTDVAPRRNAAGDKILSAFWINGVTANADIILLVANLECPCV
PENIDCAIRLFYQRITLQAKERDGDVSVAVVPAANQIEAAEGTVIYSIKGNTPGTGQMKLTIEGISRSVV
KSFAVQLFQVDTFLNDTWIATWLRAAKQVVFVASEDGKEAVDHVVERDTELEFAPQKDIVARKIRIFT
PAAPSAVVIWDPAIDREDKAEAQGTILKIYGPNLQDINCFTDIGAEPKIESLKAWDEKEKYVVHRDDIPE
DITTLVGKSSKVNKEFGIPDVAVCAPSKPGVTESIDRGEPKAGTCGEVANMAYPQESLVNTVSSANVQ
KNISMVLTGHPVGEMGISREIERHLETNMCEIFVDKDDVDVALDFHILALTPLRVGLDQPNARVQIDG
KESRKLMAVQDGDAKLQRHPKVSAATGLTQKAGTKARMPDDALILDGVMHGDVSFTATDLPEGRV
-
VTPVFNDVKAQLKIDKVILRGFHVVTLSFSNDKGEEHLIKLRLNALKKSTRHYEMLLKIRETECAESPHAI
SVGKVLKIDKLLQTICEAHYKAIEPLLADDLTALGVMGQQGLLHVVPRLMILAVMHMMRLELAQQIP
DWRLQADAVLTDQTLGLSIRAREWSF-AISEVVGNTFFARTKDDDTEQRIHARNPV-VLL-
LGKFAMI-
ISPSGLGLKSTEESPQERGNAEELILSTERLQDEMGYYVLRILLVFVDMVILAYGSGDEPIKFLVAEQAVT
GRQNPGMVTLTRRTFPLELQRNATFVLVLEDSTMKNLSHKKMHMNLVIIKSKGWLVNVSTKILISDQ
AIASYAEDAGAHTQPSESALAAITSRVMAPIVLVVSQKPTAPSKTLFLEALSLYDEVTSTVLNKPKKYYIF
EGDVVSVLGKLNAAVILGMQRPQPDRMRRAVVAQAIRLAIAIMASGIITRLEEGIINFSRAVNFAILIYTS

LETWTAE-------EFRVRNHDVSTKRSRPYKAHTKFLHKADVDKSLIATGVDTLA
>GCA_003003285
AVIKPVTMNKYAPVLMIRQQQVATVSGAGSPTLRAKTVYRKSGLQRKAETKWVSKDFYY-
GVVKMGNLVQLVSISVDNRAVCTVAQLVDQEVVVHGMVALDAVKKKIALEEIEGMTAEDPAKVVDY
-GEIELKKFRDADDRADMLPVMLGLTLKIKFDPHQLVVHDLAIPVVFEVV-
GVMVQKGTMEVDDCGVSTALSDVDPIVYVSPIDLAAVAGDAMAYIDQEAAVAAAKASIELD-
LALESNVLVVGI-PLLIPV-FEADADT-MSFANRSRTLKTIVNLAVSYPEIKRA-----IAAAM-
MDASQAAALSEKKGTAEEIRIVKRMQRSAGLADDPDLVMLEDGAEGNSNWAGQIYDDPLSGSSESD
SAGNIVFAANKRQNFARSITYLRFVEISDKAAAPLMRGVKHNAKSRASRKVDDAISSLNFPNVPEGIAI
RAEAEKLEDMLLHDAAEGEQILLIVVRWHVTWKTAIIVGLFPTADDVVGLIAIGGIGAVGRKDGFLNRH
HKLNGSLIEQRRIVEKLTLRLRAALKQKLGQDQIIIFVVKVAGDLVAAATVQM---
SVGAVVAIQGPTIFVRRSRQTVIGDQEATVEILQGVAEGPADAEALLRIRVDHTVVKLSVLLIPEVPVLV
ESVTIREGVGKALQIIQLDVYLLRDMGTGEETLAKSKSREQFREVLQVDPITGVRLAFGQLRERLVKGGT
MKELGTLL--
VSILKNVTEPRMENLPEGVCRTFVNAGPRAAVGMLFLLLDDAGKIYTIDGDLRENTKSIKSLIGLVDAAK
PKILSAAYATTRLKIRYADVHVLISDADLYAYAEERRELLRMVPVYIINAAKRRQLILFALSLKGGHRRAR
AALGVMIAAAILESQAEQGAVNHEEPKVLLDDGEIVGSEMMISRQAALELKQTFKIDIIVIFPEFQEKMV
VSLKFFCTDVAIFVIALICTVGCA_LSREPSHAQEGGAGFQKRIRGHSVLFALSVTFDRRNLRSD-A-
ALFLGLGLAPPRYGVFYTEQPKRKQMPSEADKEEVDGFDLVNAIKEDATS-EELDMPEAMALAVE-
DASINVNAGVLALNGDMAAVEKVDSKRKMALAVEKWTISPIPLVDIKISGAQPFRALG-
IEQADVSASLPNKEI-
FLFLPGANNAELEEEKLDGAGSSTAEAAEDKQSAILLVNPVLVVIQDSNSAALIANGGLEEWGTMAYK
KHYALAARMRLHMRTID----GVAMDPNKQKAEKIKTVKLEGDTRFRQLATEGVKEQMP-
LHALNPDFAVLVPVLVTPAVGRVAADAGVLGAEVPCDASL-
RELQKSQKGSALPISGIRVLGMRAARAMVDEDIMVAGKVAA-
LLVILALETITAIVLMNDSEATLPWRLSVAERLEADTKRSGGFASRPFKSKPSLCKTQAHAKIADPIQEIA
AGYVATEERFVFPVEDIPIQLILASTHSRVGTAKRAGTGHLHEMAKELQAKAVIMVQNQPATNTDGLL
NYLTTQQPTDLLFEDCLDVVIDMRRMSVV---
VHLNTV--
VVVEGVSPVGSYEIMAAAAHSGLVQVERLDERTGRERTLMWMYGAMEELWLMSVTYADWIIDCTIG
AILLHVYAQKINRAFSLERLQLAGWPPEATRWWNTWQLWLNKDGFFSYEAAFRA-
KAVLKSDLDDYDMSDVEGVGRYQSRQELRVGYRIREQVAFEQLDPRTDILLQSLTRLSVIRHALKMVA
ILIVREIVVEFT-
VVAQEDVAMGHVECVLADSFDFTAKNADIGPVGLRTPDPVLGDGTVLKEAVLGKIDLANTVTDFRYK
MLIDGGRELGVSWVTLQNMLYARIWLPPAEAALKNLVPICFFLGHTELNLNIFPALWAGISKARANKL
MAGALIEKILEKVAAFADPEATTHQSKKLRHFKYYGEEVDPVYLAYIFDTTALVSTQVTVSDRLLKDRYV
LNLKVFRGFTSLLWLSAQIGDKGPSATIITVRTIAGLGALVLFIILLMRAVVLVETLYYKYVY--
MLGYQAAKDVADADPEHFLSLSHASRDVEWIEVVPAFFYYFKTAKKNLTKNTALAKEFFGLFEERLRV
VDVLVVPPDRVEALVDFVALAGTLATSAQALVGVIACGFCEPNAAAVLEADQDWNLHRSMGITLAF
YKAQVSVKKWFDKGLHKDVAEIKLNEAGALTTPFHAGATAKKCVPAVHRVLDLGEVEAALIEIDSAVV
VADEEQTLADKPTTRENSVGAVGGIVLAGTMNMGANLDSVERGGNIIIELEEDNYSAVNGKKNDGKL
TDKVLMVLSQAYFPVLKRMASANALDRGAFGLLIEAPDDADNKDHGRTLTIPAPTVHAESAPLFAES
DIERIAWIAPIHLLGTLDAVLGFEPDQYSFISMMIEASLDIMLTAEVLDDYHVLEKGSRPPAFRVILIKGLH
IRLLAGVCTIIVVGSAGRAEVTAYIQD-

AVPITLNKHTFLPAFAASSCHVIDIFSTMLRPPERSTIEMLILETVAEL-
LEVTRIENDKLERMNALLPGLDVAALLDLLAAAKDFADIVTIDAAIAAILSVAAEAVVLREMLLTLAEIAV
TALALAMALLLMLTRDQGLHQREVGREAQLKELIQGGTVLQVKASDRMMLASTADKVRLDTDEGTT
SAFASLNLATVSDGFYVMSSSVLQMSSDLMMIVAVARVLADKETKNALVVRHPEQVKTQSTYAEGS
PGMVLQTLTGAIPPLAIKPEDVELLWTLVAALWRVKAHLANAARPHNERVLILILALSLISVLLQSEGGG
MGGG--
GPAAKILAAFSILTIKAKAGEEVRKVTRRVMDLNSRLGRPEEAVNPEFRGAELEQTKYEAESVEVDTMET
IRRFVDIPFLVSTRTQHNESEDRGARMVLDVTRVRFIDL-
VTKRSEIILAARVETDGIIAREMGKDLAALHPEAAALGKIAALQHFIAVQDAHISNYVTAEIHAVLGMFL
LVLFFQEVRVSKVVWPTRREVTMVFILASIAIFFLVIGL--
QIVFMLSGISWNQLTTIVLGEGIMVVRQTSTFPADS-
IGILQNTYADLICAGRVAKADIEKSLHIVVAGRVARGTWYVIYLYDISQAQGQSGIMQRAFRQMTLSK
SLIDGRMEVKIKVVTSDRYA-
TYEIKGGQHRKGTRYNRVKVGHNIPVQVLPRIKMAFTPPNLVLVKTFGAVVQKRFAQALTHIRHGAV
TIGPMEVSSPYISNKKARHAPGDAVVDLKPENIVRSVAVVPELIWARENEDNGAVHDKYVKTTHKLA
AKRKKAVDKAQYDHRNQVELSFIGLAVKVLPEKA---------------------------------------
---
RMRPQLNLAGQTDKKIVKCLNNNLDDVEYGKLGRRKIKPITIKSVYDVEHKRATELVKLMPYKDDVES
RKSQFAEK--
ALFEPLKAFMMLFGFIARSVYALYLRVIRRTILLVKTKIVVNAATVLSAIKIIGVSIRLRHKSTNVCETNHHI
KTIEKRARMTLAGTGKVKLNLHEGKIGMKGGRIKGFMPYKNFNIFDGLASGRRAKVAELAKLKDVDKTI
SVDGVRVKQQGGRTPKAQLSNAI-
NTRVGVDIVGENEQEESNPRANAEMGQIFAKIDARVFSHVGALVGHALEAVLAVVSIWTIGRAHNLL
AVRLKVNAGIEMHSAA-VEVAVAAA-FAQAAGEA-
AIADVSEGDLGALALSTMSVDVCRLRRRALSE--
RHGIRAKGTYGKERAMMQITPTKPVKIWVPVFVNEDELLAPIRAFAETQGDGVTVVKLTKDVKVTMFE
KAVASLAVTGKATSVVYTVLEKVTQLMKDIIYTQRAKAHYGRGRLRSHVVTTTKQRRHLSMMKRTVIA
NPSMKAARLTDGEVTVITGATMAFVMSEQDAV--
GDVDLHYHEEPRREAEVGIGGPETFLTTEQPRVLALIYLRAYLIHIVEIETTNGFADAATIYDVLRAMQN
KLDRKLSADIAKTRMVLTDNVTDMGVTDVAARRNAAGDKILSAFWINGVTANADIILLVANLECPCV
PENIDCAIRLFYQRITLQAKERDGDVSVAIVPAANQIEAAEGTVIYTIKGNTPGTAQMKLTIEGISRSVVK
SFAVQLFQVDTFLNDTWIATWLRAAKQVVFVASEDGKEAVDHVVERDTELEFAPQKDIVARKIRIFTP
AAPSAVVIWDPAIDREDKAEAQGTILKIYGPNLQDINCFTDIGAEPKIESLKAWDEKEKYVVHRDDIPE
DITTLVGKSSKVNKEFGIPDVAVCAPSKPGVTESIDRGEPKAGTCGEVANMAYPQESLVNTVSSANVQ
KNISMVLTGHPVGEMGISREIERHLETNMCEIFVDKDDVDVALDFHILALTPLRVGLDQPNARVQIDG
KESRKLMAVQDGDAKLQRHPKVSAATGTGLKAGTKARMPDDALILDGVMHGDVSFTATDLPEGRV
-
VTPVFNDVKAQLKIDKVILRGFHVVTLSFSNEKGEEHLIKLRLNALKKSTRHYEMLLKIRETECAESPHAIS
VGKVLKIDKLLQTICEAHYKAIEPLLADDLTALGVMGQQGLLHVVPRLMILAVMHMMGLELVQQIPD
WRLQADAVLTDQTLGLSMRAREWSF-AISEVVGNTFFARTKDDDTEQRIHARNPV-VLL-
LGKFAMI-
ISPSGLGLKSTEESPQDRGNAEELILSTERLQDEMGYYILRILLVFVDMVILAYGSGDEPIKFLVAEQAVT
GRQNPGMVTLTRRTFPLELQRNATFVLVLEDCTMKNLSHKKMHMNLVIIKSKGWLVNVSTKILISDQ
AIASYAEDAGAHTQPSESALAAITSRVMAPIVLVVSQKPTAPSKTLFLEALSLYDEVTSTVLNKPKKYYIF

EGDVVSVMGTLDAAVILGMQRPQPDRMRRAVVAQAIRLAIAIMASGIITRLEEGHINFSRSVSLATGL
YAMIELHPLAIIT-DTVEFRVRNHDVSTKRSRPYKAHTKFLHKADVDKSLIATGVDTLA
>GCA_003058085
AVIKPVTMNTYAPVLMIAQSQVATVSGAGSPTLRAKTVYAKSGLQRKAETKWVSKDFYY-
GVVKLGNIVQLVSISVDKRAVCTVAQLVDQEVVVRGMVALDAVKKKIAVEAIEGMTAEDPAKVVDYR
AELELRKFRDADDRADMLPVLLGLTLKIKFDPHQPVVHDLPIPVVFEVV-
GIMVQRGQIEVDECAISTAISDVDPIVYVSPIDLAAVAGEKMAYIDQESAVAAAKAEIELEELALESSVLV
VGI-PLLIPV-FAADGDV-MSFANRSRELKTIVNLAVSYPAIKRA-IDN--GSAM-
MEASQAAALAEKKGTAEEIRIVKRMQRSSGLADDPDLVMLEDAAEGNSNWAGQIYDDPLSGSSESD
SAGNIVFAANKRQNFARSITYLRAVEISDAADAPLMRGVKHNSKSRAARKLNDAIAALNFPNVPEGIA
IKAAAGKSTDLLLHDATEAEEILMIIVRWHVTWKTAIIISLFA-
AGNIVGLIELGGIGAVGRKEGLLNRHHKLNGSLIEQRRVVEKLTLRLRAALKQKLGAEQIIIFVVKVTGDL
VAKAKIQM---
SVGAVVAIQGETVFKRRGRQTVIADQEATVEILKGVAEGPADAQALLRIRVDHTVVKLSVLLIPE-
PVLVEAVTIREGVGKALQIIQLDVYLLRDMGTGDETLAKSKSREQFREVLQVDDITGVRLAFGQLKERL
VKGGSMKELGTLL--
IAIIRGITEPRMANLPAGVCRTFIAAGPRAAVGMIFLLLDDAGKIYTIDGDLRENTKSIKSMIGIVDAAKPK
ALSAGYATDRLKVRYADVTVLTSDADLYAYAEERRELLRMVPVYITNAAKRRQLIIFALSLRGGHRRAR
AALNVLIAAAILESQAERGQVNHAAPVVLLDDGEIVGSEMMISRQASLDLKQILKIDIIVIFPEFQEKMV
VSLKFFCTDVAIFVIALICTVGEMLSREPSHAQEAGAGFGKRIRSHSVLFALAVTFDRKNLGDA---
DLFLNLGLAPPRYGIVYTEQPKRKQMPSEAAEEEIDGFDLVNAIKEDTSSNHELDMPEAMALAVE-
DSTINVSAGVLAINGDMAAVEKVDSKRKMALAVEKWTISPLPISDLKISGAAKFRVLG-
IEQANVSATMPKKTVWFLYLPGANNAEMEEAKLDGAGSSTADAAEDKQLGILLVNPVLVVIQDSNT
ASLIANGGLEEWGTMAYKKHYAFAARMRLHMRTID------
VMDPNKQKAEKIKTVKLEGDTRFRQLATEGVKEQMP-
LHALNPDFAVLVPILVTPAVGRVAADAECLGARVPCDASL-
RELQKSQKGNALPISGIRLLGMRAARAMVDEDIMVAGKVAA-
LLVISAISTITAIVLMNDSEATLPWRLSVAERLEADTKRGGGFASRPFKSKPSLCKTQAHARIADPIQEIA
AGYVASEERFVFPVEDIPIQLILASTHSRVGAAKRAGLGHLHEMAKELQAKAVIMVLNEPGTNTEGLLN
YLTTQQPTDLLFEDCLDVVIDMRRMNVV--
VQLNTV-
HVLVPGVSDLGEREIMAAAAHSGLVQVEQMDDRTGREKTLMYMYGAMEELWLRSLTWGDWVIDC
TIGAILLHVYAQQINRAFSLERLQLAGWPPEATRWWNTWQLWLNKDGFFGYEAAFRAMKAVLKAD
LADYDMSDVEGVGRYQSRQEIRVGYRIRQQVAFEQLDPRTDILLQSLTRLSVIRHALTMVAILIVREVV
VEFTLVVAQEDVAMGHVECVLADAFDFTAKDADIGPVGIRTPDPVLGDGTVLKDAVLGKIDLANTVA
DFRYKMLIDGGRELGVSWLSLQNMLYKRIWLPPAQAALKNLEPITFFLGHPELNLHIFPALYAGISKAR
ADKLMAGALIEKILEQIAAFADPEATTHQSKKLRHFKYYGEEVDPVYLAYILDMFALVATQVTVSDRLL
KDRYVLSLKVFRGFTSLLWLSAQIGEKGPAATIITVMTIAGLGALVLFIILL-RAVVLVETLYYKYVY--
MLGYQAAKDVADADPEHFLSLAHASRDVEWIEIIPAFFYYFKTATKNLEKNTALAREFFGLFKERL---
EVLVVPPDRVEALAQFVALAGTLLTSARA-PGIIACG-
CEPNAAAVLEADQDWNLHRSMGITLAFYDARESVKGWFDKGLHKEVAETGLSEAGALTSPFHAGST
AKRCVPAIHRVLDLGEVEAALIEIDSAVVVADEEQTLADKPTTRENSVGAVGGIVLAGTMNMGANLD
AVERGGNIIIELEEEHYSAVNGKKADGKVTDKVLMVLSAAYFPVLRRMASANALDKGTFRLLIDAPDD
ADNQDHGGGLTIPAPSVHAETAPLYAESEIERIAWIAPIHLLGTLDAVLGFEPDQYSFISMMIDASLDIM

ETAEV--DYHVLEKGARPPGFRVILIKGLHIRLLAGICTIVIVGSAGRSEITAYIQG-
AVPITLNKQTFLPAFAASSCHAIDIFSTMLRPPERSAIEMLILETVAEL-
LEVTRIENDKLERMDALIPGLDVASLLDLVSKSSDFADIVTIDRAIAAILSVAAEAVVLREMLLTLAEIAVT
ALALAMALLLMLTRDQGLHQKETARETQLKELIQGGVVLQVKAADRMMLASTADKVRLDTDEGTTS
AFASLNLATVSDGFYVLSSAVLQLSSDLMMIVAVARVLADKETKNALVVRHPEQVKTQGTYAEGSPG
MVLQTLTAAIPPLAIKPEDVELLWTLVAALWQVKAHLANAARPHMERVLILILALSLITVLLQSEGGGIG
GG--
GPPAKMLAAFSILTIKAKAGEEVRKVTRRVMDLNSRLGRPEEAVNPEFRGAELEQSKYEAENVEVDTM
ETIRRFVDIPFLVSTRTQHNESEDRGARMVLDVTRVRFIDL-
VTKRSEIILAARAETDGIIAREMGKDLAALHPEAAALGKIAALQHFIAIQDAHISNYVTAQIHAVLGMILL
VLFIQETRISKVVWPTRREVTMVFIMAALAVFFLVIGLAVQIIFMLSGISWNALTTIVLGEGIMVVRQTST
YPQDS-
IGILQNTYADLICSGRVAKADIENSLHIVVAGRVARGTWYVIYLYEITQAQSQSGIMQRAFRQMTLSQ
RLIDGRMEVKIKVVTSDRYA-
TYEIKGGQHRKGTRYNRVKIAHNIPVQVLPRMKMAFGPPNLVLVKTFGAVVQKRFAQALTHIRHGAV
TIGPMEVSSPYISNKKARHAPGDAVVDLKPDNIVRSVAVVPELIWARENEDNGAVHDKYVKTTHKLA
AKRKKAVDKAQYDHRNQVELSFIGLAVKVLPDKA--------------------------------------
---
RMRPQLNLGGANDKKIVKCLNNNLDDVEYGKLGRRKIKPITIKSVYDVEHRRATELVKLMPYKDDVES
RKSQFAEK--
ALFEPLKAFMMLFGFIGRAIYALYLRVIRRTILLVKTKIVVNASTVLSAIKIIGVSIRLRHKSTNVAETNHHIK
TIEARARMTLAGTGKVKLNLSPGKIGMKGGRIKGYMPYNNFNIFDGLASGRRAKVKELAKLKEVDKTIS
VEGIRQKQQGGRTPKANLSNAI-
NSRVGVDIVGENEQEESNPRANAEMGQIFAKIDARVFHHVGALIGHALTAVLAVVAIWGIGRAHNLL
ADKLKVNAGIEMHSAA-VEMAVAAA-FEENGGEA-IVVDISEAYLDALAITAMSVDVCRLRRRGLA--
----
RHGIRAKGSYGKERAMMQITPTKPVKIWVPVFVNETELLAPIRAFAESTGDGVTVVKLTTDVKVTMFE
KAVASIAVTGKATSIVYTVLEKVTELMKDIIYTQRAKAHYGRGRLRSHVVTTTKQRRHLSMIKRTVVAD
PSMDAARLTDGEVSVITGATMAFVMSEQDAV--
GDVDLHFHDEPQREADVGIGGPETFLTTEQPRVLALIFLRAYLIHIVEIGTVGGFSDAATIYDVLRAMQ
NKLDRKLSADIAKTRMCLTDNIADMGVTEVAAKRDAAGDKILSAFWINGVTANADIILLVANLECPCV
PENINCAIRLFYQRITLQSKERNGDVSVSIVPAANQIEAAEGTVIYSIKGKEPGTGQMKLTIEGISRSIVKS
LAVQLFQVDTFLDDTWIATWLRAAGQVVFMHADDGKEAVDYVVERDTELEFAPQKDIVARKIRIYTP
AAPSAVVIWDPAIEREDKAEAQGTILKIYGPNLQDINCFTDIGAEPKIESLKAWDEKEKYVVHRDDIPED
I-
TLVGKSSKVNKEFAIPDVAVCAPSKPGVVESIDRGEPKAGTCGEVANMAYPQESLVNAVSSANVQKN
ISMVLAGHPVGEMGISREIERHLETNMCEIFVDKPDVDVALDFSTLTLTPLRVGLDPPNARVQIDGKES
RKLMAVLDGEADLQRHPKVSQATGLTHNAGVKARLPDDSLILDGVMHGDISFTITDLPEGRV-
VTPVFNAVKAQLKIDKVLLKAFHVVTLSFSNDKGQEHLIKLRLNALKKSTRHYEMLLKIRETECAESPHA
ISVGKVLKIDALLQTICEAHYKEIEPLLADDLTALGVMGQQGLLHIVPRLMILAVLHVLGLAAAFQVVR
W-LQQVAIVTTQTLGLRHRTRDWSFIAISEVVGNTFFARTKDDDTEQRIHARNPV-VLL-YGKFPMI-
ISPSGLGLQSTAPSAQDRENTEELILSKERLQDEMGYYVLRILLVFVDEVILAYGSGNDPIKFLVAEQAVT
GRQNPGMVTLTRRTFPLELQRNATFVMVFEDCTMKNLSHKKMHMNLVVIKSKGWIVNVSTKILISD
QAIASYAEDAGAHTQPSESALAVITSRVMAPIVLVVSQKPTAPSKTLFLEALSLYDEVTATVLNKPKKYY

IFEGDVVGKLGLIKGRLLKGVLLVMPNAFRLACVAKAVRMAIAW---------------------------
---------------DFRVRNHDVSTKRSRPYKAHPKFLHKADIDKGLIASGVDTLA
>GCA_003072065
AVIKPVVQDEYAPVLMIRQDQVATVSGAGSPSLRAKTVYAKSGLQRKTATKWVAERFYY-
GVVKASNIVQLVSISVDNRSVCTVAQLVEQEVVVHGLVALEAVKKKIALEAIDGMTTEDPAKVAQF--
--IPTVFEVV-
GIMAQKGTIEIDECGLSTALSDVDPINYVSPIDLAAVAGEQMSFIDQETAVANAREAIELEELALEASILV
VGIKPLLVPV-FHEEG-T-MSFANRSRELKSIVDLAVGYPEITRARIE-DFVAEM-
MERAKAAALAEKRGTAEEIRIVKRMQKSSGLADNPDLVMLEDAAEGNAAWAGQIYDDPISGSSESDS
VGNIVYAANKRQNFQRSITFIRRAEISDKAAAPLMRGVKHNAKSRVARKLDDAIAALNFPNVPEGIAIR
AEAGKPEATLLHDAAEGEEILLLIVRWHVTWKTAIFVLLLPDAGNVVGLIDLGGIAAVGKMEGLLNRH
HKLNGSLIEQRPIIEKMTLRLRASLKQKLGKDQVIIFVVRVAGDLVAEATVQM---
NVGLVVAIQGETIFVRRHKQTVITDQEATV-VLQGVAEGPADAAALLRIRVDHTVVQLSVLLIPE-
PVLVESVTIREGVGKALQIIQLDVYLLRDMGTGEQTLAKSKSREVIREILQVDPITGVRLAFGKIKEQLSK
GMKLKDLD-----
LALLVGVTEPRMENLPQGVCRNFIAAGPRAAVGMLFLLLDDAGRIYTIDGDLRELSKSIKSLIGLVDAAT
PKILSAGYATTRLKIRYADCTVLTSDADLYAYAEERKELMRMVPVYIIKAAKRRQLVIFALSLKGGHRRTR
AALEVLLPATILESQAEEGSVDHEDPQVLLDDAEVVGSEMMISRQSALELKRILKVDVIVVFPEFQEKM
VVSLRFFCTEVAIFVIALITTVGEMLSREPEHAQEEGAGFQKRIRGHSVLFALVVTFDRRNARCA-
APNLFLALGLAP-
RYGILYTNQPKKKQMPSEPDDDRIDGFELVHAIHNDGRDNHELEFGEAMALAVEADATINVSAGVLA
MTGGMVATEKVDSKRKVALAATRWTISNASIEDLRISGADELRCLG-IEVADVSAALNEKSI-
FLFIPGANNAELEEAKLDGAGSSTAEAAEDRQLDILIIKPVLVTIQDSNTASLIANDGVEEWGTMAYKK
HYAYAARMRLHMRTID------ALEPNKQKAEKVKTVRLEGDTRFRQLATEGVKEQMP-
LHALNPEFAV-RPLLVTPAVGQVAAEEDVLGARVPCDFSL-
RELQKSRKGNALPISAMRLLGMRAARAMIEEGIMVAGKVAA-
LLKIAALETIVHVVLRNDSEATLPWRLSVAERLEADTKRGGGFASRPFMSKPSLCDTQAHANVADPIET
IAAGYVAVEDRFVFPVEGIAIQLILAGPHSRVGGDKRAGEGHLHEMAKELKAKAVLMVKGEPENDTEG
LLNYHTTNQPTDLLLTDCLDILIDMRRMTIV--
VQLNTV-
HVVVAGVRPVGSYQIMAAAAHSGLIQVEQIDDLTGRERTLMWMYGAMEELWLLSLRYADWVINTPI
GAIILHVYAQAINRAFSLTRLQIEGWPPEADRWWNTWQLWLNKDGFFSYEAAFRAVKAVLKAELADF
EMSDTEGVGRYLSRQEIRAGYRIRQQVAFARLDPRTDILLQSKTRFAVIRHALNMVAILVVREVAPEFT
-
VVAMEDVAMGHVECVLAECFDFTAKNADIGPVGLKTPDTIFGDGTVLKDAVLGKIDLKNTVQDFRYR
FIIDGGRELGVEWVSLTNMLYARIWLPPAQAKLKDLLPIAFFLSHPELDMDLM-
SLWEGISKARADQLMASVLIEEILENIARFADPEATIHQSNKAKHFKYYGEEVDPVHLAYILSIFALVATQ
VTVSDRLLKDRYVLNLQIFRGFTSLLWMALQLAEKGPAATVFTLGTIAGLGALVLFIILL-
RDSVLVETLYYRYVYVVMRGYTAAKDVTDADPEHLLILAHATRDIEWIEIIPAFFYYFKTATSRLSKNTAI
AKEFFGLFPDRLRIVEVLAVPPERVEALVEFVALAGTLPTAADALPGIIASGMAEPNAAAVLEADQDW
NLHREMGITLAFYKAQSSVKGYFDKGLHKAVAAVSLKEAGGFTTPFHAGATAKKCAPAVHRVIDLGEL
EASLIEIDSAVVVADEEQSLASKPTTRENSVGAVGGVILAGTMNMGANLDSVERSGNIIIELEEETYNAV
NGKKADGKLTDKVLMVLSAAFFVVMRRLSSANALDKGAINLLVDAPDDADNIDHGRVLVIPAPTVHS
ETTPLYAESEIERIAWVAPLHMLGTLPAVLEFEPDQYSFISMKIKASLEIMETAEVLDDYHVLEKGSRPPD

FRIILIRGLHIRALAGIGTIIVVGSAGRTEITAYIEG-
AVPITLNKDTFLPAFAASSCHVIDIFSTMLRPPERSTIVTMILETVAEL-
LEVTRFANDKLERLKLLLPGLDMAEILTLVAQAADFSQIVTIDAAITAILSVAAEAVVLREMLLTLAEIAVT
ALALALALLLMLTPDRGLTQREAAREEQMKELIQGGVVLQVRAADRMVLASTADKVRLSTDEGTTSA
FASLNLATVSDGFYVMASAVLQMSSDLMMIVSVARVLADKESKNALVVRHPEQVKTQATYAEGSPG
MVLQTLTDHIPPLAIKPEDVELLWTLVAALWRVKAHLAQATRPHRERVLVLLLALSLIVVLLQSEGGGI
GGG-
MGAAAKILAAFSILTLKASVGEEVRKVARRVMDLNSRLGRPEEAVNPEFRGAELEQTKYEAENVEVDC
LETVRRFVDIPFLVSTRTQHNESEDRGARLVLDVTRVRFIDL-
VTKKSEIILVERAETDGVISREMSKDLQALHPEAAALGKVAALQHFVAINDGHISNYVTADIHSVLGML
LLVLFIQQVRVGKVVWPSRREVTMVLIMTTVALFFLIIGL--
QLVFMLGGISWQQLTTLVLGEGVMAVRQTSTFPQDS-
IGILQNTYADLICAGRVTKADIEASLHIVVGARVARGTWYVIYHYDITQAQQEAGIMQRAFRQKTLSKS
LIDGRMEVKIKVVTSDRYA-
TYEIKGGQHRKGTRFNRVKVGHNIPVDVLARIKMAFSPPNLILVKTFGAVVQKRFAQPLTHMRWGAV
TIGPMEVASPYIQSKKARHAPGDSVVDLKPDNILRSVAVVPELIWAREKEDQGAVHDKYVKTTHKTAA
KRNRAVDKAQYDHRNQVELSFIGLAVKVLPEKAIVEATAGQLMPTATVTVTEGLRGVAATIRLDPMEK
TLIAYIKRMRPQLNLGGKTDKKIVKCLNNNLDDVEYGKLGRRKIKPLTIKSVYDVEHEHATELVKIMPYK
DDVEGRKSQFAEKNEAIFEPLKAFMMLFGFIARAVYALSLRVVRRTIVLVKTKIVVNAATVLSAIKIIGVSI
RLRHKSTNVADTNHTIKTIEARARMTLAGAGKVKLNLRPGKVGMKGGRIKGFMPYKNYNIFDKLSSGR
RGKVKELAKLKKVDKEISVDGIRVKQQGGRTPKADLSNAI-
NTRVGVDIVGEREQQASNPRANAEMGQVFAAIDARIHHHAAAMTGHALAAVLAVAAILGIGKAHA
LLAVKLKINAGIELHSAA-VETAVAAA-YADAGGEA-AIADVSEG-
LGLISISAESLDVCRVKRRSINELKKTKKEAYMSLTCFSGKGDRVKIVEASQTQFYEIVATRTAERHGIKAK
GTYGKERAMMQITPTKPVKVWVPVFVDESELLAPIRAVAETVGDGVTVVKLTKDIKVTMFEKAVASLA
VAGKSTTVVYTVLEKVTELSKDIIYEQRSKAHYGRGRLRSHVVTHTKSKRHLSMMKRTVVAAPHMTAA
RLTDGEVTVITGATMAFVMAEQDAV--
GDVDLHYHEEPRREAGLGIAGPETFLATDQPRVLALIFLRAYLIHLVDVGTKGDFAEAATIYDILRAMQ
NKLDRKLSADIAKTRMCLHDNLADMGVTELAPRRETAGDSILSAYWINGVTANADIILLVANLECACV
PENIDCALRLFYQRITLQAKERGGDVTVAVVAAQNQIEAAEGTVIYSIKGATPSTTQMKLNVEGISRSIV
KSLAVQLFQVHHFLEDTWLGTLLRAAGDVAYVDAKGGDEAVDHVIERDTELEVGPQKDIVTRKIRIYT
PAAPSAVVIWDPAIEREDKADAQGTIIKIYGPNLQDINCFTDIGAEPKIESLKSWDEKEKYVVHRDDIPE
DI-
TLVGKSSKVNKEFGIPDVEVCAPSKPGVTESIDRGEPKAGSCGEVANMAYPQESLVNAVSSANVQKNI
SMVLGGHPVGEMGISRGIERHLETSMCEIFVDKPDIDVALDFSTLALTPLRVGLDPPNAKVQIQGKETR
KLMAVLDGEADLQRHPKVSEATGIVKTAGAKARLPDDALVYDGVQNGDVSFTITDLPQGRV-
VTPVFNTIKAQLKIDKVMLKTFHVVTLSFSNNKGDEHLIKLRLNALKKSTRHYEMLLKIRETECADSPHAI
AVGKILKIDALLQTICVAHYTEIEPLLADDLTALGVMGQQGLLHVVPRLMILAVLHLWDIAAQMYVPK
W-LAASAIVTDITMGLDQRDRDWTY-CISEVVNNTFFARTADDDTEQRLHARNPI-VLL-
YGKFGMT-
ISPSGHKLESTPVTELETDNEGNVILSAVHLQDAMEYYVANLLLVFVDEMILAFGSGDVPIRFLVAEQAI
TGRENPGMVCLTRRTFPLDLQRDATFVLVFEDSTMKNLSHKKMHMNLVLIKSKGWIVNVSTKILISDQ
AIASYAEDAGAHTQPSESALAVITSRVMAPIVLVVSQKPTAPSKTLFLEALNLYDEVTATVQNKPKKYYI
FEGDVVTILDAIDAAVLLGMQRPQPDAMRRAIVAKAIRLAIALRASHVHTRLEHGLLNFSRAVSFTQ-

AYTMLDVYAHI-------EFRVRNHKVKTKRARPYKGHTKFLHKADMDKSLVAAGVDTLA
>GCA_003201935
AVIKPVTMDAYAPVLMIRQQQVATVSGAGSPTLRAKTVYAKSGLQRKSETKWVSKDFYY-
GVVKLGNIVQLVSISVDKRAVCTVAQLVDQEVVVRGMVALEAVKKKIALEAIQGMTAEDPAKVVDY-
---IPVVFEVV-
GIMVQRGQIEVDECAISTAVSDVDPIVYVSPIDLAAVAGDKMAYIDQEAAVANAKQEINLEELALESS
VLVVGI-PLLIPV-FAADGDV-MSFANRSRTLKTIVNLAVSYPEIKRA-LSNA--AAM-
MDASQAAALAEKRGTAEEIRIVKRMQRSSGLADDPELLMLEDAAEGNSNWAGQIYDDPISGSSESDS
AGNIVYAANKRQNFARSITYLRAVEISDPAAAPLMRGVKHNAKSRASRKVDDAITALNFPNVPEGIAI
RAEAGKSRDLLLHDATEGEEILLIIVRWHVTWKTAVIISLFPLADNVVGIITMGGIGAVGRKDGLLNRHH
KLNGSLIEQRRIVEKLTLRLRAALKQKLGAEQIIIFVVKVAGDLVAKAKVQM---
SVGTVVAIQGETIFVRRHRQTVIADQEATV-ILKGVAEGPADAQALLRIRVDHTVVKLSALLIPD-
PVLTESVSIREGVGKALQIIQLDVYLLRDMGTGEETLAKSRSREVIREVLQVDEITGVRLAFGQLKERLVK
GGTMKELG-----
IAILKNITEPRMANLPAGVCRTFVNAGPRAAVGMIFLLLDEAGKIYTIDGDLRENTKSIKSMIGLVDAAK
PKTLSAGYATTRLKIRYADVTVLTSDADLYAYAEERKELLRMVPVYITNQAKRRQLIIFALSLKGGHRRA
RAALGVMIAAAILESQAEQGAVNHAEPAVLLDDGEIVGSEIMISRQAALDLKQIIKTDIIVIFPEFQEKM
VVSLKFFCTDVAIFVIALICTVGEMLSREPSHAQEEGAGFQKRIRAHSVLFALAVTFDRRNLRTD-
TPALFLNLGLAPPRYGVFYTEQPKRKQMPSEEDKEEVDGFDLVNAIKEDGTK-EELDMPEAMALAVE-
DATINVNAGVLAMSGDMAAVEKVDSKRKMALAVDKWTIARRDIVDLKISGAAKFRALG-
IEQADVSATLSKKQVWFLFLPGANNAEMEEAKLDGAGSSTAEAAADKQLGILLINPVLVVIQDSNTAG
LIANGGLDEWGTMAYKKHYALAARMRLHMRTID------
EMDPNKQKAEKIKTVKLEGDTRFRQLATEGVKEQMP-
LHALNPDFAVLVPVLVTPAVGRVAADAGVLGARVPCDASL-
RELQKSMNGNALPISGIRLLGMRAARAMVDEDIMVAGKVAA-
LLVIVAVTTITHIVLRNDSEATLPWRLSVAERIEADTKRASGFAARPFKSKPSLCKTQAHAQIADPIQEIA
AGYVATEGRFVFPVEGIPIQLILASPHSRIGAAKRAGEGHLHEMAKELQAKAVIMVLNEPDTNTEGLLN
YLTTQQPTDLLFEDCLDVVIDMRRMSVV---
VQLNTV-
HVLVPGVRELADREIMAAAAHSGLVQIERVDERTGRERTLMWMYGAMEELWLMSIRYADWVVDCA
IGAILLHVYAQQINRAFSLERLQLAGWPMEATRWWNTWQLWLNKDGFFSYEAAFRAVKAVLKADL
DDFDMSDVEGVGRYQSRQQLRVGYRIRQQVAFEQLDPRTDILLQSMTRLSVIRHALTMVAILIVREIV
VEFTLVVAKEDVAMGHVECVLADAFDFTAKDADIGPVGIRTPDPVLGNGTVLKEAVLGKIDLANTVA
DFRYKMLIDGGRELGVSWVTLQNLIYARIWVPPAQAALKNLVPIAFFLGHTELNLHIFPMLWAGISKA
RANKLMAGALIEKILEKVAAFADPEATTHQSKKIRHFKYYGEEVDPVYLAYILDMFALVATQVTVSDRL
LKDRYVLNLKIFRGFTSLLWLSAQIGDKGPAATVITVQTIMGLGALVLFIILL-RVVVLVETLYYKYVY-
VMLGYQAAKDVADADPEHFLSLAHASRDVEWIEVIPAFFYYFKTATSRLSKNTALAKEFFGLFKERLRII
DVLAVPPDRVEALVAFVALAGTLVTSAQALAGIIAYGFCEPNAAAVLEADQDWNLHRSMGITLAFYD
ARESVKGWFDKGLHKDVAATGLREAGALTTPFHAGATAKQCVPAIHRVLDLGEVEAALIEIDSAVLVA
DEEQTLADKPTTRENSVGAVGGVVLAGTMNMGANLDSVERAGNIIIELEEEQYSAVNGKKSDGKVT
DKVVMVLSAAYFPVLKRMASANALDKGAFGLLIDAPDDADNKDHGRELTIPAPTVHTESAPLYAESEI
ERIAWIAPLHLLGTLDAVLGFEPDQYSFISMMIDASLEIMETAEVLDDYHVLETGSRPPAFRVILIKGLHI
RLLAGVCTIIVVGSAGRTEVTAYIQG-
AVPITLNKKTFLPAFAASSCHAIDIFSTMLRPPERSTIQMLILETVAEL-

LEVTRGENDKLERMVALLPGLDVASLLDLVAAATDFAAIVTIDQALTAILSVAAEAVVLREMLLTLAEIA
VTALALALALLLMLTPDQGLHQKEQAREAQMKALIQGGVVLQVKAADRMMLASTADKVRLDTDEG
TTSAFASLNLATVSDGFYVLSSAVLQMTADLMMIVAVARVLADKETKNALVVRHPEQVKTQSTYAEG
SPGMVLQTLTGAIPPLAIKPENVELLWTLVAALWKVKAHLANAARPHNERVLILILALSLITVLLQSEGG
GMGGG--
GPAAKALAAFSILTIKAKAGDEVRKVARRVMDLNSRLGRPEEAVNPEFRGAELEQSKYEAENVEVDTM
ETIRRFVDIPFLVSIRTQHNESEDRGARMVLDVTRVRFIDL-
VTKRSEIILAARAETDGIIAREMSKDLAALHPE-
AALGKIAALQHFIAVQDAHISNYVTAEIHAVLGMILLVLFIQEVRVGKVVWPTRREVTMVFIMAALAIFF
LVIGLGVQIVFMLSGISWNQLTTIVLGEGIMVVRQTSTFPEDS-
IGILQNTYADLICSGRVAKADIEASLHIVVAGRVARGTWYVIYLYEISQAQDQSGIMQRAFRQMTLSQ
SLIDGRMEVKIKVVTSDRYA-
TYEIKGGQHRKGTRYNRVKVGHNIPVQVLPRMKMAFGPPNLVLVKTFGAVVQKRFAQALTHIRHGA
VTIGPMEVSSPYISNKKARHAPGDSVVDLKPDNIVRSVAVVPELIWARENEDNGAVHDKYVKTTHKL
AAKRKAAVDKAQYDHRNQVELSFIGLAVKVLPEKAIVHATSGQLMPTASVAVTDKMKGIAATIRLDP
MEKALIAYIKRMRPQLNLGGQTDKKIVKCLNNNLDDVEYGKLGRRKIKPITIKSVYDVEHRRATELVKL
MPYKDDVESRKSQFAEK--
ALFEPLKAFMMLFGYIARAVYALYLRVIRRTILLVKTKIVVNASTVLSAIKIIGVSIRLRHKSTNVAETNHHI
KTIEARARMTLAGAGKVKLNLSPGKIGKKGGRIKGYMPYKNFNIFDGLASGRRAKVKELAKLKSVDKTIS
VDGVRQKQQGGRTPKAQLSNAI-
NTRVGVDIVGENEQDESNPRANAEMGQIFAKVDARVFSHVGALIGHALEAVLAVVSIWEIGRAHNLL
AVRLKINAGIEMHSAA-
VDTAVAAAAFAQNAGEASVIADISESYLGALAISATSVDVCRLRRRGLSELKKTKKDAYMSLTCFGGK
GDKVKIVEASQTQAYEIVATRTAERHGIRAKGTYGKERAMMQILPTKPVKIWVPVFVTEVELLAPIRAF
AESVGDGVTVVKLTKDVKVTMFEKAVASIAVTGKSTSVVYTVLEKVTELMKDIIYTQRAKAHYGRGRLR
SHVVTTTKQSRHLSMIKRTVVADPSMDASRLTDGEVTVITGATMAFVMSEQEAV--
GDVDLHFHEEPRREAEVGIGGPETFLTTEQPRVLALLFLRAYLIHIVEIGTVNGFSDAATIYDVLRAMQN
KLDRKLSADIAKTRMVLTDNVTDMGVTDVAPRREAAGDKILSAFWINGVTANADIILLVANLEVLCAP
ENIDCAIRLFYQRITLQAKERGGDVSVAIVPAANQIEAAENTVIYTIKGKTPGTGQMKLTIEGISRSIVKSF
AVQLFQVDTFLDDTWIATWLRAALQVVFVGAEDGKEAVDHVVERDTELEFAPQKDIVSRKIRIYTPAA
PSAVVIWDPAIEREDKAEAQGTILKIYGPNLQDINCFTDIGAEPKIESLKAWDEKEKYVVHRDDIPEDI-
TLVGKSSKVNKEFAIPDVAVCAPSKPGVTESIDRGEPKAGTCGEVANMAYPQESLVNAVSSANVQKN
ISMVLSGHPVGEMGISREIERHLETSMCEIFVDKDDVDVALDFYILQLTPLRVGLDPPNARVQIDGKES
RKLMAIQDGDAALQRHPKVSQATGTGLKAGVKARMPDDALMVDGVMHGDISFTATDLPDGRV-
VTPVFNNVKAQLKIDKVLLKAFHVVTLSFSNDKGEEHLIKLRLNALKKSTRHYEMLLKIRETECAESPHAI
SVGKVLKIDALMQTICQAHYKEIEPLLADELTALGVMGEQGLLHIVPRLMILAVLQLFKLEIALQIDNW
GLQAEALVTDQTMGLYLRDRAWSF-AISEVVGNTFFARTKETDTEQKIRARNPV-VLL-LGKFKMI-
ISPSGLGLASTAKSPQERGNAEELILSTERLQDEMGYYVLQILLVFVDEVILAYGSGEEPIKFLVAEQAVT
GRQNPGMVTLTRRTFPLELQRNATFVMVFEDSTMKNLSHKKMHMNLVVIKSKGWIVNVSTKILISDQ
AIASYAEDAGAHTQPSESALAVITSRVMAPIVLVVSQKPTAPSKTLFLEALSLYDEVTSTVQNKPKKYYIF
EGDVVAVLGTLDAAVLLGMQRPQPDRMRRAIVADAVRVAIALRASGIVTRLEEGLINFSRAVNFAALF
YSA--------------EFRVRNHDVSTKRSRPYKAHTKFLHKADVDKGLIATGVDTLA
>GCA_003217255
AVIKPVTIDTYAPVLMIRQQEVATVSGAGSPSLRAKTVYAKSGLQRKNSTKWVAKNLYY-

GVVKMSSLPQLVSISVDSRAVCTVAQVVEQEVVVHGMVALEAVKKKIALEAINGMTSEDPAKVVDY-
SEVQLKRFREAEDRADLLPVMLGLTLSIRFDPHEPVVHDLPIPVIFEVV-
GVMAQKGTMEVDDCAVSTAISDVDPIAYVSPIDLDKVSGQKMDYIDQESAVAALKKELELEELALEST
VLVVGI-PLLIPV-FAADGERVMSFANRSRELESIVELAVSAVVQTRAR-TGAYASAM-
MQRARAAALAEKRGTAEEIRIVKRMSRSAGLADDPELVILEDAAEGNAQWAGQIYDDALSGSSESDS
QGNIVHAANKRQNETRSITYLRKVEISDQATAPMMRGVKHNAKSRTHRKLSDSIAELNFANVPEGIAI
AAAGALREALLLHEAGEGEEILLLVVRWRVTWKTAIIIILVETPGDIVGLIELGGIAAVGHPEGLLNRHHK
LNGSLIEQKRVVEKLTLRLRAALKQKLGAESVIILVVKVAGDLVADATVQMG--
DIGAVVAIQGEVIYVRRHKQTLITDREATVEVLRGVAQGPVDSQALLRIRVEHTLVEISVLLIPE-
PVLVESVAIREGVGKALQIIQLDVYLLRDMGTGEETLAKSKSRELIRDVLMVDPITGVRLAFGKLTERQA
KNTELKQLRTLL--
LSILAGVTEPRMEDLPDGVCRTLQQAGCRAAVGMLFLLLDEAGRLYAIDGELREIPKTVKSLIGLLDAAK
PKVLSASYATTRLRVRFADAAVLEAEANLYAYREERKQLMRMVPVYITVAAKRRQLVVLALSLRGGHR
RALAAIGVLIAAAIIESQAVNGAIHHDEPRILLDDAEVIGTEMMITRQAAMDLKALLKVDVVVLFPEFQE
KMVVSLKFFCTDVAIFVIALITTVGQMLSRSPAHAQESGAGFGKRIRGHSMLFALAAVFDRRNTGVDR
LPELFLGLGLALPRYGLVYTQQPKKRQMPSEPDSEKTDGFDLVHAVKADGRS-
HELDMPEAMALAVEANAPINLVGGVLGYSGGMSAIARVDSKRKTALAVT--------------------
----
LFLPGANNAELEEEKLDGAGASTADVIADRQSPILLIVPVLVVIQDSGSAGLVANGGVEEWGTMAYKK
HYAYAARMRLHMRTIDVIEVGVALEPNKQKSEKIKTVKLEGDTRFRQLAVEGVKEQMP-
IHALNPDFAVLGPVLVTPAVGRVAAATEVLGARVPCDASL-
RELQKSQKGDALPISGVRLLGMRAARAMIEEDIMVAGKIAA-
LLKIGALTTILSLVLRNDSEATLPWRLSVAERIEADARRGGGFAARPFRSKPSLCVTQAHARTADPIQEIA
ASYVASEERFVFPVEGLAIQLILSSPHGRVDSAKRAGEGHLHEMAKELQAKAVLMVSNAPDTSTEGLV
KYLTTGQPTDVLFEDCLDIIIDLRRMSVV--
VHLNTV-
HVVVKGVRDVGDFEIMNAAADSGLVQVEQVDERTGRERTLMWMYGAMEDLWLMSVTWADWVV
DVPIGAIVLHVYAQTLNRAFSLRRLQLNGWPPEAARWWNTWQLWLNKDGFFGYEAAFRAAKQVLK
ADLYDYQLRDVVGVGRHLSRQELRVGYRIRDQVEFAQLDPRTDILLQSKTRLSCIRHALALVAILILRSL
VAEFT-
VVAMEDVAMGHVECVLADRFDFTAKNAHIGPVGLKGPAPVLGDGTVFQDAVLGKIDFAKTVAEFRY
LTLTDGGRELGVRWIGL-
NMLVERIWLPPAQAALKDLEPILFFLGHPELNLDWLDALFAGISKSRAQRLMAGTLVEQILDRVVAFAE
PEATTHQSRKTRHFKYYGEEVDPVYLAYILEIFALVATQVTISDRLLRDRYALNMRVFRGFTSLLLLGLQL
GDNGPSATVISVDTVAGLGALVLFIILL-RDAVLVETLYFRYVY--
QRGYTAAKDVADADPEHFLALAEVTRDVEWVEIAPAFFYYFRTAT-
FEPFNTAIAREYFGLFEERLRIIDVLVVPPDRVEAISEFVAVAGTLPTAATLAPGVIACG-
CTPNAAGVLEAEQDWNLHRTLGITLAFFKAQESVKGWFDKGLHKGVAATGLEEAGAMTTPFAAQA
QAGRCAPVLHRVLDLGELEAALIEVDSAVMVASEEQTLADKPTTREKSVKAVGGVVLAGTMRMGAS
LDSVERGNNIIIELEEEQYHAVNGKKADGKMTDKILMVLSQAFFPVLRAPASANAMDKGAVDLLVEA
PDDADNLDHGRELTIPAPTVHAETAPLYAESEIDRIAWIAPLHLLGTLGAVLGFEPDQYSFISMMIDASL
EITETAECLDDYHVLEKGSRPSEFRIILIRGLQVKALAAIATIIVVGSAGREEATAYIQG-
AVPITLKRQTFLPAFAPSSCHKIDIFSTMLRPPERSTVMMLILETVAEL-
LEVTRGENSKLERLALLLPGLDVAALLGLVAAAEDFSRIVTIAASVTAILSVAAEAVVLRELLLTLAEIAVT

ALALGLALLLMLTPDQGQIPRETAREAQASDLIQGGAVLQANAQDRMVLASTADKVRLSTDEGTTSA
FASLNLATVSDGFYVLSSAVLQLSSDLMMVVKVARVLADKEHKNALVVRHPEQVKTQPTYAEGSPG
MVLQTLTGHVPPLAVKPEAVELLWTLVAALWRVKAHFAEAMRPRLERVLVLVLALSLIAVLLQSEGGG
--FG-
MGGAAKGLAAFSIMTIKAKVGDEVRKVTRRVMDLTSRLGRPEETVNPEFRGAELEQSKYEAEAVEVDT
VETIRRFVDIPFLVSTRTQHNESEDRGARMVLDVTRTRFIDL-
VTKRSEIILAARAETDGIISREMSKDLQALQPEAAALGKVAALQHFISLGDAHISNYVTAAIHSVLGMLL
LVLFIQQTRIAKVVWPTRREVTMVFLLATIAIFFLVIGLAVQIVFMLGGISYQAITTLVLGEGVLAVRQTST
FPADS-
VGILQNTYADLICAGRVAKADIENSLHIVVGARVSRGTWYVIYLYEITQSQGEAGILQRAFRQATLSHS
LIDGRMEVKIKVVTSDRYA-
TYEIKGGQHRKGTRFLSVKVGHNIPVDVLARIKMAFSPANLILVKTFGAVVQKRFAQPLTHVRHGAVTI
GPMEVASPYIRSKKARHAPGDAVVDIKPDNIVRSVAVVPELIWARENEDNGAVHDRYTTPTHKLQAA
RRKAVDKAQYDKRNQVDMSFIGLAVKVLPEKAMVHATGGTLLPTERVAVTEGIRGVAATIQLDPMEK
ALIAYIKRMRPQLNLAGTNDKKIVKCLNNNLDDVEWGKLGRRRIKPITIKSIYDVEPRRSTELVKLMPYK
DDVEQRKSQFAEK--
AIFEPLKAFMMLFGFIARAVYALSLRVVRRTILLVKTRIVVNADTVLSAIKIIGVSIRLRHKSTNVADTRHHI
KTIEKRARMTLAGTGKVKLNLRPGAVGKKGGRIKGYMPYKNFNIFDGLDSGRRAKVKELAKLKKVDKEI
QVEGIRMKQQGGRQSKADLSNAIANTRVDVDIVGENEQEESNPRANAEMGQVFAAIDARVYHLVG
ALIGHALEAILAVVAIYGIGRAHALLADKAKVNAGIELHSAA-VERAVAAA-
YSRTAGDAALIADVSEAYLGALSITEASMDVCRLRRRGLRELKKTKRDAYMSLTCFGGKGERVKIIEASQ
TQAYEIVATRTAERHGIHAKGTYGKERALMQIVPSKPVKVWVPIFVSEEELLAPIRAVAETNGDGVTVV
KLTTDVKITMFEKAIASLAVTGKSTSVVYTVLEKVTELSKDIIYEQRAKAHYGRGRLRSHVVTTTKSKRHL
SMIRRTVVAHPRMEAQRLTDGETSVITGATMAFVMSEQDAV--
GDADLHFHEEPRREAALGIAGPQTFLATAQPRLIALLILRAYLIHIVEIESVGGFADAATIYDILRPLQNKL
DRKLSADIAKVRMCLHDNLADMGVIDEAARRDDAGDPILSPYWINGVPATADIIMLVANMEAPCVP
ENIDCAIRLFYQRITLQARERGGDVTVAVIKAANQIEAAEGTVIYTIKGEAPGTLQMKLTVEGVSRSIVKS
LAVQLFQVTTFLNDTWIATLLRAATEVVFVDAKDGKEAVDHVVERDTELEMAPQRDVLARKIRIYTPA
APSAVVIWDPAIEREDRADAQGTILKIYGPNLQDISCFTDIGAEPKIESLKSWDEKEKYVVHRDEIPEDI-
TMVGKSSRVNKEFGIPDVAVCAPSKPGVTESIDRGEPKAGSCGEVANLAYPQSSLVNGVSSANVQKN
ISMVLTGHPVGEMGISREVERHLETSMCEIFVDKADVDVALDFYRLALTALRVGLDPPNARVSIDGRES
RKLMAILDGDAELQRHPKVSEALGITRNAGTKARLPDDSLIFDGVHHGDISFTATDLPEGRV-
VTPVTLKVKQQLKIDKVMVKTFHVVTLSFSNNKAEEHLIKLRLNALKKSTRHYEMMLKIRETECAESPH
AIPVGKILKIDALLQTICVAHYTEIEPLLSDELTALGVMGQQGLLHVVPRLMILAVLLLV-
LEARQLVPRW-LMRRAVVTDQTMGLAQRDRDWTF-AISEVVGNTFFARTADDDTADRLHARNPI-
VLL-YGKFGMT-
ISPSGLRLDSTAPDDMAEGNQTQVILSAERLHDAMEYYVVSLLLVFVDEVILAYGSGDRPIRFLVADQA
VTGRANPGMVTLTRRTFPLDCQRDAVFVLVFEDSTMKNLSHKKMHMNLVLIKSKGWVVNVSTKILIS
DQAIASYAEDAGAHTQPSESALAAITSRVIAPIVLVVKQKPTAPSKTCFLEALALYDDITATIRDRPKRYFI
FEGDVISVLGGLDAAVLLGMQRPQPDRMRRAVIARAVRLAIALR--------
EAGLINFSRAVTFAARVWGALDA-----------
EFRVRAHSVSTKRSKPYKEHPKFLHKAEAEKSLVAAGVDTVA
>GCA_003318235
AVIKPVTMNTYAPVLMIAQSQVATVSGAGSPSLRAKTVYAKSGLQRKAETKWVSKDFYY-

GVVKMGNLVQLVSISVDNRAVCTVAQLVDQEVVVHGMVALEAVKKKIALEAIEGMTAEDPAKVVDY
-GEIELRKFRDADDRADMLPVMLGLTLKIKFDPHQPLVHDLAIPVVFEVV-
GIMVQKGTMEVDDCGVSTALSDVDPIVYVSPIDLAAVSGDAMAYIDQEGAVAAAKASIDLD-
LALESNVLVVGI-PLLIPVSFEADGDT-MSFANRSRTLKTIVNLAVSYPAIKRA-----IAAAM-
MDASQAAALSEKKGTAEEIRIVKRMQRSAGLADDPDLVMLEDAAEGNSNWAGQIYDDPLSGSSESD
SAGNIVFAANKRQNFARSITYLRFVEISDKAAAPLMRGVKHNAKSRAARKLGDAIAVLNFANVPEGIAI
RAEAGKSQDLLLHDATEREEILLIVVRWHVTWKTAIIVALFPTAGDVVGLIDVGGIGAVGRKEGLLNRH
HKLNGSLIEQRRITEKLTLRLRAALKQKLGKDQIIIFVVKVAGDLVATATVQM---
AVGAVVAIQGPTIFVRRSRQTVIGDQEATVEILQGVAEGPADAEALLRIRVDHTVVKLSVLLIPQVPVLV
ESVTIREGVGKALQIIQLDVYLLRDMGTGEETLAKSKS--------------------------------------
------
ISILKHVTEPRMENLPAGVCRTFVNAGPRAAVGMLFLLLDDAGKIYTIDGDLRENTKSIKSLIGLVDAAK
PKILSAGYATRRLKIRYADVHVLISDAELYAYAEERGELLRMVPVYIINAAKRRQLILFALSLKGGHRRAR
AALGVMIAAAILESQAEQGAVNHEEPEVLLDDAEVVGSEMMISRQAALELKHVFKLDIIVIFPDFQEK
MVVSLKFFCTDVAIFVIALICTVGQMLSREPSHAQEGGAGFQK----HSVLFALSVTFERRNLRDA--
ARLFLALGLAPPRYGVFYTEQPKRKQMPSEADSEKVDGFDLVNAIKEDATSNHELDMPEAMALAVE-
DATINVNAGVLALNGDMAAVEKVDSKRKMALAVEKWTISPVSLVDIKISGAQPFRALD-
IEQADVSASLPKKEV--
LFLPGANNAELEEEKLDGAGASTADVAEDKQSAILLVNPVLVVIQDSNSAALIANDGLEEWGTMAYK
KHYAFAARMRLHMRTID----GVAMDPNKQKAEKIKTVKLDGDTRFRQLATDGVKEQMP-
LHALNPDFAVLIPVLVTPAVGRVAADAGVLGADVPCDASL-
RELQKSQKGSALPISGIRVLGMRAARAMVDEDIMVAGKVAA-
LLVILALETITAIVLMNDSEATLPWRLSVAERLEADTQRSAGYASRPFNSKPSLCKTQAHAKIADPIQEIA
AGYVATEQRFVFPVEDIPIQLILASTHSRVGAAKRAGTGHLHEMAKELQAKAVIMVQNEPATNTDGLL
NYLTTQQPTDLLFEDCLDVVIDMRRMSVV--
VHLNTV--
VVVEGVTPVGSYEIMAAAAHSGLVQVEQMDERTGRERTLMWMYGAMEELWLLSVTYADWVINCTI
GAILLHVYAQQINRAFSLERLQLAGWPPEATRWWNTWQLWLNKDGFFSYDAAFRA-
KAVLKADLDDYDMSDVEGVGRYQSRQEIRVGYRIREQVAFAQLDPRTDILLQSMTRLSVIRHALKMV
AILIVREIVVEFTMVVAQEDVAMGHVECVLADSFDFTAKNADIGPVGLRTPDPVLGDGTVLKEAVLGKI
DLANTVADFRYKMLLDGGRELGISWVTLQNMIYARIWVPPAQADLKDLQPICFFLQHPAIDLLGTDW
FYEGISKARANKLMAGALIEKILEKVAAFADPEATTHQSKKLRHFKYYGEEVDPVYLAYILNMFALVATQ
VTVSDRLLKDCYVLNLKVFRGFTSLLWLSAQIGDKGPCATIITVQTVAGLGALVLFIILLMRAVVLVETLY
YKYVYVVMLGYQAAKDVADADPEHFLSLAHASRDVEWIEVIPAFFYYFKTAKKNLTKNTALAKEFFGL
FEERLKIIDVLAVPPDRVEALVEFVALAGTLTTSAAALIGIIACGFCEPNAGAVLEADQDWNLHRSMGIT
LAFYKAQVSVKKWFDKGLHKDVAAIKLNEAGALTTPFHAGRTAKQCVPAIHRVLDLGEVEAALIEIDS
AVVVADEEQTLADKPTTRENNVGAVGGVVLAGTMNMGANLDSVERGGNIIIELEEENYNAVNGKK
NDGKMTDKVLMVLSRAYFPVLKRMASANALDRGAFGLLIDAPDDADNQDHGRSLTIPAPTIHAESA
PLFAESDIERIAWIAPIHLLGTLDAVLGFEPDQYSFISMMIEASLDIMQTAEVLDDYHVLEKGSRPPGFR
VILIKGLHIRLLAGICTIIVVGSAGRSEITAYIQG-
AVPITLNKHTFLPAFAASSCHVIDILSTMLRPPERSTIVMLILETVAEL-
LEVTRIENDKLERMDALLPGLDVAVLLGLLATAADFAEIVTIDAAITAILSVAAEAVVLREMLLTMAEIAV
TALALAMALLLMLTRDQGLHQRETAREAQMKALIQGGTVLQVKATDRMMLASTADKVRLATDEGT
TSAFASLNLATVSDGFYVMSSAVLQMSSDLMMIVAVARVLADKETKNALVVRHPEQVKTQSTYAEG

SPGMVLQTLTAAIPPLAIKPEDVELLWTLVAALWRVKAHLANAARPHAERVLILILALSLITVLLQSEGG
GMGGG--GPAAKILAAFSILTI--
KAGEEVRKVTRRVMDLNSRLGRPEEAVNPEFRGAELEQTKYEAESVEVDTMETIRRFVDIPFLVSTRTQ
HNESEDRGARMVLDVTRVRFIDL-
VTKRSEIILAARAETDGIIAREMGKDLAALHPEAAALGKIAALQHFIAIQDAHISNYVTAEIHAVLGMLLL
VLFFQEVRISKVVWPTRREVTMVFILAALAIFFLVIGL--
QIVFMLSGISWNQLTTIVLGEGIMIVRQTSTFPADS-
IGILQNTYADLICAGRVAKADIEKSLHIVVAGRVARGTWYVIYLYDISQAQGQSGIMQRAFRQMTLSK
SLIDGRMEVKIKVVTSDRYA-
TYEIKGGQHRKGTRYNRVKVGHNIPVQVLPRMKMAFTPPNLVLVKTFGAVVQKRFAQALTHMRHG
AVTIGPMEVSSPYISNKKARHAPGDSVVDLKPENIVRSVAVVPELIWARENEDNGAVHDKYVKTTHKL
AAKRKKAVDKAQYDHRNQVELSFIGLAVKVLPDKAIVHATAGQLMPTASVAVTEKMKGIAATIRLDP
MEKSLIAYIKRMRPQLNLGGQTDKKIVKCLNNNLDDVEYGKLGRRKIKPITIKSVYDVEHKRATELVKL
MPYKDDVESRKSQFAEK--
ALFEPLKAFMMLFGFIARSVYALYLRVIRRTILLVKTKIVVNAATVLSAIKIIGVSIRLRHKSTNVAETNHHI
KTIEARARMTLAGTGKVKLNLHEGKIGMKGGRIKGFMPYKNFNIFDGLASGRRAKVAELAKLKDVDKTI
SVDGVRVKQQGGRTPKAQLSNAI-
NTRVGVDIVGEDEQEASNPRANAEMGQIFAKIDARVFSHVGALIGHALEA-
LAVVAIWAIGRAHNLLAVRLKINSGIEMHSAA-VSAAVAAA-FAQAAGEA-
AIADVSEGDLGALALTATSVDVCRLRRRSLSELKKTKKEAYMSLTCFGGKGDKVKIVEATQTQSYEIVAT
RTAERHGIRAKGTYGKERAMMQITPTKPVKIWVPVFVDEVELLAPIRAFAETQGDGVTVVKLTTDVKV
TMFEKAVASLAVTGKATSIVYTVLEKVTQLMKDIIYTQRAKAHYGRGRLRSHVVTTTKQRRHLSMMKR
TVIADPSMKAARLTDGEVTVITGATMAFVMSEQDAV--
GDVDMHFHEEPSREAEVGIGGPETFLTTDQPRVLALLYLRAYLIHIVEIETTGGFADAATIYDVLRAMQ
NKLDRKLSADIAKTRMCLTDNITDMGVTDVAARRNAAGDKILSAFWINGVTANADIILLVANLECPCV
PENIDCAIRLFYQRITLQAKERSGDVSVAVVPAANQIEAAEGTVIYSIKGQTPGTGQMKLTIEGISRSVV
KSLAVQLFQVDTFLNDTWIATWLRAAKDVVFVASEDGKEAVDHVVERDTELEFAPQKDIVARKIRIYT
PAAPSAVVIWDPAIDREDKAEAQGTILKIYGPNLQDINCFTDIGAEPKIESLKAWDEKEKYVVHRDDIPE
DIDTLVGKSSKVNKEFGIPDVAVCAPSKPGVTESIDRGEPKAGTCGEVANMAYPQESLVNAVSSANV
QKNISMVLSGHPVGEMGISREIERHLETNMCEIFVNKEDVDVALDFHILALTPLRVGLDQPNARVQID
GKESRKLMAIQDGDAALQRHPKISAATGLTQ-AGTKARLPDDALILDGVMHGDLSFTATDLPEGRV-
VTPVFNEVKAQLEIDKVILRGFHVVTLSFSNEKGEEHLIKLRLNALKKSTRHYEMLLKIRETECAESPHAIS
VGKVLKIDKLLQTICKAHYKAIEPLLADDLTALGVMGQQGLLHVVPRLMILAVMHMARLEIALQIAGW
RLQAQAVLTDQTMGLSIRARDWSY-AISEVVGNTFFARTKEDDTEQRIHARNPV-VLL-LGKFVMI-
VSPSGLTLASTEPSPQDRGNAEELILSTERLQDEMGYYVLQILLVFVDMVILAYGSGDDPIKFLVAEQA
VTGKQNPGMVTLTR-
TFPLELQRNATFVLVLEDCTMKNLSHKKMHMNLIIIKSKGWVVNVSTKILISDQAIASYAEDAGAHTQ
PSESALAAITSRVMAPIVLVVSQKPTAPSKTLFLEALSLYDEVTATVLNKPKKYYIFEGDVVAVLGTLDAA
VLLGMQRPQPDRMRRAVVAEAIRLAIAIMASGVVTRLEEGLINFARSVSLAVGVYPTLDMLPAAIIA-
TALEFRVRNHDVSTKRSRPYKAHTKFLHKADVDKSLIASGVDTLA
>GCA_003337495
AVIKPVTMNTYAPVLMIAQSQVATVSGAGSPTLRAKTVYAKSGLQRKAETKWVSKDFYY-
GVVKLGNIVQLVSISVDKRAVCTVAQLVDQEVVVRGMVALDAVKKKIAVEAIEGMTAEDPAKVVDYR
AELELRKFRDADDRADMLPVLLGLTLKIKFDPHQPVVHDLPIPVVFEVV-

GIMVQRGQIEVDECAISTAISDVDPIVYVSPIDLAAVAGEKMAYIDQESAVAAAKAEIELEELALESSVLV
VGI-PLLIPV-FAADGDV-MSFANRSRELKTIVNLAVSYPAIKRA-IDN--GSAM-
MEASQAAALAEKKGTAEEIRIVKRMQRSSGLADDPDLVMLEDAAEGNSNWAGQIYDDPLSGSSESD
SAGNIVFAANKRQNFARSITYLRAVEISDAADAPLMRGVKHNSKSRAARKLNDAIAALNFPNVPEGIA
IKAAAGKSTDLLLHDATEAEEILMIIVRWHVTWKTAIIISLFA-
AGNIVGLIELGGIGAVGRKEGLLNRHHKLNGSLIEQRRVVEKLTLRLRAALKQKLGAEQIIIFVVKVTGDL
VAKAKIQM---
SVGAVVAIQGETVFKRRGRQTVIADQEATVEILKGVAEGPADAQALLRIRVDHTVVKLSVLLIPE-
PVLVEAVTIREGVGKALQIIQLDVYLLRDMGTGDETLAKSKSREQFREVLQVDDITGVRLAFGQLKERL
VKGGSMKELGTLL--
IAIIRGITEPRMANLPAGVCRTFIAAGPRAAVGMIFLLLDDAGKIYTIDGDLRENTKSIKSMIGIVDAAKPK
ALSAGYATDRLKVRYADVTVLTSDADLYAYAEERRELLRMVPVYITNAAKRRQLIIFALSLRGGHRRAR
AALNVLIAAAILESQAERGQVNHAAPVVLLDDGEIVGSEMMISRQASLDLKQILKIDIIVIFPEFQEKMV
VSLKFFCTDVAIFVIALICTVGEMLSREPSHAQEAGAGFGKRIRSHSVLFALAVTFDRKNLGDA---
DLFLNLGLAPPRYGIVYTEQPKRKQMPSEAAEEEIDGFDLVNAIKEDTSSNHELDMPEAMALAVE-
DSTINVSAGVLAINGDMAAVEKVDSKRKMALAVEKWTISPLPISDLKISGAAKFRVLG-
IEQANVSATMPKKTVWFLYLPGANNAEMEEAKLDGAGSSTADAAEDKQLGILLVNPVLVVIQDSNT
ASLIANGGLEEWGTMAYKKHYAFAARMRLHMRTID------
VMDPNKQKAEKIKTVKLEGDTRFRQLATEGVKEQMP-
LHALNPDFAVLVPILVTPAVGRVAADAECLGARVPCDASL-
RELQKSQKGNALPISGIRLLGMRAARAMVDEDIMVAGKVAA-
LLVISAISTITAIVLMNDSEATLPWRLSVAERLEADTKRGGGFASRPFKSKPSLCKTQAHARIADPIQEIA
AGYVASEERFVFPVEDIPIQLILASTHSRVGAAKRAGLGHLHEMAKELQAKAVIMVLNEPGTNTEGLLN
YLTTQQPTDLLFEDCLDVVIDMRRMNVV--
VQLNTV-
HVLVPGVSDLGEREIMAAAAHSGLVQVEQMDDRTGREKTLMYMYGAMEELWLRSLTWGDWVIDC
TIGAILLHVYAQQINRAFSLERLQLAGWPPEATRWWNTWQLWLNKDGFFGYEAAFRAMKAVLKAD
LADYDMSDVEGVGRYQSRQEIRVGYRIRQQVAFEQLDPRTDILLQSLTRLSVIRHALTMVAILIVREVV
VEFTLVVAQEDVAMGHVECVLADAFDFTAKDADIGPVGIRTPDPVLGDGTVLKDAVLGKIDLANTVA
DFRYKMLIDGGRELGVSWLSLQNMLYKRIWLPPAQAALKNLEPITFFLGHPELNLHIFPALYAGISKAR
ADKLMAGALIEKILEQIAAFADPEATTHQSKKLRHFKYYGEEVDPVYLAYILDMFALVATQVTVSDRLL
KDRYVLSLKVFRGFTSLLWLSAQIGEKGPAATIITVMTIAGLGALVLFIILL-RAVVLVETLYYKYVY--
MLGYQAAKDVADADPEHFLSLAHASRDVEWIEIIPAFFYYFKTATKNLEKNTALAREFFGLFKERL---
EVLVVPPDRVEALAQFVALAGTLLTSARA-PGIIACG-
CEPNAAAVLEADQDWNLHRSMGITLAFYDARESVKGWFDKGLHKEVAETGLSEAGALTSPFHAGST
AKRCVPAIHRVLDLGEVEAALIEIDSAVVVADEEQTLADKPTTRENSVGAVGGIVLAGTMNMGANLD
AVERGGNIIIELEEEHYSAVNGKKADGKVTDKVLMVLSAAYFPVLRRMASANALDKGTFRLLIDAPDD
ADNQDHGGGLTIPAPSVHAETAPLYAESEIERIAWIAPIHLLGTLDAVLGFEPDQYSFISMMIDASLDIM
ETAEV--DYHVLEKGARPPGFRVILIKGLHIRLLAGICTIVIVGSAGRSEITAYIQG-
AVPITLNKQTFLPAFAASSCHAIDIFSTMLRPPERSAIEMLILETVAEL-
LEVTRIENDKLERMDALIPGLDVASLLDLVSKSSDFADIVTIDRAIAAILSVAAEAVVLREMLLTLAEIAVT
ALALAMALLLMLTRDQGLHQKETARETQLKELIQGGVVLQVKAADRMMLASTADKVRLDTDEGTTS
AFASLNLATVSDGFYVLSSAVLQLSSDLMMIVAVARVLADKETKNALVVRHPEQVKTQGTYAEGSPG
MVLQTLTAAIPPLAIKPEDVELLWTLVAALWQVKAHLANAARPHMERVLILILALSLITVLLQSEGGGIG

GG--
GPPAKMLAAFSILTIKAKAGEEVRKVTRRVMDLNSRLGRPEEAVNPEFRGAELEQSKYEAENVEVDTM
ETIRRFVDIPFLVSTRTQHNESEDRGARMVLDVTRVRFIDL-
VTKRSEIILAARAETDGIIAREMGKDLAALHPEAAALGKIAALQHFIAIQDAHISNYVTAQIHAVLGMILL
VLFIQETRISKVVWPTRREVTMVFIMAALAVFFLVIGLAVQIIFMLSGISWNALTTIVLGEGIMVVRQTST
YPQDS-
IGILQNTYADLICSGRVAKADIENSLHIVVAGRVARGTWYVIYLYEITQAQSQSGIMQRAFRQMTLSQ
RLIDGRMEVKIKVVTSDRYA-
TYEIKGGQHRKGTRYNRVKIAHNIPVQVLPRMKMAFGPPNLVLVKTFGAVVQKRFAQALTHIRHGAV
TIGPMEVSSPYISNKKARHAPGDAVVDLKPDNIVRSVAVVPELIWARENEDNGAVHDKYVKTTHKLA
AKRKKAVDKAQYDHRNQVELSFIGLAVKVLPDKA--

RMRPQLNLGGANDKKIVKCLNNNLDDVEYGKLGRRKIKPITIKSVYDVEHRRATELVKLMPYKDDVES
RKSQFAEK--
ALFEPLKAFMMLFGFIGRAIYALYLRVIRRTILLVKTKIVVNASTVLSAIKIIGVSIRLRHKSTNVAETNHHIK
TIEARARMTLAGTGKVKLNLSPGKIGMKGGRIKGYMPYNNFNIFDGLASGRRAKVKELAKLKEVDKTIS
VEGIRQKQQGGRTPKANLSNAI-
NSRVGVDIVGENEQEESNPRANAEMGQIFAKIDARVFHHVGALIGHALTAVLAVVAIWGIGRAHNLL
ADKLKVNAGIEMHSAA-VEMAVAAA-FEENGGEA-IVVDISEAYLDALAITAMSVDVCRLRRRGLA--
----
RHGIRAKGSYGKERAMMQITPTKPVKIWVPVFVNETELLAPIRAFAESTGDGVTVVKLTTDVKVTMFE
KAVASIAVTGKATSIVYTVLEKVTELMKDIIYTQRAKAHYGRGRLRSHVVTTTKQRRHLSMIKRTVVAD
PSMDAARLTDGEVSVITGATMAFVMSEQDAV--
GDVDLHFHDEPQREADVGIGGPETFLTTEQPRVLALIFLRAYLIHIVEIGTVGGFSDAATIYDVLRAMQ
NKLDRKLSADIAKTRMCLTDNIADMGVTEVAAKRDAAGDKILSAFWINGVTANADIILLVANLECPCV
PENINCAIRLFYQRITLQSKERNGDVSVSIVPAANQIEAAEGTVIYSIKGKEPGTGQMKLTIEGISRSIVKS
LAVQLFQVDTFLDDTWIATWLRAAGQVVFMHADDGKEAVDYVVERDTELEFAPQKDIVARKIRIYTP
AAPSAVVIWDPAIEREDKAEAQGTILKIYGPNLQDINCFTDIGAEPKIESLKAWDEKEKYVVHRDDIPED
I-
TLVGKSSKVNKEFAIPDVAVCAPSKPGVVESIDRGEPKAGTCGEVANMAYPQESLVNAVSSANVQKN
ISMVLAGHPVGEMGISREIERHLETNMCEIFVDKPDVDVALDFSTLTLTPLRVGLDPPNARVQIDGKES
RKLMAVLDGEADLQRHPKVSQATGLTHNAGVKARLPDDSLILDGVMHGDISFTITDLPEGRV-
VTPVFNAVKAQLKIDKVLLKAFHVVTLSFSNDKGQEHLIKLRLNALKKSTRHYEMLLKIRETECAESPHA
ISVGKVLKIDALLQTICEAHYKEIEPLLADDLTALGVMGQQGLLHIVPRLMILAVLHVLGLAAAFQVVR
W-LQQVAIVTTQTLGLRHRTRDWSFIAISEVVGNTFFARTKDDDTEQRIHARNPV-VLL-YGKFPMI-
ISPSGLGLQSTAPSAQDRENTEELILSKERLQDEMGYYVLRILLVFVDEVILAYGSGNDPIKFLVAEQAVT
GRQNPGMVTLTRRTFPLELQRNATFVMVFEDCTMKNLSHKKMHMNLVVIKSKGWIVNVSTKILISD
QAIASYAEDAGAHTQPSESALAVITSRVMAPIVLVVSQKPTAPSKTLFLEALSLYDEVTATVLNKPKKYY
IFEGDVVGKLGLIKGRLLKGVLLVMPNAFRLACVAKAVRMAIAW----------------------------
---------------DFRVRNHDVSTKRSRPYKAHPKFLHKADIDKGLIASGVDTLA
>GCA_003664205
AVIKPVTQDKYAPVLLIRQQEVATVTGAGSPNLRAKTVYRKSGLQRKTSTKWVAAPIYY-
GVLKMGNIVQLVSISVDNRSVCTVAQVVEQEVVVHGLVALDAVKRKIALEEMDGMTTEDPAKVAEF
EEELDLKKFRDADARADMLPVMLGLTLQIRFDPHEPVVHDLPIPVIYEVI-

GIMADKGGIEVDDCAISNAVSDVDPIAYVSPIDLAAVAGEQMAFIDQEAAVAAAREAIELEELALEAGI
LVVGISPLLLPV-FGEEGDV-MSFANRSRTLESIVALAVAYPALSRARIDG-IASAM-
MERAQAAALSEKRGTAEEIRIVKRMQRSSGLADDPELVILEDAAEGNSSWAGQIYDDPISGSSESDSA
GNIVHAANKRQNFARSITHLRKVEISDKAAAPLMRGVKHNAKSRSARKLNDEIAVLNFPNVPAGIAIR
AAAGKSEDTLLHDATEREEILLIVVRWHVTWKTAAIVFLTFTAGDIVGLIDMGGIAAVGKPDGLLNRHH
KLNGSLIEQRPIIEKLTLRLRASLKEKLGAEQIIIFVVKVQGDLVADAKVQMG-
GPVGAVVAIQGDVIFVRRHKQTVITDQESTV-VLKGVAEGPADAQALLRIRVTHTLVELSVLLIPE-
PVLVESVTIREGVGKALQIIQLDVYLLRDMGTGTETLAESGSRELIREVLAVDPITGVRLAYGKIKERMGK
KVGLKDLD-----
LSILANVTEPRMENMPAGVCRNFIVAGPRAAVGMLFLLLDDGGKLYAIDGDLRELLKSVKSLISLVDAK
APKVLSAGYAEDRLKIRFADCEVLASEAGLYAYKEERRELLRMVPVYIILEAKRRQLIIFALSLKGGHRRT
MMALGVMIAAAIIESQADKGPIHHEEPGILLDDAEVVGSSVMISRQAALDIKQFTKVDVVVIFPEFQEK
MVVSLKFFCTDVAIFVIALITTVGDMMSREPEHALEEGAGYQKRIRGHSVLFALAVTFDRRNTGRDAT
ADLFLDLGLAQPRYGILYVNQPKKKQMPSEPDSDRVDGFELVHAIKEDEGSNHELEMPEAMELAVEY
DASINVSAGVLALSGGMVAVEKVDSKRKVALAATRWNISRLKIQDVHLSGAAPFRSCGNIELADLSAA
LSDKKLWFLFLPGASNAELEEEKLDGAGASTADAADDKQIAILLVKPVMVVIQDSNAASLVANGGVD
EWGTMAYKKHYAFAARMRLHMRTID------
QMAPNKQKSEKVKTVKLEGDTRFRQLAVDGIKEQMP-LHALNPDFAV-
LPLLVTPAVGSVAADADVLGAEVPVDASL-
RELQKSQKGAALPISGVRLLAMRAARGMIEEDIMVAGKIAA-
LLKIRALETVVDIVLRNDSEATLPWRLSVAEKLEADTKRGGGFAARPFKSKPSLCDTQAHAKVADPIQEI
AAGYVAAEDRFVYPVEGIAIQLILTGPHSRVGTAKRAGEGHLHEMAKELLAKAVLMLRNEPDTNTEGL
LNYLTTNQPTDLLFEDCLDVIIDIRRMSVV---
VHLNTV-
HVVVEGVCEVGDYEIMAAAFPSGLVQVERVDDRTGHERTLMWMYGAMEELWLLSVRWADWVFD
TPIGAIILHVYAQSINRAFSLKRLQLDGWPKEAARWWNTWQLWLNKDGFFGYAAAFRAVKQVLKAT
LADFEMSDIEEVGRYLSRQEIRAGYRIRENVEFARLDPRTDILLQAQTRLSIIRHALTVVAILVLRDVAPEF
T-
VVAYQDVARGHVECVLAESFDFTEKNAHIGPVGLRTPETIFGDGTVMKEAVLGKIDLANTVADFRYLV
LVDGGRELGVEWLSIQNMLYARIWLPPAQAALKNLLPIKFFLQHPELDLAGDWSQFNGISKARADKL
MAGALVEEILEGIARFADPEATTHQSKKAKHFKYYGEDVDPVHLAYILDIFALVSTQVTISDRLLKDRYV
LNLKVFRALTSLWWLSAQLSDKGPAATVITVGTISGLGALVLFIILL-RDSVLVETLYHRYVY--
MRGYSAAKDVADADPEHFLVLAAASRDLEWIEIVPAFFYYFKTAS-
FEPFNTAVAKEFFGLFEERLRIIDVLVVPPDRVEALTEFVALAGTLITSAEA-
PGVIATGLCEPNAAAVLEAERDWNLHRSMGITLAFFEARDSVKGYFDKGLHKGVAEISLKEAGALTTH
FHCNAEAKRCVPAAHRVLDLGEIEEALIEIDSAVTVADEEQTLADKPTTRDKTVGAIGGVVLAGTMNM
GANLDSVERGGNIIIELEEEQYSAVNGKKADGKLTDKVLMVLSLSYFQVIERPASANALDRGVFHLLIEA
PEDADNLDHGKFMTIPAPSVHTETAPLYAESDIDKIAWIAPLHLLGTLAAVL-
FEPDQYSFISMMIEASLEITETAEV--
DYHVLEKGSRPPAFRVVLIKGLHIRLLSAVGTILVVGSAGRAEITAYIQGQAVPITLRKHTFLPAFSPSSCK
VVDIMSTMLRPPERSTVEMLILETVAEI-
LEATRGENEKLERMKMLLPGLDMANLLPLVAAASDFSDIVTIDAAITAILSVAAESVVLREMLLTKAEIAI
TALALALALLLMLTADKGLTQKWRAREAQMKELIQSGAVLQVHADDAMVLAATADKVRLDTDEGTT
SAFASLNLATVSDGFYVLSSAVLQMSADLMMIVAVARVLADKESKNALVVRHPEQVKTQPTYAEGSP

GMVLQTLTQCIPPLAVKADEVELLWTLVAALWRAKAHLASAARPRLERVLILLLALGLIVVLMQSEGG
GMGGG-
MGAAAKILACFSILTLKASVGDEVRKVTRRVMDLNSRLGRPEEAVNPEFKGEELDQTKYEAENVEVDC
LETIRRFVDIPFLVSIRTQHNESEDRGARLVLDVTRVRFIDL-
VTKKSDVILVERVETDGIIARQMAKDMQSLHPEAAALGKVSSLQHFIALNDGHISNYVTAGVGSVLG
MLLLVLFIQQVRVAKVVWPTRREVTMVFIMAALAVFFLVIGLLVNLVFMIGGISPLQITTLVLGEGVMA
VRQVSTFPSES-
ITILQNTYADLICAGRVTKADIELSLHIVVAARVARGTWYVIYHYEISQAQGPSGIMQRAFRQATLSQSL
IDGRMEVKIKVVTSDRYA-
TYEIKGGQHRKGTRYNRVKVGHNIPVDVLARIKMAFGDPNLVLVKTFGAVVQKRFAQQLTHIRHGAV
TIGPMEVSSPYIQSKKARHAPGDSVVDVKPDNIVRSVAVVPELIWARENEDQSAVHEKYVKTTHKIAA
KRSSAVDKAQYDKRNQVHLSFIGLAVKVLPEKAIVQATAGTLLPTESVAVTENLKGIAATIRLDPMEKA
LIALIKRMRPQLNLQGKTDNKVVKCLNNNLDDVEWGKLGRRKIKPITIKSVYDVEHKRATELVKLMPYK
DDVEGRKSQFAEK--
ALFEPLKAFMMLFGFIARSVYALSLRVVRRTILLVKTKIVVNASTVLSAIKIIGVSIRLRHKSTNVADTKHHI
KTIEARARMTLAGAPAVKLHLRPGRVGKKGGRIKGYMPYKNFNIFDGLASGRRAKVKELAKLKKVDKTI
TVDGIRTRQQGGRLPQAELSNAL-
NTRVGVDIVGENEQEASNPRANAEMGQIFAAIDARVYSLVAALIGHALVAVLAVCAITGIGRAHALLA
VKLKINAGIELHSAA-VAAAVAAA-YAQPGGETAAIADVSEGDLGALAVIEESLDVCRLRRRQLG-
LKKTKKEAYMSLTCFAGKGDKVKIVEATQTQAFEIVATRTAERHGIKAKGTYGKERAMMQITPTKPVKV
WVPVFVNDDELLAPIRAVAETNGDGVAVVKLTTDIKVTMFEKGVASLAISGKATTLVYTVLEKVTELSK
DIIYEQRAKAHYGRGRLRSHVVTTTKQKRHLSMIKRTVVASPKMDAARLVDGEVSVVTGATMAFVMS
EQDAV--
GDVDMHFHEEPRREAQLGIAGPETFLSTEQPRALALLFLREYLIHIVDIDTTGNFSDAATIFDILRAMQA
KLDRKLSADIAKVRMCLADDVADMGVTDLAARRDSAGDSILSAYWINGVPAHADIILLVANLECHTA
PENIDCAVRLFYQRITLQAKERNGDVSVALVPAQNQIEAAEGTVIYTIKGENPGTSQMKLNVEGISRSI
VKSLAVQLFQVDVFLNDTWIATLLRAAQNVVFVDAREGKEAVDHVVERDTELEFAPQKDILGRKIRVY
TPAAPSAVVIWDPAIERADKADAQGTILMIYGPDLQDINCFTDIGAEPKIESLKSWDEKEKYVVHRDDI
PPDI-
TLVGKSSKVNKEFGIPDVAVCAPAKPGVTESIDRGEPKAGSCGEVANLPYPQDSLVNRVSSANVQKNI
SMVLGGHPVGEMGISRAVERHLETNMCQIFVDKSDVDVALDFYKLSLTALRVGLDKPNAKVQIDGKE
SRKLMAVRDGEAELQRHPKVSEESGTR-KAGEKANLPDDTLIYDGVQHGDLKFTSTDLPEGRV-
VTPVVMAVKAQLEIDKVMLKTFHVVTLSFSNDKGEEHLIKLRLNALKKSTRHYEMLLKIRDTECAQSPH
AIPVGKILKIDSLLQTICQAHYTEIEPLLADDLTALGVMGDQGLLHVVPRLMILAVLRLIQLEALALIPKW
ALQQRALITDQTMGLSTRRRDWSF-AISEVVGNTFFARTKDDDTEQRLHARNPI-VIL-YGKLGMV-
ISPSGLRLQSTAPSDQQHGNETKLILSRERLQDAMYYYILKVLLVFVDEMILAYGSGDKPIKFLVANQAI
TGRANPQLVCLTRRTFPLDLQRDATFVLVLEDSTMKNLSHKKMHMNLVLVKPKSWVENVSTKILISD
QAIASYAEDAGAHTQPSESALAVITSRVIAPIVLVVSQKPTAPSKTLFLEALNLYDEITATVQKRPKRYFIF
EGDVISVLEGLDAAVLLGMQRPQPDRLRRAITAAAIRLAIAIRASNIVASLENGCINFSRAVSFAA-HY-
----------------DFRVRNHKTSTKRDRPYKGHPKFLHKAEEEKALVAEGVDTLA
>GCA_003789055
AVIKPVTLNAYAPVLMIRQTQVATVSGAGSPSLRPKTVYAKSGLQRKTSTKWVKKNFYY-
GVLKMANLVQLVSISVDNRSVCQVAQVVEQEVVVRGLVALEAVKRKIALDEISGMTSEDPAKVIVYA
GELELKRFREADDRADMLPVLLGLTLKIRFDPHQPLVHDLPVPVIFEVV-

GIMAQRGTIEVDDCAISTAVSDVDPIAYVSPIDLEAVAGEKMAYIDQEAAVAQAKTDIALEELALESTV
LVVGI-PLLIPI-FADEG-T-MSFANRSRKLASIVALAVSAVSITRARIE-AMVSAM-
MRAARASALAEKRGTAEEIRIVKRMQKAAGLADNPDLLILEDAAEGNAQWAGQIYDDPISGSSESDS
AGNIVVAANKRQNYARSITFLRRVEISDKASAPLMRGVKHNAKSRSSRKLGDTIASLNFPNVPEGIALR
ADMGKPEDMLLHDATEGEEILLIIVRWHVTWKTAAFIVLTLRAGDVVGLIELGGIAAVGKPDGLLNRH
HKLNGSLIEQKRITDKLTLRLRAALKQKLGAEQVIILVVKVAGDIVAAAKVQM---
AVGTVVAIQGPTIFVRRHRQTVVTDSEATVEILRGVAEGPADAQALLRIRVEHTIVKMSVLLIPE-
PVLVESVSIREGVGKALQIIQLDVYLLRDMGTGEDTLAKSRSRELIRDVLMIDPITGVRLAFGRLRERVGK
ALTLKELEALF--
LAITRFVTEPRMETLPAGVCRTHVRAGPRAAVGTLFLLLDEAGRLYAISGDLRELTKSIKSLIGLIDSARPK
VLSASYATERLRVRYADVTVLSSDVDLYALAEERKEILRMVPVYITTAAKRRQLVILALSLKGGHRRARA
ALGLLLAAAIRESQAEQGPVHHEAPAILLDDAEIVGSEMMISRQSALDLKQVLKVDVIVVFPEFQEKM
VVSLRFFCTEVAIFVIALIVTVGAMLSREPAHAQESGAGFQK----ASILFALEATFERANARDA--
PDLLMSLALLPPRYGLFYTTQPKKKQMPEEPDEEKVDGFDLIHVVKEDGRSNHELEMPEAMALAVEAS
ETINVSAGVLAIDGAMVSVERVDSKRKMAVAVTKWTIAPRAIRDLPLSGASPVRALG-
AEHAAVSAALGAKPIA-
LYLPGATNAELEEAKLDGAGASSAEAAADRQAAILMVNPVLVVIQDSNAAGLITNGGVDEWGTMAY
KKHYAFAARMRLHMRTID----GVRMEPNKQKSEKVKTVRLEGDTRFRQLAVEGVKEQMP-
LHALNPDFVV-LAVLVSPAVGAVAAASDLVGARVPCGASL-
RELQKSQTGAALPISAVRVLGMGAARAMIEEDIMVAGKIAA-
LLHIGALETVAGIVLRNDSEATRPWRLSVAERLEADTRRAAGFAARPFKSKPSLCDTQAHAHVAEPIQE
IAAGYVAAEERFVFPVEDIPIQLILASTHSRVGAAKRAGEGHLHEMAKELVAKADLLVKNAPASNTEGL
LDYLTTSQPTDLLFTDCLDIIIDLRRMSIV--
VQLALV-HVVIPGARDLADRTIMAANA-
SGLVQVERVDERTGTERTKMWMYGAMEELWLMSVKWADWVVKTTIGAIVLHVYAQTINRAFSLKR
LQLEGWPPEADRWWNTWQLWLNKDGFFGYAAAFRA-
AAILKAELYDYDMSDTQGVGRFQSRQELRVGYRIRQQVAFARLDERRDILLQSRTRLAVLRHALAIVAI
LILRGLVSEFSLVVAEEDVAMGHVECVLAEAFDFTAKNAHIGPVGLRTPEPVLGDGTVFRAAVLGKVD
LENTVAEFRYRLLTDGGRRLGVTW----
NMLYARIWLPPAAAALKNLTPISFFLGHPELNLKQFDGDYD-
ISKARAQRLMAGALVEEILERVARFADPEATTHQSRKARHFKYYGEEVDPVHLAYVLALFALVATQMT
VSDRLLKDRYVLNMRVFRGFTSLLLLSLQLGEAGPAATVITVGTIAGLGALVLFIILL-
RDSVLVETLYYRYTYH-MRGYEAAKDVADAGPEHLLSLAHVTRDVEWIAIIPAFFYYFDTAT-
FEPFNTAVAREFFGLFRERLRIAEVLAVPPVRVEDLVELVALAGTYPTSAAAAPGVIACGLCEPNAAGVL
EADQDWNLHRHMGITLAFFEARVSVKGWFDKGLHKEVAETGLREAGGFTTPFHAGETAKKCIPAIHR
VLDLGDIEAALIEIDSAVTVAGEEQTLADQPTTREKAVNAIGGVVLAGTMNMGSNLEQVERSGNIIIEL
EEDQYHAVNGKKANGKLTDKVLMVLSAALFPVLARRASATALDRGAFDLLIEAPDDADNMDHGRTL
SIPAASVHAETHPLYAESEIERIAWIAPLHLLGTLDAVLGFEPDQYSFMSMKISASLGIVETAEVLDDYHV
LEKGGRPPDFRIILIKGLHIRVLAGLATIIVVGSAGRTEVTAYIAERAVPISLRRQTFLPAFAPSSCHVIDIFS
TMLRPPERSAVEMLILETVAEL-LAVTRAETGKLERL-
LLVPGLDLAARLALAALASDFAVIVTIDAAVVAILSVAAEAVVLRELLLSLAEIAVTALALALALLLMLTRD
RGLPQREAARESQMKELIQGGAVLHVHAGERMVLAATADKVRLDTDEGTTSAFASLNLATVSDGFY
VLSSAVLQLSSDLMMIVSVARVLADKEMKNALVVRHPEQVKTQATYAEGSPGMVLQTLTQAIPPLAV
KPESIELLWTLVHALWRVKSHLASAARPHAERVLILLLLALTLIVVLMQSEGGG-MGG--

GAAAKILAAFSLLTIKASVGDEVRKVTRRVMDLNSRLGRPEETVNPEFRGAELEQTKFEAENVEVDTME
TIRRFVDIPFLVSTRTQHNESEDRGARMVLDVSRVRFIDL-
VTKKSEIILAARAETDGIISRELQKDLQALHPEAAALGKIAALQHFISLNDGHISNYVTADIHSVLGMLLL
VLFISQVRVSKVVWPTRREVTMVFLLASVAIFFFVIGLGIQIVFMLGGISYQQLTTLVLGEGVMVVRQTS
TFPADS-
IGILQNTYAELICAGRVTKADIEKSLHIVVGARVARGTWYVIYHYEISQAQGPAGILQHAFRQATLSKSLI
DGRMEVKIKVVSSDRYA-
TYEIKGGQHRKGTRFLRVKIGHNIPVEILPRLKLAFGDPNLILVKTFGAVVQKRFAQPLTHGRHGAVTIG
PMEVASPYITNKKARHAPGDSVVELKPDNIVRSVAVVPELIWARENEDQGAVHDKYVKTTHKTAAKR
NRAVDKAQYDHRNQVHLSFIGLAVKVLPERAIV-
ATAGLLLPTEAVSVTEGLRGIAATIRIDPMEKALIAYIKRMKPQLNLAGKTDKRIVKCLNNGLDDVEWG
KLGRRRIKPITIKSVYDVEHKRATELVKLLPYKDDVEGRLSQFAEKNRALFEPLRAFMMLFNYIARAVYAL
SLRVVRRTILLVKTRIVVNANTVLSAIKIIGVSIRLRHKTTNVADTNHYIKTIEARARMTLAGAGKVKLNLR
PGRVGKKGGRIKGYMPYKNYNIFDGLDSGRRAKVAELAKIKKVDKEVSVDGIRQRQQGGRTPKAHLS
NAL-
NTRVDVEIVGENEQDESNPRANAEMGQIFAAIDARVYHLVAALIGHALEAILAVAAIMGVGRAHALL
ALKLRINAGIGFHSAA-VERAVAAA--
LKKTKKEAYMKLTCFSGKGDKVKIVEATQTQAYEIVATRTAERHGISAKGTYGKERAMMQILPTKPVKV
WVPIFVNEDELLAPIRAFAETNGDGVDVVKLTTDVKVTMFEKAVASLAVAGRSSSIVYTVLEKVTELMK
DIIYEQRSKAHYGRGRLRSHVVTNTKQKRHLSMMRRTVVANPRMEAARLTDGEVSVITGATVAFVM
SEQDAV--
GDVDLHYHEEPRREAELGIAGPETFLATEHPRALVLVLLREYLIHIVEIETVGGYSDAATIYDILRPLQSKL
DRKLSADIAKVRMVLHDDLPDMGVRAEAPRREAAGDPILSAYWINGVPANADIILLIANLESATAPENI
DCAVRLFYQRITLQAKERGGDVSVLVVKAQNQIEAAEGTVIYTIKGKQPGTVQMKLNVESISRSIVKSL
AVQLFQVEMFLDDTWIATLLRAAAKVVYLDAQSGREAVDHVVERDTDMIKAPQKDILPRKIRIYTPAA
PSAVVIWDPAIEREDKAEAQGTILKIYGPNLQDISCFTDIGAEPKIESLKAWDEKEKYVVHRDDIPDDI-
TMVGKSSRVNKEFGIPDVGVVAPAKPGVTESIDAGEPKAGSCGEVANMAYPQESLVNAVSSANVQK
NISMVLGGHPVGEMGIAREVERHLETNMCEIFVDKPDVDVALDFYRLALTALRVGLDSPNAKVEIDGK
ESRKLMAVRDGEAALQRHPKVSEVAGTGA-AGVRARLAEDALIHDGVHHGDIGFTVTDLPPGRV-
VTPVTINVKQQLKIDKVILKTFHVVTLSFSNNKGEDHLIKLRLNALKKNTRHYEMLLKIRETECAESPHAI
PVGKILKIDALLQTICIAHYTAIEPLLADDLAALGVMGDQGLLHVVPRLMILAVMHLWGLEAALELPR
WGLMARIGLTDLTLGLAVRDRHFTF-AISEVVGNTFFAMTKDDDTRDRLHARNPI-VLL-
WGKFAMV-
ISPSGLLLASTPPSAMRHGNEGDLILSTERLKDEMAYYVLQVLLVFVDEVILAYGSGDRPLKFLVADQA
VAGRENPGLVCLTRRTFPLDLQRDATFVLVFEDSTMKNLSHKKMHMNLIIIKSKGWVVNVSTKILISD
QAIASYAEDAGAHTQPSESALAVITSRVIAPIVLVVSQKPTAPSKTCFLEALNLYDEITATVQNAPKRYYI
FEGDLISVLGTIDAAVLKGLQRPQPDRMRRAVIAAAIRLAIAARVSNLLHALETGTINFARSITFML-
SYAAVTL--DAPMGPDAIAFRVRNHKTSTKKDRPYKGHTKFLHKAEAEKALVAEGVDTLA
>GCA_004000435
AVIKPFLYTKYAPVLMIRQQQVATVSGAGSPSLRAKTVYAKSGLQRKTETKWVAKPLYY-
GVVKSGNLVQLVSISVDNRAVCTVAQVVEQEVVVHGLVALEAVKRKIALEEINGMTSEDPAKVIQY-
DELELKRFRDADDRADLLPVLLGLTLKVKFDPHQPVVHDLPAPVIFEVV-
GIMAQKGTIEVNDCAISTALSDVDPIAYVSPIDLEAVAGDEMAYIDQESAVSAAKEAISLEELALESVILV
VGI-PLLIPV-FRDDADT-MSFANRSRSLESIVDLAVSAEHRNRARIG-NIASAM-

MDRAKAEALCEKRGTAEEIRIVKRMSRSAGLADDPELVIMEDAAEGNSAWAGQIYDDPISGSSESDSA
GNIVHAANARQNYERSITFLRKVEISDKAAAPLMRGVKHNSKSRSSRKLNDAIADLNFPNVPEGIAIRA
AGGRTTDTLLHDATEGEEILLIIVRWHVTWKTAILIVLVITAGDIVGVIELGGIAAVGKPDGLLNRHHKM
NGSLIEQRRVIEKLTLRLRAALKQKLGKEQVIIFVVKVAGDLVAEATVQMG--
DVGTVVAIQGDVIFVRRHKQTVITDQEATVEILRGVAEGPADAQALLRIRVDHTLVDVSVLLIPRVPVL
VDSVTIREGVGKALQIIQLDVYLLRDMGTGEETLAKSASRELIREILQVDAITGVRLAYGDLRERTAGGLK
LKELD-----
LSIRVGVTEPRMENLAEGVRRDFIEAGPRAAVGTLFLMLDEAGRIWSIDGDLRENPKSIKSLIGLVDAAK
PKVLSAGYATTRLRVRYADCMVLDAEANLYAYREERRELLRMVPVYIIVAAKRRQLVIFALSLKGGHRR
ARAALGVLIAAAILESQAEMGEVNHAAPVVLLDDAEIVGSEMMISRQSALDLKAILKVDIIVLFPEFQEK
MVVSLKFFCTDVAIFVIALIVTVGEMLSREPSHAQEAGAGFAKRIRGSAVLFALAATFDRRNAGVDSLG
DLFLGLGLAPPRYGLFYTTQPKRKQMPAEPDAERVDGFELVHAIKADSADNHVLEMPEAMALAVEA
NGSINLTGGVLGMNGGMAAIEKVDSKRKLALAATKWNISPVQILDISLSGASPVRALN-
IELAQLTAALGDKTVWFLFLPGANNAELEEAKLDGAGASSVDVAEDRQQGILLVAPVIVVIQDSNAAS
LIANGGVEEWGTMAYKKHYAFAARMRLHMRTIDAIEIGIALEPNKQKAEKVKTVKLEGDTRFRQLAVE
GVKEQMP-LHALNPDFAV-LAVLVSPAVGRVAAEAAVLGAEVPCDASL-
RELQKSQKGDALPISAIRVLGMRAARAMIDEDIMVAGKVAADLLKIRALQTIIAIVLRNDSEATLPWRLS
VAERLEADTKRASGFAARPFASKPSLCNTQAHAKAAEPIQEIAAGYVATAQRFVFPVEGLAIQLFMSN
PLSRIGLQKRAGEGHLHEMAKELLGKAVLMVLNEPDTNTEGLFNYLTTHQPTDLLFPDCLDVIIDLRR
MSVI--IQLNTV-
HVVVAGVRDVGSYQIMAAAAPSGLVQVERVDDRTGRERTMMWMYGAMEELWLMSVTWADWVI
DVPIGAIVLHVYAQVINRAFSLTRLQLEGWPMEADRWWNTWQLWLNKDGFFGYEAAFKE-
LAILKSDLYDYELKDVDDVGRMLSRQELRVGYRIREEVEFEKLDPRTDILLQSKTRLSVIRQALALVAILIV
RTIVSEFS-
VVAEEDVAMGHVECVLADMFSFTAKDAHIGPVGLRTPDPVFGDGTVFKAAVLGKIDLANTVAEFRYK
FIIDGGRELGVDWIGLVNMLYPRIWIPPAPAALKDLLPIKFFLSHPELDLSVLREVFDGISKARAEQLMA
GALVEQIIDNVAAFADPEATTHQSHKEKHFKYYGEEVDPVHLAYILDIFALVATQVTVSDRLLKDRYVL
NMRIFRGITSMLWMSLQLGNKGPSATIITVGSVAGLGALVIFIILL-RDEVLVETLYHRYVY-
EMRGYSAAKDVADAQPEHFLSLAHVSRDVEWIEVIPAFFYYFNTAG-
FEPFNTAKAREFFGLFEERLRVIDVLVVPRERVEALSEFVALAGTLTTSAQALTGVITTGFCEPNAAAVLE
ADQDWNLHRTMGITLAFFKAQVSVNGWFDKGLHKDVAEVGLREAGAFTTPFMAAVTVKRCVPAV
HRTLDLGDIDAALIEIDSAVVVADEEQTLADKPTTRDKAVNAVGGVVLAGTMNMGANLEQVERGN
NIIIELEEEQYHGVNGKKADGKLTDKVLMVLSAAFFPVLARFASANALDRGVFELLIEAPDDADNLDH
GRELSIPAATILTETAPLYAESEIERIAWVAPIHLLGTLDAVLGFEPDQYSFISMMIEASLDIMETAEVMED
YHVLEKGGRPPDFRIVLIRGLHIRKMAGIGTIIVVGSAGRTEITAYIQG-
AVPITLKRVTFLPAFAPSSCIAVDIFSTILRPPERSAVVTMILETVAEL-
LEVTRIENDKLERLLLLLPGLDVAALLALLAAAKDFAVIVTIDAAITAILSVAAEAVVLRELLLDLAEIAVDA
LALALALLLMLTPDTGLHQKERPREAQMKELIQGGVVLQVHAADAMVLAATADKVRLSTDEGTTSAF
ASLNLATVSDGFYVLSSAVLQLSSDLMMIVSVARVLADKETKNALVVRHPEQVKTQPTYAEGSPGMV
LQTLTQCIPPLAIKPQEIELLWTLVAALWRVKAHLASATRPHNERILVLMLALALIVVLMQSEGGGMG
GG-
LSGAAKGLAAFSILTIKAKVGDEVRKVTRRVMDLSSRLGRPEETVNPEFRGAELEQSKFEAENVEVDTIE
TIRRFVDIPFLVSTRTQHNESEDRGARMVLDVTRVRFIDL-
VTKKSEIILAARVETDGIIAREMQKDLQALHPEAAALGKVAMLQHFVSINDGHISNYVTADIHSVLGML

LLVLFIQETRIAKVVWPTRREVTMVFLLAVTAIFFLVIGLAVQIVFMLGGISYQQITTLVLGEGVMGVRQ
TSTFPADS-
IGIMQNTYADLICSGRVAKADIEQSLHIVVGARVARGTWYVIYLYEISQAQSESGIMQRAFRQRTLSQS
LIDGRMEVKIKVVTSDRYA-
TYEIKGGQHRKGTRYNRVKVGHNIPVDVLPRLKMAFTPPNLVLVKTFGAVVQKRFAQPLTHMRHGA
VTIGPMEVASPYIRNKKARHAPGDSVVDLKPDNILRSVAVVPELIWARENEDNGAVHDKYTKTTHKL
QAKRTRAVDKAQYDKRNQVAMSFIGLAVKVLPDKAIVHATAGSLMPTASVAVTEGMKGIAATIRLDP
MEKALIAYIKRMRPQLNLAGKTDKKIVKCLNNNLDDVEYGKLGRRRIKPLTIKSVYDVEHREATELVKL
MPYKDDVEGRKSQFAEK--
ALFEPLKAFMMIFGFIARAIYALSLRVVRRTILLAKCRIVVAAATVLSAIKVIGVSIRLRHKSTNVAETKHYI
KTIENRARMTLAGAGQVKLHLREGRVGKKAGRIKGYMPYKNFNIFDGLASGRRAKVKEVAKLKKVDK
EIEVTGIRMKQQGGRTPKADLSNAI-
NTRVDVEIVGENEQAESNPRANAEMGQIFAAIDARVYSHVGALIGHALAAVLAMADIWGIGRAHAL
LAVKFRMNAGIEMHSAA-VEVAVAAA-YAKSAGEA-
VIADVSEGDLGAVSITKESLTVCRLRRRQLSELKKTKKDAYMSLTCFAGKGDRVKIIDASQTQAYEIVAT
RTAERHGIKAKGTYGKERAMMQIVPTKPVKVWVPVFVTEAELLAPIRAFAETNGDGVTVVKLTKDIKV
TMFEKAVASLAVTGKSTSVVYTVLEKVTELSKDIIYEQRSKAHYGRGRLRSHVVTTTKQKRHLSMIRRTV
VADPRMEAARLTDGEVSVITGATMAFVMSEQDAV--
GDVDLHFHEEPRREAAVGIAGPETFLTTAQPRLLAVIFLKEYLIHIIDIGTVGGFSTAATIFDILRAMQNKL
DRKLSADIAKVRMVLHDDVADMGVTELAPRRETAGDAILSAYWINGVPANADIILLVANLEAPCVPE
NIDCAVRLFYQRITLQAKERGGDVSVHVVPAQNQIEAADGTVIYSTKGPQPGTAQMGLNVEGISKSIV
KSFAVQLFQVDLFLNDTWIATLLRAAEDVVFVDAKEGKEAVDHVVERDTELEMAPQKDIVARKIRIFTP
AAPSAVVIWDPAIEREDKADAQGTILKIYGPNLQDISCFTDIGSEPKIESLKAWDEKEKYVVHRDDIPEDI
TTLVGKSSRVNKEFGIPDVEVCAPAKPGVTESIDRGEPKAGTCGEVANMAYPQESLVNAVSSANVQK
NISMVLQGHPVGEMGISRGVERHLETNMCEIFVDKDDIDVALDFYILALTPLRVGLDLPNARVEIDGKE
SRKLMAVRDGDAGLQRHPKVSESVGISQGAGSKAKLPDDALIHDGVQHGDIRFTATDLPEGRV-
VTPVFNDVKAQLKIDKVILRSFHVVTLSFSNDKADEHLIKLRLNALKKSTRHYEMLLKIRETECAESPHAI
SVGKILKIDALLQTICKAHYTAIEPLLADDLTALGVHGEQGLLHVVPRLMILAVLHLWGLEANAALRVW
DLQRESLVTDQTMGL-VRDRDWSF-AISEVVGNTFFARTKDDDTAQRLHARNPV-VLL-
YGKFGMV-
ISPSGLRLDSTPPSAREHGNAEQLVLSTERLQDAMAYYILRVLLVFVDEVIVAYGSGAEPLKFLVAEQAV
TGRDNPGMVTLTRRTFPIDLQKDAVFVLVFEDSTMKNLSHRKMHMNLVLIKSKGWIVNVSTKILISDQ
AIASYAEDAGAHTQPSESALAVITSRVIAPIVLVVSQKPTAPSKTCFLEALSLYDDVTATVLNAPKKYYIF
EGDVISVLGGLDAAVLLGLQRPQPDRLRRAVVAAAIRLAIAIR------------------------------
------------EFRVRAHKTSTKRDRPYKAHPKFLHKAELEKKLVAAGVDTLA
>GCA_004145405
AVIKPVTMNAYAPVLMIAQSQVATVSGAGSPSLRAKTVYAKSGLQRKAETKWVAKDFYY-
GVVKMATLVQLVSISVDKRAVCTVAQLVEQEVVVHGMVALEAVKKKIALEAIDGMTAEDPAKVVDY-
TRIAIRKWRDADDRADLLPVLLGLTLKIKFDPHQPLVHDLPIPVIFEVV-
GVMAQKGTIEVDECGISTAVSDVDPIAYVSPIDLAAVSGSAMDYIDQESAVAAAKQSIDL--
LALESTVLVVGIKPLIIPV-FRERADE-MSFANRSRSLGTIVDLAVSFPEQSRAR--SAFVAAL-
MDASQAAALAEKRGTAEQIRIVKRMQRSAGLADDPELVMLEDAAEGNSNWAGQIYDDPISGSSESD
SAGNIVYAANKRQNFARSITYLRQVEISDQADAPLMRGVKHNAKSRASRKLDDAIATLNFPNVPEGIA
IRAEARKPEQTLLHDAAEGEGILMLIVRWNVTWKTAIIIYLLELAGDTVGLIELGGIAAVGRPDGLLNRH

HKLNGSLIEQKRVIDKLTLRLRAALKQKLGAEQVIILVVRVQGDLVAQAEVQMG--
DVGAVVAIQGETIFVRRHRQTVIGDQEATV-VLRGVAHGPADAQALLRIRVDHTLVKLSVLMIPQ-
PVLVESVTIREGVGRALQIIQLDVYLLRDMGTGEETLAKSRSRELIRELLQIDPITGVRLAYGGLRERESKA
LSIKEIDTLF--
LAILRGVTEPRMQTLPAGVCRNFVRAGPRAAVGMLFLILDDAGKIYPIDGDLRENTKSIKSLIGLVDAAK
PKALSAVYMTERLKIRYADVQVLTSDADLYAYAEARRELLRMVPVYITNQAKRRQLVLLALSLREGHRR
VRMAIDVMIAAAIIESHADNGAVHHAEPRILLDDAEVVGSEMMISRQAALDLKQVIKMDVIVLFPEFQ
EKMVVSLRFFCTDVAIFVIALICTVGSMLSREPSHAQEGGAGFQKRIRAHSVLFALAASFDSANLRVE-
V-
SLFLRLGLAPPRFGLFYTTRPKRKQMPEEPDKDKVDGFDLVHAVKEDGRDNHKLEMPEAMALAVEAD
ASINVSGGVLAMDGEMAAMEKVDSKRKMALAVTKWTIASLDLLDILISGAAPIRALK-
IEDAEVTAALAKKELWFLYLPKANNAELETDKIDGAGSSTVDAAKDRQQGILLLNPVLVVIQDSNTASL
ISNDGLDEWGTMAYKKHYAFAARMRLHMRTIDGVEVGVVMQPNKQKAEKIKTVKLEGDTRFRQLA
TEGVKEQMP-LHALNPDFAV-LAVLVTPAVGRVAADEDVLGARVPCPASL-
RELQKSQKGDALPISAIRLLGMRGARAMVDEDIMVAGKVAASLLVIRAVQTILEIVLLNDSEATLPWRL
SVAERIEADTARAAGYAARPFNSKPSLCNVQAHARVADPIQEIAAGYVAEDKRFVFPVEDLPIQLILAS
PHSRVGGAKRAGLGHLHEMAKELLGKADLMVQAASDTNTEGLLKYMTTRQPTDLLHPDCLDTVIDL
RRMSVV---VQLNTV-
HVVVKGVGDVGDYEIMQAAAPSGLVQIERIDDRTGRERTRIHMYGAMEELWLLSVRYADWVVDCDI
GAIVLHVYAQVINRAFSLDRLQIAGWPPEASSWWNTWQLWLNKDGFFGYEAGFRAQAAVLASALA
DYQMKDVEGVGRYPSRQELRVGYRIRDQVAYARLDPRTDILLQSQTRLSVIRHALDVVAILIVREVVIE
FTLVVAEEDVAMGHVECVLADAFDFTAKNASIGPVGLKTPAPVLGDGTVLNDAVLGKIDLANTVADF
RYRMIDDGGRALGVEWLTLRNILFERIWLPPAQAALKNLQPICFFLGHPELNLALLEALWDGISKARM
AALMATALIEQILDRVAAFADADATTHQSNKAKHFKYYGEEVDPVYLAYI----
ALVATQVTVSDRLLKDRYVLNLKVFRGLTALWWLGAQLAQKGPAATIIALDSIAGLGALVLFIILL-
RDAVLLETLYFRYVY--MKGYDAAKDVADAEPEHLLNLAAVSRDVEWIEIVPAFFYYFTTAG-
FEPFNTAVAREFFGLFRERLRIVAVLAVPPERVEELVEFVALAGTLETSAEAQPGVIACGLCEPNAASVK
EADQDWNLHRSMGITLAFYDARESVKGWFDKGLHKPVAETGLSEAGALTTPFHAEVTAKQCVPALH
RTLDLGEIEASLIEVDSAVVVADEEQTLADKPTTRDNSVGAVGGVVLAGTMNMGSNLDAVERGGNIII
ELEEEQYSAVNGKKDGMKLTDKVLMVLSAAYFAVLARASSANALDRGVLDLLIDVPDDADNRDQGR
EL---
PDQYSFISMMIEASLEIMETAEVLDDYHVLEKGSRPPEFRVILIKGLHVRLLAGIATIIVVGSAGRAEVTAY
ISG-AVPITLNRVTFLPAFAASSCHVIDIFSTMLRPPERSAIKMLILETVAEL-
LEVTRLENDKLERMEILLPGLDMAQLLGLGAAASDFPQIVTIGAAIVAILSVAAEPVVLRDMLLSLAEIA
VEAMALALALLLMLTPDQGLTQKEAPREAQMKLLIQGGVVLQLRAADRMVLAATADKVRLDTDDG
TTSAFASLNLATVSDGFYVLSSAVLQLSSDLMMIVAVARVLADKEHKNALVVRHPEQVKTQTTYAEG
SPGMVLQTLTGAVPPLAIKAEEVELLWTLVAALWAVKAHLANATRPRHERVLILILALTLITVLLQSEGG
GIGGG--GPAAKALAAFSILTIKAKADDEVRKVTRRVLDLNSRLGRPEESVNPEFRGAELEQSK-------
------------------------------------EVTRIRFIDL-
VTKRSEIILAARAETDGIIAREMKGDLAALHPEAAALGKIAALQHFIAVQDGHISNYVTAVINSVLGMLL
LVLFINETRIAKVVWPTRREVTMVFIMAALAAFFIVIALLVSIVFMLAGISWQQMTTIVLGEGVMAVRQ
TSTFPKDS-
IGILQNTFADLICAGRVAKADIEKSLHIVVAGRVSRGTWYVIYLYEISQAQSEAGIMQRAFRQKTLSQSL
IDGRMEVKIKVVTSDRYAQTYEIKGGQHRKGTRFNRVKVGHNIPVDVLPRMKMAFAPPNLVLVKTFG

AVVQKRFASALTHMRHGAVQIGPMEVSSPYIQAKKARHAPGDAVVDLKPDNIVRSVAVVPELIWAR
ENEDNGAVHDKYVKPTHKLQAKRRTAVDKAQYDHRNQVALSFIGLAVKVLPAKAIVHATSGQLMPT
TSVAVTEGMKGIAATIRLDPMEKALIAYIKRMRPQLNLQGKTDKKIVKCLNNGLDDVEYGKLGRRKIKP
ITIKSVYDVEPERATELVKLLPYKDDVEGRKSQFAEK--
ALFEPLKAFMMLFGFIARSVYALNLRVIRRTILLVKCKIVVAAATVLSAIKIVGLSVRLRHKSTNVADTKH
HIKTITARANLTLAGAGKIKLNLSEGKVGKKGGRIKGYMPYNNYNIFDGLASGRRAAVKEVAKLKKVDK
EIKVDGVRQKQEGGRTPKAQLSNALANTRVGVDIVGESIQEQSNPRANAEMGQIFAKIDARVFSHVG
ALVGHVLVAVLAVVSIWGIGRAHNLLALRLRINAGIEMHSAA-VDEAVACA-
FAAPAGDASLFIDASEAYVGAMLITTQATDTCRLRRRSLSELKKTKKDAYMSLTCFAGKGDKVKIVEAA
QTQAYEIVATRTAERHGIKAKGTFGKERAMMQILPTKPVKVWVPVFVGEDELLAPIRQVAETVGDGV
TVVKLQNDIKITPFEKAVASIAVTGKSTSVIYTVLEKVTQLSKDIIYTQRAKAHYGRGRLRSHVVTHTKSN
RHLSMIKRTVVADPRMDAERLTDGEVSVVTGATMAFVMSEQDAV--
GDVDMHFHEEPRREAALGIAGPETFLATEHPRTLAVLFLRAYLIHIVEIETVKGFSGAATIYDNLRAMQN
KLDRKLSADIAKTRMVLVDNVEDMGVTDVAPKREAAGDPILSAFWINGVTANADIILLVANLEVPTVP
ENIQCAIRLFYQRVTLQAKERGGDVSVAVVPASKQIEAAEGTVIYSIKGKTPGTGQMKLTVEGISRSIVK
ALAVQLFQVDLFLEDTWLGTLLRAAIDVVFVDAGETKEAVDHVVEHDTEEERIPQKDIVSRKIRIYTPAA
PSAVVIWDPAIDREDKADAQGTILKIYGPNLQDINCFTDIGAEPKIESLKAWDEKEKYVVHRDDIPEDID
TLVGKSSRVNKEFAIPDVAVCAPSKPGVTESIDRGEPKAGTCGEVANMAYPQESLVNNVSSANVQKN
ISMVLAGNPVGEMGISREIERHLETNMCQIFVDKADVDVALDFYILSLTPLRVGLDKPNARVQIDGKES
RKLMAVLDGEADLQRHPKVSQAAGTGT-AGVKARLPDDTLEVDGVVHGDLSFTATDLPEGRV-
VTPVFNAVKAQLKIDKVILKAFHVVTLSFSNGKGEDHLIKLRLNALKKNTRHYEMLLKIRETECAESPHAI
SVGKVLKIDSLLQTICVAHYKAIEPLLADDLTALGVMGQQGLLHVVPRLMILAVMHLWGLEAQLELPH
WRLQARA-VTDQTMGLSVRERHFSY-CISEVVGNTFFARTKEDDTEQRLHARNPV-VLL-
AGKFGMV-
ISPSGLRLASTPATSLGRGNADDLILSTERLQDAMGYYVLRILLVFVDEVILAYGSGERPLKFLVGDQAV
TARQNPGMVTLTRRTFPLELQRHAVFVMVFEDCTMKNLSHKKMHMNLVLIKSKGWIVNVSTKILISD
QAIASYAEDAGAHTQPSESALAVITSRVMAPIVLVVSQKPTAPSKSLFLEALSLYDEVTATVVNAPKKY
YIFEGDVVSVLEPCDAAVLLGMQRPQPDRMRRAVVADAIRLAIALRASGIVTRLGEGLINFARAVSFA
APCYRLLRL-CGAALA-PALAFRVRNHSVSTKRSKPYKGHTKFLHKADLNKSLIAAGVDTIA
>GCA_004145845
AVIKPVTQKEYAPVLMIRQQQVATVSGAGSPSLRAKTVYRKSGLQRKTSTKWVASNFYY-
GVLKMGKIVQLVSISVDNRAVCTVAQLVEQEVVVHGMVALEAVKKKIALEAIDGMTSEDPAKVIQYA
QELELKRFRDAEDRADLLPVLLGLTLKIKFDPHQPVVHDLPIPVIYEVV-
GVMAQRGTIEVDDCAISTAISDVDPIAYVSPIDIAAVAGEKMAYIDQEAAVAAAKAEIDLEELALESKVL
VVGI-PLLIPV-FAADGDT-MSFANRSRTLETIVDLAVAYPEITRARIA-DFVADM-
MDRAKASALAEKRGTAEEIRIVKRMQRSSGLADNPDLVILDDAAEGNAQWAGQIYDDPISGSSESDS
AGNIVHAANKRQNFARSITYLRKVEISDQAAAPLMRGVKHNSKSRAARKVDDAIAGLNFPNVPEGIAI
RAEAGKPADTLLHDATEGEEILLLVVRWHVTWKTAIAVFLIGTPGNVVGIMTMSGIGAVGKPDGLLNR
HHKLNGSLIEQKRIMDKMTLRLRAALKQKLGAENVIILVVKVAGDLVADAKVQM---
AVGAVVAIQGETIFVRRHKQTVITDQEATVEILRGVAEGPADAQALLRIRVEHTLVKLSILLIPEVPVLVE
SVTIREGVGKALQIIQLDVYLLRDMGTGQETLAKSCSRELIRDLLMVDPITGVRLAFGKMKERLTREIKM
KDLETLL--
LAIADGVTEPRMANLPDGVFRDFVEAGPRAAVGTLFLLLDEAGKVYTIDGDLRELPKGIKSLIGLVDASK
PKILSAQYATARLKIRYADVSVLSSEANLYAYKEERRELLRMVPVYIVLAAKRRQLIIFALSLKGGHRRAR

AALDILIAAALLESQASEGDVNHADPAVLLDDAEIVGSTIMISRQSALELKSILKTDVIVVFPEFQEKMVV
SLKFFCTEVAIFVIALICTVGQMFSREPNAAQEGGAGFQKRIRGHSVLFALAVTFDRRNLRTA-
APGLFLGMGLAGPRYGIFYTNRPKKKQMPSEPDKDRVDGFELVHAIKADTTD-HKLELPEAMALAVE-
DAPINLSAGVLAMNGGMTAVERVDSKRKMALAAEKWSISKLSILDLSLSGAWPTRALD-
IEQADLTAALAAKEIWFLFLPGANNAELEEEKLDGAGASTVEIAADRQSEILLVVPVVVVIQDSNAASLI
ANGGVEEWGTMAYKKHYAFAARMRLHMRTIDAIELGVDLEPNKQKAEKIKTVKLEGDTRFRQLAVEG
IKEQMP-LHALNPDFAVLVAVLVTPAVGLVAAEADVLGARVPCDFSL-
RELQKSQKGDALPISAIRLLGMRAARAMIDEDIMVAGKVAA-
LLKIGSLETIVKLVLLNDSEATIPWRLSVAERLEADTKRSGGYAARPFKSKPSLCNVQAHANVADPIETIA
AGYVAVEERFVYPVEGIAIQLILASPHSRVGSAKRAGEGHLHEMAKELKAKAVIMVRSEPDTNTEGLLN
YITTQQPTDLLFEDCLDIVIDMRRMSVV---
IQLNTI-
HVVVPGARDVADFEIMAAAAPSGLVQIERVDDRTGRERTKMWMYGAMEDLWLMSVTWADWVPD
CAIGAIVLHVYAQVINRAFSLKRLQLEGWPKEADRWWNTWQLWLNKDGFFGYDAAFRA-
LQVLKAELYDYEMNDINAVGRYQSRQQLRVGYRIRQQVAYERLDPRTDILLQSQTRLSVIRHALKMV
AILIVRDIVVEFTLVVAQEDVAMGHVECVLAESFDFTQKNAGIGPVGLRTPDDILGDGTVFQEAVLGKI
DLANTVADFRYRFIVDGGRALGVKWIDL-
NMLYTRIWLPPAAAALKNLTPIKFFLGHPELELLLSDSMFDGISKARADRLMAGVLVEQILDRVAAFAE
PEATTHQSKKKRHFKYYGEEVDPVHLAYILEVFALVATQVTVSDRLLKDRYVLNLKV-
RSFTSLLWLGLQLGDKGPAATVITVDTIAGLGALVLFIILL-RDTVLVETLFYRYVY--
MKGYEAAKDAADALPEHFLPLGAASRDVEWIEIVPAFFYYFKTAT-
FEPFNTAVAKEFFGLFEERLRVVEVLAVPPDRVEALLAFVALAGTLATSAEALPGVIATGFCEPNAAAVL
EADQDWNLHRTMGITLAFFKAQDSVKGWFDKGLHKTVAAVSLKEAGALSTPFLAEVEAKVCCPAVH
RVIDLGEIEAALIEIDSAVVVADEEQTLADRPTTRDKSVGAVGGVVLAGTMNMGAALDQVERGGNIII
ELEEENYHAVNGKKADGKLTDKILMVLSAAYFPVLRRTTSANALDRGVFGLLIEAPDDADNLDHGREL
TIPAPTIH-ETTPLYAESEIERIAWIAPMHMLGTLPAVL-
FEPDQYGFISMMIEASLDITETAEVLDDYHVLERGSRPPDFRVVLIKGLHIRLLAGIGTIIVVGSAGRSEVT
AYING-AVPISLKKQTFLPAFAPSSCHIVDIFSTMLRPPERSTVMMLILETVAEL-
LEVTRIENDKLERLQVLLPGLDLAELLGLVAAAEDFAAIVTIEAAITAILSVAAEAVVLRELLLSLAEIAVTA
LALALALLLMLTPDQGLTQKEVARETQMKELIQGGVVLQVHAGDRMVLASTADKVRLDTDEGTTSA
FASLNLATVSDGFYVLSSAVLQLSSDLMMIVSVARVLADKETKNALVVRHPEQVKTQATYAEGSPGM
VLQTLTQAVPPLAIKAEDVELLWTLVAALWRVKAHIASAARPHTERVLILILALSLIIVLIQSEGGGMGG-
-
VSSAAKILAGFSILTIKAKVGDEVRKVTRRVMDLNGRLGRPEEAVNPEFRGAELEQSKYEAENVEVDC
METVRRFVDIPFLVSIRTQHNESEDRGARLVLDVTRVRFIDL-
VTKRSEIILAARVETDGIIAREMAKDLQALHPDAAALGKVAALQHFVSLSDAHVSNYVTADIHSVLGM
LLLVLFIQQTRIAKVVWPTRREVTMVFIMAVLASFFLVIGL--
QIVFMLSGISYQQLTTLVLGEGVGLVRQTSTFPADS-
IGILQNTYADLVCAGRVTKADIENSLHIVVGARVARGTWYVIYLYEITQAQGQSGIMQRAFRQATLSQ
SLIDGRMEVKIKVVTSDRYA-
TYEIKGGQHRKGTRYNRVKVGHNIPVQVLPRLKMAFGPANLILVKTFGAVVQKRFAQPLTHIRHGAV
TIGPMEVASPYIQNKKARHAPGDSVVDLKPDNIVRSVAVVPELIWARENEDNGAVHDKYVKTTHKLA
AKRKRAVDKAQYDKRNQVALSFIGLAVKVLPEKAIVQATAGTLLPTESVAVTEGLKGIAATIRLDPMEK
ALIAYIKRMRPQLNLGGKADKKIVKCLNNNLDDVEYGKLGRRKIKPLTIKSVYDVEHREATELVKLMPY

KDDVEGRKSQFAEK--
ALFEPLKAFMMLFGFIARAIYALSLRVVRRTILLVKTKVVVNASTVLSAIKIIGVSIRLRHKSTNVADTNHH
IKSIEARARMTLAGAGKVKLHLREGRVGKKGGRIKGYMPYKNFNIFDKLESGRRAKVKELAKLKKVDKEI
AVEGIRVKQQGGRTPKADLSNAIANTRVGVDIVGENEQEESNPRANAEMGQIFAAIDARVYSLVGAL
VGHALEAILAICAILGIGRAHALLAVKLKVNAGIEMHSAA-VETAVACA-FAQPAGEA-
TIADVSESYLGAISITDTSLDVCRRPRRSLSELKKTKKDAYVSLTCFSGKGERIKVIEASQTQAYEIVATRT
AERHGIKAKGTYGKERALMQITPTKPVKVWVPVFVNEEELLAPVRAFAETNGDGVTVVKLTKDIKVTM
FEKAVASLAVSGKATSVVYTVLEKVTELAKDIIYEQRAKAHYGRGRLRSHVVTTTKQKRHLSMIKRTVV
ASPHVEASRLHDGEVSVITGATMAFVMSEQDAV--
GDVDLHYHEEPRREAALGIAGPETFLVTEQPRLLLFLTLRAYLIHVIDIGTVGGIADAATIYDILRAMQNK
LDRKLSADIAKVRMCLHDDLQGMGVTEAAARREEAGDTILSAYWINGVPANVDIVLLVANLEVPCVP
ENIDCAIRLFYQRITLQAKERGGDVSVAVVKAQNQIEAAEGTVIYSIKGNKPGTVQMKLNVEAISRSVV
KSLAVQLFQVTLFLDDTWIATLLRAALKVVFVQAEDGKEAVDHVVERDTDLEFAPQKDILPRKIRIYTP
AAPSAVVIWDPAIEREDKADAQGTILKIYGPNLQDISCFTDIGAEPKIESLKSWDEKEKYVVHRDDIPEDI
-
TLVGKSSKVNKEYAIPDVAVVAPAKPGVTESIDRGEPKAGSCAEVANMAYPQESLVNAVSSANVQKN
ISMVLAGHPVGEMGISRGVERHLETNMCEIFVDKADVDVALDFYKLSLTPLRVGLDRPNAKVQIDGKE
SRKLMAVLDGDADLQRHPKTSAATGLTQGAGVKAKLPDDALIVDGVQHGDIKFTATDLPDGRV-
VTPVYNAVKAQLDIDKVILKTFHVVTLSFSNDKGEEHLIKLRLNALKKSTRHYEMLLKIRETECAESPHAI
SVGKILKIDALLQTICTAHYTAIEPLLADDLTALGVMGRQGLLHVVPRLMILAVLHVVDLDARAYINRW
WVMARAIVTDITMGLDQRDRNWTF-AISEVVGNTFFARTPDDDTEQRLHARNPI-VLL-
YGKFDMV-
ISPSGLRLESTADTDQEHGNADRLILSAERLQDAMGYFVLQVLLVFVDEVLLAYGSGDEPIKFLVAEQA
VTGRQNPQMVTLTR-
TFPLDLQRDATFVLVNEDSTMKNLSHRKMHMNLVLIKSKGWIVNVSTKILISDQAIASYAEDAGAHT
QPSESALAVITSRVIAPIVMVISQKPTAPSKSLFLEALALYDAVTATVLNKPKRYYIFEGDVVSVLDTVDA
AVLKGMQRPQPDRMRRAVIAAAIRLAIAG-ASRMSTRLEAGIVNFSRAISFVG-RYTAVAL---------
--EFRVRNHSVSTKRSKPYKAHTKFLHKADVEKGLVAAGVDTLA
>GCA_006152115
AVIKPVIMAVYAPVLMIRQSQVATVSGAGSPSLRSKTVYAKSGLQRKSVTKRVRAPLYY-
GVIKSGKLVQLVSISVDHRAVCTVAKVVEQEVVVHGMVALDAVKKKIALEAMSGMTSEDPAKVVDY
ALELDLKRVRETEDRADLLPILLGLTVSIRFDPHQPLVYDLPVPVALEVL-
GIMAQKGTMEVDDCALSTAVSDVDPIAYVSPIDLARVSGARMEFIIQDAAIDAAIDEIALDELALESSVL
IVGISPLLLPV-FGEAGGTAMSFRNRSRDLETIVDLAVAALAATRARIALAIASAM-
FTRTQAAALAEKRSTAEEIRIVKRMARALGLADNPELLLLEDAAEGNSAWAGQIYDDDISGSSESDSA
GNIVFAANERQSFKRSITFLRKVEISDNAEAPLMRGVKNNAKSRSARKLNDKIAELNFPNVPGGIAIRA
EAGKSEDALLHDADEGEEILLLVVRWKVTWKTALVLLLVVRAGDVVGVVTIGGIAAVGRPDGLLNRH
HKLNGSLIEQKRVIDKLTLRLRAALKQKLGQEQVIILVVRVQGDLVAEATIQIG--
DIGAVVAIQGETIFVRRHKQTLVTDEDSTVEVLRGVAQGPADAAALLRIRVSHTLVEITVLLIPE-
PVLVEAVSIREGVGKALQIVQLDVYLLRDMGTGEPTLAMSKSRELIREILALDPITGVRLAYGNLKERQG
REMTLKDLE-----LALAA-
VTEPRMEDLPDGVRRTLIEAGPRAAVGMLFILLDPAGRVYQIDGELREGPKTVKALLGLLDTARPRVLS
AGYGTERLRIRFADAMVLISEANLYAYKEERKELLRMVPSYIVLAAERRQLVILALSLKGGHRRAIAAIGV
MIAAAIMESQAVEGAVHHAEPRVVLDDAEIVGSEMMISRQAALDLKQILKMDVIVVFPDFQEKMVVS

LRFFCTEVAIFVIALITTVGQMLSRSPDHAQEEGAGYTRRIRAHALLFALSAVLERRNAKLDATPTLFLEL
GLAPLRYGVLYTQQPKRKQMPSEPDSDKVDGFELVHAVKADTRDNHRLELREAMALAVE-
DSPINLSAGVLALSGTMAALVRVDSKRKMAVAVARWTISPVPLLDLALAGAATVRCLG-
VEQADVSAALPAKALWFLFLPGASNKELEEQRLDGAGASGIDAAEDKQNAILIVRPVVVVIQDSNTA
ALIANGGVGEWGTMSYKKHYAFAARMRLHMRTIDGVELGTSLEPTKQKAEKIKTVKLEGDTRFRQLA
VAGIREQMP-LHALNPDFAV-LAVLVTPAVGRVAADEDLLGGRVPCSASL-
RELQKSQTGDALPISAVRVTGLRAKRAFIDEDVMVAGKIAA-
LLVILATETVAALVLMNDSEATLPWRLSVAERIEADTKRAGGYAARPFRSKPSLCNTQAHAKASEPIKEI
AAGYVAKPDRFVFPVEGIPIQLILSHPHSRVGSAKRSGEGHLHEMAKELRARAVLLCRGSPGTSTEGM
ARIVTTGQPADLLQEDCLDVVIDLRRMSIV---
IQLNSV--
VLVPSVREVGKYKIMAAASPSGLIDVERVDDRTGAERTRMWMYGAMEDLWLLSIRWADWIRRAPIG
AIVLHVYAQQINRAFSLRRLQLLGWPQEAARWWNTWQLWLNKDGFFGYDAAFRA-
RQVLKAELYDYEMSDIEGVGRYQSRQELRVGYRIRQEVEFVRLDPRTDILLQSKTRLSVIRHALTIVAILI
VQQVVSEFS-
VVAMEDVAVGHVECVLADRFDFTAKNADIGPPGLRDPAPVLGDGTIMQAALLGKVDWETTAADFR
YLLLTDGGRRLGVQWVA--NMLYARIWVPPAAAALKDLLPICFFLGHPDLDLEAWEHLFE-
ISKARASRLMAGALIEDILEKIAAFADPEATTHQSKKAKHFKTYGEEVDPVHLAYILELFALVATEVTVSD
RLLKDRTVLQMRIFRGFTSLWWISLQLGNKGPAATVITVGSIAGLGALVLFIILL-
RASPLVETLFWRYVY--MRGYQAAKDVADADPEHFLVLASVTRDVEWVEVIPAFFYYFKTAK-
FEPFATALAREFFALFQERL---
DVLAVPPERVERLREFVALAGTLPTSADALPGVIACALCEPNAAQVLEQAQDWNLHRAMGISLAFFD
ARESVKGWFDKGLHKPVAATGLSEAGALTTPFRAAVDARGCAPALHRVLDLGELEQALLELASAVLV
ADEEQTLADKPTTRDRAVKAVGGVILAGTMNMGASLEQLERGSNIIIELEEENYHAVNGKKGNGKLT
DKVLMVLSAAYLPVVARFSSANALDRGAFGLLIDAPDDADNLDHGGEM---------------------
----
PDLYSFISMIIEMALAIMRTAEALADYHVLEKGGRPSDFKIILIRGLHVRTLAGIGTIIVVGSAGRDEVTAYI
RG-AVPIGLRRKTFLPAFAPSSCFVIDIFSTMLRPPERSAVQMLILETVAEL-
LDVTRPENDKLEKLLLLLPGLDIARLMVMVAAAEDFPGIVTISHAITAILSVAAEAVVLRELLLTLAEIAITA
LALALALLLMLTPDAGLVQREVGREAQLKELIQGGSVLQVSAADAMVLAATADKVRLDTDDGTTSAF
ASLNLATVSDGFYVLSSAVLQLSSDVMMIVNVAKVLADKEFKNALVVRHPEQVKTQPTYAEGSPGM
VLQTLTQSVPPLAVKPETVELLWTLVAALWRVKSHLAEAARPHGERALILILAVALIVVLLQSEGGG--
MG-
MGAQARLIGAFSVLTIKAKAGEEVRKVARRVLDLTSRLGKPEEAVNPEFRGADLEQTKFEAANVEVDC
LETIRRFVDIPFLVSIRTQHNESEDRGARMVLDVTRTRFIDL-
VTKKSEIILAARVETDGVIAREMQKDLQALHTEATALGKVAMLQHYISLNDAHISNYVTAQIHSVLGM
LLLVLFINQTRVGKVVWPTRREVTMVLVLAALAVFFLVIGL--
QIVFMLSGISYNQLTTLVLGEGIMAVRQSSTFPAES-
IGILQNTFADLIAAGRVAKADIEASLHIVVGARVARGTWYVIYLYEISQAQGPSGILQRAFRQATLSQSL
IDGRMEVKIKVVTSDRYA-
TYEVKGGQHRKGTKYNRVKIAHNIPVAIQTRLKLAFGDPNLVLVKTFGAVVQKRYAQPLTHIRHGAVT
IGPMEVSSPYITSKKARHAPGDSIVEIRPDNIVRSVAVVPELIWARENEDNNAVHDKYTKPTHKLAAKR
RKAVDKAQYDRRDQVALSFIGLAVKVLPEKAIV-
ATAGTLLPTESVAVTEGIKGIAATIRIDPMEKSIIAYIKRMRPQLNLAGKADKRIVKCLNNNLDDVEWGK

LGRRRIKPLTIKSVYDVDPREATELVKLMPYKDDVEGRKSQFAEK--
AIFEPLKAFMMVFGFIARSIYALSLRVVRRTILLAKCKIVVAADTVLSAIKIIGVTIRLRHKTTNIADTDHHIK
TIEKRARLSLAGVGQVKLNLRPGKVGVKAGRIKGYMPYKNYNIFDGLSSGRRSSVAALAKLKRVDKEIE
VNGIRVKQQGGRQPKADLSNALANTRVEVDIVGETIQEESNPRANAEMGQVFAAIDARVYHLVGALI
GHALAAVLAVVTIWGIGKTYSLLAVAFRINAGIEMHSAA-VERAVAAA-----------------------
----
LKKTKKDAYMRLTCFSGKGDRIRIIEASQTQAYEIVATRTAERHGIHAKGTYGKERALMQIVPTKPVKV
WVPIFVPEDELLAPVRAFAETNGDGVTVVKLTTDIKITPFEKAVASLAVTGKSTSVLYTVLEKVTELMKDI
IYEQRAKAHYGRGRLRSHVVTHTKTKRHLSMMRRTVVADPRMDAARLTDGEVSVITGATMAFVMSE
QDAV--
GEVDFHFHKEPQNEAALGIGGPQTFLATEQPRVLALLFLREYLLHIVEIDSVGNVADAATIYDILRAMQ
AKLDRKLSADIAKVRMCLHDNVPQMGVTEVAPRRETAGDEILSAYWINGVPANADIILLVANLEIATV
PENIDCAIRLFYQRITLQAKERGGDTSVAVVKAQNQIAEAEGVVIYSLKATRPGTLQMKLIVEAISKSIVK
SLAVQLFQVTLFINDTWIGTLLRAAADVVYLSAEEGREAVDHVVERDSAVEVAPQKDIVARKIRIFTPA
APSAVVIWDPAIEREDKAEAQGTILKIYGPNLQDISCFTDIGAEPKIESLKSWDEKEKYVVHRDDIPPDID
VLVGKSSRVNKEFGIPDVAVCAPSKPGVTESIDRGDPKAGSCGEVANLPYPQEALLNAVSSANVQKNI
SMVLGGHPVGEMGISRDVERHLETNMCEIFVDKPDVTVALDFYVIQLT-
LRVGLDPPNTRVEIDGKEGRKLMAVLDGEAELQRHPKVSVAVGIM-
TAGSKAKLPPDALIQDGVLHGDVGFTVLDIPQGRV-
VTSVTLKVKAQLKIDKVLVKTFHVVTLSFSNNKGEEHLIKLRLQALKKSTRHYEMLLKIRETECAESPHAI
DVGKILKIDALLQTICVAHYTEIEPLLADGLAALGVLGEQGLLHVVPRLMILAVLHLWHLEADLSIVRW
WMQQRAIVTDQTLGLDSRDRDWSF-AVSEVVGNTFFAKTAEDDTVQRLRARNPI-ILL-
CGKAEMV-
ISPSGLRLSSTPPTPMEEGNTSRVILSQERLQDAMEYYVLKVLLVFVDQIILAFGSGDEPLRFLVADQAV
TGRENPGMVTLTRRTFPLDVQRDATFVLVFADSTMKNLSHRKMHMNLVLVKTKGWVVNVSTKILIS
DQAIASYAEDAGAHTQPSESALAVITSRVIAPIVLVISQKPTAPSKSLFLDALALYDAITTDVQEGPKRYF
IFEGDLVSVLGGIDAAVLRALQRPQPERMRRAVIAGAIRLAIAIRASGVLVQLEAGVLNFARAVTFAA-
AHHATEALLFAAID-PALAFRVRAHSVSTKRDKPYKAHNKFLHKAEVDKRLVAEGVDTLA
>GCA_006363825
AVIKPFLYTKYAPVLMIRQQQVATVSGAGSPSLRAKTVYAKSGLQRKTLTKWVAKPFYY-
GVLKMANVVQLVSISVDNRTVCTVAQLVEQEVVVHGLVALDAVKKKIALEEIDGMTSEDPAKVVVYE
DELELKRFRDADDRADMLPVLLGLTLKIKFDPHQPVVHDLPIPTVYEVV-
GVMAQKGTIEVYDCAISTAISDVDPIAYVSPIDLDAVAGEKMAYIDQENAVSTVKQEIALEELALESGVL
VVGI-PLLIPV-FADEG-T-MSFANRSRSLATIVNLAVSHPQLTRA-IEQ-MIADM-
MREAQAAALADRRGTAEEIRIVKRMQRSAGLADDPDLVMLEDAAEGNAQWAGQIYDDPISGSSES
DSAGNIVHAANKRQNNARSITQLRKVEISDKASAPLMRGVKHNSKSRSARKIDDAIAGLNFANVPEGI
AIQADAEKPQDLLLHDADEGEEILLIIVRWHVTWKTAVFIALALRAGDLVGLISLGGIAAVGRPDGILNR
HHKLNGSLIEQKRVMDKLTLRLRASLKQKLGAENVIILVVKVAGDIVADATVQM---
SVGAVVAIQGETIFVRRHRQTLVTDSEATVEILRGVAEGPADAQALLRIRVEHTLVKMSVLLIPE-
PVLVESVTIREGVGKALQIIQLDVYLLRDMGTGEETLAKSKS---------
TDPITGVQLAYGNIKERLGKDTNLKDLETMLE-
LVITGDVTEPRMANYPMGVYRTLIEAGPRAAVGMLFLLLDEAGRLYSISGDLRELTKSIKSLIGIVDAAVP
KVLSAVYGTARLKIRYADVTVLTSDAELYAYAEERREILRMVPVYITTAAKRRQLIIFALSLKGGHRRARA
ALGVLLPAVIRESQTEQGEVNHAAPAILLDDAEVVGSEMMITRQSALDLKQVHKVDVIVVFPEFQEK

MVVSLKFFCTEVAVFVIALIVTVGEMLSREPNHAQEGGAGYQKRIRGHSILFALVCTFDRRNARCD-V-
GLFLGLALAPPRYGLLYTQQPKKKQMPSEEDDEKVDGFELIHVVKEDATN-
HELEMPEAMALAVEADTSINVSAGVLALDGGMAAVERIDSKRKMALAATKWTISTAALLDLRLSGAA
PMRALG-AEMADLTAALPKKDI--
LFLPRANNAELEEEKLDGAGASSADAAEDRQSAILLVYPVLVVIQDSNTAGLITNEGVEEWGTMAYKK
HYAFAARMRLHMRTID------QMEPNKQKSEKVKTVKLEGDTRFRQLAVEGVKEQMP-
LHALNPDFAVLVALLVSPAVGLVAANQDLAGAGVPCGASL-
RELQKSQKGDALPISAVRLLGMAAARAMIDEDIMVAGKIAA-
LFKIGALRTVIEIVLRNDSEATLPWRLSVAERLEADTRRGGGFAARPFKSKPSLCNVQAHANVADPIQEI
AAGYVATEEKFVFPVEGIPIQLILANTHSRVGEAKRAEEGHLHEMAKELIAKAVLMLKSEPDTNTEGML
DYITTGQPTDLLFKDCLDIIIDLRRMNII--
VHLNTI-
HVVVPGVRDVADFEIMAANAPSGLVQVERVDERTGRERTMMWMYGAMEELWLMSVTYADWVV
DVPIGAIVLHVYAQTLNRAYSLTRLQLAGWPKEAESWWNTWQLWLNKDGFFGYTAAFRAVKAILKA
DLYDYELRDTEGVGRMQSRQELRVGYRIRQEVSFARLSPRTDILLQSQTRLSMLRHALEVVAILIMRDI
VAEFT-
VVAEEDVAMGHVECVLADSFDFTAKNAHIGPVGLRTPENVLGDGTVFKEAVLGKVDFENTVADFRY
RVLTDGGRRLGVEW----NMLYARIWLPPAAAALKALQPISFFLGQPDLDLELW-
LLFAGISKARADKLMAGALVEEILSGVARFAAPEATTHQSHKTKHFKYYGEEVDPVYLAYILDLFALVAT
QVTVSDRLLKDRYVLNLRIFRGLTSLLLLAAQLGEKGPSATVITVDTIMGLGALVLFIILL-
RDLVLLETLYYRYVYV-MKGYEAAKDVADADPEHLLPLASVTRDVEWIEIVPAFFYYFKTAG-
FEPFNTAVAKEFFGLFEERLRIVDVLAVPPDRVEALVEFVALAGTLVTSADALEGVIACGFCEPNAAEVL
ETDQDWNLHRHMGITLAFYKAQESVKSWFDKGLHKDVAETGLREAGAFTQPFRAEVQARKCVPVA
HRVVDLGDIEAALIEIDSAVTVADEEQTLADQPTTREKAVGAIGGVVLAGTMNMGANLEQVERGNNI
IIELEEEDYRAVNGRKD-
QKMTDKVLMVLSAALFPVMARTVSANALDRGVFDLLIDVPDDADNLDHGRDLTIPAATVHLETTPLY
AESEIERIAWIAPMHLLGTLDAVLGFEPDQYSFMSMMIDASLEITETSEVLDEYHVLEKGSRPPEFRVILI
KGLHIRVLAGIATIIVVGSAGRAEVTAYIEG-
AVPITLNKHTYLPAFAPSSCVTIDIFSTLLRPPERSSVQMLILETVAEL-
LEVTRIENDKLERLDLLLPGLDIAALLTLEAAAKDFASIVTIAEAIVAILSVAAEAVVLREMLLTLAEIAVTAL
ALALALLLMLTPDRGLHQKEFAREAQMKELIQGGAVLQVRAAEDMVLAATADKVRLETDEGTTSAFA
SLNLATVSDGFYVLSSAVLQLSSDLMMVVGVARVLADKETKNALVVRHPEQVKTQPTYAEGSPGMV
LQTLTQSIPPLAVKPQEVELLWTLVAALWRVKAHIASAARPRIERILVLLLALTLIVVLMQSEGGGIGGG
-
MGSAAKGLAAFSILTIKASVGDEVRKVTRRVMDLNSRLGRPEEAVNPEFKGAELDQSKYEAANVEVDT
LETIRRFVDIPFLVSTRTQHNESEDRGARMVLDVTRVRFIDL-
VTKRSEIILAARAETDGIISREMQKDLQALHPEAAALGKIAMLQHFISLNDGHISNYVTADIHSVLGMLL
LVLFISEVRVGKVTWPTRREVTMVFLLATFAVFFLVIGL--
QLVFMLSGISYQQLTTLVLGEGVMAVRQTSTFPADS-
IGILQNTYADLICAGRVTKADIEKSLHIVVGARVARGTWYVIYHYEITQAQGEAGIMQRAFRQATLSQS
LIDGRMEVKIKVVTSDRYA-
TYEIKGGQHRKGTKFNRVKVGHNIPVQVLPRLKMAFGPPHLILVKTFGAVVQKRFAQPLTHMRHGAV
TIGPMEVASPYIVAKKARHAPGDSVVDLKPDNVVRSVAVVPELIWARENEDQGAVHDRYVKTTHKLS
AKRTKAVDKAQYDHKNQVHLSFIGLAVKVLPEKAIVHATSGTLLPTEAVAVTEGLKGVAAQIRLDPME

KALIAYIKRMRPQLNLGGKTDKRIVKCLNNNLDDVEYGKLGRRRIKPITIKSVYDVEHELATELVKLLPYK
DDVEGRLSQFALK--
AIFEPLRAFMMLFNYIARCIYALSLRVVRRTIVLVKTRIVVNADTVLSAIKIIGVSIRLRHKSTNVADTNHYI
KTIEDRARMTLAGAGKVKLNLRPGRVGKKGGRIKGYMPYKNYNIFDGLESGRRAKVKALAKIKKVDKE
VNVDGVRQKQQGGRTPKADLSNAV-
NTRVDVDIVGENEQDESNPRANAEMGQIFAAIDARVYHLVAALIGHALEAILAVAAIWGIGKAHALL
AVRLKINAGIEFHSAA-VDQAVAAA-
HAQPAGEASALADVSEYPGVALLVTDASLDVCRLRRRQLSELKKTKKEAYMKLTCFSGKGDRVKIVEA
SQTQAYEIVATRTAERHGIHAKGTYGKERAMMQILPTKPVKVWVPVFVNEAELLAPIRAFAETNGDG
VEVVKLQNDIKITMFEKAVASIAVAGKATSIVYTVLEKVTELMKDIIYEQRAKAHYGRGRLRSHVVTHTK
QKRHLSMMRRTVVADPRMDAARLVDGEVSVITGATMAFVMSEQDAV-
PADVDLHFHEEPAREADLGIAGPETFLATEQPKVLVLLFLRDYLIHIVEIDTVGGFSDAATIYDILRPLQN
KLDRKLSADIAKVRMVLHDDMADMGVTMEAPRREAAGDPILSAYWINGVPANSDIILLVANLEVATV
PENIDCAIRLFYQRVTLQAKERDGDVSVALVPAQNQIEAAEGTVIYTIKGKTPGTVQMKLNVESISRSIV
KSFAVQLFQVDTFLDDTWIATLLRVAGDVVFVDAREGKEAVDHVVERDTELELAPQKDIVARKIRIYTP
AAPSAVVIWDPAIEREDKADAQGTILKIYGPNLQDIACFTDIGAEPKIESLKAWDEKEKYVVHRDDIPED
I-
VLVGKSSKVNKEFGIPDVQVVAPSKPGVTESIDRGEPKAGTCGEVANMAYPQESIVNSVSSANVQKNI
SMVLGGHPVGEMGISREIERHLETNMCEIFVDKPDVDVALDFYRLSLTPLRVGLDPPNAKVEIDGKESR
KLMAVIDGDARLQRHPKVSGALGTG-GGGTKARLPDDTLIHDGVHHGDIKFTATDLPPGRV-
VTPVLNDVKQQLKIDKVILKTFHVVTLSFSNDKADEHLIKLRLNALKKNTRHYEMMLKIRETECAESPH
AIPVGKILKIDLLLQTICVAHYTAIEPLLADDLTALGVTGDQGILHVVPRLMIMAVMHLWGLEAAMELP
RW-LMARSLVTDLGMGLAIRERHFTY-TISEVVGNTFFAMTANDDTEERLHARNPI-VLL-
WGKFAMT-
ISPSGLKLASTPESSLQHGNENDLILSPERLTDEMAYYVLRILLVFVDEIILAYGSGSEPLRFLVAEQAVTG
RDNPGLVCLTRRTFPLDLQRDATFVLVFADSTMKNLSHKKMHMNLVIIKSKGWIVNVSTKILISDQAIA
SFAEDAGAHTQPSESALAAITSRVIAPIVLVVSQKPTAPSKTLFLEALNLYDDINATVMNPPKKYYIFEG
DVVSVLDTVDAAVLKGVQRPQPDRMRRAVIAAAIRLAIAGLASPFRVVLD-GGINFARAIDFAA--H-
---------------GFRVRNHKTSTKRDRPYKGHTKFLHKAELEKSLIAQGVDTIA
>GCA_008124775
AVIKPVTMDKYAPVLMIRQTEVATVSGAGSPSLRAKTVYAKSGLQRKTSTKWVAKPLYF-
GVLQMGKIVQLVSISVDNRSVCTVAQVVEQEVVVHGLVALDVVKRKIALEAIQGMTAEDPAKVMDY
PEEVELKKFRDADDRADMLPVMLGLTLSLKFDPHEPVVHDLAVPVIYEVLGNIMADKGGIEVDDLSIN
TALGDVDPIEYVSPIDIEAVAGDKVAYIDQEAAVAAAKEEIALEELALESSVLVVGI-PLLLPV-
FGEEGGT-MSFANRSRKLESIVDLAVAYADLPRSRIQ-DIAAEM-
MGRMRAAALAEKRGTAEEIRIVKRMQRAAGLADNPELVMKEDAAEGHSSWAGQIYDDPISGSSESD
SAGNIVFAANKRQNFARSITYLRKVEISDKAAAPLMRGVKHNAKSRSSRKLDDDIAALNFPNVPEGIAI
RAEAGKPVDTLLHDAAEGEEILMLIIRLKVTWKTAVAIMLTLVAGDVVGLIELGGIAAVGKPDGLLNRH
HKMNGSLIEQRPIIEKLTLRLRASLKQKLGAEKVIIFVVKVQGDVVAEAKIQM---
TLGTVVAIQGELIFVRRHRQTLITDSESTV-ILRGVAEGPADARALLRIRVSHTLVQLSVLLIPD-
PVLVESVTIREGVGKALQIIQLDVYLLRDMGTGEDTLAESQSRELIRDVLMIDPITGVRLAYGKIKERFGK
VTNLKDLETLF--
RFIITSVTEPRMDNLPDGVHRNLVEAGPRAIVGTLFLILDDAGKLYDIDGDMRELTKTIKATIGLVDAKA
PKVLSAAYLTKRLKIRFADVEVLVAEADLYAYKEERKELLRMVPVYVIVAAKKRQLVIFALSLRRGHRRA

RAALAVLIAAAILESQAERGAVNHADPAVLLDDAEIVKSAMMISRQASLDLKEIIKVDVIVVFPEFQEK
MVVSLRFFCTEVAIFVIALIVTVGQMLSREPESAQEDGAGYNKRIRAHSMLFALAVTFDRRNCRNA-
TPTLFLGLGLAPPRYGI-YVNQPKKKNMPTEPDDDRVDGFELVNAIKEDTTSNHELDFPEAMALAVE-
DAPINVTGGVLAMTGGMAAIERIESKRKLSLAAT--
--
LYLPGASNEELEESNLDGAGNSTADVAEDRQREILLIHPVLVVIQDSNAAGLVSNGGVDEWGTMAYK
KHYAFAARMRLHMRTIDAIEVGVELEPNKQKAEKVKTVKLEGDTRFRQLAVEGVKEQMP-
LHALNPDFAVLVPVLVTPAVGRVAADTDVLGASVPCPFSL-
NELQKSQKGAALPIAGVRLLDMRAARGMIEEDIMVAGKVAASLYKIGALDTILELVLRNDTEATRPWR
LSVAERLEADTARSGGYAARPFATKPSLCNVQAHATTAEPIQEIAVGYVAKEERFVYPVEGIAIQLILAG
PHSRVDLAKRAGEGHLHEMAKELFARAVLMVRNEPGTNTEGMLRYMTTNQPTDLLFEECLDVVIDL
RRMTVV--VQLNTV-
HVVVAGTSDVGDYSIMSANFPSGLVQVERVDDRTGRERTKMWMYGAMEELWLMSVRWADWVV
DCAIGAIILHVYAQTLNRAFSYERLQIDGWPKEASRWWNTWQLWLNTDGFFGYDGAFRAVAAILKQ
DLADYDLSDVEGVGRYQSKQELRVGYRIREEVGFSRLDPRTDILLQAKTRLSVIRHALDVVAILVLRDVV
GEFT-
VVAKEDVAMGHVECVLADRFSFTAKNAHIGPTGLRTPAPIFGDGTVFKEAVLGKIDLANTVADFRYLVI
VDGGRELGVEWVSMRNMLYPRIWLPPADAALKNLEPIKFFLGHPEVNL----SKYA-
ISKARADKLMAGALIEEILDNVARFAAPEATTHQSKKAKHFKYYGEEVDPVHLAYILNVFALVATQVVV
SDRLLKDRYVLNMKI-RGVTSLLLLSLNLSEKGPAATAITVGTVAGLGALVLFIILL-
RDSVLLETLYHRYVYV-MRGYTAAKDVADA-PEHFLSLAALSRDVEYIEIIPAFFYYFKTAG-
FEPFNTAVAKEFFGLFQERLKVIDVLVVPPDRVEALIVFVALAGTLATSAEAMVGIIACGLCEPNAAAVL
ETDQDFNLHRAMGITLAFYEARESVKGWFDKGLHKVVAAVSLKDAGAFSTPNLCEVTAKVCVPAIHR
VLDLGAIEAALIEIDSAVVVADEEQTLADRPTTRDKAVGCVGGVVLAGTMNMGGALEQVERSNNIIIE
LEEEQYSAVNGKKAAGKLTDGVLMVLSQAYFPVLAAPASANALDKGVLDVLIDAPDDADNLDHGRE
MAIPAPSIHAETHPLYAEDEIDKIAWIAPMHLLGTLPAVLGFEPDQYSFISMMIEASLQITKTAEALDDY
HVLEKGARPPGFKVILIKGLHIRLLAAVGTILVVGSAGRDEITAYIEGRAVPITLRKNTFLPAFAPSSCTVV
DILSTLLRPPERSTVQMLILETVAEL-
LEVTRIENGKLEKLQALLPGLDMAELLAILAAAEDFSAIVTIAAAIAAILSVAAEAVVLRELLLTKAEIAITAL
ALALALLLMLTRDSGLAQRERARDAQLKELIQGGAVLQLRVQEAMVLAATADKVRLSTDEGTTSAFA
SLNLATVSDGFYVMSSAVMQMNAQLMMVVTVARVLADKESKNALVVRHPEQVKTQPTYAEGSPG
MVLQTLTACVPPLAVKSEEVELLWTLVAALWRVKAHLAQATRPLEERLLILLLALSLIVVLLQSEGGGM
GGG-
VSSAAKILGAFSLLTIKASVGDEVRKVTRRVMDLNSRLGRPEEAVTPEFRGAELEQSKYEAENVEVDCV
ETVRRFVDIPFLVSIRTQHNESEDRGARLVLDVTRVRFIDL-
VTKRSHVILVESVETDGIIAREMAKDLQALHPELAALGKIAALDHFISLSDGHISNYVTAGINSVLGMLLL
ILFIQQVRVSKVTWPTRREVSMVIALTALAVFFLVIGL--
KIVFMLGGISWQQITTLVLGEGVMAVRQTNTFPADS-
ITILQNTYAELICAGRVTKADIEKSLHIVVAARVSRGTWLVIYLYEITQAQGEEGIFQRAFRQATLSQSLY
DGRMEVKIKVVSSDRYA-
TYEIKGGQHRKGTRFNRVKIAHNIPVEVLPRLKMAFGDPNLILVKTFGAVVQKRFASPLTHIRHGAVTI
GPMEVASPYITSKKARHAPGDSAVGVKPDNIVRSVAVVPELIWARENEDNGAVHDRYVKTTHKLAAK
RTTAVDKAQYDKRNQVHLSFIGLAVKVLPDKAIVHATAGTLLPTEAVSVTEGLKGVAATIRLDPMEKA
LIAYIKRMRPQLNLAGKADKKIVKCLNNNLDDVEFGKLGRRRIKPLTIKSVYDVEHEKATELVKLIPYKD

DVEGRKSKFADK--
ALFEPLQTFMMLFGFIARAVFALSLRVVRRTILLAKTRIVVNANTVLSAIKVIGVSIRLRHKSTNVADTKH
FIKTIEERARMTLAGAPSVKLNLRPGRVGKKGGRIKGYMPYKNFNIFDKLEAGRRAKVKELAKLKKVDKE
ITVDGIRTRQQGGRLPKADLSNALANTRVGVDIVGENIQEESNPRANAEMGQIFAAIDARVYHLVGA
LTGHALAAVLALCAIYGIGRAHALLAVKLKINAGIEFHSAA-VEAAVAAA-YAQLAGEAG--
ADVGEAYLGDLAVTDDSLDTANRPSRSISELKKTKKEAYLSLTCFSGKGEKIKILDASQTQAYEVVATRT
AERHGIKAKGTYGKERAMMQIVPSKPVKVWVPVFVSDEELLAPIRAFAESNGDGVEVVKLTKDVKVT
MFEKAVASLAVAGKSTSIVYTVLEKVTELSKDIIYEQRAKAHYGRGRLRSHVVTTTKQKRHLSMIKRTVV
AHPRMDASRLTDGEVTVITGATMAFVMSEQDAV--
GDVDLHFHEEPRREAALGIAGPETFLTTEQPRVLALLFLREYLIHIVDVGTKNGFADAATIFDILRAMSN
KLDRKLSAEIAKTRMVLHDDVKGMGVKAESPRRDKAGDAILSAYWINGVPANADIILLVANMECATA
PENINCAVRLFYQRITLQAKERDGDVSVRLIPAQNQIEAAEGTEIYTIKGNNPGTAQMKLNVEAISRSIV
KSLAVQLFQVDLFIDDTWIATLLRAALDVVFVEAKEGHEAVDHVIERDTELELAPQKDIVARKIRIYTPA
APSAVVIWDPAIDREEKADAQGTILKIYGPDLQDIACFTDIGAEPKIESLKAWDEKEKYVVHRDDIPEDI
TTMVGKSSRVNKEFGIPDVAVCAPAKPGVTESIDRGEPKAGSCGEVANLPYPQESIVNRVSSANVQK
NISMVLSGNPVGEMGISREVERHLETNMCEIFVDKDDVDVAMDFYTIKLTDLRVGMDKPNAKVQVE
GKESRKLMAVLDGEADLQRHPKVSEEGGTRVGAGTKAKLPDDALILDGVHHGDVKFTNTDLPEGRV
-
VTPVVLAVKAQLKIDKVLVKTFHVVTLSFSNDKGEEHLIKLRLNALKKSTRHYEMLLKIRETECAESPHAI
PVGKILKIDELLQTICEAHYTAIEPLLADDLTALGVMGDQGLLHVVPRLMILAVLHVWGVEAARIVAD
WDLQMAAGITDATMGLRVRARDWTY-AISEVVGNTFFARTKDDDTEERIHARNPI-VLL-
YGKEGMV-
ISPSGLHLDSTTEDTLQHGHDEHLILSAERLKDEMGYYVLKVLLVFVDEIILAWGSGEDPIKFLVAEQAV
TGRANPSLVCLTRRTFPLDLQRDATFVLVFADSTMKNLSHRKMHMNLVLIKSKGWIENVSTKILISDQ
AIASFAEDAGAHTQPSESALAVITSRVIAPIVIVLSQKPTAPSKTMFLEALNLYEEITATVLNSPKRYYIFEG
DVISVLGRLDAAVLLGMQRPQPDRMRRAVVAAAIRLAIAIKASGITQVLDAGLVNFSRAVSLAARCYG
DLAV-HVPVIG-AAIAFRVRNHKTSTKRDRPYKGHTKFLHKEDEDKKLIAEGVDTLA
>GCA_014199395
AVIKPVTMNTYAPVLMIAQSQVATVSGAGSPSLRAKTVYAKSGLQRKTESKSVALPIYY-
GVVKVSNIVQLVSISVDSRAVCTVAQLVEQEVVVRGMVALDVVKKKIALEAIEGMTAEDPAKIMDY--
EIEIRKFRDVEDRADLLPVMLGLTLKIRFDPHEPVVHDLAIPVVFEVV-
GIMVQRGQIEIDECGVSTAISDVDPIVYVSPIDLNAVAGDKMAYIDQEGAVSAAKDEIALEELALESAV
LVVGI-PLLIPV-FEADGDV-MSFANRSRTLTTIVNLAVGYPEVKRA-----IASAM-
MDQSQAAALAEKKGTAEEIRIVKRMQRSAGLADDADLVILDDAAEGNSSWAGQIYDDPLSGSSESES
VGNIVYAANKRQNFARSITELRKVEISDQAAAPLMRGVKHNAKSRSSRKIDDAIAVLNFPNVPEGIAIR
AEQGKPADTLLHEAAEGEEILLLIVRWHVTWKTAIIIYLMDDAGDIKGMITLGAVGAVGRKEGILNRHH
KMNGSLIEQKRVIEKLTLRLRAALKQRLGKDQVIIFVVKVAGDLVAAATIQM---
NVGAVVAVQGPTIFVRRSRQTVIGDQEATVEVLKGVAEGPADAEALLRIRVDHTVVKLTVLLIPDVPVL
VESVTIREGVGKALQIIQLDVYLLRDMGTGEETLAKSKSKEQIRSVLQVDPITGVRLAYGKLRERLVKGG
SQKELGTL---
LSILKNVTEPRMDNLPEGVCRTFIAAGPRAAVGMLFLILDDAGKIYTIDGDLQENNKTIKSMIGLVDAA
KPKILSAGYLTGRLKIRYADVQVLISEADLYAYAEPRNELMRMVPVYITNAAKRRQLILFALSLRGGHRR
ARMSLNVMLAAAILESQAEQGAVNHEDPDVLLDDGEIVGSEMMISRQSSLDIKSVLKVDVIVIFPEFQ
EKMVVSLKFFCTDVAIFVIALICTVGEMLSREPKHAQEGGAGFGKRIRKHSVLFALAATFDRTNCRDT--

PDLFAGLGLADPRYGILYTVQPKRKQMPSEPDADRTDGFELVHAIKADTTD-
EALDMPEAMALAVEATESINVSAGVLAMSGDMTAVEKVDSKRKMAVAVEKWTISKIPVVDVNISGA
APFRCQE-IEQADVSAGLPIKAL--
LFLPGANNAELEEEKLDGAGSSTIDAAADKQAAILLVNPVLVVIQDSATASLISNGGLEEWGTMAYKK
HYAFAARMRLHMRTIDAIEVGVSMNPNKQKAEKIKTVKLEGDTRYRQLATDGVKEQMP-
LHALNPDFAVIGPVLVTPAVGRVAADADVLGAEVPCDFSL-
RELQKSQKGNALPISGIRLLGMRAARAMVEEDIMVAGKVAA-
LLVIGGLETICKLVLRNDSEATLPWRLSVAERIAADTKRAGGYAARPFNSKPSLCKTQAHAKIADPIQEI
AAGYVAVDERFVFPVEGIPIQLILANPHSRIGGAKRAGLGHLHEMAKELVGKAVVMVMNEPGTNTEG
LMNYLTTGQPTDLLFEDCLDVVIDMRRMSVV--
VQLNTV-
HVVVAGVRPVGAFEIMDASADSGLVQVERVDERTGRENTLMYMYGAMEELWLNSIRYADWIIDCTI
GAILLHVYAQAINRAFSLERLQLAGWPKEATSWWNTWQLWLNKDGFFSYDAAFRA-
KAVLKADLYDFELKDVEGVSRMLSRQEVRAGYRIREQITYEQLTPRTDILLQSSTRFSIIRHAL-
MVAILIVREIVVEFT--
VAKEDVAMGHVECVLADSFDFTAKNADIGPVGLKTPDPVLGDGTVLNEAVLGKIDLANTVADFRYK
MLIDGGRALGVQWITMQNMLYARIWIPPAPAALKNLQPIKFFLSHPNLDLQSAAVIYEKISKARAEKL
MASALIEKILEKVARFAKPEATTHQSKKLRHFKYYGEEVDPVHLAYILELFALVATQVTVSDRLLKDRYVL
NLAIFRGFTSLLWLSAQLGDKSPSATIISLDSIAGLGALVLFIILL-RASVLVETLYYKYVY--
MLGYNAAKDVADADPEHFLVLAAASRDVEWIEVIPSFFYYFKTATSRLTKNTALAREFFGLFAERLRVID
VLVVPPDRVEALVEFVALAGTLVTSAEA-
VGVIACGFCEPNAAQVLEADQDWNLHRSLGITLAFFEARESVKGYFDKGLHKAVAEIGLTEAGALTTP
FHAAVTAKRCIPAVHRVIDLGEVEAALIEIDSAVVVADEEQTLADHPTTRDNSVGAVGGVVLAGTMN
MGANLDSVERAGNIIIELEEENYGAVNGKKNDGKLTDKVVMVLSRAYFPVLERAASANALDKGAFDL
LIDAPDDTENQDHGRTLTIPAPSIHTESAILFAESDIERIAWIAPIHLLGTLDAVLGFEPDQYSFISMMINA
SLDIMETAEVLDDYHVLEKGSRPPGFRVVLIKGLHIRLLAGIGTIIVVGSAGRSEVTAYIQG-
AVPITLNRVTFLPAFAASSCHVVDIFSTMLRPPERSAILTLILETVTEL-
LEVTRIENDKLERMDALLPGLDVAALLPLLAAAKDFAAIVTIDRAIAAILSVAAEAVVLREMLLNLAEIAV
TALALGLALLLMLTRDQGLHQKENAREAQMKALIQGGVVLQIKATDRMMLASTADKVRLDTDEGTT
SAFASLNLATVSDGFYVLSSAVLQLSSDLMMIVAVARVLADKETKNALVVRHPEQVKTQTTYAEGSP
GMVLQTLTGAIPPLAIKSEEVELLWTLVAALWRVKAHLANAARPHLERILILILALSLIAVLMQSEG-
GSMGGGSGAPVKILAAFSILTVKAKAGDEVRKVARRVMDLNSRLGRPEEAVSPEFRGAELEQVKYEAE
NVAVDTMETIRRFVDIPFLVSTRTQHNESEDRGSRMVLDVTRIRFIDL-
VTKRSEIILAARAETDGVIAREMKGDLAALHPEAAALGKIAFLQHFIAVQDGHISNYVTAQIHSVLGML
LLVLFINETRVAKVVWPSRREVTMVFIMATLAIFFLVIGLG-
QLVFMLSGISWNQITTIVLGEGVMAVRQTSTFPADS-
IGILQNTYADLICSGRVAKADIEKSLHIVVAGRVSRGTWYVIYHYDITQAQGPSGIMQRAFRQATLSQS
LIDGRMEVKIKIVTSDRYA-
TYEIKGGQHRKGTRYNRVKIGHNIAVQVLPRMKMCFGPPNLVLVKTFGAVVQKRFAQALTHTRHGA
VTIGPMEVSSPYIKNKKARHAPGDAVVDLKPDNILRSVAVVPELIWARENEDNGAVHDKYVKTTHKL
AAKRKKAVEKAQYDHRNQVELSFIGLAVKVLPDKAIVHATSGTLMPTASVAVTEDMKGIAATIRLDPM
EKSLIAYIKRMKPQLNLGGRTEKKIVKCLNNNLDDVEYGKLGRRKIKPITIKSVYDVESERATELVKLMPY
KDDVEGRKSQFAEK--
ALFEPLKAFMMLFGYIARAIYALYLRVIRRTILLVKTKIVVNANTVLSAVKIIGVSIRLRHKSTNVAEТNHFI

KTIEKRAKMTLAGTGKVKLNLSPGRVGKKGGRIKGFMPYNNYNIFDGLASGRRAKVKELAKLKSVDKTI
SVDGIRTKQQGGKIAKAQLSNAIANTRVGVDIVGEEVQEESNPRANAEMGQIFAKIDARVFHHVGALI
GHVLMAVLAIVSIWGIGRAHNLLAVAFRVNAGIEMHSAA-VDAAVAAA-FAEPGGEA-
VIADVSEGDLGALLISEMSVDVCRLRRRSLGELKKTKKDAYMSLTCFGGKGDKVKIVEATQTQAYEIVA
TRTAERHGIRAKGSFGKERAMMQITPTKPVKIWVPVFVSEAELLAPIRAFAETQGDGVTVVKLTTDVKV
TMFEKAVASLAVSGKATTLIYTVLEKVTQLMKDIIYTQRAKAHYGRGRLRSHVVTHTKQKRHLSMMKR
TVVADPSMDAARLTDGEVAVITGATMAFVMSEQDAV--
GDVDFHFHEEPQREAELGIGGPETFLCTEQPRVLALLFLRAYLLHIIEIETSNNFSDAATIYDVLRAMVNK
LDRKLSADIAKTRMCLTDNLLDMGVTDVAARRNAAGDKILSAFWINGVTANADIVLLVANLECPCVP
ENIDCAVRLFYQRITLQAKERGGDVSVQVVPAKNQIEAAEGTVIYSIKGKTPGTGQMKLTIEGISRSIVK
SLAVQLFQVNTFLDDTWIGTLLRAALDVVFVQAEESKEAVDHVIERDTEFEFAPQKDIVSRKIRIYTPAA
PSAVVIWDPAIDREDKADAQGTILKIYGPNLQDINCFTDIGAEPKIESLKAWDEKEKYVVHRDDIPDDI
DTLVGKSSKVNKEFAIPDVAVCAPSKPGVTESIDRGEPKAGTCGEVANMSYPQESLVNAVSSANVQK
NISMVLAGHPVGEMGISREIERHLETNMCEIFVDKADVDVALDFSVIGLTALRVGLDTPNARVEIDGKE
SRKLMAVLDGEAALQRHPKVSAATGLT-KAGTKARMEDDALIIDGVIHGDIPFTATDLPDGRV-
VTPVVNKVKAQLEIDKVILRTFHVVTLSFSNDKGEDHLIKLRLNALKKSTRHYEMLLKIRETECAESPHAI
SVGKVLKIDKLLQTICEAHYKAIEPLMADDLTALGVMGEQGLLHVVPRLMILAILLLWGVEALQLVPV
WDLQEQAIVTDQTMGL-ARDRDWSY-AISEVVSNTFFARTADDDTEQRIHARNPV-VLL-
YGKFGMV-
ISPSGLKLKSTAATDQDRGNDEDLILSTERLQDEMGYYVLRILLVFVDQVILAYGSGDKPIKFLVANQA
VTGRQNPGMVSLTRRTFPLELQRDATFIMVFDDSIMKNLSHKKMHMNLVLVKSKGWVVNVSTKILIS
DQAIASYAEDAGAHTQPSESALAIITSRVMAPIVLVVSQKPTAPSKTLFLEALALYDEVTATVLNKPKKY
YIFEGDVVSVLKSLDAAVLLGMQRPQPDRMRRAIVATAIRLAIALRAADIVVRLDNNLINFSRAVTLAV
RVYIILSDIIMIAY-----AFRVRNHDVSTKRSRPYKAHPKFLHKAEIDKSLIAEGVDTIA
>GCA_018263905
AVIKPVTMGVYAPVLMIAQSQVATVSGAGSPSLRAKTVYAKSGLQRKVETKWVAKDFYY-
GVVKMANLVQLVSISVDNRAVCTVAQLVEQEVVVHGMVALEAVKKKIALEAIEGMTAEDPAKVVDY
QTRIAIRKWRDADDRADLLPVLLGLTLKIKFDPHQPLVHDLPIPVIYEVI-
GIMAQKGTIEVDDCGISTAVSDVDPIAYVSPIDLAAISGQKMDYIDQDTAVSAAKQDIDLEELALESAV
LVVGIRPLIIPI-FRDDADT-MSFANRSRTLKTIVDLAVSYPDQTRAR---AIAAAM-
MDASQAAALAEKRGTAEEIRIVKRMQRSAGLADDPELVLLEDAAEGNSNWAGQIYDDPISGSSESDS
AGNIVFAANKRQNFARSITFLRQVEISDQAAAPLMRGVKHNAKSRSSRKLDDVIAALNFPNVPEGIAI
KAAAAKPAETLLHDADEGERILMLIVRWHVTWKTAAIIYLLDLAGDIVGLIELGGIAAVGRKDGLLNRH
HKLNGSLIEQKRVIDKLTLRLRAALKQKLGADQVIILVVRVQGDLVAEAEIQMG--
DVGAVVAIQGPTIFVRRHRQTVIGDQEATV-VLKGVAEGPADAQALLRIRVDHTLVKLSVLLIPE-
PVLVESVTIREGVGRALQIIQLDVYLLRDMGTGEETLAKSRSRELIRELLQIDPITGVRLAYGGLRERESKA
LSIKEIDTLFE-
LAILAGVTEPRMQNLPEGVCRTFIRAGPDAAAGMLFLILDDAGKIYTIDGDLREITKSIKSLIGLVDAAKP
KVLSAVYMTDRLKIRYADVHVLTSDADLYAYAEARRELLRMVPVYITNAAKRRQLVLLALSLRDGHRR
VRMAIDVMIAAAIIESQADNGAVHHAEPRILLDDAEVVGSEMMITRQAALAMKEVVKVDVIVIFEEFQ
EKMVVSLRFFCTDVAIFVIALICTVGQMLSREPSHAQEGGAGFQK---AHSVLFALVATFDRVNLRCA-
A-
GLFMGLGLAPPRHGIIYTVRPKRKQMPSEPDKDKVDGFELVHAVKEDARENHKLDMPEAMALAVE-
DTSINVSGGVLAMDGDMASLEKVDSKRKMALAVTKWNISKINLDDLVISGAKPFRALS-

IEEADMTAALAVKTL-
FLYLPNANNAELEADKVDGAGSSTVDAAADRQQGILLLNPVMIVIQDSNTAALISNEGLDEWGTMA
YKKHYAYAARMRLHMRTIDGIEVGVAMEPNKQKAEKIKTVKLEGDTRFRQLATEGVKEQMP-
LHALNPDFAVLIAILVTPAVGRVAADQDVLGARVPCDASL-
RELQKSQKGDALPISAIRLLGMRAARAMVDEDIMVAGKVSASLLVIVALETICRLVLLNDSEATLPWRL
SVAERIEADTARAAGYAARPFMSKPSLCKVQAHANVADPIQEIAAGYVAKPNRFVFPVEGLAIQLILAS
PHSRVGGAKRAGLGHLHEMAKELLGKADLMVQAASGTNTEGILTYMTTRQPTDLLHDDCLDIVIDM
RRMSVV---VQLNTV-
HVVVSGVGDVGEFEIMQAAAPSGLVQVEKIDDR-
GRERTMMYMYGAMEDLWLLSVRYADWVVDCAIGAIVLHVYAQTINRAFSLERLQLAGWPPEASSW
WNTWQLWLNKDGFFDYDAAFRAQAAVLAAALYDYDMTDIEAVGRYLSRQELRVGYRIREQVAFAR
LDPRTDILLQSQTRLSVIRHALEVVAIMIVREIVIEFTLVVAQEDVAMGHVECVLADAFDFTAKNADIGP
VGLKTPDPVLGDGTVLNDAVLGKIDLANTVADFRYRMIDDGGRQLGVEWLTL-
NMLYTRIWLPPAQAALKNLQPICFFLGHPDLNLALLADLWAGISKARMQALMASALIEQILDKVAAF
ADPDATTHQSNKVKHFKYYGEEVDPVYLAYILKMFALVATQVTVSDRLLKDRYVLNLKIFRGFTSLWW
LGAQLTDKGPAATIIALDSIAGLGALVLFIILL-RDSVLVETLYFRYVY--
MKGYEAAKDVADADPEHLLNLAAVTRDVEWIEVVPAFFYYFKTAG-
FEPFNTAIAREFFGLFRERLRIVDVLVVPPERVEQLVAFVALAGTLPTSAEAQPGIIACGLCEPNAAGVLE
ADQDWNLHRSMGITLAFYDARESVKGWFDKGLHKDVAATGLSEAGTLTTPFHAAVTAKVCVPALHR
VLDLGEFEASLIELDSAVTVADEEQTLADKPTTRDNNVGAVGGVVLAGTMNMGANLDSVERGNNIII
ELEEESYSAVNGKKDG-
KLTDKVLMVLSGAYFVVLTRIASANALDRGAFDLLIDVPNDADNRDQGREL-------------------
----
PDQYSFISMMIEASLDIMKTAEVLDDYHVLEKGSRPPDFRVILIKGLHVRMLAGIATIIVVGSAGRSEVT
AYIQE-AVPITLRRVTFLPAFAASSCHVIDIFSTMLRPPERSSVKMLILETVAEL-
LEVTRLENDKLERMEVLLPGLDLAQLLPLEAAADDFPQIVTIGAAIVAILSVAAEPVVLRDMLLSLAEIAV
EAMALALALLLMLTPDQGLTQKETAREAQMKELIQGGVVLQVRASDRMLLAATADKVRLDTDEGTT
SAFASLNLATVSDGFYVMSSAVLQLSSDLMMIISVARVLADKEHKNALVVRHPEQVKTQTTYAEGSP
GMVLQTLTGAIPPLAVKAEEVELLWTLVAALWAVKAHLANATRPRKERVLILILALTLITVLLQSEGGGI
GGG--
GPAPKFLAAFSILTIKAKAGDEVRKVTRRVLDLNSRLGRPEESVNPEFRGAELEQSKYEAANVEVDTME
TIRRFVDIPFLVSTRTQHNESEDRGARMVLDVTRVRFIDL-
VTKKSEIILAARAETDGIIAREMKGDLAALHPEAAALGKIAALQHFIAIQDAHISNYVTAAIYSVLGMLLL
VLFINETRIAKVVWPTRREVTMVFIMAALATFFLVIGLVVSIVFMLAGISWNQLTTIVLGEGVMAVRQT
STFPKDS-
IGILQNTFADLICAGRVAKADIEKSLHIVVAGRVTRGTWYVIYLYEISQAQSPAGIMQRAFRQKTLSQSL
IDGRMEVKIKVVTSNRYAQTYEIKGGQHRKGTRYNRVKVGHNIPVDVMPRMKMAFTPPNLVLVKTF
GAVVQKRFASALTHMRHGAVQIGPMEVSSPYIRNKKARHAPGDAVVDLKPDNIVRSVAVVPELIWA
RENEDNGAVHDKYVKPTHKLKAKRKTAVDKAQYDHRNQVELSFIGLAVKVLPQKAIVHATAGMLMP
TTSVAVTEGMKGIAATIRLDPMEKALIAYIKRMRPQLNLEGRTDKKIVKCLNNNLDDVEYGKLGRRKIK
PITIKSVYDVESRRATELVKLLPYKDDVEGRKSQFAEK--
ALFEPLKAFMMLFGYIARAVYALNLRVIRRTILLVKCKIVVAAATVLSAIKIIGLSVRLRHKTTNVADTKHH
IKTITARANLTLAGAGKIKLNLSAGRVGKKGGRIKGYMPYNNFNIFDGLESGRRAKVKEVAKLKKVDKEI
KVDGVRVKQEGGRTPKAQLSNAIANSRVGVDIVGENVQEQSNPRANAEMGQIFAAIDARVFSHVG

ALIGHALEAVLAVVAIWGIGRAHNLLALKLRINAGIEMHSAA-VDVAVACA-
FATAAGEAAALWDASEGYLDDALITQHAVDTCRLRRRSLSELKKTKKDAYMSLTCFAGKGDRFKVVE
ATQTQAYEIVATRTAERHGIKAKGTFGKERAMMQILPTKPVKVWVPVFVDEVELLAPIRQFAETVGDG
VTVVKLQNDIKITPFEKAVASLAVSGKSTSVIYTVLEKVTELSKDIIYTQRAKAHYGRGRLRSHVVTHTKS
SRHLSMMKRTVVADPSMDAARLTDGEVSVVTGATMAFVMSEQDAV--
GDVDMHFHEEPRREADVGIAGPETFLATEHPRTLAVFFLRAYLIHIVEIQTVKGFANAATIYDVLRAMQ
NKLDRKLSADIAKTRMVLVDNLKDMGVSEVAAKREAAGDPILSAFWINGVTANADIVLLVANLEVPT
VPENIHCAIRLFYQRITLQAKERGGDVSVAVVPANKQIEAAEDTVIYSIKGKTPGSGQMKLTIEGISRSIV
KSLAVQLFQVDTFLEDTWLGTLLRAAIEVVFVDAGDSKEAVDHVVERDTEYEFAPQKDIVSRKIRIYTP
AAPSAVVIWDPAIDREDKAEAQGTILKIYGPNLQDINCFTDIGAEPKIESLKAWDEKEKYVVHRDDIPE
DI-
TLVGKSSRVNKEFAIPDVAVCAPSKPGVTESIDRGDPKAGTCGEVANMAYPQESLVNNVSSANVQK
NISMVLAGHPVGEMGISRQIERHLETNMCEIFVDRDDIDVALDFYILALTPLRVGLDPPNARVQIDGKE
SRKLMAVLDGEAALQRHPKVSQAANLT-NAGIKARLPDDTLVVDGVVHGDISFTATDLPDGRV-
VTPVFNTVKAQLKIDKVILKAFHVVTLSFSNGKGEEHLIKLRLNALKKNTRHYEMLLKIRETECAESPHAI
SVGKVLKIDSLLQTICIAHYKAIEPLLADDLTALGVMGHQGLLHVVPRLMIL--
LHLWGVAARMELAHW-LQAEA-VTDQTMGLQVRERHYSY-
SISEVVGNTFFAKTKEDDTEQRLHARNPI-VLL-GAKFGMV-
ISPSGLKLRSTAEPTIGRGNTEDLILSTERLQDAMGYYVLQILLVFVDEVILAYGSGEDPLKFLVGDQAVT
GRENPGMVTLTRRTFPLDLQRHAVFVMVFEDCTMKNLSHKKMHMNLVLIKSKGWIVNVSTKILISDQ
AIASYAEDAGAHTQPSESALAAITSRVMAPIVLVVSQKPTAPSKTLFLEALALYDEVTATVLNKPKKYYIF
EGDVVSILGTCDAAVLLGMQRPQPDRMRRAVVADAIRLAIALRASRVLDHLDNGLINFARGVSFIRGE
HATLAPLLSAAIGPPAVAFRVRNHAVSTKRSKPYKAHTKFLHKADINKSLIAAGVDTIA
>GCA_020531945
AVIKPVTMNAYAPVLMIAQSQVATVSGAGSPSLRAKTVYAKSGLQRKSETKWVAKDFYY-
GVVKMANLVQLVSISVDNRAVCTVAQLVEQEVVVHGMVALDAVKKKIALEAIEGMTAEDPAKVVDY
QTRIAIRKWRDADDRADLLPVLLGLTLKIKFDPHQPLVHDLPIPVVYEVI-
GIMAQKGTIEVDDCGISTAVSDVDPIAYVSPIDLAAVSGQKMDYIDQEAAVAQAKQDIDLEELALESS
VLVVGIKPLIIPI-FRDDADE-MSFANRSRTLKTIVDLAVSYPEQTRAR----IAAAM-
MDASQAAALAEKRGTAEEIRIVKRMQRSAGLADDPELVMLEDAAEGNSNWAGQIYDDPISGSSESD
SAGNIVYAANKRQNFARSITFLRTVEISDQAAAPLMRGVKHNAKSRASRKLDDAIAALNFPNVPEGIAI
RAAAGKATATLLHDADEGEQILMLIVRWHVTWKTAAIIYILEVAGNIVGLIELGGIAAVGRKDGLLNRH
HKLNGSLIEQKRVIDKLTLRLRAALKQKLGQDQVIILVVRVQGDLVAEAEIQMG--
DVGAVVAIQGPTIFVRRHRQTVIGDQEATV-VLKGVAEGPADAQALLRIRVDHTLVKLSVLLIPE-
PVLVESVTIREGVGRALQIIQLDVYLLRDMGTGEETLAKSRSRELIRELLQIDPITGVRLAYGNLREREAKV
LNIKEIDTLF--
LAIIQGVTEPRMGNLPEGVCRTFVRAGPRAAAGMLFLILDDAGKIYAIDGDLREITKSIKSLIGLVDAAKP
KALSAVYMTDRLKIRYEDVHVLISDADLYAYAEARRELLRMVPVYITNQAKRRQLVLLALSLRDGHRRV
RMAIDVMIAAAIIESQAVNGAVHHAEPRILLDDAEVVGSEMMINRQAALAMKEVLKVDVIVIFPEFQE
KMVVSLRFFCTDVAIFVIALICTVGAMLSREPSHAQESGAGFQK--RAHSVLFALQATFDAQNLKDA-
-ANLFIGLGISAPRYGILYTVRPKRKQMPSEPDADKVDGFELVHAVKEDGRDN-KLDMPEAMALAVE-
DATINVSGGVLAMDGDMAAIEKVDSKRKLALAVTKWNIARVSIQDLTISGARPVRAFD-
ITVAQVTAALGEKVVS-
LYLPKANNAELEADKIDGAGSSTVDAAADRQQGILLLNPVLVVIQDSNTAALISNEGLDEWGTMAYK

KHYAYAARMRLHMRTIDGIEVGVAMEPNKQKAEKIKTVKLEGDTRFRQLATEGVKEQMP-
LHALNPDFAV-LAVLVTPAVGRVAADQDVLGARVPCDASL-
RELQKSQKGDALPISGIRLLGMRAARAMVEEDIMVAGKVAA-
LLVIVAVETICKMVLLNDSEATLPWRLSVAERIEADTARAAGYAARPFMSKPSLCKVQAHANVADPIQ
EIAAGYVATEQRFVFPVEGLPIQLILASPHSRVGVAKRAGLGHLHEMAKELIGKADLMVQAASDTNTE
GILNYLTTRQPTDLLHDDCLDIVIDMRRMSVV---
VQLNTV--
VVVSGVRDVGAYEIMQAAAPSGLIQVERIDDRTGRERTMMYMYGAMEELWLLSVRYADWVVDCAI
GAIVLHVYAQVINRAFSLERLQLAGWPPEASSWWNTWQLWLNKDGFFGYEAAFRAQAAVLSAALY
DYEMTDVEDVGRYPSRQELRVGYRIRAQVAFDRLDPRTDILLQSRTRLSVIRHALEVVAIMIVREIVIEFT
LVVAQEDVAMGHVECVLADCFDFTAKNADIGPVGLKTPDPVLGDGTVLNDAVLGKIDLANTVADFR
YRMIDDGGRQLGVEWLTLRNLLYSRIWLPPAEAALKNLQPICFFLGHPNLNLHLLDALWAGISKARM
QALMASALIEQILDKVAAFADPDATTHQSNKVKHFKYYGEEVDPVYLAYILQGYALVATQVTVSDRLL
KDRYVLNLKV-RGFTSLWWLGAQLATKSPAATIIALDSIAGLGALVLFIILL-RDSVLVETLYYRYVY--
MKGYEAAKDVADADPEHVLNLAAVSRDVEYIEIVPAFFYYFKTAG-
FEPFNTAVAREFFGLFKERLRVIDVLVVPPDRVMSLVAFVALAGTLATSAEALPGVIATGLCEPNAAGV
LEADQDWNLHRSMGITLAFYDARESVKGWFDKGLHKDVAQTGLSEAGALTTPFHVAATAKACVPAI
HRVLDLGEIEASLIEIDSAVTVADEEQTLADKPTTRDNSVGAVGGVVLAGTMNMGANLDSVERGNNI
IIELEEESYSAVNGKKDG-
KLTDKVLMVLSGAYFVVMERIASANALDRGAFDLLIDVPDDADNRDQGREL------------------
------------------------PDQYSFISMMINASLDIMQTAEV--
DYHVLEKGSRPPAFRVILIKGLHVRHLAGIATIIVVGSAGRSEVTAYIQG-
AVPITLKRVTFLPAFAASSCHVIDIFSTMLRPPERSTIKMLILETVAEL-
LEVTRLENDKLERMEVLLPGLDMAQLLPLEAHAADFPQIVTIDASIVAILSVAAEPVVLRDMLLSLAEIA
VEAMALALALLLMLTPDQGLTQKETSREAQMKELIQGGVVLQVRAADRMVLAATADKVRLDTDDG
TTSAFASLNLATVSDGFYVMSSAVLQLSSDLMMIISVARVLADKEHKNALVVRHPEQVKTQSTYAEGS
PGMVLQTLTCAIPPLAIKADEVELLWTLVTALWAVKAHLANATRPRNERVLILILALTLITVLLQSEGGGI
GGG--
GPPAKGLAAFSILTIKAKAGDEVRKVTRRVLDLNSRLGRPEESVNPEFRGAELEQSKYEAANVEVDTME
TIRRFVDIPFLVSTRTQHNESEDRGARMVLDVTRVRFIDL-
VTKKSEIILAARAETDGIIAREMKGDLAALHSEAAALGKIAALQHFIAIQDAHISNYVTANIHSVLGMLLL
VLFINETRIAKVVWPTRREVTMVFIMAALATFFLVIGLVISIVFMLSGISWNQMTTIVLGEGVMAVRQTS
TFPKDS-
IGILQNTFADLICSGRVAKADIEKSLHIVVAGRVTRGTWYVIYLYEISQAQSPAGIMQRAFRQKTLSQSLI
DGRMEVKIKVVTSDRYAQTYEIKGGQHRKGTRYNRVKVGHNIPIDVLPRMKMAFTPPNLVLVKTFGA
VVQKRFASALTHMRHGAVQIGPMEVSSPYIRNKKARHAPGDAVVDLKPELIVRSVAVVPELIWAREN
EDNGAVHDKYVKPTHKLKAKRTTAVDKAQYDHRNQVEMSFIGLAVKVLPQKAIVHATAGMLMPTTS
VAVTEGMKGIAATIRLDPMEKALIAYIKRMRPQLNLEGRTDKKIVKCLNNNLDDVEYGKLGRRKIKPITI
KSVYDVESRRATELVKLLPYKDDVEGRKSQFAEK--
ALFEPLKAFMMLFGYIARSVYALNLRVIRRTILLVKCKIVVAAATVLSAIKIIGLSIRLRHKSTNIADTKHHIK
TIEDRANLTMAGAGKVKLNLSEGKVGKKGGRIKGYMPYNNYNIFDGLASGRRAKVKEVAKLKKVDKEI
KVEGIRVKQEGGRTPKAQLSNAVANTRVGVDIVGEKIQEQSNPRANAEMGQIFAAIDARVYSHVGAL
IGHALEAVLAIVAIWGIGRAHNLLALKLRINAGIEMHSAA-VTHAVACA-
FAAPAGDASALWDASEAYLGAIAITEHAVDTCRLRRRSLSELKKTKKDAYMSLTCFAGKGDRVKIVEAS

QTQAYEIVATRTAERHGIKAKGTFGKERAMMQILPTKPVKVWVPVFVDEVELLAPIRNFAETVGDGVT
VVKLQNDIKITPFEKAVASIAVTGKSTSVVYTVLEKVTELSKDIIYTQRAKAHYGRGRLRSHVVTHTKSSR
HLSMMKRTVVADPSMDAARLTDGEVSVVTGATMAFVMSEQDAV--
GDVDMHFHEEPRREAEVGIGGPETFLATEHPRTLAVLFLRAYLIHIVEIETTKDFSNAATIYDVLRAMAN
KLDRKLSADIAKTRMVLVDNLMDMGVTEVAAKRDAAGDKILSAFWINGVTANADIVLLVANLEVPTV
PENIHCAIRLFYQRITLQAKERDGDVSVAVVPANKQIEAAEDTVIYSIKGKTPGNAQMKLTIEGISRSVV
KSLAVQLFQVDTFLDDTWLGTLLRAAIDVVFVDAGETKEAVDHVVERDTEYEFAPQKDIVSRKIRIYTP
AAPSAVVIWDPAIDREDKADAQGTILKIYGPNLQDINCFTDIGAEPKIESLKAWDEKEKYVVHRDDIPE
DI-
TLVGKSSKVNKEFAIPDVAVCAPSKPGVTESIDRGEPKAGTCGEVANMAYPQESLVNAVSSANVQKN
ISMVLAGHPVGEMGISREIERHLETNMCEIFVDKADVDVALDFYILSLTPLRVGLDPPNARVSIDGKESR
KLMAVMDGDADLQRHPKVSQATGTG-NAGIKARLPDDTLIVDGVVHGDVSFTATDLPEGRV-
VTPVFNDVKAQLKIDKVILKAFHVVTLSFSNGKGEDHLIKLRLNALKKNTRHYEMLLKIRETECAESPHAI
SVGKVLKIDSLLQTICVAHYKAIEPLLADDLTALGVMGHQGLLHVVPRLMILAVLHLWGMAAAMEVA
QWGLQANA-VTDQTMGLALRERHFTY-SISEVVGNTFFAKTKEDDTEQRLHARNPI-VLL-
AAKFGMV-
ISPSGLKLTSTQASALGRENAEDLILSTERLQDAMGYYVLRILLVFVDEVILAYGSGERPIKFLVGDQAVT
GRQNPGMVTLTRRTFPLDLQRHAVFVMVFEDCTMKNLSHKKMHMNLILIKSKGWIVNVSTKILISDQ
AIASYAEDAGAHTQPSESALAVITSRVMAPIVLVVSQKPTAPSKTLFLEALALYDEVTATVQNKPKKYYI
FEGDVVSILGACDAAVLLGMQRPQPDRMRRAVVADAIRLAIALR-------
LEAGLINFARAVSLAVGVYTRFQAPP-AAMP-
PALAFRVRNHSVSTKRSKPYKGHTKFLHKADINKSLIAAGVDTIA
>GCA_021735925
AVIKPVTIDDYSPVLMIKQQQVATVSGAGSPTLRAKTVYAKSGLQRKAETKWVAKPFYY-
GVVKMSNLVQLVSISVDNRAVCTVAQLVEQEVVVRGMVALVAVKKKIALEAIEGMTAEDPAKVVDY
AVRIAIRKFRDADDKSDLLPVLLGLTLKIKFDPHQPVVHDLPVPVIFEVI-
GIMAQKGTIEVDECGISTAVGDVDPINYVSPIDLAAVAGDKMAYIDQEAAVSKAKDDIALEELALESN
VLVVGI-PLIIPI-FREEADE-MSFANRSRTLRTIVDMAVSAVSQTRA----AIASAL-
MDQSQAAALADKRGTAEEIRIVKRMQRSAGLADDPELVVLEDAAEGNSNWAGQIYDDDISGSSESD
SAGNIVHAANKRQNFARSITFLRSVEIADQAAAPLMRGVKHNAKSRSSRKLDDAIATLNFANVPEGIA
IAAAAAKPAETLLHDATEGEQILMLIVRWHVTWKTAIVLYLM--
AGDVVGLITLGGMAGVGRKEGILNRHHKMNGSLIEQKRIIDKLTLRLRASLKQKLGAEQVIILVVRVQG
DLVAQAEIQMG--
DIGAVVAIQGPTIFVRRHRQTVIGDQEATVEVLKGVAQGPADAQALLRIRVDHTLVKLSVLLIPEVPVL
VESVTIREGVGKALQIIQLDVYLLRDMGTGEETLAMSKSRELIRELLQIDPITGVRLAFGDLREREAKVLNI
KEIDTLF--
LAIATNLTEPRMANLPDGVCRNFVRAGPRAAVGMIFLILDPAGRIYAIDGDLRENTKGIKSLIGLVDAA
KPKALSAVYATERLKVRYEDVHVLVSDANLYAYAEARSELLRMVPVYIINAAKRRQIILLALSLRDGHRR
VRMSIDVMIAAAIIESHADHGTVHHAEPRILLDDAEVVGSEMMISRQAALDLKQLIKTDVIVLFPEFQE
KM---RIRAHSVLFALTATFDKANLRRA---
GLFLGLGLAAPRYGILYTVQPKRKQMPTEPDKEELDGFDLVHAVKEDGRDNHKLDMPEAMALAVEA
TDTINVSGGVLGFDGDMAAVEKVDSKRKMALAIKQWTIAPKACLDLHISAARPFRALK-
LEPADVTAALEQKQLD-
LFLPGANNAELEAEKIDGAGSSTVEAAADRNRGILLLNPVLVVIQDSNTAALIANDGLDEWGTMAYK

KHYAFAARMRLHMRTIDGIEVGVQMEPNKQKNEKIKTVKLEGDTRFRQLATEGVKEQMP-
LHALNPDFAVLIAVLVTPAVGRVAADQGVLGARVPCGASL-
RELQKSQTGDALPISGIRLLGMRAARAMVDEDIMVAGKVAA-
LLVIVALETICRLVLLNDSEATLPWRLSVATRLEADAKRAAGFAARPFMSKPSLCNTQAHAKIADPIQEI
AAGYVAREERFVFPVEGLPIQLILSSPHSRVGGAKRAGLGHLHEMAKELQGRAVLMVLAASNTNTEGL
LRYATTRQPTDLLHDDCLDIVIDMRRMSVV---
VRMNTV-HVVIDGV-
PVGDFEIMQAAAASGLIEVEQVDDRTGRERTMMWMYGAMEELWLMSLRYADWIVDCTIGAIVLHV
YAQTINRAFSLERLQLAGWPQEAQGWWNTWQLWLNKDGFFGYAAAFRAVAAVLKADLYDYDMR
DVDGVGRYQSRQELRVGYRIREQVDFAKLDPRTDILLQSMTRLSVIRHALEVVAIMIVREIVIEFTLVVA
QEDVAMGHVECVLADAFDFTAKDADIGPVGLKTPDPVLGDGTVLNDAVLGKIDLSNTVADFRYRMI
DDGGRELGVTWLTLQNILYARIWLPPAAAALKNLQPICFFLGHPDLNLL-
LEALWAGISKARAQALMAGALIEKILDKVAAFAEPDATTHQSNKVKHFKYYGEEVDPVYLAYILDLFAL
VATQVTVSDRLLTERYVLNLKV-RGFTSVLWLAAQIGNKGPSATIISLDSVAGLGALVLFIILL-
RESVLIETLYYRYVY-VMKGYEAAKDVADADPEHLLNLAAVTRDVEWIEIVPAFFYYFKTAT-
FQPFNTAVAREFFGLFKERLRIVDVLLVPPDRVTALVEFVALAGTLPTSAAALPGVIACGLCEPNAAGVL
EADQDWNLHRSMGITLAFYDARESVKKWFDKGLHKPVAATGLSEAGALTTPFHVGATAKVCAPALH
RVLDLGEIEGSLIELDSAVTVADEEQTLADKPTTRDNSVGAVGGVVLAGTMNMGANLDSVERGNNIII
ELEEEQYSTVNGKKDG-
KLTDRVLMVLSQAYFPVLRRMASANALDRGTFDLLVDVPDDADNRDQGGEL-----------------
--------------------------PDQYSFISMMIEASLDIMETAEVL-
DYHVLETGSRPPDFRIILIKGLHIRQLAGIATIIVVGSAGRSEVTAYIQG-
AVPITLRRKTFLPAFAASSCHVIDIFSTMLRPPERSTIVMLILETVAEL-
LAVTRGTNEKLERMQVLLPGLDLAELLPLEAAAADFPAIVTIEAAIVAILSVAAEPVVLREMLLSLAEIAV
DAMALALALLLMLTPDQGLTQRELAREAQMKELIQGGVVLQIKAADRMVLAATADKVRLDTDDGTT
SAFASLNLATVSDGFYVMSSAVLQMGSDLMMIIKVARVLADKETKNALVVRHPEQVKTQTTYAEGSP
GMVLQTLTGSIPPLAIKAEEIELLWTLVAALWRVKAHLANATRAHNERVLILILALTLITVLLQSEGGGVG
GG--
GPAAKILAGFSILTIKAKVGEEVRKVTRRVLDLNSRLGRPEESVNPEFRGAELEQSKFEAENVEVDTMETI
RRFVDIPFLVSTRTQHNESEDRGSRMVLDVTRTRFIDL-
VTKRSEIILAARAETDGIIAREMSKDLAALHPEAAALGKIAALQHFIAISDAHISNYVTQEINSVLGMLLL
VLFINETRIAKVVWPTRREVTMVFIMAALATFFIVIGLFISIVFMLSGISWNQMTTIVLGEGVMAVRQTS
TFPADS-
IGILQNTYADLICAGRVAKADIEKSLHIVVAGRVTRGTWYVIYLYEISQAQGEAGIMQRAFRQKTLSQS
LIDGRMEVKIKVVTSDRYA-
TYEIKGGQHRKGTRYNRVKVGHNIPVQVLPRMKMAFTPPNLVLVKTFGAVVQKRFAQALTHMRHG
AVQIGPMEVSSPYINSKKARHAPGDAVVELKPENIVRSVAVVPELIWARENEDNNAVHDKYVKTTHK
LAAKRKKAVDKAQYDHRNQVEMSFIGLAVKVLPDKA-------------------------------------
------
RMRPQLNLAGKTDKKIVKCLNNNLDDVEYGKLGRRKIKPITIKSVYDVESRRATELVKLLPYKDDVEGR
KSQFAEK--
ALFEPLKAFMMLFGYIARAVYALNLRVIRRTILLVKCKIVVAAATVLSAIKIVGVSIRLRHKSTNVAETNH
HIKTIDKRARMTLAGDGKVKLNLSEGRVGKKGGRIKGYMPYNNYNIFDGLASGRRAKVKEVAKLKKV
DKEIKVEGIRAKQEGGRTPKPQLSNAVANTRVGVD-

VGENIQEESNPRANAEMGQIFAKIDARVYSHVGALIGHALEAVLAVVAIWGIGRAHNLLALRLRINAGI
ELHSAA-VEVAVACA-YAVPAGEASLMVDVSEGPKPALLITEASVDICRLRRRSLSE--------------
----
RHGIHAKGTYGKERAMMQILPTKPVKVWVPVFVDEVELLAPIRAFAETVGDGVTVVKLTTDVKITMFE
KAVASLAVAGKNTSVIYTVLEKVTQLMKDIIYTQRAKAHYGRGRLRSHVVTHTKSRRHLSMMKRTVV
ADPSMDAARLTDGEVSVITGATMAFVMSEQDAV-
PGDVDMHFHEEPRREAEVGIAGPETFLATEHPRVLAVIFLRAYLLHIVEIQTVKDFSNAATIYDVLRAM
NAKLDRKLSADIAKTRMVLADNLMDMGVTEVAARREAAGDPILSSYWINGVTANADIVLLVANLEVP
TVPENIHCAIRLFYQRVTLQAKERGGDVSVAVVPANKQIEAAEDTVIYSIKGKAPGSAQMKLTIEGISRS
IVKALAVQLFQVDTFLDDTWLGTLLRAAMDVVFVDAGDGKEAVDHVVERDTEYEFAPQKDIVSRKIRI
YTPAAPSAVVIWDPAIEREDKAEAQGTILKIYGPNLQDINCFTDIGSEPKIESLKSWDEKEKYVVHRDDI
PEDIDTLVGKSSRVNKEFAIPDVAVCAPSKPGVTESIDRGEPKAGTCGEVANMAYPQESLVNNVSSAN
VQKNISMVLAGHPVGEMGISREIERHLETNMCEIFVDKPDIDVALDFSTLALTVLRVGLDPPNARVQIG
GKESRKLMAVNDGDAPLQRHPKVSEATGTGI-AGVKATLADDALELDGVVHGDISFTLTDLPEGRV-
VTPVFNDVKAQLKIDKVILRAFHVVTLSFSNGKGEDHLIKLRLNALKKNTRHYEMLLKIRETECAESPHA
IDVGKVLKIDALLQTICVAHYKAIEPLLAADLTALGVMGEQGLLHVVPRLMILAVMHLWGLEATMALP
RWGLQTRRCVTDQTMGLATRARHYSY-CISEVVGNTFFAMTKEDDTEQELHARNPI-VLL-
CGKFGMV-
ISPSGLRLASTAPAPLGRDNHEDLILTTERLQDNMGYYVLQILLVFVDRVILAYGSGEDPIKFLVGDQAV
TGRQNPGMVTLARRTFPLELQRDAVFVMVFADSTMKNLSHRKMHMNLVLIKSKGWVVNVSTKILIS
DQAIASYAEDVGAHTQPSESALAAITSRVMAPIVLVVSQKPTAPSKSLFLEALALYDEVTATVLNAPKK
YYIFEGDVISVLNTCDAAVLLGMQRPQPNRMRRAMVADAIRLAIALRAAGIITRLEEGLINFARAVGLA
AGVYPDFGALVMAIIMADALAFRVRNHAVSTKRSKPYKGHTKFLHKADVNKQLIAAGVDTIA
>GCA_022096995
AVIKPVTMNTYAPVLMIAQSQVATVSGAGSPSLRAKTVYAKSGLQRKAETKWVSKDFYY-
GVVKMGNLVQLVSISVDNRAVCTVAQLVDQEVVVHGMVALEAVKKKIALEAIEGMTAEDPAKVVDY
-GEIELKKFRDADDRADMLPVMLGLTLKIKFDPHQPLVHDLAIPVVFEVV-
GVMVQQGTMEVDDCGVSTALSDVDPIVYVSPIDLAAVAGDAMAYIDQEAAVAAAKASIDLD-
LALESNVLVVSI-PLLIPV-FEAGGDT-MSFANRSRTLKTIVNLAVSYPAIKRA-----IAAAM-
MDASQAAALSEKKGTAEEIRIVKRMQRSAGLADDPDLVMLEDAAEGNSNWAGQIYDDPLSGSSESD
SAGNIVFAANKRQNFARSITYLRFVEISDKAAAPLMRGVKHNAKSRASRKLDDAIAVLNFANVPEGIAI
RAEAGKSQDLLLHDATEREEILLIVVRWDVTWKTAVIVSLFPTAGDVVGLIDVGGIGAVGRKEGLLNRH
HKLNGSLIEQRRITEKLTLRLRAALKQKLGKDQIIIFVVKVAGDLVAAATVQM---
AVGAVVAIQGPTIFVRRSRQTVIGDQEATVEILQGVAEGPADAEALLRIRVDHTVVKLSVLLIPQ-
PVLVESVTIREGVGKALQIIQLDVYLLRDMGTGEETLAKSKS--------------------------------
----
ISILKNLTEPRMDNLPAGVCRTFVNAGPRAAVGMLFLLLDDAGKIYTIDGDLRENTKSIKSLIGLVDAAK
PKILSAGYATRRLKIRYADVHVLISDADLYAYAEERRELLRMVPVYIINAAKRRQLILFALSLKGGHRRAR
AALGVMIAAAILESQAEQGAVNHEEPEVLLDDAEVVGSEMMISRQAALELKHVFKLDIIVIFPEFQEKM
VVSLKFFCTDVAIFVIALICTVGQMLSREPSHAQEGGAGFQK----HSVLFALSVTFERRNLRDA--
AGLFLALGLAPPRYGVFYTEQPKRKQMPSEADSEKVDGFDLVNAIKEDTTS-EELDMPEAMALAVE-
DASINVNAGVLALNGDMAAVEKVDSKRKMALAVEKWTISPVSLVDIKISGAQPFRALN-
IEQADVSASLPKKEV--
LFLPGANNAELEEEKLDGAGASTAEVAEDKQSAILLVNPVLVVIQDSNSAALIANDGLEEWGTMAYKK

HYALAARMRLHMRTID----GVAMDPNKQKAEKIKTVKLDGDTRFRQLATDGVKEQMP-
LHALNPDFAVLIPVLVTPAVGRVAADAGVLGARVPCDASL-
RELQKSQKGSALPISGIRVLGMRAARAMVDEDIMVAGKVAA-
LLVILALETITAIVLMNDSEATLPWRLSVAERLEADTQRSAGYASRPFNSKPSLCKTQAHAKIADPIQEIA
AGYVATEQRFVFPVEDIPIQLILASTHSRVGAAKRAGTGHLHEMAKELQAKAVIMVQNEPATNTDGLL
NYLTTQQPTDLLFEDCLDVVIDMRRMSVV--
VHLNTV--
VVVEGVTPVGSYEIMAAAAHSGLVQVERMDERTGRERTLMWMYGAMEELWLLSITYADWVINCAI
GAILLHVYAQQINRAFSLERLQLAGWPPEATRWWNTWQLWLNKDGFFSYEAAFRA-
KAVLKADLDDYDMSDVEGVGRYQSRQEIRVGYRIREQVAFAQLDPRTDILLQSMTRLSVIRHALKMV
AILIVREIVVEFTMVVAQEDVAMGHVECVLADSFDFTAKNADIGPVGLRTPDPVLGDGTVLKEAVLGKI
DLANTVADFRYKMLLDGGRELGVSWVTLQNMIYARIWVPPAQADLKDLQPICFFLQHPQLDL-----
----
ISKARANKLMAGALIEKILEKVAAFADPEATTHQSKKLRHFKYYGEEVDPVYLAYILDMFALVATQVTV
SDRLLKDRYVLNLKVFRGFTSLLWLSAQIGDKGPCATIITVQTVAGLGALVLFIILL-
RAVVLVETLYYKYVYVVMLGYQAAKDVADADPEHFLSLAHASRDVEWIEVIPAFFYYFKTAKKNLTKN
TALAKEFFGLFEERLKIIDVLVVPPDRVEALVEFVALAGTLATSAKALIGIIACGFCEPNAAAVLEADQDW
NLHRSMGITLAFYKAQVSVKKWFDKGLHKDVAAIKLTEAGALTTPFHAGSTAKQCVPAIHRVLDLGD
VEAALIEIDSAVVVADEEQTLADKPTTRENSVGAVGGVVLAGTMNMGANLDSVERGGNIIIELEEESY
NAVNGKKNDGKMTDKVLMVLSQAYFPVLKRMASANALDRGAFGLLIDAPNDADNQDHGRCLTIP
APTIHAESAHLFAESDIERIAWIAPIHLLGTLDAVLGFEPDQYSFISMMIEASLDIMETAEVMEDYHVLEK
GSRPPGFRVILIKGLHIRLLAGICTIIVVGSAGRSEITAYIQG-
AVPITLNKQTFLPAFAASSCHVIDILSTMLRPPERSTIKMLILETVAEL-
LEVTRIENDKLERMDVLLPGLDIALLLGLLAAAADFAEIVTIDAAITAILSVAAEAVVLREMLLTMAEIAVT
ALALAMALLLMLTRDQGLHQREAAREAQMKQLIQGGTVLQVKAADRMMLASTADKVRLATDEGTT
SAFASLNLATVSDGFYVMSSAVLQMSSDLMMIVAVARVLADKETKNALVVRHPEQVKTQSTYAEGS
PGMVLQTLTSAIPPLAIKPEDVELLWTLVAALWRVKSHLANAARPHIERVLILILALSLITVLLQSEGGGM
GGG-AGPAAKILAAFSILTI--
KAGEEVRKVTRRVMDLNSRLGRPEEAVNPEFRGAELEQTKYEAESVEVDTMETIRRFVDIPFLVSTRTQ
HNESEDRGARMVLDVTRVRFIDL-
VTKRSEIILAARAETDGIIAREMGKDLAALHPEAAALGKIAALQHFIAIQDAHISNYVTAEIHAVLGMLLL
VLFFQEVRVSKVVWPTRREVTMVFILAALAIFFLVIGL--
QIVFMLSGISWNQLTTIVLGEGIMVVRQTSTFPADS-
IGILQNTYADLICAGRVAKADIEKSLHIVVAGRVARGTWYVIYLYDISQAQGQSGIMQRAFRQMTLSQ
SLIDGRMEVKIKVVTSDRYA-
TYEIKGGQHRKGTRYNRVKVGHNIPVQVLPRMKMAFAPPNLVLVKTFGAVVQKRFAQALTHMRHG
AVTIGPMEVSSPYISNKKARHAPGDSVVDLKPENIVRSVAVVPELIWARENEDNGAVHDKYVKTTHKL
AAKRKKAVDKAQYDHRNQVELSFIGLAVKVLPDKA---------------------------------------
-----
RMRPQLNLGGQTDKKIVKCLNNNLDDVEYGKLGRRKIKPITIKSVYDVEHKRATELVKLMPYKDDVES
RKSQFAEK--
ALFEPLKAFMMLFGFIARSVYALYLRVIRRTILLVKTKIVVNAATVLSAIKIIGVSIRLRHKSTNVAETNHHI
KTIEARARMTLAGTGKVKLNLHEGKIGMKGGRIKGFMPYKNFNIFDGLASGRRAKVAELAKLKDVDKTI
SVDGVRVKQQGGRTPKAQLSNAI-

NTRVGVDIVGEDEQDASNPRANAEMGQIFAKIDARVFSHVGALIGHALEA-
LAVVAIWAIGRAHNLLAVRLKINAGIEMHSAA-VAAAVAAA-FAQAAGEA-
AIADVSEGDLGALALSAMSVDVCRLRRRSLSE---
RHGIRAKGTYGKERAMMQITPTKPVKIWVPVFVGEDELLAPIRAFAETQGDGVTVVKLTTDVKVTMFE
KAVASLAVTGKATSIVYTVLEKVTQLMKDIIYTQRAKAHYGRGRLRSHVVTTTKQRRHLSMMKRTVIA
DPSMKAARLTDGEVTVITGATMAFVMSEQDAV--
GDVDMHFHEEPSREAEVGIGGPETFLTTDQPRVLALLFLRAYLIHIVEIETTGGFADAATIYDVLRAMQ
NKLDRKLSADIAKTRMVLTDNVTDMGVTDVAPRRNAAGDKILSAFWINGVTANADIILLVANLECPC
VPENIDCAIRLFYQRITLQAKERGGDVSVAVVPAANQIEAAEGTVIYSIKGQTPGTGQMKLTIEGISRSV
VKSLAVQLFQVDTFLNDTWIATWLRAAKDVVFVASEDGKEAVDHVVERDTELEFAPQKDIVARKIRIF
TPAAPSAVVIWDPAIDREDKAEAQGTILKIYGPNLQDINCFTDIGAEPKIESLKAWDEKEKYVVHRDDIP
EDIDTLVGKSSKVNKEFGIPDVAVCAPSKPGVTESIDRGEPKAGTCGEVANMAYPQESLVNAVSSANV
QKNISMVLTGHPVGEMGISREIERHLETNMCEIFVDKADVDVALDFHILALTPLRVGLDQPNARVQID
GKESRKLMAIQDGEAALQRHPKISAATGTG-KAGTKARLPDDALILDGVMHGDLSFTATDLPEGRV-
VTPVFNDVKAQLEIDKVILRGFHVVTLSFSNEKGEEHLIKLRLNALKKSTRHYEMLLKIRETECAESPHAIS
VGKVLKIDKLLQTICQAHYKAIEPLLADDLTALGVMGQQGLLHVVPRLMILAVMHMARLEIALQIAG
WRLQAQAILTDQTMGLSIRARDWSY-AISEVVGNTFFARTKEDDTEQRIHARNPV-VLL-LGKFQMI-
VSPSGLTLASTEPSPQDRGNAEELILSTERLQDEMGYYVLQILLVFVDMVILAYGSGDDPIKFLVAEQA
VTGKQNPGMVTLTR-
TFPLELQRNATFVLVLEDCTMKNLSHKKMHMNLIIIKSKGWVVNVSTKILISDQAIASYAEDAGAHTQ
PSESALAAITSRVMAPIVLVVSQKPTAPSKTLFLEALSLYDEVTATVLNKPKKYYIFEGDVVAVLGTLDAA
VLLGMQRPQPNRMRRAVVAQAIRLAIAIMASGVITRLEEGRINFARSVSLAVGVYPTLDMLPAAIIH-
TALEFRVRNHDVSTKRSRPYKAHTKFLHKADVDKSLIATGVDTLA
>GCA_023015625
AVIKPVVMDTYAPVLMIRQQQVATVSGAGSPTLRAKTVYAKSGLQRKVETKWVSKDFYY-
GVVKLGNIVQLVSISVDKRSVCTVAQLVDQEVVVHGMVALEAVKKKIALEAIDGMTAEDPAKVVDYA
SEIELRKFRDTDDRADMLPVMLGLTLKIKFDPHQPVVHDLPIPVVFEVV-
GIMVQRGQIEVDECGISTALSDVDPIVYVSPIDLAAVAGDQMAYIDQEAAVTAAKAAIELEELALESAV
LVVGI-PLLIPV-FAADGDV-MSFANRSRTLKTIVNLAVSYPEIKRA-LDN-IAAAM-
MDASQAAALSEKKGTAEEIRIVKRMQRSSGLADDPDLVMMEDAAEGNSNWAGQIYDDPLSGSSES
DSAGNIVFAANKRQNFARSITYLRAVEISDQAAAPLMRGVKHNSKSRSSRKVNDAIAVLNFPNVPEGI
AIRAAAGKTRDILLHDATEGEEILLIIVRWHVTWKTAIIISLFPTANDIVGIIEMGGIGAVGRKDGLLNRHH
KLNGSLIEQRRIVEKLTLRDRAALKQKIGKEQIIIFVVKVAGDLVAKAKVQM---
SVGTVVAIQGETIFVRRHRQTVIADQEATV-VLRGVAEGPADAQALLRIRVDHTVVKLSVLLIPD-
PVLTESVTIREGVGKALQIIQLDVYLLRDMGTGDETLAKSKSREVIREVLQVDDITGVRLAFGQLKERLV
KGGTMKELGTL---
IAILKKITEPRMANLPDGVCRTFINAGPRAAVGMIFLLLDDAGKIYTIDGDLRENTKSIKSMIGLVDAAKP
KTLSAGYATARLKIRYADVTVLTSDADLYAYAEERNELLRMVPVYITNQAKRRQLIIFALSLKGGHRRAR
AALGVMIAAAILESQAEQGPVNHEEPGVLLDDGEIVGSEIMISRQAALDVKQIIKTDIIVIFPEFQEKMV
VSLKFFCTDVAIFVIALICTVGEMLSREPSHAQEEGAGFQKRIRGHSVLFALAVTFERRNLRDA--
ADLFLNLGLAPPRYGVFYTEQPKRKQMPSEEDKKKVDGFDLVAAIKSDATK-EELDMPEAMALAVE-
NETINVNAGVLAMNGDMAAVEKVDSKRKMAVAVEKWTIARVALTDLKISGAQPFRALS-
IEQADVSATLPSKQVWFLFLPGANNAEMEEEKLDGAGSSTAEAAEDKQLGILLVEPVLVVIQDSNTAS
LIANGGLEEWGTMAYKKHYAFAARMRLHMRTID------

EMDPNKQKAEKIKTVKLEGDTRFRQLATEGVKEQMP-
LHALNPDFAVLVPVLVTPAVGRVAADAGVLGARVPCDASL-
RELQKSMKGNALPISGIRLLGMRAARAMVDEDIMVAGKVSA-
LLVIVALETITHLVLMNDSEATLPWRLSVAERLEADTKRAGGFAARPFNSKPSLCKTQAHHKIADPIQEI
AAGYVATEERFVFPVEGIPIQLILASPHSRVGEAKRAGLGHLHEMAKELMGKAVIMVLNEPGTNTEGLL
NYLTTQQPTDLLFEDCLDVVIDMRRMSVV---
VQLNTV-
HCMIPGTRDLAEREIMAAAAHSGLVQIERVDERTGRERTLMYMYGAMEELWLRSLTWGDWVVDCA
IGAILLHVYAQQINRAFSLERLQLAGWPPEASRWWNTWQLWLNKDGFFGYEAAFRAVKAVLKADLD
DFEMSDVEGVGRYQSRQQLRVGYRIRQQVAFEQLDPRTDILLQSMTRLSVIRHALTMVAILIVREVVV
EFT-
VVAKEDVAMGHVECVLADSFDFTAKDADIGPVGIRTPDPVLGDGTVMKEAVLGKIDLANTVADFRYK
MLIDGGRDLGVSWVTLQNLLYARIWVPPAQAALKNLVPIAFFLGHTELNLAIFPELWAGISKARANKL
MAGALIEKILEKVAAFADPEATTHQSKKLRHFKYYGEEVDPVYLAYILELFSLVATQVTVSDRLLKDRYV
LNLKIFRGFTSLLWLSAQIGAKGPAATIITVQTIAGLGALVLFIILLLRVVVLVETLYFKYVY--
MLGYQAAKDVADADPEHFGSLAHASRDVEWIEVVPAFFYYFKTATSRLSKNTALAKEFFGLFKERLRII
DVLVVPPDRVEALVAFVALAGTLATSAQALVGVIACEFCEPNAAAVLEADQDWNLHRSMGITLAFYD
ARESVKGWFDKGLHKDVAATGLREAGALTTPFHAGENAKQCVPAIHRVLDLGEVEAALIEIDSAVVV
ADEEQSLADKPTTRENSVGAVGGVVLAGTMNMGANLDSVERGGNIIIELEEEQYSAVNGKKADGKV
TDKVVMVLSAAYFPVLKRIASANALDKGAFGLLIDAPDDADNKDHGRDLTIPAPTVHAESAPLYAESEI
ERIAWIAPIHLLGTLDAVLGFEPDQYSFISMMIDASLEIMETAEVLDDYHVLETGSRPPGFRIILIKGLHIRL
LAGVCTIIVVGSAGRTEITAYIQG-
AVPITLNKKTFLPAFAASSCHVIDIFSTMLRPPERSAIQMLILETVTEL-
LEVTRIENDKLERMLALLPGLDVAALLSLVAAAKDFASIVTIDQAITAILSVAAEAVVLREMLLTLAEIAVT
ALALALALLLMLTPDQGLHQKERAREAQMKALIQGGVVLQVKAADRMMLASTADKVRLDTDEGTTS
AFASLNLATVSDGFYVLSSAVLQMSADLMMIVAVARVLADKETKNALVVRHPEQVKTQSTYAEGSP
GMVLQTLTAAIPPLAIKPETVELLWTLVTALWKVKAHLANAARPHIERVLILILALSLITVLLQSEGGGM
GGG--
GPAAKALAAFSILTIKAKAGDEVRKVARRVMDLNSRLGRPEEAVNPEFRGAELEQSKYEAENVEVDTM
ETIRRFVDIPFLVSTRTQHNESEDRGARMVLDVTRVRFIDL-
VTKRSEIILAARAETDGIIAREMGKDLAALHPEAAALGKIAALQHFIAVQDAHISNYVTATIHAVLGMLL
LVLFIQEVRISKVVWPTRREVTMVFIMAALAIFFLVIGLGVQLVFMLSGISWNQLTTIVLGEGIMVVRQT
NTFPEDS-
IGILQNTYADLICSGRVAKADIENSLHIVVAGRVARGTWYVIYLYEISQAQDQSGIMQRAFRQMTLSQ
SLIDGRMECKIKVVTSDRYA-
TYEIKGGQHRKGTRYNRVKVGHNIPVQVLPRMKMAFAPPNLVLVKTFGAVVQKRFAQALTHIRHGA
VTIGPMEVSSPYITNKKARHAPGDAVVDLKPENIVRSVAVVPELIWARENEDNGAVHDKYVKTTHKL
QAKRKAAVDKAQYDHRNQVELSFIGLAVKVLPDKAIVDATAGTLMPTETVTVTEGLKGVAATIRLDP
MEKTLIAYIKRMRPQLNLGGQTDKKIVKCLNNNLDDVEYGKLGRRKIKPITIKSVYDVEHRRATELVKL
MPYKDDVEGRKSQFAEK--
ALFEPLKAFMMLFGYIARAVYALYLRVIRRTILLVKTKIVVNASSVLSAIKIIGVSIRLRHKSTNVCETKHHI
KTIEKRARMTLAGTGKVKLNLSPGKVGKKGGRIKGYMPYKNFNIFDGLASGRRAKVKELAKLKNVDKAI
SVDGIRTKQQGGRTPKAQLSNAI-
NTRVGVDIVGENEQEESNPRANAEMGQIFAKIDARVFSHVGALIGHALEAVLAVVAIWDIGRAHNLL

AIRLKINAGIEMHSAA-
VKNAVAAAAFAQSAGEASALWDISEAYLGTLAISDMSVDVCRLRRRSLNELKKTKKDAYMSLTCFGG
KGDKVKIVEATQTQAYEIVATRTAERHGIRAKGTYGKERAMMQITPTKPVKIWVPVFVNETELLAPIRA
FAESTGDGVTVVKLTTDVKVTMFEKAVASIAVTGKATSIVYTVLEKVTELMKDIIYTQRAKAHYGRGRLR
SHVVTTTKQRRHLSMIKRTVVADPSMDAARLTDGEVYVITGATMAFVMSEQEAV--
GDVDLHFHEEPQREADLGIAGPETFLATDQPRVLLFIHLKAYLIHIVEIGTVNGFSDAATIYDVLRAMQ
NKLDRKLSADIAKTRMCLTDNVSDMGVTEAAAKRDAAGDKILSAFWINGVTANADIILLVANLETHC
VPENIDCAIRLFYQRITLQAKERGGDVSVAIVPAANQIEAAEGTVIYSIKGQTPGTGQMKLTIEGISRSIV
KTLAVQLFQVDTFLDDTWIATWLRAALQVVFVGAEDGKEAVDHVVERDTELEFAPQKDIVARKIRIFT
PAAPSAVVIWDPAIEREDKAEAQGTILKIYGPNLQDINCFTDIGAEPKIESLKAWDEKEKYVVHRDDIPE
DI-
TLVGKSSKVNKEFAIPDVAVCAPSKPGVTESIDRGEPKAGTCGEVANMSYPQESLVNAVSSANVQKNI
SMVLSGHPVGEMGISREIERHLETSMCEIFVDKDDVDVALDFYILALTPLRVGLDPPNARVQIDGKESR
KLMAIQDGDADLQRHPKVSQSTGTGLKAGVKARMPDDALMVDGVMHGDVAFTATDLPDGRV-
VTPVFNEVKAQLKIDKVLLRAFHVVTLSFSNDKGEEHLVKLRLNALKKSTRHYEMLLKIRETECAESPHA
ISVGKVLKIDALMQTICQAHYKEIEPLLADELTALGVMGEQGLLHIVPRLMILSVLQLLHLEIALQLHRW
GLQAAAMVTDQTMGLYIRDRDWSF-AISEVVGNTFFARTKESDTEQKIRARNPV-VLL-LGKFKMI-
ISPSGLGLASTAESSQGRGNTEELILSAERLQDEMGYYVLQILLVFVDEVILAYGSGEGPIKFLVAEQAVT
GRQNPGMVTLTR-
TFPLELQRNATFVMVFEDCTMKNLSHKKMHMNLVVIKSKGWIVNVSTKILISDQAIASYAEDAGAHT
QPSESALAVITSRVMAPIVLVVSQKPTAPSKTLFLEALSLYDEVTSTVQNKPKKYYIFEGDVVAVLSTLD
AAVLLGMQRPQPDRMRRAIVADAIRLAIALRASGIVTRLKEGIINFSRAVNFAARFYPVETA---------
--EFRVRNHDVSTKRSRPYKAHPKFLHKADVDKSLIATGVDTLA
>GCA_023015725
AVIKPVTMNTYAPVLMIAQSQVATVSGAGSPALRAKTVYAKSGLQRKTESKSVALPIYY-
GVVKMSNIVQLVSISVDSRAVCTVAQLVEQEVVVHGMVALDAVKKKIALEAIDGMTAEDPAKVMDY
--EIEIRKFRDVDDRADLLPVMLGLTLKIKFDPHEPVVHDLAIPVVFEVV-
GIMVQRGQIEVDECGVSTAISDVDPIVYVSPIDLNAVAGDKMAYIDQEGAVSAAKDDIALEELALESA
VLVVGI-PLLIPV-FEADGDV-MSFANRSRELKTIVNLAVGYPEVKRAR----IASAM-
MEQSQAAALAEKKGTAEEIRIVKRMQRSAGLADDADLVILDDSAEGNSSWAGQIYDDPLSGSSESDS
VGNIVFAANKRQNFARSITELRKVEISDKAAAPLMRGVKHNAKSRSSRKLDDAIAVLNFPNVPEGIAIR
AEQEKPKDTLLHEAAEGEEILLLIVRWHVTWKTAIIIYLMNDAGDIKDMITLGGVGAVGRKEGILNRHH
KMNGSLIEQKRVIEKLTLRLRAALKQRLGKDQVIIFVVKVAGDLVAAAQIQM---
NVGAVVAVQGPTIFVRRSRQTVIGDQEATVEVLKGVAEGPADAEALLRIRVDHTVVKLSVLLIPDVPVL
VESVTIREGVGKALQIIQLDVYLLRDMGTGEETLAKSKSKEQIRSVLQVDPITGVRLAYGKLRERLVKGG
SQKELGTL---
LSILNNITEPRMANLAEGVCRTFIEAGPRAAVGMIFLVLDNAGKIYSIDGDLQENTKSIKSMIGLVDAAK
PKILSAGYLTGRLKIRYADVTVLISEADLYAYAEVRRELMRMVPVYITNAAKRRQLILFALSLRGGHRRA
RMSLNVMLAAAILESQAEQGAVNHADPDILLDDGEVVGSEMMISRQASLDLKAVLKIDVIVIFPEFQE
KMVVSLKFFCTDVAIFVIALICTVGDMLSREPKHAQEGGAGFQKRIRAHSVLFALATTFDRANCR--
DVPDLFAGLGLADPRYGILYTEQPKRKQMPSEPDKDRVDGFELVHAIKADATDNHGLEMPEAMALA
VEATESINVSAGVLAMKGDMTAVEKVDSKRKMAHAVEKWTIAKVPIIDVHISGAAPFRCLG-
IEQADVSAALPRKELDALYLPGANNAELEEEKLDGAGSSTIEAAADKQAAILLVNPVLVVIQDSATASLI
SNGGLEEWGTMAYKKHYAFAARMRLHMRTID------

GMAPNKQKAEKIKTVKLEGDTRYRQLATEGVKEQMP-
IHALNPDFAVLIPVLVTPAVGRVAADADVLGAEVPCDYSL-
RELQKSQKGNALPISGIRLLGMRAARAMVEEDIMVAGKVAA-
LLVIGGLETICKLVLMNDSEATLPWRLSVAERIAADTKRAGGYAARPFNSKPSLCKTQAHAKIADPIQEI
AAGYIAVEERFVFPVEGIPIQLILASPHSRVGGAKRAGLGHLHEMAKELIGKAVVMVLNEPGTNTEGLL
NYLTTGQPTDLLFEDCLDVVIDMRRMSVV---
VQLNTV-
HVVVAGVRDVGSYEIMDASADSGLVQVERVDERTGRENTLMYMYGAMEELWLNSIRYADWVIDCK
IGAILLHVYAQAINRAFSLERLQLAGWPKEASSWWNTWQLWLNKDGFFSYEAAFRALKAVLKADLYD
FELKDVEGVGRMLSRQEVRAGYRIREQITYEQLTPRTDILLQSATRFSIIRHALTMVAILIVREVVVEFT-
VVAKEDVAMGHVECVLADSFDFTAKNADIGPVGLKTPDPVLGDGTVLKEAVLGKIDLANTVADFRYK
MLIDGGRALGVQWISLQNMLYERIWVPPAPAALKNLQPIKFFLGHPEVDL---------
ISKARAEKLMATALIEKILAGVARFAKPEATTHQSKKLRHFKYYGEEVDPVFLAYILAMFALVSTQVTVS
DRLLKDRYVLNLAV-RGFTSLLWLSAQLGEKSPAATIISLDSIAGLGALVLFIILL-RASVLVETLYYKYVY-
-
MLGYKAAKDVADADPEHFLVLAAASRDVEWIEVIPSFFYYFKTATSRLTKNTALAREFFGLFAERLRVID
VLVVPPDRVEALVDFVALAGTLPTSAEA-
TGVIACGFCEPNAAQVLEADQDWNLHRSLGITLAFFEARESVKGYFDKGLHKAVAEIGLSEAGALTTP
FHAAVTAKRCVPAIHRVIDLGEMEAALIEIDSAVLVADEEQTLADQPTTRDNSVGAVGGVVLAGTMN
MGANLDSVERGGNIIIELEEEGYNAVNGKKADGKLTDKVVMVLSRAYFPVLERAASANALDKGAFDL
LIDAPDDTENLDHGRTLTIPAPSIHAESAILFAESDIERIAWIAPIHLLGTLDAVLGFEPDQYSFISMMINA
SLDIMETAEVLDDYHVLEKGSRPPGFRVVLIKGLHVRLLAGIGTIIVVGSAGRSEVTAYIQG-
AVPITLNRVTFLPAFAASSCYVVDIFSTMLRPPERSAIKMLILETVTEL-
LEVTRVENDKLERMDALLPGLDVAALLPLVAAAKDFAKIVTIDRAIAAILSVAAEAVVLREMLLTLAEIAV
TALALALALLLMLTRDQGLHQKESAREAQMKALIQGGVVLQIKAADKMMLASTADKVRLDTDEGTT
SAFASLNLATVSDGFYVLSSAVLQMSSDLMMIVAVARVLADKETKNALVVRHPEQVKTQTTYAEGSP
GMVLQTLTGAVPPLAIKSEEVELLWTLVAALWRVKAHLANAARPHVERILILILALSLIAVLMQSEG-
GSMGGGSGAPVKIIAAFSILTVKAKAGDEVRKVARRVMDLNSRLGRPEEAVSPEFRGAELEQVKYEAE
NVAVDTMETIRRFVDIPFLVSTRTQHNESEDRGSRMVLDVTRVRFIDL-
VTKRSEIILAARAETDGVIAREMKSDLAALHPEAAALGKIAFLQHFIAVQDGHISNYVTAQTNSVLGML
LLVLFINETRVAKVVWPTRREVTMVFIMATLAIFFLVIGLGVQLVFMLSGISYNQITTIVLGEGVMAVRQ
TSTFPADS-
IGILQNTYADLICSGRVAKADIEKSLHIVVAGRVSRGTWYVIYHYDITQAQGPSGIMQRAFRQATLSQS
LIDGRMEVKIKVVTSDRYA-
TYEIKGGQHRKGTRYNRVKIGHNIAVDVLPRMKMCFTPPNLVLVKTFGAVVQKRFAQALTHTRHGA
VTIGPMEVSSPYIKNKKARHAPGDAVVDLKPDNILRSVAVVPELIWARENEDNGAVHDKYVKTTHKL
AAKRKKAVDKAQYDHRNQVELSFIGLAVKVLPDKAIVHATSGTLMPTASVAVTEEMKGIAATIRLDPM
EKSLIAYIKRMKPQLNLGGKTDKKVVKCLNNNLDDVEYGKLGRRKIKPITIKSVYDVESERATELVKLMP
YKDDVEGRKSQFAEK--
ALFEPLKAFMMLFGYIARAIYALYLRVIRRTILLVKTKIVVNANTVLSAVKIIGVSIRLRHKSTNVADTNHH
IKTIEKRAKMTIAGAGKVKLNLSPGKIGKKGGRIKGFMPYNNNYNIFDGLASGRRAKVKELAKLKSVDKTI
SVDGIRTKQQGGRTPKADLSNAIANTRVGVDIVGEEVQEESNPRANAEMGQIFAKIDARVYHHVGAL
IGHALMAVLAVVAIWGIGRAHNLLADAFRINAGIEMHSAA-VEAAVAAA-
FAQPGGEASVIADVSEAYLGALLISEMSVDVCRLRRRSLGELKKTKKDAYMSLTCFGGKGDKVKIIEAT

QTQAYEIVATRTAERHGIRAKGSFGKERAMMQITPTKPVKIWVPVFVSESELLAPIRAFAETQGDGVTV
VKLTTDVKVTMFEKAVASLAVTGKATTLIYTVLEKVTQLMKDIIYTQRAKAHYGRGRLRSHVVTHTKQK
RHLSMMKRTVVADPSMNAARLTDGEVSVVTGATMAFVMSEQDAV-
PGDVDFHFHEEPRREADVGIGGPETFLCTEQPRVLALLFLRAYLLHIVEIETSNDFSDAATIYDILRAMV
NKLDRKLSADIAKTRMCLTDNVMDMGVTDVAARRNAAGDKILSAFWINGVTANADIILLVANLECP
CVPENIDCAVRLFYQRITLQAKERGGDVTVQVVPAANQIEAAEGTVIYSIKGTAPGTGQMKLTIEGISR
SIVKSLAVQLFQVDTFLDDTWIGTLLRAALDVVFVGAEDSKEAVDHVIERDTELEFAPQKDIVSRKIRIFT
PAAPSAVVIWDPAIDREDKADAQGTILKIYGPNLQDINCFTDIGAEPKIESLKAWDEKEKYVVHRDDIP
EDI-
TLVGKSSKVNKEFAIPDVAVCAPSKPGVTESIDRGEPKAGTCGEVANMSYPQESLVNAVSSANVQKNI
SMVLAGHPVGEMGISREIERHLETNMCEIFVDKDDVDVALDFSVIALTALRVGLDTPNARVQIDGKES
RKLMAVLDGEANLQRHPKVSAATGTGLKAGTKARMDDDALIVDGVVHGDIPFTATDLPAGRV-
VTPVVNNVKAQLEIDKVILRTFHVVTLSFSNDKGEDHLIKLRLNALKKSTRHYEMLLKIRETECAESPHAI
SVGKVLKIDKLLQTICEAHYKAIEPLMADDLTALGVMGEQGLLHIVPRLMILAVLLAATIECEFLIHSWGL
QADAIVTDQTMGLPTRTREWSF-AISEVVSNTFFARTADDDTEQRIHARNPV-VLL-YGKFGMV-
ISPSGLRLKSTAQTEQERGNKEDLILSTERLQDEMGYYVLRILLVFVDMVILAYGSGDEPIKFLVANQAV
TGRQNPGMVSLTRRTFPLELQRDATFVMVFDDSTMKNLSHRKMHMNLVLVKSKGWVVNVSTKILIS
DQAIASYAEDAGAHTQPSESALAIITSRVMAPIVLVVSQKPTAPSKTLFLEALALYDDVTATVLNKPKKY
YIFEGDVVSVLNTLDAAVLIGMQRPQPDRMRRAIVATAIRLAIALRAAGVVVRLQNGLINFSRAVTLA
VRVYAQITMI----------AFRVRNHDVSTKRSRPYKAHPKFLHKAEIDKSLIAEGVDTIA
>GCA_2517487002
AVIKPVTMKEYAPVLMIAQSQVATVSGAGSPSLRAKTVYAKSGLQRKSETKWVAKDFYY-
GVVKMANLVQLVSISVDKRSVCTVAQLVDQEVVVRGMVALEAVKKKIALEAIDGMTAEDPAKVVDY
AGEIELRKFRDADDRADMLPVLLGLTLKIKFDPHQPVVHDLPIPVVFEVV-
GIMVQRGQIEVDECAISTAVSDVDPIEYVSPIDLAAVAGDAMAYIDQESAVATAREEIGLEELALESAVL
VVGI-PLLIPV-FAEEGDT-MSFANRSRTLRTIVNLAVSYPEIKRA-LDS-IAAAM-
MEQSQAAALAEKKGTAEEIRIVKRMQRSSGLADDPDLVMLEDAAEGNSNWAGQIYDDPISGSSESD
SAGNIVYAANKRQNFKRSITYLRSVEISDQAAAPLMRGVKHNAKSRASRKLDDSIAALNFPNVPEGIAI
RAEAGKTRDILLHDATEGEEILLIIVRWHVTWKTAIIIGLAFLAGDVVELIAMGGIGGVGRKEGVLNRHH
KLNGSLIEQRRVVEKLTLRLRAALKQKLGAEQIIILVVKVAGDLVATAKIQM---
SVGTVVAIQGEVNVKRRSRQTVIGDQEATV-VLQGVAEGPADAQALLRIRVDHTVVKLSVLLIPE----
----
REQFREVLQVDDITGVRLAFGHLRERLVKGGTMKELGTLL--
IAIIQGITEPRMANLPEGVCRTFIRAGPRAAVGMLFLLLDDA--------------------------------
----
IRYADVTVLTSDADLYAYAEERREVLRMVPVYIVTQAKRRQLIIFALSLRGGHRRARAALGVLIAAAILES
QADQGGVNHEAPEVLLDDAEVVGSEMMISRQAALDLKQIIKTDIIVVFPEFQEKM----------
MAIFVIALICTVGSMLSRDPSHAQEGGAGFQKRIRGHSVLFALAVTFDRRNLRGADGAQLFLGLGLAP
PRYGIFYTDQPKRKQMPTEPDKDKVDGFDLVNAIKEDTASNHSLEMPEAMALAVE-
DGTINVNAGVLALTGDMAAVEKIDSKRKMALAVEKWNISKLDLVDLSISGAQPFRALS-
IEQADVSATLPHKAI--
LYLPGANNAEMEEEKLDGAGASTAAAAEDKQKGILLVVPVLVVIQDSNTAGLIANGGLEEWGTMAY
KKHYALAARMRLHMRTID------AMEPNKQKAEKIKTVKLEGDTRFRQLATEGVKEQMP-
LHALNPDFAVLVPVLVTPAVGRVAADQDVLGARVPCDASL-

RELQKSMKGDALPISGIRLLGMRAARAMVDEDIMVAGKIAA-
LLVIVALETITALVLRNDSEATLPWRLSVAERLEADTKRASGYAARPFKSKPSLCKTQAHARIADPIQEIA
AGYVATEERFVFPVEGIAIQLILASPHSRVGSAKRAGLGHLHEMAKELQAKAVIMVLNEPGTNTEGLLN
YMTTQQPTDLLFEDCLDVVIDMRRMSVV--
VQLTTI-
HVVVSGTRDVAAYEIMAAAAHSGLVQVERVDDRTGRERTLMWMYGAMEELWLMSVTWADWVT
DCAIGTIILHVYAQAINRAFSLTRLQLEGWPKEADRWWNTWQLWLNKDGFFGYEAAFRAVAAVLKS
ELYDYEMSDTEGVGRYQSRQELRVGYRIRQQVAYERLDPRTDILLQSLTRLSVLRHALNMVAIMIVRE
VVVEFTLVVAQEDVAMGHVECVLADAFDFTAKNADIGPVGLRTPDSVLGDGTVLNEAVLGKIDLANT
VADFRYKMLIDGGRRLGVTWISVQNMLYARIWLPPAQAALKNLVPIAFFLGHTELNLAIFPALWAGIS
KARANKLMAGALIERILENVAAFADPEATTHQTKKARHFKYYGEEVDPVYLTYILDMFALVATQVTVS
DRLLKDHYVLNLKIFRGFTALLWLAAQIGEKGPAATIITVQTIAGLGALVLFIILLMRALVLVETLYYRYVY
--MLGYKAAKDVADADPEHFLTLAHASRDVEWIEVIPAFFYYFDTAG-
FEPFNTAVAKEFFGLFTERLRIVDVLLVPPDRVEALVEFVALAGTLATSAQALVGVIACGFC---------
----
KDVAETGLREADALTTPFHAGQTAKQCVPAVHRVLDLGELEAALIEIDSAVVVAGEEQTLADKPTTRE
NSVGAVGGVVLAGTMNMGANLESVERGGNIIIELEEENYGAVNGKKADGKLTDKVLMVLSEAYFPVL
KRMASANALDRGAFGLLIDAPDDADNRDHGRRLTIPAPTVHAESAPLFAESEIERIAWIAPLHLLGTLD
AVLGFEPDQYSFISMMIEASLDIMETAEVL-
DYHVLETGSRPPGFRIILIKGLHIRLLAGIATIIVVGSAGRSEITAYIQ-
GAVPITLNKKTFLPAFAASSCAVIDIFSTMLRPPERSTIEMLILETVAEL-
LEVTRIENDKLERLPILLPGLDVAAMLALLAAAQDFANI---
DRAITAILSVAAEAVVLREMLLALAEIAVTALALAMALLLMLTPDQGLHQKELTREAQMKDLIQGGAV
LQVKAADRMVLAATADKVRLDTDEGTTSAFASLNLATVSDGFYVLSSAVLQMSSDLMMIISVARVLA
DKETKNALVVRHPEQVKTQTTYAEGSPGMVLQTLTGAVPPLAIKPGEVELLWTLVAALWRVKAHLAN
ATRPHNERVLILILALSLITVLLQSEGGGMGGG--
GPAAKALAAFSILTIKAKAGDEVRKVTRRVMDLNSRLGRPEESVNPEFRGAELEQSKYEAESVEVDTME
TIRRFVDIPFLVSIRTQHNESEDRGARMVLDVTRVRFIDL-
VTKRSEIILAARAETDGIIAREMKGDLAALHPEVAALGKIAALQHFIAVQDAHISNYVTAEIHAVLGMLL
LVLFIQEVRISKVVWPTRREVTMVFIMAAVALFFLVIGL--
QVVFMLSGISWNQLTTIVLGEGIMVVRQTSTFPQDS-
IGILQNTYADLICAGRVAKADIEGSLHIVVAGRVARGTWYVIYLYEITQAQGQSGIMQRAFRQMTLSQ
SLIDGRMEVKIKVVTSDRYA-
TYEIKGGQHRKGTRYNRVKVGHNIPVQVLPRMKMAFHPPNLVLVKTFGAVVQKRFAQPLTHMRHG
AVTIGPMEVSSPYITNKKARHAPGDSVVDLKPDNIVRSVAVVPELIWARENEDQGAVHDKYVKTTHK
LAAKRKKAVDKAQYDHRNQVELSFIGLAVKVLPEKAIVHATAGMLMPTASVAVTDGMKGIAATIRLD
PMEKTLIAYIKRMRPQLNLAGKNDKKIVKCLNNNLDDVEYGKLGRRKIKPLTIKSVYDVEHRRATELVKL
LPYKDDVESRKSQFAEK--
ALFEPLSAFMMMFGFIARAVYALSLRVIRRTILLVKTKIVVNASTVLSAIKIIGVSIRLRHKSTNVAETNHHI
KTIEARARMTLAGTGKVKLHLREGKVGKKGGRIKGYMPYKNYNIFDGLASGRRAKVKELAKLKKVDKEI
SVDGIRQKQQGGRTPKADLSNAIANTRVGVDIVGENEQEESNPRANAEMGQIFAQIDARVYKHVGA
LIGHALEA---------IGRAHNLLAVKVKINAGIAMHSAA-VEAAVAAA-FAQAGGEA-ALADVSEG-
LGALLLTAMSIDVCRLRRRGLAELKKTKKDAYMSLTCFSGKGDKVKLIEATQTQAYEIVATRTAERHGIR
AKGTYGKERAMMQILPTKPVKVWVPIFVSEDELLAPIRAFAETQGDGVTVVKLTTDIKVTMFEKAVASL

AVTGKATSVVYTVLEKVTELMKDIIYTQRAKAHYGRGRLRSHVVTTTKQRRHLSMMKRTVVASPSMD
AARLTDGEVSVVTGATMAFVMSEQEAV--
GDVDLHFHEEPRREAEVGIGGPETFLTTEQPRVLALLFLRAYLLHIVEIETVNKFSDAATIYDVLRAMVN
KLDRKLSADIAKTRMVLVDDLKDMGVTDVAPRREEAGDKILSAFWINGVTANADIILLVANLECPCVP
ENIKCAIRLFYQRITLQAKERDGDVSVAIVPAKNQIEAAEGTVIYSIKGKTPGTGQMKLTIEGISRSVVKSF
AVQLFQVDTFLDDTWIATWLRAADDVVFVAADDGKEAVDHVVERDTELEFAPQKDIVARKIRIYTPA
APSAVVIWDPAIEREEKAEAQGTILKIYGPNLQDINCFTDIGAEPKIESLKAWDEKEKYVVHRDDIPEDI-
TLVGKSSRVNKEFAIPDVAVCAPSKPGVTESIDRGEPKAGTCGEVANMSYPQESLVNAVSSANVQKNI
SMVLGGHPVGEMGVSRGVERHLETNMCEIFVDSPNVDIALDFYVLALTPLRVGLDPPNARVEIDGKE
SRKLMAVMDGEANLQRHPKISAAPGTG-KAGTKARMPDDALILDGVMHGDISFTITDLPL-RV-
VTPVYNTVKAQLKIDKVILKAFHVVTLSFSNNKGEEHLIKLRLNALKKSTRHYEMLLKIRETECAESPHAI
SVGKVLKIDK--
AVLHLARLEIALQLPHWGLQADSVITDQTMGLAVRVRHWSF-
AISEVVGNTFFARTKEDDTEQRIHARNPV-VLL-LGKFEMI-
ISPSGLGLKSTAESQQQRGNTDELILSPERLQDEMGYYILRILLVFVDEIILAYGSGEEPIKFLVADQAVTG
RQNPGMVTLTRRTFPLELQRDATFVMVFEDSTMKNLSHKKMHMNLVLIKSKGWLVNVSTKILISDQA
IASYAEDAGAHTQPSESALAVITSRVMAPIVLVVSQKPTAPSKTLFLEALSLYDAVKATVLNKPKKYYIFE
GDVVSVMGGLDAAVLLGMQRPQPDRMRRAIIAAAVRLAIALRASGIVTRLEEGLINFARSVSFAA-
VYAEL-------------AFRVRNHDVSTKRSKPYKAHTKFLHKADVDKGLIAAGVDTLA
>GCA_2521172554
AVIKPVTQDVYAPVLMIRQTEVATVSGAGSPSLRAKTVYAKSGLQRKTSSKWVAGPFYY-
GVVKMSNIVQLVSISVDSRAVCTVAQLVEQEVVVHGLVALDAVKKKIALEAIDGMTTEDPAKVAEFE-
EIDLKKFRDAEARADMLPVMLGLTLKIKFDPHEPVVHDLPIPVIFEVL-
GIMAERGGIEVDECAISHSVSDVDPLSYVGPIDLDAVAGTDMAFLDQEAAVSAARDVIAL--
LALEAGILVVGI-PLILAPIFGEDGDV-MSFANRSRQLESIVDMAVAAEQLTRARIEGA--SAM-
MTQAQAEALCEKRGTAEEIRIVKRMQRSSGLADNPELVIMEDSAEGNSSWAGQIYDDPVSGSSESDS
AGNIVHAANKRQNFARSITHLRKVEISDKSAAPLMRGVKHNSKSRSARKVYDAIAELNFANVPEGIAIR
AEAGKPQNTLLHDATEGEEILLLIVRWGVTWKTAIIIALKH-
AGDVVGLITLGGVGAIGRPDGLLNRHHKLNGSLIEQRPIIEKMTLKLRAALKEKLGADQIIIFVVKVEGDL
VADATVQMG-GTVGAVVAIQGEVIFVRRHKQTVITDQESTV-
ILRGVAEGPADAQALLRIRVTHTLVELSVLLIPE-
PVLVDSVTIREGVGKALQIIQLDVYLLRDMGTGTETLAESGSRELIREVLSVDPITGVRLAYGKLKERAGK
KTGLKDLHT----LSIL-
GITEPRTENLPEGICRSYIPAGPRAAVGMIFLLLEDAGKIYTIDGDLRELTKSIKSLIGLVDAKAPKVLSASY
ATERLKLRYADCSVLEGEADLYAYAEERRELLRMVPVYIINAAKRRQLIIFALSLKRGHRRTMASLDVLIA
AAILESQAEMGTVHHEEPAILLEDAEIVGSAIMISRQSALDLKARFKMDVIVVFPEFQEKMVVSLKFFCT
EVAIFVIALIVTVGEMLSREPEHAQEEGAGFQKRIRGHSVLFALNVTLDRRNTRDA-
TPDLFLALGLASPRYGIFYVNQPKKKQMPSEPDGDREDGFELVHAIKDDGGS-EELEMPEAMALAVE-
NEPINVSGGVLALKGGMAAVEKVESKRKMSLAAEKWNISKLSLLDVRISGAAAIRCAG-
IEQADLSAALAAKKV-
SLFLPGASNAELEEEKLAGAGASTADAAADKQARILVVLPVLVVIQDSNTASLIANGGVFEWGTMAYK
KHYAFAARMRLHMRTID------VMAPNKQKSEKIKTVKLEGDTRFRQLATDGVKEQMP-
LHALNPDFAVLVQILVTPAVGLVAADESVLGAEVPCSFSL-
RELQKSQKGSALPISGIRLLPMRTARAMIDEDIMVAGKVAA-

LLKISALETVIHIILRNDSEATVPWRLSVAERLEADTKRSSGYAARPFMSKPSLCDTQAHHNVADPIQEI
AAGYVASEDRFVFPVEGIAIQLILSSPHSRVGEEKRAGEGHLHGMAKELFARAVLMLKNEPETNTEGLL
NYLTTNQPTDLLFEDCLDVIIDMRRMSVV--
IQLNTI-
HVVVPGVRDVGNFEIMTAAAASGLVQEERVDDRTGRERTMMWMYGAMEELWLNSLKWAGWVV
NCAIGAIILHVYAQSINRAFSLKRLQLQGWPLEADRWWNTWQLWLNKDGFFGYEAAFRALKQVLKS
TLFGFDMTNIEGVGRMQSRQQIRVGYRIREQVDFARLDPRTDILLQAQTRLSVIRHALEVVAILILRDV
VGEFTLVVAMEDVAKGHVECVLADSFDFTAKDADIGPVGLRDPDSIFGDGTVLKEAVLGKIDLANTIA
DFRYLVITDGGRELGVKWVTLENMIYERIWLPPAQAALKNLQPICFFLSHPELDL---------
ISKARAERLMAGALVEEILEGVARFAEPEATTHQSNKERHFKYYGEEVDPVHLAYILETFALVATQVTVS
DRLLKDRYVLNLRVFRGFTSLLWLAVQLASKGPASTIITLGTIAGLGALVLFIILL-RDNVLVETLFYRYVY-
VMRGYTAAKDVADADPEHLLALAAASRDMEWIEIVPAFFYYFKTATKNLKKNTAVAREFFGLFEERL--
-
AVLAVPPDRVLELVELVALAGTLVTSAEALSGVIACGLCEPNAAGVLEADQDWNLHRTMGITLAFFKA
QQSVNGYFDKGLHKDVATTGLRDAGGFTTMNLCEVEAKQCCPAVHRVVDLGEVEAALLEINSAVLV
ADEEQTLADKPTTRDKAVGAVGGVVLAGTMNMGGALEQVDRSQNIVIELEEEQYNAVNGKKSDGK
LTDKVLMV-----
FPVLRRTSSGNALDQGVFDTLVEAPEDADNQDHARELVIPAPTIHTETTPLYAESDIDRIAWIAPMHM
LGTLPAVLGFEPDHYSFISMMIEASLDITETAEVLDDYNVLEKGARPPGFRKILIKGLHIRLLAAVGTILVV
GSAGRSEITAYIQDRAVPISLSRKTFLPAFAASSCEVVDIMSTLLRPPERSAVEMLILETVTEL-
LEVTRLENDKLERLLILLPGLDTSELLNLVAAAKDFSAIVTIDAAITAILSVAAEAVVLRELLLTKAEIAVTAL
AFAIALLLMLTADQGLTLKDHARERQMKDLIQGGAVLQVHARDAMVLAATADKVRLETDEGTTSAF
ASLNLATVSDGFYVLSSAVLQMSADLMMIVAVARVLADKETKNALVVRHPEQVKTQPTYAEGSPGM
VLQTLTECIPPLAIKPEEVELLWTLVKALWKVKAHLASAARPRLERVLVLLLALSLIAVLLQSEGGGMGG
G-
VGAAAKFLAAFSILTIKASAGDEVRKVTRRVMDLNSRLGRPEEAVNPEFRGEELEQTKYEAENVEVDCT
ETIRRFVDIPFLVSTRTQHNESEDRGSRLVLDVTRVRFIDLDVTKRSQIILVERVETDGVIAREMAKDMQ
SLQPE-
AALGKIAALEHFISLQDGHISNYVTAEINSVLGMLLLVLFIQQVRTAKIVWPTRREVTMVFIMAALAVFF
IVIGLG-NLVFMLGGISPIQITTLVLGEGVMVVRQTSTFPAEA-
VTILQNTYADLICAGRVTKADIESSLHIVVGARVARGTWYVIYHYEITQAQGESGIMQRAFRQATLSQS
LIDGRMEVKIKVVTSDRYA-
TYEIKGGQHRKGTRYNRVKVGHNIPVDVIPRMKMAFTPPNLVLVKTFGAVVQKRFAQPLTHVRHGA
VTIGPMEVSSPYITQKKARHAPGDSVVEIKPENIVRSVAVVPELIWAREHEDNSAVHEKYVKTTHKIAA
KRNSAVDKAQYDKRNQVHLSFIGLAVKVLPDQAIVAATAGTLLPTATTTVTEGLKGVAATIRLDPMEK
ALIAYIKRMRPQLNLGGKVDNKIVKCLNNNLDDVEYGKLGRRKIKPLTIKSVYDVEHKRATELVKIMPYK
DDVESRKSQFAEKELALFEPLKAFMMVFGFIARSVYALYLRVIRRTILLVKTKIVVNADSVLSAIKIIGVSIR
LRHKSTNVAGTKHHIKTIEKRARMTLAGAPSVKLHLSPGRVGKKGGRIKGYMPYKNYNIFDGLASGRR
NKVKDLAKLKKLDKTISVDGIRVRQ-
GGRVPKAALSNAFANSRVGVDIVGENIQEESNPRANAEMGQLFAEIDARVYHLVGALVGHALEAVL
AVCAILGIGKAHALLAVKLKINAGIEFHTAA-VEEAVAAA-
YAEPAGEAAVLLDVSEYPLGDLALTQASLDVCRMRRRQLGELKKTKKEAYMKLTCFSGKGDKVKILEC
TQTQAYEIVATRTAERHGIKAKGSFGKERAMMQIVPTKPVKVWVPVFVSDDELLAPVRALAETCGDG
VDVVKLTTDVKVTMFEKAVASLSMAGKATTIVYTVLEKVTELSKDIIYEQRAKAHYGRGRLRSHVVTHS

KQKRHLSMMKRTVVSSPSVSAARLTDGEVTVITGATMAFVMSEQDAV--
GEVDMHFHEEPRREAVLGIAGPETFLSTEQPRLLALLFLREYLMHIVDIGTTGNFSDAATIYDILRALQN
KLDRKLSADIAKVRMCLHDDVVKMGVTSEAPRRDAAGDSILSAFWINGVTANSDIILLVANLECETAP
ENIDCAVRLFYQRVTLQAKERGGDVSVALIPAQNQIEAAEGTEIYTIKGSTPGTAQMKLNVEAISRSIVK
SLAVQLYQVDLFTNDTWIATMLRAAQTVVYVGAADGKEAVDHVIERDTELEFAPQKDIVRRKIRVFTP
AAPSAVVIWDPAIDRADKADAQGTILKIYGPDLQDIACFTDIGAEPKIESLASWDEKEKYVVHRDDIPE
DI-
TLVGKSSKVNKEFGIPDVAVCAPSKPGVTESIDRGEPKAGSCGEVANLPYPQDSLVNRVSSANVQKNI
SMVLGGHPVGEMGISREVERHLETSMCEIFVDKDDVDVALDFYKISLTPLRVGIDRPNAKVEIAGKESR
KLMAVLDGDADLQRHPKVSDAAGIG-KAGAKAKLPEDALVRDGVQHGDIEFTSTDLPEGRV-
VTPVFNNVKAQLQIDKVIMRTFHVVTLSFSNDKGDEHLIKLRLNALKKSTRHYDMLLKIRDTECAQSP
HAIPVGKILKIDRLLQTICQAHYTAVEPLLADDLTALGVMGDQGLLHVVPRLMILAVLYLWSVEAIDHF
PLWDLQEAGLITDQTLGLNVRSREWSF-CISEVVGNTFFARTKDDDTEQRLYARNPI-VLL-
YGKFDMI-
ISPSGLRLESTAISDQQHGNQDKLILSTERLQDSMAYYVLEILLVFADMMILAWGSGDEPLKFLVAEQA
ITGRDNPQLVCLTRRTFPLDLQRDATFVLVLEDATMKNLSHKKMHMNLVLVKSKGWVENVSTKILISD
QAIASYAEDAGAHTQPSESALAVITSRVMAPIVLVVSQKPTAPSKTMFLEALSLLEAVTATVLNKPKKY
YIFEGDVIAVLGSIDAAVLIGMQRPQPDRMRRAVVAAAIRLAIALRGSDAISELEAGKINFSRAVSFVAA
TYKQLNPLMRAAIKLEAVDFRVRNHKVNTKRARPYKGHTKFLHKADEEKSLIAEGVDTIA
>Yoonia sp. 67-2
AVIRPVVMKVYAPVLMIAQSQVATVSGAGSPSLRAKTVYAKSGLQRKSETKWVVKDFYY-
GVVKLGNLVQLVSISVDNRAVCTVAQLVEQEVVVHGMVALDAVKKKIALEAIDGMTAEDPAKVIDY
NSELDLRKFRDADDRADMLPILLGLTLKIRFDPHQPLVHDLPIPVIFEVV-
GIMVQRGTIEVDECAISTAISDVDPIVYVSPIDLAAVSGDKMAYIDQESAVSAAKADIAL--
LALESAVLVVGI-PLLIPV-FAADADV-MSFANRSRTLKTIVNLAVSYPTVKRA-----IAAAL-
MEASQAAALAEKRGTAEQIRIVKRMQRSAGLADDPDLVLLEDAAEGNSNWAGQIYDDQLSGSSESD
SAGNIVFAANKRQNFQRSITYLRKVEIADQAAAPLMRGVKHNAKSRSARKLDDAIAALNFPNVPEGIA
IRAAAGKPTELLLHDATEGEEILMIIVRWKVTWKTAIIISLFT-
AGDVVGLIELGGIGAVGRKDGLLNRHHKMNGALIEQRRIVEKLTLRLRAALKQKIGAEQIIIFVVKVAGD
LVAKAKVQM---
SIGAVVAIQGETVFKRRGRQTVIGDEDATVEVLRGVAEGPADAQALLRIRVDHTLVKLSVLLIPE-
PVLVESVSIREGVGKALQIIQLDVYLLRDMGTGEETLAKSKSREMIREILQVDPITGVRLAFGELRERLVK
GGTMKELGTLL--
LAILTGITEPRMANLPAGVCRTFINAGPRAAVGMIFLLLDDAGKIYTIDGDLREITKSIKSLIGLVDAAKPK
IMSAGYAAARLQVRYADVMVLVSDANLYAYAEERRELLRMVPVYITNAAARRQLIIFALSLRGGHRRA
RAAIGVLIAAAILESQAEQGPVNHADPQVLLDDGEIVGSEMMISRQAALDLKQLIKTDVIVIFPEFQEK
MVVSLKFFCTDVAIFVIALICTVGAMLSREPSSAQESGAGFQKRIRAHSVLFALAVTFDRRNLRTS-V-
DLFLGLGLAAPRYGVLYTEQPKRKQMPTEPDKDKVDGFDLVNAIKEDTASNHELEMPEAMALAVE-
DASINVTAGVLAMNGDMAAMEKVDSKRKMALAVEKWTIGAKAIVDIIISGAAPFRACD-
VEVADVSAGLPTKSI-
FLYLAGANNAELEEEKLDGAGASTAEAAADKQKGILLVVPVLVVIQDSNTAALITNGGLEEWGTMAY
KKHYALAARMRLHMRTID------QMEPNKQKAEKIKTVKLEGDTRFRQLATEGVKEQMP-
LHALNPDFAV-LPVLVTPAVGRVAADEQVLGARVPCDASL-
RELQKSMTGNALPISGIRLLGMRAARAMVDEDIMVAGKVAA-

LLVIGSLETITHLVLRNDSEATLPWRLSVAERLESDTKRAGGYAARPFKSKPSLCNTQAHAKIADPIQEIA
AGYVATEARFVFPVEGIPIQLILASPHSRVGAAKRAGLGHLHEMAKELQAKAVIMVLNEPGTNTDGLL
NYLTTHQPTDLLFEDCLDIVIDMRRMSVV---
VQLNTV-
HVVVAGTRDVGDYEIMAAAAPSGLVQVERVDDRTGRERTLMWMYGAMEELWLLSVTYADWVVN
CAIGAILLHVYAQVINRAFSLDRLQLAGWPKQAARWWNTWQLWLNKDGFFSYEAAFRAVLAVLSA
DLDDYEMSDVEGVGRHLSRQELRVGYRIREQVAFEQLDPRTDILLQSRTRLSVIRHALKMVAILIVREIV
AEFTLVVAKEDVAMGHVECVLAESFDFTAKDADIGPVGIRTPDLILGDGTVFNEAVLGKIDLANTVADF
RYKMLADGGRELGVSWITLQNMLYARIWLPPAQAALKNLVPVAFFLGHTELNLIPFPALWAGISKARA
QKLMAGALIEKILAKVAAFADPEATTHQSKKAPHFKYYGEEVDPVYLAYILEIFALVATQVTVSDRLLTD
RYVLNLKI-RGFTSLLWLSAQIAEKGPSATVITVDTIMGLGALVLFIILL-RASVLIETLYYRYVYV-
MLGYQAAKDVADADPEHFLSLAHASRDVEYIEVIPAFFYYFKTAT-
FEPFNTALAREFFGLFEERLRIIEVLAVPPDRVEALVEFVALAGTLPTSADAAPGVIACGFCEPNAAGVLE
ADQDWNLHRHMGITLAFFKAQVSVHGWFDKGLHKDVAATGLSEAGALTTPFHAEVTAKQCVPAV
HRVLDLGEVEAALIEIDSAVLVADEEQTLADKPTTRENSVGAVGGVVLAGTMNMGANLDSVERGNN
IIIELEEENYSAVNGKKGDGKITDKVLMVLSEAYFPVLRRMASANALDKGRFGLLIDAPDDADNQDHG
GSLTIPAPTVLSESAPLFAESEIERIAWIAPIHLLGTLDAVLGFEPDQYSFISMMIDASLDIMETAEVLDDY
HVLEKGSRPPGFRVILIKGLHIRLLAGICTIIVVGSAGRAEITAYIQG-
AVPITLNKKTFLPAFAASSCHAIDIFSTMLRPPERSTIMTLILETVTEL-
LEVTRLENDKLERLETLLPGLDIAELLSLLAAATDFTAIVTIDRAITAILSVAAEAVVLREMLLTLAEIAVTAL
ALALALLLMLTKDQGLHQKEHAREAQMKDLIQGGVVLQVKAAARMMLASTADKVRLDTDEGTTSA
FASLNLATVSDGFYVLSSSVLQMSSDLMMIIKVARVLADKETKNALVVRHPEQVKTQSTYAEGSPGM
VLQTLTGAIPPLAIKPEEVELLWTLVAALWQVKAHLANAARPHNERVLILILALSLITVLLQSEGGGMGG
--
TNPPAKMLAAFSILTIKAKAGDEVRKVTRRVMDLNSRLGRPEESVNPEFRGAELEQSKYEAENVEVDT
METIRRFVDIPFLVSIRTQHNESEDRGARMVLDVTRIRFIDL-
VTKKSEIILAARAETDGIIAREMGKDLAALHPEAAALGKIAALQHFIAIQDAHISNYVTADIHAVLGMLLL
VLFIQEVRVAKVVWPTRREVTMVFILAALAVFFLVIGLGVQIVFMLSGISWNALTTIVLGEGIMVVRQTS
TFPEDS-
IGILQNTFAELICAGRVAKADIENSLHIVVAGRVARGTWYVIYLYEISQAQGQSGIMQRAFRQMTLSQ
SLIDGRMEVKIKVVTSDRYA-
TYEIKGGQHRKGTRYNRVKVGHNIPVQVLPRMKMMFTPPNLVLVKTFGAVVQKRFAQALTHIRHGA
VTIGPMEVSSPYIQNKKARHAPGDAVVDLKPDNIVRSVAVVPELIWARENEDQGAVHDKYVKTTHKL
AAKRKKAVDKAQYDHRNQVELSFIGLAVKVLPEQAIVHATSGQLMPTASVAVTEGMKGIAATIRLDP
MEKALIALIKRMRPQLNLAGKQDKKIVKCLNNNLDDVEYGKLGRRKIKPLTIKSVYDVDSRRATELVKL
MPYKDDVESRKSQFAEK--
ALFEPLKAFMMMFGFIARAVYALYLRVIRRTILLAKTKIVVNADSVLSAIKIIGVSIRLRHKSTNVAETNHH
IKTIEKRARMTLAGAGKVKLNLSPGKIGMKGGRIKGYMPYKNFNIFDGLASGRRAKVQDLAKLKDVDK
TIAVEGIRQKQQGGRTPKAQLSNAI-
NTRVGVDIVGENEQDESNPRANAEMGQIFAKIDARVYSHVGALIGHALEAVLAIVAIWGIGRAHSILA
VRLHINAGIEMHSAA-VEVAVAAA-
FAENAGEASLVTDVSEGPGPTLLVTATSVDVCRLRRRSLAELKKTKKDAYMSLTCFSGKGDKVKIVEAS
QTQAYEIVATRTAERHGIRAKGTYGKERAMMQILPTKPVKIWVPVFVSEEELLAPIRAFAETQGDGVTV
VKLSTDIKVTMFEKAVASLAVSGKATTVVYTVLEKVTELMKDIIYTQRAKAHYGRGRLRSHVVTTTKQR

RHLSMIKRTVVADPSMDAARLTDGEVSVITGATMAFVMSEQEAV--
GDVDLHFHEEPRREVAFGIAGPETFLTTEQPRLLAVLFLREYLLHIVEIGTTNGFADAATIYDVLRAMVY
KLDRKLSADIAKTRMVLVDDLMDMGVTEVAPRREAAGDKILSAYWINGVTANADIVLLVANLECPCV
PENIDCAIRLFYQRITLQAKERGGDVSVAVVAANKQIEAAEDTVIYTIKGKTPGTAQMKLTIEGISRSVV
KSFAVQLFQIDTFLDDTWIATWLRAADDVVFVAAEDGKEAVDHVVERDTEVEFAPQKDIVSRKIRIYT
PAAPSAVVIWDPAIDRADKAEAQGTILKIYGPNLQDINCFTDIGAEPKIESLKAWDEKEKYVVHRDDIP
EDIDTLVGKSSKVNKEFAIPDVAVCAPSKPGVTESIDRGEPKAGTCGEVANMAYPQESLVNAVSSANV
QKNISMVLAGHPVGEMGISREIERHLETNMCEIFVDKADVDVALDFYTLALTPLRVGLDPPNARVQID
GKESRKLMAVQDGDAALQRHPKVSAATGTGLGAGVKARMPDDALILDGVVHGALSFTATDLPAG-
----
KLRLNALKKNTRHYEMLLKIRETECAESPHAISVGKVLKIDKLLQTICVAHYKEIEPLLADDLTALGVMGR
QGLLHVVPRLMILAVLHLLS-ACAIIMPRWDLQKDAIITDQTMGLAVRERIWSY-
AISEVVGNTFFARTKEDDTEQRIHARNPV-VLL-LGKFVMI-
ISPSGLGLVSTAISEQELGNHEPLILSPERLQDEMGYYIIAILLVFVDQVILAYGSGNEPIKFLVADQAVTG
KQNPGMVTLTRRTFPLELQRDATFVMVFEDSTMKNLSHKKMHMNLILIKSRGWLVNVSTKILISDQAI
ASYAEDAGAHTQPSESALAAITSRVMAPIVLVVSQKPTAPSKSLFLEALALYDEVTATVLNKPKKYFIFE
GDVVSILGDVDAAVLLGMQRPQPDAMRRAVVAAAIRLAIASLASGVVTRLEEGLINFSRAVSLAAGIY
GELDLPAVAIIE-PTIAFRVRNHDVSTKRSKPYKAHTKFLHKADVDKSLIAEGVDTLA
>Yoonia sp. 67
AVIRPVVMKVYAPVLMIAQSQVATVSGAGSPSLRAKTVYAKSGLQRKSETKWVVKDFYY-
GVVKLGNLVQLVSISVDNRAVCTVAQLVEQEVVVHGMVALDAVKKKIALEAIDGMTAEDPAKVIDY
NSELDLRKFRDADDRADMLPILLGLTLKIRFDPHQPLVHDLPIPVIFEVV-
GIMVQRGTIEVDECAISTAISDVDPIVYVSPIDLAAVSGDKMAYIDQESAVSAAKADIAL--
LALESAVLVVGI-PLLIPV-FAADADV-MSFANRSRTLKTIVNLAVSYPTVKRA-----IAAAL-
MEASQAAALAEKRGTAEQIRIVKRMQRSAGLADDPDLVLLEDAAEGNSNWAGQIYDDQLSGSSESD
SAGNIVFAANKRQNFQRSITYLRKVEIADQAAAPLMRGVKHNAKSRSARKLDDAIAALNFPNVPEGIA
IRAAAGKPTELLLHDATEGEEILMIIVRWKVTWKTAIIISLFT-
AGDVVGLIELGGIGAVGRKDGLLNRHHKMNGALIEQRRIVEKLTLRLRAALKQKIGAEQIIIFVVKVAGD
LVAKAKVQM---
SIGAVVAIQGETVFKRRGRQTVIGDEDATVEVLRGVAEGPADAQALLRIRVDHTLVKLSVLLIPE-
PVLVESVSIREGVGKALQIIQLDVYLLRDMGTGEETLAKSKSREMIREILQVDPITGVRLAFGELRERLVK
GGTMKELGTLL--
LAILTGITEPRMANLPAGVCRTFINAGPRAAVGMIFLLLDDAGKIYTIDGDLREITKSIKSLIGLVDAAKPK
IMSAGYAAARLQVRYADVMVLVSDANLYAYAEERRELLRMVPVYITNAAARRQLIIFALSLRGGHRRA
RAAIGVLIAAAILESQAEQGPVNHADPQVLLDDGEIVGSEMMISRQAALDLKQLIKTDVIVIFPEFQEK
MVVSLKFFCTDVAIFVIALICTVGAMLSREPSSAQESGAGFQKRIRAHSVLFALAVTFDRRNLRTS-V-
DLFLGLGLAAPRYGVLYTEQPKRKQMPTEPDKDKVDGFDLVNAIKEDTASNHELEMPEAMALAVE-
DASINVTAGVLAMNGDMAAMEKVDSKRKMALAVEKWTIGAKAIVDIIISGAAPFRACD-
VEVADVSAGLPTKSI-
FLYLAGANNAELEEEKLDGAGASTAEAAADKQKGILLVVPVLVVIQDSNTAALITNGGLEEWGTMAY
KKHYALAARMRLHMRTID------QMEPNKQKAEKIKTVKLEGDTRFRQLATEGVKEQMP-
LHALNPDFAV-LPVLVTPAVGRVAADEQVLGARVPCDASL-
RELQKSMTGNALPISGIRLLGMRAARAMVDEDIMVAGKVAA-
LLVIGSLETITHLVLRNDSEATLPWRLSVAERLESDTKRAGGYAARPFKSKPSLCNTQAHAKIADPIQEIA

AGYVATEARFVFPVEGIPIQLILASPHSRVGAAKRAGLGHLHEMAKELQAKAVIMVLNEPGTNTDGLL
NYLTTHQPTDLLFEDCLDIVIDMRRMSVV---
VQLNTV-
HVVVAGTRDVGDYEIMAAAAPSGLVQVERVDDRTGRERTLMWMYGAMEELWLLSVTYADWVVN
CAIGAILLHVYAQVINRAFSLDRLQLAGWPKQAARWWNTWQLWLNKDGFFSYEAAFRAVLAVLSA
DLDDYEMSDVEGVGRHLSRQELRVGYRIREQVAFEQLDPRTDILLQSRTRLSVIRHALKMVAILIVREIV
AEFTLVVAKEDVAMGHVECVLAESFDFTAKDADIGPVGIRTPDLILGDGTVFNEAVLGKIDLANTVADF
RYKMLADGGRELGVSWITLQNMLYARIWLPPAQAALKNLVPVAFFLGHTELNLIPFPALWAGISKARA
QKLMAGALIEKILAKVAAFADPEATTHQSKKAPHFKYYGEEVDPVYLAYILEIFALVATQVTVSDRLLTD
RYVLNLKI-RGFTSLLWLSAQIAEKGPSATVITVDTIMGLGALVLFIILL-RASVLIETLYYRYVYV-
MLGYQAAKDVADADPEHFLSLAHASRDVEYIEVIPAFFYYFKTAT-
FEPFNTALAREFFGLFEERLRIIEVLAVPPDRVEALVEFVALAGTLPTSADAAPGVIACGFCEPNAAGVLE
ADQDWNLHRHMGITLAFFKAQVSVHGWFDKGLHKDVAATGLSEAGALTTPFHAEVTAKQCVPAV
HRVLDLGEVEAALIEIDSAVLVADEEQTLADKPTTRENSVGAVGGVVLAGTMNMGANLDSVERGNN
IIIELEEENYSAVNGKKGDGKITDKVLMVLSEAYFPVLRRMASANALDKGRFGLLIDAPDDADNQDHG
GSLTIPAPTVLSESAPLFAESEIERIAWIAPIHLLGTLDAVLGFEPDQYSFISMMIDASLDIMETAEVLDDY
HVLEKGSRPPGFRVILIKGLHIRLLAGICTIIVVGSAGRAEITAYIQG-
AVPITLNKKTFLPAFAASSCHAIDIFSTMLRPPERSTIMTLILETVTEL-
LEVTRLENDKLERLETLLPGLDIAELLSLLAAATDFTAIVTIDRAITAILSVAAEAVVLREMLLTLAEIAVTAL
ALALALLLMLTKDQGLHQKEHAREAQMKDLIQGGVVLQVKAAARMMLASTADKVRLDTDEGTTSA
FASLNLATVSDGFYVLSSSVLQMSSDLMMIIKVARVLADKETKNALVVRHPEQVKTQSTYAEGSPGM
VLQTLTGAIPPLAIKPEEVELLWTLVAALWQVKAHLANAARPHNERVLILILALSLITVLLQSEGGGMGG
--
TNPPAKMLAAFSILTIKAKAGDEVRKVTRRVMDLNSRLGRPEESVNPEFRGAELEQSKYEAENVEVDT
METIRRFVDIPFLVSIRTQHNESEDRGARMVLDVTRIRFIDL-
VTKKSEIILAARAETDGIIAREMGKDLAALHPEAAALGKIAALQHFIAIQDAHISNYVTADIHAVLGMLLL
VLFIQEVRVAKVVWPTRREVTMVFILAALAVFFLVIGLGVQIVFMLSGISWNALTTIVLGEGIMVVRQTS
TFPEDS-
IGILQNTFAELICAGRVAKADIENSLHIVVAGRVARGTWYVIYLYEISQAQGQSGIMQRAFRQMTLSQ
SLIDGRMEVKIKVVTSDRYA-
TYEIKGGQHRKGTRYNRVKVGHNIPVQVLPRMKMMFTPPNLVLVKTFGAVVQKRFAQALTHIRHGA
VTIGPMEVSSPYIQNKKARHAPGDAVVDLKPDNIVRSVAVVPELIWARENEDQGAVHDKYVKTTHKL
AAKRKKAVDKAQYDHRNQVELSFIGLAVKVLPEQAIVHATSGQLMPTASVAVTEGMKGIAATIRLDP
MEKALIALIKRMRPQLNLAGKQDKKIVKCLNNNLDDVEYGKLGRRKIKPLTIKSVYDVDSRRATELVKL
MPYKDDVESRKSQFAEK--
ALFEPLKAFMMMFGFIARAVYALYLRVIRRTILLAKTKIVVNADSVLSAIKIIGVSIRLRHKSTNVAETNHH
IKTIEKRARMTLAGAGKVKLNLSPGKIGMKGGRIKGYMPYKNFNIFDGLASGRRAKVQDLAKLKDVDK
TIAVEGIRQKQQGGRTPKAQLSNAI-
NTRVGVDIVGENEQDESNPRANAEMGQIFAKIDARVYSHVGALIGHALEAVLAIVAIWGIGRAHSILA
VRLHINAGIEMHSAA-VEVAVAAA-
FAENAGEASLVTDVSEGPGPTLLVTATSVDVCRLRRRSLAELKKTKKDAYMSLTCFSGKGDKVKIVEAS
QTQAYEIVATRTAERHGIRAKGTYGKERAMMQILPTKPVKIWVPVFVSEEELLAPIRAFAETQGDGVTV
VKLSTDIKVTMFEKAVASLAVSGKATTVVYTVLEKVTELMKDIIYTQRAKAHYGRGRLRSHVVTTTKQR
RHLSMIKRTVVADPSMDAARLTDGEVSVITGATMAFVMSEQEAV--

GDVDLHFHEEPRREVAFGIAGPETFLTTEQPRLLAVLFLREYLLHIVEIGTTNGFADAATIYDVLRAMVY
KLDRKLSADIAKTRMVLVDDLMDMGVTEVAPRREAAGDKILSAYWINGVTANADIVLLVANLECPCV
PENIDCAIRLFYQRITLQAKERGGDVSVAVVAANKQIEAAEDTVIYTIKGKTPGTAQMKLTIEGISRSVV
KSFAVQLFQIDTFLDDTWIATWLRAADDVVFVAAEDGKEAVDHVVERDTEVEFAPQKDIVSRKIRIYT
PAAPSAVVIWDPAIDRADKAEAQGTILKIYGPNLQDINCFTDIGAEPKIESLKAWDEKEKYVVHRDDIP
EDIDTLVGKSSKVNKEFAIPDVAVCAPSKPGVTESIDRGEPKAGTCGEVANMAYPQESLVNAVSSANV
QKNISMVLAGHPVGEMGISREIERHLETNMCEIFVDKADVDVALDFYTLALTPLRVGLDPPNARVQID
GKESRKLMAVQDGDAALQRHPKVSAATGTGLGAGVKARMPDDALILDGVVHGALSFTATDLPAGR
V-
VTPVFNTVKAQLKIDKVLLKAFHVVTLSFSNNKGEEHLIKLRLNALKKNTRHYEMLLKIRETECAESPHAI
SVGKVLKIDKLLQTICVAHYKEIEPLLADDLTALGVMGRQGLLHVVPRLMILAVLHLLS-
ACAIIMPRWDLQKDAIITDQTMGLAVRERIWSY-AISEVVGNTFFARTKEDDTEQRIHARNPV-VLL-
LGKFVMI-
ISPSGLGLVSTAISEQELGNHEPLILSPERLQDEMGYYIIAILLVFVDQVILAYGSGNEPIKFLVADQAVTG
KQNPGMVTLTRRTFPLELQRDATFVMVFEDSTMKNLSHKKMHMNLILIKSRGWLVNVSTKILISDQAI
ASYAEDAGAHTQPSESALAAITSRVMAPIVLVVSQKPTAPSKSLFLEALALYDEVTATVLNKPKKYFIFE
GDVVSILGDVDAAVLLGMQRPQPDAMRRAVVAAAIRLAIASLASGVVTRLEEGLINFSRAVSLAAGIY
GELDLPAVAIIE-PTIAFRVRNHDVSTKRSKPYKAHTKFLHKADVDKSLIAEGVDTLA
>Yoonia sp. 72
AVIRPVTMKTYAPVLMIAQSQVATVSGAGSPSLRAKTVYAKSGLQRKSETKRVVKDFYY-
GVVKLGNLVQLVSISVDNRAVCTVAQLVEQEVVVHGMVALDAVKKKIALEAITGMTAEDPAKVVDY
ASELDLRKFRDADDRADMLPILLGLTLKIRFDPHQPLVHDLPIPVVFEVV-
GIMVQRGTIEVDECAISTAISDVDPIVYVSPIDLAAVSGDKMAYIDQESAVSAAKADIAL--
LALESAVLVVGI-PLLIPV-FAADADV-MSFANRSRTLKTIVNLAVSYPTIKRA-IDG-IAASL-
MEASQAAALAEKRGTAEQIRIVKRMQRSAGLADDPELVLLEDAAEGNSNWAGQIYDDPLSGSSESD
SAGNIVYAANKRQNFQRSITFLRKVEISDQAAAPLMRGVKHNAKSRSARKVDDTIAALNFPNVPEGIA
IRAAAGSQVHLLLHDATEGEEILMIIVRWHVTWKTAIIISLFT-
AGDIVGLIELGGIGAVGRKEGLLNRHHKMNGALIEQRRIVEKLTLRLRAALKQKIGAEQIIIFVVKVAGDL
VAKAKIQM---
SVGAVVAIQGETIYVRRHRQTVIADEDATVEVLRGVAEGPADAQALLRIRVDHVLVKLSVLLIPE-
PVLVESVSIREGVGKALQIIQLDVYLLRDMGTGEETLAKSRSREMIREILQVDPITGVRLAFGILRERLVKG
GTMKELGTL---
LAIIKGITEPRMANLPDGVCRTFINAGPRAAVGMIFLLLDDAGKIYTIDGDLRELTKSIKSLIGLVDAAKPK
IMSAAYAAARLKVRYADVTVLVSDANLYAYAEERRELLRMVPVYITNAAARRQLIIFALSLRGGHRRAR
AAIGVLIAAAILESQAEQGAVNHASPAVLLDDGEIVGSEMMISRQAALDLKQIIKTDVIVIFPEFQEKMV
VSLKFFCTDVAIFVIALICTVGAMLSREPSSAQEDGAGFLKRIRAHSVLFALVATFDRRNLRTS-A-
NLFMALGLAPPRYGVLYTEQPKRKQMPSEPDADEVDGFDLVHAIKEDTASNHELDMPEAMALAVE-
DATINVTAGVLAMTGDMAAMEKVDSKRKMALAVTKWTISAEEIVDILISGAHPFRALD-
IETADVSAGMPRKNI--
LYLPGANNAEMEEEKLDGAGASTAEAAADKQKGILMVVPVLVVIQDSNTAALIANGGLEEWGTMAY
KKHYALAARMRLHMRTID------QMEPNKQKAEKIKTVKLEGDTRFRQLATEGVKEQMP-
LHALNPDFAV-LPVLVTPAVGRVAADEQVLGARVPCDASL-
RELQKSMSGDALPISGIRLLGMRAARAMVDEDIMVAGKVAA-
LLVILALETITHLVLRNDSEATLPWRLSVAERLEADMKRASGYAARPFQSKPSLCNTQAHAKIADPIQEI

AAGYVATEARFVFPVEGIPIQLILASPHSRVGSAKRAGLGHLHEMAKELHAKAVIMVLNEPDTNTDGL
LNYLTTQQPTDLLFEDCLDIVIDMRRMNVV--
VQLNTV-
HIVIPGARELGDREIMAAAAPSGLVQTERVDDRTGRERTLMWMYGAMEELWLLSVTYADWVVNCAI
GAILLHVYAQVINRAFSLDRLQLAGWPREASRWWNTWQLWLNKDGFFGYDAAFRAVLAVLSADLD
DYEMSDVEGVGRHLSRQQLRVGYRIREQVAFEQLDPRTDILLQSRTRLSVIRHALAMVAILIVREIVVEF
TLVVAKEDVAMGHVECVLADSFDFTAKDADIGPVGIRTPDTVLGDGTVFNEAVLGKIDFANTVADFR
YKMLADGGRDLGVSWITLQNMLYARIWLPPAQAALKNLVPISFFLGHTELNLAIFPALWAGISKARAQ
KLMAGALIEKILANVAAFADPEATTHQSKKAPHFKYYGEEVDPVYLAYILDIFALVATQVTVSDRLLTD
HYVLNLKI-RGFTSLLWLSAQIADKGPAATVITVDTIMGLGALVLFIILL-RASVLIETLYYRYVYV-
MLGYQAAKDVADANPEHFLSLAHASRDVEYIEIIPAFFYYFKTAT-
FEPFNTALAREFFGLFEERLRIIEVLAVPPDRVLVLVQFVALAGTLPTSAEALPGVIACGFCEPNAAGVLE
ADQDWNLHRSMGITLAFYDARESVKGWFDKGLHKDVAATGLREAGALTTPFHAEVTAKQCVPAVH
RVLDLGEVEAALIEIDSAVMVADEEQTLADKPTTRENSVGAVGGVVLAGTMNMGANLDSVERAGNII
IELEEENYQAVNGKKADGKLTDKVLMVLSEAYFPVLRRMASANALDKGRFGLLIDAPDDADNQDHG
RSLTIPAPTVLAESAPLFAETEIERIAWIAPIHLLGTLDAVLGFEPDQYSFISMMIDASLNIMKTAEV--
DYHVLEKGSRPPGFRIILIKGLHIRLLAGICTIIVVGSAGRAEVTAYIQG-
AVPITLNKKTFLPAFAASSCHAIDIFSTMLRPPERSTIQTLILETVTEL-
LEVTRLENDKLERMETLLPGLDIAELLGLVAAAADFTAIVTIDRAITAILSVAAEAVVLREMLLTLAEIAVT
ALALALALLLMLTKDQGLHQKDHAREAQMKELIQGGVVLQVKAAERMMLASTADKVRLDTDEGTTS
AFASLNLATVSDGFYVLSSSVLQMSSDLMMIIKVARVLADKETKNALVVRHPEQVKTQSTYAEGSPG
MVLQTLTGAIPPLAIKPEAVELLWTLVAALWKVKAHLANAARPHQERVLILILALSLITVLLQSEGGGM
GG--
TNPPAKMLAAFSILTIKAKAGDEVRKVTRRVMDLNSRLGRPEESVNPEFRGAELEQSKYEAENVEVDT
METIRRFVDIPFLVSIRTQHNESEDRGARMVLDVTRIRFIDL-
VTKKSEIILAARAETDGIIAREMGKDLAALHPQAAALGKIAALQHFIAIQDAHISNYVTADIHAVLGMLL
LVLFIQEVRVAKVVWPTRREVTMVFILAALAVFFLVIGLGVQIVFMLSGISWNALTTIVLGEGIMVVRQT
STFPEDS-
IGILQNTFAELICSGRVAKADIENSLHIVVAGRVARGTWYVIYLYEINQAQGQSGIMQRAFRQMTLSQ
SLIDGRMEVKIKVVTSDRYA-
TYEIKGGQHRKGTRYNRVKVGHNIPVQVLPRMKMAFTPPNLVLVKTFGAVVQKRFAQALTHIRHGA
VTIGPMEVSSPYIQNKKARHAPGDAVVDLKPDNIVRSVAVVPELIWARENEDQGAVHDKYVKTTHKL
AAKRKKAVDKAQYDHRNQVELSFIGLAVKVLPEQAIVHATSGQLMPTASVAVTEGMKGIAATIRLDP
MEKALIAYIKRMRPQLNLAGKQDKKIVKCLNNNLDDVEYGKLGRRKIKPLTIKSVYDVESRRATELVKL
MPYKDDVESRKSQFAEK--
ALFEPLKAFMMMFGFIARAVYALYLRVIRRTILLAKTKVVVNADTVLSAIKIIGVSIRLRHKSTNVAETNH
HIKTIEKRARMTLAGAGKVKLNLSPGKIGMKGGRIKGYMPYKNFNIFDGLASGRRAKVQDLAKLKNVD
KTIAVDGIRQKQQGGRTPKAQLSNAI-
NTRVGVDIVGENEQEESNPRANAEMGQIFAKIDARVFSHVGALIGHALEAVLAVVTIWRIGRAHSLLA
VRLHINAGIEMHSAA-VARAVAAA-FAQNAGEA-
LVADVSEGDLGALAVTAMSVDVCRLRRRSLAELKKTKKDAYMSLTCFSGKGDKVKIVEATQTQAYEIV
ATRTAERHGIRAKGTYGKERAMMQILPTKPVKIWVPVFVSEEELLAPIRAFAETQGDGVTVVKLTKDIK
VTMFEKAVASLAVSGKATTVVYTVLEKVTELMKDIIYTQRAKAHYGRGRLRSHVVTTTKQRRHLSMIKR
TVVADPSMDAARLTDGEVSVITGATMAFVMSEQEAV--

GDVDLHYHEEPRREVEFGIGGPETFLTTEQPRLLAVLFLREYLLHIVEIGTTNGFSDAATIYDVLRAMVH
KLDRKLSADIAKTRMVLVDDLMDMGVTEVAPRREAAGDKILSSYWINGVTANADIVLLVANLECPCV
PENINCAIRLFYQRITLQAKERDGDVSVAVVAADKQIEAAEGTVIYTIKGKTPGTAQMKLTIEGISRSVV
KSLAVQLFQIDTFMDDTWIATWLRAADDVVFVAAEDGKEAVDHVVERDTEVEFAPQKDIVSRKIRIYT
PAAPSAVVIWDPAIDREDKAEAQGTILKIYGPNLQDINCFTDIGAEPKIESLKAWDEKEKYVVHRDDIP
DDIDTLVGKSSKVNKEFAIPDVAVCAPSKPGVTESIDRGEPKAGTCGEVANMAYPQESLVNAVSSAN
VQKNISMVLAGHPVGEMGISREIERHLETNMCEIFVDKADVDVALDFYTLVLTVLRVGLDPPNARVQI
DGKESRKLMAIQDGEAALQRHPKVSAATGTGLGAGVKARMPDDALILDGVVHGALSFTATDLPAGR
V-
VTPVFNTVKAQLKIDKVLLKSFHVVTLSFSNNKGEEHLVKLRLNALKKNTRHYEMLLKIRETECAESPHA
ISVGKVLKIDKLLQTICVAHYKAIEPLLADDLTALGVMGRQGLLHVVPRLMIL------------
IFAKWDLQQQAIITDQTMGLAVRARDWSY-AISEVVGNTFFARTKEDDTEQRIHARNPV-VLL-
LGKFVMI-
ISPSGLGLQSTAETEQDLGNAEPLILSPERLQDEMGYYVIAILLVFVDQVILAYGSGEEPIKFLVADQAVT
GKQNPGMVTLTRRTFPLELQRDATFVMVFEDSTMKNLSHKKMHMNLVLIKSRGWLVNVSTKILISDQ
AIASYAEDAGAHTQPSESALAVITSRVMAPIVLVVSQKPTAPSKSLFLEALALYDAVTATVLNKPKKYFIF
EGDVVSVLGRLDAAVLLGMQRPQPDAMRRAIVAAAIRLAIAALASGIVTRLEEGLINFSRAVSFTR-
AY----------------AFRVRNHDVSTKRSKPYKAHTKFLHKADVDKSLIAEGVDTLA
>Yoonia sp. 76
AVIRPVTMKTYAPVLMIAQSQVATVSGAGSPSLRAKTVYAKSGLQRKSETKRVVKDFYY-
GVVKLGNLVQLVSISVDNRAVCTVAQLVEQEVVVHGMVALDAVKKKIALEAITGMTAEDPAKVVDY
ASELDLRKFRDADDRADMLPILLGLTLKIRFDPHQPLVHDLPIPVVFEVV-
GIMVQRGTIEVDECAISTAISDVDPIVYVSPIDLAAVSGDKMAYIDQESAVSAAKADIAL--
LALESAVLVVGI-PLLIPV-FAADADV-MSFANRSRTLKTIVNLAVSYPTIKRA-IDG-IAASL-
MEASQAAALAEKRGTAEQIRIVKRMQRSAGLADDPELVLLEDAAEGNSNWAGQIYDDPLSGSSESD
SAGNIVYAANKRQNFQRSITFLRKVEISDQAAAPLMRGVKHNAKSRSARKVDDTIAALNFPNVPEGIA
IRAAAGSQVHLLLHDATEGEEILMIIVRWHVTWKTAIIISLFT-
AGDIVGLIELGGIGAVGRKEGLLNRHHKMNGALIEQRRIVEKLTLRLRAALKQKIGAEQIIIFVVKVAGDL
VAKAKIQM---
SVGAVVAIQGETIYVRRHRQTVIADEDATVEVLRGVAEGPADAQALLRIRVDHVLVKLSVLLIPE-
PVLVESVSIREGVGKALQIIQLDVYLLRDMGTGEETLAKSRSREMIREILQVDPITGVRLAFGILRERLVKG
GTMKELGTL---
LAIIKGITEPRMANLPDGVCRTFINAGPRAAVGMIFLLLDDAGKIYTIDGDLRELTKSIKSLIGLVDAAKPK
IMSAAYAAARLKVRYADVTVLVSDANLYAYAEERRELLRMVPVYITNAAARRQLIIFALSLRGGHRRAR
AAIGVLIAAAILESQAEQGAVNHASPAVLLDDGEIVGSEMMISRQAALDLKQIIKTDVIVIFPEFQEKMV
VSLKFFCTDVAIFVIALICTVGAMLSREPSSAQEDGAGFLKRIRAHSVLFALVATFDRRNLRTS-A-
NLFMALGLAPPRYGVLYTEQPKRKQMPSEPDADEVDGFDLVHAIKEDTASNHELDMPEAMALAVE-
DATINVTAGVLAMTGDMAAMEKVDSKRKMALAVTKWTISAEEIVDILISGAHPFRALD-
IETADVSAGMPRKNI--
LYLPGANNAEMEEEKLDGAGASTAEAAADKQKGILMVVPVLVVIQDSNTAALIANGGLEEWGTMAY
KKHYALAARMRLHMRTID------QMEPNKQKAEKIKTVKLEGDTRFRQLATEGVKEQMP-
LHALNPDFAV-LPVLVTPAVGRVAADEQVLGARVPCDASL-
RELQKSMSGDALPISGIRLLGMRAARAMVDEDIMVAGKVAA-
LLVILALETITHLVLRNDSEATLPWRLSVAERLEADMKRASGYAARPFQSKPSLCNTQAHAKIADPIQEI

AAGYVATEARFVFPVEGIPIQLILASPHSRVGSAKRAGLGHLHEMAKELHAKAVIMVLNEPDTNTDGL
LNYLTTQQPTDLLFEDCLDIVIDMRRMNVV--
VQLNTV-
HIVIPGARELGDREIMAAAAPSGLVQTERVDDRTGRERTLMWMYGAMEELWLLSVTYADWVVNCAI
GAILLHVYAQVINRAFSLDRLQLAGWPREASRWWNTWQLWLNKDGFFGYDAAFRAVLAVLSADLD
DYEMSDVEGVGRHLSRQQLRVGYRIREQVAFEQLDPRTDILLQSRTRLSVIRHALAMVAILIVREIVVEF
TLVVAKEDVAMGHVECVLADSFDFTAKDADIGPVGIRTPDTVLGDGTVFNEAVLGKIDFANTVADFR
YKMLADGGRDLGVSWITLQNMLYARIWLPPAQAALKNLVPISFFLGHTELNLAIFPALWAGISKARAQ
KLMAGALIEKILANVAAFADPEATTHQSKKAPHFKYYGEEVDPVYLAYILDIFALVATQVTVSDRLLTD
HYVLNLKI-RGFTSLLWLSAQIADKGPAATVITVDTIMGLGALVLFIILL-RASVLIETLYYRYVYV-
MLGYQAAKDVADANPEHFLSLAHASRDVEYIEIIPAFFYYFKTAT-
FEPFNTALAREFFGLFEERLRIIEVLAVPPDRVLVLVQFVALAGTLPTSAEALPGVIACGFCEPNAAGVLE
ADQDWNLHRSMGITLAFYDARESVKGWFDKGLHKDVAATGLREAGALTTPFHAEVTAKQCVPAVH
RVLDLGEVEAALIEIDSAVMVADEEQTLADKPTTRENSVGAVGGVVLAGTMNMGANLDSVERAGNII
IELEEENYQAVNGKKADGKLTDKVLMVLSEAYFPVLRRMASANALDKGRFGLLIDAPDDADNQDHG
RSLTIPAPTVLAESAPLFAETEIERIAWIAPIHLLGTLDAVLGFEPDQYSFISMMIDASLNIMKTAEV--
DYHVLEKGSRPPGFRIILIKGLHIRLLAGICTIIVVGSAGRAEVTAYIQG-
AVPITLNKKTFLPAFAASSCHAIDIFSTMLRPPERSTIQTLILETVTEL-
LEVTRLENDKLERMETLLPGLDIAELLGLVAAAADFTAIVTIDRAITAILSVAAEAVVLREMLLTLAEIAVT
ALALALALLLMLTKDQGLHQKDHAREAQMKELIQGGVVLQVKAAERMMLASTADKVRLDTDEGTTS
AFASLNLATVSDGFYVLSSSVLQMSSDLMMIIKVARVLADKETKNALVVRHPEQVKTQSTYAEGSPG
MVLQTLTGAIPPLAIKPEAVELLWTLVAALWKVKAHLANAARPHQERVLILILALSLITVLLQSEGGGM
GG--
TNPPAKMLAAFSILTIKAKAGDEVRKVTRRVMDLNSRLGRPEESVNPEFRGAELEQSKYEAENVEVDT
METIRRFVDIPFLVSIRTQHNESEDRGARMVLDVTRIRFIDL-
VTKKSEIILAARAETDGIIAREMGKDLAALHPQAAALGKIAALQHFIAIQDAHISNYVTADIHAVLGMLL
LVLFIQEVRVAKVVWPTRREVTMVFILAALAVFFLVIGLGVQIVFMLSGISWNALTTIVLGEGIMVVRQT
STFPEDS-
IGILQNTFAELICSGRVAKADIENSLHIVVAGRVARGTWYVIYLYEINQAQGQSGIMQRAFRQMTLSQ
SLIDGRMEVKIKVVTSDRYA-
TYEIKGGQHRKGTRYNRVKVGHNIPVQVLPRMKMAFTPPNLVLVKTFGAVVQKRFAQALTHIRHGA
VTIGPMEVSSPYIQNKKARHAPGDAVVDLKPDNIVRSVAVVPELIWARENEDQGAVHDKYVKTTHKL
AAKRKKAVDKAQYDHRNQVELSFIGLAVKVLPEQAIVHATSGQLMPTASVAVTEGMKGIAATIRLDP
MEKALIAYIKRMRPQLNLAGKQDKKIVKCLNNNLDDVEYGKLGRRKIKPLTIKSVYDVESRRATELVKL
MPYKDDVESRKSQFAEK--
ALFEPLKAFMMMFGFIARAVYALYLRVIRRTILLAKTKVVVNADTVLSAIKIIGVSIRLRHKSTNVAETNH
HIKTIEKRARMTLAGAGKVKLNLSPGKIGMKGGRIKGYMPYKNFNIFDGLASGRRAKVQDLAKLKNVD
KTIAVDGIRQKQQGGRTPKAQLSNAI-
NTRVGVDIVGENEQEESNPRANAEMGQIFAKIDARVFSHVGALIGHALEAVLAVVTIWRIGRAHSLLA
VRLHINAGIEMHSAA-VARAVAAA-FAQNAGEA-
LVADVSEGDLGALAVTAMSVDVCRLRRRSLAELKKTKKDAYMSLTCFSGKGDKVKIVEATQTQAYEIV
ATRTAERHGIRAKGTYGKERAMMQILPTKPVKIWVPVFVSEEELLAPIRAFAETQGDGVTVVKLTKDIK
VTMFEKAVASLAVSGKATTVVYTVLEKVTELMKDIIYTQRAKAHYGRGRLRSHVVTTTKQRRHLSMIKR
TVVADPSMDAARLTDGEVSVITGATMAFVMSEQEAV--

GDVDLHYHEEPRREVEFGIGGPETFLTTEQPRLLAVLFLREYLLHIVEIGTTNGFSDAATIYDVLRAMVH
KLDRKLSADIAKTRMVLVDDLMDMGVTEVAPRREAAGDKILSSYWINGVTANADIVLLVANLECPCV
PENINCAIRLFYQRITLQAKERDGDVSVAVVAADKQIEAAEGTVIYTIKGKTPGTAQMKLTIEGISRSVV
KSLAVQLFQIDTFMDDTWIATWLRAADDVVFVAAEDGKEAVDHVVERDTEVEFAPQKDIVSRKIRIYT
PAAPSAVVIWDPAIDREDKAEAQGTILKIYGPNLQDINCFTDIGAEPKIESLKAWDEKEKYVVHRDDIP
DDIDTLVGKSSKVNKEFAIPDVAVCAPSKPGVTESIDRGEPKAGTCGEVANMAYPQESLVNAVSSAN
VQKNISMVLAGHPVGEMGISREIERHLETNMCEIFVDKADVDVALDFYTLVLTVLRVGLDPPNARVQI
DGKESRKLMAIQDGEAALQRHPKVSAATGTGLGAGVKARMPDDALILDGVVHGALSFTATDLPAGR
V-
VTPVFNTVKAQLKIDKVLLKSFHVVTLSFSNNKGEEHLVKLRLNALKKNTRHYEMLLKIRETECAESPHA
ISVGKVLKIDKLLQTICVAHYKAIEPLLADDLTALGVMGRQGLLHVVPRLMIL------------
IFAKWDLQQQAIITDQTMGLAVRARDWSY-AISEVVGNTFFARTKEDDTEQRIHARNPV-VLL-
LGKFVMI-
ISPSGLGLQSTAETEQDLGNAEPLILSPERLQDEMGYYVIAILLVFVDQVILAYGSGEEPIKFLVADQAVT
GKQNPGMVTLTRRTFPLELQRDATFVMVFEDSTMKNLSHKKMHMNLVLIKSRGWLVNVSTKILISDQ
AIASYAEDAGAHTQPSESALAVITSRVMAPIVLVVSQKPTAPSKSLFLEALALYDAVTATVLNKPKKYFIF
EGDVVSVLGRLDAAVLLGMQRPQPDAMRRAIVAAAIRLAIAALASGIVTRLEEGLINFSRAVSFTR-
AY-----------------AFRVRNHDVSTKRSKPYKAHTKFLHKADVDKSLIAEGVDTLA
>GCA_900102015
AVIKPVTMDVYAPVLMIRQQQVATVSGAGSPSLRAKTVYRKSGLQRKTLTKWVAKPIYY-
GVLQMSKLPQLVSISVDNRAVCTVAQVVEQEVVVHGLVALEAVKRKIALEAIDGMTSEDPAKVVDFE
GELELRRFRDAEDRADMLPVLLGLTLKVKFDPHQPVVHDLPVPVIFEVV-
GLMAQKGTIEVDECGISTAVSDVDPIAYVSPIDLDAVAGEKMAFIDQEAAVANAREEIALEELALESTV
LVVGI-SLLIPV-FAEDGAR-MSFANRSRTLESIVELAVAFPELGRARIS-GIAAAM-
MGRARAAALAEKRGTAEEIRIVKRMMRSAGLADDPELVILEDAAEGNAQWAGQIYDDPVSGSSESD
SQGNIVHAANKRQNFARSITFLRKVEISQQAAAPLMRGVKHNAKSRSSRKLSDTIAELNFPNVPEGVA
IRAEAGRRQDTLLHDAAEREEILMLIIRLHVTWKTAVLIVLLSRAGDVVGLMTLGGIAAVGRQDGLLNR
HHKLNGSLIEQRRVIEKLTLRLRAALKQKLGAEQVIIFVVKVAGDLVAEATVQMG--
DIGAVVAIQGDVIFVRRHRQTVVTDQESTVEILRGVAQGPADAQALLRIRVDHTVVEISVLLIPE-
PVLVESVTIREGVGKALQIIQLDVYLLRDMGTGDETLAKSRSRELIRDLLTLEPITGVRLAFGGLKERQAR
SMKLKDLRTLL--
LSILECVTEPRMQDHPGGVRRDLIEAGPRAAVGMLFLILDEAGRIYTIDGELREIPKTIKSLIGLLDAARPK
VLSAGYATERLRIRYADCMVLTSEANLYAYREERGELLRMVPVYIVTAAKRRQLVIFALSLRGGHRRAK
AALEVLIAAAIIESQAVKGAVHHEEPRILLDDAEVVGSEMMISRQAALDLKQVLKTDVIVVFPEFQEKM
VVSLRFFCTDVAIFVIALITTVGQMLSRSPSHAQETGAGYGKRIRGHSALFALAVTLDRRNMRD--
APTLFLGLGLAPPRYGVLYTQQPKKKQMPAEPDGDKVDGFDLVNAVKADAAS-
HELELREAMALAVEADAPVNLTAGVLALTGGMAAMAKVDSKRKVALAATKWNIAKLELADLHLSGA
APFRALD-IELADVSAALAAKDI--
LFLPGANNAELEEEKLDGAGSSTVDSAEDRQRGILLVVPVVVVIQDSNAASLVANGGVEEWGTMAY
KKHYAFAARMRLHMRTID-IELGVSLEPNKQKAEKIKTVKLEGDTRFRQLAVEGVKEQMP-
LHALNPDFAVLVPLLVTPAVGRVAAEREVLGARVPCDASL-
RELQKSQTGQALPISAIRLLGMRAARGMIEEDIMVAGKVAA-
LFKIGALETVAAFVLRNDSEATLPWRLSVAERLEADHKRAGGYAARPFRSKPSLCNVQAHAKVSDPIQ
EIAAGYIAKEERFVFPVEGLAIQLILSSPHSRVGTAKRAGEGHLHEMAKELQGKAVLMVSNAPDTNTEG

LLKYLTTGRPTDVLFEDCLDIIIDMRRMAVV--
VQLNVV-
HVVVPGCGPVGEFDIMSAAADSGLVQVERVDERTGRERTIMWMYGAMEELWLLSVTWADWVVDA
PIGAILLHVYAQSINRAFSLTRLQLQGWPREAERWWNTWQLWLNKDGFFGYEAAFRAVKQVLKADL
HDYDMSDVEGVGRYQSRQELRVGYRIREEVEFERLSPRTDILLQSTTRLAVLRHALTIVAILIIRDIVAEFT
-
VVAQEDVAMGHVECVLADSFDFTAKNAHIGPVGIRTPATVLGDGTVFKDAVLGKVDFANTVADFRY
KVLMDGGRELGVRWVTLQDLLVGRIWLPPAQAALKDLEPIAFFLGHPELNMDLAPALWAGISKARA
QRLMASVLVERILDRVAAFAAPEATTHQSRKEKHFKYYGEEVDPVHLAYILPMFALVATQVTVSDRLL
KDRYALNMRV-RGMTSLLLLGLQLSANGPAATVITVDTVMGLGALVLFVILL-RDTVIIETLYHKYVY--
MRGYEAAKDVADADPEHFLTLAQVSRDVEWIEIVPAFFYYFKTAT-
FEPFNTAVAKEFFGLFEERLRVVDVLVAPPPRVEEIVEFVALAGTLPTAGDALPGVIACD-
CTPNAAAVLEADQDWNLHRSMGITLASFKAQQSVKGYFDKGLHKNVAQFGLGEAGALTTPFIAGSE
AKRCAPAVHRVLDLGEIEAALIEIDSAVRVATEEQTLADKPTTREKSVGAVGGVVLAGTMNMGAALE
QVERAGNIIIELEEETYHAVNGKKADGKLTDKVLMVLSGAYFPVLRTLTSANALDRGVFDILVDAPDDA
ENLDHGREMTIPAPSVHTETTPLYAEAEIDRIAWIAPLHLLGTLAAVLGFEPDQYSFISMMIEASLEIMET
AEVMEDYHVLEKGARPPDFRTILIRGLHVRTLAGVGTIIVVGSAGRSEVTAYIQG-
AVPISLRRQTFLPAFAPSSCHKVDIFSTMLRPPERSAVQTLILETVAEL-
LEVTRIENSKLERMALLLPGLDVARLLELVAAAEDFASIVTISAAITAILSVAAEPVVLRELLLTLAEIAVTAL
ALALALLLMLTPDKGLSQREDAREKQMKELIQGGVVLQVKAADRMVLASTADKVRLDTDEGTTSAFA
SLNLATVSDGFYVLSSAVLQLSSDLVMIVQVARVLADKEHKNALVVRHPEQVKTQPTYAEGSPGMVL
QTLTSSIPPLAIKPEEVELLWTLVAALWRVKAHLASAARPHAERVLVLILALALIVVLLQSEGGGMGGG-
MGAAAKGLAAFSILTIKAKVGDEVRKVTRRVMDLTSRLGRPEESVKPEFRGAELEQAKFEAENVEVDT
METIRRFVDIPFLVSTRTQHNESEDRGARMVLDVTRVRFIDL-
VTKRSEIILAARVETDGIIAREMAKDLQALQAELAALGKVAALQHFISLSDGHISNYVTQDIHSVLGMLI
LVLFAQQTRISKVVWPTRREVTMVFLLAAVALFFLVIGLAVQIVFMLSGISYQQITTLVLGEGVMGVRQ
TSTFPSES-
IGILQNTYADLIAAGRVAKADIEKSLHIVVGARVARGTWYVIYLYEISQAQSEAGIVQRAFRQATLSQSL
IDGRMEVKIKVVTSDRYA-
TYEIKGGQHRKGTRFNRVKVGHNIPVDVLPRLKMAFGPPNLILVKTFGAVVQKRFAQPLTHIRHGAVT
IGPMEVSSPYIKSKKARHAPGDSVVDLKPDNIVRSVAVVPELIWARENEDNGAVHDKYTKTTHKLQAK
RRKAVDKAQYDKRNQVHMSFIGLAVKVLPEKAIVHATAGTLLPTSSVAVTENLKGIAATIRLDPMEKA
LIAYIKRMRPQLNLAGKSDKKIVKCLNNNLDDVEFGKLGRRKIKPLTIKSVYDVEHTQATELVKLMPYK
DDVEGRKSQFAEK--
ALFEPLKAFMMLFGFIARAVYALSLRVVRRTILMVKCRIVVNASSVLSAIKVIGVSIRLRHKSTNVADTKH
HIKTIEARARMTLAGAGKVKLHLRDGAVGLKGGRIKGYMPYVNYNIFDGLASGRRSKVKELAKLKKVD
KEIQVEGIRVKQQGGRQPKADLSNAIANTRVDVDIVGENVQEASNPRANAEMGQIFAAIDARVYSLV
GALIGHALAAILAVASIWGIGIRAHNLLAVAFRVNAGIELHSAA-VEVAVAAA-
WAQAAGEAALFADASESYLKLLSVTAASLAVCRLRRRGLSELKKTKKDAYMKLTCFGGKGERVKIVEA
TQTQAYEIVATRTAERHGIHAKGSYGKERALMQITPTKPVKVWVPVFVSEPELLAPIRAFAETNGDGVT
VVKLTKDIKITMFEKAIASLAVAGKATSVLYTVLEKVTELAKDIIYEQRAKAHYGRGRLRSHVVTHTKSKR
HLSMIRRTVVADPKMDAERLTDGEVSVITGATMAFVMSEQDAV--
GDVKFHFHEEPRREAEVGIAGPETFLATEQPRVIALVFLRAYLIHIVEIETVGDFANAATIYDILRPLQNKL
DRKLSADIAKVRMCLADDVAAMGVTDQAPRREDAGDPILSAFWINGVPANADIILLVANLETPSVPE

NIDAAIRLFYQRVTLQAKERGGDVSVAVVKAENQIEAAEGTVIYTIKGTLPGTLQMKLTVEGVSKSIVKS
MAVQLFQVTTFLDDTWIATLLRAASAVVFVEAKEGREAVDHVVERDTALEFAPQKDVLARKIRIYTPA
APSAVVIWDPAIEREDRADAQGTILKIYGPNLQDISCFTDIGAEPKIESLKAWDEKEKYVVHRDEIPEDIT
TLVGKSSRVNKEFGIPDVAVCAPSKPGVTESIDRGEPKAGSCGEVANMAYPQESLVNAVSSANVQKN
ISMVLAGHPVGEMGISRTVERHLETNMCEIFVDREDVDVALDFSRLSLTPLRVGLDRPNARVEIEGRES
RKLMAVLDGEADLQRHPKVSEAVGVVTTAGTKAKLPDDALIFDGVLHGDVSFTATDIPEGRV-
VTPVMLQVKAQLKIDKVMVKTFHVVTLSFSSNKGEEHLIKLRLNALKKSTRHYEMPLKIRETECAESPH
AIPVGKILKIDNLLQTICQAHYTEIEPLLADDLTALGVMGDQGLLHVVPRLMILAVMHLAGLEARRAVP
EWGLQARALVTDQTMGLAIRERDWSF-AISEVVGNTFFAKTADEDTEQRLHARNPV-VLL-
YGKFGMV-
ISPSGLRLASTAPGDMDRENEEELILSAERLQDEMGYYVLRVLLVFVDEIILAYGSGTQPLKFLVAEQAV
TGRDNPGMVTLTRRTFPLDLQRDAVFVLVFEDSTMKNLSHKKMHMNLILIKNKGWVVNVSTKILISD
QAIASYAEDAGAHTQPSESALAVITSRVIAPIVLVVSQKPSAPSKSLFLEALSLYDQVTATIQNAPKRYFI
FEGDLVSVLGGLDAAVLLGLQRPQPDRMRRAVIAAAIRLAIALRASEVPARLDEGLINFSRAVSFAARL
WGALEAV--A-------AFRVRAHSVSTKRSKPYKEHPKFLHKADEEKSLVAAGVDTLA
>GCA_900109295
AVIKPVTMPIYAPVLMIRQQQVATVSGAGSPSLRAKTVYAKSGLQRKSETKWVAKPIYY-
GVLKMGNLVQLVSISVDNRAVCTIAQLVEQEVVVHGMVALEAVKKKIALEAIEGMTAEDPAKVIDY--
EIALKKFRDAEDKADMLPVMLGLTLKIKFDPHQPLVHDLPIPVIFEVI-
GIMAQKGTIEVDECGISTAVSDVDPIAYVSPIDLKAVKGKEMKYIDQEAAVSAAKEAIAL--
LALESGVLVVSIKPLLIPV-FAAEA-T-MSFANRSRTLKTIVNLAVGYPELSRARLGIGIASSM-
MDRAQAAALAEKKGTAEQIRIVKRMQKSAGLADDADLVILEDAAEGNSSWAGQIYDDPISGSSESDS
AGNIVY-
ANKRQNFARSITHLRAVEISDKAAAPLMRGVKHNSKSRAARKLDDRIAQLNFPNVPEGIAIAAGANKP
SEILLHEAAEGEEILLIIVRWHTTWKTAIIITLVAVADNIVGLIDLGAIGAVGRKDGMLNRHHKLNGSLIE
QKRVIEKLTLRLRASLKQKLGAEQVIIFVVKVTGDLVAQAKIQMG--
DVGTVVAIQGPTIFVRRSRQTVIGDQEATVEILKGVAEGPADAQALLRIRVDHTLVKLSVLLIPEVPVLV
ESVTIREGVGKALQIIQLDVYLLRDMGTGEETLAKSKSREVIRELLQIDPITGVRLAYGQLRERQSKALTIK
EIDT----
LSIMEHITEPRMENLPEGVCRTFIAAGPRAAVGMIFLIIDPAGKIYEIDGDLRENTKSIKSMIGLVDAAKP
KTLSAGYATARLKIRYSDVTVLISDADLYAYAEERRELLRMVPVYIIVEAKRRQLIIFALSLKGGHRRASM
ALGIMIAAAILESQAEEGAVNHAEPKILLDDAEVVGSEIMISRQAALDMKQIIKVDVIVIFPEFQEKMVV
SLRFFCTDVAIFVIALICTVGSMLSREPSHAQETGAGFQKRIRAHSVLFALAVTFDRRNLRSD--
PVLFINRGLASPRYGIFYTEQPKRKQMPSEPDKDRVDGFDLVHAIKNDATNNHALDMPEAMALAVE-
SATINVSAGVLALSGEMTAVEKVDSKRKMAVAVEQWNISNIAIEDILASGAAPTRSLD-
AEVADFSAMLFNKKIWFLYLPGANNAEMEEAKLDGAGSSTAEAAEDKQINILLVRPVLVVIQDSNTAS
LIANGGLEEWGTMAYKKHYALAARMRLHMRTID-------
MEPNKQKAEKIKTVKLEGDTRFRQLATEGVKEQMP-
LHALNPDFAVLIPVLVTPAVGRVAADEKVLGAKVPCDVSL-
RELQKSQKGRALPISGIRILGMRAARAMIEEDIMVAGKVAA-
LLVIGALETICGLVLMNDSEATLPWRLSVAERLEADTKRAGGYAARPFKSKPSLCNTQAHAKIADPIQEI
AAGYVALEKRFVYPVESIAIQLILASPHSRVGNAKRAGLGHLHEMAKELQSKAVMMVLNEPDTNTEG
LLNYLTTRQPTDLLFEDCLDVVIDMRRMSVV---
VQLNTV-

HVLVPGVRDMAKREIMQAAAHSGLVQVERVDDRVGRERTLMYMYGSMEELWLRSITWGDWVIDC
AIGAIVLHVYAQSINRAFSLERLQLAGWPPEASSWWNTWQLWLNKDGFFGYAAGFRAMAAVLKAD
LADFEFNDTEGVARLLSRQEIRAGYRIRQQIAFARLDPRTDILLQSKTRLSVIRHALAVVAILIVREVVAEF
TVVVAMEDVAMGHVECVLADCFEFTAKDADIGPVGIRTPDPVLGDGTVMKEAVLGKIDLANTVADF
RYKMLVDGGRKLGVEWVSV-
NMLYDRIWLPPAQAALKNMVPIVFFLSHPELNLAIFPALWAGISKARAQQLMAGALIEKILDDIAAFA
DPEATTHQSNKAKHFKYYGEDVDPVYLTYILPVFALVATQVTVSDRLLKDRYVLNLKIFRSFTSLLWMS
LQLGNKGPAATIITLGTVAGLGALVLFIILL-RAVVLLETLYYRYVY--
MKGYQAAKDVADADPEHFLILASATRDVEWIEIIPAFFYYFKTATSRLTKNTANAREFFGLFRERLRVVD
VLCVPPDRVEALSDFVALAGTLPTSAEA-
PGVIACDFCEPNAAAVLEADQDHNLHRSMGITLAFYDARASVKGWFDKGLHKDVAATGLTEAGALT
TPFHAGADAKRCVPAVHRVLDLGEIAAALIEIDSAVVVADEEQTLADKPTTRDNSVGAVGGVVLAGT
MNMGANLDSVERGGNIIIELEEDNYGAVNGKKADGKLTDKVLMVLSQAYFPVMTRTSSANALDKGV
FDLLIDAPDDADNLDHGGTLTIPAPSIL-
ETAPLFAESEIERIAWIAPIHLLGTLDAVLGFEPDQYSFISMMIEASLDIMDTAEVLDDYHVLKKGSRPPG
FRVILIKGLHIRLLAGIGTIIVVGSAGRVEITAYIQG-
AVPITLNKVTFLPAFAASSCHVIDILSTMLRPPERSAIEMLILETVAEL-
LEVTRFENDKLERLDVLLPGLDLAELLKLVALASDFAAIVTIDRAIAAILSVAAEAVVLREMLLTLAEIAITA
LALALALLLMLTRDKGLTQKEQAREDQMKDLIQGGVVLQIKAEDRMMLASTADKVRLDTDEGTTSAF
ASLNLATVSDGFYVLSSAVLQMSSDLMMIVNVARVLADKETKNALVVRHPEQVKTQTTYAEGSPGM
VLQTLTAAIPPLAIKAEDVELLWTLVAALWQVKAHLANAARPHKERVLVLILALALITVLMQSEGGGIG
GG--
GAPAKLLAAFSILTIKAKAGDEVRKVARRVMDLNSRLGRPEEAVNPEFRGAELEQSKYEAENVEVDTM
ETIRRFVDIPFLVSTRTQHNESEDRGSRMVLDVTRIRFIDL-
VTKRSEIILAARAETDGIIAREMQKDLQALHEEVAALGKIAALQHYIAIQDGHISNYVTADINSVLGMLL
LVLFINETRVAKVVWPTRREVTMVFIMAALAVFFLVIGLAVQIVFMLSGISWQQLTTIVLGEGVMAVR
QTSTFPADS-
IGILQNTYADLICSGRVAKADIENSLHIVVAGRVSRGTWYVIYHYDISQAQQNSGIMQRAFRQATLSQ
SLIDGRMEVKIKVVTSDRYAQTYEIKGGQHRKGTRYNRVKVGHNIPVQVLPRMKMCFTPPNLVLVKT
FGAVVQKRFAQPLTHIRHGAVTIGPMEVSSPYITSKKARHAPGDSVVDLKPENILRSVAVVPELIWARE
NEDNGAVHDKYVKTTHKLAAKRKKAVDKAQYDHRNQVELSFIGLAVKVLPAKAIVHATSGTLMPTAS
VAVTEGMKGIAATIRLDPMEKALIAYIKRMKPQLNLGGQTDKKIVKCLNNNLDDVEYGKLGRRKIKPITI
KSVYDVESRRATELVKIMPYKDDVEGRKSQFAEK--
AIFEPLKAFMMLFGFIARSVYALYLRVIRRTILLVKTKIVVNAGTVLSAIKYIGVSIRLRHKSTNVAETNHHI
KTIEARARMTLAGAGKVKLNLHPGRVGTKGGRIKGYMPYKNFNIFDKLASGRRAKVAALAKLKKVDKE
IKVDGIREKQQGGRTPKAQLSNAIANSRVGVDIVGENEQEESNPRANAEMGQIFAKIDARVYSHVGA
LIGHALAAVLAVVAIWGISQAHNLLADKLKINAGIEMHSAA-VDAAVAAA-
FAENGGEASTLADVSEAYLGSLLVTANSMDICRLRRRSLNELKKTKKDAYMSLTCFGGKGDKVKIVEAT
QTQFYEIVATRTAERHGIRAKGTYGKERAMMQITPTKPVKIWVPIFVSEDELLAPIRAFAETVGDGVTV
VKLTTDVKVTPFEKAVASIAVTGKSTSVIYTVLEKVTELSKDIIYTQRAKAHYGRGRLRSHVVTTTKQKRH
LSMMKRTVVADPSMDAARLTNGEVSVVTGATMAFVMSEQDAV--
GDVDLHYHEEPRREAAVGIAGPETFLATEHPRTLALLFLRAYLLHIVEIETEKGFSDAATIYDVLRAMQN
KLDRKLSADIAKIRMVLTDNLTDMGVTEVAAKREAAGDKILSAFWINGVTANADIILMVANMEVPCV
PENINCAVRLFYQRITLQAKERDGDVSVAVVPAANQIEAAEGTVIYTIKGKEPGSAQMKLTIEGISRSIV

KSLAVQLFQVDTFLDDTWLGTLLRAAIEVVFVDAGEGKEAVDHVVERDTELEMAPQKDIVARKIRIYTP
AAPSAVVIWDPAIEREDKADAQGTILKIYGPNLQDINCFTDIGAEPKIESLKAWDEKEKYVVHRDDIPE
DI-
TLVGKSSKVNKEFAIPDVAVCAPSKPGVTESIDRGEPKAGTCGEVANMSYPQESLVNQVSSANVQKN
ISMVLGGHPVGEMGISRSIERHLETSMCQIFVDKDDIDVALDFYILALTALRVGLDPPNARVQIDGKES
RKLMAIQDGDADLQRHPKVSQATGGQ-KAGVKARMPDDALILNGVMHGDLSFTATDLPDGRV-
VTPVFNQVKAQLTIDKVMLREFHVVTLSFSNDKGEEHLVKLRLNALKKSTRHYEMLLKIRETECAESPH
AISVGKVLKIDKLLQTICEAHYKAIEPLLADDLTALGVMGEQGLLHVVPRLMILAVLHLWGIGAAKFIAV
WDLQADAIITDSTMGLEARDRSWSFVAISEVVSNTFFARTADDDTEQRIRAQNPV-VLL-
YGKFDMT-
VSPSGLGLDSTEDSDQERGNADDLILSSERLQDEMSYYILRILLVFVDEVILAFGSGERPIKFLVAEQAVT
GRQNPGMVTLTRRTFPLELQRDATFVMVFEDSTMKNLSHKKMHMNLVVIKSKGWIVNVSTKILISDQ
AIASYAEDAGAHTQPSESALAVITSRVMAPIVLVVSQKPTAPSKTLFLEALSLYDEVTATVLNKPKKYYIF
EGDVIAILGSIDAAVLTGLQRPQPNRMRRAVVADAIRLAIAL-
AADVIGSLENQIVNFARGVSFVRDSYPTCAAIRTT-------
AFRVRNHKVSTKRARPYKGHTKFLHKADIDKSLIASGVDTLA
>GCA_900110065
AVIKPVVIDDYSPVLMIKQQQVATVSGAGSPTLRAKTVYAKSGLQRKVETKWVAKDFYY-
GVVKMANLVQLVSISVDNRAVCTVAQLVEQEVVVRGMVALVAVKKKIALDAIEGMTADDPAKVVD
Y-VRIALRKFRDADDKSDLLPILLGLTLKIKFDPHQPVVHDLPIPVIFEVI-
GIMAQRGTIEVDDCGISTAVGDVDPIEYVSPIDLAAVAGDKMAYIDQEAAVQQAKDDIALAELALESA
VLVVGIRPLIIPI-FRDEADT-MSFANRSRTLTTIVDMAVSAVTQTRA-----IASTL-
MDRSQAAALADKRGTAEEIRIVKRMQRSAGLADNPELVMLEDAAEGNSNWAGQIYDDDISGSSESD
SAGNIVHAANKRQSFARSITFLRGVEISDQAAAPLMRGVKHNAKSRSSRKLDDAITVLNFANVPEGIAI
AAAADKPADTLLHDATEGERILMIIVRWHVTWKTAIILFLME-
AGDIVGLLTLGGMAGVGRKDGVLNRHHKMNGSLIEQKRIIDKLTLRLRASLKQKLGAEQVIILVVRVQ
GDLVAQAEIQMG--
DIGAVVAIQGPTIFVRRHRQTVIGDQEATVEVLRGVAQGPADAQALLRIRVDHTLVKLSVLLIPEVPVL
VESVTIREGVGKALQIIQLDVYLLRDMGTGEETLAMSRSRELIRELLQIDPITGVRLAFGNLREREAKVLTI
KEIDTLF--
LAIASGITEPRMANLPQGVCRHFVRAGPRAAVGMIFLILDPAGRIYTIDGDLRELPKTIKALISLVDAAKP
KALSAVYATERLKVRYEDVHVLVSDANLYAYAEARSEMLRMVPVYIINAAKRRQIVLLALSLRDGHRR
VRMSIDVMIAAAIIESHADHGPVHHAEPRILLDDAEVVGSEMMISRQAALDLKTLVKIDVIVIFPEFQEK
MVVSLRFFCTDVAIFVIALICTVGEMLSREPAAAQESGAGFQKRIRAHSVLFALSVTFDAANMKR--A-
DLFIGMGLAPPRYGILYTVQPKRKQMPTEPDDEAVDGFDLVHAVKEDGRD-
QKLDLPEAMALAVEATDTINVSGGVLAFDGSMAALEKVDSKRKMALAVVQWTIAPTTIADLRLSGAR
PFRALT-IELADVTAALEHKDVD-
LYLPGANNAEMEADKIDGAGASTVEAAADRQRGILLLTPVLVVIQDSNTAALIANDGLDEWGTMAY
KKHYAFAARMRLHMRTIDGIEVGVQMEPNKQKNEKIKTVKLEGDTRFRQLATEGVKEQMP-
LHALNPDFVV-LAVLVTPAVGRVAADEDVLGAQVPCGASL-
RELQKSQTGAALPISGIRLLGMRAVRAMVDEDIMVAGKVAA-
LLVIVAFETICRLVLLNDSEATLPWRLSVATRLEADAKRAAGFAARPFMSKPSLCDTQAHARIADPIQEI
AAGYVARDDRFVFPLEGLPIQLILSSAHSRVGTAKRAGLGHLREMAKELQAKAVLMVLAASDTNTEGL
LRYATTRQPTDLLHDDCLDIVIDMRRMSVV---

VRLNTV-
HVLVPGVCPLGDYQIMQAAAPSGLVEVEQVDDRTGPERTIMWMYGAMEALWLLSVRYADWVVDC
AIGAIVLHVYAQTINRAFSLDRLQLAGWPPEAASWWNTWQLWLNKDGFFGYDAGFRA-
AAALGADLAGFDMTDVKGVGRHQSRQELRVGYRIREQVAFEKLDPRTDILLQSMTRLSVIRHALEVV
AIMIVREIVIEFT-
VVAQEDVAMGHVECVLADAFDFTAKNADIGPVGLKTPDPVLGDGTVLNDAVLGKIDFANTVANFRY
RMIDDGGRALGVQWLTLANILVGRIWLPPAAAGLKNLQPICFFLGHPELNLDLLELLWRGISKARADA
LMAGALIEKILEKVAAFADPDATTHQSNKVKHFKYYGEEVDPVYLAYIV---
ALVATQVTVSDRLLTDRYVLNLKI-RGLTSLLWLAAQIGNKGPSATIISLDSVAGLGALVLFIILL-
RDRVLLETLYWRYVY--MRGYDAAKDVADADPEHLLNLAAATRDVEWIAVVPAFFYYFRTAG-
FQPFNTAVAREFFGLFRERLRIVAVVAVPPDRVAALVVFVALAGTLPTSAQALIGVIACGLCEPNAAGV
LEADQDWNLHRSMGITLAFYDARESVKRWFDKGLHKPVAATGLSEAGALTTPFHAGQTAKVCVPAI
HRVVDLGELEKSLIELDSAVVVADEEQTLADKPTTRDNSVGAVGGVVLAGTMKMGANLDSVERGNN
IIIELEEEQYSAVNGRKDGMKLTDKVLMVLSQAYFPVLRRMASANALDRGAFDLLIDVPDDADNRDQ
G-GLSIPAPTVHAETAVLFAESDIERIAWIAPLHLLGTLPAVLGFEPDQYSFISMIIEASLDIMETAEV--
DYHVLETGSRPSDFRVILIKGLHVRQLAGIATIIVVGSAGRGEVTAYIQG-
AVPITLRRRTFLPAFAASSCHTIDIFSTMLRPPERSTIIMLILETVTEL-
LAVTRLTNDKLERMLVLIPGLDMALLLDLEAASEDFQQIVTIEEAIVAILSVAAEPVVLREMLLSLAEIAV
DAMALALALLLMLTPDTGLSQKEAAREGQLKVLIQGGAVLQIRAADRMVLAATADKVRLDTDDGTT
SAFASLNLATVSDGFYVLSSAVLQMGSDLMMIIKVARVLADKETKNALVVRHPEQVKTQSTYAEGSP
GMVLQTLTGSIPPLAVKPEDIELLWTLVAALWRVKAHLANATRPHTERVLILILALALITVLVQSEGGGI
GGA--
GPAARILAAFSILTIKAKVGDEVRKVTRRVLDLNSRLGRPEESVNPEFRGAELEQSKFEAANVEVDTMET
IRRFVDIPFLVSTRTQHNESEDRGSRMVLDVTRVRFIDL-
VTKRSEIILAARAETDGIIARQMKGDLAALHPAAAALGKIAALQHFIAISDAHISNYVTQEIHSVLGMVL
LVLFINETRIAKVVWPTRREVTMVLIMATLAIFFIVIGLFVSIVFMLSGISWNQLTTIVLGEGVMAVRQTS
TFPQDS-
IGILQNTYADLIAAGRVAKADIEKSLHIVVAGRVTRGTWYVIYLYEISQAQGEAGIMQRAFRQKTLSQS
LIDGRMEVKIKVVTSDRYA-
TYEIKGGQHRKGTRYNRVKVGHNIPVQVMPRMKMAFTPPNLVLVKTFGAVVQKRFAQALTHMRHG
AVQIGPMEVSSPYINSKKARHAPGDAVVELKPENIVRSVAVVPELIWARENEDNNAVHDKYVKTTHK
LAAKRKKAVDKAQYDHRNQVEMSFIGLAVKVLPDKAIVHATAGMLMPTASVAVTEGMKGIAATIRL
DPMEKALIALIKRMRPQLNLAGKTDKKIVKCLNNNLDDVEYGKLGRRKIKPITIKSVYDVESRRATELVK
LLPYKDDVEGRKSQFAEK--
ALFEPLKAFMMLFGYIARAVYALNLRVIRRTILLAKCKIVVNAATVLSAIKIVGVSIRLRHKSTNVAETNH
HIKTIDKRARMTLAGEGKVKLNLSEGRVGKKGGRIKGYMPYNNNYNIFDGLASGRRAAVQEVAKLKKV
DKQIQVEGIRAKQEGGRTPKAQLSNAMANTRVGVD-
VGEHIQEESNPRANAEMGQIFAAIDARVYNHVGALIGHALEAVLAIVAIWGIGRAHNMLALRLRVNA
GIELHSAA-
VEVAVACAGFATPAGEASLLVDVSEGPLGALVVTAAAVDVCRLRRRSLSELKKTKKDAYMSLTCFAGK
GDRIKIVEATQTQAYEIVATRTAERHGIHAKGTYGKERAMMQILPTKPVKVWVPMFVDEEELLAPIRAF
AETVGDGVTVVKLSTDIKITPFEKAVASLAVSGKNTSVVYTVLEKVTQLMKDIIYTQRAKAHYGRGRLR
SHVVTHTKSRRHLSMMKRTVVADPSMDAARLTDGEVSVVTGATMAFVMSEQDAV-
PGDVKMHFHEEPRREAEVGIAGPETFLATEHPRVLAVVFLRAYLLHIVEIQTVKDFSSAATIYDVLRAM

NSKLDRKLSADIAKTRMVLADNLMAMGVTEVAARREAAGDPILSSYWINGVTANADIVLLVANLEVP
TVPENIHCAIRLFYQRVTLQAKERGGDVSVAVVPANKQIEAAEDTVIYTIKGKAPGSGQMKLTIEGISRS
IVKSFAVQLFQVDTFLDDTWLGTLLRAAADVVFVDAGDGKEAVDHVVERDTEYEFAPQKDIVSRKIRI
YTPAAPSAVVIWDPAIEREDKAEAQGTILKIYGPNLQDINCFTDIGSEPKIESLKAWDEKEKYVVHRDDI
PEDIDTLVGKSSRVNKEFAIPDVAVCAPSKPGVTESIDRGEPKAGTCGEVANMAYPQESLVNNVSSAN
VQKNISMVLAGHPVGEMGISREIERHLETNMCEIFVDKADIDVALDFSVLALTVLRVGLDPPNARVAID
GKESRKLMAVYDGDAPLQRHPKVSAATGGQ--
AGVKATLGDDALEMDGVVHGDISFTLTDLPDGRV-
VTPVYNNVKAQLKIDKVILRAFHVVTLSFSNGKGEDHLIKLRLNALKKNTRHYEMLLKIRETECAESPHA
ISVGKVLKIDALLQTICVAHYKAIEPLLADDLTALGVMGEQGLLHVVPRLMILAVMHLWGLEAALELSR
WGLQAERCVTDQTMGLAVRERHFSY-CISEVVGNTFFAMTKDDDTEQELHARNPI-VLL-
WGKFGMV-
ISPSGLKLVSTSADALDRGNQDDLILSTERLQDAMGYYVLRILLVFVDRVILAYGSGEEPIKFLVADQAV
TGRQNPGMVTLARRTFPLELQRDAVFVMVFADSTMKNLSHRKMHMNLVLIKSKGWVVNVSTKILIS
DQAIASYAEDVGAHTQPSESALAAITSRVMAPIVLVVSQKPSAPSKSLFLEALALYDEVTATVLNAPKK
YYIFEGDVISVLHACDAAVLLGMQRPQPDRMRRAMVADAIRLAIAFRASGIVTRLRQGLINFARAVGL
AGGVYPDFGALVMAIIL-EALAFRVRNHAVSTKRSKPYKGHTKFLHKADVNKSLIAAGVDTIA
>GCA_900110705
AVIKPVTMNAYAPVLMIAQSQVATVSGAGSPSLRAKTVYAKSGLQRKSETKWVAKDFYY-
GVVKMANLVQLVSISVDNRAVCTVAQLVEQEVVVHGMVALDAVKKKIALEAIEGMTAEDPAKVVDY
QTRIAIRKWRDADDRADLLPVLLGLTLKIKFDPHQPLVHDLPIPVVYEVI-
GIMAQKGTIEVDDCGISTAVSDVDPIAYVSPIDLAAVSGQKMDYIDQEAAVAQAKQDIDLEELALESS
VLVVGIKPLIIPI-FRDDADE-MSFANRSRTLKTIVDLAVSYPEQTRAR----IAAAM-
MDASQAAALAEKRGTAEEIRIVKRMQRSAGLADDPELVMLEDAAEGNSNWAGQIYDDPISGSSESD
SAGNIVYAANKRQNFARSITFLRTVEISDQAAAPLMRGVKHNAKSRASRKLDDAIAALNFPNVPEGIAI
RAAAGKATSTLLHDADEGEQILMLIVRWHVTWKTAAIIYILEIAGNIVGLIELGGIAAVGRKDGLLNRHH
KLNGSLIEQKRVIDKLTLRLRAALKQKLGQDQVIILVVRVQGDLVAEAEIQMG--
DVGAVVAIQGPTIFVRRHRQTVIGDQEATV-VLKGVAEGPADAQALLRIRVDHTLVKLSVLLIPE-
PVLVESVTIREGVGRALQIIQLDVYLLRDMGTGEETLAKSRSRELIRELLQIDPITGVRLAFGNLREREAKV
LNIKEIDTLF--
LAIIQGVTEPRMGNLPEGVCRTFVRAGPRAAAGMLFLILDDAGKIYAIDGDLREITKSIKSLIGLVDAAKP
KALSAVYMTDRLKIRYEDVHVLISDADLYAYAEARRELLRMVPVYITNQAKRRQLVLLALSLRDGHRRV
RMAIDVMIAAAIIESQAVNGAVHHAEPRILLDDAEVVGSEMMINRQAALAMKEVLKVDVIVIFPEFQE
KMVVSLRFFCTDVAIFVIALICTVGAMLSREPSHAQESGAGFQK--
RAHSVLFALQATFDAQNLKKADAANLFVGLGISAPRYGILYTVRPKRKQMPSEPDADKVDGFELVHA
VKEDGRDN-KLDMPEAMALAVE-
DATINVSGGVLAMDGDMAAIEKVDSKRKLALAVTKWNIARVSIQDLTISGARPIRAFD-
ITVAKVTAALGEKAVS-
LYLPKANNAELEADKIDGAGSSTVDAAADRQQGILLLNPVLVVIQDSNTAALISNEGLDEWGTMAYK
KHYAYAARMRLHMRTIDGIEVGVAMEPNKQKAEKIKTVKLEGDTRFRQLATEGVKEQMP-
LHALNPDFAV-LAVLVTPAVGRVAADQDVLGARVPCDASL-
RELQKSQKGDALPISGIRLLGMRAARAMVEEDIMVAGKVAASLLVIVAVETICKMVLLNDSEATLPWR
LSVAERIEADTARAAGYAARPFMSKPSLCKVQAHANVADPIQEIAAGYVATEQRFVFPVEGLPIQLILA
SPHSRVGVAKRAGLGHLHEMAKELIGKADLMVQAASDTNTEGILNYLTTRQPTDLLHDDCLDIVIDM

RRMSVV--VQLNTV--
VVVSGVRDVGAYEIMQAAAPSGLIQVERIDDRTGRERTMMYMYGAMEELWLLSVRYADWVVDCAI
GAIVLHVYAQVINRAFSLERLQLAGWPPEASSWWNTWQLWLNKDGFFGYEAAFRAQAAVLSAALY
DYEMSDVEDVGRYPSRQELRVGYRIRAQVAFDRLDPRTDILLQSRTRLSVIRHALEVVAIMIVREIVIEFT
LVVAQEDVAMGHVECVLADCFDFTAKNADIGPVGLKTPDPVLGDGTVLNDAVLGKIDLANTVADFR
YRMIDDGGRQLGVEWLTLRNLLYSRIWLPPAEAALKNLQPICFFLGHPDLNLHLLDALWAGISKARM
QALMASALIEQILDKVAAFADPDATTHQSNKVKHFKYYGEEVDPVYLAYILDMFALVATQVTVSDRLL
KDRYVLNLKV-RGFTSLWWLGAQLATKSPAATIIALDSIAGLGALVLFIILL-RDSVLVETLYYRYVY--
MKGYEAAKDVADADPEHVLNLAAVSRDVEYIEIVPAFFYYFKTAG-
FEPFNTAVAREFFGLFKERLRVIDVLVVPPDRVESLVAFVALAGTLATSAEALPGVIATGLCEPNAASVL
EADQDWNLHRSMGITLAFYDARESVKGWFDKGLHKDVAQTGLSEAGALTTPFHVAVTAKACVPAIH
RVLDLGEIEASLIEIDSAVTVADEEQTLADKPTTRDNSVGAVGGVVLAGTMNMGANLDSVERGNNIII
ELEEESYSAVNGKKDG-
KLTDKVLMVLSGAYFVVMERIASASALDRGAFDLLIDVPDDADNRDQGREL-------------------
------------------------PDQYSFISMMINASLDIMQTAEV--
DYHVLEKGSRPPAFRVILIKGLHVRHLAGIATIIVVGSAGRSEVTAYIQG-
AVPITLKRVTFLPAFAASSCHVIDIFSTMLRPPERSTIKMLILETVAEL-
LEVTRLENDKLERMEVLLPGLDMAQLLPLEAHAADFPQIVTIDASIVAILSVAAEPVVLRDMLLSLAEIA
VEAMALALALLLMLTPDQGLTQKETSREAQMKELIQGGVVLQVRAADRMVLAATADKVRLDTDDG
TTSAFASLNLATVSDGFYVMSSAVLQLSSDLMMIISVARVLADKEHKNALVVRHPEQVKTQSTYAEGS
PGMVLQTLTGAIPPLAIKADEVELLWTLVTALWAVKAHLANATRPRNERVLILILALTLITVLLQSEGGGI
GGG--
GPPAKGLAAFSILTIKAKAGDEVRKVTRRVLDLNSRLGRPEESVNPEFRGAELEQSKYEAANVEVDTME
TIRRFVDIPFLVSTRTQHNESEDRGARMVLDVTRVRFIDL-
VTKKSEIILAARAETDGIIAREMKGDLAALHSEAAALGKIAALQHFIAIQDAHISNYVTANIHSVLGMLLL
VLFINETRIAKVVWPTRREVTMVFIMAALATFFLVIGLVISIVFMLSGISWNQMTTIVLGEGVMAVRQTS
TFPKDS-
IGILQNTFADLICSGRVAKADIEKSLHIVVAGRVTRGTWYVIYLYEISQAQSPAGIMQRAFRQKTLSQSLI
DGRMEVKIKVVTSDRYAQTYEIKGGQHRKGTRYNRVKVGHNIPIDVLPRMKMAFTPPNLVLVKTFGA
VVQKRFASALTHMRHGAVQIGPMEVSSPYIRNKKARHAPGDAVVDLKPELIVRSVAVVPELIWAREN
EDNGAVHDKYVKPTHKLKAKRTTAVDKAQYDHRNQVEMSFIGLAVKVLPQKAIVHATAGMLMPTTS
VAVTEGMKGIAATIRLDPMEKALIAYIKRMRPQLNLEGRTDKKIVKCLNNNLDDVEYGKLGRRKIKPITI
KSVYDVESRRATELVKLLPYKDDVEGRKSQFAEK--
ALFEPLKAFMMLFGYIARSVYALNLRVIRRTILLVKCKIVVAAATVLSAIKIIGLSIRLRHKSTNIADTKHHIK
TIEDRANLTMAGAGKVKLNLSEGKVGKKGGRIKGYMPYNNYNIFDGLASGRRAKVKEVAKLKKVDKEI
KVEGIRVKQEGGRTPKAQLSNAVANTRVGVDIVGEKIQEQSNPRANAEMGQIFAAIDARVYSHVGAL
IGHALEAVLAIVAIWGIGRTHNLLALKLRINAGIEMHSAA-VTHAVACA-
FAAPAGDASALWDASEAYLGAIAITEHAVDTCRLRRRSLSELKKTKKDAYMSLTCFAGKGDRVKIVEAS
QTQAYEIVATRTAERHGIKAKGTFGKERAMMQILPTKPVKVWVPVFVDEVELLAPIRNFAETVGDGVT
VVKLQNDIKITPFEKAVASIAVTGKSTSVVYTVLEKVTELSKDIIYTQRAKAHYGRGRLRSHVVTHTKSSR
HLSMMKRTVVADPSMDAARLTDGEVSVVTGATMAFVMSEQDAV--
GDVDMHFHEEPRREAELGIGGPETFLATEHPRTLAVLFLRAYLIHIVEIETTKDFSNAATIYDVLRAMASK
LDRKLSADIAKTRMVLVDNLMDMGVTEVAAKRDAAGDKILSAFWINGVTANADIVLLVANLEVPTVP
ENIHCAIRLFYQRITLQAKERDGDVSVAVVPANKQIEAAEDTVIYSIKGKTPGNAQMKLTIEGISRSVVK

SLAVQLFQVDTFLDDTWLGTLLRAAIDVVFVDAGETKEAVDHVVERDTEYEFAPQKDIVSRKIRIYTPA
APSAVVIWDPAIDREDKADAQGTILKIYGPNLQDINCFTDIGAEPKIESLKAWDEKEKYVVHRDDIPEDI
-
TLVGKSSKVNKEFAIPDVAVCAPSKPGVTESIDRGEPKAGTCGEVANMAYPQESLVNAVSSANVQKN
ISMVLAGHPVGEMGISREIERHLETNMCEIFVDKADVDVALDFYILSLTPLRVGLDPPNARVSIDGKESR
KLMAVMDGDADLQRHPKVSQATGTG-NAGIKARLPDDTLIVDGVVHGDVSFTATDLPEGRV-
VTPVFNDVKAQLKIDKVILKAFHVVTLSFSNGKGEDHLIKLRLNALKKNTRHYEMLLKIRETECAESPHAI
SVGKVLKIDSLLQTICVAHYKAIEPLLADDLTALGVMGHQGLLHVVPRLMILAVLHLWGMAAAMEVA
QWGLQANA-VTDQTMGLALRERHFTY-SISEVVGNTFFAKTKEDDTEQRLHARNPI-VLL-
AAKFGMV-
ISPSGLKLTSTQASALGRENAEDLILSTERLQDAMGYYVLRILLVFVDEVILAYGSGERPIKFLVGDQAVT
GRQNPGMVTLTRRTFPLDLQRHAVFVMVFEDCTMKNLSHKKMHMNLILIKSKGWIVNVSTKILISDQ
AIASYAEDAGAHTQPSESALAVITSRVMAPIVLVVSQKPTAPSKTLFLEALALYDEVTATVQNKPKKYYI
FEGDVVSILGACDAAVLLGMQRPQPDRMRRAVVADAIRLAIALR-------
LEAGLINFARAVSLAVGVYTRFQAPP-AAMP-
PALAFRVRNHSVSTKRSKPYKGHTKFLHKADINKSLIAAGVDTIA
>GCA_900114485
AVIKPVTIDDYSPVLMIKQQQVATVSGAGSPTLRAKTVYAKSGLQRKVETKWVAADFYY-
GVVKMANLVQLVSISVDNRAVCTVAQLVEQEVVVHGMVALEAVKKKIALEAIEGMTAEDPAKVVDY
-VRIAIRKFRDADDKSDLLPVLLGLTLKIKFDPHQPVVHDLPIPVIFEVI-
GIMAQKGTIEVDDCGISTAVGDVDPIEYVSPIDLAAVAGDKMAYIDQESAVAQAKDDIALEELALESSV
LVVGI-PLILPI-FRDEADT-MSFANRSRTLRTIVDMAVSAVTQTRA-----IASDL-
MGQSQAAALSDKRGTAEEIRIVKRMQRSAGLADDPELVMLEDAAEGNSNWAGQIYDDDISGSSESD
SAGNIVYAANKRQNFARSITFLRTVEISDQAAAPLMRGVKHNAKSRSSRKLDDDIAALNFPNVPEGIAI
AAAAGKPTETLLHDATEGEQILMLIVRWHVTWKTAIILYMMDRAGDVVGLIRLGGVGAVGRKDGML
NRHHKMNGSLIEQKRIIDKLTLRLRAALKQKLGAEMVIILVVRVQGDLVAQAEIQMG--
DIGAVVAIQGPTIFVRRHRQTVIGDQEATVEVLKGVAEGPADAQALLRIRVDHTLVKLSVLLIPEVPVLV
ESVTIREGVGKALQIIQLDVYLLRDMGTGEETLAKSRS------------
ITGVRLAFGDLREREAKVLSIKEIDTLF--
LAIATGLTEPRMANLPEGICRNYVRAGPRAAVGMIFLILDPAGRIYAIDGDLRENTKGIKSLIGLVDAAK
PKALSAVYATDRLKVRYEDVHVLVSDANLYAYAEPRSELMRMVPVYIINAAKRRQIVLLALSLRDGHR
RVRMSIDVMIAAAIIESHADHGMVHHEEPRILLDDAEVVGSEMMISRQAALELKNIIKIDVIVLFPEFQE
KMVVSLRFFCTDVAIFVIALICTVGEMLSREPAAAQESGAGFQKRIRGHSVLFALSVTFDKANVKRA--
-GLFLQLGLAAPRYGILYTVQPKRKQMPSEPDKDEVDGFDLVHAVKEDGRSN-
QLDMPEAMALAVEATETINVSGGVLGFDGDMAAMEKVDSKRKLALAIKKWTIAPISSVDIKISGAAKS
RALG-IETADLTAALSKKDI--
LFLPGGNNAELETDKIDGAGSSTVDAAEDRQQGILLLNPVLVVIQDSNTAALIANDGLDEWGTMAYK
KHYAFAARMRLHMRTIDGIEVGVQMEPNKQKAEKIKTVKLEGDTRFRQLATEGVKEQMP-
LHALNPDFAVLIPVLVTPAVGRVAADQDVLGAHVPCGASL-
RELQKSQTGEVLPISGIRVLGMRAARAMVDEDIMVAGKVAA-
LLVIVATETICKLVLLNDSEATLPWRLSVATRLEADTKRAAGFASRPFMSKPSLCNTQAHARIADPIQEI
AAGYVAREERFVFPVEGLPIQLILSSPHSRVGGAKRAGLGHLHEMAKELQGKAVLMVLAASDTNTEGL
LRYATTRQPTDLLHDDCLDIVIDMRRMSVV---
VQLNTV-

HVVIKGATPVGDFEIMQAAAASGLVEVEQVDDRTGRERTMMWMYGAMEELWLMSVKYADWIVN
CAIGAIVLHVYAQTINRAFSLERLQLAGWPPEASSWWNTWQLWLNKDGFFGYPAAFRAVAAAFEA
QLYDFDMRDVEGVGRHLSRQELRVGYRIREQVAFAKLDPRTDIMLQSMTRLSVIRHALEVVAIMIVREI
VIEFTLVVAQEDVAMGHVECVLADQFDFTAKNADIGPVGLKTPDPVLGDGTVLNDAVLGKIDLSNTI
ADFRYRMIDDGGRELGVEWLTLQNILYARIWLPPAAAALKNLQPICFFLGHPDLNLDLLDQLWAGISK
ARAAALMAGALIEKILEKVATFADPDATTHQSNKIKHFKYYGEEVDPVYLAYILDLFALVATQVTVSDR
LLTDRYVLNLKIFRGFTSLLWLAAQIGEKGPSATIISLDSVAGLGALVLFIILL-
RDSILVETLYYRYVYVVMKGYEAAKDVADADPEHLLNLAGATRDVEYIEVIPAFFYYFKTAT-
FQPFNTAVAREFFGLFAERLRIVEVLAVPPDRVLDLVDFVALAGTLPTSAQALVGVIACGLCEPNAAGV
LEADQDWNLHRSMGITLAFYDARESVKKWFDKGLHKPVAQTGLSEAGALTTPFHVAVTAKVCVPAI
HRVLDLGEIDGSLIELDSAVVVADEEQTLADKPTTRDNSVGAVGGVVLAGTMNMGANLDSVERGN
NIIIELEEEGYATVNGKKDGMKLTDKVLMVLSEAYFPVLQRAASANALDRGAFDLLIDVPSDADNRDQ
GDRL---PDQYSFISMMIEASLDIMETAEV--
DYHVLETGSRPPAFRVILIKGLHIRQLAGIAAIIVVGSAGRSEVTAYIQG-
AVPITLRRKTFLPAFAASSCHVIDIFSTMLRPPERSTIQMLILETVAEL-
LAVTRGTNDKLERMQVLIPGLDLAELLALEAAAADFPQIVTIEAAIVAILSVAAEPVVLREMLLTLAEIAV
DAMALALALLLMLTPDQGLTQREQAREAQMKELIQGGVVLQVKAADLMVLAATADKVRLDTDDGT
TSAFASLNLATVSDGFYVMSSAVLQMGSDLMMIIKVARVLADKETKNALVVRHPEQVKTQTTYAEGS
PGMVLQTLTGSIPPLAIKAEEIELLWTLVAALWRVKAHLANATRGHTERVLILILALSLITVLLQSEGGGI
GGG--
GPAAKILAGFSILTIKAKVGDEVRKVTRRVLDLNSRLGRPEESVNPEFRGAELEQSKYEAENVEVDTMET
IRRFVDIPFLVSTRTQHNESEDRGSRMVLDVTRVRFIDL-
VTKRSEIILAARAETDGIIAREMSKDLAALHPEAAALGKIAFLQHFIAISDAHIANYVTQEINSVLGMLLL
VLFINETRIAKVVWPTRREVTMVFIMAALATFFIVIGLFVSIVFMLSGISWNQMTTIVLGEGVMAVRQTS
TFPADS-
IGILQNTYADLICAGRVAKADIEKSLHIVVAGRVTRGTWYVIYLYELSQAQGESGIMQRAFRQKTLSQS
LIDGRMEVKIKVVTSDRYA-
TYEIKGGQHRKGTRYNRVKVGHNIPIAVLPRMKMAFTPPNLVLVKTFGAVVQKRFAQALTHMRHGA
VAIGPMEVSSPYINSKKARHAPGDAVVDLKPENIVRSVAVVPELIWARENEDNGAVHDKYVKTTHKL
AAKRKKAVDKAQYDHRNQVEMSFIGLAVKVLPAKA------------------------------------
-----
RMRPQLNLAGKTDKKIVKCLNNNLDDVEYGKLGRRKIKPITIKSVYDVESRRATELVKLLPYKDDVEGR
KSQFAEK--
ALFEPLKAFMMLFGYIARAVYALNLRVIRRTILLAKCKIVVAAGTVLSAIKIVGVSIRLRHKTTNVAETNH
HIKTIDKRARMTLAGTGKVKLNLSEGRVGKKGGRIKGYMPYNNNYNIFDGLASGRRAKVKEVAKLKKVD
KEIKVEGIRAKQEGGRTPKAQLSNSLANTRVGVD-
VGENVQEESNPRANAEMGQVFAAIDARVYHHVGALIGHALEAVLAIVAIWGIGRAHNLLALKLRINA
GIELHSAA-VEVAVACA-FAAAAGEASLMVDVSEYPLNMATISAASVDICRLRRRSLSE-----------
----
RHGIHAKGTYGKERAMMQILPTKPVKVWVPVFVDEVELLAPIRAFAETVGDGVTVVKLTTDVKITPFEK
AVASLAVAGKNTSVIYTVLEKVTQLMKDIIYTQRAKAHYGRGRLRSHVVTHTKSRRHLSMMKRTVVA
DPSMDAARLTDGEVSVITGATMAFVMSEQDAV--
GDVDMHFHEEPRREADVGIAGPETFLATEHPRTLAVIFLRAYLLHIVEIQTVKDFSNAATIYDVLRAMN
NKLDRKLSADIAKTRMVLVDNLMEMGVTEVAAKRDAAGDKILSAFWINGVTANADIVLLVANLEVPT

VPENIHCAIRLFYQRVTLQAKERDGDVSVAVVPANKQIEAAEDTVIYSIKGKLPGSAQMKLTVEGISRSI
VKSFAVQLFQVDTFLEDTWLGTLLRAAADVVFVDAGDGKEAVDHVVERDTDYEFAPQKDIVSRKIRIY
TPAAPSAVVIWDPAIEREDKAEAQGTILKIYGPNLQDINCFTDIGSEPKIESLKAWDEKEKYVVHRDDIP
EDIDTLVGKSSKVNKEFAIPDVAVCAPSKPGVTESIDRGEPKAGTCGEVANMAYPQESLVNAVSSANV
QKNISMVLAGHPVGEMGISREIERHLETNMCEIFVDKDDIDVALDFSTVALTVLRVGLDPPNARVEIG
GKESRKLMAVYDGDAPLQRHPKVSAATGTGI-AGVKATLDDDALELDGVVHGDISFTVTDLPDGRV-
VTPVFNEVKAQLKIDKVILRAFHVVTLSFSNGKGEDHLIKLRLNALKKNTRHYEMLLKIRETECAESPHAI
AVGKVLKIDALLQTICKAHYKAIEPLLAEDLTALGVMGDQGLLHVVPRLMILAVMHLWGLAAVMELP
RW-LQEQA-VTNQTMGLSERARHFSY-CISEVVGNTFFAMTKDDDTEQELHARNPI-VLL-
CGKFGMV-
ISPSGLKLASTAADPLGRGNKEDLILSTERLQDTMGYYVLKILLVFVDRIILAYGSGEDPIKFLVGDQAVT
GRQNPGMVTLARRTFPLELQRDAVFVMVFADSTMKNLSHRKMHMNLILIKSKGWVVNVSTKILISD
QAIASYAEDVGAHTQPSESALAAITSRVMAPIVLVVSQKPTAPSKSLFLEALALYDEVTATVLNAPKKY
YIFEGDVISVLNKCDAAVLFGMQRPQPDRMRRAMVADAIRLAIALRASGIITRLEEGLINFARAVGLAA
GIYPEFGAVVMAIIMPEALAFRVRNHSVSTKRSKPYKGHTKFLHKADVNKSLIAAGVDTIA
>GCA_900114675
AVIKPVTMTQYAPVLMIRQQQVATVSGAGSPSLRAKTVYRKSGLQRKTETKWVSKDFYY-
GVVKMGNLVQLVSISVDARSVCTVAQLVDQEVVVHGMVALEAVKRKIALEEIQGMTAEDPAKVVDF
ASEIELRKFRDAEDRADLLPVLLGLTLKIKFNPHEPLVHDLPVPVVFEVI-
GIMVQRGQIEVDECGISTALSDVDPIEYVSPIDLEAVAGDKMAYIDQETTVAAVKAEIELEELALESSVL
VVGI-PLLIPV-FAEDGDV-MSFANRSRDLKTIVNLAVSYPEVQRARLDSA--AAM-
MERSQAAALAEKRGTAEEIRVVKRMQRSSGLADDPELVMLEDAAEGNSNWAGQIYDDPISGSSESD
SAGNIVYAANKRQNFARSITYLRAVEISDQAAAPLMRGVKHNSKSRSSRKVDDAITALNFSNVPEGIAI
RAAAGKSRQVLLHDAAEGEEILLIIVRWHVTWRTAIIISLFVTANNVVGIIELGGIGAVGRKDGLLNRHH
KLNGSLIEQRRVVEKLTLRLRAALKQKIGAEQIIIFVVKVAGDLVANAKVQM---
SVGTVVAIQGETIFVRRHRQTVISDQEATVEVLKGVAEGPADAQALLRIRVDHTVVKLSVLLIPEVPVLT
DSVSIREGVGKALQIIQLDVYLLRDMGTGEETLAKSRSREMIREVLQVDEITGVRLAFGQLTERQVKGG
TMKELGTLL--
ISILDDITEPRMANLPEGVCRTFINAGPRAAVGMIFLLLDEAGKIYAIDGDLRENTKSIKSMIGLVDAAKP
KTLSAGYATARLKVRFADVHVLESDADLYAYAEERKEVLRMVPVYITTEAKRRQLIIFALSLKGGHRRAR
AALGVMIAAAILESQASQGDVNHAEPNILLDDGEIVGSEMMISRQAALDLKQVIKTDVIVIFPEFQEK
MVVSLKFFCTEVAIFVIALICTVGEMLSREPSHAQEGGAGFQKRIRGHSVLFALSVTFDRRNLRDA---
NLFLGLGLAAPRYGIFYTEQPKRKQMPSEEDKDKVDGFDLVNAIKEDATKNHELDMPEAMALAVEAD
GTINVTAGVLALSGDMADVEKVDSKRKMALAVTKWTIAKTTIVDLHISGAHPFRCLS-
IEQADVSAALPAKSI--
LYLPGANNAEMEEEKLDGAGASTADAAEDKQKGILLVIPVLVVIQDSNTAGLIANGGLSEWGTMAYK
KHYALAARMRLHMRTID-----VALEPNKQKAEKIKTVKLEGDTRFRQLATEGVKEQMP-
LHALNPDFAVRVPVLVTPAVGRVAADESVLGARVPCDASL-
RELQKSMKGDALPISGIRVLGMRAARAMVDEDIMVAGKVAA-
LLVIVAIETITHIVLMNDSEATLPWRLSVAERLEADTKRAGGFAARPFKSKPSLCKTQAHAKIADPIKEIA
AGYVAIEERFVFPVEGIPIQLILASPHSRVGSAKRAGLGHLHEMAKELQAKAVIMVLSEPDTNTEGLLNY
LTTQQPTDLLFEDCLDVVIDMRRMSVV---
VQLTTV-
HVVVAGVRDVGSYEIMAAAAHSGLVQVERVDDRTGRERTLMWMYGAMEELWLMSVTYADWVTD

CAIGAILLHVYGQQINRAFSLERLQLAGWPKEATRWWNTWQLWLNKDGFFSYEAAFRAVKAVLKAE
LDDYEMSDVEGVGRYQSRQQLRVGYRIRQQVEYEQLDPRTDILLQSLTRLSVIRHALTMVAILIVREVV
VEFT-
VVAMEDVAKGHVECVLADVFDFTAKDADIGPVGIRTPDPVLGDGTVLKEAVLGKIDLANTVADFRYK
MLIDGGRELGVSWLSV-
NLIYERIWVPPAQAALKNLVPIAFFLGHTELNLDIFAALWAGISKARANKLMAGALIECIIENVAAFADP
EATTHQSKKLRHFKYYGEEVDPVFLAYILDMFSLVATQVTVSDRLLKDRYVLNLKIFRGFTSLLWLSAQI
GDKGPAATVITVHTIMGLGALVLFIILL-
RAVVLVETLYYKYVYVVMLGYKAAKDVADADPEHFLSLAHASRDVEWIEIIPAFFYYFDTATSRLSKNT
ALAKEFFGLFEERLRVIDVLAVPPERVEALVDFVALAGTLATSAEATEGVIACDFCEPNAAAVLEADQD
WNLHRSMGITLAFYDARESVKGWFDKGLHKDVAATGLREASAFTTPFHA-
DTAKQCVPVVHRVLDLGEVEAALIEIESAVVVADEEQTLADKPTTRENSVGAVGGVVLAGTMNMGS
NLEAVERGGNIIIELEEEQYSAVNGKKADGKVTDKVLMVLSEAYFPVMKRVASANALDKGAFGLLIDA
PDDADNRDHGRELTIPAPTVHTESAPLFAESEIERIAWIAPLHLLGTLDAVLGFEPDQYSFISMMIEASL
EIMETAEVLDDYHVLEKGARPPGFRVILIKGLHIRLLAGICTIIVVGSAGRTEVTAYIQG-
AVPITLNKSTFLPAFAASSCHVIDIFSTMLRPPERSAIEMLILETVTEL-
LEVTRIENDKLERLDALLPGLDMAELLELVAAAADFADIVTIDKAITAILSVAAEAVVLREMLLSLAEIAVT
ALALALALLLMLTPDQGLHQKEESREDQMKLLIQGGVVLQVKANERMVLASTADKVRLDTDEGTTSA
FASLNLATVSDGFYVLSSAVLQMSSDLMMIVAVARVLADKESKNALVVRHPEQVKTQATYAEGSPG
MVLQTLTTAVPPLAIKPEEVELLWTLVAALWRVKAHLANSTRPIQERVLILILALSLITVLLQSEGGGMG
GG--
GPAAKALAAFSILTIKAKAGDEVRKVARRVMDLNSRLGRPEEAVNPEFRGAELEQTKYEAENVEVDTM
ETIRRFVDIPFLVSTRTQHNESEDRGARMVLDVTRVRFIDL-
VTKRSEIILAARAETDGIIAREMSKDLAALQSEAAALGKIAALQHFIAVQDGHISNYVTADIHAVLGMLL
LVLFIQEVRVAKVVWPTRRETTMVFIMATLAIFFLILGLGVQLVFMLSGISWQQLTTIVLGEGIMVVRQT
STFPEDS-
IGILQNTYADLICAGRVAKADIENSLHIVVAGRVARGTWYVIYHYDITQAQDKSGIMQRAFRQMTLSQ
SLIDGRMEVKIKVVTSDRYA-
TYEIKGGQHRKGTRYNRVKVGHNIPVQVLPRMKMAFGPPNLVLVKTFGAVVQKRFAQPLTHIRHGA
VTIGPMEVSSPYIKNKKARHAPGDSVVDLKPDNIVRSVAVVPELIWARENEDNGAVHDKYVKTTHKL
AAKRKKAVDKAQYDHRNQVELSFIGLAVKVLPEKAIVHATSGTLMPTASVAVTEGMKGIAATIRLDPM
EKALIAYIKRMRPQLNLGGQNDKKVVKCLNNNLDDVEYGKLGRRKIKPITIKSVYDVEHRRATELVKLM
PYKDDVETRKSQFAEK--
ALFEPLKAFMMLFGFIARAIYALHLRVIRRTILLVKTKIVVNASTVLSAIKIIGVSIRLRHKSTNVADTRHHIK
TIEARARMTLAGAGKVKLNLREGKIGKKGGRIKGYMPYKNYNIFDGLASGRRAKVKELAKLKDVDKTIS
VDGIRQKQQGGRTPQAELSNAI-
NTRVGVDIVGENEQEQSNPRANAEMGQIFAAIDARVFSHVGALSGHALAAVLAVVAIWDIGKAHNL
LAVKLKINAGIEMHSAA-VDAAVAAAAFATNAGEA-
TLADISESYIGQLALTEASVDVCRLRRRGLQELKKTKKDAYMSLTCFSGKGSKVKIVEATQTQAYEIVAT
RTAERHGIRAKGSYGKERAMMQILPTKPVKVWVPVFVNEDELLAPIRAFAESQGDGVTVVKLTTDIKV
TMFEKAVASIAVSGKATTVVYTVLEKVTELMKDIIYTQRAKAHYGRGRLRSHVVTTTKQRRHLSMIKRT
VIADPSMDAARLTDGEVSVVTGATMAFVMSEQEAV--
GDVDLHFHEEPRREAEVGIGGPETFLTTEQPRVLALIFLREYLIHIVEIGTVNGFSDAATIYEVLRAMQNK
LDRKLSADIAKIRMVLADNVTDMGVTEVAPRREQAGDKILSAFWINGVPANADIILLVANLEAPCAPE

NIDCAIRLFYQRITLQAKERGGDVSVAVVPASNQIEAAEGTVIYSIKGQTPGTGQMKLNIEGISRSIVKSL
AVQLFQVDLFLDDTWIATWLRAALDVVFVAAEDGKEAVDHVVERDTELEFAPQKDIVARKIRIYTPAA
PSAVVIWDPAIDREDKAEAQGTILKIYGPNLQDINCFTDIGAEPKIESLKAWDEKEKYVVHRDDIPEDID
TLVGKSSKVNKEFAIPDVAVCAPSKPGVTESIDRGEPKAGTCGEVANMSYPQESLVNAVSSANVQKNI
SMVLSGHPVGEMGISREIERHLETNMCEIFVDKSDVDVALDFYIVVLTALRVGLDPPNARVTIDGKESR
KLMAVEDGDADLQRHPKVSAAAGTG-KAGTKARMEEDTLIVDGVMHGDLSFTVTDLPEGRV-
VTPVFNAVKAQLKIDKVILKAFHVVTLSFSNDKGEEHLVKLRLNALKKNTRHYEMLLKIRETECAESPHA
ISVGKVLKIDALLQTICQAHYKAIEPLLSDELTALGVMGEQGLLHIVPRLMILAVLHL-
PLEIALQLRHWQLQAAAAVTDQTMGLAMRDREWSY-AISEVVSNTFYARTKEDDTEQRIRARNPV-
VLL-LGKFEMI-
ISPSGLKLQSTLASDMDQGNAEDLILSTERLQDAMEYYVLDILLVFVDEVILAYGSGEEPIKFLVADQAV
TGRQNPGMVTLTRRTFPLELQRDATFVMVFEDSTMKNLSHKKMHMNLVVIKAKGWIVNVSTKILISD
QAIASYAEDAGAHTQPSESALAVITSRVMAPIVLVVSQKPTAPSKTLFLEALSLYDEVTATVQNKPKKY
YIFEGDVVSVLQKLDAAVLLGMQRPQPDRMRRAVVADAIRLAIALKASGIVTRLQSGLINFSRAVNFT
AGYYPFTSA-----------EFRVRNHDVSTKRSRPYKAHPKFLHKADIDKGLVATGVDTLA
>GCA_900115105
AVIKPVTMNTYAPVLMIAQSQVATVSGAGSPTLRAKTVYAKSGLQRKAETKWVSKDFYY-
GVVKMGNLVQLVSISVDNRAVCTVAQLVDQEVVVRGMVALDAVKKKIALEAITGMTAEDPAKVMD
Y--EIELKKFRDADDRADMLPVMLGLTLKIKFDPHQPLVHDLAIPVVFEVV-
GVMVQKGTMEVDDCGVSTALSDVDPIVYVSPIDLAAVAGDAMAYIDQEAAVAAAKASIDLD-
LALESNVLVVGI-PLLIPV-FEADGDT-MSFANRSRTLKTIVNLAVSYPAIKRA-----IAAAM-
MDASQAAALAEKKGTAEEIRIVKRMQRSAGLADDPDLVMLEDAAEGNSNWAGQIYDDSLSGSSESD
SAGNIVFAANKRQNFARSITYLRFVEISDQAAAPLMRGVKHNAKSRAARKLDDAIAALNFANVPEGIA
IRAEAGKSVDLLLHDATEREAILLIVVRWHVTWKTAIIVSLFPIAGDVVGLIDVGGIGAVGRKDGLLNRH
HKLNGSLIEQRRITEKLTLRLRAALKQKLGQDQIIIFVVKVAGDLVASATVQM---
AVGAVVAIQGPTIFVRRSRQTVIGDQEATVEILQGVAEGPADAEALLRIRVDHTIVKLSVLLIPQ-
PVLVESVTIREGVGKALQIIQLDVYLLRDMGTGEETLAKSRSREQFREVLQVDPITGVRLAFGDLRERLV
KGGTMKELGTLL--
ISILQGVTEPRMANLPAGVCRTFINAGPRAAVGMLFLLLDDAGKIYAIDGDLRENTKSIKSLIGLVDAAK
PKILSAAYATARLKIRYADVHVLISDADLYAYAEERREILRMVPVYIVNAAKRRQLILFALSLKGGHRRAR
AALGIMIAAAILESQAEQGAVNHEEPAVLLDDAEIVGSEMMISRQAALELKQVFKIDIIVVFPEFQEKM
VVSLKFFCTDVAIFVIALICTVGQMLSREPSHAQEGGAGFQKRIRAHSVLFALAATFDRRNLRSADVPG
LFLGLGLAPPRYGIFYTEQPKRKQMPSEADAEKVDGFDLVNAIKEDATSNHELDMPEAMALAVE-
DATINVNAGVLALTGDMAAVEKVDSKRKMAVAVEKWTISPVELIDIKISGAQPFRALR-
IEQADVSASLPNKEV-
FLYLPGANNAELEEEKLDGAGASTAEAALDKQSAILLVNPVLVVIQDSNSAALIANEGLEEWGTMAYK
KHYALAARMRLHMRTID----GVAMEPNKQKAEKIKTVKLEGDTRFRQLATEGVKEQMP-
LHALNPDFAVLIPVLVTPAVGRVAADAGVLGAEVPCDASL-
RELQKSQKGAALPISGIRVLGMRTARAMVDEDIMVAGKVAA-
LLVILALETITAIVLMNDSEATLPWRLSVAERLEADTQRSAGYASRPFNSKPSLCKTQAHAKIADPIQEIA
AGYVATEQRFVFPVEDIPIQLILASTHSRVGSAKRAGTGHLHEMAKELQAKAVIMVQNEPATNTDGLL
NYLTTQQPTDLLFEDCLDVVIDMRRMSVV---
VHLNTV--
VVVDGVSPVGSYEIMAAAAHSGLVQVERIDERTGRERTLMWMYGAMEELWLLSVTYADWVINCAIG

AILLHVYAQQINRAFSLERLQLAGWPPEASRWWNTWQLWLNKDGFFSYDAAFRA-
KAVLKADLDDYEMSDVEGVGRYQSRQEIRVGYRIREQVAFEQLDPRTDILLQSKTRLSVIRHALKMVAI
LIVREIVVEFTMVVAQEDVAMGHVECVLADSFDFTAKDADIGPVGLRTPDPVLGDGTVLKEAVLGKID
LANTVADFRYKMLVDGGRDLGVSWITLQNMLYPRIWLPPAQADLKDLLPICFFLQHPQLNLEMD-
VIWQ-
ISKARANKLMAGALIEKILEKIAAFADPEATTHQSKKLRHFKYYGEEVDPVYLAYILDVFALVATQVTVS
DRLLKDRYVLNLKVFRGFTSLLWLSAQIGDKGPCATIITVQTIAGLGALVLFIILL-
RAVVLVETLYYKYVY--
MLGYQAAKDVADADPEHFLSLAHASRDVEWIEVIPAFFYYFKTATKNLTKNTALAKEFFGLFEERLKIID
VLAVPPDRVEALVEFVALAGTLTTSATALIGVIACGFCSPNAAAVLEADQDWNLHRSMGITLAFYKAQ
VSVKKWFDKGLHKDVAEIKLNEAGALTTPFHAGSTAKLCVPAIHRVLDLGDVEAALIEIDSAVVVADEE
QTLADKPTTRENSVGAVGGVVLAGTMNMGANLDSVERGGNIIIELEEENYNAVNGKKANGKLTDKV
LMVLSRAYFPVLKRMASANALDRGAFGLLIDAPDDADNQDHGRSLTIPAPTVHAESAPLFAESDIERI
AWIAPIHLLGTLDAVLGFEPDQYSFISMMIEASLDIMETAEVLDDYHVLEKGSRPPSFRVILIKGLHIRQL
AGICTIIVVGSAGRSEVTAYIQD-
AVPITLNKRTFLPAFAASSCHVIDILSTMLRPPERSTIEMLILETVAEL-
LEVTRIENDKLERMDALLPGLDVAALLGLLAAANDFADIVTIDAAIAAILSVAAEAVVLREMLLTLAEIAV
TALALAMALLLMLTRVQGLHQREAAREAQMKALIQGGTVLQVKATDRMVLASTADKVRLATDEGTT
SAFASLNLATVSDGFYVMSSAVLQMSSDLMMIVAVARVLADKETKNALVVRHPEQVKTQSTYAEGS
PGMVLQTLTAAIPPLAIKAEEVELLWTLVAALWRVKAHLANAARPHTERVLILILALSLITVLLQSEGGG
MGGG--
GPAAKILAAFSILTIKAKAGEEVRKVTRRVMDLNSRLGRPEEAVNPEFRGAELEQTKYEAESVEVDTMET
IRRFVDIPFLVSTRTQHNESEDRGARMVLDVTRVRFIDL-
VTKRSEIILAARVETDGIIAREMGKDLAALHPEAAALGKIAALQHFIAIQDAHISNYVTAEIHAVLGMLLL
VLFFQEVRVSKVVWPTRREVTMVFILAAVALFFLVIGL--
QIVFMLSGISWNQLTTIVLGEGIMLVRQTGTFPADS-
IGILQNTYADLVCSGRVAKADIEKSLHIVVGGRVARGTWYVIYHYDISQAQGQSGIMQRAFRQMTLS
KSLIDGRMEVKIKVVTSDRYA-
TYEIKGGQHRKGTRYNRVKIGHNIPVQVLPRMKMAFGDPSLVLVKTFGAVVQKRFAQALTHMRHGA
VTIGPMEVSSPYISNKKARHAPGDSVVDLKPDNIVRSVAVVPELIWARENEDNGAVHDKYVKTTHKL
AAKRKKAVEKAQYDHRNQVELSFIGLAVKVLPEKAIVHATAGQLMPTASVAVTEGMKGIAATIRLDP
MEKSLIAYIKRMRPQLNLAGQTDKKIVKCLNNNLDDVEYGKLGRRKIKPITIKSVYDVEHRRATELVKL
MPYKDDVEGRKSQFAEK--
ALFEPLKAFMMLFGFIARSVYALYLRVIRRTILLVKTKIVVNAATVLSAIKIIGVSIRLRHKSTNVAETNHHI
KTIEKRARMTLAGTGKVKLNLREGKVGMKAGRIKGFMPYKNFNIFDGLASGRRAKVAELAKLKDVDKT
ISVDGVRVKQQGGRTPKAQLSNAI-
NTRVGVDIVGEDEQEASNPRANAEMGQIFAKIDARVFSHVGALKGHALEAVLAVVAIWGIGWAHNL
LAVRLKINAGIEMHSAA-VDAAVAAA-FEQPAGEA-
AIADVSEGDLGALALTTTSVDVCRLRRRSLSELKKTKKEAYMSLTCFGGKGDKVKIVEATQTQAYEIVAT
RTAERHGIRAKGTYGKERAMMQILPTKPVKIWVPVFVDEVELLAPIRAFAETQGDGVTVVKLTKDVKV
TMFEKAVASLAVTGKATSVVYTVLEKVTELMKDIIYTQRAKAHYGRGRLRSHVVTTTKQRRHLSMMK
RTVIADPSMKAARLTDGEVTVITGATMAFVMSEQDAV--
GDVDLHFHEEPRREAEVGIGGPETFLTTDQPRVLALLALKAYLLHIVEIETTNGFSDAATIYDVLRAMQ
NKLDRKLSADIAKTRMVLTDNVTDMGVTEVAARRNAAGDKILSAFWINGVTANADIILLVANLECPC

VPENIDCAIRLFYQRITLQAKERGGDVSVAVVPAKNQIEAAEGTVIYSIKGKTPGTAQMKLTIEGISRSV
VKSLAVQLFQVDTFLNDTWIATWLRAAQEVVFVASEDGKEAVDHVVERDTELEFAPQKDIVSRKIRIY
TPAAPSAVVIWDPAIDREDKAEAQGTILKIYGPNLQDINCFTDIGAEPKIESLKAWDEKEKYVVHRDDIP
EDIDTLVGKSSKVNKEFGIPDVAVCAPSKPGVTESIDRGEPKAGTCGEVANMAYPQESLVNAVSSANV
QKNISMVLTGHPVGEMGISREIERHLETNMCEIFVDKADVDVALDFHILALTPLRVGLDQPNARVQID
GKESRKLMAIQDGAAALQRHPKVSAVTGTG-KAGIKARMPDDALILDGVMHGDITFTATDLPEGRV-
VTPVFNEVKAQLAIDKVILRGFHVVTLSFSNDKGEEHLIKLRLNALKKNTRHYEMLLKIRETECAESPHAI
SVGKVLKIDKLLQTICKAHYTAIEPLLADDLTALGVMGQQGLLHVVPRLMILAVMHMARLEIALQIAG
WRLQAQAILTDQTMGLAFRARDWSF-AISEVVGNTFFARTKEDDTEQRIHARNPV-VLL-LGKFAMI-
VSPSGLGLTSTATSPQDRGNAEELILSTERLQDDMGYYVLQILLVFVDMVILAYGSGEDPIKFLVAEQA
VTGKQNPGMVTLTR-
TFPLELQRNATFVLVLEDCTMKNLSHKKMHMNLIIIKSKGWVVNVSTKILISDQAIASYAEDAGAHTQ
PSESALAAITSRVMAPIVLVVSQKPTAPSKTLFLEALSLYDEVTSTVLNKPKKYYIFEGDVVAVLGTLDAA
VILGMQRPQPKRMRRAVVAQAIRLAIAIMASGVITRLEQGLINFARAVSLAVGVYPTLDMLPAAIIT-
DALEFRVRNHDVSTKRSRPYKAHTKFLHKAEIDKSLIASGVDTLA
>GCA_900128995
AVIKPVTIDDYSPVLMIKQQQVATVSGAGSPTLRAKTVYAKSGLQRKSETKWVAKDFYY-
GVVKMAKLVQLVSISVDNRAVCTVAQLVEQEVVVRGMVALVAVKKKIALDAIEGMTAEDPAKVVDY
NVRIAIRKFRDADDKADLLPVLLGLTLKIKFDPHQPVVHDLPVPVIFEVI-
GIMAQKGTIEVDDCAISTAVGDVDPINYVSPIDLAAVAGDKMAYIDQEAAVAKAKEDIALEELALESN
VLVVGI-PLIIPI-FRDEADE-MSFANRSRTLRTIVDMAVSAVSQTRA----AIASAL-
MAESQAAALADKRGTAEEIRIVKRMQRSAGLADDPELVVLEDAAEGNSNWAGQIYDDPISGSSESDS
AGNIVHAANKRQNFARSITFLRSVEISDQAAAPLMRGVKHNAKSRSSRKLDDAIAALNFPNVPEGIAI
AAAADKPADTLLHEATEGEQILMLIVRWHVTWKTAIVLYLMD-
AGDIVGLITLGGMAGVGRKEGILNRHHKMNGSLIEQKRIIDKLTLRLRASLKQKLGAEQVIILVVRVQG
DLVAQAEIQMG--DIGAVVAIQGPTIFVRRHRQTVIGDQEATV-
VLRGVAQGPADAQALLRIRVDHTLVKLSVLLIPEVPVLVESVTIREGVGKALQIIQLDVYLLRDMGTGEE
TLAMSKSRELIRELLQIDPITGVRLAFGDLREREAKVLNIKEIDTLF--
LAIALDLTEPRMANLPEGVCRDFVRAGPRAAVGMLFLILDPAGRIYAIDGDLREVTKGIKSLIGLVDAAK
PKALSAVYSTERLKVRYEDVRVLTSDADLYAYAEARSELLRMVPVYIVNAAKRRQIVLLALSLRGGHRR
VRMSIDVMIAAAIIESHADHGTVHHAEPRILLDDAEVVGSEMMISRQAALDLKQLIKTDVIVLFPEFQE
KMVVSLRFFCTDVAIFVIALICTVGAMLSREPATAQESGAGFQKRIRAHSVLFALAVTFDKANLRRA---
GLFLNLGLAPLRYGILYTVQPKRKQMPTEPDKDDLDGFDLVHAVKEDGRD-
QKLDMPEAMALAVEATETINVSGGVLGFDGSMAAVEKVDSKRKMALAVKAWTIAPADVADLSISGA
DKFRALK-
IEQADLTAALLHKNLWFLFLPGANNAELEAEKIDGAGSSTVEAAADRNRGILLLNPVLVVIQDSNTAAL
IANDGLDEWGTMAYKKHYAFAARMRLHMRTIDGIEVGVQMEPNKQKNEKIKTVKLEGDTRFRQLAT
EGVKEQMP-LHALNPDFAVLIPVLVTPAVGRVAADQGVLGARVPCGASL-
RELQKSQTGDALPISGIRLLGMRAARAMVEEDIMVAGKVAA-
LLVIVAMETICKLVLLNDSEATLPWRLSVATRLEADTKRAAGFAARPFMSKPSLCNTQAHARIADPIQEI
AAGYIAREDRFVFPVEGLPIQLILSSPHSRVGGAKRAGLGHLHEMAKELQGKAVLMVLAASNTNTEGL
LRYATTRQPTDLLHDDCLDIVIDMRRMSVV--
VRLNTV-
HVLVEGVRDVGDYEIMQAAAASGLVEVEQVDDRTGRERTIMWMYGAMEELWLMSVRYADWIVDC

TIGAIVLHVYAQTINRAFSLERLQLAGWPPEASSWWNTWQLWLNKDGFFGYDAAFRAVAAVLKAEL
YDYDMRDTDGVGRYQSRQELRVGYRIREQVDFAKLDPRTDILLQSMTRLSVIRHALEVVAIMIVREIVI
EFTLVVAQEDVAMGHVECVLADAFNFTAKDADIGPVGLKTPDPVLGDGTVLNDAVLGKIDFANTVA
DFRYRMIDDGGRELGVEWLTLQNLLYARIWLPPAAAALKNLQPICFFLGHPDLNLALLEALWAGISKA
RAAALMAGALIEKILEKVAAFADPDATTHQSNKAKHFKYYGEEVDPVYLAYILAMFALVATQVTVSDR
LLTDRYVLNLKVFRGFTSLLWLAAQIGNKGPSATIISLDSVAGLGALVLFIILL-RESVLIETLYYRYVY-
VMKGYEAAKDVADADPEHLLNLAAVTRDVEWIEIVPAFFYYFKTAG-
FQPFNTAVAREFFGLFKERLRIVDVLLVPPDRVMALVEFVALAGTLPTSGSALPGVVACGLCEPNAAG
VLEADQDWNLHRSLGITLAFYKAQVSVKKWFDKGLHKPVAASGLSEAGALTTPFHVQVTAKVCAPV
VHRVLDLGEIEGSLIELDSAVVVADEEQTLADKPTTRDNSVGAVGGVVLAGTMNMGANLDSVERGN
NIIIELEEEQYATVNGRKDGMKLTDKVLMVLSKAYFPILRRMASANALDRGTFGLLVDVPDDADNCD
QGGEL---
PDQYSFISMMIEASLEIMETAEVLDDYHVLETGSRPPDFRIILIKGLHIRPLAGVATIIVVGSAGRSEVTAYI
QG-AVPITLRRRTFLPAFAASSCHVIDIFSTMLRPPERSTIQMLILETVAEL-
LAVTRGTNEKLERMQVLLPGLDLAELLALEAASDDFPAIVTIEAAIVAILSVAAEPVVLREMLLTLAEIAV
DAMALALALLLMLTPDQGLTQRELAREGQMKDLIQGGVVLQIKAADRMVLAATADKVRLDTDDGT
TSAFASLNLATVSDGFYVMSSAVLQMGSDLMMIIKVARVLADKETKNALVVRHPEQVKTQTTYAEGS
PGMVLQTLTGSIPPLAIKAEEIELLWTLVAALWRVKAHLANATRPHRERVLILILALTLITVLLQSEGGGM
GGG--
GPAAKILACFSILTIKAKVGEEVRKVTRRVLDLNSRLGRPEESVNPEFRGAELEQSKFEAENVEVDTMETI
RRFVDIPFLVSTRTQHNESEDRGSRMVLDVTRVRFIDL-
VTKRSEIILAARAETDGIIAREMKGDLAALHPEAAALGKIAALQHYIAISDAHISNYVTAEINSVLGMLLL
VLFINETRIAKVVWPTRREVTMVFIMAALATFFLVIGLFISIVFMLSGISWNQMTTIVLGEGVMAVRQTS
TFPKDS-
IGILQNTYADLICAGRVAKADIEKSLHIVVAGRVTRGTWYVIYLYEISQAQGEAGIMQRAFRQKTLSQS
LIDGRMEVKIKVVTSDRYA-
TYEIKGGQHRKGTRYNRVKVGHNIPVQVLPRMKMAFTPPNLVLVKTFGAVVQKRFAQALTHMRHG
AVQIGPMEVSSPYINSKKARHAPGDAVVELKPENIVRSVAVVPELIWARENEDNNAVHDKYVKTTHK
LAAKRKKAVDKAQYDHRNQVEMSFIGLAVKVLPDKAIVHATAGMLMPTASVAVTEGMKGIAATIRL
DPMEKALIAYIKRMRPQLNLAGKTDKKIVKCLNNNLDDVEYGKLGRRKIKPITIKSVYDVESRRATELVK
LLPYKDDVEGRKSQFAEK--
ALFEPLKAFMMLFGYIARSVYALNLRVIRRTILLVKCKIVVAAATVLSAIKIVGVSIRLRHKSTNVAETNHH
IKTIEKRARMTLAGAGKVKLNLSEGRVGKKGGRIKGYMPYNNYNIFDGLASGRRAKVKEVAKLKKVDK
EIKVEGIRAKQEGGRTPKPQLSNAIANTRVGVD-
VGENVQEESNPRANAEMGQIFAKIDARVYSHVGALIGHALEAVLAVVAIWGIGRAHNLLALRLRVNA
GIELHSAA-VEVAVACA-
YAAPAGEASLMVDVSEAYLQALLITADAVDVCRLRRRSLSELKKTKKDAYMSLTCFAGKGDRVKVVEA
AQTQAYEIVATRTAERHGIHAKGTYGKERAMMQILPTKPVKVWVPVFVDEVELLAPIRAFAETVGDG
VTVVKLTTDVKITMFEKAVASLAVSGKNTSVIYTVLEKVTQLMKDIIYTQRAKAHYGRGRLRSHVVTHT
KSRRHLSMMKRTVVADPSMDAARLTDGEVSVVTGATMAFVMSEQDAV-
PGDVKMHFHEEPRREAEVGIAGPETFLATEHPRVLAVLFLRAYLIHIVEIQTVKDFSNAATIYDVLRAMA
NKLDRKLSADIAKTRMVLADNLMEMGVTEVAARREAAGDPILSSYWINGVTANADIVLLVANLEVPT
VPENIHCAIRLFYQRVTLQAKERGGDVSVAVVPANKQIEAAEDTVIYSIKGKLPGSAQMKLTVEGISRSI
VKTLAVQLFQVDTFLDDTWLGTLLRAAMDVVFVDAGDGREAVDHVVERDTEFEFAPQKDIVSRKIRI

YTPAAPSAVVIWDPAIEREDKAEAQGTILKIYGPNLQDINCFTDIGSEPKIESLKSWDEKEKYVVHRDDI
PEDI-
TLVGKSSRVNKEFAIPDVAVCAPSKPGVTESIDRGEPKAGTCGEVANMAYPQESLVNNVSSANVQKN
ISMVLAGHPVGEMGISREIERHLETNMCEIFVDKADIDVALDFSTLALTVLRVGLDPPNARVQIGGKES
RKLMAVNDGDAPLQRHPKVSEAAGGQ--AGVKATLEDEALELDGVVHGDITFTLTDLPEGRV-
VTPVFNSVKAQLKIDKVILKAFHVVTLSFSNGKGEDHLIKLRLNALKKNTRHYEMLLKIRETECAESPHAI
AVGKVLKIDKLLQTICVAHYKAIEPLLAADLTALGVMGEQGLLHVVPRLMILAVMHLWGLEAAMELRR
WDLQAARCVTDQTMGLATRARHYSY-CISEVVGNTFFAMTKEDDTEQELHARNPI-VLL-
CGKFGMV-
ISPSGLRLGSTAPTPLGRDNREDLILSTERLQDNMSYYVLQILLVFVDRVILAYGSGEAPIKFLVGDQAV
TGRQNPGMVTLARRTFPLELQRDAVFVMVFADSTMKNLSHRKMHMNLVLIKSKGWVVNVSTKILIS
DQAIASYAEDVGAHTQPSESALAAITSRVMAPIVLVVSQKPTAPSKSLFLEALALYDEVTATVLNAPKK
YYIFEGDVISILGACDAAVLLGMQRPQPDRMRRAMVADAIRLAIALRAAGIVTRLEDGLINFARAVGLA
AGVYPDFGALVMAVIMPDALAFRVRNHAVSTKRSKPYKGHTKFLHKADVNKSLIAAGVDTIA
>GCA_900129845
AVIKPVTMPVYAPVLMIRQQQVATVSGAGSPSLRSKTVYAKSGLHRKDETKWVAKPLYH-
GVLKLGNIVQLVSISVDNRAVCTVAQLVEQEVVVHGMVALEVVKKKIALEAIEGMTAEDPAKVVDY--
EIALKKFRDADDRADLLPVMLGLTLKIKFDPHQPVIHDLPIPVIYEVV-
GIMAQKGTIEVDDCAISTAVSDVDPIQYVSPIDLEVLAGDQMAFIDQEAAVAAAREAIALEELALESGV
LVVGI-PLLIPV-FEADGDT-MSFANRSRTLKTIVNMAISYEHLNRARLELAIAAAM-
MEQSQAAALAEKRGTAEEIRIVKRMQKSAGLADDPELVMLDDAAEGNSSWAGQIYDDPISGSSESD
SAGNIVHAANKRQNYARSITHLRAVEISDQAAAPIMRGVKHNAKSRSARKLDDEIAELNFPNVPFGV
AIKAESDKPKSTLLHDATEGEQILMLIVRWSVTWKTAIVIFLLS-
ADDVVGLIEIGAIGAIGQKDGLLNRHHKLNGSLIEQKRVVEKLTLRLRAALKQKLGANNVIIFVVKVAG
DLVASAKVQM---SVGTVVAIQGPTIFVRRSRQTVIGDQEATV-
VLKGVAEGPADAQALLRIRVDHTLVKLSVLLIPEVPVLVESVTIREGVGKALQIIQLDVYLLRDMGTGEE
TLAKSQSRELIREMLQVDPITGVRLAYGGLRERESKALSIKEIDTML--
LAIVKHITEPRMENLPAGVCRTFIEAGPRAAVGMLFLLLDDAGKIYEIDGDLRENTKSIKSMIGIVDAAKP
KALSAGYATTRLKLRYADVYVLLSDADLYAYAEERKEVLRMVPVYITNAAKRRQLIIFALSLKGGHKRAR
AALDVMIAAAILESQAENAHVNHEEPEILLDDAEVVGSEMMISRQSALEVKQVIKIDVIVIFPEFQEKM
VVSLKFFCTEVAIFVIALIVTVGQMLSREPSHAQEGGAGYQKRIRAHSILFALAAMFERSNLRDA--
PDLFMGLGLASPRYGIIYVSQPKRKQMPSEPDRDRVDGFDLVHAVKADTTKNHELDMPEAMALAVE
-DATINVNAGVLATKGEMTDVEKVDSKRKMSLAVEKWNIAKLDVIDVNISGAYPFRSLE-
IEQADISAALHKKDIWFLFLPGANNADMEEEKLDGAGASTAEVAEDKQTGILLVNPVLVVIQDSNTAS
LIANDGLEEWGTMAYKKHYALAARMRLHMRTIDAIEVGVVMEPNKQKAEKIKTVKLEGDTRFRQLAT
EGVKEQMP-IHALNPDFAV-LPVLVTPAVGRVAADESVLGADVPCDFSL-
RELQKSQKGKALPISGIKVLGMRAARAMIEEDIMVAGKVAA-
LLAIGAVETILEIVLMNDSEATLPWRLSVAERLEADTKRASGFASRPFKSKPSLCKTQAHAKVADPIQEI
AAGYVAAEERFVFPVEGIAIQLILASPHSRVGGAKRAGIGHLHEMAKELIGKAVLMVLNAPDTNTEGLL
NYLTTNQPTDLLFEDCLDVVIDMRRMSVV--
VHINTV-
HVVVKGVAEVGDYEIMAAAFPSGLVQVERVDDRTGRERTIMWMYGAMEELWLNSVTWADWVQQ
VQIGAIILHVYAHAINRAFSLERLQIAGWPKEASHWWNTWQLWLNKDGFFGYDAAFRA-
AAVLKSDLYDYEMSDVEGVGRYQSRQELRVGYRIREQVSYARLDPRTDILLQSQTRLSVIRHALDMVA

ILIIRDIVAEFTLVVAMEDVAMGHVECVLADCFDFTKKNADIGPVGIRTPDPILGDGTVMKEAVLGKIDL
ANTVADFRYKILVDGGRELGVEWLSI-NMLYERIWLPPAQAALKDLTPIRFFLSHPEINV---------
ISKARADRLMAGALVEKILENIAAFAEPEATTHQSNKERHFKYYGEEVDPVFLAYIFDATALVSTQVTVS
DRLLKDRYVLNLKVFRGLTSLWWMSLQIGNKGPAATIITLGTVAGLGALVLFIILL-
RQSVIIETLYFRYVYV-
MRGYEAAKDVADAEPEHFLILASASRDVEWIQIIPAFFYYFDTATSNLTKNTALAREFFGLFAERLRVIV
VLVVPPERVLELVEFVALAGTLSTSAEALVGVIACEFCEPNAAEVLEADQDWNLHRSMGITLAFYDARE
SVKGWFDKGLHKDVAETGLSEAGALTTPFHAGEEAKRCVPAVHRVIDLGEIAAALIEIDSAVVVADEE
QTLADKPTTRDNSVGAVGGVVLAGTMNMGSNLDSVERGGNIIIELEEEQYSAVNGKKADGKMTDKV
LMVLSKAYFPVITGFTSGNALDKGVINVLIQAPSDADNQDHGQGLTIPAPTIHAETAPLFAEADIERMA
WVAPMHMLGTLDAVLGFEPDQYSFISMMIEASLDIMETAEV--
DYHVLTKGSRPPAFRVILIKGLHIRLLAGIGTIIVVGSAGRSEVTAYIQG-
AVPITLTKVTFLPAFAASSCHAIDIFSTMLRPPERSAIEMLILETVAEL-
LEATRIENDKLERLDALLPGLDLAVLLELVAAAADFAKIVTIDKAIAAILSVAAEAVVLRELLLTLAEIAVTA
LALAMALLLMLTRDQGLTQKETSREGQMKELIQGGVVLQVKAADKMMLASTADKVRLDTDEGTTSA
FASLNLATVSDGFYVLSSAVLQLSSDLMMIVSVARVLADKEAKNALVVRHPEQVKTQATYAEGSPGM
VLQTLTGSVPPLAIKSEDVELLWTLVAALWKVKAHIANAARPRLERVLILILASSLIAVLMQSEAGGLGG
G--
GAAAKLIAGFSILTIKAKAGDEVRKVARRVMDLNSRLGRPEEAVNPEFRGAELEQTKYEAENVEVDTM
ETIRRFVDIPFLVSTRTQHNESEDRGSRMVLDVTRVRFIDL-
VTKRSEIILAARAETDGIISREMQKDLQALQAELAALGKIAALQHFIAVSDGHISNYVTADINSVLGMILL
VLFINETRISKVVWPTRREVTMVFIMAALAVFFLVIGLG-
QIVFMLSGISWQQLTTIVLGEGVMAVRQTGTFPADS-
IGILQNTYADLICSGRVAKADIEQSLHIVVAGRVSRGTWYVIYHYEITQAQQNSGIMQRAFRQATLSQ
SLIEGRMEVKIKVVTTDRYA-
TYEIKGGQHRKGTRYNRVKVGHNIPVQVLPRMKMCFGPPNLVLVKTFGAVVQKRFAQPLTHIRHGA
VTIGPMEVSSPYIKNKKARHAPGDSVVDLKPDNILRSVAVVPELIWARENEDNNAVHEKYVKTTHKLA
AKRKKAVDKAQYDHRNQVALSFIGLAVKVLPEKAIVHATAGQLMPTASVAVTEGMKGIAATIRLDPM
EKALIAYIKRMRPQLNLGGQTDKKIVKCLNNNLDDVEYGKLGRRRIKPITIKSVYDVEHEEGTELVKLMP
YKDDVETRKSQFAEK--
ALFEPLSAFMMMFGFIARAVYALSLRVIRRTILLVKTKIVVNASTVLSAIKYIGISIRLRHKSTNVAETNHHI
KTIEDRARMTLAGAGKVKLNLRPGKVGVKAGRIKGYMPYKNFNIFDKLESGRRAKVKALAKLKEVDKTI
SVDGIRQKQ-GGRTPQADLSNAV-
NSRVGVDIVGESEQEESNPRANAEMGQIFAKIDARVYSHVGALVGHALTAVLAVCSIWGIGKAHNLL
AVKLKINAGIEMHSAA-
VEAAVAAAAFADNGGEASIIADISEAYLGVLLMTETGIDVCRLRRRGLAMLKKTKKDAYMSLTCFGGK
GDKIKIVEASQTQAYEIVATRTAERHGIKAKGTYGKERAMMQIVPSKPVKVWVPIFVPEPELLAPIRAFA
ETVGDGVTVVKLTTDIKVTPFEKAVASIAVTGKSTSVVYTVLEKVTQLSKDIIYTQRSKAHYGRGRLRSH
VVTTTKQKRHLSMMKRTVVADPHMSAARLTDGEVSVVTGATMAFVMSEQDAV---
DADLHYHEEPRREAALGIAGPETFLTTEHPRVLALIFLRAYLIHIVEIDTEKGFADAATIYEVLRAMQNKL
DRKLSADIAKIRMVLADNVANMGVTDVAPRREEAGDKILSAFWINGVPANADIILLVANMEVPCVPE
NIDCAVRLFYQRITLQAKERGGDVSVALVPAQNQIEAAEGTVIYSIKGQTPGTSQMKLNIEGISRSIVKS
LATQLFQVDTFLNDTWLGTLLRAAEDVIFVDAGDDKEAVDHVVERDTLLEFAPQKDIVARKIRIYTPAA
PSAVVIWDPAIDREDKADAQGTILKIYGPNLQDINCFTDIGAEPKIESLKAWDEKEKYVVHRDDIPEDIT

TLVGKSSKVNKEFAIPDVAVCAPSKPGVTESIDRGEPKAGTCGEVANMSYPQESLVNQVSSANVQKN
ISMVLGGHPVGEMGISRGIERHLETNMCEIFVDKSDIDVALDFHILRLTAQRVGLDAPNAKVQIDGKES
RKLMAVQDGDADLQRHPKISEATGTGLGAGIKARMPDDTLILDGVMHGDISFTATDLPEGRV-
VTPVFNAVKAQLKIDKVMLKEFHVVTLSFSNDKGEEHLVKLRLNALKKSTRHYEMLLKIRETECAESPH
AISVGKVLKIDALLQTICVAHYKEIEPLLADDLTALGVMGEQGLLHVVPRLMILAVMYAL-
LELALAVAHWGLQAEASVTDQNLGLQIRERAWTY-AISEVVGNTFFARTKDDDTEQRIHARNPI-
VLL-YGKFGMT-
ISPSGLGLESTPASDQEQENRNDLILSSERLQDQMVYYILRILLVFVDEVILAYGSGGAPIKFLVADQAV
TGRQNPGMVTLTRRTFPLELQRDATFVLVFEDSTMKNLSHRKMHMNLVLIKSKGWIVNVSTKILISDQ
AIASYAEDAGAHTQPSESALAVITSRVMAPIVLVVSQKPTAPSKTLFLEALSLYEEVTATVLNKPKKYYIF
EGDLVSVLNQLTAALLIGMQRPQPDCMRRAVIAQAIRLAIALKASELAQRLDDGLINFSRAVSLAAGL
YSALDMLPVAIIT-EAIAFRVRNHKTSTKRDRPYKGHTKFLHKADLDKGLVANGVDTVA
>GCA_900143545
AVIKPVTMDTYAPVLMIRQQQVATVSGAGSPTLRAKTVYRKSGLQRKAETKWVSKDFYY-
GVVKLGNIVQLVSISVDKRSVCTVAQLVDQEVVVHGMVALEAVKKKIALEAIEGMTAEDPAKVVDY-
SEIELRKFRDADDRADLLPVMLGLTLKIRFDPHEPLVHDLPIPVVFEVV-
GIMVQRGQIEVDECGVSTALSDVDPIVYVSPIDLAAVAGDQMAYIDQEAAVTAAKAAIELEELALESS
VLVVGI-PLLIPV-FETDGDV-MSFANRSRTLKTIVNLAVSYPAIKRA-LDSA--AAM-
MGQSQAAALAEKKGTAEEIRIVKRMQRSSGLADDPDLVMLEDAAEGNSNWAGQIYDDPISGSSESD
SAGNIVFAANKRQNFARSITYLRAVEISDQAEAPLMRGVKHNAKSRTSRKVNDAIAVLNFPNVPEGIAI
RAEAGKTGDILLHDATEGEEILL---
LNRHHKLNGSLIEQRRIVEKLTLRLRVALKQKIGKEQIIIFVVKVAGDLVANAKIQM---
SVGTVVAIQGETIFVRRHRQTVIADQEATV-VLRGVAEGPADAQALLRIRVDHTVVKLSVLLIPE-
PVLTESVTIREGVGKALQIIQLDVYLLRDMGTGDETLAKSKS---------------------------------
----
IAILENITEPRMANLPDGVCRTFVNAGPRAAVGMIFLLLDDAGKIYTIDGDLRENTKSIKSMIGIVDAAK
PKTLSAGYATARLKVRYADVMVLTSDADLYAYAEERKEVLRMVPVYITNAAKRRQLIIFALSLKGGHRR
ARAALGVMIAAAILESQAEEGSVNHEDHAVLLDDGEIVGSEIMISRQAALDIKQIIKTDIIVIFPEFQEKM
VVSLKFFCTDVAIFVIALICTVGDMLSREPSHAQEGGAGFQKRIRGHSVLFALAVTFDRRNLRGTDA-
DLFLDLGLAAPRYGVLYTEQPKRKQMPSEEDKEKVDGFDLVNAIKSDTTKNHELDMPEAMALAVE-
DGSINVNAGVLAMTGDMAAVEKVDSKRKMALAVEKWTISRSDIVDIKLSGAHPFRALS-
VGQADVSAALGHKQI-
FLFLPGANNAEMEEAKLDGAGSSTAEAADDKQLGILLVKPVLVVIQDSNTASLIANGGLEEWGTMAY
KKHYAFAARMRLHMRTID----GVVMDPNKQKAEKIKTVKLEGDTRFRQLATEGVKEQMP-
LHALNPDFAV-LPVLVTPAVGRVAADADVLGADVPCDASL-
RELQKSMKGDALPISGIRLLGMRGARAMVDEDIMVAGKVAA-
LLVIVAAETILHIVLLNDSEATLPWRLSVAERLEADTKRAAGYAARPFKSKPSLCKTQAHAKIADPIQEIA
AGYVATEERFVFPVEGIPIQLILASPHSRVGEAKRAGLGHLHEMAKELQAKAVIMVLNEPDTNTEGLLN
YLTTQQPTDLLFEDCLDVVIDMRRMSVV--
VQLNTV-
HCMIPGTSDLADREIMAAAAHSGLVQVERVDDRTGRERTLMWMYGAMEELWLLSVRYADWVVDC
AIGAILLHVYAQNINRAFSLNRLQLAGWPKEATRWWNTWQLWLNKDGFFGYDAAFRA-
KAVLKSELDDFEMSDVEGVGRYQSRQQLRVGYRIRQQVAFEQLDPRTDILLQSMTRLSVIHHALDVV
AILIVREVVVEFTLVVAMEDVAMGHVECVLADSFDFTAKDADIGPVGIRTPDPVLGDGTVLKEAVLGKI

DFANTVADFRYKMLTDGGRQLGVSWVTLQNLLYARIWLPPAHAALKNLVPIAFFLGHTELNLAIFTEL
WAGISKARANKLMAGALIEKILEKVAAFADPEATTHQSKKLRHFKYYGEEVDPVYLAYIFQQTALVATQ
VTVSDRLLKDRYVLNLKIFRGFTSLLWLSAQIGDKGPAATVITVQTIMGLGALVLFIILLLRVVVLVETLYF
KYVY--
MLGYQAAKDVADADPEHFLSLAHASRDVEWIEVVPAFFYYFKTATSRLSKNTALAKEFFGLFQERLRIV
DVLVVPPERVEALVEFVALAGTLATAAKVLPRVIACGFCEPNAAAVLEADQDWNLHRSMGITLAFYD
ARESVKGWFDKGLHKDVAATGLREAGALTTPFHAGVTAKQCVPAIHRVLDLGEVEAALIEIDSAVVVA
DEEQSLADKPTTRENSVGAVGGVVLAGTMNMGANLDSVERGGNIIIELEEEQYSAVNGKKSDGKVT
DKVVMVLSAAYFPVLKRIASANALDKGAFGLLIEAPDDADNKDHGRGLTIPAPTVHAESAPLYAESEIE
RIAWIAPLHLLGTLDAVLGFEPDQYSFISMMIEASLEIMETAEV--
DYHVLEKGARPPSFRVILIKGLHIRLLAGVCTIIVVGSAGRAEITAYIQG-
AVPITLNKQTFLPAFAASSCHVIDIFSTMLRPPERSAIEMLILETVAEL-
LEVTRIENDKLERMEALLPGLDASALLQLVAAAKDFASIVTIDQAITAILSVAAEAVVLREMLLSLAEIAV
TALALALALLLMLTPDQGLHQKEAAREAQMKALIQGGVVLQVKAADRMMLASTADKVRLDTDEGT
TSAFASLNLATVSDGFYVLSSAVLQMSSDLMMIVAVARVLADKETKNALVVRHPEQVKTQSTYAEGS
PGMVLQTLTQAIPPLAIKAEDVELLWTLVAALWRVKAHLANAARPHTERVLVLILALALITVLLQSEGG
GMGGG--
GPAAKLLAAFSILTIKAKAGDEVRKVARRVMDLNSRLGRPEEAINPEFRGEELEQSKYEAENVEVDTME
TIRRFVDIPFLVSTRTQHNESEDRGARMVLDVTRVRFIDL-
VTKRSEIILAARAETDGIIAREMGKDLAALHPEAAALGKISALQHFIAVQDAHISNYVTATIHAVLGMILL
VLFIQEVRVSKVVWPTRREVTMVFIMAALAVFFLVIGL--
QIVFMLSGISWNALTTIVLGEGIMVVRQTNTFPEDS-
IGILQNTYADLICSGRVAKADIEASLHIVVAGRVARGTWYVIYLYEISQAQDQSGIMQRAFRQMTLSQ
SLIDGRMECKIKVVTSDRYA-
TYEIKGGQHRKGTRYNRVKVGHNIPVQVLPRMKMAFGPPNLVLVKTFGAVVQKRFAQALTHIRHGA
VTIGPMEVSSPYISNKKARHAPGDSVVDLKPDNIVRSVAVVPELIWARENEDNGAVHDKYVKTTHKL
AAKRKKAVDKAQYDHRNQVELSFIGLAVKVLPDKAIVHATSGALMPTASVAVTEGMKGIAATIRLDP
MEKALIAYIKRMRPQLNLGGQTDKKIVKCLNNNLDDVEYGKLGRRKIKPITIKSVYDVEHRRATELVKL
MPYKDDVETRKSQFAEK--
ALFEPLSAFMMMFGFIGRAVYALYLRVIRRTILLVKTKIVVNASTVLSAIKIIGVSIRLRHKSTNVAETKHHI
KTIEARARMTLAGAGKVKLNLSPGKIGMKGGRIKGYMPYKNFNIFDGLASGRRAKVKELAKLKSVDKSI
TVDGIRTKQQGGRTPKAQLSNAIAKTRVGVDIVGENEQEESNPRANAEMGQIFAKIDARVFSHVGALI
GHALEAVLAVVAIWQIGRAHNLLAIRLKVNAGIELHSAA-VDVAVAAAAFAQNAGEA-
VVADISEAYLGALALSEMSVEVCRLRRRGLNELKKTKKDAYMNLTCFGGKGDKVKIIEATQTQAYEIVA
TRTAERHGIRAKGTYGKERAMMQITPTKPVKIWVPVFVNETELLAPIRAFAESTGDGVTVVKLTTDVKV
TMFEKAVASIAVTGKATSIVYTVLEKVTELMKDIIYTQRAKAHYGRGRLRSHVVTTTKQRRHLSMIKRTV
VADPSMDASRLTDGEVSVITGATMAFVMSEQDAV--
GDVRLHFHEEPSREADLGIGGPETFLTTEQPRVLALLFLRAYLIHIVEIGTTNGFSDAATIYDVLRAMQN
KLDRKLSADIAKVRMVLSDNVTSMGVTDQAPRREAAGDKILSAFWINGVTANADIILLVANLEAPCVP
ENIDCAIRLFYQRITLQAKERGGDVSVAIVPAANQIEAAENTVIYTIKGKEPGTGQMKLTIEGISRSIVKSF
AVQLFQVDTFLDDTWIATWLRAALQVVFVGAEDGKEAVDHVVERDTELEFAPQKDIVARKIRIFTPAA
PSAVVIWDPAIEREDKAEAQGTILKIYGPNLQDINCFTDIGAEPKIESLKAWDEKEKYVVHRDDIPEDI-
TLVGKSSKVNKEFAIPDVAVCAPSKPGVTESIDRGEPKAGTCGEVANMSYPQESLVNTVSSANVQKNI
SMVLSGHPVGEMGISREIERHLETSMCEIFVDKDDVDVALDFHIVALTALRVGLDPPNARVQIDGKES

RKLMAVQDGEADLQRHPKVSQATGTGLKAGVKARMPDDNLILDGVMHGDVSFTATDLPEGRV-
VTPVFNEVKAQLKIDKVILKAFHVVTLSFSNDKGEEHLVKLRLNALKKSTRHYEMLLKIRETECAESPHAI
SVGKVLKIDALMQTICEAHYKAIEPLLADELTALGVMGEQGLLHIVPRLMIL--MHL-
GIAVAHQIQKWGLQAAAVITDQTMGLALRDRDWSF-AISEVVGNTFFARTKADDTEQKIQARNPV-
VLL-LGKFQMI-
ISPSGLKLASTAASDIEEGNQEELILSTERLQDAMEYFIVDILLVFVDEVILAYGSGDDPIKFLVAEQAVTG
RQNPGMVTLTRRTFPLELQRNATFVMVFEDCTMKNLSHKKMHMNLVVIKSKGWIVNVSTKILISDQA
IASYAEDAGAHTQPSESALAVITSRVMAPIVLVVSQKPTAPSKTLFLEALSLYDEVTATVQNKPKKYYVF
EGDVVSVLDPLDAAVLLGMQRPQPDRMRRAVVAEAIRLAIALQASDIVTRLEEGLVNFSRAVNFAAR
YY-IQAAP----------EFRVRNHDVSTKRSRPYKAHPKFLHKAEVDKSLIATGVDTLA
>GCA_900156505
AVIKPVTMDAYAPVLMIRQQQVATVSGAGSPTLRAKTVYAKSGLQRKSETKWVSKDFYY-
GVVKLGNIVQLVSISVDKRAVCTVAQLVDQEVVVRGMVALEAVKKKIALEAIQGMTAEDPAKVVDYA
SEIELRKFRDADDRADMLPVLLGLTLKIKFDPHQPVVHDLPIPVVFEVV-
GIMVQRGQIEVDECAISTAISDVDPIVYVSPIDLAAVAGDKMAYIDQEAAVANAKQEIDLEELALESSV
LVVGI-PLLIPV-FAADGDV-MSFANRSRTLKTIVNLAVSYPEIKRA-----IAAAM-
MDASQAAALAEKRGTAEEIRIVKRMQRSSGLADDPELLMLEDAAEGNSNWAGQIYDDPISGSSESDS
AGNIVYAANKRQNFARSITYLRAVEISDPAAAPLMRGVKHNSKSRAARKVDDAIAALNFPNVPEGIAI
RAAAGKPRDLLLHDATEGEEILLIIVRWHVTWKTAVIISLFPLADNVVGIITMGGIGAVGRKDGLLNRH
HKLNGSLIEQRRIVEKLTLRLRAALKQKLGAEQIIIFVVKVAGDLVAKAKVQM---
SVGTVVAIQGETIFVRRHRQTVIADQEATV-ILKGVAEGPADAQALLRIRVDHTVVKLSVLLIPD-
PVLTESVSIREGVGKALQIIQLDVYLLRDMGTGEETLAKSRSREVIREVLQVDDITGVRLAFGQLRERLVK
GGTMKELG-----
IAILKDITEPRMANLPAGVCRTFVNAGPRAAVGMIFLLLDEAGKIYTIDGDLRENTKSIKSMIGLVDAAK
PKTLSAGYATTRLKIRYADVTVLTSDADLYAYAEERKELLRMVPVYITNQAKRRQLIIFALSLKGGHRRA
RAALGVMIAAAILESQAEQGAVNHAEPAVLLDDGEIVGSEIMISRQAALDLKQIIKTDIIVIFPEFQEKM
VVSLKFFCTDVAIFVIALICTVGEMLSREPSHAQEEGAGFQKRIRAHSVLFALAVTFDRRNLRTD-
TPTLFLNLGLAPPRYGIFYTEQPKRKQMPSEADDDEVDGFDLVNAIKEDGTK-EELDMPEAMALAVE-
DGTINVNAGVLAMSGDMAAVEKVDSKRKMALAVDKWTIARVDIVDLKISGAAPFRALN-
IEQADVSATLSNKQV-
FLFLPGANNAEMEEAKLDGAGSSTAEAAADKQLGILLINPVLVVIQDSNTAGLIANGGLEEWGTMAY
KKHYALAARMRLHMRTID----GVEMDPNKQKAEKIKTVKLEGDTRFRQLATEGVKEQMP-
LHALNPDFAVLVPVLVTPAVGRVAADADVLGARVPCDASL-
RELQKSMKGNALPISGIRLLGMRAARAMVDEDIMVAGKVAA-
LLVIVAVTTITHIVLRNDSEATLPWRLSVAERIEADTKRASGFAARPFKSKPSLCKTQAHAQIADPIQEIA
AGYVATEGRFVFPVEGIPIQLILASPHSRIGAAKRAGEGHLHEMAKELQAKAVIMVLNEPDTNTEGLLN
YLTTQQPTDLLFEDCLDVVIDMRRMSVV--
VQLNTV-
HVLVPGVRDLADREIMAAAAHSGLVQIERVDERTGRERTLMWMYGAMEELWLMSIRFADWVVDC
AIGAILLHVYAQQINRAFSLERLQLAGWPMEATRWWNTWQLWLNKDGFFSYEAAFRAVKAVLKAD
LDDFDMSDVEGVGRYQSRQQLRVGYRIRQQVAFEQLDPRTDILLQSMTRLSVIRHALTMVAILIVREV
VVEFTLVVAKEDVAMGHVECVLADSFDFTAKDADIGPVGIRTPDPVLGDGTVLKEAVLGKIDLANTVA
DFRYKMLIDGGRELGVSWVTLQNLIYKRIWVPPAQAALKNLVPIAFFLGHTELNLDIFPALWAGISKAR
ANKLMAGALIEKILEKVAAFANPEATTHQSKKIRHFKYYGEEVDPVYLAYILDMFALVATQVTVSDRLL

KDRYVLNLKIFRGFTSLLWLSAQIGDKGPAATVITVQTIMGLGALVLFIILL-RVVVLVETLYYKYVY--
MLGYQAAKDVADADPEHFLSLAHASRDVEWIEVIPAFFYYFKTATSRLSKNTALAKEFFGLFKERLRVID
VLAVPPDRVEALVAFVALAGTLVTSAQALAGIIACG-
CEPNAAAVLEADQDWNLHRSMGITLAFYDARESVKGWFDKGLHKDVAATGLREAGALTTPFHAGA
TAKQCVPAIHRVLDLGEVEAALIEIDSAVVVADEEQTLADKPTTRENSVGAVGGVVLAGTMNMGAN
LDSVERGGNIIIELEEEQYSAVNGKKSDGKVTDKVVMVLSAAYFPVLKRMASANALDKGAFGLLIDAP
DDADNKDHGRELTIPAPTVHAESAPLYAESEIERIAWIAPLHLLGTLDAVLGFEPDQYSFISMMIDASLE
IMETAEVLDDYHVLETGSRPPGFRVILIKGLHIRLLAGVCTIIVVGSAGRTEVTAYIQG-
AVPITLNKKTFLPAFAASSCHAIDIFSTMLRPPERSTIQMLILETVAEL-
LEVTRIENDKLERMFALLPGLDVASLLDLVAAATDFAAIVTIDQALTAILSVAAEAVVLREMLLTLAEIAV
TALALALALLLMLTPDQGLHQKEQAREAQMKALIQGGVVLQVKAADRMMLASTADKVRLDTDEGT
TSAFASLNLATVSDGFYVLSSAVLQMTADLMMIVAVARVLADKETKNALVVRHPEQVKTQSTYAEGS
PGMVLQTLTGAIPPLAIKPETVELLWTLVAALWRVKAHLANAARPHNERVLILILALSLITVLLQSEGGG
MGGG--
GPAAKALAAFSILTIKAKAGDEVRKVARRVMDLNSRLGRPEEAVNPEFRGAELEQSKYEAENVEVDTM
ETIRRFVDIPFLVSIRTQHNESEDRGARMVLDVTRVRFIDL-
VTKRSEIILAARAETDGIIAREMGKDLAALHPEAAALGKIAALQHFIAVQDAHISNYVTAEIHAVLGMILL
VLFIQEVRVGKVVWPTRREVTMVFIMAALAIFFLVIGLGVQIVFMLSGISWNQLTTIVLGEGIMVVRQT
STFPEDS-
IGILQNTYADLICSGRVAKADIEASLHIVVAGRVARGTWYVIYLYEISQAQDQSGIMQRAFRQMTLSQ
SLIDGRMEVKIKVVTSDRYA-
TYEIKGGQHRKGTRYNRVKVGHNIPVQVLPRMKMAFGPPNLVLVKTFGAVVQKRFAQALTHIRHGA
VTIGPMEVSSPYISNKKARHAPGDSVVDLKPDNIVRSVAVVPELIWARENEDNGAVHDKYVKTTHKL
AAKRKAAVDKAQYDHRNQVELSFIGLAVKVLPEKAIVHATSGQLMPTASVAVTDKMKGIAATIRLDP
MEKALIAYIKRMRPQLNLGGQTDKKIVKCLNNNLDDVEYGKLGRRKIKPITIKSVYDVEHRRATELVKL
MPYKDDVESRKSQFAEK--
ALFEPLKAFMMLFGYIARAVYALYLRVIRRTILLVKTKIVVNASTVLSAIKIIGVSIRLRHKSTNVAETNHHI
KTIEARARMTLAGAGKVKLNLSPGKIGKKGGRIKGYMPYKNFNIFDGLASGRRAKVKELAKLKEVDKTIS
VDGVRQKQQGGRTPKAQLSNAI-
NTRVGVDIVGENEQDESNPRANAEMGQIFAKIDARVFSHVGALIGHALEAVLAVVSIWDIGRAHNLL
AVRLKINAGIEMHSAA-
VDTAVAAAAFAQSAGEASVIADISESYLGALAISAMSVDVCRLRRRGLSELKKTKKDAYMSLTCFGGK
GDKVKIVEATQTQAYEIVATRTAERHGIRAKGTYGKERAMMQILPTKPVKIWVPVFVTEVELLAPIRAF
AESVGDGVTVVKLTKDIKVTMFEKAVASIAVTGKSTSVVYTVLEKVTELMKDIIYTQRAKAHYGRGRLR
SHVVTTTKQSRHLSMIKRTVVADPSMDASRLTDGEVTVITGATMAFVMSEQEAV--
GDVDLHFHEEPRREAEVGIGGPETFLTTEQPRVLALLFLRAYLIHIVEIGTVNGFSDAATIYDVLRAMQN
KLDRKLSADIAKTRMVLTDNVTDMGVTEVAPRREAAGDKILSAFWINGVTANADIILLVANLEVQCAP
ENIDCAIRLFYQRITLQAKERGGDVSVAIVPAANQIEAAENTVIYTIKGKTPGTGQMKLTIEGISRSIVKSL
AVQLFQVDTFLDDTWIATWLRAALQVVFVGAEDGKEAVDHVVERDTELEFAPQKDIVSRKIRIYTPAA
PSAVVIWDPAIEREDKAEAQGTILKIYGPNLQDINCFTDIGAEPKIESLKAWDEKEKYVVHRDDIPEDI-
TLVGKSSKVNKEFAIPDVAVCAPSKPGVTESIDRGEPKAGTCGEVANMAYPQESLVNAVSSANVQKN
ISMVLSGHPVGEMGISREIERHLETSMCEIFVDKDDVDVALDFYILQLTPLRVGLDPPNARVQIDGKES
RKLMAIQDGDAALQRHPKVSQATGTGLKAGVKARMPDDALMVDGVMHGDISFTATDLPDGRV-
VTPVFNNVKAQLKIDKVLLKAFHVVTLSFSNDKGEEHLIKLRLNALKKSTRHYEMLLKIRETECAESPHAI

SVGKVLKIDALMQTICQAHYKEIEPLLADELTALGVMGEQGLLHIVPRLMILAVLQLFRLEIALQIDNW
GLQAEALVTDQTMGLYLRDRAWSF-AISEVVGNTFFARTKETDTEQKIRARNPV-VLL-LGKFEMI-
ISPSGLGLASTAESPQERGNADELILSTERLQDEMGYYVLQILLVFVDEVILAYGSGEGPIKFLVAEQAVT
GRQNPGMVTLTRRTFPLELQRNATFVMVFEDSTMKNLSHKKMHMNLVVIKSKGWIVNVSTKILISDQ
AIASYAEDAGAHTQPSESALAVITSRVMAPIVLVVSQKPTAPSKTLFLEALSLYDEVTSTVQNKPKKYYIF
EGDVVAVLGTLDAAVLLGMQRPQPDRMRRAIVADAVRLAIALRASGIVTHLEEGLINFSRAVNFAALF
Y-SNGA-----------EFRVRNHDVSTKRSRPYKAHPKFLHKADVDKGLIATGVDTLA
>GCA_900172245
AVIKPVSLNTYAPVLMIRQTQVATVSGAGSPSLRAKTVYRKSGLSRKTSTKWVAKPLYY-
GVLKLGDIVQLVSISVDNRSVCTVAQLVEQEVVVHGLVALDAVKRKIALEAIDGMTTEDPARVAEFEA
ELDLQKFRDAQEHADLLPVMLGLTLKIKFDPHEPVVHDLPIPVIFEVI-
GIMAERGGIEVDECAISNAVSDVDPITYVSPIDLDAVTGEQMAFIDQEAAVNAARTAIELEELALEAGIL
VVGIDPLLIPV-FADDAAV-MSFANRSRELGSIVDLAVAYPEITRARI-AGIASAL-
MVRAQAEALAEKRGTAEEIRIVKRMSRSSGLADNPDLMVLEDAAEGNSAWAGQIYDDPISGSSESDS
AGNIVHAANKRQNFARSITYLRQVEISDKAKAPLMRGVKHNSKSRSARNVDDAIAVLNFPNVPHGV
AIRAEQDKPAATLLHDATEAEAILLLIVRWKVTWKTAVFLLLVF-
AGDIVGLVQLGGLAAVGRPDGILNRHHKLNGSLIEQKRVVEKLTLRIRAALKEKLGADQVIILVVKVSG
DLVADAKVQMG-
GTLGSVVAIQGDVIFVRRHKQTIVTDQESTVEILRGVAEGPADAAALLRIRVDHTLVKLTVLLIPE-
PVLVESVTIREGVGKALQIIQLDVYLLRDMGTGDETLAESQSRELIRELLMVDPITGVRLAYGQVKERLN
KQLTIKDLD-----RSIL-
DITEPRMENLPEGVCRTFIQAGPEAAVGQLFLLLAEAGKLYELDGDLRELPKTVKALIGLVDAKAPKVLS
ASYATARLKLRYADCTVLEAEADLYAYAEERRELLRMVPVYIINAAKRRQLVIFMLSLRGGHRRALAAIG
VMLAAAILESHADHGKVHHAAPEILLDSAEIVGTAMMISRQASLDLKARSKTDVIVVFPEFQEKMVVS
LRFFCTDVAIFVIALIVTVGEMLSREKSHAQEAGAGYQKRIRGHSMLFALAVVFDRRNLRDA-
TGDLFLALGLAAPRYGILYTNQPKKKQMPSEPDSDRVDGFELVHAIKEDERKNHELEMPEAMALAVE-
SETINLSAGVLALSGEMAAVERVDSKRKMSKAVEKWNISRLQVRDVALSGAQPVRATG-
IEQAALSAALGAKNV-
FLYLLGASNEELEESRLDGAGSSTANVAEDRQRQILLVHPVLVVIQDSGAASLVSNGGVDEWGTMAY
KKHYAYAARMRLHMRTIDAIEVGVEMAPNKQKSEKVKTVKLEGDTRFRQLAVEGIKEQMP-
IHALNPDFAVLVQLLVTPAVGRVAADAGVLGAEVPCDFSL-
RELQKSMKGAALPISAIRLLAMRAARAMIEEDIMVAGKIAA-
LLKISALETILAVILRNDSEAWGPWRLSVAERLEADTKRASGFAARPFKSKPSLCETQAHAKVADVIQEI
AAGYVAAEQKFVFPVEGIAIQLILSSPHSRVGEAKRAAEGHLHDMAKELVAKAVLMLQNEPDTNTEG
LLNYITTNQPTDLLFPDCLDVIIELRRMSVV--
IHLNTV-HVVVRGVRDVGDFQIMDANA-
SGLVQVERIDERTGMEKTIMWMYGAMEELWLLSITYADWVTDCAIGAIVLHVYSQSINRAFSLKRLQL
NGWPKEAARWWNTWQLWLNKDGFFGYEAAFRAAAQVLKAALYDYDMSDVEGVGRYQSRQQLR
VGYRIREQVEFARLDPRTDILLQARTRLSVIRHALTVVAILVLREVVGEFT-
VVAEEDVAMGHVECVLADCFDFTRKNAHIGPVGLRTPETVLGDGTVFKEALLGKVDWANTVADFRY
LVIVDGGRRLGVTWVALRNILYPRIWLPPAEAALKNLLPICFFLSHPELDL---------
ISKARADKLMAGALVEEILEGVARFADPEATTHQTNKERHFKYYGEEVDPVYLAYILEIFALVATQVTVS
DRLLKDRYVLNLKV-RGVTALLLLALQLAEKGPAATVITVGTIAGLGALVLFIILL-
RDEVLVETLYHRYVYV-MRGYSAAKDVADADPEHFLRLAAVSRDVEWIEIIPAFFYYFKTAG-

FQPFNTAVAREFFGLFAERLRVVDVLAVPPDRVVALVEFVALAGTLPTSAQALPGIIATD-
CEPNAAAVLEAEQDWNLHRSMGITLAFFEARESVKGWFDKGLHKEVAETGLSEAGGFTTHNDCEVE
AKKCLPAIHRVLDLGEIEAALIEIDSAVVVADEEQTLADTPTTRDKAVNAIGGVVLAGTMNMGANLDS
VERGGNIIIELEEDQYHAVNGKKSDGKLTDKVLMVLSRAYFVVLRALASANALDRGVIDLLIDAPQEAD
NLDHGG-MVIPAPSVLGETTPLYAESEIDRIAYIAPIHLLGTLAAVL-
FEPDQFSFISMMIEASLEIMETAEV--
DYHVLEKGGRPPAFRVILIKGLHIRLMAAIGTIIVVGSAGRAEVTAYIQGRAVPITLNKNTFLPAFAASSC
KAVDIFSTMLRPPERSTVQMLILETVAEL-
LEVTRLENEKLERIEMLLPGLDMAELLDLAAAATDFANIVTIGAAITAILSVAAEAVVLRELLLTKAEIAITA
LALAIALLLMLTGDRGLTQKESARDGQMKELIQGGAVLQIRADDRMVLAATADKVRLDTDEGTTSAF
ASLNLATVSDGFYVLSSAVLQMSADLMMIVTVARVLADKEAKNALVVRHPEQVKTQPTYAEGSPGM
VLQTLTGCIPPLAIKPEEVELLWTLVTALWRVKAHLAQARRPLRERVLILLLALALIVVLMQSEGGGMG
GG-
VGSAAKVLAAFSILTIKAAVGDEVRKVTRRVMDLNSRLGRPEETVSPEFRGEELEQSKFEAENVEVDTM
ETVRRFVDIPFLVSVRTQHNESEDRGARLVLDVTRVRFIDL-
VTKKSDVILVERVETDGIIAREMAKDLQALQTEAAALGKIAALDHFIALNDGHISNYVTEKIHSVLGMLL
LVLFIQQVRIAKVVWPTRREVTMVFIMATLAIFFIVIGLAVNLVFMVGGISPLQITTLVLGEGVMAVRQT
STFPAES-
VTILQNTYADLICAGRVTKADIEASLHIVVGARVARGTWYVIHHYEISQAQGEAGIMQRAFRQATLSQ
SLIDGRMEVKVKVVTSDRYA-
TYEIKGGQHRKGTRFHRVKMGHNIPVEILARIKLAFGDPNLILVKTFGAVVQKRFAQPLTHIRHGAVTIG
PMEVASPYITMKKARHAPGDSVVDVKPDNIVRSVAVVPELIWARENEDNSAVHDKYVKTTHKTAAK
RNRAVDKAQYDKRSQVHLSFIGLAVKVLPEQAIVHATAGTLLPTESVAVTENLKGVAATIRLDPMEKA
LIAYIKRMRPQLNLGGKTDKKIVKCLNNGLDDVEYGKLGRRKIKPITIKSVYDVEHREATELVKVMPYKD
DVESRKSQFAEK--
AIFEPLKAFMMLFGFIARAVYALYLRVVRRTILLAKTKIVVNANTVLSAIKIIGVSIRLRHKSTNIADTKHHI
KTIEARARMTLAGTPSVKLNLREGKVGMKAGRIKGYMPYKNFNIFEGLASGRRAKVKDLAKLKKVDKTI
SVDGIRTRQ-
GGRIPKAQLSNALANTRVGVDIVGENTQEESNPRANAEMGQVFAAIDARVYSLVAALRGHALVAVL
AVCAIQGIGRAHSLLAVY-KINAGIEFHSAA-VETAVAAA-
YAQPGGEAAALWDVSEADLGDISLTEASLDVVRRPRRSLGELKKTKKEAYMSLTCFGGKGDKVKIVEA
TQTQAYEIVATRTAERFGIAAKGTYGKERAMMQITPTKPVKVWVPVFVSEDELLAPIRAFAETNGDGV
TVVKLTKDIKVTMFEKAVASLAVAGKSTSVVYTVLEKVTELSKDIIYDQRAKAHYGRGRLRSHVVTHTK
QKRHLSMIKRTVVASPKMDAARLHDGEVTVITGATMAFVMSEQDAV--
GDVDMHFHEEPRREAALGIAGPETFLATEQPRVLALLLLREYLIHIVDIETVGGFADAATIFDILRAMQN
KLDRKLSAEIAKVRMCLHDDVADMGVKAEAPRREAAGDPILSAYWINGVTASSDIILLVANLECHTAP
ENIDCAVRLFYQRITLQAKERGGDVSVALVPAQNQIEAAEGTVIYTIKGKTPSTVQMKLNIEAISNSIVK
SLAVQLFQVELFLNDTWIAVLLRAAEEVVFVDSGEGREAVDHVVERDTELEMAPQKDIVRRKIRIYTPA
APSAVVIWDPAIERADKADAQGTILKIYGPDLQDIACFTDIGAEPKIESLKAWDEKERYVVHRDDIPPDI
TTLVGKSSRVNKEFGIPDVAVCAPSKPGV-
ESIDRGEPKAGSCGEVANLPYPQDSLVNRVSSANVQKNISMVLGGHPVGEMGISRGVERHLETNMC
QIFVDKDDVDVALDFYKLALTPLRVGLDSPNAKVQIDGKESRKLMAVLDGEAKLQRHPKVSTAAGG-
RKAGMKARLPDDALIFDGVHHGDIKFTATDLPEGRV-
VTPVFNNVKAQLEIDKVILKTFHVVTLSFSNDKGEEHLIKLRLNALKKSTRHYEMLLKIRDTECATSPHAI

PVGKILKIDSLLQTICQAHYKAIEPLLAEDLTALGVLGDQGLLHIVPRLMILAVLTLWGLEAALEHRRW-
LQATRLVTDQTMGLAVRERHWSF-AISEVVGNTFFARTKDDDTAEQLHARNPI-VIL-YGKEGMV-
ISPSGLKLASTKPSPTGHGNENRLVLSTERLQDSMSYFVLKVLLVFVDEMLVAWGSGEPPLKFLVAEQA
ITGRANPQLVCLTRRTFPLDLQRHAVFVMVFEDSTMKNLSHKKMHMNLVLIKSKGWVVNVSTKILIS
DQAIASYAEDAGAHTQPSESALAIITSRVIAPIVLVISQKPAAPSKTMFLEALNLLDEITVTVQNKPKRYYI
FEGDVVAVLGGLDAAVLLGMQRPQPARMRRAVVAAAIRLAIALRGSGVVAQLEGGVLNFARAVSLA
A-VYPDLDF-TGAAIE-RAIAFRVRNHKTSTKRDRPYKGHPKVLHKAEAEKSLVADGVDTLA
>GCA_900172275
AVIKPVTQDAYAPVLMIRQDQVATVSGAGSPSLRAKTVYAKSGLQRKVATKWVAAPFYY-
GVVKMGNIVQLVSISVDNRAVCTVAQLVEQEVVVHGMVALDAVKKKIALDEIDGMTSEDPAKVVQY
AVDIVLKAYRDADDRADLLPVLLGLTLKIAFDPHEPVVHDLPIPVIYEVV-
GIMAQKGTIEVDDCAISTAVSDVDPIAYVSPIDLAAVAGTEMAFIDQEAAVANARETVEL--
LALEASILVVGIKPLLVPI-FEKDG-T-MSFANRSRELKSIVDLAVAYATITRARIELEFVAEM-
MDRAKAAALSEKRGTAEEIRIVKRMQKSSGLADDPDLVMLEDAAEGNSSWAGQIYDDPISGSSESDS
VGNIVHAANKRQNFERSITYLRKAEISDKAAAPLMRGVKHNAKSRVARKLDDTIAALNFPNVPEGIAIA
AAGKIPEHILLHDAAEGEEILLLIVRWHVTWKTAIFVVLLPLAGNIVGLIELGGIAAVGKMDGVLNRHHK
LNGSLIEQRPIIEKMTLRLRASLKQKLGAEQIIIFVVRVAGDLIAEAKVQM---
NVGAVVAIQGETIFVRRHKQTVITDQEATV-VLQGVAEGPADAAALLRIRVDHTLVKLTVLLIPE-
PVLVESVTIREGVGKALQIIQLDVYLLRDMGTGDQTLAKSKSRELLREVLQVDPITGVRLAFGKIKERLTK
GMKLKDLD-----
LSLLAGVTEPRMENLPEGVRRDFVEAGPRAAVGMVFLLLDEAGRVYSIDGDLRELTKSIKSLIGLVDAA
TPKILSAGYAATRLKIRYADCSVLVSDADLYAYAEERRELLRMVPVYIVKAAKRRQLVIFALSLRGGHRR
ARASLQVLLPATILESQAEEGTVDHEEPEVLLDDAEIVGSEMMISRQSALDLKQILKVDVVVLFPEFQEK
MVVSLRFFCTEVAIFVIALITTVGEMLSREPEHAQEEGAGYQKRIRKHSVLFALVCTFDRRNARAA--
PDLFLALGLAEVRYGILYTNQPKKKQMPSEPDGDRIDGFELVHAIHNDGSSNHELEFGEAMALAVE-
DATINVTAGVLALNGGMVAVEKVDSKRKVAVAAEKWNIAPLNVEDITISGADEIRCLN-
IESADLSAALQKKDI-
FLFLPGANNKELEEGKLDGAGNSSADAAEDRQTAILIVTPVLVTIQDSNTASLIANGGVEEWGTMAYK
KHYAYAARMRLHMRTID-------
MEPNKQKSEKVKTVKLEGDTRFRQLATEGVKEQMPVLHALNPDFAVLAPVLVTPAVGQVAADENVL
GARVPCDFSL-RELQKSRKGTALPISAMRLLGVRKARAMIEEGIMVAGKVAA-
LLKIAALETITKLVLRNDTEATLPWRLSVAERLEADTKRAGGFASRPFMSKPSLCNTQAHANVADPIETI
AAGYVATKERFVFPVEGIAIQLILASPHSRVGGAKRAGEGHLHEMAKELMAKAVLM--
GEPESDTEGLLNYHTTNQPTDLLLTDCLDILIDLRRMTIV------------------------------------
--------VQLTTV-HVVVAGVRDVGSYEIMAAAA-
SGLVQVERIDDLTGRERTLMWMYGAMEELWLLSLRYADWVIDVPIGAIILHVYAQAINRAFSLARLQI
DGWPPEADRWWNTWQLWLNKDGFFSYEAAFRAVKAVLKQELADFDMSDTDGVGRYLSRQEIRAG
YRIRQQVTFKRLDPRTDILLQSKTRFAIIRHALDMVAILVIREIAPEFT-
VVAMQDVAMGHVECVLADCFDFTAKNADIGPVGLKTPDNIFGDGTVLKEAVLGKIDLKNTVADFRY
RFIIDGGRELGVDWIGLQNMLYARIWVPPAQAALKNLTPICMFLSHPELDLLLE-
SLFDGISKARADQLMAGVLIEEILDGIARFAHPEATTHQSNKEKHFKYYGEEVDPVHLAYILEIFALVAT
QVTVSDRLLKDRYVLNLQIFRSLTSLLWMALQLAEKGPAATVITLGTIAGLGALVVFIILLMRDSVLVETL
YFRYVY--MRGYSAAKDVTDADPEHLL-LAHASRDVEWIEIIPAFFYYFVTATSRR-
ENTAVAKEFFGLFKERLRIVDVLVVPPERVDDLVDFVALAGTLVTRADALPGLIATGLCEPNAAAVLEA

DQDWNLHRSMGITLAFYKAQESVKGWFDKGLHKDVATISLSEAEGFTTPFHAGAQANNCAPAIHRV
IDLGEVEASLIEIDSAVVVADEEQTLADKPTTRDNSVNAVGGVVLAGTMNMGANLDSVERGGNIIIEL
EEETYNAVNGKKADGKLTNKVLMVISAAFFVVMKRLASANALDKGAVDLLIDAPDDADNLDHGRTM
TIPAPTVHGETTPLYAESEIERIAWVAPIHMLGTLPAVLEFEPDQYSFISMKIEASLEIMSTAEVLDDYHVL
DKGSRPSAFRVVLIRGLHIRQLAGIGTIIVVGSAGRTEVTAYIEG-
AVPITLNKNTFLPAFAASSCHAIDIMSTMLRPPERSTIVTMILETVAEL-
LEVTRFSNDKLERLKLLLPGLDMAEMLTLVAAAKDFAEIVTIDAAITAILSVAAEAVVLREMLLTLAEIAV
SALALALALLLMLTPDRGLTQKEAAREEQMKELIQGGVVLQVRAADRMVLASTADKVRLSTDEGTTS
AFASLNLATVSDGFYVLSSAVLQMSSDLMMVVAVARVLADKETKNALVVRHPEQVKTQPTYAEGSP
GMVLQTLTGHIPPLAIKPEDVELLWTLVAALWRVKAHLAQATRPHGERVLVLLLALALIVVLLQSEGG
GIGGGAMGAAAKLLAAFSVLTLKASVGDEVRKVARRVMDLNSRLGRPEEAVNPEFRGAELEQTKYEA
ENVEVDCLETVRRFVDIPFLVSIRTQHNESEDRGARLVLDVTRVRFIDL-
VTKKSEIILVERAETDGVISREMSKDLQALHPEAAALGKVAALQHYVAISDGHISNYVTADIHSVLGMLI
LVLFIQQVRVGKVVWPTRREVTMVLIMAAVALFFLVIGL--
QLVFMLGGISWQQLTTLVLGEGVMAVRQTSTFPKDS-
IGILQNTYADLICAGRVTKADIEASLHIVVGARVARGTWYVIYHYEITQAQQEAGIMQRAFRQKTLSQS
LIDGRMEVKIKVVTSDRYA-
TYEIKGGQHRKGTRYNRVKVGHNIPVDVLPRIKMAFGPPNLILVKTFGAVVQKRFAQQLTHMRHGAV
TIGPMEVASPYIKSKKARHAPGDSVVDLKPDNILRSVAVVPELIWAREKEDNGAVHDKYTKTTHKTAA
KRNRAVDKAQYDHRNQVELSFIGLAVKVLPEKAIVEATAGTLMPTESVTVTEGLKGVAATIRLDPMEK
ALIAYIKRMRPQLNLQGKVDKKIVKCLNNNLDDVEFGKLGRRKIKPLTIKSVYDVEHEQATELVKIIPYK
DDVEGRKSQFAEK--
ALFEPLKAFMMLFGFIARAIYALSLRVVRRTIILVKTKIVVNAATVLSAIKIIGVSIRLRHKSTNVADTNHTI
KTIEDRARMTLAGTGKVKLNLHPGKVGMKGGRIKGYMPYKNYNIFDKLASGRRAKVKELAKLKKVDKE
ISVDGIRVKQQGGRTPQADLSNAIANTRVGVDIVGESTQEESNPRANAEMGQIFAAIDARIYHHVAA
LIGHALVAILAIVDILGIGKAHALLTDALKINAGIELHSAA-VDTAVAAA-YAQTGGEA-
AIADVSEAYLGLIRITAESLDVCRLKRRSISELKKTKKDAYMALTCFGGKGDKVKIVEATQTQFYEIVATR
TAERHGIKAKGTYGKERAMMQITPTKPVKVWVPIFVNEDELLAP-
RAFAETVGDGVTVVKLTKDIKVTMFEKAVASLSVAGKSTTIVYTVLEKVTELSKDIIYEQRSKTHYGRGRL
RSHVVTHTKSRRHLSMMKRTVVAAPSLDAARLTDGEVTVITGATMAFVMSEQDAV--
GDVDLHYHEEPRREAPLGIAGPETFLATEQPRVLALIFLRAYLIHLVDVGTKGGFADAATIYDILRAMQ
NKLDRKLSADIAKTRMCLHDNLADMGVTELAPRRDSAGDAILSAYWINGVTANVDIILLVANLECGC
APENIDCAVRLFYQRVTLQAKERGGDVSVAVVPAQNQIEAAEGTVIYSIKGNTPSSTQMKLTVEGISR
SIVKSLAVQLFQVNLFLNDTWLGTLVRAAGDVAYVDAKGGDEAVDHVVERDTELEQDPQKDIVTRKI
RIFTPAAPSAVVIWDPAIEREDKADAQGTIIKIYGPNLQDINCFTDIGAEPKIESLKSWDEKEKYVVHRD
DIPEDI-
TMVGKSSKVNKEFAIPDVAVVAPAKPGVTESIDRGEPKAGSCGEVANMAYPQESLVNAVSSANVQK
NISMVLGGHPVGEMGISRGIERHLETSMCEIFVDKNDIDVALDFSTLKLTPLRVGLDPPNAKVQIDGKE
TRKLMAVLDGDADLQRHPKVSEATGIVKTAGTKARLPDDTLMVDGVQNGDIKFTITDLPGGRV-
VTPVFNAVKAQLKIDKVMLKSFHVVTLSFSNEKGEEHLIKLRLNALKKSTRHYEMLLKIRETECADSPHA
IAVGKILKIDALLQTICQAHYTAIEPLLADDLTALGVMGQQGLLHVVPRLMILAVVHVWGVDAEAFIYK
W-IPAEAIMTDVTMGLDQRDRDWTY-CISEVVNNTFFARTAADDTTERLHARNPI-ILL-YDKFGMT-
ISPSGHKLASTAEADLKHGNTNQLVLSAERLQDDMSYYVLQVLLVFVDEVILAFGSGDEPIRFLVAEQA
ITGRENPGMVCLTRRTFPLDLQRDATFVLVFEDSTMKNLSHKKMHMNLVLIKSKGWIVNVSTKILISD

QAIASYAEDAGAHTQPSESALAVITSRVMAPIVLVVSQKPTAPSKTLFLEALNLYDEVTATVLNKPKKY
YIFEGDVISVLGTIDAAVILGMQRPQPDAMRRAIVAAAIRLAIALRASLMVHVLGHDQINFSRAMSFT
Q-AYTDMAA-----------AFRVRNHKVSTKRARPYKGHTKFLHKADMDKNLVAEGVDTLA
>GCA_900172355
AVIKPVTINVYAPVLMIKQQQVATVSGAGSPTLRPKTVYAKSGLQRKTMTKWVASSYYY-
GVVKMSKIVQLVSISVDNRTVCTVAQLVEQEVVVHGIVALEAVKKRIALKEIEGMTSEDPAKVVQYEPE
LELKRFRDAEDRADMLPVLLGLTLKIRFDPHQPLVHDLPIPVIFEVV-
GIMVQRGQIEVDECAVSTAVSDVDPIAYVSPIDLEVVAGEGIAYIDQETAIAAAKDAIALEELALESTVL
VVGI-PLILPI-FRDAGRR-MSFANRSRSLESIVNLAVAAERLFRARIE-PMISAL-
MREARASALATKRGTAEEIRIVKRMQKSAGLADDPDLVILEDAAEGNSSWAGQIYDDPLSGSSESDSA
GNIVHAANKRQNNARSITELRKVEISNQAAAPLMRGVKHNAKSRSSRKLDDAIAALNFPNVPEGIAIA
ATMGKTPATLLHDALEGEEILLIIVRWHVTWKTAVFIVLALRAGDVVGLIELGGIAAVGAPDGILNRHH
KLNGSLIEQKRIVDKLTLPLRAALKQKLGAEKVIILVVKVAGDLVADATVQM---
GVGAVVAIQGVTIYVRRHRQTIVTDSDATVEVLRGVAEGPADAQALLRIRVDHTLVKLSVLLIPE-
PVLVESVTIREGVGKALQIIQLDVYLLRDMGTGEDTLAKSRSRELIRDILMVDPITGVRLAFGHIRERLSK
GLTIKELKTLL--
QAIMRDVTEPRMATLPDGINRSLIEAGPRAAVGTLFLVLDDAGRLYAISGDLRELTKSIKSLIGLVDAAV
PKVLSATYATDRLRIRYADVTVLTSDAGLYAYAEERREILRMVPVYITTAAKRRQLIIFALSLKGGHRRAQ
AALGVLIAAALRESQAEKGPVHHADPAILLDDAEVMGSEMMISRQASLDLKAVVKVDVIVVFPEFQEK
MVVSLRFFCTEVAIFVIALIVTVGAMLSREPAHAQEGGAGFQKRIRGHSILFALAATFERANARDA--
ADLFLGLGLCPPRYGLFYTLQPKRKQMPTEPDEEKVDGFELIAVVKEDGRSNHELEMPEAMALAVE-
DAPINVTAGVLALSGDMVDLERVDSKRKMALAVTKWTIAPVAILDLHLSGARPMRALD-
IELAPVSAALSSKEIATLFLPGANNAELEEDTLDGAGASSADAAEDRQTAILLVNPVLVVIQDSNAAGL
VTNDGVDEWGTMAYKKHYAFAARMRLHMRTID------
QMEPNKQKSEKVKTVKLEGDTRFRQLAVEGVKEQMP-
LHALNPDFAVMVPVLVTPAVGRVAATSAIVGAGVPCGDSL-
RELQKSQKGDALPISAVRLLGMGAARAMVEEDIMVAGKVAA-
LLRIGALEVVAGFVLFNDSEATLPWRLSVAERLESDTKRASGFAARPFRSKPSLCNVQAHAAVAEPIREI
AAGYIAAGGRFVFPVEGIPIQLILASIHSRVGEAKRAGEGHLHEMAKELVAKAVLMVKSEPGSNTEGLL
DYITTGQPTDLLFKDCLDIIIDLRRMNII--
IQLNTV-
HIVIPGTRELADREIMAANADSGLVQVERVDERTGRERTKMWMYGAMEELWLMSVKYAEWVIDTPI
GAIILHVYAQTINRAFSLTRLQLEGWPPEADRWWNTWQLWLNKDGFFGYDAAFRAVRAILRADLYD
YEMSDVEGVGRYQSRQELRVGYRIRQEVEFARLDPRTDILLQSQTRLSVIRHALKLVAILILRQLVAEFT-
VVAEEDVAMGHVECVLADQFSFTDKNAHIGPVGLRTPAPVLGDGTVFNAAVLGKIDLENTIADFRYR
MITDGGRRLGVHW----NMLYARIWLPPAAAALKNLAPIAFFLGHPALDLRVF-
PLFQGISKARADRLMAGALIEEILESVARFADPEATTHQTHKERHFKYYGEEVDPVHLTYIFQKVKLVAT
QVTISDRLLRDRYVLNLKVFRGFTSLLLLAIQLGTKGPSATVITVDTIMGLGALVLFIILL-
RDVVLLETLFYRYVYH-MRGYEAAKDVADADPEHLLSLAQVSRDLEWIEIVPAFFYYFKTAG-
FEPFNTAVAKEFFGLFEERLRIVDVLVVPPSRVEALHELVAFAGTVETAAEALEGVIACGLCEPNAAAVL
EADQDWNLHRHLGITLAFLEARVSVKGWFDKGLHKAVAQTGLSEAGGFTTPFMAGEAAQKCVPVV
HRVLDLLGMEAALIEIDSAVVVADEEQTLADKPTTREKAVGAIGGVVLAGTMNMGANLEQVERSGNI
IIELEEEHYNAVNGKKADGKLTNKVLMVLSAALFPVVARAASATAMDRGAYDLLIEAPDDVANLDHG
RDLSIPAPTVHLETTPLYAESEIERIAWIAPLHMLGTLEAVLGFEPDQYSFISMMIDASLDITETAEV--

DYHVLEKGGRPPDFRVILIKGLHIRVLAGISTIIVVGSAGRAEVTAYIHG-
AVPITLNKHTFLPAFAASSCHVIDIFSTMLRPPERSTVQMLILETVAEL-LEVTRGENDKLERL-
LLLPGLDMAALLALAAAAVDFAAIVTIDAAIVAILSVAAEAVVLRELLLTLAEIAVTALALAMALLLMLTR
DQGLHQRERAREAQMKTLIQGGAVLQVRAADRMVLAATADKVRLDTDEGTTSAFASLNLATVSDG
FYVLSSAVLQLSSDLMMIVHVARVLADKEHKNALVVRHPEQVKTQATYAEGSPGMVLQTLTGTIPPL
AVKPEAIELLWTLVAALWRVKAHFASAMRPRLERVLVLLLALTLIVVLMQSEGGGMGG--
MGSAAKGLAAFSILTIKASVGDEVRKVTRRVMDLNGRLGRPEEAVNPEFRGAELEQSKFEAENVAVDT
METIRRFVDIPFLVSTRTQHNESEDRGSRMVLDVTRVRFIDL-
VTKRSEIILAARAETDGIISREMQKDLQALHPEAAALGKIAALQHFIALNDGHISNYVTADIHSVLGMLL
LVLFVSQVRVAKVVWPTRREVTMVFLLAAVAVFFLVIGLGVQIVFMLSGISYNALTTLVLGEGVMAVR
QTSTFPADS-
IGILQNTYADLICAGRVTKADIENSLHIVVGARVARGTWYVIYPYEITQAQAPAGIMQRAFRQATLSQS
LIDGRMEVKIKVVTSDRYA-
TYEIKGGQHRKGTRFQQVKIGHNIPVELLPRIKMAFGPPNLILVKTFGAVVQKRFAQALTHMRHGAVT
IGPMEVASPYITAKKSRHAPGDSVVDLKPDNIVRSVAVVPELIWAREHEDNGAVHDKYVKTTHKTAA
KRNKAVDKAQYDHRNQVELSFIGLAVKVLPEKA--------------------------------------
--
RMKPQLNLGGKTDKKIVKCLNNNLDDVEYGKLGRRKIKPITIKSVYDVEHRKGTELVKLLPYKDDVESR
KSQFAEK--
ALFEPLQAFMMIFGFIARAVYALHLRVVRRTILLVKTRIVVNANTVLSAIKIIGVSIRLRHKSTNVADTNH
YIKTIEKRARMTLAGAGKVKLNLRPGRVGKKGGRIKGYMPYNNFNIFDGLESGRRAKVQDLAKIKKVD
KEVSVDGIRTKQQGGRQPKADLSNAL-
NTRVDVDIVGENEQEESNPRANAEMGQIFAAIDARVYHLVGALIGHALEA-
LAVADIWGIGKAHALLGDKLKINAGIEFHSAA-VERAVAAA-YAQPAGEA-
ALADVSEAYLGALLLTEQALDVCRLRRRQLSE--
RHGIKAKGTYGKERAMMQITSTKPVKVWVPIFVNDDELLAPIRAVAETNGDGVEVVKLDNDIKITMFE
KAVASLAVTGKSSSLIYTVLEKVTELMKDIIYEQRAKAHYGRGRLRSHVVTHTKQKRHLSMMRRTVVA
DPKMDAARLTDGEVSVITGATIAFVMSEQDAV--
GDVDFHFHEEPRREADLGIAGPETFLATEQPRVLVLLFLRAYLIHIVEIDTVGGYSDAATIYDILRAMQN
KLDRKLSADIAKVRMVLHDTVSHMGVRTEAARRDSAGDTILSAYWLNGVTAKSDIILLVANLECRTVP
ENIDCAVRLFYQRVTLQAKERGGDVSVAVVKAQNQIEAAEGTVIYTIKGKTPGTVQMALNVEGISRSI
VKSLAVQLFQVDTFIDDTWIATLLRAAGQVVFVDARDGKEAVDHVVERDTELEFAPQKDILPRKIRIYT
PAAPSAVLIWDPAIEREDKADAQGTILKIYGPNLQDISCFTDIGSEPKIESLTSWDEKEKYVVHRDDIPA
DITTLVGKSSRVNKEFGIPDVAVVAPSKPGVTESIDAGEPKAGTCGEVANMAYPQESIVNAVSSANVQ
KNISMVLGGHPVGEMGIAREVERHLETNMCQIFVDKPDVDVALDFYRLALTPLRVGLDRPNAKVEID
GKESRKLMAVRDGDASLQRHPKVSAAAGTGAKAATKAKLPDDALIHDGVHHGDVSFTATDLPEGRV
-
VTPVFNTVKAQLKIDKVLLRTFHVVTLSFSNDKGDEHLIKLRLNALKKSTRHYEMMLKIRETECAESPNA
IPVGKILKIDSLLQTICQAHYTAIEPLLADDLTALGVMGDQGLLHVVPRLMILAVMHLWGLEAAMELST
WHLMAQCVVTDVTMGLAIRERQYTF-AISEVVGNTFFAMTKDDDTEQRLHARNPI-VLL-
WGKFAMVVISPSGMRLDSTAESALEHGNQEKLVLSPERLQDAMGYYILRLLLVFVDEMILAYGSGKEP
IKFLVAEQAVTGRENPGLVCLTRRTFPLDLQRDATFVMVFEDSTMKNLSHKKMHMNLVLIKSKGWIV
NVSTKILISDQAIASYAEDAGAHTQPSESALAAITSRVIAPIVLVVSQKPTAPSKTLFLEALNLYDEVTAT
VLNAPKKYYIFEGDLISVLGPIDAAVLKGLQRPQPDRMRRAVIAAAIRLAIAGR---

LTARLEAGLLNFSRSISFMR-
EYPQLSPLVRAAMQPGAVAFRVRNHKTSTKRDRPYKGHTKFLHKAEEDKSLVAEGVDTLA
>GCA_900184895
AVIKPVTMPIYAPVLMIRQQQVATVSGAGSPSLRAKTVYAKSGLQRKTETKWVAKPLYY-
GVIKMGKIVQLVSISVDNRVVCTVAQLVEQEVVVHGMVALEAVKKKIALEAIDGMTAEDPAKVVDYQ
GELELKRFRDAEDRADMLPVMLGLTLGIKFDPHQPVVHDLPVPVIFEVV-
GIMAQRGGIEVDECAISTAVSDVDPIAYISPIDLEAVAGDKMAYIDQEAAVSAAKDAIELEELALESSVL
VVGI-PLLLPV-FGEDGDT-MSFANRSRTLESIVDLAVAYPEQTRARISVGIASEM-
MGRARAAALSEKKGTAEEIRIVKRMSKSAGLADDPDLVILEDAAEGNSSWAGQIYDDPISNSSESDSA
GNIVFAANERQNFARSITELRKVEISDKAAAPLMRGVKHNAKSRSNRKVNDAIAALNFPNVPVGIAIA
AAAGRTHHALLHDATEGEEILMLVVRLHVTWKTAIIIALAVTAGDIVGVITLGGLAAVGRKDGILNRHH
KMNGSLIEQKRVIDKLTLRLRASLKQKLGAEQVIIFVVKVAGDLVAEATVQMG--
DIGAVVAIQGETIFVRRHRQTVVTDQEATVEILRGVAEGPADAQALLRIRVDHTLVDISVLLIPQVPVLV
ESVTIREGVGKALQIIQLDVYLLRDMGTGEDTLAKSKSRELLREVLQVDEITGVRLAYGKLKERLVKGMT
LKELDTLF--
LVLVKDVTEPRMENLAEGVCRTLVEAGPRAAVGTLFLMLEDAGKLYTIDGDLREILKSIKSLIGLVDAAK
PKVLSAQYAAARLKIRYADCTVLTSEADLYAYKEERKELLRMVPVYITNAAKRRQLVILALSLKGGHRRA
KASLGVLIAAAIIESQTVEGATHHDEPRVLLEDAEVVGSEIMVSRQASLDLKAVIKVDIIVVFPEFQEKM
VVSLRFFCTDVAIFVIALIVVVGAMLSREPAHAQEEGAGFGKRIRGSAVLFALAASFDKRNAGDA---
DLFLGMGLAPPRYGILYNMQPKKKQMPSEPDAERVDGFDLVNAVKADSSTNHVLELREAMALAVE-
DSPINLAAGVLALKGTMAAVEKVDSKRKMALAVEKWTIAPLPLLDLTLSGASPVRALG-
IDLADVTAALPAKTI-
GLFLPGANNAELEEEKLGGAGASSAEAAADRQLSILLVRPVLVVIQDSSAASLVANGGVEEWGTMAY
KKHYAYAARMRLHMRTIDGIEVGVSLEPNKQKAEKIKTVKLEGDTRFRQLAIEGVREQMP-
LHALNPDFAV-LAVLVSPAVGRVAADQDVLGASVPCDASL-
RELQKSQKGDALPISGIRLLGMGAARAMIEEDIMVAGKVGA-
LLVIRALETIAAFVLRNDSEATLPWRLSVAERIEADTKRAGGFASRPFKSKPSLCNTQAHARVADPIQEI
AAGYVAVDERFVFPVEGLAIQLFMANTLSRIGSAKRAGEGHLHEMAKELKSKAVIMVQNEPNTNTEG
LLTYMTTQQPTDLLFEDCLDVIIDLRRMNIV--
IQLNTV-
HVVVAGVRDVGAFEIMAAAAASGLVQVEQVDDRTGREKSLMWMYGAMEDLWLMSVTWADWVI
DCSIGAIILHVYAQTINRAFSLKRLQLSGWPQEADRWWNTWQLWLNKDGFFSYEAAFRA-
LAVLNADLYDYDMSDVEGVGRYLSRQQLRVGYRIREEVAFERLDPRTDILLQSHTRLSVIRHALTLVNI
LIVRTIVAEFT-
VVAQEDVAMGHVECVLADQFDFTAKNADIGPVGLRTPAPVLGDGTVFAEAVLGKIDWANTVADFR
YKFLTDGGRRLGVSWVKI-
NMLVGRIWLPPAAAALKNLLPICFFLSHPELNLDLLAALWDGISKARADKLMAGALIEKLLPGVAAFA
DPEATTHQSKKEKHFKYYGEEVDPVHLAYILAMFALVATQVTISDRLLKDRYVLNMKI-
RGFTSLLWMSLQLGLKGPSATVITVDTIAGLGALVLFIILL-RDEVLVETLYWRYVY--
MRGYTAAKDVADADPEHLLTLAHASRDVEWIEIVPAFFYYFPTAT-
FEPFNTAVAREFFGLFEERLKVVAVLVVPPDRVEALVEFVALAGTLATSAEALVGVIACAFCEPNAAAVL
EADQDWNLHRSMGITLAAFAAQVSVKGWFDKGLHKDVAATGLREAGALTTPFHAGATAKFCVPAV
HRVLDLGEIEASLIEIDSAVLVADEELTLADKPTTREKSVGAVGGIVLAGTMAMGSALDSVERGNNIIIEL
EEESYSAVNGAADSSKLTDKVLMVLSAAYFPVLARMASANALDKGVFGLLIDAPDDADNLDHGG-

MTIPAPTVHAETTPLYAESDIERIAWVAPIHMLGTLPAVLGFEPDQYSFISMMIEASLEIMETAEV--
DYHVLETGSRPPDFRVTLIRGLHIRQMASIGTIIVVGSAGRSEITAYIQG-
AVPITLKRETFLPAFAASSCHVIDVFSTMLRPPERSTVVTMILETVAEL-
LNVTRIENDKLERLLLLLPGLDVAALLDLLAAAADFATIVTIDAAITAILSVAAEAVVLRELLLTLAEIAVTA
LALALALLLMLTPDKGLHQREAAREAQLKDLIQGGVVLQVKASDSMVLAATADKVRLDTDEGTTSAF
ASLNLATVSDGFYVLSSAVLQLSSDLMMIISVARVLADKESKNALVVRHPEQVKTQPTYAEGSPGMVL
QTLTDCVPPLAVKPQEIELLWTLVAALWRVKAHLASAARPHRERVLVLILALSLIVVLLQSEGGGMGG
G-
VGSAAKILAAFSILTIKAKAGDEVRKVTRRVMDLSQRLGRPEESVNPEFRGAELEQSKYEAENVEVDTM
ETIRRFVDIPFLVSTRTQHNESEDRGSRMVLDVTRVRFIDL-
VTKRSEIILAARVETDGIIAREMQKDMQSLHPEAAALGKVAALQHYISINDGHISNYVTADIHAVLGML
LLVLFVQETRIAKVVWPTRREVTMVFLLAAVAVFFLVIGLTVQLVFMLGGISWQQLTTLVLGEGVMVV
RQTSTFPADS-
IGILQNTFADLIAAGRVAKADIEASLHIVVGARVARGTWYVIYLYEITQAQAPGGILQRAFRQATLSKSLI
DGRMEVKIKVVTSDRYA-
TYEIKGGQHRKGTRYNRVKVGHNIPVQVLPRMKMAFSPPNLVLVKTFGAVVQKRFAQQLTHMRHG
AVAIGPMEISSPYISAKKARHAPGDAVVELKPENILRSVAVVPELIWARENEDNGAVHDKYVKTTHKLA
AKRKKAVDKAQYDKRNQVEMSFIGLAVKVLPEKAIVHATAGMLMPTASVAVTEGMKGIAATIRLDP
MEKQLIAYIKRMRPQLNLAGKTDKKIVKCLNNNLDDVEYGKLGRRKIKPLTIKSVYDVESEEATELVKLL
PYKDDVEGRKSQFAEK--
ALFEPLKAFMMLFGFIARAIYALYLRVVRRTILLAKTKVVVAAATVLSAIKIIGISIRLRHKSTNVAETNHHI
KTLEDRARMTLAGTGKVKLHLHPGRIGKKGGRIKGYMPYKNYNIFDGLASGRRAKVKELAKLKKVDKEI
SVNGIRMKQQGGRVPKPNLSNAIANSRVGVDIVGENEQEESNPRANAEMGQIFAAIDARIYSLVGAL
IGHALEAVLAVVAIWGIGRAHCLMAVAFRINAGIELHSAA-VARAVAAA-
FAQNGGEAAVIADVSECYLGALSISQVSLTVCRLRRRRLSELKKTKKDAYMSLTCFAGKGDRVKIIEAA
QTQAYEIVATRTAERHGISAKGTYGKERAMMQITSTKPVKVWVPIFVTEDELLAPIRAVAETNGDGVT
VVKLTKDIKVTMFEKAVASIAVTGNATSVLYTVLEKVTELMKDIIYEQRAKAHYGRGRLRSHVVTHTKQ
KRHLSMIRRTVVADPRMDASRLTDGEVSVITGATMAFVMSEQDAV--
GDVDLHFHEEPRREAEVGIAGPETFLTTAQPRVLALLFLKAYLLHIVEIETSNGFSDAATIYDILRAMQNK
LDRKLSADIAKTRMCLHDNIPTMGVTELAARREAAGDPILSAYWINGVTANSDIILLVANLETACTPEN
IDCAIRLFYQRVTLQAKERGGDVTVSVVKAQNQIEAAEGTVIYSTKGTQPGTLQMKLTVEGVSRSIVKS
FAVQLFQVDTFLNDTWIATLLRAAQDVVFVQATEGKEAVDHVVERDTELEFAPQKDIVARKIRIFTPA
APSAVVIWDPAIEREDKADAQGTILKIYGPNLQDISCFTDIGSEPKIESLKAWDEKEKYVVHRDDIPEDI
DTLVGKSSRVNKEFGIPDVAVCAPSKPGVTESIDRGEPKAGSCGEVANMSYPQESLVNAVSSANVQK
NISMVLQGHPVGEMGISRGIERHLETNMCEIFVDKADVDVALDFYKLELTPLRVGLDKPNARVDIDGK
ESRKLMAVLDGAAGLQRHPKVSAATGLTQNAGSKAKLEDEALIFDGVQHGDVSFTALDLPDGRV-
VTPVFNAVKAQLKIDKVILRAFHVVTLSFSNNKGDEHLVKLRLNALKKSTRHYEMMLKIRETECAESPH
AISVGKILKIDSLLQTICVAHYTAIEPLLADDLTALGVMGDQGLLHVVPRLMILAVLHLWGVEAEALVP
QW-IQAEAVVTDITIGLRQRERDWSY-AISEVVGNTFFARTFDDDTEQRLHARNPI-VLL-
YGKFDMT-
ISPSGLRLDSTAPTAREHGNDEQLVLSKERLQDHMGYYILKVLLVFVDEVIVAFGSGDEPLKFLVAHQA
VTGRGNPGMVTLTRRTFPVDLQRDPVFVMVFEDSTMKNLSHKKMHMNLVLIKSKGWVVNVSTKILI
SDQAIASYAEDAGAHTQPSESALAAITSRVMAPIVLVVSQKPTAPSKTLFLEALALYDEVTATVLNKPK
KYYIFEGDVISILGRLDAAVLLGMQRPQPNRMRRAVIAAAIRLAIAIR-------

LEEGLINFSRAVDLAVGVYPTLEATVPAVVP-
DAMAFRVRAHAVSTKKSRPYKEHTKFLHKAELEKSLIAAGVDTLA
